# Extracellular Matrix in Heart Failure: Role of ADAMTS5 in Proteoglycan Remodeling

**DOI:** 10.1161/CIRCULATIONAHA.121.055732

**Published:** 2021-11-22

**Authors:** Javier Barallobre-Barreiro, Tamás Radovits, Marika Fava, Ursula Mayr, Wen-Yu Lin, Elizaveta Ermolaeva, Diego Martínez-López, Eric L. Lindberg, Elisa Duregotti, László Daróczi, Maria Hasman, Lukas E. Schmidt, Bhawana Singh, Ruifang Lu, Ferheen Baig, Aleksandra Malgorzata Siedlar, Friederike Cuello, Norman Catibog, Konstantinos Theofilatos, Ajay M. Shah, Maria G. Crespo-Leiro, Nieves Doménech, Norbert Hübner, Béla Merkely, Manuel Mayr

**Affiliations:** King’s BHF Centre of Research Excellence, London, UK (J.B.-B., M.F., U.M., W.-Y.L., E.E., E.D., M.H., L.E.S., B.S., R.L., F.B., A.M.S., N.C., K.T., A.M.S., M.M.).; Heart and Vascular Center, Department of Cardiology, Semmelweis University, Budapest, Hungary (T.R., L.D., B.M.).; Tri-Service General Hospital, National Defense Medical Center, Taipei, Taiwan (W.-Y.L.).; IIS-Fundación Jiménez Díaz–Universidad Autónoma and CIBERCV, Madrid, Spain (D.M.-L.).; Max Delbrück Center for Molecular Medicine in the Helmholtz Association (MDC), Berlin, Germany (E.L.L., N.H.).; Institute of Experimental Pharmacology and Toxicology, University Medical Center Hamburg-Eppendorf, German Center for Heart Research (DZHK), Hamburg, Germany (F.C.).; Instituto de Investigación Biomédica de A Coruña (INIBIC)–CIBERCV, Complexo Hospitalario Universitario de A Coruña (CHUAC), Universidade da Coruña, Spain (M.G.C.-L., N.D.).; Charité-Universitätsmedizin, Berlin, Germany (N.H.).; DZHK (German Center for Cardiovascular Research), Partner Site Berlin, Berlin, Germany (N.H.).

**Keywords:** adrenergic beta-agonists, extracellular matrix, heart failure, proteoglycans

## Abstract

Remodeling of the extracellular matrix (ECM) is a hallmark of heart failure (HF). Our previous analysis of the secretome of murine cardiac fibroblasts returned ADAMTS5 (a disintegrin and metalloproteinase with thrombospondin motifs 5) as one of the most abundant proteases. ADAMTS5 cleaves chondroitin sulfate proteoglycans such as versican. The contribution of ADAMTS5 and its substrate versican to HF is unknown.

**Methods:**

Versican remodeling was assessed in mice lacking the catalytic domain of ADAMTS5 (Adamts5^ΔCat^). Proteomics was applied to study ECM remodeling in left ventricular samples from patients with HF, with a particular focus on the effects of common medications used for the treatment of HF.

**Results:**

Versican and versikine, an ADAMTS-specific versican cleavage product, accumulated in patients with ischemic HF. Versikine was also elevated in a porcine model of cardiac ischemia/reperfusion injury and in murine hearts after angiotensin II infusion. In Adamts5^ΔCat^ mice, angiotensin II infusion resulted in an aggravated versican build-up and hyaluronic acid disarrangement, accompanied by reduced levels of integrin β1, filamin A, and connexin 43. Echocardiographic assessment of Adamts5^ΔCat^ mice revealed a reduced ejection fraction and an impaired global longitudinal strain on angiotensin II infusion. Cardiac hypertrophy and collagen deposition were similar to littermate controls. In a proteomics analysis of a larger cohort of cardiac explants from patients with ischemic HF (n=65), the use of β-blockers was associated with a reduction in ECM deposition, with versican being among the most pronounced changes. Subsequent experiments in cardiac fibroblasts confirmed that β1-adrenergic receptor stimulation increased versican expression. Despite similar clinical characteristics, patients with HF treated with β-blockers had a distinct cardiac ECM profile.

**Conclusions:**

Our results in animal models and patients suggest that ADAMTS proteases are critical for versican degradation in the heart and that versican accumulation is associated with impaired cardiac function. A comprehensive characterization of the cardiac ECM in patients with ischemic HF revealed that β-blockers may have a previously unrecognized beneficial effect on cardiac chondroitin sulfate proteoglycan content.

## Abstract 2

Supplemental Digital Content is available in the text.

**Clinical Perspective**

**What Is New?**

- Left ventricular tissues from 80 patients with heart failure and 6 nonfailing control hearts were analyzed by quantitative mass spectrometry, constituting the largest proteomics analysis on human heart failure to date.
- Proteoglycan accumulation in ischemic heart failure was attenuated by β-blocker administration.
- Accumulation of the proteoglycan versican was regulated by ADAMTS5 (a disintegrin and metalloproteinase with thrombospondin motifs 5) and associated with a reduction in proteins involved in intercellular communication.

**What Are the Clinical Implications?**

- Besides their negative chronotropic and inotropic effects, β-blockers may modulate extracellular matrix remodeling.
- Proteoglycan secretion by cardiac fibroblasts constitutes an important component of cardiac fibrosis after ischemic heart failure, which contributes to impaired cardiac function.

Extracellular matrix (ECM) remodeling is a key pathologic feature of heart failure (HF). Once initiated, ECM remodeling is continuous and contributes to systolic and diastolic impairment.^1^ The mechanisms governing ECM remodeling are not well understood, and there is no therapy for cardiac fibrosis.^2^ Recent genome-wide association studies implicated the family of ADAMTS (a disintegrin and metalloproteinase with thrombospondin motifs) proteases in cardiovascular diseases.^3–5^ In a previous proteomics study, we identified ADAMTS5 as one of the most abundant proteases secreted by mouse cardiac fibroblasts (CFs).^6^ ADAMTS1, ADAMTS4, and ADAMTS5 are key enzymes responsible for the degradation of chondroitin sulfate proteoglycans (CSPGs).^7,8^

Using proteomics in a porcine model of ischemia/reperfusion (I/R) injury, we have previously demonstrated that CSPGs are altered during cardiac remodeling.^9^ Versican is the main CSPG in the myocardium. Whereas collagens mediate force transmission and stretch resistance, CSPGs fill the extracellular space and are important for tissue hydration and signaling. Chondroitin sulfates (CS) are negatively charged, which favors electrostatic attraction of water.^2,10^ Accumulation of CS during pathologic remodeling can be detrimental for cardiac function.^11^ Moreover, versican interacts with both hyaluronic acid (HA) and the HA receptor CD44.^12,13^ The interaction between HA and cells is key during remodeling after myocardial infarction. For example, migration of fibroblasts and inflammatory cells is driven by CD44-dependent pathways.^14^

In the present study, we extend our proteomic analyses of the cardiac ECM from preclinical models^9,15^ to patients with ischemic HF and explore the involvement of ADAMTS5 in cardiac CSPG remodeling. Cardiac function was assessed in mice lacking the catalytic activity of ADAMTS5 (Adamts5^ΔCat^). We demonstrate that excessive versican deposition impairs cardiac function and alters proteins implicated in intercellular communication and that use of β-adrenoreceptor antagonists (β-blockers) is associated with reduced CSPG accumulation in patients with ischemic HF.

## Methods

An expanded Methods section is available in the Supplemental Material. Proteomics datasets have been deposited in a publicly available repository as indicated in the following. All other data that support the findings of this study are available from the corresponding authors on reasonable request.

### Human Cardiac Tissue

Human cardiac tissues were obtained from the Transplantation Biobank of the Heart and Vascular Center at Semmelweis University (Budapest, Hungary) and A Coruña University Hospital’s Biobank and Advanced Heart Failure and Heart Transplantation Unit (A Coruña, Spain). Cardiac tissue from patients with end-stage HF was obtained from explanted hearts during cardiac transplantation. Control tissues were obtained from unused donor hearts. Written consent was obtained from all patients enrolled in the study and institutional approvals were obtained at all source institutions (see Supplemental Material). Internal Research Ethics Committee approval was obtained at King’s College London for the use of human tissues (REC LRS-17/18–5080, REC decision entry 17440).

### ECM Protein Extraction and Proteomics Analyses

ECM protein extraction was performed using our previously published method, involving sequential incubation with 0.5 M NaCl, 0.1% sodium dodecyl sulfate (SDS), and a final incubation with 4 M guanidine hydrochloride (GuHCl). GuHCl extracts were enzymatically deglycosylated.^16^ Liquid chromatography acquisition was performed as previously described.^16^ Label-free tandem mass spectrometry (MS/MS) analyses on GuHCl extracts were performed on a Q Exactive HF Hybrid Quadrupole-Orbitrap mass spectrometer (Thermo Scientific). SDS extracts were labeled using tandem mass tags (TMT) and analyzed on an Orbitrap Fusion Lumos Tribrid mass spectrometer (Thermo Scientific). MS/MS data were deposited in PRIDE (PXD024135, PXD028906, PXD028908, PXD028887, PXD028942).

### Animal Experiments

All animal procedures were performed by authorized researchers. Housing and animal care was in accordance with the UK Animals (Scientific Procedures) Act 1986 and institutional ethical approval. Male Adamts5^ΔCat^ mice (ie, lacking the catalytic domain of ADAMTS5; JAX stock 005771, B6.129P2-*Adamts5*^tm1Dgen^/J) and Adamts5^+/+^ wild-type littermates on a C57Bl/6J background were used at 10 weeks of age for angiotensin II infusion.^17^ Murine CFs were isolated from 4- to 7-week-old C57Bl/6J mice.

### Statistical Analyses

The limma package was used for all statistical comparisons using the Ebayes algorithm and correcting for age and sex. Correlations were determined using Spearman correlation. For functional experiments, *t* tests were used for comparisons involving 2 groups, and 1-way or 2-way ANOVAs with Bonferroni corrections for comparisons involving >2 groups. Label-free MS data were normalized using the total ion intensity. TMT data were normalized against the reference channel to remove batch effects. The relative quantities of all proteins were log_2_ transformed. All values are summarized as mean±SD or as percentages unless otherwise specified. For all significance testing, a 2-tailed *P*<0.05 was deemed significant.

## Results

### ECM Composition of the Scarred Myocardium in Ischemic HF

**Figure 1.**
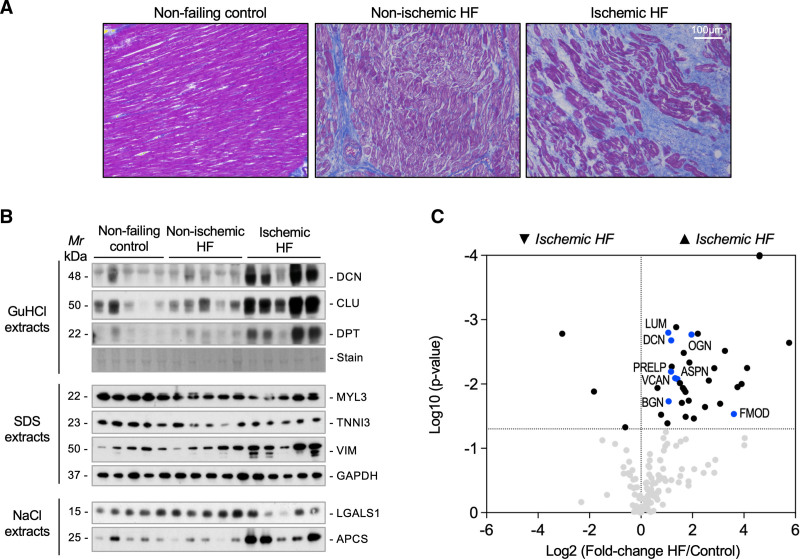
**Extracellular matrix remodeling in human heart failure. A**, Histologic characterization demonstrates diffuse fibrosis in patients with nonischemic heart failure (HF) and a pattern of focal replacement fibrosis in patients with ischemic HF. **B**, Immunoblotting for extracellular and intracellular proteins in 3 different extracts. Extracellular matrix proteins in the 4 M GuHCl extracts: PGS2 (decorin), CLU (clusterin), and DPT (dermatopontin); cellular proteins in the SDS extracts: the cardiac proteins MYL3 (myosin light chain 3) and TNNI3 (cardiac muscle troponin I) as well as the fibroblast marker VIM (vimentin) and GAPDH (glyceraldehyde 3-phosphate dehydrogenase) as loading control; soluble extracellular proteins in the 0.5 M NaCl extracts: LGALS1 (galectin 1) and APCS (serum amyloid P-component). **C**, Volcano plot comparing extracellular matrix proteomics profiles from patients with ischemic HF (n=5) with controls (n=6). Extracellular matrix accumulation in patients with ischemic HF includes the proteoglycans ASPN (asporin), BGN (biglycan), DCN (decorin), LUM (lumican), OGN (mimecan, osteoglycin), PRELP (prolargin), and VCAN (versican). These proteoglycans are highlighted in blue.

Using proteomics in a porcine model of I/R injury, we have previously characterized the ECM in the focal injury and the border region after myocardial ischemia.^9^ In the current study, we applied a similar proteomics approach to patients with HF of ischemic origin (n=5, all male, 62.8±6.6 years). Patients with HF of nonischemic origin showing left ventricular (LV) dilatation (n=10, all male, 50.8±10.6 years) were used for comparison. Donor hearts discarded for transplantation were used as controls (n=6, all male, 49.6±2.1 years). All samples included in this comparison were obtained from A Coruña University Hospital. Diffuse fibrosis was observed in nonischemic HF; patients with ischemic HF displayed focal replacement fibrosis. Representative histologic images are presented in Figure 1A. In nonischemic and ischemic HF, a reduction in myofilament proteins MYL3 (myosin light chain 3) and TNNI3 (cardiac troponin I) was observed. In ischemic samples, this was accompanied by an increase in the fibroblast marker VIM (vimentin) and ECM proteins DCN (decorin) and DPT (dermatopontin), as well as a deposition of serum proteins, such as APCS (serum amyloid component P; Figure 1B). Similar to our previous proteomics analyses, LV tissues were subjected to a 3-step ECM extraction procedure: 0.5 M NaCl followed by 0.1% SDS for decellularization and 4 M GuHCl for extraction of core ECM proteins.^9^ A total of 200 ECM and ECM-associated proteins were identified by MS (Table S1). Besides fibrillar collagens, interstitial proteoglycans accumulated in the myocardial scar of patients with ischemic HF. These included members of the SLRP (small leucine-rich proteoglycan) family and the large aggregating proteoglycan VCAN (versican) as main CSPG (Figure 1C). Key findings of core ECM proteins in our previous analysis of a porcine model of I/R injury^9^ were replicated in patients with ischemic HF including a marked increase in VCAN (Table). In contrast, few changes in ECM abundance were detected in patients with nonischemic HF and none involved proteoglycans (Figure S1A and Table S1).

**Table.**
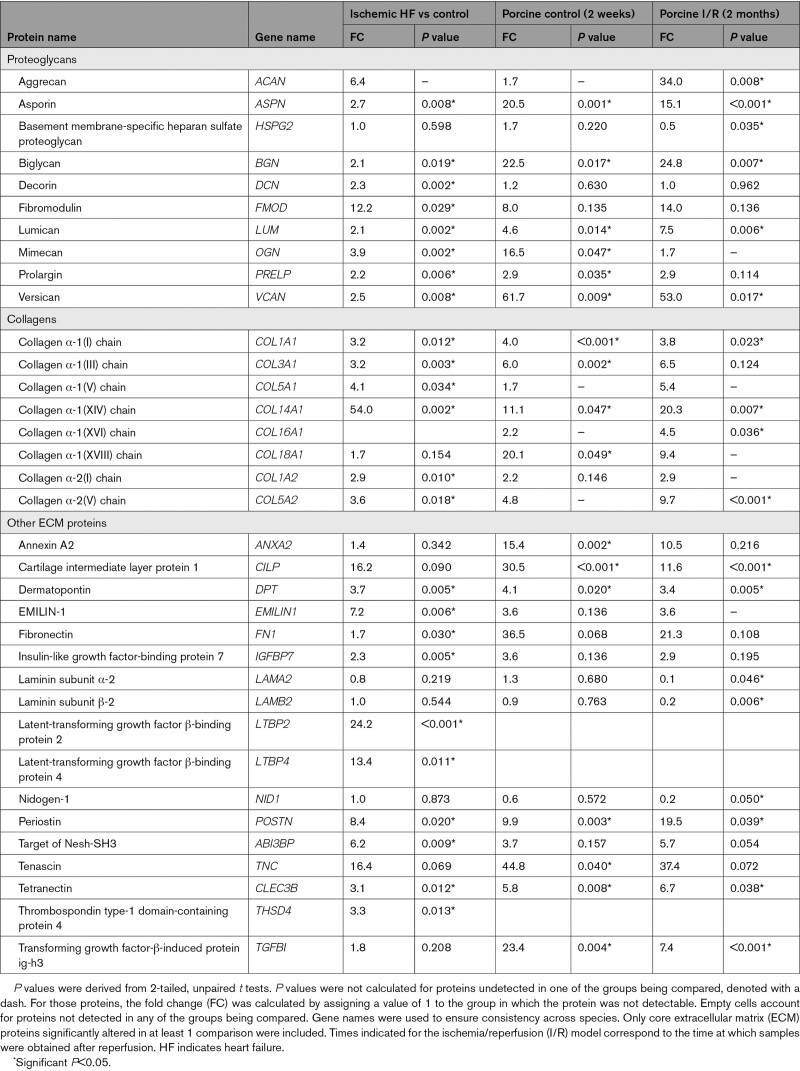
Extracellular Matrix Remodeling in Ischemic Heart Failure: Comparison With a Previous Proteomics Analysis of a Porcine Model of Ischemia and Reperfusion Injury

### Versican and its ADAMTS-Specific Cleavage Product Versikine

**Figure 2.**
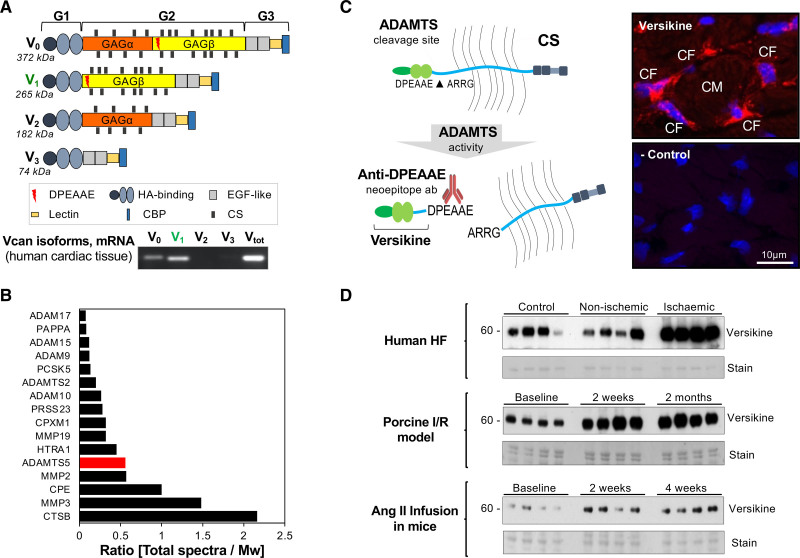
**Regulation of versican accumulation in the heart. A**, Versican can be expressed as 4 isoforms (V0–V3). Versican V1 is the predominant cardiac isoform. GAGα and β are 2 glycosaminoglycan modules. **B**, Our previous proteomics characterization of the secretome of murine cardiac fibroblasts (CFs) returned ADAMTS5 (a disintegrin and metalloproteinase with thrombospondin motifs 5) as one of the most abundant proteases in the secretome of CFs. **C**, Versikine is generated on cleavage of versican by members of the ADAMTS family and can be detected by an antibody against the versican neoepitope DPEAAE. Versican is expressed by CFs, and versikine is detected in areas surrounding cardiomyocytes (CMs) in mouse hearts. **D**, Immunoblotting for versikine in left ventricular tissue from patients with heart failure (HF) with ischemic and nonischemic origin, in a porcine model of cardiac ischemia/reperfusion injury (I/R), and in a murine model of angiotensin II–induced cardiac hypertrophy. CS indicates chondroitin sulfate; EGF, epidermal growth factor; and HA, hyaluronic acid.

In both humans and mice, versican V1 is the main cardiac isoform, followed by V0 (Figure 2A and Figure S1B). Several members of the ADAMTS family, termed hyalectanases, cleave versican. In our previous proteomics analysis of murine CFs,^6^ ADAMTS5 was the only hyalectanase identified in their secretome (Figure 2B). ADAMTS-mediated cleavage of versican generates an N-terminal fragment, versikine,^18^ which can be detected by a neoepitope antibody (Figure S1C–S1E). The abundance of versikine is a surrogate of hyalectanase activity as well as substrate availability. Versikine staining was observed in the pericellular region of cardiomyocytes in murine hearts (Figure 2C). Versikine levels were higher in patients with HF of ischemic than nonischemic origin. Likewise, versikine was abundant in cardiac tissue from pigs subjected to I/R injury and from mice after angiotensin II infusion (Figure 2D and Figure S2A).

### ADAMTS5-Mediated Versican Cleavage

**Figure 3.**
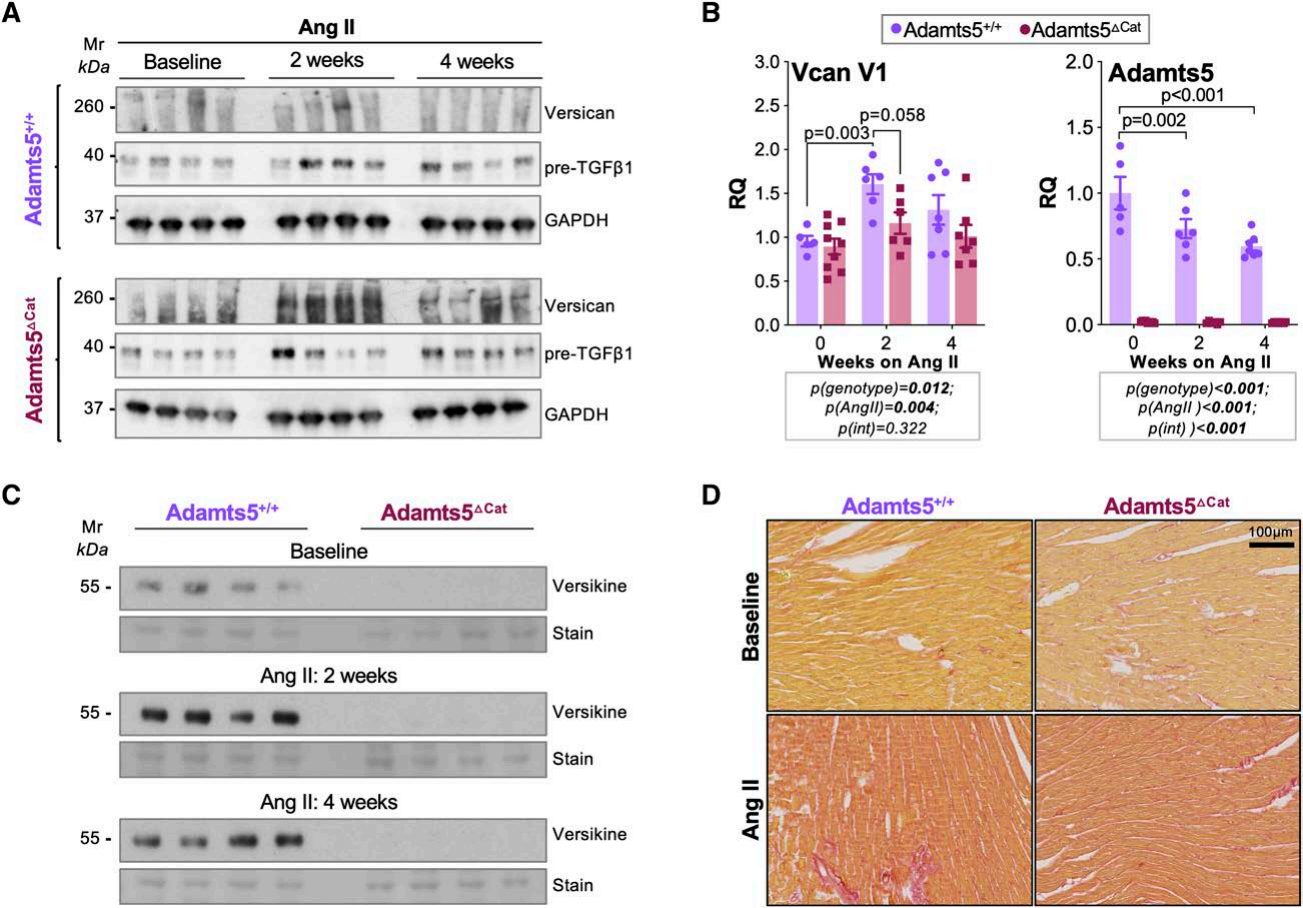
**ADAMTS5 is responsible for versican cleavage in vivo. A**, In mice lacking the catalytic activity of ADAMTS5 (a disintegrin and metalloproteinase with thrombospondin motifs 5; Adamts5^ΔCat^ mice), accumulation of intact versican is observed in cardiac tissue after angiotensin II infusion for 2 or 4 weeks. Transforming growth factor β1 (TGFβ1) levels were similar to Adamts5^+/+^ controls. **B**, Gene expression level of Vcan V1 and Adamts5 in the left ventricle of Adamts5^+/+^ and Adamts5^Δcat^ mice at baseline (n=5 and n=9, respectively) and after angiotensin II treatment for 2 weeks (n=6 in both cases) or 4 weeks (n=7 and n=9, respectively). *P* values were calculated using a 2-way ANOVA with Bonferroni corrections. **C**, Versikine, the main fragment produced on ADAMTS-mediated versican cleavage, was undetectable in hearts of Adamts5^ΔCat^ mice at baseline and after angiotensin II infusion for 2 or 4 weeks. **D**, Picrosirius Red staining for collagen. A similar increase in collagen content was observed in Adamts5^+/+^ and Adamts5^ΔCat^ mice after angiotensin II for 2 weeks.

To demonstrate the contribution of ADAMTS5 to cardiac versican degradation, mice with genetic deletion of the catalytic site of ADAMTS5 (Adamts5^ΔCat^) were subjected to angiotensin II infusion for 2 and 4 weeks.^17^ Contrary to models of acute cardiac ischemia, this model induces a more global, homogeneous, and reproducible ECM remodeling, which allows for direct assessment of cardiac function. Versican levels were markedly increased in Adamts5^ΔCat^ mice compared with Adamts5^+/+^ controls, particularly at 2 but also at 4 weeks of angiotensin II infusion (Figure 3A and Figure S2B). Whereas the angiotensin II–induced increase in transforming growth factor β-1 (TGFβ1) was similar to Adamts5^+/+^ mice (Figure 3A), the induction of versican expression was less pronounced in Adamts5^ΔCat^ mice (Figure 3B, left). Angiotensin II infusion led to a reduction in *Adamts5* gene expression in Adamts5^+/+^ mice (Figure 3B, right). To test whether reduced proteolytic turnover may contribute to the accumulation of versican in Adamts5^ΔCat^ mice, we probed for the ADAMTS-specific cleavage product, versikine. Versikine was undetectable in Adamts5^ΔCat^ mice even after angiotensin II infusion (Figure 3C). In contrast, collagenous fibrosis was similar to Adamts5^+/+^ mice as indicated by Picrosirius Red staining (Figure 3D and Figure S3). No significant changes in inflammatory cell infiltration or expression of inflammatory markers (CD68 and galectin-3/LGALS3) were detected between genotypes after angiotensin II infusion (Figure S4A and S4B). Edema was present on angiotensin II infusion in subepicardial areas of both Adamts5^+/+^ and Adamts5^ΔCat^ mice (Figure S4C).

### Cardiac Function in Adamts5^ΔCat^ Mice

**Figure 4.**
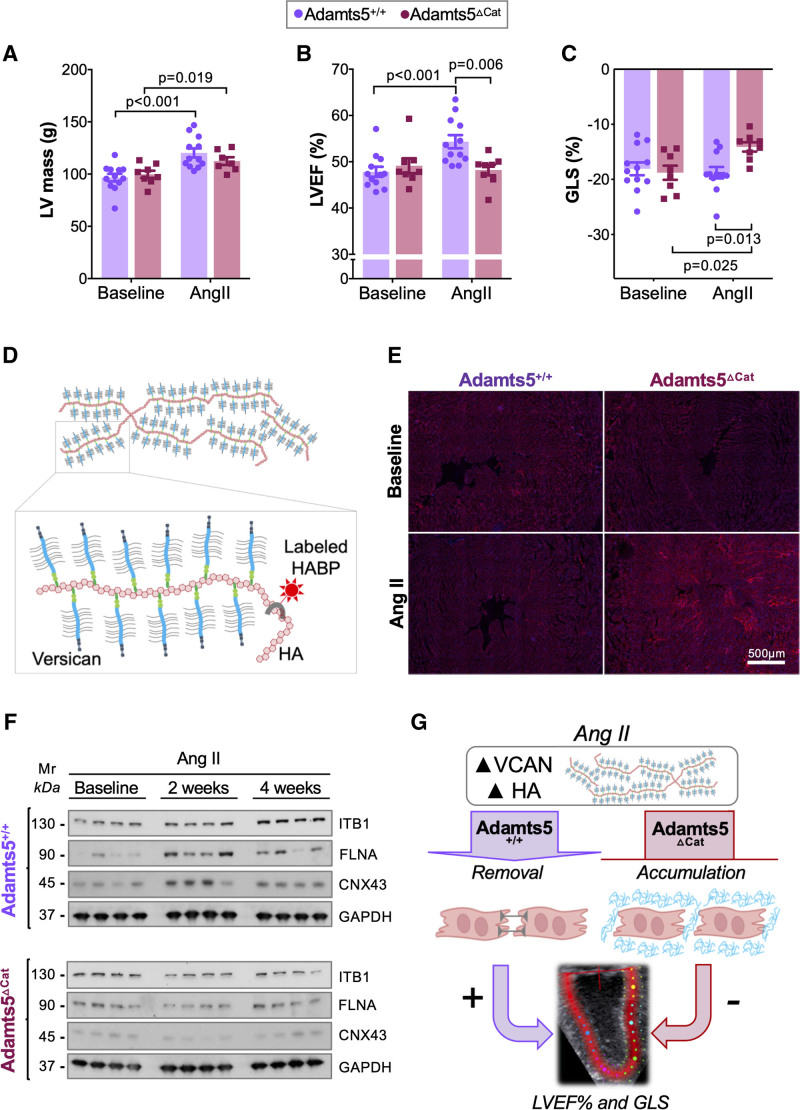
**Effect of versican accumulation on cardiac function. A**, Angiotensin II infusion induced a similar increase of left ventricular (LV) mass in both Adamts5^+/+^ and Adamts5^ΔCat^ mice (n=12 and n=8, respectively). **B**, No differences in LV ejection fraction % (LVEF%) were observed at baseline. After 2 weeks of angiotensin II treatment, however, a reduced LVEF% was observed in Adamts5^ΔCat^ compared with Adamts5^+/+^ mice. **C**, Speckle tracking echocardiography revealed impaired global longitudinal strain (GLS) in Adamts5^ΔCat^ mice in response to angiotensin II infusion. **D**, Versican binds and coats hyaluronic acid (HA). Biotinylated HABP (HA-binding protein) can be used to assess HA abundance in tissues. **E**, HA accumulation in Adamts5^ΔCat^ mice. HA was stained in cardiac tissue sections using HABP. **F**, Immunoblotting for ITB1 (integrin β1), FLNA (filamin A), and CNX43 (connexin 43). Levels increased after angiotensin II infusion in Adamts5^+/+^ mice but not in Adamts5^ΔCat^ mice. **G**, Pericellular accumulation of versican and HA was associated with impaired LV function and a decrease in proteins implicated in cell–cell communication. *P* values were calculated using paired 2-way ANOVAs with Bonferroni corrections.

To evaluate the functional consequences of CSPG accumulation in the heart, echocardiographic assessments were performed in Adamts5^ΔCat^ and Adamts5^+/+^ mice. Representative echocardiographic images are shown in Figure S5A and S5B. LV mass as estimated by echocardiography was higher after angiotensin II infusion, but did not differ significantly between genotypes (Figure 4A). This was in agreement with the extent of cardiac hypertrophy on tissue harvesting (Figure S5C and S5D). Standard 2D ultrasound echocardiography revealed no baseline differences in LV ejection fraction % (LVEF%). On angiotensin II infusion, however, the increase in LVEF% was attenuated in Adamts5^ΔCat^ mice (Figure 4B). Similarly, global longitudinal strain (GLS) as measured by high-frequency speckle tracking echocardiography (STE)^19^ was impaired in Adamts5^ΔCat^ mice on angiotensin II treatment (Figure 4C). Diastolic function as assessed by reverse-longitudinal strain rate and left atrial size in echocardiography was not different.^19,20^ A summary of echocardiographic measurements is provided in Table S2.

### Pericellular Accumulation of ECM

Lack of ADAMTS5 activity predominantly resulted in cardiac versican accumulation on angiotensin II infusion (Figure S6A and S6B and Table S3). Versican binds to HA through its HA-binding domain and is an important regulator of HA remodeling in the heart (Figure 4D). Gene expression levels of HA-degrading enzymes (hyaluronidases, HYAL1, and HYAL2) and HA synthases (HYAS1, HYAS2, and HYAS3) were similar in Adamts5^ΔCat^ and Adamts5^+/+^ mice (Figure S6C). Adamts5^ΔCat^ mice, however, displayed a higher HA content on angiotensin II infusion (Figure 4E and Figure S6D). Because pericellular versican and HA accumulation may affect cell–cell communication,^21^ we determined levels of proteins involved in cardiac cell adhesion and communication. In response to angiotensin II infusion, ITB1 (integrin β1), FLNA (filamin A), and CNX43 (connexin 43) were increased in hearts of Adamts5^+/+^ mice. In Adamts5^ΔCat^ mice, however, this response was blunted even after 4 weeks of angiotensin II infusion (Figure 4F). Thus, loss of ADAMTS5 activity reduces the abundance of cardiac proteins implicated in cell–cell communication (Figure 4G).

### Versican and ADAMTS Expression in Human Hearts

**Figure 5.**
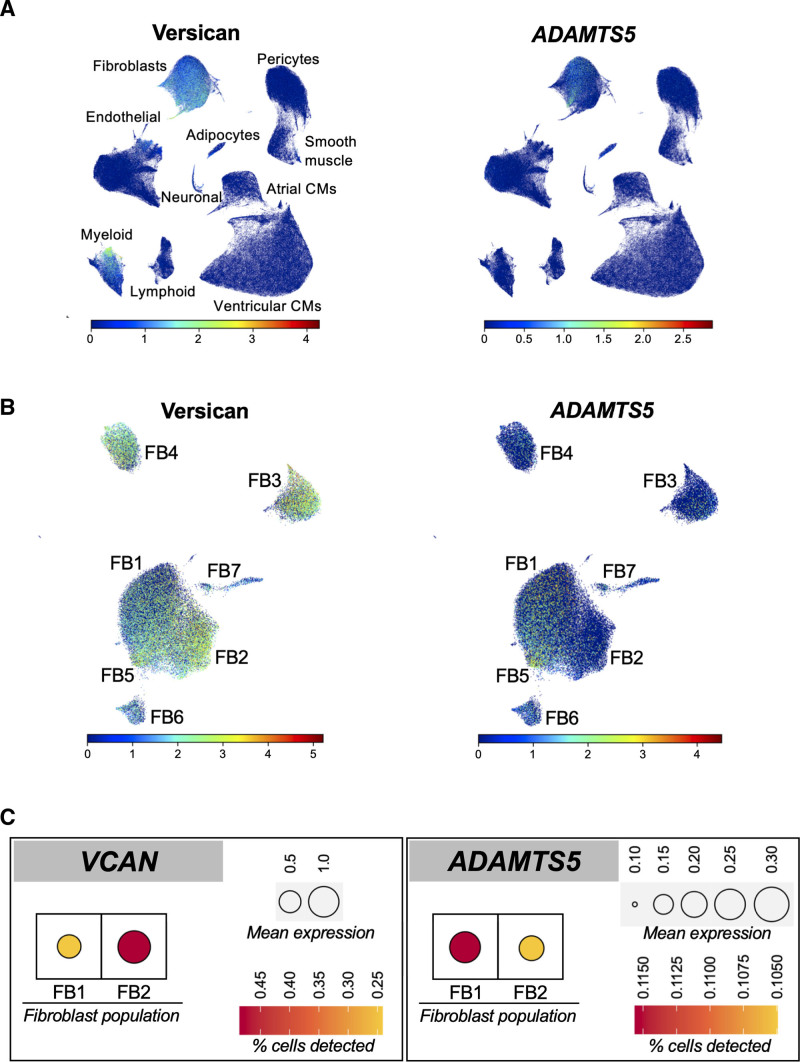
**Expression of ADAMTS5 and versican in human hearts. A**, Single-cell RNA sequencing data in human cardiac tissue. Versican expression was restricted to cardiac fibroblasts (CFs) and a subset of myeloid cells. ADAMTS5 (a disintegrin and metalloproteinase with thrombospondin motifs 5) was expressed by CFs (FB). **B**, FB1 showed high ADAMTS5 but low versican expression. The opposite was the case for FB2. Thus, there was an inverse association between the expression of ADAMTS5 and of its main substrate versican in CFs of human hearts. **C**, Aggregated expression for ADAMTS5 and VCAN in the cardiac fibroblast populations FB1 and FB2, displaying both levels of expression and the percentage of cells in which each transcript expression was detected. CM indicates cardiomyocyte.

In a single-cell RNA sequencing (scRNAseq) dataset from different human cardiac regions,^22^ expression of versican was mainly restricted to CFs. Consistent with our previous proteomics results in mouse CFs,^6^ ADAMTS5 was the main ADAMTS expressed in CFs (Figure 5A). In the heart, ADAMTS1 was expressed in endothelial cells, pericytes and smooth muscle cells, and ADAMTS4 was abundant only in pericytes and smooth muscle cells (Figure S7A). Among the different CF populations (Figure 5B), including ECM-producing and ECM-remodeling CFs, all expressed versican in abundance. ADAMTS5 expression, however, was mainly restricted to a specific subgroup of CFs. This population (FB1) was the main contributor to the ventricular CF pool and was distinct from the predominant CF population in atria (FB2; Figure S7B and S7C). Ventricular CFs had comparatively higher expression levels of ADAMTS5 and lower expression of versican (Figure 5C and Figure S8A and S8B). A proteomics comparison of human LV and left atrial tissues from unused donor hearts (n=7 each, obtained from A Coruña University Hospital) showed lower levels of versican in LV (Figure S8C).

### Effects of Medication on the ECM Composition in Ischemic HF

**Figure 6.**
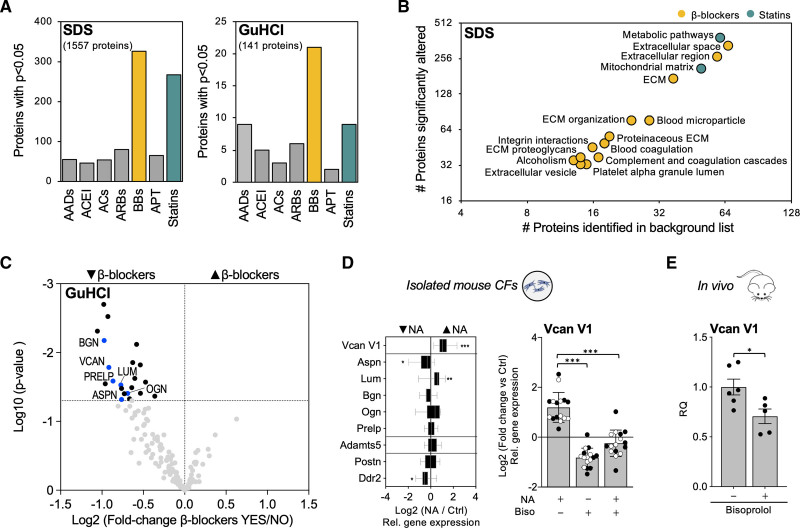
**Extracellular matrix remodeling in ischemic heart failure. A**, Compared with other medications, the use of β-blockers (44 of 65 patients) was associated with the highest number of significant changes in SDS and the extracellular matrix (ECM)–rich GuHCl extracts. **B**, Term enrichment analyses on proteins changing significantly in patients with ischemic heart failure with the use of different medications. DAVID functional annotation tool was used for the analysis. The horizontal axis represents the number of proteins quantifiable proteins in our proteomics analysis for a specific Gene Ontology/Reactome/Kyoto Encyclopedia of Genes and Genomes term. The vertical axis represents the number of proteins significantly altered in that term. Only terms accounting for <20% of the background list and >10 proteins are displayed. A list of all proteins per term is provided in Table S8. EASE score threshold was set to 0.1, minimum number of proteins for each corresponding term was set to 2, and *P* values were corrected for multiple testing using the Benjamini-Hochberg method. *P*<0.05 was considered significant. **C**, Volcano plot showing a reduction of ECM and ECM-associated proteins in the proteomics analysis of GuHCl extracts in patients with ischemic HF with β-blockers. Proteoglycans (labeled) account for the majority of changes. **D**, CFs isolated from wild-type mice (n=8 male and n=12 female) were stimulated with noradrenaline (NA). Compared with untreated controls, Versican V1 (Vcan V1) expression was increased after incubation with NA. *P* values were derived from 2-tailed paired *t* tests with Bonferroni corrections for multiple comparisons. *P<0.05; **P<0.01; ***P<0.001. The effect of β-blockers on CFs stimulated with NA was assessed using bisoprolol (biso). Bisoprolol prevented the NA-induced increase in Vcan V1 expression in CFs. CFs were obtained from male and female mice (n=8 each, black and white dots, respectively). *P* values were derived from a 2-tailed ANOVA with Bonferroni correction for multiple comparisons. ***P<0.001. **E**, Compared with controls (n=6), administration of bisoprolol in vivo for 2 weeks (n=5) reduced cardiac expression of Vcan V1. *P* value was derived from a 2-tailed paired *t* test. *P<0.05. AC indicates anticoagulant; ACEI, angiotensin-converting enzyme inhibitor; APT, antiplatelet therapy; ASPN/Aspn, asporin; BB, β-blocker; BGN/Bgn, biglycan; Ddr2, discoidin domain receptor 2; LUM/Lum, lumican; OGN/Ogn, mimecan; Postn, periostin; and PRELP/Prelp, prolargin.

Next, LV tissues from a larger cohort of patients with ischemic HF (n=65, obtained from the Transplantation Biobank of the Heart and Vascular Center at Semmelweis University) were subject to proteomics analysis. Of 177 ECM and ECM-associated proteins in the ECM-enriched fractions (4 M GuHCl extracts; Table S4), 141 (79.7%) were suitable for quantification. The 0.1% SDS extracts containing predominantly intracellular proteins were analyzed by multiplexed proteomics (Table S5). Age- and sex-corrected comparisons based on comorbidities and long-term medications are shown in Tables S6 and S7. Use of β-blockers returned the highest number of altered proteins in both extracts (Figure 6A). Enrichment analysis using Gene Ontology annotation, Reactome, and Kyoto Encyclopedia of Genes and Genomes pathway databases (0.1% SDS extracts, Table S8), returned terms related to ECM synthesis and processing for use of β-blockers (Figure 6B). In contrast, few changes were observed with statins, in particular in the ECM-enriched fraction (4 M GuHCl extracts; Figure 6A). Instead, statin use was associated with terms related to metabolism (Figure 6B). Among ECM proteins, patients with ischemic HF on β-blockers showed predominantly a reduced abundance of proteoglycans. Both SLRPs as well as VCAN and its fragmentation product, versikine, were affected (Figure 6C, Table S6, and Figure S9A). CD44, the main receptor for HA, was also reduced with β-blocker usage (see Table S7). Heart rate showed a positive correlation with cardiac ECM protein abundance (see Table S9), with versican protein abundance displaying the strongest correlation (Figure S9B). In contrast, there were no significant changes in mRNA expression levels (Figure S9C).

### Noradrenalin Induces Versican Expression in CFs

To explore potential direct effects of β-adrenergic signaling on CFs, we treated mouse ventricular CFs with noradrenaline. TGFβ1 treatment was used for comparison (Figure S10A). The reduction in Adamts5 expression in TGFβ1-treated CFs was consistent with our observations in angiotensin II–treated mice, where an increase in pre-TGFβ1 accompanied versican accumulation (see Figure 3A and 3B). Treatment with noradrenaline induced versican expression. Unlike TGFβ1, noradrenaline did not affect Adamts5 (Figure 6D, left). Treatment of murine CFs with the β1-selective adrenoreceptor antagonist bisoprolol blocked the noradrenaline-induced upregulation of versican expression in CFs from both female and male mice, demonstrating a direct effect of β-blockers in regulating this key proteoglycan (Figure 6D, right). Again, Adamts5 expression was not affected (Figure S10B). Similarly, administration of bisoprolol for 2 weeks reduced versican expression in murine hearts (Figure 6E), with no effect on gene expression levels of Adamts5 (Figure S10C).

### Clustering Analysis of Ischemic HF

**Figure 7.**
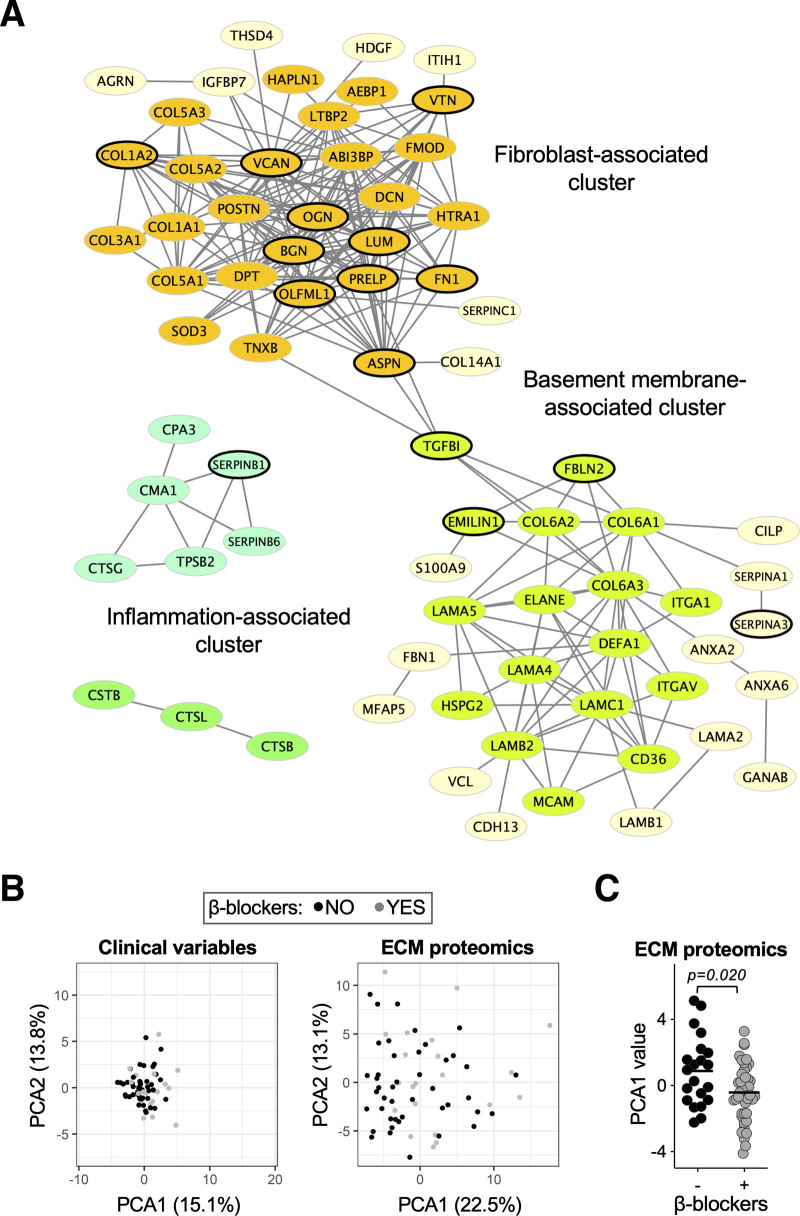
**Effect of β-blockers on cardiac extracellular matrix remodeling. A**, Abundance pattern-based clustering of extracellular matrix (ECM) proteins. A dense cluster of interstitial, fibroblast-secreted ECM proteins comprised the majority of those altered with β-blocker usage (circled in black). Serum-derived proteins were excluded from this analysis. **B**, Principal component analyses (PCAs) based on clinical variables or ECM proteomics. Patients with or without β-blocker administration shared similar clinical characteristics. Their ECM proteomic profiles, however, were distinct. **C**, A comparison of PCA values for ECM proteomics profiles between patients with or without β-blocker administration detected significant differences according to PCA1. The limma package was used for *P* value calculation using the Ebayes algorithm and correcting for age and sex.

Clustering analyses based on protein-to-protein correlation of abundance returned a dense cluster of fibroblast-secreted interstitial ECM proteins. Versican was central to this cluster, as were the majority of ECM proteins showing changes in patients on β-blockers. A second cluster comprised ECM proteins associated with the basement membrane of nonfibroblasts (Figure 7A). In a principal component analysis, clinical variables did not separate patients on β-blockers from those without. However, when ECM proteomic profiles were used, a good separation was obtained (Figure 7B and Figure S11A). The first principal component (PCA1), accounting for 22.5% of the variability, determined the best separation between patients with and without β-blockers (Figure 7C and Figure S11B) and was mainly dependent on proteoglycan abundance (Figure S11C). No significant separation was obtained for other medications (data not shown). Thus, β-blockers reduce cardiac ECM deposition, independent of other clinical variables, with proteoglycans being most affected.

## Discussion

This study generated several novel findings. First, using proteomics, we obtained an unprecedented depth of coverage for the human cardiac ECM. We confirmed our previous findings in a porcine model of I/R injury^9^ by demonstrating that versican and other proteoglycans also accumulate in the scarred myocardium of patients with ischemic HF. Second, we confirmed that ADAMTS proteases cleave versican during cardiac remodeling. Third, we demonstrated that versican accumulation in Adamts5^ΔCat^ mice was associated with impaired cardiac function and a reduction in proteins involved in cell–cell communication. Last, we explored the effect of medications on ECM remodeling and revealed that β-blockers attenuate versican accumulation in ischemic HF.

### CSPGs in the Cardiac ECM

CSPGs are critical components of the ECM.^2,10^ Aggrecan is the major proteoglycan of cartilage tissue. Brevican and neurocan are essential components of the ECM in nervous tissues.^23^ Versican is the predominant CSPG in the heart, with CFs being the main source.^24,25^ Whereas genetic deletion of other CSPGs results in viable mice until birth,^26–28^ mice deficient in versican die embryonically and display major cardiovascular defects. Even partial deletion of the N-terminal G1 domain of versican is detrimental to the heart.^29^ Notwithstanding the importance of collagens in cardiac remodeling,^9,15,30^ the contribution of CSPGs may be underestimated given the challenges in detection of these highly glycanated proteins using antibody-based methods.^31^ Proteomics studies analyzing human cardiac ECM tend to be performed without deglycosylation, resulting in incomplete coverage and possibly inaccurate quantification by mass spectrometry.^32^ Thus, we used stringent buffer conditions that allow for efficient extraction of CSPGs, followed by deglycosylation of the ECM extracts before proteomics analysis.^9,16^

### Role of ADAMTS5 in the Heart

A constant turnover of versican is required to avoid accumulation as shown in different tissues.^17,33,34^ The VCAN/ADAMTS5 axis emerged as an important determinant for cardiac ECM composition. On the basis of the scRNAseq data from 2 cardiac regions of human hearts, the CF subsets enriched in ADAMTS5 are the least enriched for VCAN.^22^ Thus, there is an inverse correlation between the gene expression of the protease and its main substrate. In mice, ADAMTS-mediated cleavage of versican is dependent on ADAMTS5. In wild-type mice, angiotensin II infusion reduced cardiac ADAMTS5 expression. In Adamts5^ΔCat^ mice, loss of ADAMTS5 activity was sufficient to exacerbate the cardiac buildup of versican and HA. The former observation was supported by our finding that versikine, an ADAMTS-specific versican fragment, was absent in Adamts5^ΔCat^ mice. Versican binds to HA through the G1 domain contained within versikine.^35^ Without cleavage by ADAMTS5, versican–HA complexes cannot be readily accessed by other versican-degrading enzymes, leading to accumulation of versican–HA aggregates.^36^

### Effect of ADAMTS5 on Cardiac Function

Adamts5^ΔCat^ mice have normal baseline cardiac function, in line with a previous report.^37^ In response to angiotensin II treatment, their increase in LV mass was similar to littermate controls. The accumulation of versican–HA aggregates, however, was associated with reduced LVEF and impaired GLS in Adamts5^ΔCat^ mice. Versican contains between 12 and 23 CS chains, depending on the isoform,^35^ and is a major contributor to the CS content in the heart. In a mouse model of cardiac I/R injury, CSPGs in the scar area prevented sympathetic reinnervation, and treatment with chondroitinase was sufficient to overcome this effect.^38^ The accumulation of versican–HA aggregates may also alter cell–cell communication.^21^ First, the angiotensin II–induced upregulation of connexin 43 and integrin β1 was blunted in Adamts5^ΔCat^ mice. Both connexin 43 and integrin β1 are localized at intercalated discs and costameres. β1 integrins have been implicated as a stiffness mechanosensor.^39^ Reductions in connexin 43 have been reported in advanced HF stages.^32^ Second, migration of CFs and inflammatory cells is regulated by CD44-dependent pathways,^14^ the main receptor for HA. HA size is an important factor determining the recruitment of inflammatory cells,^40^ and versikine can induce macrophage polarization.^41^ Third, HA synthesis contributes to postinfarct healing by supporting macrophage survival and by promoting the myofibroblast response during the first days after myocardial infarction.^42^

### Effect of Medications in Ischemic HF

Our proteomics analysis in a larger cohort of patients with ischemic HF (n=65) revealed that versican levels were significantly reduced on treatment with β-blockers. Previous proteomics studies using human cardiac tissue were not designed for the study of ECM proteins^43^ or were too small to assess the effects of medications.^15,30,32,43,44^ Given the negative inotropic and chronotropic effects of β-blockers, the reduction of versican could be an indirect effect. In a rat model of chronic ischemic HF, ivabradine resulted in a marked attenuation of cardiac fibrosis.^45^ Ivabradine is a cardiotonic agent and reduces the heart rate through mechanisms that are distinct from β-blockers. We observed a positive correlation between heart rate and versican protein abundance. Thus, the effect of β-blockers on the cardiac ECM could be attributed to their negative inotropic and chronotropic activity. On the other hand, β-adrenergic stimulation of CFs may have direct effects. β1-adrenoceptor stimulation has been shown to induce synthesis of collagen 1 and fibronectin in CFs isolated from neonatal rats.^46,47^ We now demonstrate that noradrenaline stimulation induces versican expression in adult murine CFs through β1 adrenoreceptor-dependent mechanisms. Thus, in addition to affecting cardiomyocytes, β-blockers may directly affect CFs.

### Limitations

Whereas ADAMTS5 has a high specificity for CSPGs,^18^ other targets could be affected by loss of ADAMTS5 activity. Approximately 12% of Adamts5^ΔCat^ mice have bicuspid aortic valves.^48^ Thus, all mice with valve defects were excluded from our experiments. We have previously reported an attenuated rise in blood pressure in Adamts5^ΔCat^ mice on angiotensin II infusion.^17^ Whereas blood pressure is an important determinant of cardiac remodeling and cardiac function, reduced blood pressure would only reduce any prohypertrophic effects, supporting a direct cardiac effect of ADAMTS5 deficiency. A reduced blood pressure in Adamts5^ΔCat^ compared with Adamts5^+/+^ mice was also observed by Hemmeryckx et al,^37^ with no differences in associated cardiac phenotypes on feeding a high-fat diet. We used HF tissue from end-stage explanted failing hearts, which might be different from less severe human HF. However, myocardial samples from the latter group are difficult to procure.

### Conclusions

Besides collagen deposition, CSPG deposition can be detrimental to cardiac function. We provide evidence that ADAMTS proteases, in particular ADAMTS5, control versican turnover. Buildup of versican in Adamts5^ΔCat^ mice on angiotensin II infusion resulted in HA accumulation, which was accompanied by compromised cardiac function and a decrease in proteins implicated in cell–cell communication. Use of β-blockers was associated with reduced CSPG deposition in patients with ischemic HF. The effects of β-blockers on nonmyocytes and the composition of the cardiac ECM deserve further investigation in HF.

## Article Information

### Sources of Funding

Dr Barallobre-Barreiro is a British Heart Foundation Intermediate Fellow (FS/19/33/34328). Drs Mayr and Shah are British Heart Foundation Chair Holders (CH/16/3/32406 and CH/1999001/11735, respectively) and received support from the British Heart Foundation Center for Vascular Regeneration With Edinburgh/Bristol (RM/17/3/33381). Dr Doménech’s work was supported by Project PI16/02049 integrated in the National Plan for Scientific Research, Development and Technological Innovation, 2013–2016, and funded by the ISCIII–General Subdirection of Assessment and Promotion of Research–European Regional Development Fund. Dr Merkely’s work was funded by the National Research, Development and Innovation Fund (NVKP_16-1–2016-0017) and the Thematic Excellence Program of the Ministry for Innovation and Technology (2020-4.1.1.-TKP2020), Hungary. Dr Radovits is supported by the National Research, Development and Innovation Office of Hungary (K134939).

### Disclosures

None.

### Supplemental Material

Methods

Figures S1–S11

Tables S1–S10

References 49 and 50

## Supplementary Material


