# Supplementary material for: Extracellular Matrix in Heart Failure: Role of ADAMTS5 in Proteoglycan Remodeling
Source: Circulation. 2021 Nov 22;144(25):2021–34. doi: 10.1161/CIRCULATIONAHA.121.055732 (PMC8687617; doi:10.1161/CIRCULATIONAHA.121.055732)
Supplement: Supplementary file 1 [file cir-144-2021-s001.pdf]

SUPPLEMENTAL MATERIAL

THE EXTRACELLULAR MATRIX IN HEART FAILURE: THE ROLE OF ADAMTS5 IN PROTEOGLYCAN REMODELLING

Javier Barallobre-Barreiro, Tamás Radovits, Marika Fava, Ursula Mayr, Wen-Yu Lin, Elizaveta Ermolaeva, Diego Martínez-López, Eric L. Lindberg, Elisa Duregotti, László Daróczi, Maria Hasman, Lukas E. Schmidt, Bhawana Singh, Ruifang Lu, Ferheen Baig, Aleksandra Malgorzata Siedlar, Friederike Cuello, Norman Catibog, Konstantinos Theofilatos, Ajay M. Shah, Maria G. Crespo-Leiro, Nieves Doménech, Norbert Hübner, Béla Merkely, Manuel Mayr.

SUPPLEMENTAL MATERIALS AND METHODS

Further details regarding general reagents, commercial kits, enzymes, oligonucleotide primers, antibodies and mouse strains are provided in the Supplemental Table X (**Major Resources Table**).

Human samples. Well-characterised myocardial tissues from end-stage heart failure (HF) patients were obtained after cardiac transplantation from a collection of A Coruña University Hospital's Advanced Heart Failure and Heart Transplantation Unit (Registration Number C.0000419, A Coruña, Spain), and the Transplantation Biobank of the Heart and Vascular Center at Semmelweis University (Budapest, Hungary). Control tissues were obtained from unused donor hearts at A Coruña Biobank (Registration Number B.0000796, A Coruña, Spain). All samples from end-stage HF patients were biopsied from the anterior wall of the left ventricle (LV) of explanted hearts during heart transplantation. All myocardial samples were immediately frozen in liquid nitrogen, and stored at −80°C. All samples were pseudonymised. Written consent was obtained from all patients enrolled in the study and institutional REC approval was obtained for the use of human tissues at all Institutions involved in the study: King's College London, REC LRS-17/18-5080, REC decision Entry-17440; A Coruña University, REC Entry 2015/312; Semmelweis University, ETT TUKEB 7891/2012/EKU (119/PI/12.) and ETT TUKEB IV/10161-1/2020.

Animal Experiments. All animal procedures were performed by authorized researchers. Housing and animal care was in accordance with the UK Animals (Scientific Procedures) Act

1986 and institutional ethical approval. Male Adamts5$^{\triangle Cat}$ mice (JAX stock no. 005771, B6.129P2-*Adamts5*^{tm1Dgen}/J) and Adamts5$^{+/+}$ wildtype littermates on a C57Bl/6J background (i.e. back-crossed 8 times) were used at 10 weeks of age for Ang II infusion. Genotyping of Adamts5$^{\Delta cat}$ and Adamts5$^{+/+}$ mice was performed according to the specifications of the provider. Murine cardiac fibroblasts (CFs) were isolated from 4-7 weeks old C57Bl/6J mice.

3-step protein extraction and deglycosylation. ECM protein enrichment was performed using our previously published 3-step extraction method, involving sequential incubation with 0.5 mol/L NaCl for 1 hour, 0.1% sodium dodecyl sulfate (SDS) for 16 hours, and a final incubation for 72 hours with 4 mol/L guanidine hydrochloride (GuHCl). GuHCl extracts were enzymatically deglycosylated. For samples undergoing a subsequent in-gel liquid chromatography and mass spectrometry (LC-MS) analysis, removal of glycosaminoglycan (GAG) side chains (deglycanation) of the extracts was achieved with chondroitinase, heparanase and ketaranase in a 150 mM NaCl, 50 mM sodium acetate pH 6.8 buffer supplemented with proteinase/phosphatase inhibitors and 10 mM EDTA for 16 hours at 37 °C.[16] For samples undergoing in-solution LC-MS analysis, a two-step deglycosylation protocol was pursued to ensure pan-deglycosylation. Unlike PNGase-F, the majority of deglycosylation enzymes act on the sugar residues. Resuspended samples were first incubated with a combination of deglycanation enzymes for GAG-removal as described above, plus an additional 4 enzymes: Endo-α-N-acetylgalactosaminidase (O-glycosidase from Streptococcus pneumoniae) cleaves serine- or threonine-linked unsubstituted Galβ1,3GalNAca; The debranching enzymes remove terminal residues: α2-3,6,8,9-Neuraminidase (from Arthrobacter ureafaciens) cleaves all non-reducing terminal branched and unbranched sialic acids; β1,4-Galactosidase (from *Streptococcus pneumoniae*) releases only β1,4- linked, non-reducing terminal galactose from complex carbohydrates and glycoproteins; β-N-Acetylglucosaminidase (from *Streptococcus pneumoniae*) cleaves all non-reducing terminal β-linked N-acetylglucosamine residues from complex carbohydrates and glycoproteins. Removal of sugar monomers facilitates the later cleavage by PNGase-F. After 24-hour treatment, water was evaporated from the extracts and

substituted for an equal amount of O^{18}-labeled water containing PNGase-F. This enzyme cleaves all asparagine-linked complex, hybrid, or high mannose oligosaccharides unless α1,3-core fucosylated. Importantly, asparagine must be peptide-bonded at both termini. This second step was carried out, under constant agitation, at 37 °C for 24 hours.[16]

In-gel LC-MS protein separation and digestion. Deglycanated GuHCl extracts were denatured and reduced in sample buffer containing 100 mM Tris, pH 6.8, 40% glycerol, 0.2% SDS, 2% beta-mercaptoethanol and 0.02% bromophenol blue and boiled at 96 °C for 10 min. 20 µg of protein per sample was loaded and separated on Bis-Tris discontinuous 4-12% polyacrylamide gradient gels (NuPage, Life Technologies). Pre-stained protein standards were run alongside the samples to allow molecular mass estimation of proteins (Life Technologies). Ten gel bands were excised across the entire lanes, leaving no empty gel pieces behind. Subsequently, all gel bands were subjected to in-gel digestion with trypsin using an Investigator ProGest (Digilab) robotic digestion system according to a previously described protocol.[9] Tryptic peptides were lyophilized, reconstituted with 0.05% formic acid.

In-gel LC-MS, database search and data filtering. Peptides were separated on a nanoflow LC system (Ultimate 3000 RSLCnano, Thermofisher Scientific). 15 µl of peptide solution were injected onto a trap column (Thermo Scientific, Acclaim PepMap 100 C18, inner diameter 0.1 mm × length 20 mm, particle size 5 µm, pore size 100Å) at a flow rate of 8 µl/min for 5 min, using 0.1% formic acid (FA, aq). The following nano LC gradient was then used to separate the peptides at 0.3 µl/min: 0–5 min, 3% B; 5–10 min, 3–10% B; 10−40 min, 10−40% B; 40–45 min, 40–80% B; 45–49 min, 80% B; 49–50 min, 80–3% B; 50–65 min, 3% B; where A = 0.1% FA (aq), and B = 0.1% FA, 80% acetonitrile (aq). The nano column (Thermo Scientific, Acclaim PepMap 100 C18, 0.075 mm × 500 mm, 3 µm, 100 Å) was kept at 40°C. The LC eluate was analysed using a Q Exactive mass spectrometer (Thermo Scientific) operating in data-dependent TopN mode (loop count 10). Survey full scan spectra were acquired over the mass-to-charge (m/z) range 400–2000 using Orbitrap detection (resolution 70,000). Data-dependent MS^2 scans were performed in the Orbitrap (resolution 17,500) following higher-energy C-trap dissociation. Proteome Discoverer software (Thermo Scientific, version 2.4.1.15) was used to search raw data files against a human database (UniProtKB/Swiss-Prot version from January

2020, 20,365 protein entries) using Mascot (Matrix Science, version 2.6.0). Mass tolerance was set at 10 ppm for precursor ions and 20 mmu for fragment ions. Trypsin was used as the protein-digesting enzyme with up to two missed cleavages being allowed. Carbamidomethylation of cysteine was chosen as a static modification. Oxidations of lysine, methionine and proline were chosen as dynamic modifications. Spectral counts were calculated using Scaffold (Proteome Software, version 4.11.1). Peptide identification threshold was set to 95% and protein identification threshold to 99% with a minimum number of two peptides per protein.

In-solution protein digestion. 20 µg of SDS protein extract or 20 µg of GuHCl protein extract were denatured using urea/thiourea (final concentration 6 M urea, 2 M thiourea) and reduced using dithiothreitol (final concentration 10 mM) at 37 °C and 180 rpm for 1 hour. Reduced proteins were cooled down to room temperature before being alkylated in the dark for 1 hour using iodoacetamide (final concentration 50 mM). Pre-chilled (-20 °C) acetone was used in ten-fold excess to precipitate the alkylated proteins overnight at -20 °C. Proteins were then centrifuged at 16,000 g for 45 min at 4 °C and the acetone supernatant was discarded. Protein pellets were dried using a vacuum centrifuge (Thermo Scientific, Savant SPD131DDA) and resuspended in 0.1 M triethylammonium bicarbonate, pH 8.2. 0.4 µg of trypsin protease (Thermo Scientific, 90057) were used to digest the samples at 37 °C and 180 rpm for 19 hours. The digestion was stopped by acidification of the samples with 10% TFA (final concentration 1% TFA). Peptide samples were purified using a 96-well C18 spin plate (Harvard Apparatus). The resin was activated using 200 µl methanol and centrifuged at 1,000 g for 1 min. Wash steps included 200 µl of 80% ACN, 0.1% TFA in H_2O, and three equilibration steps using 200 µl of 1% ACN, 0.1% TFA in H_2O with centrifugation (1,000 g for 1min) after each step. Samples were loaded onto the resin and centrifuged at 2,000 g for 1 min; the flow-through was reloaded onto the resin a second time and centrifugation repeated. The resin was then washed three times with 200 µl 1% ACN, 0.1% TFA in H_2O (centrifugation at 1,500 g for 1 min). Finally, the samples were eluted with 170 µl of 50% acetonitrile, 0.1% TFA in H_2O (centrifugation at 1,500 g for 1 min); this step was repeated, combining the collected eluate. The eluates were dried using a speed vac (Thermo Scientific, Savant SPD131DDA) and re-suspended in 0.05% TFA in 2% ACN for LC-MS/MS analysis.

Tandem Mass Tag (TMT) labelling for SDS protein extracts and high pH reversed-phase peptide fractionation. 10 µg of peptide were labelled using a TMT10plex Isobaric Mass Tag Labelling Reagent Set (Thermo Scientific, 90406) according to the manufacturer's instructions. A pooled sample consisting of aliquots of peptides from all individual samples was used as a cross-10plex control and labelled with TMT Label Reagent TMT10-131. After combining the labelled peptides into 10plex mixes, 50 µg of every 10plex mix were dried down in a vacuum centrifuge. The dried 10plexes were resuspended in 300 µl of 0.1% trifluoroacetic acid (TFA, aq) and fractionated using a High pH Reversed-Phase Peptide Fractionation Kit (Thermo Scientific, 84868) according to the manufacturer's instructions for TMT-labelled peptides. Eight fractions per 10plex were vacuum centrifuged to dryness and reconstituted in 26 µl of 2% acetonitrile (ACN), 0.05 % TFA (aq). The eight fractions were combined into three mixes according to a previously established protocol (data not shown; 16 µl per fraction; mix 1 = fractions 1, 4 and 8; mix 2 = fractions 2 and 6; mix 3 = fractions 3, 5 and 7).

In-solution liquid chromatography-mass spectrometry (LC-MS). TMT-labelled, fractionated peptides were analysed using an LC-MS assembly consisting of a nano-flow UltiMate 3000 high-performance liquid chromatography (HPLC) system (Thermo Scientific) coupled via an EASY-Spray NG Source (Thermo Scientific) to an Orbitrap Fusion Lumos Tribrid mass spectrometer (Thermo Scientific). Peptides were trapped on a C18 cartridge (Thermo Scientific, 160454) at a flow rate of 25 µl/min for 3 min using 0.1% formic acid (FA, aq). Peptides were eluted from the trap cartridge and separated using an EASY-Spray C18 column (Thermo Scientific, ES803) at 45 °C and a flow rate of 0.25 µl/min using the following gradient: 0–3 min, 4% B; 3–10 min, 4–8% B; 10–200 min, 8–30% B; 200–210 min, 30–40% B; 210–215 min, 40–99% B; 215–220 min, 99% B; 220–250 min, 4% B; with A being 0.1% FA (aq), and B being 80% ACN, 0.1% FA (aq). MS data was acquired using a Synchronous Precursor Selection (SPS)-MS3 method with a cycle time of 3 s. An Orbitrap full MS scan (scan range 375–1500 m/z; resolution 120,000; automatic gain control target 4e5; max. injection time 50 ms) was followed by an ion trap data-dependent MS2 scan using collision-induced dissociation (CID) fragmentation (dynamic exclusion duration 60 s, MS isolation window 0.7 m/z; CID collision energy 35%; automatic gain control target 1e4; max. injection time 50 ms), and an Orbitrap data-dependent SPS-MS3 scan

using higher-energy C-trap dissociation (HCD) fragmentation to generate TMT reporter ions from 5 SPS precursors (scan range 100-500 m/z; resolution 60,000; MS2 isolation window 2 m/z; HCD collision energy 65%; automatic gain control target 1e5; max. injection time 105 ms). Peptides originating from human GuHCl extracts were analyzed using nano-flow reversed-phase LC-MS (UltiMate 3000 coupled to Q Exactive HF via EASY-Spray Source, all Thermo Scientific). Flow rate 0.25 µl/min, LC gradient: 0–5 min, 4–10% B; 5–75 min, 10–30% B; 75−80 min, 30−40% B; 80–85 min, 40–99% B; 85–89.8 min, 99% B; 89.8–90 min, 99–4% B; 90–120 min, 4% B; A = 0.1% formic acid in water, B = 80% acetonitrile, 0.1% formic acid in water. Peptides originating from murine GuHCl extracts were analyzed using nano-flow reversed-phase LC-MS (UltiMate 3000 coupled to Q Exactive HF via EASY-Spray Source, all Thermo Scientific). Flow rate 0.30 µl/min, LC gradient: 0–20 min, 4–8 % B; 20–200 min, 8–25 % B; 200–210 min, 25–40 % B; 210–215 min, 40–99 % B; 215–220 min, 99 % B; 220–250 min, 4 % B; A = 0.1% formic acid in water, B = 80% acetonitrile, 0.1% formic acid in water. Q Exactive HF was operating in data-dependent TopN mode (loop count 15). Survey full scan spectra were acquired over m/z range 350–1,600 using Orbitrap detection (resolution 60,000). HCD-type data-dependent MS2 spectra were obtained using Orbitrap detection (resolution 15,000).

Database search of in-solution LC-MS data and data filtering. LC-MS data was processed using Proteome Discoverer (Thermo Scientific, version 2.2.0.388) and Mascot (Matrix Science, version 2.6.0). For the analysis of TMT-labelled peptides (SDS extracts), raw files were searched against a human database (UniProtKB/Swiss-Prot, version from May 2018, 20,349 protein entries). The mass tolerance was set at 10 ppm for precursor ions and 0.8 Da for fragment ions. Trypsin was set as the protein-digesting enzyme with up to two missed cleavages being allowed. Carbamidomethylation of cysteine was set as a static modification, and oxidation of methionine was set as a dynamic modification. To detect TMT-labelled peptides, the modifications specified in the quantification method (lysine and N-terminal residue modification +229.163 Da) were included in the search. Correction for the reagent lot-specific isotopic impurities of TMT tags was applied (TMT10plex lot no. TG271817A). To account for variation in abundances between samples, data was normalised to the total peptide amount. A pooled sample comprising all individual samples had been added to every 10plex mix as a control. After

normalisation, protein abundance was scaled to the average abundance of the pooled sample. Before exporting the data from Proteome Discoverer for further analysis, it was filtered for Master Proteins, a minimum number of two unique peptides per protein and a High Protein FDR Confidence as determined by Proteome Discoverer's Protein FDR Validator node (FDR Confidence Threshold for High Confidence = 0.01). For label-free analyses (GuHCl extracts), Proteome Discoverer (version 2.2.0.388, Thermo Scientific) was used to search raw data files against a human database (UniProtKB/Swiss-Prot version January 2017, 20,171 protein entries) or a mouse database (UniProtKB/Swiss-Prot version January 2021; 17,063 entries) using Mascot (version 2.6.0, Matrix Science). Precursor mass tolerance was 10 ppm, fragment mass tolerance was 20 mmu. Digestion enzyme was trypsin, maximum number of missed cleavages was 2. Cysteine carbamidomethylation (static), oxidation of lysine/methionine/proline (dynamic) and deamidation of asparagine in presence of ^{18}O water (dynamic) were chosen as modifications. Peptide abundances were determined based on precursor ion intensity.

Echocardiography. All imaging studies were performed using the VisualSonicsVevo® 2100 imaging system (Scanhead: RMV707B, 15–45 MHz, cardiac mouse). Anaesthesia was induced for one minute in an induction chamber anesthetized using 5% isoflurane mixed with 1 L/min of 100% oxygen. After placing the mouse in a supine position atop a pad with embedded ECG electrodes, anaesthesia was maintained via inhalation of 1.0–1.5% isoflurane and 98.5-99.0% O_2 at a flow rate of 1 L/min using a nose mask. The ECG signal was monitored throughout the procedure. After immobilising the mouse on the echocardiography stage with tape, chest hair was removed with hair removal cream and a layer of preheated ultrasound gel was applied to the chest. Body temperature was monitored throughout the whole procedure by an inserted rectal probe and maintained within a narrow range (37.0 °C ± 1.5 °C) via the heated platform and a heat lamp. Two-dimensional (B-Mode) images were recorded in parasternal long- and short-axis projections with guided one-dimensional M-mode recordings at the mid-ventricular level, apical of the papillary muscle in both views. Standard measurements of inter-ventricular septum (IVS), left ventricular internal diameter (LVID) and left ventricular posterior wall (LVPW) were performed in systole and diastole in parasternal long-axis projection. Left ventricular (LV) cavity size and wall thickness were measured during at least three beats from this projection

and averaged. Left ventricular anterior wall (LVAW) thickness was determined during at least three beats in systole and diastole in short-axis projection. LV volume [µl] LVvol;d [7.0/(2.4 + LVID;d)] * LVID;d3, LVvol;s [7.0/(2.4 + LVID;s)] * LVID;s3, LV fractional shortening (FS) [%] [(LVID;d - LVID;s)/LVvol;d] * 100 and LV ejection fraction (EF) [%] [(LVvol;d - LVvol;s)/LVvol;d] * 100 were calculated from M-mode measurements. ECG and respiration gating were used to suppress movement artefacts. High-frequency speckle tracking echocardiography (STE) has been previously validated to provide reliable and reproducible assessment of cardiac structure and function in mouse models.[19,20] Semiautomated tracing of the endocardial and epicardial borders were performed in the parasternal long axis view. Subsequent frame by frame tracking through 3 consecutive cardiac cycles allowed automatic calculation of myocardial strain and strain rates. All echocardiographic views were stored digitally in cine loops consisting of 300 frames. Subsequent analysis was performed off-line with the Vevo software (Version 3.2.6) by an experienced cardiologist. By using speckle tracking method, the global longitudinal strain (GLS) was acquired. The LV mass (LVM) was calculated with conventional area-length (AL) formula, a commonly used method based on short- and long-axis B-mode data. The LVM = 1.05 [(5/6) A1 (L+T) - (5/6) A2 L], where 1.05 is the specific gravity of muscle; A1 and A2 are the epicardial and endocardial parasternal short-axis area, respectively; L is the parasternal long-axis length; and T is the wall thickness calculated from A1 and A2.

Ang II infusion and administration of bisoprolol. Mice were subjected to subcutaneous implantation of osmotic mini-pumps (ALZET model 1004, Durect Corporation, Cupertino, California, USA; supplied by Charles River UK Ltd., Kent, UK). After an incision was made on the back, slightly posterior to the scapulae, a distal subcutaneous pocket was created, and the pump was located posterior to the right flank. For Ang II infusion, osmotic mini-pumps (Alzet, model 1004) containing Ang II (1.44 µg/g/day dissolved in saline) were implanted in 10-week-old male Adamts5$^{\Delta Cat}$ and Adamts5$^{+/+}$ mice (JAX stock no. 005771, B6.129P2-Adamts5tm1Dgen/J). Animals were euthanized after 2 or 4 weeks, and the heart was excised and immediately washed in sterile PBS. During the surgical intervention, mice were anesthetised with 3% isoflurane mixed with 97% O2 (flow rate 1 L/min) and the mini-pumps were implanted via a 0.5 cm interscapular incision under sterile surgical conditions. The wound was closed with

an interrupted suture using a 6–0 silk thread. Bisoprolol was administered to 10-week-old male mice for 2 weeks in drinking water at a dose of 20 µg/g/day.

Western blot. Aliquots from GuHCl and total protein extracts were mixed with protein loading buffer and denatured at 98 °C for 5-10 min before being briefly centrifuged. Protein samples were separated on 4-12% Bis-Tris gradient gels at 130-170 V for 100-120 min in 1x NuPage MOPs SDS Running Buffer. Subsequently, the samples were transferred to nitrocellulose membranes in ice-cold transfer buffer (25 mM Tris-Base and 200mM Glycine in 20% methanol). Ponceau S red staining was used to ensure efficient transfer and equal loading. Next, membranes were blocked in 5% milk in PBS with 0.1% Tween-20 (PBS-T) for 1 hour on a shaker at room temperature. After a brief wash in PBS-T, they were incubated overnight with primary antibody solution. The following day, after three washes in PBS-T (15 min each), the membranes were incubated for 1 hour at room temperature with the HRP-conjugated secondary antibody diluted in 5% milk in PBS-T, according to the source of the primary antibody. The membranes were then washed three times in PBS-T for 15 min and developed using ECL western blotting detection reagent using either X-ray films (FUJIFILM) or Chemidoc Touch, Imaging System (BIORAD). Immunoblots were quantified by densitometry using Image-J software.

Reverse transcription and quantitative PCR. RNA extraction was performed using the miRNeasy Mini Kit (Qiagen) following the manufacturers' protocol. RNA concentration (Abs 260 nm) and purity (260/280) were measured in 1 µL of eluted RNA using spectrophotometry (NanoDrop ND-1000; Thermo Scientific). The RNA was then reverse transcribed using random hexamers with SuperScript VILO MasterMix (Invitrogen) according to manufacturers' protocol, with sample preparation being performed on a StarChill PCR rack to maintain low temperature. The reverse transcription reaction was set up by mixing the 8 µl of RNA with 2 µl of SuperScript VILO MasterMix. The reverse transcription reaction was performed in a Veriti thermal cycler (Applied Biosystem) with the following protocol: incubation at 25 °C for 10 min followed by 2 hours incubation at 42 °C. The reaction was terminated by incubation at 85 °C for 5 min. Samples were kept at 4 °C for immediate use. The reverse transcription product was diluted 1:15 to 1:25 using RNase-free water. TaqMan hydrolysis probes (Applied Biosystems) were used for quantitative PCR analysis according to the manufacturer's instructions. For custom-

made primers, quantitative PCR was performed using SybrGreen (Applied Biosystems) according to the manufacturer's instructions. Data were analyzed using ViiA 7 software (Applied Biosystems). GAPDH was used as reference gene throughout the study. Relative amounts of the targets were calculated using the $2^{-\Delta\Delta Cq}$ method, with statistical analysis performed on ΔCq values.

Histological analyses. Masson's trichrome staining, Picrosirius Red staining and hematoxylin and eosin staining were performed using standard protocols on 7 µm thick sections obtained from paraffin-embedded samples after overnight fixation in 4% paraformaldehyde in PBS at 4 °C. To detect hyaluronic acid in histological sections, frozen mouse heart samples embedded in OCT were cut using a cryostat (Cryostar NX-70) into 6 µm thick sections. They were then put on SuperfrostTM Ultra Plus Adhesion Slides (Thermo-Fischer Scientific) and stored at -20 °C until the process of staining. The frozen sections were kept at room temperature for 10 min before being fixed in cold acetone for 10 min. Then, the samples were washed 3 times in PBS-T (1xPBS plus 0.05% Tween 20) for 5 min each wash. The sections used as a negative control were treated with 40 U/ml hyaluronidase (H3506) for 1 hour at 37 °C, followed by three washes for 5 min with PBS-T. The endogenous avidin and biotin were blocked in the experimental sections using Avidin-Biotin Blocking Kit (Vector Lab: SP-2001). One drop of solution A was added per slide which was then incubated at room temperature for 10 min. This was followed by 2 washes for 5 min in PBS-T buffer. Next, one drop per slide of solution B was added followed by incubation at room temperature for 10 min. The slides were subsequently washed 2 times for 5 min in PBS-T buffer. They were then blocked with 10% FBS solution and incubated for 1 hour at room temperature. Biotinylated Hyaluronic Acid Binding Protein (HABP, derived from recombinant versican G1 domain, Cat: AMS.HKD-BC41 from AMSBIO, 1:200), was added to the tissue sections and incubated overnight at 4 °C. For uncleaved versican and for versikine staining, the corresponding antibodies were added at a 1:200 dilution in blocking buffer and incubated overnight at 4 °C. The next day, tissue sections were washed 3 times for 5 min with PBS-T. In order to detect the binding complex, Streptavidin conjugated Alexa Fluor 546 (cat: S11225, Life Technology) diluted in 10% FBS (1:400) was added and incubated for 1 hour at room temperature in the dark for hyaluronic acid detection. For versikine and versican detection

a donkey anti-rabbit secondary antibody conjugated with Alexa Fluor 594 (1:400, 10% serum) was used to incubate in the dark for 1 hour. This was followed by another 3 washes for 5 min in PBS-T. Then, the samples were counter-stained with DAPI at 1 ng/µl in PBS for 10 min and again, washed 3 times with PBS-T for 5 min each wash. The slides were then mounted with mounting medium (VectaMount) and stored in the dark for imaging. Imaging was performed at King's Nikon Imaging Centre using a Nikon Eclipse Ti spinning-disc confocal microscope. The images were acquired at 200x magnification. The image analysis was carried out with NIS Element 5.1.

Generation, overexpression and digestion of versican fragments. Synthetic double-strand DNA (gBLOCKS, Integrated DNA Technologies, UK) were designed to encode for the versican region comprising amino acids 1-560 of mouse versican V1 (Ensembl ID: ENSMUST00000159910.8) with a c-terminal myc-tag and flanked by restriction sites for *Nhe*I and *Sal*I. The designed fragments were either wild type versican or contained a single amino acid mutation (DPEAAA instead of DPEAAE) at the site of ADAMTS cleavage. An A-tailing reaction was performed on the synthetic DNA using GoTaq Hot Start Polymerase (Promega) according to the manufacturer's protocol. DNA fragments were inserted into a pGEMT vector which contains an open reading frame for prokaryotic replication and a lacZ marker. Introduction of an insert results in interruption of the β-galactosidase activity. The ligation reaction was performed using 2x Ligase Buffer and T4 ligase (Promega) following the manufacturer's guidelines. The reaction was left at room temperature for 1 hour and then stored at -20 °C. Next, Max Efficiency DH5α Competent Cells (*E.coli,* Invitrogen) were transformed using with 2.5 µl of the ligation reaction and following the manufacturer's recommendations. Transformed bacteria were then plated on a lysogenic broth containing ampicillin (LB Amp) agar plate, and containing X-gal and IPTG for colony selection based on β-galactosidase activity, and incubated overnight at 37 °C. Positive colonies were grown overnight in LB at 37 °C under constant agitation. The culture was centrifuged at 4,000 g for 10 min and the QIAprep Spin Miniprep kit (Qiagen) was used to extract the plasmid DNA from pellets following the manufacturer's instructions. Once the designed DNA fragments encoding for versican were inserted into the pGEMT vector, we sought to incorporate them into the pZac2.1 plasmid for expression in mammalian cells. A restriction

digest was performed in parallel on 5 μg of the pZac2.1 empty vector and 4 μg of the pGEMT containing the inserts of interest. *Nhe*I and *Sal*I restriction enzymes (New England Biolabs) were used per reaction following the manufacturer's guidelines. Digested samples were cleaned using GeneJet Gel Extraction and DNA Cleanup kit (ThermoFisher) according to manufacturer's instructions (Protocol B, for PCR cleanup and dimers removal). pZac2.1 concentration and purity were measured using spectrophotometry and digested vector was stored at -20 °C for future use. Agarose gel electrophoresis was performed with the digested and cleaned pGEMT plasmids DNA, and the gel slices containing the DNA fragments of interest were rapidly excised using a scalpel under UV light. DNA fragments were cleaned using the GeneJet Gel Extraction and DNA Cleanup kit (ThermoFisher) according to manufacturer's instructions (Protocol C, for DNA extraction from gel). After measuring concentration and purity, the DNA was stored at -20 °C. Before the ligation reaction, the digested pZac2.1 was dephosphorylated. The following reaction volumes were used: 7 μl of the double digested pZac2.1, 2 μl of 10x buffer and 1 μl of alkaline phosphatase (ThermoFisher). The reaction volume was adjusted with RNase-free water to 20 μl. Dephosphorylation was performed for 30 min at 37 °C followed by incubation for 20 min at 80 °C. The ligation reaction was performed for 250 ng of digested pZac2.1 and 250 ng of the double digested insert using 1 μl of T4 ligase in 1μl of 10x ligation buffer (Promega). The reaction volume was adjusted to 10 μl using RNase-free water and incubated overnight at room temperature. The following day, plasmid safe (Lucigen) was used to remove contaminating chromosomal DNA. The ligation reaction was topped up with 1 μl of water to make a total volume of 11 μl. Then, 1.5 μl of 10x reaction buffer, 1.5 μl of 10 mM ATP and 1 μl of plasmid safe enzyme (an ATP- dependent DNase) were added and the samples were left at 37 °C for 30 min. The competent cells were then transformed with 3 μl of the product as described before. The Amp agarose plates were incubated at 37 °C overnight. The next day, colonies were picked and screened for the presence of the insert using KAPA HiFi HotStart ReadyMix (Kapa Biosystems, Roche) according to the manufacturer's instructions. Colonies positive for the insert were selected and expanded in LB broth overnight at 37 °C under constant agitation. Then, the plasmid DNA was extracted using the QIAprep Spin Miniprep kit (Qiagen) and its concentration was measured before being sent for sequencing (SourceBioscience). HEK293T cells were

seeded in 6-well plates at a density of 500,000 cells per well using DMEM/F12 medium supplemented with 10% FBS. After 24 hours, the Lipofectamine 2000 Transfection Reagent (ThermoFisher) was used to transfect cells with 2 µg of empty pZac2.1 (control) or pZac2.1 containing the inserts for wildtype or mutant versican fragments, following the manufacturers' instructions. Conditioned media was collected after 24 hours and precipitated using acetone. Recombinant human ADAMTS5 (R&D Systems) was used to digest one quarter of the available conditioned medium resuspended in 20 µl of digestion buffer (5 mM CaCl2, 100 nM NaCl, 0.05% Brij35, 50 mM Tris, pH 7.5) for 6 hours at a protease concentration of 20 ng/µl. The digestion reaction was stopped using Western blot sample buffer and samples were kept at -20 °C until use.

Isolation and stimulation of murine cardiac fibroblasts (CFs). Primary CFs were isolated from the hearts of wild type C57Bl/6J female and male mice (4–7-week-old) by standard collagenase II–based digestion and maintained as separate cell lines. Briefly, the hearts were diced into small pieces, carefully washed in ice-cold phosphate buffered saline (PBS, Sigma-Aldrich) to remove plasma contaminants, followed by 2 washes in serum-free DMEM. The pieces were then pre-digested in collagenase II solution for 10 min at 37 ºC, 160 rpm. The collagenase II solution was replaced and the tissue pieces were incubated for 60-90 min at 37 ºC, 160 rpm. After collagenase II reaction was stopped by adding complete medium, the digested tissue pieces were plated in complete medium. Primary mouse CFs were cultured on gelatine 0.1% and grown in DMEM medium supplemented with L-Glutamine, 10% heat-inactivated FBS and antibiotics (100 U/ml penicillin and 100 µg/ml streptomycin) at 37 ºC in a humidified atmosphere of 95% air / 5% CO_2. Cells were detached with 0.05% trypsin for passaging. After two passages the cell population is mainly composed of cardiac fibroblasts. Cells at passage 2-4 were used in all experiments. For experiments involving noradrenalin (NA) and TGFβ1 stimulation, CFs were cultured in 6-well plates and treated with NA (Sigma-Aldrich, 10 µM) or murine recombinant TGFβ1 (R&D System, 10 ng/mL) in serum-free medium. Untreated cells were used as control. After 48 hours, cells were harvested for RNA isolation and quantitative PCR analysis. For experiments aimed to study the effect of β-blockers, CFs were seeded in 6-well plates in serum free medium and pre-treated with the β1 adrenergic receptor

antagonist bisoprolol (Merck, 100 µM). After 6 hours, NA (10 µM) was added alone to untreated CFs, or in combination to one well pre-treated with bisoprolol. Untreated cells and cells treated with bisoprolol-only were used as controls. CFs were incubated for 48 h and then harvested for RNA isolation and quantitative PCR analysis.

Statistical methods. GraphPad Prism software (version 9) was used for the analysis of data concerning *in vivo* and *in vitro* experiments. For each experiment, Shapiro–Wilk normality test was applied to test the distribution of the data. The appropriate tests and post hoc analysis were chosen according to the data distribution as specified in each figure legend. Paired tests were performed for experiments on which more than one data point was collected from the same animal or cell line as indicated in each figure legend. Data are represented as mean ± standard deviation (SD) or mean ± standard error of the mean (SEM) as indicated in each figure legend. The statistical analysis conducted on the demographics, clinical parameters and medication usage of the human heart failure cohort were conducted using non-parametric testing as indicated. TMT data were normalized against the reference channel to remove batch effects. The proteins retrieved by mass spectrometry experiments using the GuHCl extract have been filtered to keep the ECM related proteins using ECM protein annotation from the Matrisome Project and manually adding known ECM proteins that were missing. The label-free data were scaled using the total ion intensity. Both datasets were filtered to keep only proteins with less than 30% missing values or with more than 90% missing values one of the examined phenotypes and less than 10% for the rest of the phenotypes. In the latter case missing values of the phenotype which presented more than 90% of missing values were imputed with zeros. All remaining missing values were imputed with KNN-Impute method with k equal to 3 (default value). The relative quantities of the proteins were scaled using log2 transformation. The limma package[49] has been used for all statistical comparisons using the Ebayes algorithm and correcting for age and sex. The initial p-values were adjusted for multiple testing using Benjamini-Hochberg (BH) method and a threshold of 0.05 was used for the adjusted p-values to infer statistically significant changes. Beanplots, Volcano Plots and correlation plots were constructed using the Beanplots, Ggplot2 and Corrplot packages of R programming environment. Spearman correlation was used to correlate protein's relative expression levels

with continues variables and point biserial correlation to correlate protein's relative expression levels with categorical variables. Enrichment analysis was conducted using the DAVID tool. This analysis included pathway terms from Reactome data repository, KEGG and molecular function, cellular compartment and biological process annotations from Gene Ontology. Significantly enriched terms were inferred with Benjamini-Hochberg adjusted p-value threshold of 0.05. The total proteins revealed from each extract were used as a background for the enrichment analysis. Principal component analysis (PCA) was used for feature extraction using the scikit-learn python library version 0.19.2. The Scree test was used to retain an adequate number of principal components to maintain at least the 90% variability of the data set. The absolute weight of the contribution of its protein to each PCA was calculated and proteins were rank based on that value to identify the top contributing proteins in each PCA. Protein co-expression networks were reconstructed calculating the Spearman correlation among all ECM protein pairs and setting the Spearman correlation coefficient threshold performing grid search in the (0.5,0.9) interval and selecting the smallest value that provides a scale free network with an average degree of the network's nodes in between 3 and 6.[50] Network clustering and the calculation of p-values for each cluster were performed using ClusterOne and Cytoscape was used to visualize the network and significant subnetworks, e.g. the subnetwork with significantly changing proteins in the medications comparisons.

Supplemental Figure I

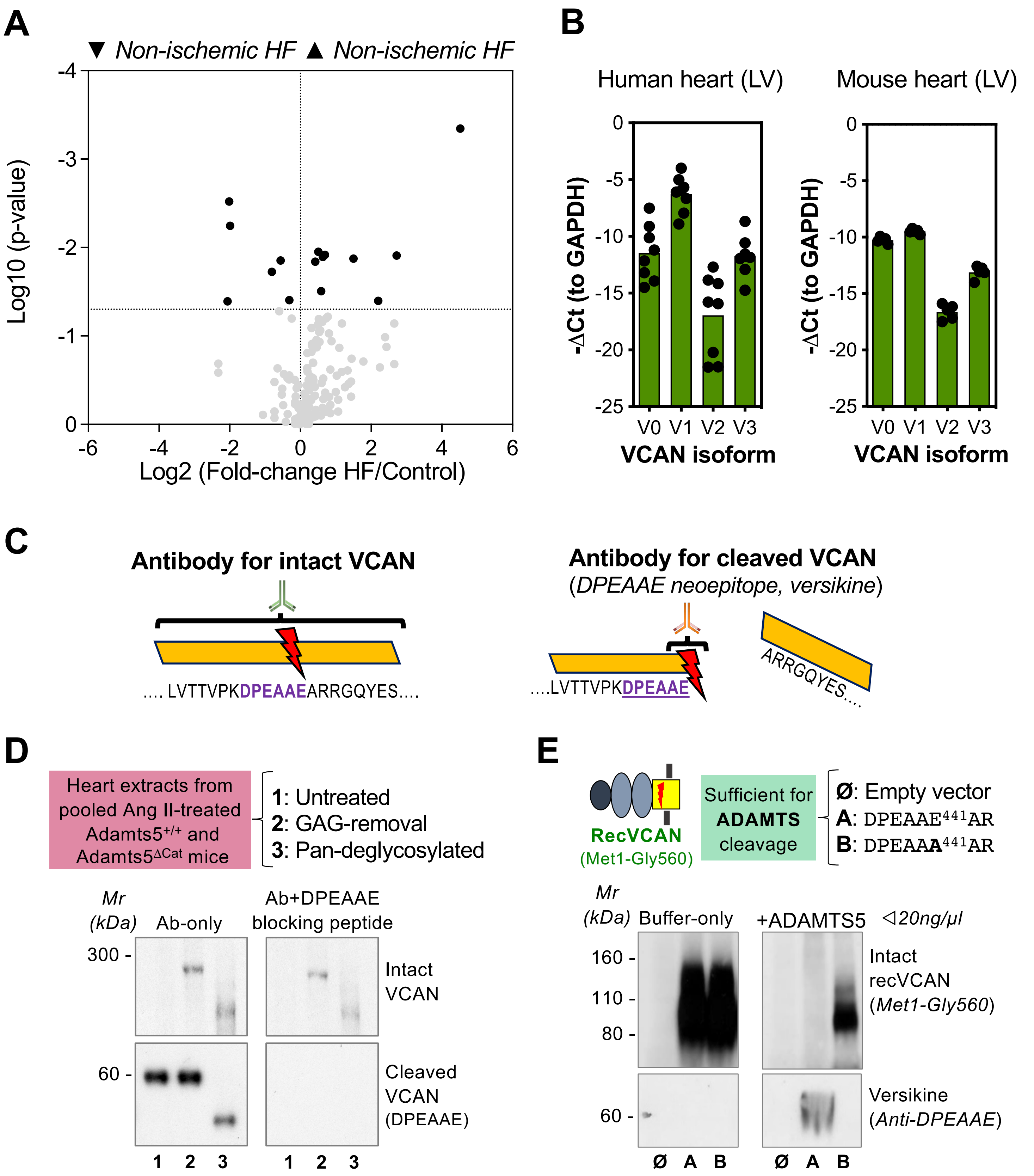

Supplemental Figure I. A) Volcano plot for the proteomics comparison between control (n=6) and non-ischemic HF (n=10) patients (GuHCl extracts). **B)** Gene expression levels of the four VCAN isoforms in human and mouse hearts (left ventricle; LV). In both species VCAN V1 is the predominant cardiac isoform. **C)** The antibodies used in this study are specific for either intact versican or cleaved versican. **D)** Co-incubation with the immunizing peptide used to generated the antibody for cleaved versican prevents detection of versikine by this antibody. The detection of versican by the antibody against the intact proteoglycan is not affected. **E)** Expression plasmids were used to generate recombinant versican (RecVCAN, expanding Met1 to Gly560). A single amino acid mutation to this cleavage site (i.e., DPEAAA instead of DPEAAE) prevents versican cleavage and generation of versikine, confirming the high level of specificity of the used antibodies and the specificity of ADAMTS5 for the cleavage site.

Supplemental Figure II

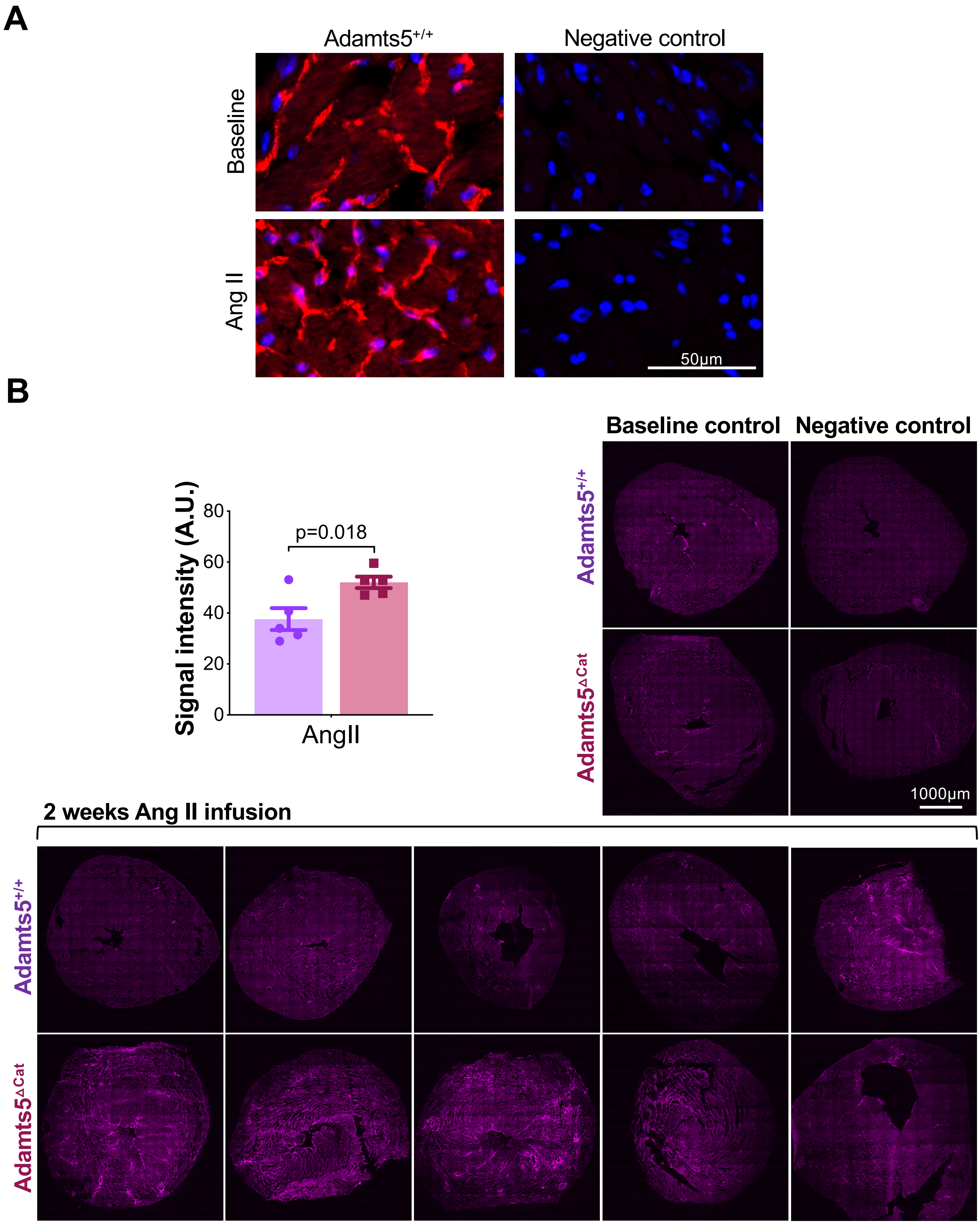

Supplemental Figure II. A) Detection of versikine in hearts of Adamts5$^{+/+}$ mice using an antibody against the neoepitope DPEAAE, generated by ADAMTS-mediated cleavage of versican. Versikine concentrates in areas surrounding cardiomyocytes at baseline and after Ang II infusion . **B)** Upon Ang II infusion, the lack of ADAMTS5 activity in Adamts5$^{\Delta Cat}$ mice leads to accumulation of the intact proteoglycan compared to Adamts5$^{+/+}$ mice. Quantification was performed in 20-30 areas of the left ventricle of each heart, and values represent the average intensity. P-value was calculated using a two-tailed unpaired t-test.

Supplemental Figure III

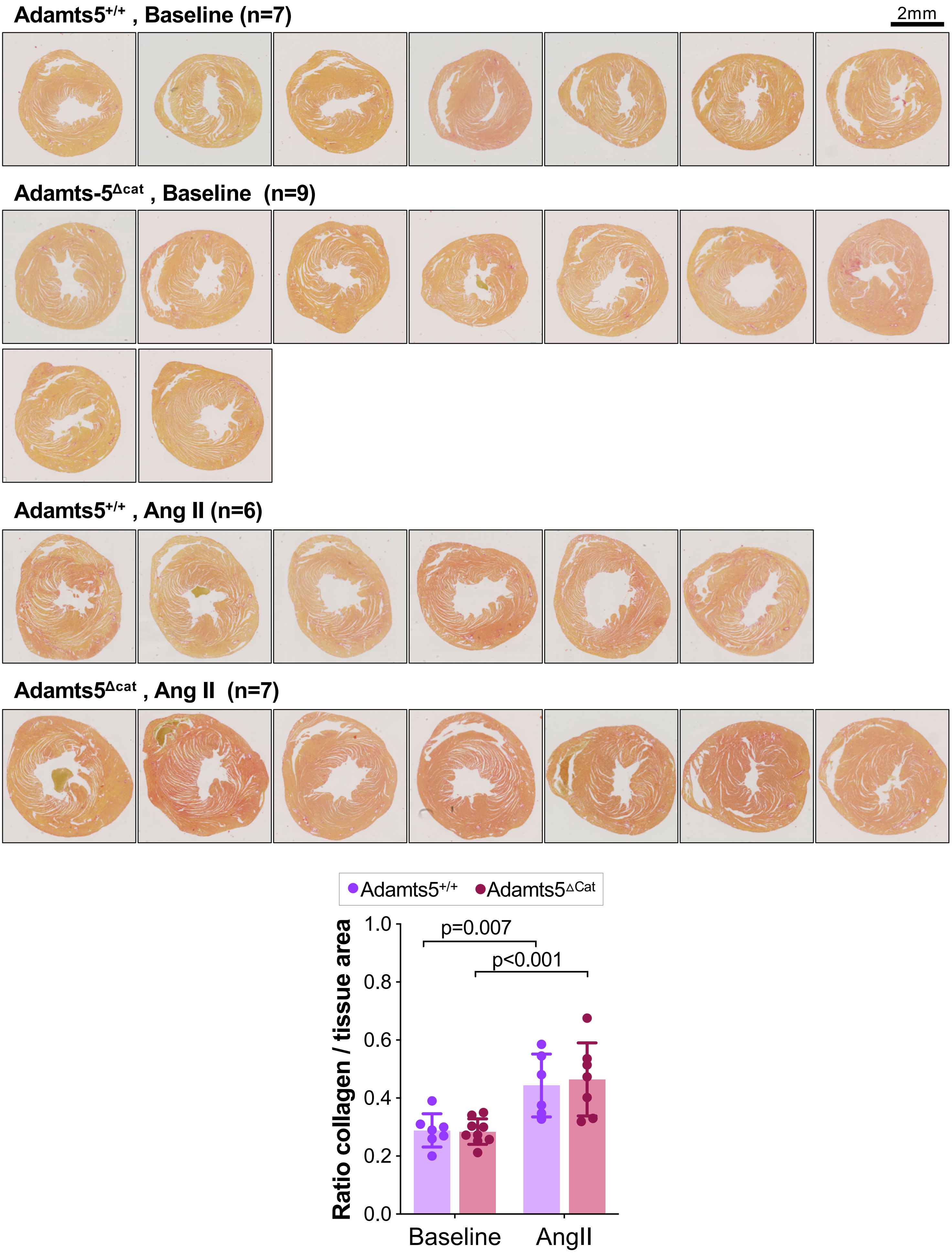

Supplemental Figure III. Picrosirius Red staining of Adamts5$^{+/+}$ and Adamts5$^{\Delta cat}$ mouse hearts. Mice were either untreated (baseline) or infused with Ang II for 2 weeks. Four areas of the left ventricle were quantified per sample, and dots represent sample averages (n=6-9). P-values were calculated using a two-way ANOVA with Bonferroni corrections.

Supplemental Figure IV

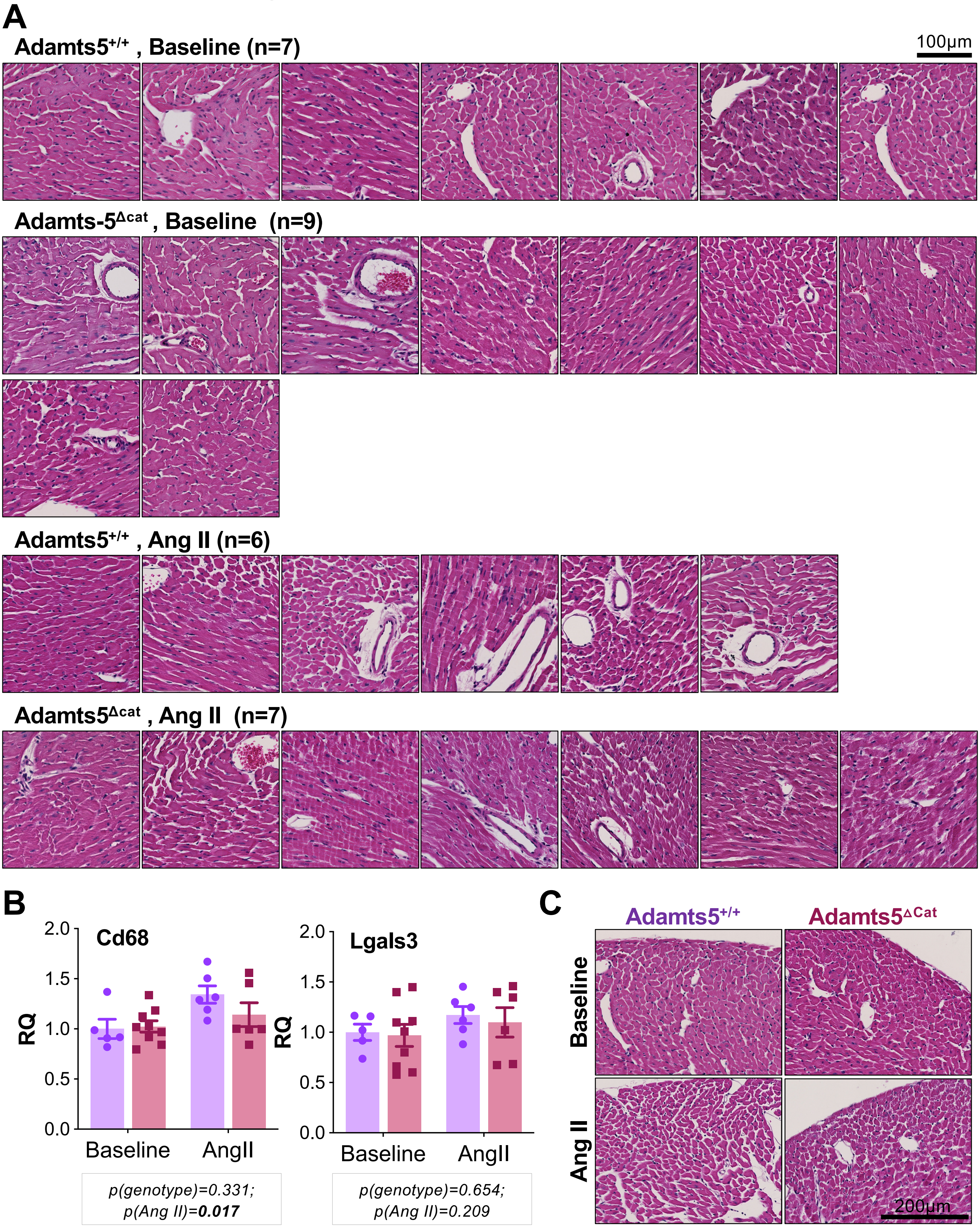

Supplemental Figure IV. A) Hematoxylin and eosin staining of Adamts5$^{+/+}$ and Adamts5$^{\Delta cat}$ mouse hearts. Mice were either untreated (baseline) or infused with Ang II for 2 weeks. No presence of inflammatory infiltrates was detected in the myocardial tissue or surrounding capillaries. **B)** We failed to detect significant differences in inflammatory cell infiltration between Adamts5$^{+/+}$ and Adamts5$^{\Delta cat}$ mice after AngII infusion as determined by gene expression levels of Cd68 and galectin 3 (Lgals3). P-values were derived from a two-way ANOVA with Bonferroni corrections. **C)** Edematous areas were found in the subepicardial region after Ang II infusion in both Adamts5$^{+/+}$ and Adamts5$^{\Delta cat}$ mice.

Supplemental Figure V

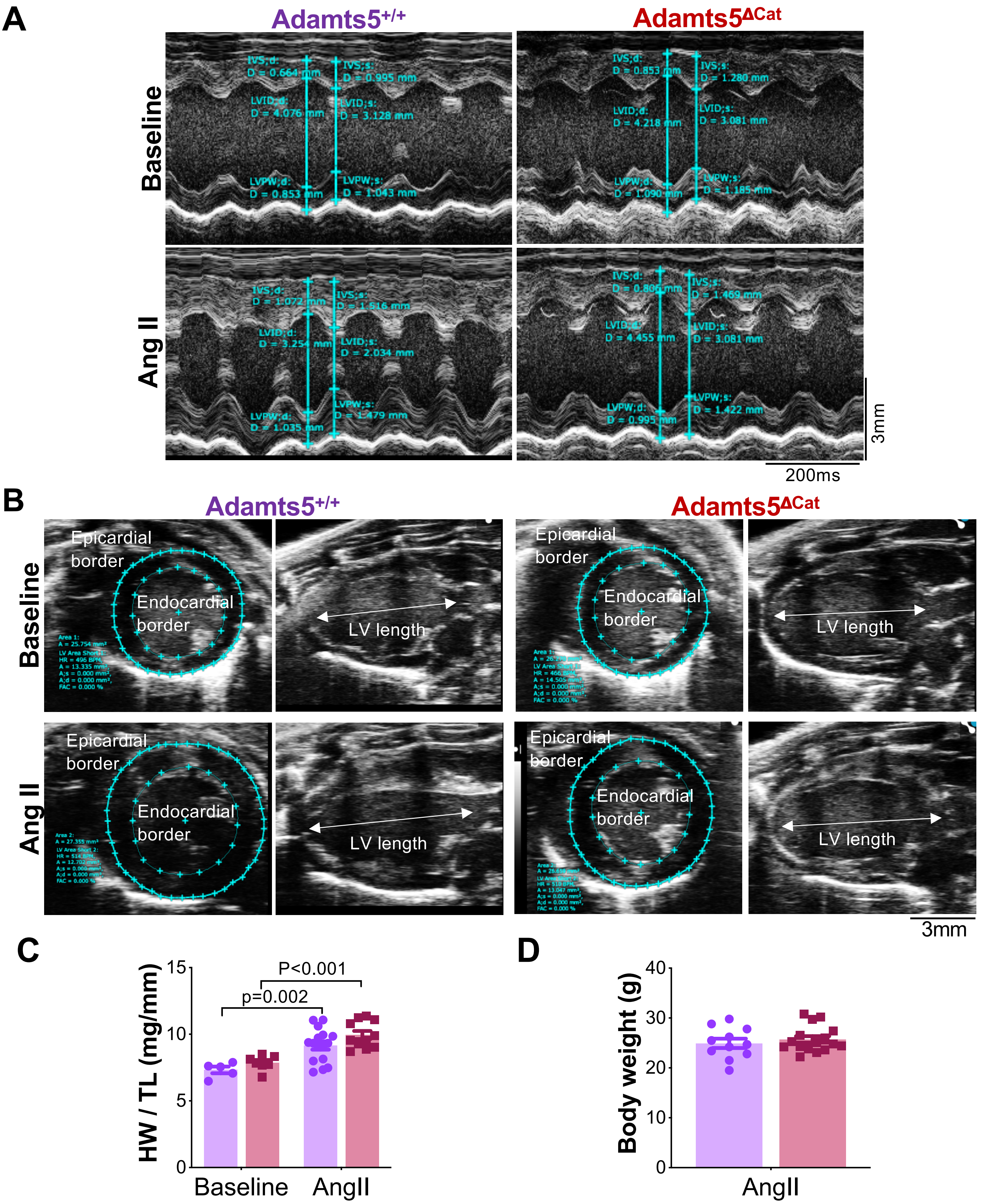

Supplemental Figure V. A) Representative LV M-mode echocardiography images at baseline and after 2 weeks of continuous Ang II infusion. **B)** Representative LV B-mode short-axis view over papillary muscle level and long-axis view. Endocardial area, epicardial area, and LV length were measured. **C)** Heart weight to tibial length ratios (HW/TL) in Adamts5$^{+/+}$ and Adamts5$^{\Delta cat}$ mice untreated (n=5 and n=7, respectively) and after 2 weeks of Ang II infusion (n=16 and n=11, respectively). P-values were calculated using a two-way ANOVA with Bonferroni corrections. All animals were 12 weeks of age at the time of harvesting. **D)** Body weight after 2 weeks of Ang II infusion was not different between Adamts5$^{+/+}$ and Adamts5$^{\Delta cat}$ mice (n=16 and n=11, respectively).

Supplemental Figure VI

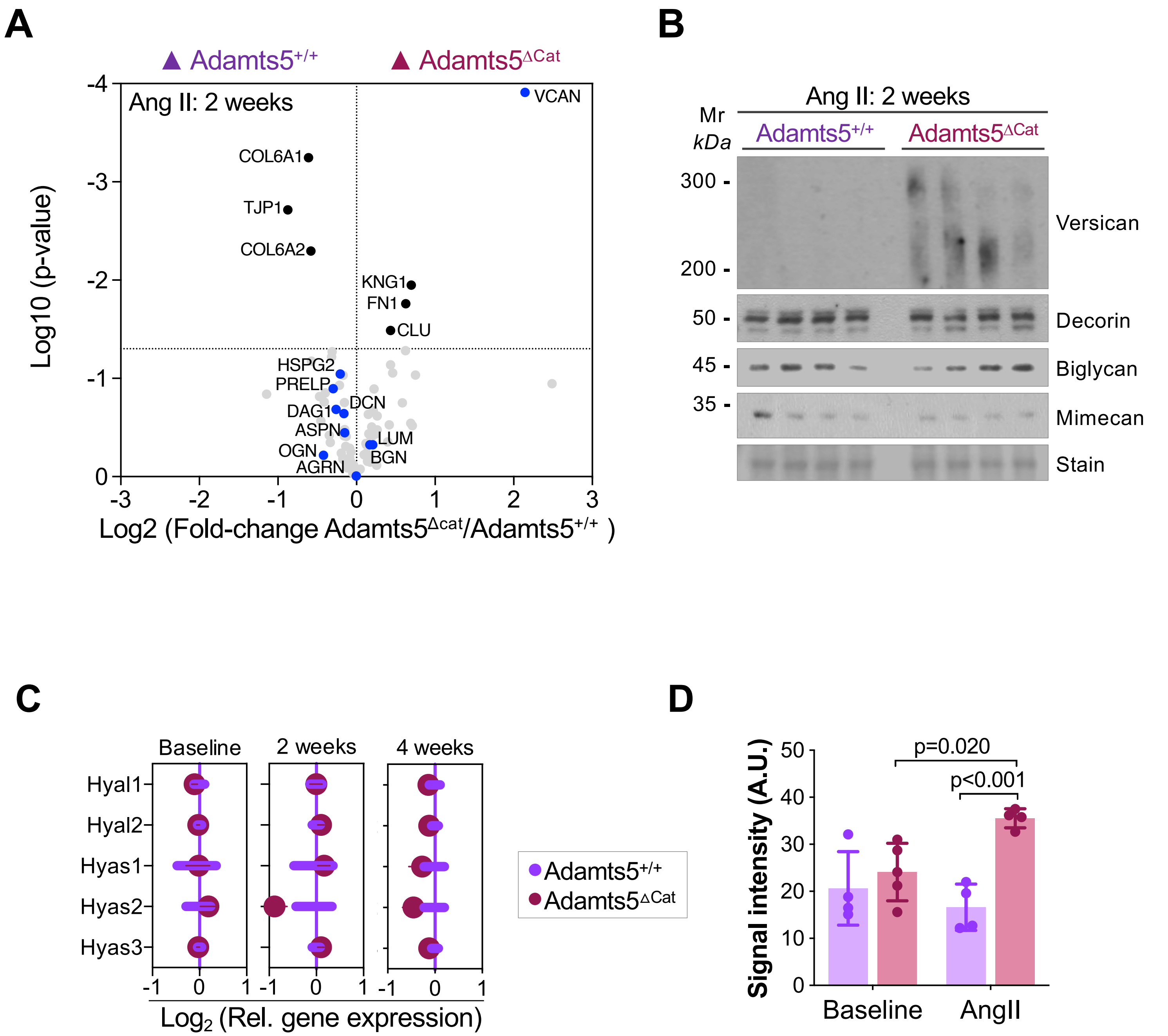

Supplemental Figure VI. A) Proteomics analysis of GuHCl extracts from Adamts5$^{+/+}$ (n=6) and Adamts5$^{\Delta cat}$ (n=4) mouse hearts after Ang II infusion for 2 weeks. Versican accumulation was the most prominent change. Other proteoglycans (labeled in blue) were not affected. Note the reduction in tight junction protein 1 (TJP1), a protein associated to CNX43 in cardiomyocyte junctions. **B)** Immunoblotting confirming the change in versican and the lack of differences in the detection of decorin, biglycan and mimecan. **C)** Comparison of gene expression level of hyaluronidases (Hyal1, Hyal2) and hyaluronic acid synthases (Hyas1, Hyas2, Hyas3) between Adamts5$^{+/+}$ and Adamts5$^{\Delta Cat}$ mice after Ang II treatment. No significant changes were observed between genotypes. **D)** Quantification of HABP signal intensity. Dots are averages per sample (n=4-5). P-values were derived from a two-way ANOVA with Bonferroni corrections.

Supplemental Figure VII

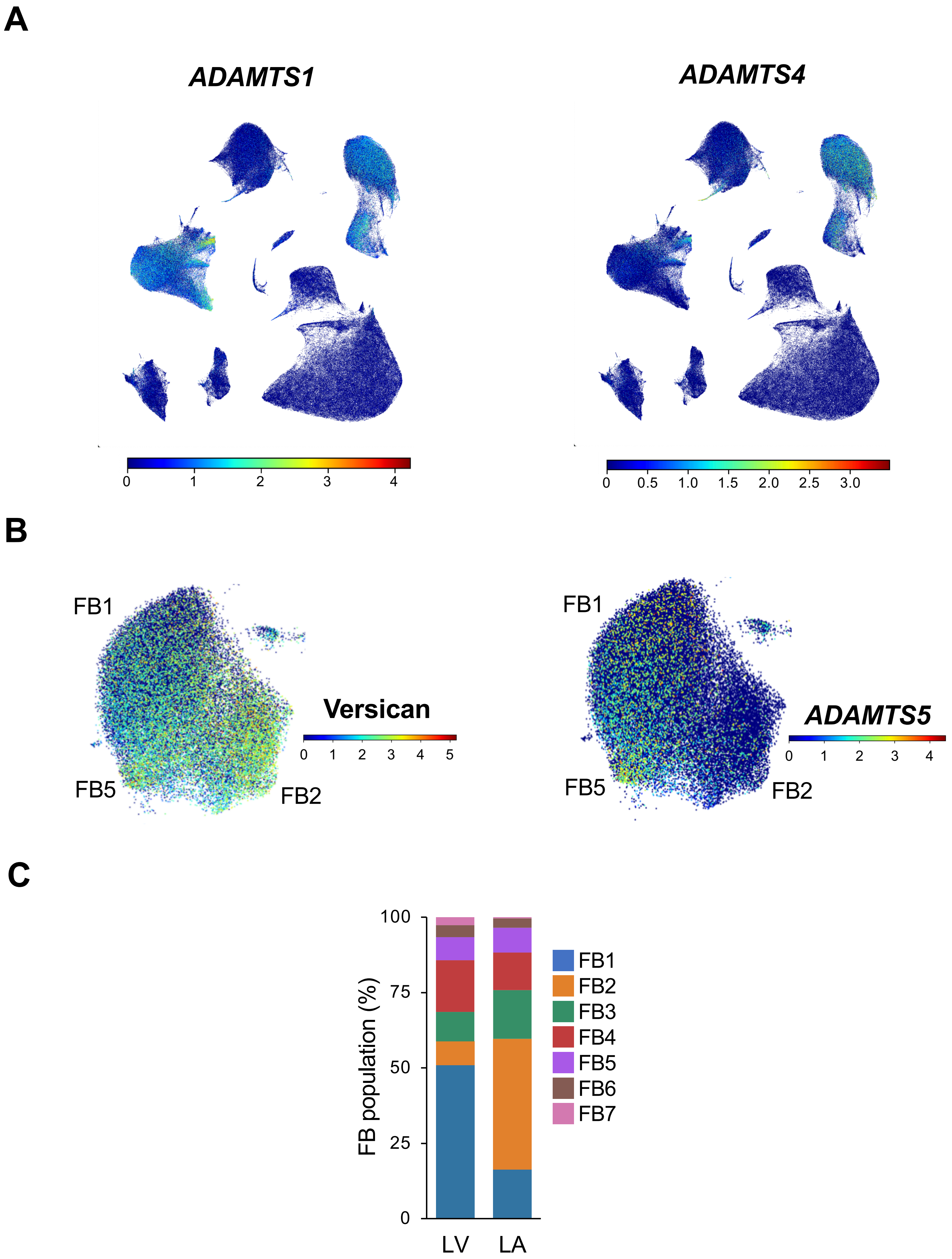

Supplemental Figure VII. A) ADAMTS1 is primarily expressed in endothelial cells, pericytes and smooth muscle cells. ADAMTS4 is mainly produced by pericytes and smooth muscle cells. **B)** Detail of the cardiac fibroblast cluster containing populations FB1, FB2 and FB5. **C)** Subpopulations of cardiac fibroblasts (FB1 to FB7) varied according to the different cardiac regions. FB1 was predominant in the LV while FB2 was mainly found in the left atrium (LA).

Supplemental Figure VIII

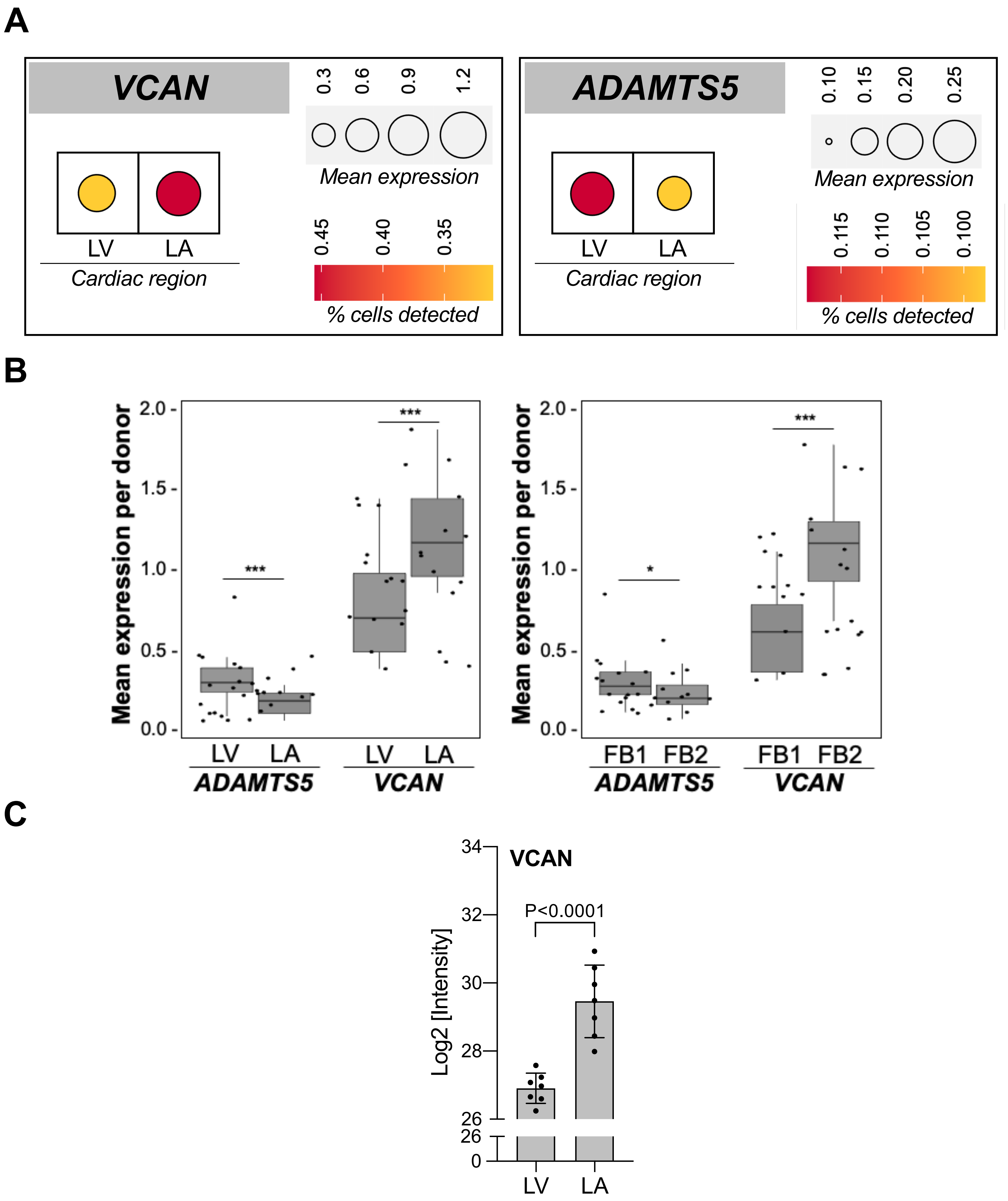

Supplemental Figure VIII. A) Aggregated expression for ADAMTS5 and VCAN in the LV and the LA displaying both levels of expression and the percentage of cells in which each transcript was detected. **B)** Average gene expression levels per donor for ADAMTS5 and VCAN in the left ventricle (LV) and the left atrium (LA) (left panel), and in the cardiac fibroblast populations FB1 and FB2 (right panel). P-values were derived from one-tailed Wilcoxon tests. . * $p<0.05$; *** $p<0.001$. **C)** Proteomics analysis of human cardiac tissue from LV (n=7, 61.8±12.2 years, 14.2% female) and LA (n=7, 67.7±6.7 years, p=0.373; 28.5% female, p=1.00) demonstrates increased versican accumulation in LA compared to LV. P-value derives from a two-tailed, unpaired Student’s t-test.

Supplemental Figure IX

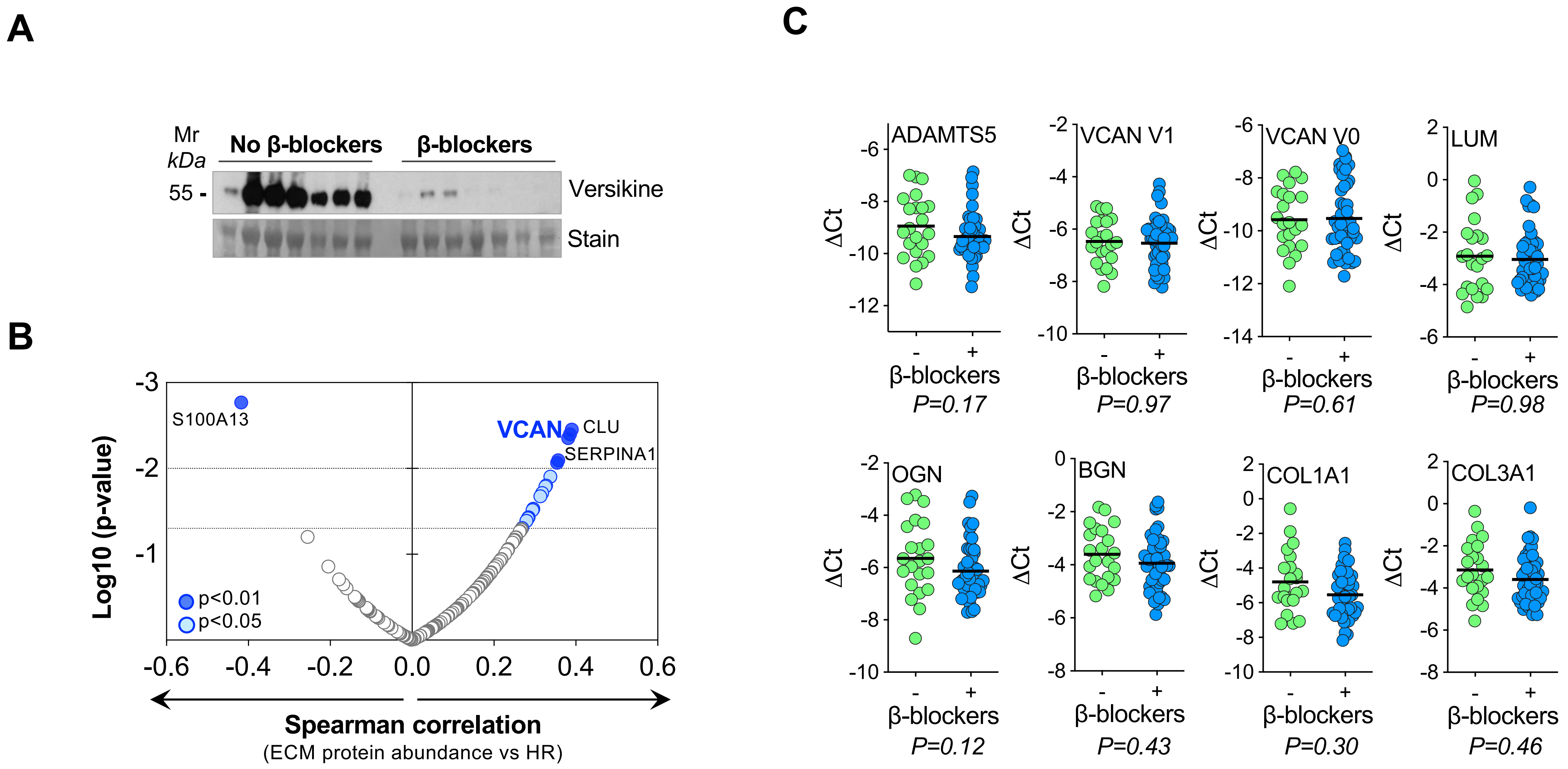

Supplemental Figure IX. A) Detection of versikine, the fragmentation product of VCAN, in GuHCl extracts from a selection of age- and sex-matched ischaemic HF patients grouped according to β-blocker usage. **B)** Correlation between ECM protein abundance and heart rate (HR) in ischemic HF patients. Among all ECM and ECM-related proteins detected in the GuHCl extracts, versican showed one of the highest correlation levels with heart rate. Other non-structural ECM-related proteins correlated with HR ($P<0.01$): clusterin (CLU), alpha-1-antitrypsin (SERPINA1) showed a positive correlation. S100 calcium-binding protein A13 (S100A13) was inversely correlated with HR. **C)** Gene expression levels of different ECM proteins in ischemic HF patients with (n=44) or without β-blocker medication (n=21). The limma package was used for p-value calculation using the Ebayes algorithm and correcting for age and sex.

Supplemental Figure X

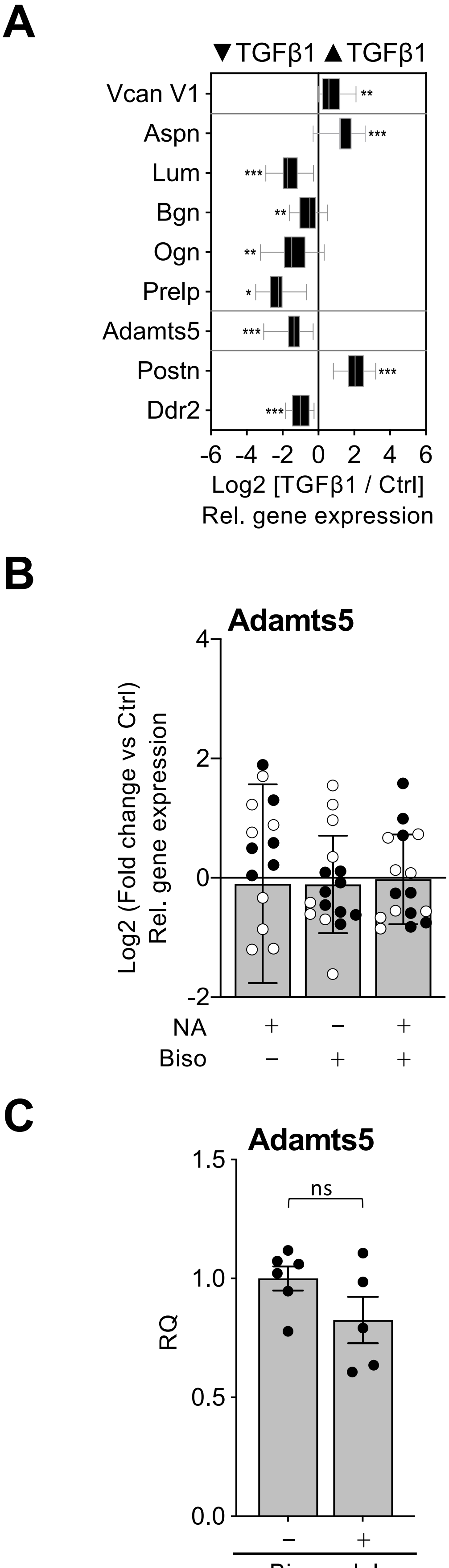

Supplemental Figure X. A) CFs isolated from wildtype female mice were stimulated with TGFβ1 as a positive control to stimulate ECM synthesis. P-values were derived from two-tailed paired t-tests with Benjamini corrections for multiple comparisons. * $p<0.05$; ** $p<0.01$; *** $p<0.001$. **B)** In CFs, the expression of ADAMTS5 was not altered upon treatment with noradrenaline, bisoprolol, or a combination of both. Males (n=8) are labelled as full black dots and females (n=8) in white dots. **C)** Compared to controls (n=6), administration of bisoprolol for 2 weeks (n=5) did not affect ADAMTS5 expression in wildtype mice. P-value was derived from a two-tailed unpaired t-test.

Supplemental Figure XI

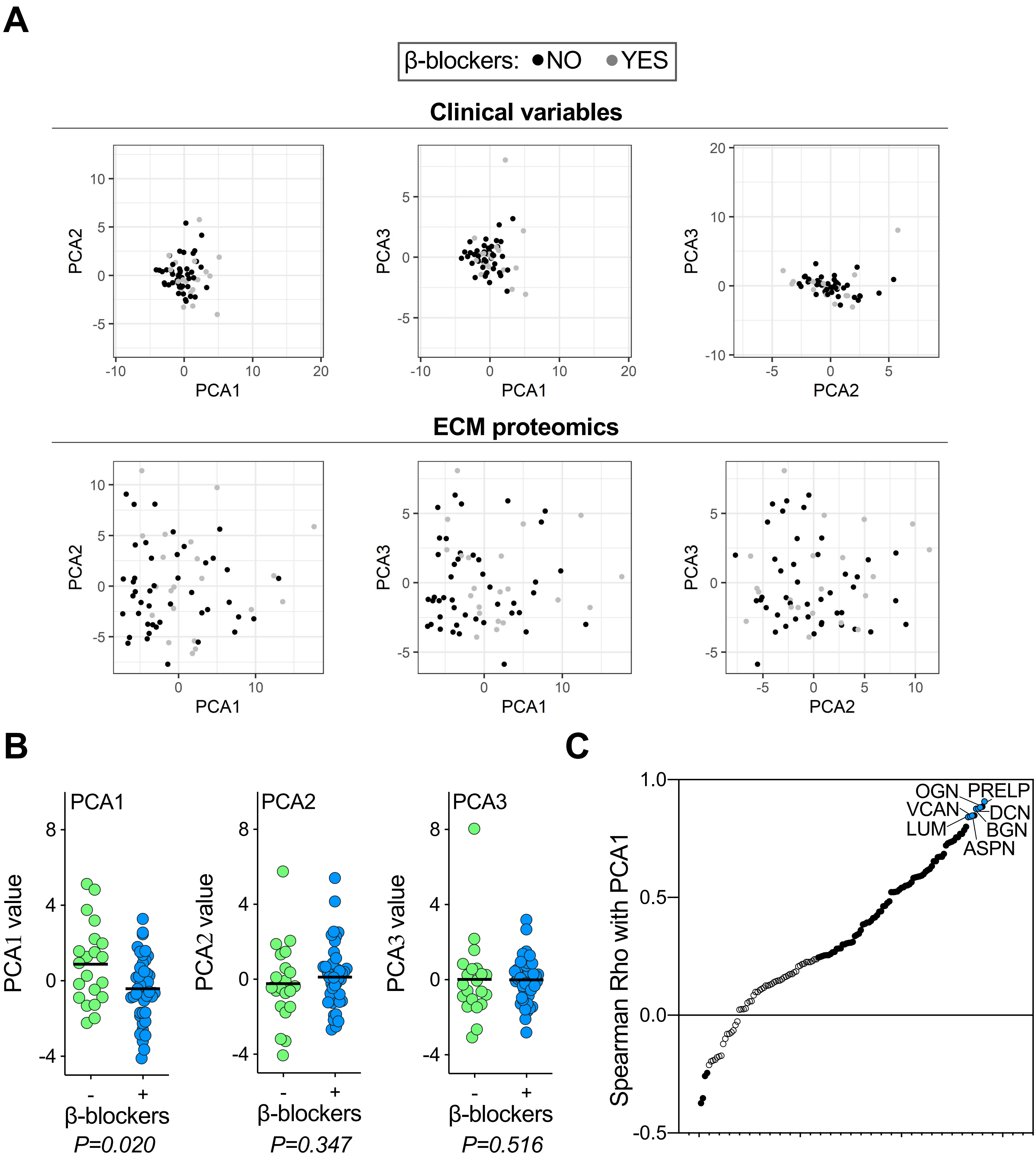

Supplemental Figure XI. A) Principal component analyses (PCA) based on clinical variables (top) or ECM proteomics (bottom). Patients with or without β-blocker administration shared similar clinical characteristics. Their ECM proteomic profiles, however, were distinct according to PCA1. **B)** A comparison of PCA values for ECM proteomics profiles between patients with (n=44) or without β-blocker administration (n=21) detected significant differences according to PCA1. The limma package was used for p-value calculation using the Ebayes algorithm and correcting for age and sex. **C)** Spearman correlation analysis between PCA1 and ECM protein abundance demonstrates that this principal component is predominantly determined by proteoglycan content (labelled in blue). Significant correlations ($p<0.05$) are represented by black dots. Abbreviations: asporin (ASPN), biglycan (BGN), decorin (DCN), lumican (LUM), mimecan (OGN), prolargin (PRELP), versican (VCAN).

Supplemental Table I. Proteomics analysis of cardiac ECM in control patients and patients with ischaemic HF and non-ischaemic HF.

Protein Information										Abundance Per Group (Average ± Standard Deviation)			Ischaemic vs Control			Non-ischaemic vs Control			Ischaemic vs Non-ischaemic		
**Protein Name**	Accesion number	Uniprot ID	Gene name	Coverage [%]	# AAs	# Peptides	# Unique Peptides	Total Spectrum Count	MW (kDa)	Control (n=6)	Ischaemic HF (n=5)	Idiopathic HF (n=10)	FC	P-value	BH Adjusted P-value	FC	P-value	BH Adjusted P-value	FC	P-value	BH Adjusted P-value
Adipocyte enhancer-binding protein 1	AEBP1_HUMAN	Q8IUX7	AEBP1	9.76	1158	16	15	158	131	0	18.0±15.6	6.8±8.1	N/A	N/A	N/A	N/A	N/A	N/A	2.65	0.1516	0.4382
Adiponectin	ADIPO_HUMAN	Q15848	ADIPOQ	21.7	244	4	4	80	26	3.0±3.95	4.4±2.6	4.0±3.2	1.47	0.3482	0.5780	1.33	0.4744	0.8169	1.10	0.7081	0.8998
Aggrecan core protein	PGCA_HUMAN	P16112	ACAN	2.65	2530	8	8	32	261	0	6.4±8.3	0	N/A	N/A	N/A	N/A	N/A	N/A	N/A	N/A	N/A
Agrin	AGRIN_HUMAN	O00468	AGRN	21.1	2068	55	55	1796	217	81.7±33.4	82.8±19.8	89.2±31.7	1.01	0.8045	0.9189	1.09	0.5661	0.8786	0.93	0.7681	0.9139
Alpha-1-acid glycoprotein 1	A1AG1_HUMAN	P02763	ORM1	45.8	201	13	7	314	24	0	19.2±14.3	14.6±5.5	1.60	0.2611	0.5264	1.22	0.2860	0.6918	1.32	0.5463	0.8047
Alpha-1-acid glycoprotein 2	A1AG2_HUMAN	P19652	ORM2	31.3	201	10	4	184	24	4.7±5.16	14.0±8.1	8.6±5.1	3.00	0.0196	0.0949	1.84	0.1146	0.4953	1.63	0.1508	0.4382
Alpha-1-antichymotrypsin	AACT_HUMAN	P01011	SERPINA3	27.7	423	17	17	590	48	29.0±17.6	36.8±17.1	23.2±16.6	1.27	0.4835	0.6942	0.80	0.3105	0.6918	1.59	0.1417	0.4276
Alpha-1-antitrypsin	A1AT_HUMAN	P01009	SERPINA1	50.5	418	26	26	2346	47	104.0±14.8	118.0±38.7	113.2±43.5	1.13	0.6071	0.7684	1.09	0.8509	0.9391	1.04	0.7759	0.9139
Alpha-1B-glycoprotein	A1BG_HUMAN	P04217	A1BG	34.3	495	23	23	542	54	16.0±3.3	41.2±19.7	24.0±7.9	2.58	0.0013	0.0352	1.50	0.0312	0.3879	1.72	0.0253	0.1711
Alpha-2-HS-glycoprotein	FETUA_HUMAN	P02765	AHSG	19.3	367	6	6	32	39	0	4.0±4.2	1.2±1.9	N/A	N/A	N/A	N/A	N/A	N/A	3.33	0.1323	0.4261
Alpha-2-macroglobulin	A2MG_HUMAN	P01023	A2M	11	1474	16	16	212	163	3.0±2.4	28.8±19.3	5.0±5.8	9.60	0.0031	0.0434	1.67	0.7216	0.9349	5.76	0.0063	0.0904
Angiogenin	ANGI_HUMAN	P03950	ANG	21.1	147	3	3	12	17	0	1.6±3.6	0.4±1.3	N/A	N/A	N/A	N/A	N/A	N/A	4.00	0.4221	0.7375
Annexin A1	ANXA1_HUMAN	P04083	ANXA1	48.6	346	18	18	410	39	14.7±11.7	20.0±14.8	22.2±21.6	1.36	0.3561	0.5802	1.51	0.3406	0.7147	0.90	0.9709	0.9921
Annexin A11	ANX11_HUMAN	P50995	ANXA11	17	505	17	17	412	54	29.0±12.4	14.4±10.0	16.6±5.9	0.50	0.0852	0.2685	0.57	0.0188	0.2551	0.87	0.2667	0.6236
Annexin A2	ANXA2_HUMAN	P07355	ANXA2	61.4	339	24	24	1278	39	51.7±27.6	72.0±38.3	60.8±22.5	1.39	0.3418	0.5763	1.18	0.3131	0.6918	1.18	0.6741	0.8866
Annexin A4	ANXA4_HUMAN	P09525	ANXA4	15	319	3	3	34	36	0.7±1.6	2.4±3.6	1.8±3.3	3.60	0.3259	0.5693	2.70	0.4864	0.8169	1.33	0.6998	0.8998
Annexin A5	ANXA5_HUMAN	P08758	ANXA5	13.4	320	5	5	76	36	4.0±3.6	4.8±4.4	2.8±4.5	1.20	0.7484	0.8877	0.70	0.3930	0.7413	1.71	0.2478	0.5969
Annexin A6	ANXA6_HUMAN	P08133	ANXA6	9.36	673	13	13	92	76	4.0±5.1	5.6±7.8	4.0±4.0	1.40	0.9366	0.9700	1.00	0.8469	0.9391	1.40	0.9422	0.9856
Antileukoproteinase	SLPI_HUMAN	P03973	SLPI	12.9	132	2	2	4	14	0	0.8±1.8	0	N/A	N/A	N/A	N/A	N/A	N/A	N/A	N/A	N/A
Antithrombin-III	ANT3_HUMAN	P01008	SERPINC1	30	464	13	13	268	53	8.7±10.8	17.6±9.9	12.8±7.2	2.03	0.0850	0.2685	1.48	0.0863	0.4640	1.38	0.3328	0.7055
Apolipoprotein A-I	APOA1_HUMAN	P02647	APOA1	68.2	267	31	30	1644	31	63.3±45.9	79.2±14.8	86.8±30.4	1.25	0.2542	0.5264	1.37	0.1114	0.4953	0.91	0.8022	0.9310
Apolipoprotein A-II	APOA2_HUMAN	P02652	APOA2	74	100	9	9	134	11	4.3±3.9	8.0±3.2	6.8±5.2	1.85	0.1085	0.3083	1.57	0.3035	0.6918	1.18	0.3931	0.7375
Apolipoprotein A-IV	APOA4_HUMAN	P06727	APOA4	50.8	396	22	22	358	45	1±2.45	24.4±15.06	23.0±14.4	24.40	<0.0001	0.0038	23.00	0.0005	0.0671	1.06	0.5727	0.8263
Apolipoprotein C-I	APOC1_HUMAN	P02654	APOC1	34.9	83	5	5	18	9	1.3±3.3	0	1.0±2.2	N/A	N/A	N/A	0.75	0.9401	0.9811	N/A	N/A	N/A
Apolipoprotein C-III	APOC3_HUMAN	P02656	APOC3	30.3	99	3	3	98	11	2.3±3.7	3.6±3.6	6.6±2.7	1.54	0.4927	0.7004	2.83	0.0133	0.2149	0.55	0.0956	0.3578
Apolipoprotein D	APOD_HUMAN	P05090	APOD	30.7	189	6	6	46	21	1.0±2.4	5.6±3.6	1.2±2.5	5.60	0.0228	0.1034	1.20	0.8768	0.9467	4.67	0.0142	0.1396
Apolipoprotein E	APOE_HUMAN	P02649	APOE	20.8	317	8	8	68	36	0.7±1.6	10.0±8.2	1.4±3.3	15.00	0.0100	0.0689	2.10	0.7149	0.9349	7.14	0.0089	0.1092
Asporin	ASPN_HUMAN	Q9BXN1	ASPN	56.6	380	27	27	2248	43	77.0±45.4	205.2±78.9	76.0±33.0	2.66	0.0084	0.0675	0.99	0.9560	0.9824	2.70	0.0029	0.0573
Basal cell adhesion molecule	BCAM_HUMAN	P50895	BCAM	38.1	628	23	23	906	67	37.3±7.8	43.6±13.8	46.4±11.5	1.17	0.4166	0.6358	1.24	0.0812	0.4640	0.94	0.6074	0.8493
Basement membrane-specific heparan sulfate proteoglycan co	PGBM_HUMAN	P98160	HSPG2	39.4	4391	189	189	19572	469	934.3±80.6	893.6±271.5	949.8±80.0	0.96	0.5984	0.7684	1.02	0.7119	0.9349	0.94	0.4170	0.7375
Beta-2-glycoprotein 1	APOH_HUMAN	P02749	APOH	64.1	345	21	21	2134	38	86.0±22.3	134.0±20.5	94.8±41.6	1.56	0.0115	0.0696	1.10	0.6754	0.9318	1.41	0.2853	0.6389
Beta-2-microglobulin	B2MG_HUMAN	P61769	B2M	46.2	119	7	7	154	14	6.3±2.3	7.6±2.6	7.8±4.0	1.20	0.4403	0.6581	1.23	0.7733	0.9391	0.97	0.8096	0.9328
Biglycan	PGS1_HUMAN	P21810	BGN	59.8	368	31	30	5022	42	189.3±65.0	398.0±149.9	189.6±54.3	2.10	0.0186	0.0930	1.00	0.9482	0.9811	2.10	0.0044	0.0769
Cadherin-13	CAD13_HUMAN	P55290	CDH13	15.8	713	15	15	1108	78	48.7±17.3	52.0±29.2	55.6±6.5	1.07	0.8861	0.9446	1.14	0.1561	0.5410	0.94	0.3187	0.6877
Calpastatin	ICAL_HUMAN	P20810	CAST	28.7	708	21	21	680	77	24.7±15.4	20±20.2	43.2±21.6	0.81	0.3067	0.5574	1.75	0.0934	0.4640	0.46	0.0258	0.1711
Cartilage intermediate layer protein 1	CILP1_HUMAN	O75339	CILP	8.28	1184	11	11	134	133	1.3±3.3	21.6±35.5	1.8±3.0	16.20	0.0896	0.2730	1.35	0.6862	0.9349	12.00	0.0663	0.2850
Cartilage intermediate layer protein 2	CILP2_HUMAN	Q8IUL8	CILP2	1.9	1156	2	2	4	126	0	0.8±1.8	0	N/A	N/A	N/A	N/A	N/A	N/A	N/A	N/A	N/A
Cartilage oligomeric matrix protein	COMP_HUMAN	P49747	COMP	5.68	757	2	2	4	83	0	0.8±1.8	0	N/A	N/A	N/A	N/A	N/A	N/A	N/A	N/A	N/A
Cathepsin B	CATB_HUMAN	P07858	CTSB	14.5	339	9	9	98	38	4.3±3.4	4.4±3.6	5.0±7.0	1.02	0.9255	0.9655	1.15	0.9175	0.9765	0.88	0.8394	0.9376
Cathepsin D	CATD_HUMAN	P07339	CTSD	45.4	412	23	23	2608	45	142.0±35.4	92.8±33.0	129.2±33.2	0.65	0.0472	0.1799	0.91	0.4818	0.8169	0.72	0.0532	0.2563
Cathepsin G	CATG_HUMAN	P08311	CTSG	25.1	255	7	7	178	29	2.0±2.2	14.8±18.1	9.2±8.3	7.40	0.1514	0.3989	4.60	0.0401	0.4036	1.61	0.9899	0.9962
Cathepsin L1	CATL1_HUMAN	P07711	CTSL	8.41	333	2	2	4	38	0	0	0.4±1.3	N/A	N/A	N/A	N/A	N/A	N/A	N/A	N/A	N/A
Cathepsin Z	CATZ_HUMAN	Q9UBR2	CTSZ	8.58	303	2	2	8	34	0	1.6±2.2	0	N/A	N/A	N/A	N/A	N/A	N/A	N/A	N/A	N/A
CD109 antigen	CD109_HUMAN	Q6YHK3	CD109	3.53	1445	3	3	6	162	0	1.2±2.7	0	N/A	N/A	N/A	N/A	N/A	N/A	N/A	N/A	N/A
CD5 antigen-like	CD5L_HUMAN	O43866	CD5L	9.22	347	2	2	4	38	0	0.8±1.8	0	N/A	N/A	N/A	N/A	N/A	N/A	N/A	N/A	N/A
CD59 glycoprotein	CD59_HUMAN	P13987	CD59	19.5	128	3	3	28	14	0	1.6±3.6	2.0±2.7	N/A	N/A	N/A	N/A	N/A	N/A	0.80	0.6367	0.8619
CD97 antigen	CD97_HUMAN	P48960	ADGRE5	6.83	835	5	5	20	92	0.7±1.6	0.8±1.8	1.2±2.7	1.20	0.8957	0.9446	1.80	0.7454	0.9391	0.67	0.8566	0.9400
Cell surface glycoprotein MUC18	MUC18_HUMAN	P43121	MCAM	6.66	646	6	6	52	72	1.3±2.1	4.0±3.7	2.4±3.4	3.00	0.2107	0.4775	1.80	0.6021	0.8863	1.67	0.3999	0.7375
Chymase	CMA1_HUMAN	P23946	CMA1	46.6	247	12	12	1144	27	41.7±37.2	80.0±19.9	49.4±38.0	1.92	0.0923	0.2730	1.19	0.6324	0.9060	1.62	0.1367	0.4261
Clusterin	CLUS_HUMAN	P10909	CLU	35	449	19	19	1506	52	59.7±16.7	116.4±59.0	56.6±23.0	1.95	0.0560	0.2082	0.95	0.6067	0.8863	2.06	0.0211	0.1644
Collagen alpha-1(I) chain	CO1A1_HUMAN	P02452	COL1A1	65.6	1464	89	85	7896	139	217.3±70.6	693.2±574.6	312.6±122.2	3.19	0.0122	0.0710	1.44	0.0897	0.4640	2.22	0.0355	0.2006
Collagen alpha-1(II) chain	CO2A1_HUMAN	P02458	COL2A1	4.24	1487	7	1	108	142	0	14.4±25.1	3.6±5.9	N/A	N/A	N/A	N/A	N/A	N/A	4.00	0.4460	0.7545
Collagen alpha-1(III) chain	CO3A1_HUMAN	P02461	COL3A1	56.8	1466	71	70	3750	139	100.3±30.6	318.0±194.8	155.8±47.8	3.17	0.0033	0.0434	1.55	0.0127	0.2149	2.04	0.0153	0.1396
Collagen alpha-1(IV) chain	CO4A1_HUMAN	P02462	COL4A1	8.81	1669	13	11	638	161	28.3±18.6	40.0±25.4	26.8±10.2	1.41	0.5076	0.7146	0.95	0.8475	0.9391	1.49	0.4393	0.7511
Collagen alpha-1(V) chain	CO5A1_HUMAN	P20908	COL5A1	11.2	1838	26	23	672	184	15.3±12.4	63.6±62.4	26.2±11.4	4.15	0.0344	0.1386	1.71	0.0604	0.4440	2.43	0.0646	0.2850
Collagen alpha-1(VI) chain	CO6A1_HUMAN	P12109	COL6A1	40.7	1028	52	52	4590	109	171.7±62.0	281.2±145.7	215.4±50.3	1.64	0.2741	0.5370	1.25	0.1237	0.4953	1.31	0.6196	0.8566
Collagen alpha-1(XII) chain	COCA1_HUMAN	Q99715	COL12A1	3.82	3063	12	12	42	333	0	8.4±11.5	0	N/A	N/A	N/A	N/A	N/A	N/A	N/A	N/A	N/A
Collagen alpha-1(XIV) chain	COEA1_HUMAN	Q05707	COL14A1	15.1	1796	27	27	220	194	0.7±1.6	36.0±23.4	3.6±4.2	54.00	0.0023	0.0369	5.40	0.1332	0.4953	10.00	0.0084	0.1092
Collagen alpha-1(XV) chain	COFA1_HUMAN	P39059	COL15A1	10.7	1388	16	16	882	142	32.7±6.9	42.8±21.4	47.2±18.2	1.31	0.7027	0.8491	1.44	0.0903	0.4640	0.91	0.4936	0.7844
Collagen alpha-1(XVIII) chain	COIA1_HUMAN	P39060	COL18A1	8.38	1754	22	22	1280	178	43.0±7.7	72.4±44.8	66.0±28.3	1.68	0.1540	0.3989	1.53	0.0693	0.4440	1.10	0.9102	0.9585
Collagen alpha-1(XXVIII) chain	COSA1_HUMAN	Q2UY09	COL28A1	4	1125	3	3	24	117	2.0±3.3	1.6±3.6	0.4±1.3	0.80	0.7665	0.8963	0.20	0.2052	0.6320	4.00	0.4221	0.7375
Collagen alpha-2(I) chain	CO1A2_HUMAN	P08123	COL1A2	71.2	1366	88	88	9016	129	260.3±102.6	742.0±538.9	374.4±136.6	2.85	0.0097	0.0689	1.44	0.0702	0.4440	1.98	0.0366	0.2006
Collagen alpha-2(IV) chain	CO4A2_HUMAN	P08572	COL4A2	9.52	1712	19	19	892	168	39.0±20.9	51.6±17.9	40.0±14.4	1.32	0.2896	0.5539	1.03	0.7186	0.9349	1.29	0.2756	0.6261
Collagen alpha-2(V) chain	CO5A2_HUMAN	P05997	COL5A2	39.8	1499	42	40	1476	145	36.3±21.1	131.2±110.4	60.2±31.0	3.61	0.0182	0.0930	1.66	0.0632	0.4440	2.18	0.0624	0.2833
Collagen alpha-2(VI) chain	CO6A2_HUMAN	P12110	COL6A2	30.3	1019	40	40	1960	109	74.7±33.2	116.4±74.2	93.0±24.4	1.56	0.6277	0.7780	1.25	0.1510	0.5359	1.25	0.8728	0.9400
Collagen alpha-2(VIII) chain	CO8A2_HUMAN	P25067	COL8A2	4.27	703	2	2	4	67	0	0.8±1.8	0	N/A	N/A	N/A	N/A	N/A	N/A	N/A	N/A	N/A
Collagen alpha-3(V) chain	CO5A3_HUMAN	P25940	COL5A3	10.8	1745	15	15	420	172	11±10.71	36.8±33.54	17.0±15.7	3.35	0.0722	0.2491	1.55	0.6486	0.9192	2.16	0.1687	0.4624

Collagen alpha-3(VI) chain	CO6A3_HUMAN	P12111	COL6A3	33.1	3177	157	157	16586	344	619.3±257.8	1005.6±573.3	784.2±204.9	1.62	0.3107	0.5574	1.27	0.1315	0.4953	1.28	0.7206	0.8998
Collagen alpha-6(VI) chain	CO6A6_HUMAN	A6NMZ7	COL6A6	1.1	2263	3	3	12	247	0	1.6±3.6	0.4±1.3	N/A	N/A	N/A	N/A	N/A	N/A	4.00	0.4221	0.7375
Cystatin-B	CYTB_HUMAN	P04080	CSTB	33.7	98	3	3	36	11	1.3±3.3	2.8±3.9	1.4±3.0	2.10	0.4502	0.6661	1.05	0.9245	0.9769	2.00	0.4289	0.7412
Cysteine-rich protein 2	CRIP2_HUMAN	P52943	CRIP2	52.4	208	14	14	1164	22	47.0±16.3	50.4±12.8	63.0±9.0	1.07	0.6143	0.7684	1.34	0.0144	0.2149	0.80	0.0415	0.2201
Decorin	PGS2_HUMAN	P07585	DCN	77.4	359	47	46	7986	40	278.3±76.6	628.0±206.0	317.6±61.2	2.26	0.0021	0.0369	1.14	0.2310	0.6657	1.98	0.0004	0.0245
Dermatopontin	DERM_HUMAN	Q07507	DPT	58.7	201	14	14	1256	24	34.0±21.7	125.2±59.6	42.6±10.5	3.68	0.0046	0.0551	1.25	0.1265	0.4953	2.94	0.0001	0.0180
Dystroglycan	DAG1_HUMAN	Q14118	DAG1	9.05	895	12	12	586	97	30.7±10.9	25.2±7.7	27.6±8.7	0.82	0.4163	0.6358	0.90	0.5871	0.8863	0.91	0.6747	0.8866
EMILIN-1	EMIL1_HUMAN	Q9Y6C2	EMILIN1	7.87	1016	15	15	254	107	3.7±4.5	26.4±22.1	10.0±13.3	7.20	0.0057	0.0551	2.73	0.1807	0.6120	2.64	0.0506	0.2515
EMILIN-2	EMIL2_HUMAN	Q9BXX0	EMILIN2	5.7	1053	12	12	64	116	4.0±6.7	6.4±6.8	0.8±1.7	1.60	0.4581	0.6709	0.20	0.2590	0.6770	8.00	0.0313	0.1914
Extracellular matrix protein 1	ECM1_HUMAN	Q16610	ECM1	7.41	540	5	5	34	61	0	4.8±5.2	1.0±2.2	N/A	N/A	N/A	N/A	N/A	N/A	4.80	0.0727	0.3041
Extracellular superoxide dismutase [Cu-Zn]	SODE_HUMAN	P08294	SOD3	44.2	240	13	13	282	26	9±2.1	23.2±15.5	11.2±4.9	2.58	0.0916	0.2730	1.24	0.5091	0.8399	2.07	0.1337	0.4261
Fibrillin-1	FBN1_HUMAN	P35555	FBN1	10.2	2871	43	41	1768	312	53.0±23.8	154.4±105.8	67.8±21.9	2.91	0.0784	0.2584	1.28	0.2005	0.6320	2.28	0.1118	0.3950
Fibromodulin	FMOD_HUMAN	Q06828	FMOD	27.1	376	9	9	216	43	2.3±3.7	28.4±26.2	6.0±6.4	12.17	0.0293	0.1282	2.57	0.3476	0.7194	4.73	0.0911	0.3578
Fibronectin	FINC_HUMAN	P02751	FN1	34.7	2477	75	75	6666	272	243.0±95.4	417.6±114.5	312.0±118.4	1.72	0.0301	0.1282	1.28	0.2019	0.6320	1.34	0.1266	0.4261
Fibulin-1	FBLN1_HUMAN	P23142	FBLN1	4.69	703	2	2	4	77	0	0.8±1.8	0	N/A	N/A	N/A	N/A	N/A	N/A	N/A	N/A	N/A
Fibulin-2	FBLN2_HUMAN	P98095	FBLN2	5.07	1184	10	10	44	127	0	7.2±7.0	0.8±2.5	N/A	N/A	N/A	N/A	N/A	N/A	9.00	0.0220	0.1644
Fibulin-5	FBLN5_HUMAN	Q9UBX5	FBLN5	7.14	448	4	4	28	50	0	3.6±3.6	1.0±2.2	N/A	N/A	N/A	N/A	N/A	N/A	3.60	0.0990	0.3578
Galectin-1	LEG1_HUMAN	P09382	LGALS1	82.2	135	17	17	1130	15	48.3±10.1	61.2±15.6	53.4±14.7	1.27	0.1540	0.3989	1.10	0.6178	0.8938	1.15	0.3729	0.7375
Galectin-3	LEG3_HUMAN	P17931	LGALS3	32.8	250	8	8	1012	26	46.3±12.3	45.2±12.3	51.0±16.0	0.98	0.8990	0.9446	1.10	0.4707	0.8169	0.89	0.4170	0.7375
Galectin-3-binding protein	LG3BP_HUMAN	Q08380	LGALS3BP	39.7	585	24	24	2320	65	118.0±38.3	110.0±38.4	106.2±52.6	0.93	0.8347	0.9446	0.90	0.5630	0.8786	1.04	0.7104	0.8998
Gelsolin	GELS_HUMAN	P06396	GSN	41.7	782	56	56	7340	86	350.3±28.6	374.4±65.5	336.6±27.1	1.07	0.6059	0.7684	0.96	0.3585	0.7304	1.11	0.2567	0.6091
Glia-derived nexin	GDN_HUMAN	P07093	SERPINE2	13.8	398	7	7	26	44	0	2.4±3.6	1.4±3.3	N/A	N/A	N/A	N/A	N/A	N/A	1.71	0.4883	0.7842
Hepatoma-derived growth factor	HDGF_HUMAN	P51858	HDGF	30.4	240	9	8	84	27	5.0±3.0	4.4±3.0	3.2±4.0	0.88	0.7878	0.9138	0.64	0.2323	0.6657	1.38	0.3712	0.7375
Hepatoma-derived growth factor-related protein 3	HDGR3_HUMAN	Q9Y3E1	HDGFL3	13.8	203	4	4	36	23	2.0±3.3	0.8±1.8	2.0±2.8	0.40	0.5291	0.7306	1.00	0.9173	0.9765	0.40	0.4093	0.7375
Hornerin	HORN_HUMAN	Q86YZ3	HRNR	3.79	2850	6	6	28	282	4.0±9.8	0.8±1.8	0	0.20	0.6833	0.8326	N/A	N/A	N/A	N/A	N/A	N/A
Hyaluronan and proteoglycan link protein 1	HPLN1_HUMAN	P10915	HAPLN1	38.1	354	15	13	392	40	4.7±6.0	28.4±35.8	22.2±36.2	6.09	0.1806	0.4515	4.76	0.2267	0.6657	1.28	0.7713	0.9139
Hyaluronan and proteoglycan link protein 3	HPLN3_HUMAN	Q96S86	HAPLN3	15.6	360	5	3	12	41	0	0.8±1.8	0.8±2.5	N/A	N/A	N/A	N/A	N/A	N/A	1.00	0.8427	0.9376
Hyaluronan-binding protein 2	HABP2_HUMAN	Q14520	HABP2	9.82	560	6	6	36	63	0.7±1.6	3.6±4.98	1.4±2.32	5.40	0.2418	0.5178	2.10	0.5263	0.8399	2.57	0.3956	0.7375
Immunoglobulin superfamily containing leucine-rich repeat p	ISLR_HUMAN	O14498	ISLR	11.9	428	4	4	8	46	0	1.6±3.6	0	N/A	N/A	N/A	N/A	N/A	N/A	N/A	N/A	N/A
Insulin-like growth factor-binding protein 2	IBP2_HUMAN	P18065	IGFBP2	7.69	325	3	3	16	35	0	3.2±1.79	0	N/A	N/A	N/A	N/A	N/A	N/A	N/A	N/A	N/A
Insulin-like growth factor-binding protein 7	IBP7_HUMAN	Q16270	IGFBP7	43.3	282	12	12	336	29	10.7±5.3	24.4±6.5	15.0±4.8	2.29	0.0054	0.0551	1.41	0.0745	0.4440	1.63	0.0113	0.1286
Integrin alpha-1	ITA1_HUMAN	P56199	ITGA1	1.87	1179	2	2	4	131	0.6±1.6	0	0	N/A	N/A	N/A	N/A	N/A	N/A	N/A	N/A	N/A
Integrin alpha-V	ITAV_HUMAN	P06756	ITGAV	10.3	1048	15	15	240	116	14.7±6.9	9.2±5.8	10.6±7.5	0.63	0.2464	0.5178	0.72	0.2688	0.6904	0.87	0.8433	0.9376
Integrin beta-1	ITB1_HUMAN	P05556	ITGB1	8.65	798	11	11	192	88	9.3±8.0	8.4±5.9	9.4±6.0	0.90	0.8943	0.9446	1.01	0.7536	0.9391	0.89	0.8691	0.9400
Inter-alpha-trypsin inhibitor heavy chain H1	ITIH1_HUMAN	P19827	ITIH1	11.2	911	13	13	324	101	8.7±7.8	24.0±25.0	15.2±14.2	2.77	0.3374	0.5763	1.75	0.4879	0.8169	1.58	0.6464	0.8637
Inter-alpha-trypsin inhibitor heavy chain H2	ITIH2_HUMAN	P19823	ITIH2	8.67	946	7	7	358	106	16.7±7.0	12.4±7.4	19.6±10.3	0.74	0.2614	0.5264	1.18	0.8249	0.9391	0.63	0.2252	0.5509
Inter-alpha-trypsin inhibitor heavy chain H4	ITIH4_HUMAN	Q14624	ITIH4	6.13	930	8	8	70	103	0.7±1.6	4.8±7.8	4.2±6.1	7.20	0.2399	0.5178	6.30	0.2078	0.6320	1.14	0.9718	0.9921
Intercellular adhesion molecule 1	ICAM1_HUMAN	P05362	ICAM1	10.3	532	6	6	42	58	2.0±3.1	2.4±3.6	1.8±3.0	1.20	0.8636	0.9446	0.90	0.8755	0.9467	1.33	0.7294	0.8998
Kallistatin	KAIN_HUMAN	P29622	SERPINA4	11.7	427	4	4	22	49	0.67±1.6	0.8±1.8	1.4±2.3	1.20	0.8957	0.9446	2.10	0.5263	0.8399	0.57	0.6397	0.8619
Kininogen-1	KNG1_HUMAN	P01042	KNG1	17.5	644	13	13	336	72	11.0±5.2	22.4±9.7	15.8±5.0	2.04	0.0409	0.1602	1.44	0.0645	0.4440	1.42	0.2166	0.5412
Lactadherin	MFGM_HUMAN	Q08431	MFGE8	50.6	387	20	20	728	43	27.3±21.4	40.4±30.0	36.2±31.5	1.48	0.5772	0.7540	1.32	0.3774	0.7357	1.12	0.9797	0.9921
Laminin subunit alpha-2	LAMA2_HUMAN	P24043	LAMA2	23.4	3122	104	103	4010	344	204.7±37.6	162.4±86.7	197.0±35.4	0.79	0.2187	0.4879	0.96	0.6539	0.9192	0.82	0.1579	0.4482
Laminin subunit alpha-4	LAMA4_HUMAN	Q16363	LAMA4	20.4	1823	43	43	1558	203	61.7±12.1	80.0±46.8	78.8±29.6	1.30	0.9768	0.9842	1.28	0.2994	0.6918	1.02	0.6067	0.8493
Laminin subunit alpha-5	LAMA5_HUMAN	O15230	LAMA5	12.4	3695	60	59	2536	400	149.3±46.6	131.2±69.9	98.4±42.1	0.88	0.4043	0.6304	0.66	0.0525	0.4440	1.33	0.7632	0.9139
Laminin subunit beta-1	LAMB1_HUMAN	P07942	LAMB1	25.9	1786	61	61	1956	198	97.0±19.5	65.6±38.7	104.6±31.7	0.68	0.1069	0.3083	1.08	0.6703	0.9318	0.63	0.0341	0.2006
Laminin subunit beta-2	LAMB2_HUMAN	P55268	LAMB2	28.1	1798	68	68	4030	196	203.0±40.6	198.4±96.7	182.0±45.8	0.98	0.5439	0.7350	0.90	0.3137	0.6918	1.09	0.7300	0.8998
Laminin subunit gamma-1	LAMC1_HUMAN	P11047	LAMC1	37.4	1609	79	78	5268	178	257.0±27.2	232.0±101.7	256.6±57.5	0.90	0.4043	0.6304	1.00	0.8490	0.9391	0.90	0.3558	0.7347
Latent-transforming growth factor beta-binding protein 2	LTBP2_HUMAN	Q14767	LTBP2	17.3	1821	34	34	634	195	3.3±6.4	80.8±52.3	21.0±22.2	24.24	0.0001	0.0075	6.30	0.0724	0.4440	3.85	0.0204	0.1644
Latent-transforming growth factor beta-binding protein 4	LTBP4_HUMAN	Q8N2S1	LTBP4	13.1	1624	22	22	232	173	1.7±4.1	22.4±17.0	11.0±9.6	13.44	0.0112	0.0696	6.60	0.0124	0.2149	2.04	0.3908	0.7375
Leucine-rich alpha-2-glycoprotein	A2GL_HUMAN	P02750	LRG1	11.8	347	5	5	28	38	3.3±2.7	0	0.8±1.7	N/A	N/A	N/A	0.24	0.0406	0.4036	N/A	N/A	N/A
Leukocyte elastase inhibitor	ILEU_HUMAN	P30740	SERPINB1	15	379	7	6	232	43	9.7±3.8	12±6.8	11.4±6.6	1.24	0.6692	0.8224	1.18	0.9938	0.9984	1.05	0.7568	0.9139
Lipoprotein lipase	LIPL_HUMAN	P06858	LPL	7.58	475	2	2	18	53	1.7±4.1	0	0.8±1.7	N/A	N/A	N/A	0.48	0.7779	0.9391	N/A	N/A	N/A
Lumican	LUM_HUMAN	P51884	LUM	57.1	338	35	35	5356	38	193.3±53.4	404.0±95.6	217.6±51.6	2.09	0.0016	0.0352	1.13	0.3627	0.7304	1.86	0.0008	0.0245
Lysozyme C	LYSC_HUMAN	P61626	LYZ	57.4	148	8	8	196	17	4.3±3.4	14.4±8.2	9.8±4.8	3.32	0.0323	0.1338	2.26	0.0725	0.4440	1.47	0.3723	0.7375
Macrophage migration inhibitory factor	MIF_HUMAN	P14174	MIF	17.4	115	4	4	26	12	1.3±2.1	0	1.8±2.4	N/A	N/A	N/A	1.35	0.7334	0.9391	N/A	N/A	N/A
Mast cell carboxypeptidase A	CBPA3_HUMAN	P15088	CPA3	10.1	417	5	5	68	49	2.0±3.3	3.2±3.3	4.0±4.7	1.60	0.4755	0.6894	2.00	0.4301	0.7911	0.80	0.9516	0.9889
Matrix Gla protein	MGP_HUMAN	P08493	MGP	32	103	3	3	64	12	3.0±2.4	5.6±3.6	1.8±2.4	1.87	0.2942	0.5539	0.60	0.3316	0.7059	3.11	0.0489	0.2510
Matrix-remodeling-associated protein 5	MXRA5_HUMAN	Q9NR99	MXRA5	1.38	2828	2	2	4	312	0	0.8±1.8	0	N/A	N/A	N/A	N/A	N/A	N/A	N/A	N/A	N/A
Matrix-remodeling-associated protein 7	MXRA7_HUMAN	P84157	MXRA7	19.6	204	2	2	12	21	0	1.6±2.2	0.4±1.3	N/A	N/A	N/A	N/A	N/A	N/A	4.00	0.1828	0.4765
Mesencephalic astrocyte-derived neurotrophic factor	MANF_HUMAN	P55145	MANF	44	182	8	8	100	21	4.3±5.4	3.6±3.6	5.6±6.9	0.83	0.9722	0.9842	1.29	0.8307	0.9391	0.64	0.8577	0.9400
Metalloproteinase inhibitor 1	TIMP1_HUMAN	P01033	TIMP1	15	207	2	2	4	23	0	0.8±1.8	0	N/A	N/A	N/A	N/A	N/A	N/A	N/A	N/A	N/A
Microfibril-associated glycoprotein 4	MFAP4_HUMAN	P55083	MFAP4	20.4	255	4	4	112	29	0	16.4±19.7	3.0±6.3	N/A	N/A	N/A	N/A	N/A	N/A	5.47	0.0802	0.3268
Microfibrillar-associated protein 5	MFAP5_HUMAN	Q13361	MFAP5	27.2	173	5	5	430	20	17.67±6.86	21.6±1.67	21.6±8.2	1.22	0.1876	0.4611	1.22	0.3697	0.7344	1.00	0.7166	0.8998
Mimecan	MIME_HUMAN	P20774	OGN	54.7	298	27	27	3710	34	99.3±38.8	386.0±191.2	118.4±43.3	3.89	0.0017	0.0352	1.19	0.4090	0.7618	3.26	0.0005	0.0245
Monocyte differentiation antigen CD14	CD14_HUMAN	P08571	CD14	9.87	375	2	2	4	40	0	0	0.4±1.3	N/A	N/A	N/A	N/A	N/A	N/A	N/A	N/A	N/A
Multimerin-2	MMRN2_HUMAN	Q9H8L6	MMRN2	7.48	949	10	10	260	104	13.3±6.4	10.0±6.2	13.0±6.6	0.75	0.3915	0.6239	0.98	0.9420	0.9811	0.77	0.3130	0.6877
Neutrophil defensin 1	DEF1_HUMAN	P59665	DEFA1							0.7±1.6	0	0.4±1.3	N/A	N/A	N/A	0.60	0.7139	0.9349	N/A	N/A	N/A
Nidogen-1	NID1_HUMAN	P14543	NID1	35.6	1247	55	54	5074	136	232.7±60.2	232.8±25.2	251.4±53.1	1.00	0.8734	0.9446	1.08	0.4479	0.8040	0.93	0.5616	0.8193
Nidogen-2	NID2_HUMAN	Q14112	NID2	35.8	1375	58	57	6294	151	336.0±34.2	289.2±119.2	283.2±63.2	0.86	0.2985	0.5548	0.84	0.0636	0.4440	1.02	0.7871	0.9202
Nucleobindin-1	NUCB1_HUMAN	Q02818	NUCB1	15.6	461	8	8	80	54	2.7±3.0	3.2±1.8	4.8±3.9	1.20	0.5553	0.7386	1.80	0.2401	0.6751	0.67	0.5466	0.8047
Olfactomedin-like protein 1	OLFL1_HUMAN	Q6UWY5	OLFML1	22.4	402	6	6	24	46	0	4.8±10.7	0	N/A	N/A	N/A	N/A	N/A	N/A	N/A	N/A	N/A
Olfactomedin-like protein 3	OLFL3_HUMAN	Q9NRN5	OLFML3	8.87	406	7	7	16	46	0	3.2±4.6	0	N/A	N/A	N/A	N/A	N/A	N/A	N/A	N/A	N/A
Palmitoyl-protein thioesterase 1	PPT1_HUMAN	P50897	PPT1	33	306	11	11	270	34	16.0±7.2	10.0±6.8	12.4±8.8	0.63	0.1957	0.4688	0.78	0.2982	0.6918	0.81	0.8319	0.9376

Papilin	PPN_HUMAN	O95428	PAPLN	6.96	1278	7	7	32	138	0	5.6±3.6	0.4±1.3	N/A	N/A	N/A	N/A	N/A	N/A	14.00	0.0007	0.0245
Pentraxin-related protein PTX3	PTX3_HUMAN	P26022	PTX3	7.61	381	3	3	20	42	0	0	2.0±5.1	N/A	N/A	N/A	N/A	N/A	N/A	N/A	N/A	N/A
Perilipin-2	PLIN2_HUMAN	Q99541	PLIN2	14	437	4	4	8	48	1.33±3.27	0	0	N/A	N/A	N/A	N/A	N/A	N/A	N/A	N/A	N/A
Periostin	POSTN_HUMAN	Q15063	POSTN	53	836	56	55	3330	93	40.3±33.5	340.0±280.9	138.8±131.0	8.43	0.0203	0.0950	3.44	0.1948	0.6320	2.45	0.1425	0.4276
Peroxiredoxin-1	PRDX1_HUMAN	Q06830	PRDX1	62.8	199	19	16	622	22	21.7±9.4	28.4±12.4	35±11.2	1.31	0.4335	0.6548	1.62	0.0121	0.2149	0.81	0.2179	0.5412
Peroxiredoxin-2	PRDX2_HUMAN	P32119	PRDX2	53	198	14	13	1480	22	67.0±23.0	61.6±9.0	77.0±17.7	0.92	0.8515	0.9446	1.15	0.2538	0.6770	0.80	0.0972	0.3578
Peroxiredoxin-6	PRDX6_HUMAN	P30041	PRDX6	60.3	224	11	11	250	25	17.0±12.8	6.0±5.3	11.8±7.5	0.35	0.0751	0.2531	0.69	0.4595	0.8151	0.51	0.1912	0.4903
Phospholipase A2, membrane associated	PA2GA_HUMAN	P14555	PLA2G2A	42.4	144	7	7	52	16	3.0±5.6	6.8±7.2	0	2.27	0.3114	0.5574	N/A	N/A	N/A	N/A	N/A	N/A
Pigment epithelium-derived factor	PEDF_HUMAN	P36955	SERPINF1	17.9	418	10	10	76	46	1.7±2.7	4.8±5.2	4.2±4.4	2.88	0.2693	0.5349	2.52	0.2462	0.6770	1.14	0.8940	0.9484
Plasma protease C1 inhibitor	IC1_HUMAN	P05155	SERPING1	5.4	500	2	2	8	55	0	0	0.8±1.7	N/A	N/A	N/A	N/A	N/A	N/A	N/A	N/A	N/A
Plasma serine protease inhibitor	IPSP_HUMAN	P05154	SERPINA5	10.3	406	5	5	24	46	0	2.4±3.3	1.2±1.9	N/A	N/A	N/A	N/A	N/A	N/A	2.00	0.4767	0.7814
Platelet glycoprotein 4	CD36_HUMAN	P16671	CD36	14.2	472	7	7	268	53	13.0±9.4	9.2±11.4	14.4±12.2	0.71	0.2436	0.5178	1.11	0.8491	0.9391	0.64	0.1242	0.4261
Pleiotrophin	PTN_HUMAN	P21246	PTN	36.3	168	5	5	72	19	0.7±1.6	11.6±8.6	1.0±2.2	17.40	0.0057	0.0551	1.50	0.7926	0.9391	11.60	0.0020	0.0466
Plexin-B2	PLXB2_HUMAN	O15031	PLXNB2	5.22	1838	12	12	118	205	5.7±4.8	5.6±4.3	5.6±4.7	0.99	0.8882	0.9446	0.99	0.9984	0.9984	1.00	0.8750	0.9400
Podocan	PODN_HUMAN	Q7Z5L7	PODN	4.73	613	3	3	12	69	0	0.8±1.8	0.8±1.7	N/A	N/A	N/A	N/A	N/A	N/A	1.00	1.0000	1.0000
Prenylcysteine oxidase 1	PCYOX_HUMAN	Q9UHG3	PCYOX1	6.53	505	2	2	4	57	0.7±1.6	0	0	N/A	N/A	N/A	N/A	N/A	N/A	N/A	N/A	N/A
Procollagen C-endopeptidase enhancer 1	PCOC1_HUMAN	Q15113	PCOLCE	24.7	449	8	8	20	48	0	4.0±6.2	0	N/A	N/A	N/A	N/A	N/A	N/A	N/A	N/A	N/A
Progranulin	GRN_HUMAN	P28799	GRN	5.9	593	3	3	6	64	0	1.2±2.7	0	N/A	N/A	N/A	N/A	N/A	N/A	N/A	N/A	N/A
Prolargin	PRELP_HUMAN	P51888	PRELP	57.9	382	33	33	5428	44	198.7±89.9	446.4±166.6	200.4±56.4	2.25	0.0064	0.0583	1.01	0.8201	0.9391	2.23	0.0009	0.0245
Prolow-density lipoprotein receptor-related protein 1	LRP1_HUMAN	Q07954	LRP1	1.47	4544	9	9	52	505	1.3±2.1	5.6±7.4	1.6±3.9	4.20	0.2102	0.4775	1.20	0.8574	0.9393	3.50	0.1344	0.4261
Prosaposin	SAP_HUMAN	P07602	PSAP	13.2	524	12	12	242	58	11.3±4.1	13.2±2.7	10.8±6.0	1.16	0.3405	0.5763	0.95	0.5965	0.8863	1.22	0.3201	0.6877
Prostaglandin-H2 D-isomerase	PTGDS_HUMAN	P41222	PTGDS	21.6	190	4	4	124	21	4.3±3.9	8.4±6.1	5.6±2.5	1.94	0.3217	0.5689	1.29	0.3157	0.6918	1.50	0.6280	0.8607
Protein AMBP	AMBP_HUMAN	P02760	AMBP	30.1	352	13	13	398	39	9.3±3.0	43.2±30.3	12.6±12.5	4.63	0.0017	0.0352	1.35	0.8086	0.9391	3.43	0.0142	0.1396
Protein disulfide-isomerase	PDIA1_HUMAN	P07237	P4HB	39.4	508	26	26	622	57	34.0±11.4	28.8±5.8	27.4±10.3	0.85	0.5214	0.7269	0.81	0.2800	0.6918	1.05	0.6089	0.8493
Protein S100-A10	S10AA_HUMAN	P60903	S100A10	32	97	3	3	28	11	0.7±1.6	1.2±2.7	1.8±2.4	1.80	0.7530	0.8877	2.70	0.3275	0.7059	0.67	0.5769	0.8263
Protein S100-A11	S10AB_HUMAN	P31949	S100A11	37.1	105	6	6	26	12	1.3±3.3	2±2.8	0.8±1.7	1.50	0.5726	0.7540	0.60	0.8457	0.9391	2.50	0.3431	0.7179
Protein S100-A13	S10AD_HUMAN	Q99584	S100A13	27.6	98	4	4	8	11	0.7±1.6	0	0.4±1.3	N/A	N/A	N/A	0.60	0.7139	0.9349	N/A	N/A	N/A
Protein S100-A4	S10A4_HUMAN	P26447	S100A4	17.8	101	3	3	12	12	0	2.4±2.2	0	N/A	N/A	N/A	N/A	N/A	N/A	N/A	N/A	N/A
Protein S100-A8	S10A8_HUMAN	P05109	S100A8	23.7	93	2	2	8	11	0.7±1.6	0.8±1.8	0	1.20	0.8957	0.9446	N/A	N/A	N/A	N/A	N/A	N/A
Protein S100-A9	S10A9_HUMAN	P06702	S100A9	35.1	114	5	5	56	13	2.3±3.7	3.2±3.0	2.6±4.4	1.37	0.5431	0.7350	1.11	0.9855	0.9984	1.23	0.5081	0.7844
Protein-lysine 6-oxidase	LYOX_HUMAN	P28300	LOX	15.1	417	4	4	24	47	0	4.8±5.0	0	N/A	N/A	N/A	N/A	N/A	N/A	N/A	N/A	N/A
Secreted frizzled-related protein 1	SFRP1_HUMAN	Q8N474	SFRP1	16.6	314	6	6	102	35	5.3±4.7	6.4±3.8	3.8±3.7	1.20	0.6147	0.7684	0.71	0.6048	0.8863	1.68	0.2712	0.6249
Serine protease HTRA1	HTRA1_HUMAN	Q92743	HTRA1	9.79	480	5	5	12	51	0	2.4±3.6	0	N/A	N/A	N/A	N/A	N/A	N/A	N/A	N/A	N/A
Serpin B6	SPB6_HUMAN	P35237	SERPINB6	30.3	376	13	12	256	43	11.0±8.8	10.8±7.6	13.6±9.3	0.98	0.9953	0.9953	1.24	0.5299	0.8399	0.79	0.5458	0.8047
Serpin H1	SERPH_HUMAN	P50454	SERPINH1	33	418	14	14	484	46	19.0±6.8	21.6±2.6	26.2±9.9	1.14	0.3508	0.5780	1.38	0.1342	0.4953	0.82	0.4828	0.7834
Serum amyloid A-1 protein	SAA1_HUMAN	P0DJI8	SAA1	66.4	122	11	6	152	14	16.67±9.69	2.0±4.5	4.2±6.7	0.12	0.0017	0.0352	0.25	0.0057	0.2149	0.48	0.5012	0.7844
Serum amyloid A-2 protein	SAA2_HUMAN	P0DJI9	SAA2	60.7	122	10	5	112	14	11.3±5.7	3.2±4.6	2.8±4.9	0.28	0.0131	0.0712	0.25	0.0030	0.2149	1.14	0.7738	0.9139
Serum amyloid P-component	SAMP_HUMAN	P02743	APCS	37.7	223	13	13	1588	25	86.0±16.7	98.0±38.0	58.2±18.0	1.14	0.7252	0.8691	0.68	0.0140	0.2149	1.68	0.0300	0.1910
Signal recognition particle 14 kDa protein	SRP14_HUMAN	P37108	SRP14	48.5	136	6	6	38	15	1.3±2.1	0	3.0±2.9	N/A	N/A	N/A	2.25	0.2547	0.6770	N/A	N/A	N/A
Somatomedin-B and thrombospondin type-1 domain-contair	SBSPO_HUMAN	Q8IVN8	SBSPON	51.9	264	9	9	196	30	7.7±5.00	12.8±4.8	8.6±6.5	1.67	0.1525	0.3989	1.12	0.8047	0.9391	1.49	0.1648	0.4598
SPARC-like protein 1	SPRL1_HUMAN	Q14515	SPARCL1	4.07	664	2	2	4	75	0.7±1.6	0	0	N/A	N/A	N/A	N/A	N/A	N/A	N/A	N/A	N/A
Spondin-1	SPON1_HUMAN	Q9HCB6	SPON1	6.94	807	6	6	118	91	3.3±4.1	6.0±4.5	6.8±6.0	1.80	0.2942	0.5539	2.04	0.2812	0.6918	0.88	0.9763	0.9921
Sushi repeat-containing protein SRPX2	SRPX2_HUMAN	O60687	SRPX2	7.74	465	2	2	4	53	0	0.8±1.8	0	N/A	N/A	N/A	N/A	N/A	N/A	N/A	N/A	N/A
Target of Nesh-SH3	TARSH_HUMAN	Q7Z7G0	ABI3BP	21.7	1075	25	25	1684	119	32.0±34.1	198.4±93.3	50.0±51.5	6.20	0.0089	0.0675	1.56	0.5175	0.8399	3.97	0.0158	0.1396
Tenascin	TENA_HUMAN	P24821	TNC	22.7	2201	43	43	374	241	2.7±4.8	43.6±77.8	14.0±18.3	16.35	0.0693	0.2452	5.25	0.1032	0.4953	3.11	0.5212	0.7969
Tenascin-X	TENX_HUMAN	P22105	TNXB	24.4	4244	153	153	5830	458	206.0±84.9	377.2±196.1	270.8±79.7	1.83	0.0692	0.2452	1.31	0.1197	0.4953	1.39	0.1820	0.4765
Tetranectin	TETN_HUMAN	P05452	CLEC3B	30.2	202	9	9	100	23	2.3±2.7	7.2±1.8	5.0±3.3	3.09	0.0115	0.0696	2.14	0.1363	0.4953	1.44	0.1810	0.4765
Thioredoxin	THIO_HUMAN	P10599	TXN	31.4	105	5	5	70	12	3.3±3.7	4.0±2.4	3.0±2.7	1.20	0.5474	0.7350	0.90	0.9838	0.9984	1.33	0.4685	0.7760
Thrombospondin type-1 domain-containing protein 4	THSD4_HUMAN	Q6ZMP0	THSD4	7.76	1018	16	16	272	112	7.0±3.7	23.2±14.2	11.4±8.5	3.31	0.0133	0.0712	1.63	0.4375	0.7950	2.04	0.0613	0.2833
Thrombospondin-4	TSP4_HUMAN	P35443	THBS4	13.4	961	14	13	98	106	0	16.0±35.8	1.8±4.5	N/A	N/A	N/A	N/A	N/A	N/A	8.89	0.5050	0.7844
Transforming growth factor-beta-induced protein ig-h3	BGH3_HUMAN	Q15582	TGFBI	42.2	683	31	31	1932	75	65.3±41.3	118.4±69.0	94.8±45.7	1.81	0.2075	0.4775	1.45	0.1170	0.4953	1.25	0.7129	0.8998
Transgelin-2	TAGL2_HUMAN	P37802	TAGLN2	48.7	199	9	9	60	22	3.3±5.3	2.8±2.7	2.6±5.2	0.84	0.8049	0.9189	0.78	0.7662	0.9391	1.08	0.5426	0.8047
Tubulointerstitial nephritis antigen-like	TINAL_HUMAN	Q9GZM7	TINAGL1	55.7	467	25	25	4486	52	209.7±28.1	212.0±44.0	216.8±47.9	1.01	0.9774	0.9842	1.03	0.8400	0.9391	0.98	0.8947	0.9484
Versican core protein	CSPG2_HUMAN	P13611	VCAN	5.18	3396	26	26	2144	373	73.0±30.2	182.8±70.5	79.2±46.4	2.50	0.0081	0.0675	1.08	0.8396	0.9391	2.31	0.0227	0.1644
Vinculin	VINC_HUMAN	P18206	VCL	51.3	1134	100	100	7496	124	396.3±77.5	288.0±147.7	367.8±30.5	0.73	0.1622	0.4126	0.93	0.3896	0.7413	0.78	0.0988	0.3578
Vitronectin	VTNC_HUMAN	P04004	VTN	27.2	478	13	13	1538	54	75.3±8.5	96.0±23.1	60.6±13.5	1.27	0.1230	0.3429	0.80	0.0395	0.4036	1.58	0.0051	0.0805
von Willebrand factor	VWF_HUMAN	P04275	VWF	1.21	2813	3	3	8	309	0	1.6±2.2	0	N/A	N/A	N/A	N/A	N/A	N/A	N/A	N/A	N/A
von Willebrand factor A domain-containing protein 1	VWA1_HUMAN	Q6PCB0	VWA1	32.4	445	12	12	290	47	13.7±6.7	12.4±4.6	14.6±5.7	0.91	0.9598	0.9842	1.07	0.5823	0.8863	0.85	0.4585	0.7674
Y-box-binding protein 1	YBOX1_HUMAN	P67809	YBX1	42.3	324	18	12	776	36	29.3±5.0	36.8±9.6	41.6±9.7	1.25	0.1972	0.4688	1.42	0.0112	0.2149	0.88	0.4074	0.7375
Zinc-alpha-2-glycoprotein	ZA2G_HUMAN	P25311	AZGP1	24.2	298	8	8	48	34	1.0±2.4	3.6±6.1	2.4±3.4	3.60	0.3788	0.6103	2.40	0.3802	0.7357	1.50	0.8367	0.9376

P-values were derived from two-tailed Student's t-tests, and were only calculated when the protein was detected in the two groups being compared. N/A denotes "not applicable".

Supplemental Table II. Echocardiographic parameters measured at baseline and after ang II infusion in Adamts5$^{+/+}$ and Adamts5$^{\triangle Cat}$ mice.

	Baseline			Ang II			ANOVA p-values		
	**Adamts5$^{+/+}$**	**Adamts5$^{\Delta Cat}$**	**p-value**	**Adamts5$^{+/+}$**	**Adamts5$^{\Delta Cat}$**	**p-value**	**Ang II x Gen.**	**AngII**	**Genotype**
Heart rate (bpm)	464.7±35.3	457.4±24.9	>0.999	464.7±48.3	463.4±59.5	>0.999	0.779	0.779	0.802
LVEF (%)	47.8±3.8	49.1±4.5	>0.999	54.3±4.9	48.2±3.2	**0.006**	**0.005**	**0.027**	0.134
GLS (%)	-18.1±4.0	-18.8±3.6	>0.999	-18.8±3.6	-14.1±2.4	**0.013**	**0.024**	0.086	0.116
LV mass (mg)	96.6±12.7	99.8±9.1	>0.999	120.2±14.9	112.4±9.1	0.407	**0.048**	**<0.001**	0.696
LVESV (μL)	37.0±6.3	33.1±7.5	0.455	29.7±7.1	33.6±6.3	0.454	**0.031**	0.051	0.999
LVEDV (μL)	70.8±10.5	64.5±10.9	0.428	64.7±11.5	64.6±10.7	>0.999	0.187	0.210	0.483
Reverse LSR (1/s)	6.5±2.0	5.9±1.7	>0.999	6.2±2.0	5.3±1.9	0.599	0.764	0.454	0.284
LA size (mm^2)*	4.4±0.5	4.3±0.5	>0.999	4.7±0.4	4.1±0.7	0.081	0.080	0.612	0.186

Values are shown as averages ± standard deviation. Adamts5$^{+/+}$ (n=12) and Adamts5$^{\triangle Cat}$ mice (n=8) were subject to echocardiography imaging at baseline and after ang II infusion for 2 weeks. P-values were derived from two-way ANOVAs with Bonferroni corrections. LVEF, left ventricular ejection fraction; GLS, global longitudinal strain; LV, left ventricle; LVESV, left ventricular end-systolic volume; LVEDV, left ventricular end-diastolic volume; LSR, longitudinal strain rate. *For LA size, data are based on 8 Adamts5$^{+/+}$ and 6 Adamts5$^{\triangle Cat}$ mice.

Supplemental Table III. Proteomics analysis of cardiac ECM in Adamts5$^{+/+}$ and Adamts5$^{\Delta Cat}$ after Ang II infusion for 2 weeks.

Protein information										Abundance (Average±SD)		Adamts5$^{+/+}$ vs Adamts5$^{\Delta Cat}$		
Protein Name	Accesion number	Uniprot ID	Gene name	Coverage [%]	# AAs	# Peptides	# Unique Peptides	Total Spectrum Count	MW [kDa]	Adamts5$^{+/+}$ (Ang II)	Adamts5$^{\Delta Cat}$ (Ang II)	FC	P.Value	BH Adjusted P-value
Adiponectin	ADIPO_MOUSE	Q60994	Adipoq	20	247	5	5	37	26.8	23.24±0.22	22.66±0.70	0.67	0.0669	0.4875
Agrin	AGRIN_MOUSE	A2ASQ1	Agrn	30	1950	30	30	168	207.4	25.36±0.21	25.36±0.31	1.00	0.9860	0.9860
Annexin A2	ANXA2_MOUSE	P07356	Anxa2	37	339	12	12	63	38.7	24.08±0.31	24.21±0.14	1.09	0.4741	0.7375
Apolipoprotein A-I	APOA1_MOUSE	Q00623	Apoa1	55	264	19	19	149	30.6	26.48±0.42	27.10±0.55	1.54	0.0522	0.4875
Apolipoprotein A-IV	APOA4_MOUSE	P06728	Apoa4	8	395	2	2	4	45	20.40±0.52	20.30±0.87	0.93	0.8077	0.8845
Apolipoprotein C-I	APOC1_MOUSE	P34928	Apoc1	10	88	1	1	3	9.7	20.34±0.82	20.92±0.36	1.50	0.1772	0.5726
Apolipoprotein C-III	APOC3_MOUSE	P33622	Apoc3	27	99	2	2	37	11	22.81±0.66	22.98±0.39	1.12	0.6470	0.8361
Apolipoprotein E	APOE_MOUSE	P08226	Apoe	45	311	12	12	49	35.8	23.64±0.31	23.84±0.42	1.15	0.3869	0.6827
Asporin	ASPN_MOUSE	Q99MQ4	Aspn	23	373	6	6	24	42.5	22.84±0.22	22.69±0.23	0.90	0.3577	0.6827
Basal cell adhesion molecule	BCAM_MOUSE	Q9R069	Bcam	28	622	12	12	32	67.6	23.10±0.54	23.27±0.62	1.13	0.6258	0.8361
Basement membrane-specific heparan sulfate proteoglycan	PGBM_MOUSE	Q05793	Hspg2	52	3707	135	135	1666	398	29.90±0.14	29.69±0.08	0.87	0.0901	0.4875
Biglycan	PGS1_MOUSE	P28653	Bgn	47	369	12	12	100	41.6	24.74±0.32	24.91±0.44	1.13	0.4740	0.7375
Cadherin-13	CAD13_MOUSE	Q9WTR5	Cdh13	4	714	2	2	6	78.1	22.23±0.07	22.01±0.25	0.86	0.1178	0.5392
Cadherin-2	CADH2_MOUSE	P15116	Cdh2	13	906	7	7	31	99.7	22.66±0.24	22.78±0.21	1.09	0.4606	0.7375
Cadherin-5	CADH5_MOUSE	P55284	Cdh5	4	784	2	2	8	87.8	19.63±0.77	19.29±0.65	0.79	0.4480	0.7375
Cathepsin B	CATB_MOUSE	P10605	Ctsb	9	339	3	3	14	37.3	21.88±0.41	21.92±0.23	1.03	0.8372	0.8901
Cathepsin D	CATD_MOUSE	P18242	Ctsd	49	410	14	14	141	44.9	26.38±0.25	26.23±0.10	0.90	0.3317	0.6827
Caveolae-associated protein 1	CAVN1_MOUSE	O54724	Cavin1	38	392	22	22	528	43.9	29.17±0.06	29.00±0.05	0.89	0.0929	0.4875
Chymase	CMA1_MOUSE	P21844	Cma1	12	247	2	2	13	27.6	21.70±0.31	22.13±0.40	1.35	0.0726	0.4875
Clusterin	CLUS_MOUSE	Q06890	Clu	21	448	6	6	36	51.6	23.42±0.31	23.85±0.16	1.35	0.0325	0.3901
Collagen alpha-1(I) chain	CO1A1_MOUSE	P11087	Col1a1	56	1453	54	53	944	137.9	27.50±0.62	27.45±0.91	0.97	0.9188	0.9412
Collagen alpha-1(III) chain	CO3A1_MOUSE	P08121	Col3a1	19	1464	12	12	131	138.9	24.43±0.34	24.61±1.18	1.13	0.7110	0.8582
Collagen alpha-1(IV) chain	CO4A1_MOUSE	P02463	Col4a1	4	1669	6	6	24	160.6	23.26±0.21	23.46±0.37	1.14	0.3112	0.6827
Collagen alpha-1(V) chain	CO5A1_MOUSE	O88207	Col5a1	4	1838	4	4	37	183.6	21.54±0.46	21.07±0.58	0.72	0.1529	0.5584
Collagen alpha-1(VI) chain	CO6A1_MOUSE	Q04857	Col6a1	26	1025	22	22	180	108.4	26.37±0.15	25.76±0.16	0.65	0.0006	0.0238
Collagen alpha-1(XV) chain	COFA1_MOUSE	O35206	Col15a1	14	1367	15	15	186	140.4	26.01±0.23	25.70±0.12	0.81	0.0535	0.4875
Collagen alpha-1(XVIII) chain	COIA1_MOUSE	P39061	Col18a1	4	1774	5	5	26	182.1	23.27±0.37	23.53±0.28	1.20	0.2409	0.6528
Collagen alpha-2(I) chain	CO1A2_MOUSE	Q01149	Col1a2	48	1372	31	31	453	129.5	31.03±0.21	31.00±0.21	0.98	0.8213	0.8845
Collagen alpha-2(IV) chain	CO4A2_MOUSE	P08122	Col4a2	8	1707	7	7	30	167.2	22.42±0.05	22.58±0.43	1.12	0.3667	0.6827
Collagen alpha-2(V) chain	CO5A2_MOUSE	Q3U962	Col5a2	13	1497	9	9	61	144.9	23.45±0.51	23.33±0.84	0.92	0.7604	0.8699
Collagen alpha-2(VI) chain	CO6A2_MOUSE	Q02788	Col6a2	38	1034	24	24	182	110.3	26.10±0.26	25.52±0.20	0.67	0.0050	0.1060
Collagen alpha-6(VI) chain	CO6A6_MOUSE	Q8C6K9	Col6a6	7	2265	9	9	25	246.2	23.3±0.55	22.89±0.28	0.75	0.1737	0.5726
Decorin	PGS2_MOUSE	P28654	Dcn	50	354	16	16	196	39.8	26.47±0.20	26.21±0.42	0.83	0.2070	0.6389
Dermatopontin	DERM_MOUSE	Q9QZZ6	Dpt	27	201	5	5	56	24	24.55±0.23	24.40±0.25	0.90	0.3492	0.6827
Desmoplakin	DESP_MOUSE	E9Q557	Dsp	4	2883	8	8	15	332.7	21.45±0.61	21.00±0.74	0.73	0.2703	0.6827
Dystroglycan	DAG1_MOUSE	Q62165	Dag1	7	893	5	5	30	96.8	24.38±0.14	24.22±0.19	0.89	0.2278	0.6508
Extracellular superoxide dismutase [Cu-Zn]	SODE_MOUSE	O09164	Sod3	6	251	1	1	4	27.4	18.90±0.42	18.80±0.46	0.93	0.7152	0.8582
Fibrinogen alpha chain	FIBA_MOUSE	E9PV24	Fga	23	789	17	17	77	87.4	25.00±0.80	24.89±0.82	0.92	0.8148	0.8845
Fibrinogen beta chain	FIBB_MOUSE	Q8K0E8	Fgb	71	481	23	23	138	54.7	25.28±0.59	25.09±0.51	0.88	0.5782	0.8232
Fibrinogen gamma chain	FIBG_MOUSE	Q8VCM7	Fgg	29	436	9	9	80	49.4	24.13±0.70	23.96±0.60	0.89	0.6651	0.8465
Fibronectin	FINC_MOUSE	P11276	Fn1	27	2477	42	42	175	272.4	25.25±0.33	25.88±0.39	1.55	0.0174	0.2433
Galectin-1	LEG1_MOUSE	P16045	Lgals1	47	135	6	6	102	14.9	25.65±0.40	25.25±0.41	0.76	0.1408	0.5514
Galectin-9	LEG9_MOUSE	O08573	Lgals9	4	353	1	1	2	40	19.78±0.15	19.70±0.40	0.94	0.6387	0.8361
Gelsolin	GELS_MOUSE	P13020	Gsn	46	780	26	26	234	85.9	27.42±0.21	27.63±0.27	1.16	0.2130	0.6389
Insulin-like growth factor-binding protein 7	IBP7_MOUSE	Q61581	Igfbp7	10	281	2	2	11	29	21.51±0.55	21.65±0.66	1.10	0.6992	0.8582
Integrin alpha-5	ITA5_MOUSE	P11688	Itga5	2	1053	2	2	3	115	19.59±0.86	20.34±0.19	1.68	0.0923	0.4875
Integrin alpha-6	ITA6_MOUSE	Q61739	Itga6	6	1091	5	5	16	122.1	22.20±0.33	22.46±0.55	1.20	0.3349	0.6827
Integrin alpha-7	ITA7_MOUSE	Q61738	Itga7	6	1179	5	5	18	129.2	21.08±0.20	21.30±0.15	1.17	0.1284	0.5392
Integrin beta-1	ITB1_MOUSE	P09055	Itgb1	18	798	10	10	35	88.2	23.69±0.11	23.84±0.19	1.11	0.2324	0.6508
Inter-alpha-trypsin inhibitor heavy chain H1	ITIH1_MOUSE	Q61702	Itih1	5	907	3	3	5	101	25.94±0.95	26.33±0.64	1.32	0.4432	0.7375

Kininogen-1	KNG1_MOUSE	O08677	Kng1	3	661	1	1	1	73.1	20.10±0.21	20.80±0.53	1.62	0.0112	0.1886
Lactadherin	MFGM_MOUSE	P21956	Mfge8	25	463	8	8	48	51.2	23.48±0.28	23.63±0.17	1.11	0.3655	0.6827
Laminin subunit alpha-2	LAMA2_MOUSE	Q60675	Lama2	39	3118	84	84	695	343.6	28.34±0.17	28.25±0.16	0.94	0.5121	0.7681
Laminin subunit alpha-4	LAMA4_MOUSE	P97927	Lama4	21	1816	28	28	172	201.7	25.45±0.14	25.44±0.20	0.99	0.9324	0.9436
Laminin subunit alpha-5	LAMA5_MOUSE	Q61001	Lama5	9	3718	21	21	110	403.8	24.37±0.17	24.34±0.09	0.98	0.7664	0.8699
Laminin subunit beta-1	LAMB1_MOUSE	P02469	Lamb1	38	1786	48	48	306	197	26.51±0.20	26.38±0.21	0.91	0.3901	0.6827
Laminin subunit beta-2	LAMB2_MOUSE	Q61292	Lamb2	35	1799	45	45	298	196.5	27.05±0.17	27.07±0.16	1.02	0.8703	0.9026
Laminin subunit gamma-1	LAMC1_MOUSE	P02468	Lamc1	47	1607	54	54	542	177.2	27.69±0.21	27.67±0.25	0.98	0.8634	0.9026
Lumican	LUM_MOUSE	P51885	Lum	53	338	15	15	126	38.2	25.40±0.47	25.61±0.45	1.16	0.4726	0.7375
Mast cell protease 4	MCPT4_MOUSE	P21812	Mcpt4	23	246	3	3	19	27.2	22.25±0.41	22.49±0.44	1.18	0.3675	0.6827
Microfibrillar-associated protein 5	MFAP5_MOUSE	Q9QZJ6	Mfap5	34	164	6	6	93	18.5	25.92±0.11	25.77±0.10	0.90	0.1766	0.5726
Mimecan	MIME_MOUSE	Q62000	Ogn	7	298	2	2	3	34	20.74±1.34	20.32±1.43	0.75	0.6079	0.8361
Nidogen-1	NID1_MOUSE	P10493	Nid1	55	1245	52	51	885	136.5	28.72±0.14	28.65±0.13	0.95	0.5597	0.8232
Nidogen-2	NID2_MOUSE	O88322	Nid2	38	1403	39	38	285	153.8	26.94±0.16	26.90±0.17	0.98	0.7834	0.8774
Osteoclast-stimulating factor 1	OSTF1_MOUSE	Q62422	Ostf1	27	215	4	4	5	23.8	21.20±0.98	20.06±1.61	0.45	0.1444	0.5514
Periostin	POSTN_MOUSE	Q62009	Postn	1	838	1	1	3	93.1	19.97±1.51	20.23±1.35	1.19	0.7660	0.8699
Peripherin	PERI_MOUSE	P15331	Prph	9	475	5	1	112	54.2	18.72±0.57	18.37±0.75	0.79	0.3792	0.6827
Progranulin	GRN_MOUSE	P28798	Grn	20	589	7	7	44	63.4	23.07±0.33	22.94±0.22	0.91	0.4932	0.7532
Prolargin	PRELP_MOUSE	Q9JK53	Prelp	23	378	6	6	33	43.3	23.56±0.28	23.26±0.27	0.81	0.1273	0.5392
Protein S100-A9	S10A9_MOUSE	P31725	S100a9	12	113	1	1	1	13	18.86±2.66	21.35±2.18	5.61	0.1130	0.5392
Serum amyloid A-1 protein	SAA1_MOUSE	P05366	Saa1	22	122	3	3	4	13.8	20.14±1.28	20.83±0.49	1.61	0.2868	0.6827
Tight junction protein ZO-1	ZO1_MOUSE	P39447	Tjp1	12	1745	10	10	30	194.6	21.92±0.42	21.04±0.10	0.55	0.0019	0.0540
Tight junction protein ZO-2	ZO2_MOUSE	Q9Z0U1	Tjp2	5	1167	4	4	14	131.2	21.29±0.17	21.14±0.37	0.90	0.3840	0.6827
Tubulointerstitial nephritis antigen-like	TINAL_MOUSE	Q99JR5	Tinagl1	40	466	14	14	104	52.6	25.39±0.25	25.49±0.30	1.07	0.6003	0.8361
Versican	CSPG2_MOUSE	Q62059	Vcan	1	3357	3	3	12	366.6	20.87±0.67	23.01±0.47	4.42	0.0001	0.0103
Vinculin	VINC_MOUSE	Q64727	Vcl	72	1066	67	67	713	116.6	28.62±0.23	28.30±0.19	0.80	0.0604	0.4875
Vitronectin	VTNC_MOUSE	P29788	Vtn	21	478	7	7	36	54.8	24.07±0.37	24.19±0.31	1.09	0.5773	0.8232

Supplemental Table IV. ECM and ECM-associated proteins identified by proteomics in the GuHCl extracts of the human ischaemic heart failure cohort (n=65).

Protein Name	Accession number	Uniprot ID	Gene name	Coverage [%]	# AAs	# Peptides	# Unique Peptides	# PSMs	MW (kDa)
Basement membrane-specific heparan sulfate proteoglycan core protein	PGBM_HUMAN	P98160	HSPG2	71	4391	205	205	9881	468.5
Collagen alpha-1(I) chain	CO1A1_HUMAN	P02452	COL1A1	69	1464	91	90	8652	138.9
Collagen alpha-2(I) chain	CO1A2_HUMAN	P08123	COL1A2	68	1366	70	70	5144	129.2
Collagen alpha-3(VI) chain	CO6A3_HUMAN	P12111	COL6A3	47	3177	148	148	5120	343.5
Decorin	PGS2_HUMAN	P07585	DCN	82	359	40	40	3742	39.7
Tenascin-X	TENX_HUMAN	P22105	TNXB	46	4242	132	132	3116	457.9
Gelsolin	GELS_HUMAN	P06396	GSN	66	782	51	51	2820	85.6
Collagen alpha-1(III) chain	CO3A1_HUMAN	P02461	COL3A1	60	1466	55	54	2799	138.5
Lumican	LUM_HUMAN	P51884	LUM	78	338	37	37	2739	38.4
Laminin subunit gamma-1	LAMC1_HUMAN	P11047	LAMC1	54	1609	76	76	2618	177.5
Nidogen-2	NID2_HUMAN	Q14112	NID2	55	1375	68	67	2607	151.2
Fibronectin	FINC_HUMAN	P02751	FN1	58	2386	90	90	2590	262.5
Tubulointerstitial nephritis antigen-like	TINAL_HUMAN	Q9GZM7	TINAGL1	83	467	33	33	2449	52.4
Vinculin	VINC_HUMAN	P18206	VCL	65	1134	69	69	2387	123.7
Nidogen-1	NID1_HUMAN	P14543	NID1	65	1247	56	55	2368	136.3
Prolargin	PRELP_HUMAN	P51888	PRELP	72	382	31	31	2322	43.8
Biglycan	PGS1_HUMAN	P21810	BGN	74	368	34	34	2231	41.6
Mimecan	MIME_HUMAN	P20774	OGN	55	298	31	31	2200	33.9
Collagen alpha-1(VI) chain	CO6A1_HUMAN	P12109	COL6A1	57	1028	50	50	1710	108.5
Laminin subunit beta-2	LAMB2_HUMAN	P55268	LAMB2	52	1798	65	65	1615	195.9
Collagen alpha-2(V) chain	CO5A2_HUMAN	P05997	COL5A2	49	1499	42	42	1557	144.8
Collagen alpha-2(VI) chain	CO6A2_HUMAN	P12110	COL6A2	40	1019	41	41	1279	108.5
Agrin	AGRIN_HUMAN	O00468	AGRN	39	2067	53	53	1277	217.1
Versican core protein	CSPG2_HUMAN	P13611	VCAN	25	3396	62	62	1170	372.6
Laminin subunit alpha-2	LAMA2_HUMAN	P24043	LAMA2	39	3122	84	84	1155	343.7
Galectin-3-binding protein	LG3BP_HUMAN	Q08380	LGALS3BP	52	585	25	25	1078	65.3
Periostin	POSTN_HUMAN	Q15063	POSTN	65	836	55	55	1028	93.3
Cathepsin D	CATD_HUMAN	P07339	CTSD	49	412	18	18	981	44.5
Beta-2-glycoprotein 1	APOH_HUMAN	P02749	APOH	80	345	23	23	928	38.3
Galectin-1	LEG1_HUMAN	P09382	LGALS1	82	135	13	13	925	14.7
Laminin subunit alpha-4	LAMA4_HUMAN	Q16363	LAMA4	41	1823	53	53	885	202.4

Fibrillin-1	FBN1_HUMAN	P35555	FBN1	22	2871	43	43	845	312
Target of Nesh-SH3	TARSH_HUMAN	Q7Z7G0	ABI3BP	61	1075	50	50	841	118.6
Dermatopontin	DERM_HUMAN	Q07507	DPT	67	201	12	12	756	24
Laminin subunit beta-1	LAMB1_HUMAN	P07942	LAMB1	38	1786	54	54	744	197.9
Laminin subunit alpha-5	LAMA5_HUMAN	O15230	LAMA5	21	3695	52	52	715	399.5
Collagen alpha-1(V) chain	CO5A1_HUMAN	P20908	COL5A1	22	1838	21	21	694	183.4
Basal cell adhesion molecule	BCAM_HUMAN	P50895	BCAM	52	628	24	24	655	67.4
Asporin	ASPN_HUMAN	Q9BXN1	ASPN	68	380	24	24	647	43.4
Collagen alpha-1(IV) chain	CO4A1_HUMAN	P02462	COL4A1	14	1669	15	13	603	160.5
Dystroglycan	DAG1_HUMAN	Q14118	DAG1	20	895	18	18	596	97.4
Alpha-1-antitrypsin	A1AT_HUMAN	P01009	SERPINA1	73	418	25	25	573	46.7
Collagen alpha-2(IV) chain	CO4A2_HUMAN	P08572	COL4A2	29	1712	25	25	495	167.4
Peroxiredoxin-1	PRDX1_HUMAN	Q06830	PRDX1	68	199	16	15	491	22.1
Alpha-1-antichymotrypsin	AACT_HUMAN	P01011	SERPINA3	65	423	23	23	484	47.6
Microfibrillar-associated protein 5	MFAP5_HUMAN	Q13361	MFAP5	32	173	10	10	476	19.6
Vitronectin	VTNC_HUMAN	P04004	VTN	30	478	14	14	471	54.3
Galectin-3	LEG3_HUMAN	P17931	LGALS3	40	250	10	10	462	26.1
Y-box-binding protein 1	YBOX1_HUMAN	P67809	YBX1	51	324	11	7	438	35.9
Cysteine-rich protein 2	CRIP2_HUMAN	P52943	CRIP2	73	208	9	9	434	22.5
Clusterin	CLUS_HUMAN	P10909	CLU	39	449	15	15	421	52.5
Transforming growth factor-beta-induced protein ig-h3	BGH3_HUMAN	Q15582	TGFBI	49	683	24	24	413	74.6
Chymase	CMA1_HUMAN	P23946	CMA1	61	247	14	14	413	27.3
Cadherin-13	CAD13_HUMAN	P55290	CDH13	27	713	12	12	411	78.2
Peroxiredoxin-2	PRDX2_HUMAN	P32119	PRDX2	69	198	15	14	395	21.9
Collagen alpha-1(XVIII) chain	COIA1_HUMAN	P39060	COL18A1	20	1754	20	20	375	178.1
Collagen alpha-1(XV) chain	COFA1_HUMAN	P39059	COL15A1	16	1388	16	16	372	141.6
Collagen alpha-1(II) chain	CO2A1_HUMAN	P02458	COL2A1	11	1487	7	5	346	141.7
Annexin A2	ANXA2_HUMAN	P07355	ANXA2	52	339	16	16	338	38.6
Serum amyloid P-component	SAMP_HUMAN	P02743	APCS	45	223	12	12	298	25.4
Platelet glycoprotein 4	CD36_HUMAN	P16671	CD36	44	472	15	15	293	53
Extracellular superoxide dismutase [Cu-Zn]	SODE_HUMAN	P08294	SOD3	72	240	12	12	273	25.8
Tryptase beta-2	TRYB2_HUMAN	P20231	TPSB2	38	275	8	8	252	30.5
Apolipoprotein A-I	APOA1_HUMAN	P02647	APOA1	63	267	20	20	247	30.8
Latent-transforming growth factor beta-binding protein 2	LTBP2_HUMAN	Q14767	LTBP2	22	1821	23	23	241	194.9
Collagen alpha-3(V) chain	CO5A3_HUMAN	P25940	COL5A3	10	1745	11	11	224	172
EMILIN-1	EMIL1_HUMAN	Q9Y6C2	EMILIN1	21	1016	17	17	211	106.6
Thrombospondin type-1 domain-containing protein 4	THSD4_HUMAN	Q6ZMP0	THSD4	22	1018	17	17	203	112.4

Alpha-1B-glycoprotein	A1BG_HUMAN	P04217	A1BG	37	495	10	10	198	54.2
Collagen alpha-1(XIV) chain	COEA1_HUMAN	Q05707	COL14A1	32	1796	37	37	197	193.4
Microfibril-associated glycoprotein 4	MFAP4_HUMAN	P55083	MFAP4	62	255	11	11	195	28.6
von Willebrand factor A domain-containing protein 1	VWA1_HUMAN	Q6PCB0	VWA1	42	445	11	11	193	46.8
Lysozyme C	LYSC_HUMAN	P61626	LYZ	55	148	7	7	189	16.5
Lactadherin	MFGM_HUMAN	Q08431	MFGE8	37	387	12	12	180	43.1
Protein AMBP	AMBP_HUMAN	P02760	AMBP	19	352	8	8	164	39
Beta-2-microglobulin	B2MG_HUMAN	P61769	B2M	38	119	4	4	163	13.7
Multimerin-2	MMRN2_HUMAN	Q9H8L6	MMRN2	20	949	13	13	162	104.3
Cathepsin G	CATG_HUMAN	P08311	CTSG	44	255	9	9	159	28.8
Mast cell carboxypeptidase A	CBPA3_HUMAN	P15088	CPA3	18	417	8	8	159	48.6
Prostaglandin-H2 D-isomerase	PTGDS_HUMAN	P41222	PTGDS	55	190	8	8	159	21
Insulin-like growth factor-binding protein 7	IBP7_HUMAN	Q16270	IGFBP7	43	282	8	8	157	29.1
Protein disulfide-isomerase	PDIA1_HUMAN	P07237	P4HB	30	508	14	14	147	57.1
Cartilage intermediate layer protein 1	CILP1_HUMAN	O75339	CILP	11	1184	10	10	146	132.5
Cell surface glycoprotein MUC18	MUC18_HUMAN	P43121	MCAM	23	646	10	10	141	71.6
Calpastatin	ICAL_HUMAN	P20810	CAST	38	708	14	14	135	76.5
Fibromodulin	FMOD_HUMAN	Q06828	FMOD	46	376	11	11	133	43.2
Integrin beta-1	ITB1_HUMAN	P05556	ITGB1	24	798	12	12	130	88.4
Annexin A11	ANX11_HUMAN	P50995	ANXA11	17	505	8	8	129	54.4
Serpin B6	SPB6_HUMAN	P35237	SERPINB6	32	376	9	9	127	42.6
Prosaposin	SAP_HUMAN	P07602	PSAP	9	524	6	6	115	58.1
Serpin H1	SERPH_HUMAN	P50454	SERPINH1	49	418	12	12	106	46.4
Hyaluronan and proteoglycan link protein 1	HPLN1_HUMAN	P10915	HAPLN1	55	354	14	14	97	40.1
Neutrophil defensin 1	DEF1_HUMAN	P59665	DEFA1	20	94	4	4	91	10.2
Cystatin-B	CYTB_HUMAN	P04080	CSTB	24	98	2	2	89	11.1
CD59 glycoprotein	CD59_HUMAN	P13987	CD59	38	128	4	4	85	14.2
Protein S100-A9	S10A9_HUMAN	P06702	S100A9	60	114	6	6	84	13.2
Thioredoxin	THIO_HUMAN	P10599	TXN	47	105	4	4	82	11.7
Apolipoprotein A-II	APOA2_HUMAN	P02652	APOA2	68	100	5	5	80	11.2
Protein S100-A10	S10AA_HUMAN	P60903	S100A10	45	97	3	3	76	11.2
Leukocyte elastase inhibitor	ILEU_HUMAN	P30740	SERPINB1	37	379	11	11	68	42.7
Inter-alpha-trypsin inhibitor heavy chain H2	ITIH2_HUMAN	P19823	ITIH2	16	946	10	10	68	106.4
Hepatoma-derived growth factor	HDGF_HUMAN	P51858	HDGF	18	240	3	3	66	26.8
Apolipoprotein A-IV	APOA4_HUMAN	P06727	APOA4	36	396	13	13	65	45.4
Protein S100-A7	S10A7_HUMAN	P31151	S100A7	73	101	8	8	65	11.5
Apolipoprotein C-III	APOC3_HUMAN	P02656	APOC3	41	99	5	5	61	10.8

Plexin-B2	PLXB2_HUMAN	O15031	PLXNB2	8	1838	10	10	61	205
Tenascin	TENA_HUMAN	P24821	TNC	26	2201	33	33	60	240.7
Annexin A6	ANXA6_HUMAN	P08133	ANXA6	10	673	5	5	59	75.8
Somatomedin-B and thrombospondin type-1 domain-containing protein	SBSPO_HUMAN	Q8IVN8	SBSPON	33	264	5	5	57	29.6
Cathepsin B	CATB_HUMAN	P07858	CTSB	14	339	4	4	55	37.8
Apolipoprotein E	APOE_HUMAN	P02649	APOE	26	317	6	6	51	36.1
Procathepsin L	CATL1_HUMAN	P07711	CTSL	20	333	4	4	49	37.5
Adipocyte enhancer-binding protein 1	AEBP1_HUMAN	Q8IUX7	AEBP1	14	1158	9	9	46	130.8
Inter-alpha-trypsin inhibitor heavy chain H1	ITIH1_HUMAN	P19827	ITIH1	7	911	5	5	45	101.3
Protein S100-A13	S10AD_HUMAN	Q99584	S100A13	30	98	4	4	45	11.5
Neutrophil elastase	ELNE_HUMAN	P08246	ELANE	22	267	4	4	42	28.5
Cystatin-C	CYTC_HUMAN	P01034	CST3	19	146	3	3	41	15.8
Kininogen-1	KNG1_HUMAN	P01042	KNG1	8	644	3	3	41	71.9
Signal recognition particle 14 kDa protein	SRP14_HUMAN	P37108	SRP14	38	136	4	4	39	14.6
Serine protease HTRA1	HTRA1_HUMAN	Q92743	HTRA1	21	480	7	7	37	51.3
Collagen alpha-6(VI) chain	CO6A6_HUMAN	A6NMZ7	COL6A6	3	2263	6	6	36	247
Annexin A1	ANXA1_HUMAN	P04083	ANXA1	29	346	6	6	31	38.7
Fibulin-2	FBLN2_HUMAN	P98095	FBLN2	8	1184	6	6	29	126.5
Olfactomedin-like protein 1	OLFL1_HUMAN	Q6UWY5	OLFML1	26	402	9	9	29	45.9
Phospholipase A2, membrane associated	PA2GA_HUMAN	P14555	PLA2G2A	24	144	2	2	27	16.1
Secreted frizzled-related protein 1	SFRP1_HUMAN	Q8N474	SFRP1	21	314	4	4	26	35.4
Integrin alpha-V	ITAV_HUMAN	P06756	ITGAV	4	1048	4	4	25	116
Antithrombin-III	ANT3_HUMAN	P01008	SERPINC1	20	464	7	7	24	52.6
Hornerin	HORN_HUMAN	Q86YZ3	HRNR	8	2850	3	3	23	282.2
Intercellular adhesion molecule 1	ICAM1_HUMAN	P05362	ICAM1	9	532	4	4	23	57.8
Mesencephalic astrocyte-derived neurotrophic factor	MANF_HUMAN	P55145	MANF	20	182	3	3	22	20.7
Latent-transforming growth factor beta-binding protein 4	LTBP4_HUMAN	Q8N2S1	LTBP4	9	1624	9	9	21	173.3
Collagen alpha-1(XII) chain	COCA1_HUMAN	Q99715	COL12A1	5	3063	9	9	20	332.9
Dermcidin	DCD_HUMAN	P81605	DCD	35	110	3	3	20	11.3
Alpha-2-HS-glycoprotein	FETUA_HUMAN	P02765	AHSG	11	367	3	3	20	39.3
Tetranectin	TETN_HUMAN	P05452	CLEC3B	22	202	3	3	20	22.5
Alpha-1-acid glycoprotein 1	A1AG1_HUMAN	P02763	ORM1	28	201	4	4	19	23.5
EMILIN-2	EMIL2_HUMAN	Q9BXX0	EMILIN2	8	1053	5	5	19	115.6
Integrin alpha-1	ITA1_HUMAN	P56199	ITGA1	5	1179	5	5	19	130.8
Matrix Gla protein	MGP_HUMAN	P08493	MGP	23	103	2	2	17	12.3
Serum amyloid A-1 protein	SAA1_HUMAN	P0DJI8	SAA1	41	122	4	3	16	13.5

Alpha-2-macroglobulin	A2MG_HUMAN	P01023	A2M	10	1474	12	12	15	163.2
Collagen alpha-1(XXVIII) chain	COSA1_HUMAN	Q2UY09	COL28A1	7	1125	5	5	15	116.6
Transgelin-2	TAGL2_HUMAN	P37802	TAGLN2	12	199	2	2	15	22.4
Progranulin	GRN_HUMAN	P28799	GRN	6	593	3	3	14	63.5
Aggrecan core protein	PGCA_HUMAN	P16112	ACAN	3	2415	5	5	14	250
Thrombospondin-4	TSP4_HUMAN	P35443	THBS4	6	961	4	4	14	105.8
Protein S100-A8	S10A8_HUMAN	P05109	S100A8	41	93	4	4	13	10.8
Fibulin-5	FBLN5_HUMAN	Q9UBX5	FBLN5	10	448	4	4	12	50.1
Olfactomedin-like protein 3	OLFL3_HUMAN	Q9NRN5	OLFML3	21	406	6	6	11	46
Papilin	PPN_HUMAN	O95428	PAPLN	5	1278	3	3	11	137.6
Palmitoyl-protein thioesterase 1	PPT1_HUMAN	P50897	PPT1	15	306	3	3	11	34.2
Chondroitin sulfate proteoglycan 4	CSPG4_HUMAN	Q6UVK1	CSPG4	5	2322	7	7	10	250.4
Serum amyloid A-2 protein	SAA2_HUMAN	P0DJI9	SAA2	39	122	4	3	10	13.5
Apolipoprotein C-I	APOC1_HUMAN	P02654	APOC1	24	83	2	2	9	9.3
Collagen alpha-1(XVI) chain	COGA1_HUMAN	Q07092	COL16A1	3	1604	2	2	9	157.7
Macrophage migration inhibitory factor	MIF_HUMAN	P14174	MIF	17	115	2	2	9	12.5
Serpin B4	SPB4_HUMAN	P48594	SERPINB4	14	390	6	6	8	44.8
Amyloid-beta precursor protein	A4_HUMAN	P05067	APP	3	770	2	2	7	86.9
Angiogenin	ANGI_HUMAN	P03950	ANG	24	147	3	3	7	16.5
Apolipoprotein D	APOD_HUMAN	P05090	APOD	26	189	3	3	7	21.3
Fibulin-1	FBLN1_HUMAN	P23142	FBLN1	9	703	3	3	7	77.2
Neutral alpha-glucosidase AB	GANAB_HUMAN	Q14697	GANAB	6	944	3	3	7	106.8
Hepatoma-derived growth factor-related protein 3	HDGR3_HUMAN	Q9Y3E1	HDGFL3	20	203	3	3	7	22.6
Inter-alpha-trypsin inhibitor heavy chain H3	ITIH3_HUMAN	Q06033	ITIH3	5	890	4	4	7	99.8
Pentraxin-related protein PTX3	PTX3_HUMAN	P26022	PTX3	15	381	4	4	7	41.9
Procollagen C-endopeptidase enhancer 1	PCOC1_HUMAN	Q15113	PCOLCE	13	449	3	3	6	47.9
Pleiotrophin	PTN_HUMAN	P21246	PTN	24	168	2	2	6	18.9
ADAMTS-like protein 4	ATL4_HUMAN	Q6UY14	ADAMTSL4	4	1074	2	2	4	116.5
Annexin A5	ANXA5_HUMAN	P08758	ANXA5	8	320	2	2	3	35.9
Cartilage intermediate layer protein 2	CILP2_HUMAN	Q8IUL8	CILP2	2	1156	2	2	3	126.2
Spondin-1	SPON1_HUMAN	Q9HCB6	SPON1	5	807	2	2	3	90.9
Fibroleukin	FGL2_HUMAN	Q14314	FGL2	6	439	2	2	2	50.2
Lysyl oxidase homolog 1	LOXL1_HUMAN	Q08397	LOXL1	4	574	2	2	2	63.1
Matrix-remodeling-associated protein 5	MXRA5_HUMAN	Q9NR99	MXRA5	1	2828	2	2	2	312
Peroxidasin homolog	PXDN_HUMAN	Q92626	PXDN	2	1479	2	2	2	165.2
von Willebrand factor	VWF_HUMAN	P04275	VWF	1	2813	2	2	2	309.1

Supplemental Table V. Proteins identified by proteomics in the SDS extracts of the human ischaemic heart failure cohort (n=65).

Protein Name	Accession number	Uniprot ID	Gene name	MW (kDa)	Sequence coverage [%]	# Unique peptides	Total PSMs	Quantified Yes/No
Myosin-7	MYH7_HUMAN	P12883	MYH7	223	81	103	21342	Yes
Myosin-6	MYH6_HUMAN	P13533	MYH6	223.6	56	16	13448	No
Myosin regulatory light chain 2, ventricular/cardiac muscle isoform	MLRV_HUMAN	P10916	MYL2	18.8	96	33	13029	Yes
Actin, alpha skeletal muscle	ACTS_HUMAN	P68133	ACTA1	42	94	5	11799	Yes
Actin, aortic smooth muscle	ACTA_HUMAN	P62736	ACTA2	42	94	7	11641	Yes
Alpha-actinin-2	ACTN2_HUMAN	P35609	ACTN2	103.8	92	93	9831	Yes
Titin	TITIN_HUMAN	Q8WZ42	TTN	3813.7	17	506	9571	Yes
Myosin light chain 3	MYL3_HUMAN	P08590	MYL3	21.9	95	35	9246	Yes
Actin, cytoplasmic 1	ACTB_HUMAN	P60709	ACTB	41.7	79	15	8424	Yes
Serum albumin	ALBU_HUMAN	P02768	ALB	69.3	93	100	8371	Yes
Tropomyosin alpha-1 chain	TPM1_HUMAN	P09493	TPM1	32.7	94	36	7824	Yes
ATP synthase subunit beta, mitochondrial	ATPB_HUMAN	P06576	ATP5F1B	56.5	79	46	7567	Yes
Neuroblast differentiation-associated protein AHNAK	AHNK_HUMAN	Q09666	AHNAK	628.7	77	299	6533	Yes
Vinculin	VINC_HUMAN	P18206	VCL	123.7	80	111	6215	Yes
Trifunctional enzyme subunit alpha, mitochondrial	ECHA_HUMAN	P40939	HADHA	82.9	81	88	5992	Yes
ATP synthase subunit alpha, mitochondrial	ATPA_HUMAN	P25705	ATP5F1A	59.7	78	69	5546	Yes
Myosin-binding protein C, cardiac-type	MYPC3_HUMAN	Q14896	MYBPC3	140.7	78	104	5508	Yes
Aconitate hydratase, mitochondrial	ACON_HUMAN	Q99798	ACO2	85.4	75	80	5413	Yes
Troponin T, cardiac muscle	TNNT2_HUMAN	P45379	TNNT2	35.9	64	45	5241	Yes
Creatine kinase S-type, mitochondrial	KCRS_HUMAN	P17540	CKMT2	47.5	78	44	5136	Yes
Spectrin alpha chain, non-erythrocytic 1	SPTN1_HUMAN	Q13813	SPTAN1	284.4	68	199	4782	Yes
Filamin-C	FLNC_HUMAN	Q14315	FLNC	290.8	64	132	4768	Yes
Desmin	DESM_HUMAN	P17661	DES	53.5	91	65	4681	Yes
Tropomyosin beta chain	TPM2_HUMAN	P07951	TPM2	32.8	83	30	4551	Yes
Troponin C, slow skeletal and cardiac muscles	TNNC1_HUMAN	P63316	TNNC1	18.4	93	17	4486	Yes
Vimentin	VIME_HUMAN	P08670	VIM	53.6	95	62	4459	Yes
Basement membrane-specific heparan sulfate proteoglycan core protein	PGBM_HUMAN	P98160	HSPG2	468.5	54	157	4431	Yes
60 kDa heat shock protein, mitochondrial	CH60_HUMAN	P10809	HSPD1	61	86	73	4050	Yes
Creatine kinase M-type	KCRM_HUMAN	P06732	CKM	43.1	83	52	4049	Yes
Stress-70 protein, mitochondrial	GRP75_HUMAN	P38646	HSPA9	73.6	71	66	4037	Yes

Tropomyosin alpha-3 chain	TPM3_HUMAN	P06753	TPM3	32.9	89	23	4017	Yes
Myomesin-2	MYOM2_HUMAN	P54296	MYOM2	164.8	82	123	4001	Yes
Cytochrome b-c1 complex subunit 1, mitochondrial	QCR1_HUMAN	P31930	UQCRC1	52.6	73	42	3855	Yes
Alpha-actinin-1	ACTN1_HUMAN	P12814	ACTN1	103	68	37	3815	Yes
Myosin-3	MYH3_HUMAN	P11055	MYH3	223.8	18	3	3716	No
Beta-actin-like protein 2	ACTBL_HUMAN	Q562R1	ACTBL2	42	39	7	3715	No
Laminin subunit alpha-2	LAMA2_HUMAN	P24043	LAMA2	343.7	53	149	3661	Yes
Myosin-4	MYH4_HUMAN	Q9Y623	MYH4	222.9	19	4	3627	No
Myomesin-1	MYOM1_HUMAN	P52179	MYOM1	187.5	70	123	3619	Yes
2-oxoglutarate dehydrogenase, mitochondrial	ODO1_HUMAN	Q02218	OGDH	115.9	66	68	3538	Yes
Endoplasmic reticulum chaperone BiP	BIP_HUMAN	P11021	HSPA5	72.3	68	60	3515	Yes
Aspartate aminotransferase, mitochondrial	AATM_HUMAN	P00505	GOT2	47.5	85	43	3511	Yes
Dihydrolipoyl dehydrogenase, mitochondrial	DLDH_HUMAN	P09622	DLD	54.1	81	46	3499	Yes
Prelamin-A/C	LMNA_HUMAN	P02545	LMNA	74.1	72	71	3419	Yes
Malate dehydrogenase, mitochondrial	MDHM_HUMAN	P40926	MDH2	35.5	76	41	3412	Yes
Alpha-actinin-4	ACTN4_HUMAN	O43707	ACTN4	104.8	76	55	3400	Yes
Serotransferrin	TRFE_HUMAN	P02787	TF	77	78	76	3393	Yes
Transitional endoplasmic reticulum ATPase	TERA_HUMAN	P55072	VCP	89.3	82	80	3392	Yes
Glycogen phosphorylase, brain form	PYGB_HUMAN	P11216	PYGB	96.6	71	68	3320	Yes
Succinyl-CoA:3-ketoacid coenzyme A transferase 1, mitochondrial	SCOT1_HUMAN	P55809	OXCT1	56.1	70	39	3253	Yes
Spectrin beta chain, non-erythrocytic 1	SPTB2_HUMAN	Q01082	SPTBN1	274.4	64	140	3238	Yes
Heat shock cognate 71 kDa protein	HSP7C_HUMAN	P11142	HSPA8	70.9	72	42	3199	Yes
Filamin-A	FLNA_HUMAN	P21333	FLNA	280.6	58	120	3189	Yes
Trifunctional enzyme subunit beta, mitochondrial	ECHB_HUMAN	P55084	HADHB	51.3	75	45	3164	Yes
NADH-ubiquinone oxidoreductase 75 kDa subunit, mitochondrial	NDUS1_HUMAN	P28331	NDUFS1	79.4	76	58	3128	Yes
Medium-chain specific acyl-CoA dehydrogenase, mitochondrial	ACADM_HUMAN	P11310	ACADM	46.6	68	45	3113	Yes
Talin-1	TLN1_HUMAN	Q9Y490	TLN1	269.6	65	127	3095	Yes
Sarcoplasmic/endoplasmic reticulum calcium ATPase 2	AT2A2_HUMAN	P16615	ATP2A2	114.7	49	58	3060	Yes
Troponin I, cardiac muscle	TNNI3_HUMAN	P19429	TNNI3	24	71	28	3037	Yes
Heat shock protein HSP 90-alpha	HS90A_HUMAN	P07900	HSP90AA1	84.6	68	41	3031	Yes
Annexin A6	ANXA6_HUMAN	P08133	ANXA6	75.8	81	70	2912	Yes
Heat shock protein HSP 90-beta	HS90B_HUMAN	P08238	HSP90AB1	83.2	67	34	2816	Yes
Xin actin-binding repeat-containing protein 1	XIRP1_HUMAN	Q702N8	XIRP1	198.4	58	82	2805	Yes
Plectin	PLEC_HUMAN	Q15149	PLEC	531.5	49	212	2736	Yes
Tropomyosin alpha-4 chain	TPM4_HUMAN	P67936	TPM4	28.5	75	16	2701	Yes
NAD(P) transhydrogenase, mitochondrial	NNTM_HUMAN	Q13423	NNT	113.8	47	62	2700	Yes

Fumarate hydratase, mitochondrial	FUMH_HUMAN	P07954	FH	54.6	72	34	2659	Yes
Isocitrate dehydrogenase [NADP], mitochondrial	IDHP_HUMAN	P48735	IDH2	50.9	62	37	2659	Yes
Laminin subunit gamma-1	LAMC1_HUMAN	P11047	LAMC1	177.5	51	79	2635	Yes
3-ketoacyl-CoA thiolase, mitochondrial	THIM_HUMAN	P42765	ACAA2	41.9	94	43	2627	Yes
MICOS complex subunit MIC60	MIC60_HUMAN	Q16891	IMMT	83.6	74	77	2621	Yes
Heat shock 70 kDa protein 1A	HS71A_HUMAN	P0DMV8	HSPA1A	70	71	51	2615	Yes
Endoplasmin	ENPL_HUMAN	P14625	HSP90B1	92.4	58	57	2590	Yes
Dystrophin	DMD_HUMAN	P11532	DMD	426.5	47	166	2544	Yes
Protein disulfide-isomerase	PDIA1_HUMAN	P07237	P4HB	57.1	76	51	2502	Yes
Collagen alpha-3(VI) chain	CO6A3_HUMAN	P12111	COL6A3	343.5	32	99	2493	Yes
Hexokinase-1	HXK1_HUMAN	P19367	HK1	102.4	59	69	2427	Yes
Glycogen debranching enzyme	GDE_HUMAN	P35573	AGL	174.7	64	92	2383	Yes
Acetyl-CoA acetyltransferase, mitochondrial	THIL_HUMAN	P24752	ACAT1	45.2	78	41	2382	Yes
Pyruvate kinase PKM	KPYM_HUMAN	P14618	PKM	57.9	79	52	2355	Yes
Aldehyde dehydrogenase, mitochondrial	ALDH2_HUMAN	P05091	ALDH2	56.3	73	37	2304	Yes
Delta(3,5)-Delta(2,4)-dienoyl-CoA isomerase, mitochondrial	ECH1_HUMAN	Q13011	ECH1	35.8	85	26	2272	Yes
Cytochrome b-c1 complex subunit 2, mitochondrial	QCR2_HUMAN	P22695	UQCRC2	48.4	67	36	2226	Yes
Complement C3	CO3_HUMAN	P01024	C3	187	61	96	2131	Yes
Fructose-bisphosphate aldolase A	ALDOA_HUMAN	P04075	ALDOA	39.4	97	39	2084	Yes
Myoglobin	MYG_HUMAN	P02144	MB	17.2	81	29	2072	Yes
Synemin	SYNEM_HUMAN	O15061	SYNM	172.7	55	90	2042	Yes
Pyruvate dehydrogenase E1 component subunit alpha, somatic form, mitochondrial	ODPA_HUMAN	P08559	PDHA1	43.3	83	43	2037	Yes
Citrate synthase, mitochondrial	CISY_HUMAN	O75390	CS	51.7	65	32	2018	Yes
Protein-glutamine gamma-glutamyltransferase 2	TGM2_HUMAN	P21980	TGM2	77.3	62	42	2012	Yes
Very long-chain specific acyl-CoA dehydrogenase, mitochondrial	ACADV_HUMAN	P49748	ACADVL	70.3	67	47	1999	Yes
Myosin-11	MYH11_HUMAN	P35749	MYH11	227.2	56	103	1951	Yes
Myozenin-2	MYOZ2_HUMAN	Q9NPC6	MYOZ2	29.9	73	28	1951	Yes
Sarcalumenin	SRCA_HUMAN	Q86TD4	SRL	100.7	42	43	1943	Yes
Laminin subunit beta-1	LAMB1_HUMAN	P07942	LAMB1	197.9	46	79	1926	Yes
Catenin alpha-1	CTNA1_HUMAN	P35221	CTNNA1	100	73	70	1926	Yes
2,4-dienoyl-CoA reductase, mitochondrial	DECR_HUMAN	Q16698	DECR1	36	66	31	1925	Yes
Keratin, type II cytoskeletal 1	K2C1_HUMAN	P04264	KRT1	66	69	50	1910	Yes
Heat shock protein beta-1	HSPB1_HUMAN	P04792	HSPB1	22.8	91	20	1892	Yes
Leucine-rich PPR motif-containing protein, mitochondrial	LPPRC_HUMAN	P42704	LRPPRC	157.8	52	75	1877	Yes
Glyceraldehyde-3-phosphate dehydrogenase	G3P_HUMAN	P04406	GAPDH	36	85	33	1874	Yes

Nestin	NEST_HUMAN	P48681	NES	177.3	55	82	1860	Yes
Electron transfer flavoprotein-ubiquinone oxidoreductase, mitochondrial	ETFD_HUMAN	Q16134	ETFDH	68.5	69	44	1860	Yes
Nebulette	NEBL_HUMAN	O76041	NEBL	116.4	68	87	1849	Yes
LIM domain-binding protein 3	LDB3_HUMAN	O75112	LDB3	77.1	56	36	1824	Yes
Succinate--CoA ligase [GDP-forming] subunit beta, mitochondrial	SUCB2_HUMAN	Q96I99	SUCLG2	46.5	69	36	1810	Yes
Glutamate dehydrogenase 1, mitochondrial	DHE3_HUMAN	P00367	GLUD1	61.4	69	15	1794	Yes
Talin-2	TLN2_HUMAN	Q9Y4G6	TLN2	271.4	49	88	1793	Yes
Electron transfer flavoprotein subunit beta	ETFB_HUMAN	P38117	ETFB	27.8	87	26	1792	Yes
Phosphoglycerate kinase 1	PGK1_HUMAN	P00558	PGK1	44.6	89	39	1789	Yes
Fatty acid-binding protein, heart	FABPH_HUMAN	P05413	FABP3	14.8	88	21	1778	Yes
Apoptosis-inducing factor 1, mitochondrial	AIFM1_HUMAN	O95831	AIFM1	66.9	62	35	1763	Yes
Gelsolin	GELS_HUMAN	P06396	GSN	85.6	60	44	1747	Yes
Protein disulfide-isomerase A3	PDIA3_HUMAN	P30101	PDIA3	56.7	70	45	1744	Yes
Annexin A2	ANXA2_HUMAN	P07355	ANXA2	38.6	78	41	1734	Yes
Sorbin and SH3 domain-containing protein 1	SRBS1_HUMAN	Q9BX66	SORBS1	142.4	55	68	1700	Yes
Pyruvate dehydrogenase E1 component subunit beta, mitochondrial	ODPB_HUMAN	P11177	PDHB	39.2	75	22	1691	Yes
Heat shock 70 kDa protein 4	HSP74_HUMAN	P34932	HSPA4	94.3	78	58	1690	Yes
Sarcoplasmic reticulum histidine-rich calcium-binding protein	SRCH_HUMAN	P23327	HRC	80.2	70	43	1688	Yes
14-3-3 protein epsilon	1433E_HUMAN	P62258	YWHAE	29.2	76	26	1657	Yes
Hydroxyacyl-coenzyme A dehydrogenase, mitochondrial	HCDH_HUMAN	Q16836	HADH	34.3	79	33	1646	Yes
Biglycan	PGS1_HUMAN	P21810	BGN	41.6	63	29	1624	Yes
Alpha-crystallin B chain	CRYAB_HUMAN	P02511	CRYAB	20.1	91	26	1615	Yes
Malate dehydrogenase, cytoplasmic	MDHC_HUMAN	P40925	MDH1	36.4	61	33	1612	Yes
Carnitine O-palmitoyltransferase 2, mitochondrial	CPT2_HUMAN	P23786	CPT2	73.7	68	52	1611	Yes
Dynamin-like 120 kDa protein, mitochondrial	OPA1_HUMAN	O60313	OPA1	111.6	63	67	1601	Yes
Alpha-enolase	ENOA_HUMAN	P06733	ENO1	47.1	71	36	1596	Yes
Sorbin and SH3 domain-containing protein 2	SRBS2_HUMAN	O94875	SORBS2	124	38	46	1592	Yes
Keratin, type I cytoskeletal 10	K1C10_HUMAN	P13645	KRT10	58.8	63	32	1589	Yes
Periostin	POSTN_HUMAN	Q15063	POSTN	93.3	55	53	1586	Yes
Succinate dehydrogenase [ubiquinone] flavoprotein subunit, mitochondrial	SDHA_HUMAN	P31040	SDHA	72.6	72	39	1573	Yes
Catenin beta-1	CTNB1_HUMAN	P35222	CTNNB1	85.4	59	36	1566	Yes
Perilipin-4	PLIN4_HUMAN	Q96Q06	PLIN4	134.3	71	63	1563	Yes
Neutral alpha-glucosidase AB	GANAB_HUMAN	Q14697	GANAB	106.8	62	50	1557	Yes
Superoxide dismutase [Mn], mitochondrial	SODM_HUMAN	P04179	SOD2	24.7	82	21	1548	Yes

Methylmalonate-semialdehyde dehydrogenase [acylating], mitochondrial	MMSA_HUMAN	Q02252	ALDH6A1	57.8	67	42	1548	Yes
Succinate--CoA ligase [ADP-forming] subunit beta, mitochondrial	SUCB1_HUMAN	Q9P2R7	SUCLA2	50.3	60	31	1545	Yes
Kinectin	KTN1_HUMAN	Q86UP2	KTN1	156.2	59	85	1544	Yes
Keratin, type II cytoskeletal 2 epidermal	K22E_HUMAN	P35908	KRT2	65.4	83	41	1541	Yes
Heat shock-related 70 kDa protein 2	HSP72_HUMAN	P54652	HSPA2	70	63	24	1536	Yes
NADH dehydrogenase [ubiquinone] flavoprotein 1, mitochondrial	NDUV1_HUMAN	P49821	NDUFV1	50.8	73	37	1521	Yes
Sarcolemmal membrane-associated protein	SLMAP_HUMAN	Q14BN4	SLMAP	95.1	33	51	1508	Yes
AFG3-like protein 2	AFG32_HUMAN	Q9Y4W6	AFG3L2	88.5	48	47	1503	Yes
Glycogen phosphorylase, muscle form	PYGM_HUMAN	P11217	PYGM	97	67	50	1492	Yes
Apolipoprotein A-I	APOA1_HUMAN	P02647	APOA1	30.8	79	32	1485	Yes
Fibrillin-1	FBN1_HUMAN	P35555	FBN1	312	28	66	1484	Yes
Long-chain-fatty-acid--CoA ligase 1	ACSL1_HUMAN	P33121	ACSL1	77.9	54	38	1474	Yes
Enoyl-CoA hydratase, mitochondrial	ECHM_HUMAN	P30084	ECHS1	31.4	71	32	1471	Yes
Methylcrotonoyl-CoA carboxylase beta chain, mitochondrial	MCCB_HUMAN	Q9HCC0	MCCC2	61.3	67	36	1471	Yes
Lumican	LUM_HUMAN	P51884	LUM	38.4	49	25	1465	Yes
L-lactate dehydrogenase B chain	LDHB_HUMAN	P07195	LDHB	36.6	64	26	1460	Yes
Tubulin beta-4B chain	TBB4B_HUMAN	P68371	TUBB4B	49.8	75	2	1460	Yes
ADP/ATP translocase 1	ADT1_HUMAN	P12235	SLC25A4	33	68	11	1459	Yes
14-3-3 protein gamma	1433G_HUMAN	P61981	YWHAG	28.3	75	16	1449	Yes
Glucose-6-phosphate isomerase	G6PI_HUMAN	P06744	GPI	63.1	65	41	1437	Yes
Calreticulin	CALR_HUMAN	P27797	CALR	48.1	66	34	1433	Yes
Laminin subunit beta-2	LAMB2_HUMAN	P55268	LAMB2	195.9	53	77	1420	Yes
Cytochrome c oxidase subunit 4 isoform 1, mitochondrial	COX41_HUMAN	P13073	COX4I1	19.6	66	20	1417	Yes
Isocitrate dehydrogenase [NAD] subunit alpha, mitochondrial	IDH3A_HUMAN	P50213	IDH3A	39.6	66	34	1415	Yes
Cathepsin D	CATD_HUMAN	P07339	CTSD	44.5	61	28	1413	Yes
Glutamine amidotransferase-like class 1 domain-containing protein 3A, mitochondrial	GAL3A_HUMAN	P0DPI2	GATD3A	28.2	68	22	1412	Yes
Electron transfer flavoprotein subunit alpha, mitochondrial	ETFA_HUMAN	P13804	ETFA	35.1	80	28	1408	Yes
Junction plakoglobin	PLAK_HUMAN	P14923	JUP	81.7	60	32	1407	Yes
Creatine kinase B-type	KCRB_HUMAN	P12277	CKB	42.6	77	25	1396	Yes
ATP synthase-coupling factor 6, mitochondrial	ATP5J_HUMAN	P18859	ATP5PF	12.6	59	13	1393	Yes
Myosin-9	MYH9_HUMAN	P35579	MYH9	226.4	52	92	1381	Yes
Moesin	MOES_HUMAN	P26038	MSN	67.8	65	46	1373	Yes
Aspartate aminotransferase, cytoplasmic	AATC_HUMAN	P17174	GOT1	46.2	77	33	1372	Yes
Alpha-aminoadipic semialdehyde dehydrogenase	AL7A1_HUMAN	P49419	ALDH7A1	58.5	63	30	1361	Yes

Acyl-coenzyme A thioesterase 1	ACOT1_HUMAN	Q86TX2	ACOT1	46.2	72	2	1359	Yes
Alpha-1-antitrypsin	A1AT_HUMAN	P01009	SERPINA1	46.7	65	36	1353	Yes
Propionyl-CoA carboxylase alpha chain, mitochondrial	PCCA_HUMAN	P05165	PCCA	80	66	52	1348	Yes
Ubiquinone biosynthesis protein COQ9, mitochondrial	COQ9_HUMAN	O75208	COQ9	35.5	58	18	1342	Yes
Immunoglobulin heavy constant gamma 2	IGHG2_HUMAN	P01859	IGHG2	35.9	58	9	1338	Yes
Tubulin beta chain	TBB5_HUMAN	P07437	TUBB	49.6	72	4	1338	Yes
Triosephosphate isomerase	TPIS_HUMAN	P60174	TPI1	30.8	80	24	1318	Yes
Calsequestrin-2	CASQ2_HUMAN	O14958	CASQ2	46.4	57	27	1310	Yes
14-3-3 protein zeta/delta	1433Z_HUMAN	P63104	YWHAZ	27.7	64	18	1305	Yes
Hydroxysteroid dehydrogenase-like protein 2	HSDL2_HUMAN	Q6YN16	HSDL2	45.4	69	35	1303	Yes
Immunoglobulin heavy constant gamma 3	IGHG3_HUMAN	P01860	IGHG3	41.3	56	7	1301	Yes
Beta-enolase	ENOB_HUMAN	P13929	ENO3	47	60	29	1290	Yes
ATP synthase subunit O, mitochondrial	ATPO_HUMAN	P48047	ATP5PO	23.3	80	21	1288	Yes
Apolipoprotein A-IV	APOA4_HUMAN	P06727	APOA4	45.4	82	41	1287	Yes
ATP synthase subunit d, mitochondrial	ATP5H_HUMAN	O75947	ATP5PD	18.5	93	20	1251	Yes
ATP-dependent 6-phosphofructokinase, muscle type	PFKAM_HUMAN	P08237	PFKM	85.1	47	34	1251	Yes
Dihydrolipoyllysine-residue acetyltransferase component of pyruvate dehydrogenase complex, mitochondrial	ODP2_HUMAN	P10515	DLAT	69	48	31	1250	Yes
Immunoglobulin gamma-1 heavy chain	IGG1_HUMAN	P0DOX5	N/A	49.3	50	10	1248	Yes
Calcium-binding mitochondrial carrier protein Aralar1	CMC1_HUMAN	O75746	SLC25A12	74.7	58	29	1246	Yes
Cytochrome b-c1 complex subunit 7	QCR7_HUMAN	P14927	UQCRB	13.5	89	19	1244	Yes
Cytochrome c oxidase subunit 5A, mitochondrial	COX5A_HUMAN	P20674	COX5A	16.8	61	13	1233	Yes
Acyl-coenzyme A thioesterase 2, mitochondrial	ACOT2_HUMAN	P49753	ACOT2	53.2	67	2	1220	Yes
Myosin light chain 4	MYL4_HUMAN	P12829	MYL4	21.6	93	15	1218	Yes
Plakophilin-2	PKP2_HUMAN	Q99959	PKP2	97.4	57	48	1217	Yes
Spectrin beta chain, erythrocytic	SPTB1_HUMAN	P11277	SPTB	246.3	52	83	1213	Yes
Caveolae-associated protein 1	CAVN1_HUMAN	Q6NZI2	CAVIN1	43.5	44	24	1212	Yes
Pyruvate dehydrogenase protein X component, mitochondrial	ODPX_HUMAN	O00330	PDHX	54.1	53	29	1194	Yes
Phosphoglycerate mutase 2	PGAM2_HUMAN	P15259	PGAM2	28.7	77	20	1192	Yes
4-trimethylaminobutyraldehyde dehydrogenase	AL9A1_HUMAN	P49189	ALDH9A1	53.8	50	27	1191	Yes
Glycogen [starch] synthase, muscle	GYS1_HUMAN	P13807	GYS1	83.7	56	42	1187	Yes
ATP synthase subunit delta, mitochondrial	ATPD_HUMAN	P30049	ATP5F1D	17.5	44	5	1187	Yes
Voltage-dependent calcium channel subunit alpha-2/delta-1	CA2D1_HUMAN	P54289	CACNA2D1	124.5	40	43	1183	Yes
Fructose-bisphosphate aldolase C	ALDOC_HUMAN	P09972	ALDOC	39.4	86	26	1181	Yes
Hemoglobin subunit beta	HBB_HUMAN	P68871	HBB	16	95	10	1181	Yes
D-glutamate cyclase, mitochondrial	GLUCM_HUMAN	Q7Z3D6	DGLUCY	66.4	52	31	1172	Yes

Cadherin-2	CADH2_HUMAN	P19022	CDH2	99.7	35	27	1171	Yes
Dihydrolipoyllysine-residue succinyltransferase component of 2-oxoglutarate dehydrogenase complex, mitochondrial	ODO2_HUMAN	P36957	DLST	48.7	40	22	1170	Yes
Microtubule-associated protein 4	MAP4_HUMAN	P27816	MAP4	120.9	52	58	1169	Yes
Caldesmon	CALD1_HUMAN	Q05682	CALD1	93.2	55	55	1153	Yes
NADH dehydrogenase [ubiquinone] iron-sulfur protein 2, mitochondrial	NDUS2_HUMAN	O75306	NDUFS2	52.5	66	27	1149	Yes
Acid ceramidase	ASAH1_HUMAN	Q13510	ASAH1	44.6	58	27	1130	Yes
Calnexin	CALX_HUMAN	P27824	CANX	67.5	42	34	1128	Yes
14-3-3 protein beta/alpha	1433B_HUMAN	P31946	YWHAB	28.1	64	9	1120	Yes
Galectin-1	LEG1_HUMAN	P09382	LGALS1	14.7	82	14	1108	Yes
Lipoamide acyltransferase component of branched-chain alpha-keto acid dehydrogenase complex, mitochondrial	ODB2_HUMAN	P11182	DBT	53.5	56	31	1105	Yes
Tubulin beta-2A chain	TBB2A_HUMAN	Q13885	TUBB2A	49.9	74	5	1103	Yes
Protein disulfide-isomerase A4	PDIA4_HUMAN	P13667	PDIA4	72.9	57	46	1102	Yes
Lon protease homolog, mitochondrial	LONM_HUMAN	P36776	LONP1	106.4	42	44	1101	Yes
Myosin light chain 1/3, skeletal muscle isoform	MYL1_HUMAN	P05976	MYL1	21.1	47	4	1095	Yes
Glutamate dehydrogenase 2, mitochondrial	DHE4_HUMAN	P49448	GLUD2	61.4	48	3	1094	No
Keratin, type I cytoskeletal 9	K1C9_HUMAN	P35527	KRT9	62	65	36	1079	Yes
Haptoglobin	HPT_HUMAN	P00738	HP	45.2	59	13	1078	Yes
3-hydroxyisobutyrate dehydrogenase, mitochondrial	3HIDH_HUMAN	P31937	HIBADH	35.3	62	23	1078	Yes
Lamin-B2	LMNB2_HUMAN	Q03252	LMNB2	69.9	64	45	1077	Yes
Cytochrome c oxidase subunit 6B1	CX6B1_HUMAN	P14854	COX6B1	10.2	84	10	1074	Yes
Enoyl-CoA delta isomerase 2, mitochondrial	ECI2_HUMAN	O75521	ECI2	43.6	59	24	1072	Yes
WD repeat-containing protein 1	WDR1_HUMAN	O75083	WDR1	66.2	69	36	1071	Yes
GTP:AMP phosphotransferase AK3, mitochondrial	KAD3_HUMAN	Q9UIJ7	AK3	25.6	83	24	1069	Yes
Ribosome-binding protein 1	RRBP1_HUMAN	Q9P2E9	RRBP1	152.4	65	71	1068	Yes
NADH dehydrogenase [ubiquinone] iron-sulfur protein 3, mitochondrial	NDUS3_HUMAN	O75489	NDUFS3	30.2	66	26	1068	Yes
Prolow-density lipoprotein receptor-related protein 1	LRP1_HUMAN	Q07954	LRP1	504.3	23	82	1068	Yes
Prolargin	PRELP_HUMAN	P51888	PRELP	43.8	52	21	1058	Yes
Four and a half LIM domains protein 2	FHL2_HUMAN	Q14192	FHL2	32.2	92	30	1055	Yes
NADH dehydrogenase [ubiquinone] flavoprotein 2, mitochondrial	NDUV2_HUMAN	P19404	NDUFV2	27.4	63	17	1052	Yes
Sodium/potassium-transporting ATPase subunit alpha-3	AT1A3_HUMAN	P13637	ATP1A3	111.7	45	25	1048	Yes
Phosphoglycerate mutase 1	PGAM1_HUMAN	P18669	PGAM1	28.8	79	15	1043	Yes
Myosin light polypeptide 6	MYL6_HUMAN	P60660	MYL6	16.9	67	7	1043	Yes

Protein kinase C and casein kinase substrate in neurons protein 3	PACN3_HUMAN	Q9UKS6	PACSIN3	48.5	76	30	1041	Yes
Protein phosphatase 1 regulatory subunit 12B	MYPT2_HUMAN	O60237	PPP1R12B	110.3	44	45	1040	Yes
Annexin A5	ANXA5_HUMAN	P08758	ANXA5	35.9	67	27	1035	Yes
Sodium/potassium-transporting ATPase subunit alpha-2	AT1A2_HUMAN	P50993	ATP1A2	112.2	47	32	1033	Yes
Glucosidase 2 subunit beta	GLU2B_HUMAN	P14314	PRKCSH	59.4	42	31	1032	Yes
Tubulin beta-4A chain	TBB4A_HUMAN	P04350	TUBB4A	49.6	64	3	1030	No
Delta-1-pyrroline-5-carboxylate dehydrogenase, mitochondrial	AL4A1_HUMAN	P30038	ALDH4A1	61.7	53	26	1025	Yes
Cadherin-13	CAD13_HUMAN	P55290	CDH13	78.2	35	22	1025	Yes
Catenin alpha-3	CTNA3_HUMAN	Q9UI47	CTNNA3	99.7	48	46	1020	Yes
cAMP-dependent protein kinase type II-alpha regulatory subunit	KAP2_HUMAN	P13861	PRKAR2A	45.5	75	28	1019	Yes
UTP--glucose-1-phosphate uridylyltransferase	UGPA_HUMAN	Q16851	UGP2	56.9	67	38	1014	Yes
Fibrinogen alpha chain	FIBA_HUMAN	P02671	FGA	94.9	42	41	1005	Yes
Tropomodulin-1	TMOD1_HUMAN	P28289	TMOD1	40.5	74	28	1003	Yes
Dynactin subunit 2	DCTN2_HUMAN	Q13561	DCTN2	44.2	59	25	996	Yes
Thioredoxin-dependent peroxide reductase, mitochondrial	PRDX3_HUMAN	P30048	PRDX3	27.7	61	17	991	Yes
Tubulin alpha-1A chain	TBA1A_HUMAN	Q71U36	TUBA1A	50.1	59	3	991	Yes
DNA damage-binding protein 1	DDB1_HUMAN	Q16531	DDB1	126.9	46	48	984	Yes
Alpha-2-macroglobulin	A2MG_HUMAN	P01023	A2M	163.2	47	58	982	Yes
Stress-induced-phosphoprotein 1	STIP1_HUMAN	P31948	STIP1	62.6	63	48	979	Yes
Protein Niban	NIBA1_HUMAN	Q9BZQ8	NIBAN1	103.1	44	36	973	Yes
ATP synthase subunit gamma, mitochondrial	ATPG_HUMAN	P36542	ATP5F1C	33	41	19	968	Yes
Myomesin-3	MYOM3_HUMAN	Q5VTT5	MYOM3	162.1	48	60	968	Yes
MICOS complex subunit MIC19	MIC19_HUMAN	Q9NX63	CHCHD3	26.1	69	24	968	Yes
Peptidyl-prolyl cis-trans isomerase A	PPIA_HUMAN	P62937	PPIA	18	82	15	962	Yes
Dihydropyrimidinase-related protein 2	DPYL2_HUMAN	Q16555	DPYSL2	62.3	66	26	961	Yes
cAMP-dependent protein kinase type I-alpha regulatory subunit	KAP0_HUMAN	P10644	PRKAR1A	43	59	22	958	Yes
Clathrin heavy chain 1	CLH1_HUMAN	Q00610	CLTC	191.5	41	62	958	Yes
T-complex protein 1 subunit theta	TCPQ_HUMAN	P50990	CCT8	59.6	70	42	957	Yes
Fibrinogen beta chain	FIBB_HUMAN	P02675	FGB	55.9	67	39	955	Yes
Propionyl-CoA carboxylase beta chain, mitochondrial	PCCB_HUMAN	P05166	PCCB	58.2	60	28	955	Yes
Tubulin alpha-4A chain	TBA4A_HUMAN	P68366	TUBA4A	49.9	59	7	952	Yes
10 kDa heat shock protein, mitochondrial	CH10_HUMAN	P61604	HSPE1	10.9	93	23	950	Yes
ATP synthase F(0) complex subunit B1, mitochondrial	AT5F1_HUMAN	P24539	ATP5PB	28.9	48	21	943	Yes
Nidogen-1	NID1_HUMAN	P14543	NID1	136.3	36	37	935	Yes
Phosphatidylethanolamine-binding protein 1	PEBP1_HUMAN	P30086	PEBP1	21	89	17	934	Yes
Cytochrome c oxidase subunit 5B, mitochondrial	COX5B_HUMAN	P10606	COX5B	13.7	75	14	933	Yes

Cytosol aminopeptidase	AMPL_HUMAN	P28838	LAP3	56.1	67	31	928	Yes
Phosphoglucomutase-1	PGM1_HUMAN	P36871	PGM1	61.4	49	34	928	Yes
Presequence protease, mitochondrial	PREP_HUMAN	Q5JRX3	PITRM1	117.3	53	46	928	Yes
Methylcrotonoyl-CoA carboxylase subunit alpha, mitochondrial	MCCA_HUMAN	Q96RQ3	MCCC1	80.4	56	34	926	Yes
Elongation factor Tu, mitochondrial	EFTU_HUMAN	P49411	TUFM	49.5	69	28	925	Yes
Filamin-B	FLNB_HUMAN	O75369	FLNB	278	38	65	918	Yes
Annexin A11	ANX11_HUMAN	P50995	ANXA11	54.4	46	29	916	Yes
Synaptopodin 2-like protein	SYP2L_HUMAN	Q9H987	SYNPO2L	102.4	51	32	915	Yes
Nexilin	NEXN_HUMAN	Q0ZGT2	NEXN	80.6	53	41	910	Yes
Nucleolin	NUCL_HUMAN	P19338	NCL	76.6	52	48	907	Yes
Fibronectin	FINC_HUMAN	P02751	FN1	262.5	35	63	900	Yes
UDP-glucose:glycoprotein glucosyltransferase 1	UGGG1_HUMAN	Q9NYU2	UGGT1	177.1	37	46	899	Yes
Nascent polypeptide-associated complex subunit alpha, muscle-specific form	NACAM_HUMAN	E9PAV3	NACA	205.3	50	62	898	Yes
Histone H4	H4_HUMAN	P62805	H4C1	11.4	59	14	895	Yes
Decorin	PGS2_HUMAN	P07585	DCN	39.7	48	24	891	Yes
Mitochondrial proton/calcium exchanger protein	LETM1_HUMAN	O95202	LETM1	83.3	39	34	888	Yes
Elongation factor 1-alpha 2	EF1A2_HUMAN	Q05639	EEF1A2	50.4	64	18	887	Yes
Sodium/potassium-transporting ATPase subunit alpha-1	AT1A1_HUMAN	P05023	ATP1A1	112.8	38	20	882	Yes
Ubiquitin-like modifier-activating enzyme 1	UBA1_HUMAN	P22314	UBA1	117.8	42	40	876	Yes
Prenylcysteine oxidase 1	PCYOX_HUMAN	Q9UHG3	PCYOX1	56.6	48	22	874	Yes
Peroxiredoxin-2	PRDX2_HUMAN	P32119	PRDX2	21.9	74	18	871	Yes
ADP/ATP translocase 2	ADT2_HUMAN	P05141	SLC25A5	32.8	50	5	870	Yes
Elongation factor 2	EF2_HUMAN	P13639	EEF2	95.3	51	41	870	Yes
Isoleucine--tRNA ligase, mitochondrial	SYIM_HUMAN	Q9NSE4	IARS2	113.7	42	39	869	Yes
Proteasome subunit alpha type-1	PSA1_HUMAN	P25786	PSMA1	29.5	92	27	867	Yes
Adenylate kinase 2, mitochondrial	KAD2_HUMAN	P54819	AK2	26.5	72	18	866	Yes
Prostaglandin E synthase 2	PGES2_HUMAN	Q9H7Z7	PTGES2	41.9	69	28	865	Yes
PDZ and LIM domain protein 5	PDLI5_HUMAN	Q96HC4	PDLIM5	63.9	49	29	858	Yes
Phosphoglucomutase-like protein 5	PGM5_HUMAN	Q15124	PGM5	62.2	58	30	853	Yes
Prohibitin	PHB_HUMAN	P35232	PHB	29.8	82	20	848	Yes
Peroxiredoxin-1	PRDX1_HUMAN	Q06830	PRDX1	22.1	80	20	846	Yes
Complement C4-B	CO4B_HUMAN	P0C0L5	C4B	192.6	38	3	842	Yes
Methylmalonyl-CoA mutase, mitochondrial	MUTA_HUMAN	P22033	MMUT	83.1	49	36	836	Yes
NADH dehydrogenase [ubiquinone] 1 alpha subcomplex subunit 10, mitochondrial	NDUAA_HUMAN	O95299	NDUFA10	40.7	47	20	831	Yes

Short-chain specific acyl-CoA dehydrogenase, mitochondrial	ACADS_HUMAN	P16219	ACADS	44.3	58	22	823	Yes
Isovaleryl-CoA dehydrogenase, mitochondrial	IVD_HUMAN	P26440	IVD	46.3	53	21	821	Yes
Ceruloplasmin	CERU_HUMAN	P00450	CP	122.1	46	37	812	Yes
PDZ and LIM domain protein 1	PDLI1_HUMAN	O00151	PDLIM1	36	88	25	806	Yes
Isocitrate dehydrogenase [NAD] subunit beta, mitochondrial	IDH3B_HUMAN	O43837	IDH3B	42.2	59	25	793	Yes
Dynactin subunit 1	DCTN1_HUMAN	Q14203	DCTN1	141.6	40	46	793	Yes
Prosaposin	SAP_HUMAN	P07602	PSAP	58.1	42	23	792	Yes
T-complex protein 1 subunit beta	TCPB_HUMAN	P78371	CCT2	57.5	65	36	792	Yes
14-3-3 protein theta	1433T_HUMAN	P27348	YWHAQ	27.7	60	12	789	Yes
Protein unc-45 homolog B	UN45B_HUMAN	Q8IWX7	UNC45B	103.7	52	52	788	Yes
Nidogen-2	NID2_HUMAN	Q14112	NID2	151.2	36	40	787	Yes
Ezrin	EZRI_HUMAN	P15311	EZR	69.4	47	22	781	Yes
Calpastatin	ICAL_HUMAN	P20810	CAST	76.5	62	35	781	Yes
Prohibitin-2	PHB2_HUMAN	Q99623	PHB2	33.3	75	24	778	Yes
Immunoglobulin heavy constant alpha 1	IGHA1_HUMAN	P01876	IGHA1	37.6	57	10	774	Yes
Dolichyl-diphosphooligosaccharide--protein glycosyltransferase subunit 1	RPN1_HUMAN	P04843	RPN1	68.5	54	35	774	Yes
Voltage-dependent anion-selective channel protein 1	VDAC1_HUMAN	P21796	VDAC1	30.8	75	20	774	Yes
4-aminobutyrate aminotransferase, mitochondrial	GABT_HUMAN	P80404	ABAT	56.4	66	30	773	Yes
Cytochrome b-c1 complex subunit Rieske, mitochondrial	UCRI_HUMAN	P47985	UQCRFS1	29.6	56	19	771	Yes
14-3-3 protein eta	1433F_HUMAN	Q04917	YWHAH	28.2	57	15	768	Yes
Golgi apparatus protein 1	GSLG1_HUMAN	Q92896	GLG1	134.5	41	48	768	Yes
Hemoglobin subunit delta	HBD_HUMAN	P02042	HBD	16	95	7	766	Yes
Succinate-semialdehyde dehydrogenase, mitochondrial	SSDH_HUMAN	P51649	ALDH5A1	57.2	49	30	766	Yes
Palmdelphin	PALMD_HUMAN	Q9NP74	PALMD	62.7	60	39	763	Yes
Tensin-1	TENS1_HUMAN	Q9HBL0	TNS1	185.6	42	47	762	Yes
Transcription factor A, mitochondrial	TFAM_HUMAN	Q00059	TFAM	29.1	58	22	760	Yes
Integrin beta-1	ITB1_HUMAN	P05556	ITGB1	88.4	38	31	759	Yes
Thioredoxin reductase 2, mitochondrial	TRXR2_HUMAN	Q9NNW7	TXNRD2	56.5	56	24	759	Yes
Hypoxia up-regulated protein 1	HYOU1_HUMAN	Q9Y4L1	HYOU1	111.3	45	40	756	Yes
NADH dehydrogenase [ubiquinone] 1 alpha subcomplex subunit 9, mitochondrial	NDUA9_HUMAN	Q16795	NDUFA9	42.5	54	24	755	Yes
Immunoglobulin lambda constant 2	IGLC2_HUMAN	P0DOY2	IGLC2	11.3	67	6	754	Yes
Cytochrome c	CYC_HUMAN	P99999	CYCS	11.7	85	19	754	Yes
NAD-dependent malic enzyme, mitochondrial	MAOM_HUMAN	P23368	ME2	65.4	57	28	752	Yes
Latent-transforming growth factor beta-binding protein 2	LTBP2_HUMAN	Q14767	LTBP2	194.9	24	37	751	Yes

Annexin A1	ANXA1_HUMAN	P04083	ANXA1	38.7	73	26	747	Yes
Ribonuclease inhibitor	RINI_HUMAN	P13489	RNH1	49.9	62	25	741	Yes
Cytoplasmic aconitate hydratase	ACOC_HUMAN	P21399	ACO1	98.3	54	42	739	Yes
Immunoglobulin heavy constant gamma 4	IGHG4_HUMAN	P01861	IGHG4	35.9	61	9	738	Yes
Branched-chain-amino-acid aminotransferase, mitochondrial	BCAT2_HUMAN	O15382	BCAT2	44.3	58	21	736	Yes
Unconventional myosin-Ic	MYO1C_HUMAN	O00159	MYO1C	121.6	46	48	734	Yes
Protein disulfide-isomerase A6	PDIA6_HUMAN	Q15084	PDIA6	48.1	48	20	734	Yes
Heat shock protein beta-7	HSPB7_HUMAN	Q9UBY9	HSPB7	18.6	88	12	734	Yes
Succinate--CoA ligase [ADP/GDP-forming] subunit alpha, mitochondrial	SUCA_HUMAN	P53597	SUCLG1	36.2	47	22	731	Yes
Vitamin D-binding protein	VTDB_HUMAN	P02774	GC	52.9	65	28	717	Yes
Glycogenin-1	GLYG_HUMAN	P46976	GYG1	39.4	25	14	717	Yes
EH domain-containing protein 2	EHD2_HUMAN	Q9NZN4	EHD2	61.1	69	34	716	Yes
Heterogeneous nuclear ribonucleoproteins A2/B1	ROA2_HUMAN	P22626	HNRNPA2B1	37.4	62	21	710	Yes
LIM domain only protein 7	LMO7_HUMAN	Q8WWI1	LMO7	192.6	30	47	710	Yes
Probable D-lactate dehydrogenase, mitochondrial	LDHD_HUMAN	Q86WU2	LDHD	54.8	75	29	708	Yes
Nucleobindin-1	NUCB1_HUMAN	Q02818	NUCB1	53.8	70	42	706	Yes
Acetyl-coenzyme A synthetase 2-like, mitochondrial	ACS2L_HUMAN	Q9NUB1	ACSS1	74.8	43	25	698	Yes
Enoyl-CoA delta isomerase 1, mitochondrial	ECI1_HUMAN	P42126	ECI1	32.8	49	12	696	Yes
L-lactate dehydrogenase A chain	LDHA_HUMAN	P00338	LDHA	36.7	60	20	692	Yes
Glycerol-3-phosphate dehydrogenase 1-like protein	GPD1L_HUMAN	Q8N335	GPD1L	38.4	58	22	691	Yes
Apolipoprotein E	APOE_HUMAN	P02649	APOE	36.1	68	26	690	Yes
Histone H2B type 1-C/E/F/G/I	H2B1C_HUMAN	P62807	H2BC10	13.9	72	3	688	Yes
Hemoglobin subunit alpha	HBA_HUMAN	P69905	HBA1/HBA2	15.2	98	14	688	Yes
Protein/nucleic acid deglycase DJ-1	PARK7_HUMAN	Q99497	PARK7	19.9	60	17	681	Yes
2-oxoglutarate dehydrogenase-like, mitochondrial	OGDHL_HUMAN	Q9ULD0	OGDHL	114.4	22	10	680	Yes
Adenylate kinase isoenzyme 1	KAD1_HUMAN	P00568	AK1	21.6	84	23	672	Yes
Serine/threonine-protein phosphatase 2A 65 kDa regulatory subunit A alpha isoform	2AAA_HUMAN	P30153	PPP2R1A	65.3	51	27	671	Yes
Superoxide dismutase [Cu-Zn]	SODC_HUMAN	P00441	SOD1	15.9	97	16	668	Yes
T-complex protein 1 subunit gamma	TCPG_HUMAN	P49368	CCT3	60.5	54	29	665	Yes
Catalase	CATA_HUMAN	P04040	CAT	59.7	59	30	664	Yes
Peptidyl-prolyl cis-trans isomerase F, mitochondrial	PPIF_HUMAN	P30405	PPIF	22	73	15	664	Yes
Adenylyl cyclase-associated protein 2	CAP2_HUMAN	P40123	CAP2	52.8	52	22	664	Yes
Annexin A7	ANXA7_HUMAN	P20073	ANXA7	52.7	38	17	659	Yes
Myosin regulatory light polypeptide 9	MYL9_HUMAN	P24844	MYL9	19.8	71	7	657	Yes

Fibrinogen gamma chain	FIBG_HUMAN	P02679	FGG	51.5	50	26	653	Yes
Protein-L-isoaspartate(D-aspartate) O-methyltransferase	PIMT_HUMAN	P22061	PCMT1	24.6	74	17	653	Yes
EH domain-containing protein 4	EHD4_HUMAN	Q9H223	EHD4	61.1	64	22	653	Yes
Clusterin	CLUS_HUMAN	P10909	CLU	52.5	39	22	651	Yes
Protein NipSnap homolog 2	NIPS2_HUMAN	O75323	NIPSNAP2	33.7	66	15	649	Yes
Keratin, type II cytoskeletal 6A	K2C6A_HUMAN	P02538	KRT6A	60	55	3	649	Yes
Ferrochelatase, mitochondrial	HEMH_HUMAN	P22830	FECH	47.8	60	24	649	Yes
Adenylyl cyclase-associated protein 1	CAP1_HUMAN	Q01518	CAP1	51.9	55	30	649	Yes
Cytoplasmic dynein 1 heavy chain 1	DYHC1_HUMAN	Q14204	DYNC1H1	532.1	19	74	648	Yes
Asporin	ASPN_HUMAN	Q9BXN1	ASPN	43.4	66	26	646	Yes
Ubiquitin-40S ribosomal protein S27a	RS27A_HUMAN	P62979	RPS27A	18	38	11	643	Yes
Mitochondrial-processing peptidase subunit alpha	MPPA_HUMAN	Q10713	PMPCA	58.2	49	24	642	Yes
Voltage-dependent anion-selective channel protein 2	VDAC2_HUMAN	P45880	VDAC2	31.5	66	18	633	Yes
Thrombospondin-4	TSP4_HUMAN	P35443	THBS4	105.8	34	22	631	Yes
Short/branched chain specific acyl-CoA dehydrogenase, mitochondrial	ACDSB_HUMAN	P45954	ACADSB	47.5	50	20	630	Yes
Carnitine O-acetyltransferase	CACP_HUMAN	P43155	CRAT	70.8	36	21	629	Yes
Cofilin-2	COF2_HUMAN	Q9Y281	CFL2	18.7	78	13	628	Yes
Keratin, type I cytoskeletal 16	K1C16_HUMAN	P08779	KRT16	51.2	69	20	627	Yes
Cysteine and glycine-rich protein 3	CSRP3_HUMAN	P50461	CSRP3	21	75	15	626	Yes
Hydroxyacid-oxoacid transhydrogenase, mitochondrial	HOT_HUMAN	Q8IWW8	ADHFE1	50.3	64	22	626	Yes
Tubulin alpha-8 chain	TBA8_HUMAN	Q9NY65	TUBA8	50.1	53	7	624	Yes
Synaptopodin-2	SYNP2_HUMAN	Q9UMS6	SYNPO2	117.4	46	41	622	Yes
Immunoglobulin kappa constant	IGKC_HUMAN	P01834	IGKC	11.8	91	2	620	Yes
Histone H1.4	H14_HUMAN	P10412	H1-4	21.9	43	7	620	Yes
Myosin regulatory light chain 12B	ML12B_HUMAN	O14950	MYL12B	19.8	71	7	618	Yes
Pyruvate dehydrogenase phosphatase regulatory subunit, mitochondrial	PDPR_HUMAN	Q8NCN5	PDPR	99.3	39	33	618	Yes
Keratin, type II cytoskeletal 6B	K2C6B_HUMAN	P04259	KRT6B	60	53	3	617	No
Polyadenylate-binding protein 4	PABP4_HUMAN	Q13310	PABPC4	70.7	41	22	617	Yes
Sulfite oxidase, mitochondrial	SUOX_HUMAN	P51687	SUOX	60.2	56	24	612	Yes
Nebulin-related-anchoring protein	NRAP_HUMAN	Q86VF7	NRAP	197	45	72	595	Yes
Glypican-1	GPC1_HUMAN	P35052	GPC1	61.6	47	22	594	Yes
Serpin H1	SERPH_HUMAN	P50454	SERPINH1	46.4	61	26	593	Yes
Septin-7	SEPT7_HUMAN	Q16181	SEPTIN7	50.6	52	25	592	Yes
Thioredoxin domain-containing protein 5	TXND5_HUMAN	Q8NBS9	TXNDC5	47.6	48	18	591	Yes

Acyl-coenzyme A thioesterase 9, mitochondrial	ACOT9_HUMAN	Q9Y305	ACOT9	49.9	53	25	590	Yes
Receptor expression-enhancing protein 5	REEP5_HUMAN	Q00765	REEP5	21.5	31	11	589	Yes
2-oxoisovalerate dehydrogenase subunit alpha, mitochondrial	ODBA_HUMAN	P12694	BCKDHA	50.4	49	22	587	Yes
Heat shock protein 75 kDa, mitochondrial	TRAP1_HUMAN	Q12931	TRAP1	80.1	44	26	585	Yes
Synaptopodin	SYNPO_HUMAN	Q8N3V7	SYNPO	99.4	39	26	585	Yes
Isocitrate dehydrogenase [NAD] subunit gamma, mitochondrial	IDH3G_HUMAN	P51553	IDH3G	42.8	54	17	579	Yes
1,4-alpha-glucan-branching enzyme	GLGB_HUMAN	Q04446	GBE1	80.4	44	27	578	Yes
Elongation factor 1-gamma	EF1G_HUMAN	P26641	EEF1G	50.1	39	21	577	Yes
Keratin, type II cytoskeletal 5	K2C5_HUMAN	P13647	KRT5	62.3	51	22	576	Yes
Succinate dehydrogenase [ubiquinone] iron-sulfur subunit, mitochondrial	SDHB_HUMAN	P21912	SDHB	31.6	58	23	574	Yes
Integrin alpha-7	ITA7_HUMAN	Q13683	ITGA7	128.9	35	35	574	Yes
Keratin, type I cytoskeletal 14	K1C14_HUMAN	P02533	KRT14	51.5	57	8	573	No
NADH dehydrogenase [ubiquinone] 1 alpha subcomplex subunit 7	NDUA7_HUMAN	O95182	NDUFA7	12.5	84	15	569	Yes
von Willebrand factor	VWF_HUMAN	P04275	VWF	309.1	21	51	569	Yes
ATPase inhibitor, mitochondrial	ATIF1_HUMAN	Q9UII2	ATP5IF1	12.2	40	13	569	Yes
Fatty acid-binding protein 5	FABP5_HUMAN	Q01469	FABP5	15.2	75	17	568	Yes
Actin-binding LIM protein 1	ABLM1_HUMAN	O14639	ABLIM1	87.6	54	35	566	Yes
BAG family molecular chaperone regulator 3	BAG3_HUMAN	O95817	BAG3	61.6	71	28	564	Yes
Kinesin-1 heavy chain	KINH_HUMAN	P33176	KIF5B	109.6	54	46	563	Yes
26S proteasome non-ATPase regulatory subunit 1	PSMD1_HUMAN	Q99460	PSMD1	105.8	38	31	563	Yes
Isobutyryl-CoA dehydrogenase, mitochondrial	ACAD8_HUMAN	Q9UKU7	ACAD8	45	50	23	563	Yes
Leiomodin-2	LMOD2_HUMAN	Q6P5Q4	LMOD2	61.6	56	28	558	Yes
3-hydroxyacyl-CoA dehydrogenase type-2	HCD2_HUMAN	Q99714	HSD17B10	26.9	80	17	555	Yes
Galectin-3-binding protein	LG3BP_HUMAN	Q08380	LGALS3BP	65.3	41	21	554	Yes
Agrin	AGRIN_HUMAN	O00468	AGRN	217.1	33	48	552	Yes
Caveolae-associated protein 2	CAVN2_HUMAN	O95810	CAVIN2	47.1	49	23	552	Yes
Cardiomyopathy-associated protein 5	CMYA5_HUMAN	Q8N3K9	CMYA5	448.9	25	73	551	Yes
26S proteasome non-ATPase regulatory subunit 2	PSMD2_HUMAN	Q13200	PSMD2	100.1	40	28	549	Yes
Liver carboxylesterase 1	EST1_HUMAN	P23141	CES1	62.5	45	22	548	Yes
Adenylate kinase 4, mitochondrial	KAD4_HUMAN	P27144	AK4	25.3	78	17	548	Yes
Aldehyde dehydrogenase X, mitochondrial	AL1B1_HUMAN	P30837	ALDH1B1	57.2	54	23	548	Yes
Hemopexin	HEMO_HUMAN	P02790	HPX	51.6	54	22	547	Yes
Histone-lysine N-methyltransferase SMYD1	SMYD1_HUMAN	Q8NB12	SMYD1	56.6	56	34	543	Yes
Mitochondrial-processing peptidase subunit beta	MPPB_HUMAN	O75439	PMPCB	54.3	39	18	542	Yes
Kininogen-1	KNG1_HUMAN	P01042	KNG1	71.9	41	27	541	Yes

Hsc70-interacting protein	F10A1_HUMAN	P50502	ST13	41.3	38	17	541	Yes
Heterogeneous nuclear ribonucleoprotein K	HNRPK_HUMAN	P61978	HNRNPK	50.9	47	21	540	Yes
3-hydroxyisobutyryl-CoA hydrolase, mitochondrial	HIBCH_HUMAN	Q6NVY1	HIBCH	43.5	51	21	540	Yes
Ankyrin-3	ANK3_HUMAN	Q12955	ANK3	480.1	13	38	539	Yes
Reticulon-4	RTN4_HUMAN	Q9NQC3	RTN4	129.9	41	34	539	Yes
Antithrombin-III	ANT3_HUMAN	P01008	SERPINC1	52.6	45	22	537	Yes
T-complex protein 1 subunit epsilon	TCPE_HUMAN	P48643	CCT5	59.6	60	28	536	Yes
Ketimine reductase mu-crystallin	CRYM_HUMAN	Q14894	CRYM	33.8	49	14	536	Yes
Glutathione S-transferase kappa 1	GSTK1_HUMAN	Q9Y2Q3	GSTK1	25.5	62	14	535	Yes
Calmodulin-1	CALM1_HUMAN	P0DP23	CALM1	16.8	79	11	532	Yes
Ferritin heavy chain	FRIH_HUMAN	P02794	FTH1	21.2	72	16	529	Yes
ATP-dependent 6-phosphofructokinase, platelet type	PFKAP_HUMAN	Q01813	PFKP	85.5	45	32	526	Yes
Quinone oxidoreductase	QOR_HUMAN	Q08257	CRYZ	35.2	73	22	521	Yes
Haptoglobin-related protein	HPTR_HUMAN	P00739	HPR	39	37	2	519	No
Cytochrome b-c1 complex subunit 6, mitochondrial	QCR6_HUMAN	P07919	UQCRH	10.7	89	8	519	Yes
D-beta-hydroxybutyrate dehydrogenase, mitochondrial	BDH_HUMAN	Q02338	BDH1	38.1	65	23	519	Yes
Isocitrate dehydrogenase [NADP] cytoplasmic	IDHC_HUMAN	O75874	IDH1	46.6	54	20	518	Yes
Clathrin light chain B	CLCB_HUMAN	P09497	CLTB	25.2	33	14	518	Yes
Serpin B6	SPB6_HUMAN	P35237	SERPINB6	42.6	62	21	517	Yes
Transgelin	TAGL_HUMAN	Q01995	TAGLN	22.6	88	21	517	Yes
Sulfide:quinone oxidoreductase, mitochondrial	SQOR_HUMAN	Q9Y6N5	SQOR	49.9	59	24	517	Yes
Delta-sarcoglycan	SGCD_HUMAN	Q92629	SGCD	32.1	46	12	516	Yes
Immunoglobulin lambda-1 light chain	IGL1_HUMAN	P0DOX8	N/A	22.8	43	5	514	Yes
Aldo-keto reductase family 1 member B1	ALDR_HUMAN	P15121	AKR1B1	35.8	61	16	514	Yes
Amine oxidase [flavin-containing] B	AOFB_HUMAN	P27338	MAOB	58.7	41	18	514	Yes
28S ribosomal protein S36, mitochondrial	RT36_HUMAN	P82909	MRPS36	11.5	74	8	513	Yes
Neural cell adhesion molecule 1	NCAM1_HUMAN	P13591	NCAM1	94.5	34	26	512	Yes
Laminin subunit alpha-4	LAMA4_HUMAN	Q16363	LAMA4	202.4	23	38	511	Yes
Profilin-1	PROF1_HUMAN	P07737	PFN1	15	79	15	509	Yes
Histone H1.2	H12_HUMAN	P16403	H1-2	21.4	39	5	507	Yes
Atypical kinase COQ8A, mitochondrial	COQ8A_HUMAN	Q8NI60	COQ8A	71.9	31	18	507	Yes
NADH dehydrogenase [ubiquinone] iron-sulfur protein 8, mitochondrial	NDUS8_HUMAN	O00217	NDUFS8	23.7	56	13	506	Yes
Cytoskeleton-associated protein 4	CKAP4_HUMAN	Q07065	CKAP4	66	68	35	505	Yes
Peroxiredoxin-4	PRDX4_HUMAN	Q13162	PRDX4	30.5	66	14	502	Yes
Dysferlin	DYSF_HUMAN	O75923	DYSF	237.1	32	48	501	Yes

Mitochondrial import receptor subunit TOM70	TOM70_HUMAN	O94826	TOMM70	67.4	51	27	501	Yes
NAD(P)H-hydrate epimerase	NNRE_HUMAN	Q8NCW5	NAXE	31.7	55	16	501	Yes
Retinal dehydrogenase 1	AL1A1_HUMAN	P00352	ALDH1A1	54.8	53	16	500	Yes
Cytoplasmic dynein 1 intermediate chain 2	DC1I2_HUMAN	Q13409	DYNC1I2	71.4	37	18	500	Yes
Myosin-10	MYH10_HUMAN	P35580	MYH10	228.9	34	44	495	Yes
Histone H2A type 2-C	H2A2C_HUMAN	Q16777	H2AC20	14	46	3	495	Yes
Heat shock protein beta-6	HSPB6_HUMAN	O14558	HSPB6	17.1	71	9	494	Yes
26S proteasome regulatory subunit 6A	PRS6A_HUMAN	P17980	PSMC3	49.2	65	26	492	Yes
Alpha-centractin	ACTZ_HUMAN	P61163	ACTR1A	42.6	60	11	489	Yes
Guanine nucleotide-binding protein G(i) subunit alpha-2	GNAI2_HUMAN	P04899	GNAI2	40.4	63	13	486	Yes
Peroxiredoxin-5, mitochondrial	PRDX5_HUMAN	P30044	PRDX5	22.1	47	12	486	Yes
Ankyrin repeat domain-containing protein 1	ANKR1_HUMAN	Q15327	ANKRD1	36.2	56	18	486	Yes
Ras suppressor protein 1	RSU1_HUMAN	Q15404	RSU1	31.5	54	16	484	Yes
Beta-sarcoglycan	SGCB_HUMAN	Q16585	SGCB	34.8	54	16	483	Yes
NADH dehydrogenase [ubiquinone] 1 alpha subcomplex subunit 2	NDUA2_HUMAN	O43678	NDUFA2	10.9	65	8	482	Yes
Immunoglobulin kappa light chain	IGK_HUMAN	P0DOX7	N/A	23.4	59	3	482	Yes
Eukaryotic translation initiation factor 3 subunit A	EIF3A_HUMAN	Q14152	EIF3A	166.5	27	39	481	Yes
Protein NipSnap homolog 3B	NPS3B_HUMAN	Q9BS92	NIPSNAP3B	28.3	67	14	477	Yes
Proteasome subunit alpha type-4	PSA4_HUMAN	P25789	PSMA4	29.5	63	18	474	Yes
Protein phosphatase 1 regulatory subunit 12C	PP12C_HUMAN	Q9BZL4	PPP1R12C	84.8	47	36	473	Yes
Proteasome subunit alpha type-7	PSA7_HUMAN	O14818	PSMA7	27.9	68	17	472	Yes
Glycogen phosphorylase, liver form	PYGL_HUMAN	P06737	PYGL	97.1	18	5	470	No
Proteasome subunit alpha type-3	PSA3_HUMAN	P25788	PSMA3	28.4	53	18	470	Yes
NADH dehydrogenase [ubiquinone] 1 beta subcomplex subunit 10	NDUBA_HUMAN	O96000	NDUFB10	20.8	73	15	469	Yes
Mitofusin-2	MFN2_HUMAN	O95140	MFN2	86.3	39	30	468	Yes
Alpha-sarcoglycan	SGCA_HUMAN	Q16586	SGCA	42.8	44	14	468	Yes
L-xylulose reductase	DCXR_HUMAN	Q7Z4W1	DCXR	25.9	58	14	466	Yes
Elongation factor 1-alpha 1	EF1A1_HUMAN	P68104	EEF1A1	50.1	49	13	465	Yes
Perilipin-3	PLIN3_HUMAN	O60664	PLIN3	47	61	19	463	Yes
NADH dehydrogenase [ubiquinone] 1 alpha subcomplex subunit 12	NDUAC_HUMAN	Q9UI09	NDUFA12	17.1	84	15	461	Yes
Prothrombin	THRB_HUMAN	P00734	F2	70	49	25	460	Yes
Galectin-3	LEG3_HUMAN	P17931	LGALS3	26.1	38	10	459	Yes
Amine oxidase [flavin-containing] A	AOFA_HUMAN	P21397	MAOA	59.6	32	13	458	Yes
Dystroglycan	DAG1_HUMAN	Q14118	DAG1	97.4	24	20	458	Yes
Mycophenolic acid acyl-glucuronide esterase, mitochondrial	ABHDA_HUMAN	Q9NUJ1	ABHD10	33.9	51	15	458	Yes
Elongation factor 1-delta	EF1D_HUMAN	P29692	EEF1D	31.1	64	12	457	Yes

Phosphate carrier protein, mitochondrial	MPCP_HUMAN	Q00325	SLC25A3	40.1	37	20	457	Yes
Lysosomal alpha-glucosidase	LYAG_HUMAN	P10253	GAA	105.3	26	22	454	Yes
Polyadenylate-binding protein 1	PABP1_HUMAN	P11940	PABPC1	70.6	36	18	453	Yes
Voltage-dependent anion-selective channel protein 3	VDAC3_HUMAN	Q9Y277	VDAC3	30.6	61	14	453	Yes
Glutathione reductase, mitochondrial	GSHR_HUMAN	P00390	GSR	56.2	48	19	451	Yes
X-ray repair cross-complementing protein 6	XRCC6_HUMAN	P12956	XRCC6	69.8	42	27	451	Yes
NADH dehydrogenase [ubiquinone] 1 alpha subcomplex subunit 8	NDUA8_HUMAN	P51970	NDUFA8	20.1	76	14	451	Yes
Rho GTPase-activating protein 1	RHG01_HUMAN	Q07960	ARHGAP1	50.4	54	23	449	Yes
Glutaryl-CoA dehydrogenase, mitochondrial	GCDH_HUMAN	Q92947	GCDH	48.1	48	17	448	Yes
UV excision repair protein RAD23 homolog B	RD23B_HUMAN	P54727	RAD23B	43.1	36	14	444	Yes
Proteasome subunit alpha type-6	PSA6_HUMAN	P60900	PSMA6	27.4	62	16	441	Yes
Basigin	BASI_HUMAN	P35613	BSG	42.2	35	9	440	Yes
Basal cell adhesion molecule	BCAM_HUMAN	P50895	BCAM	67.4	53	27	440	Yes
Coagulation factor XIII A chain	F13A_HUMAN	P00488	F13A1	83.2	36	24	436	Yes
NADP-dependent malic enzyme, mitochondrial	MAON_HUMAN	Q16798	ME3	67	46	19	433	Yes
NADH dehydrogenase [ubiquinone] 1 alpha subcomplex subunit 13	NDUAD_HUMAN	Q9P0J0	NDUFA13	16.7	72	13	433	Yes
Proteasome subunit beta type-5	PSB5_HUMAN	P28074	PSMB5	28.5	63	15	432	Yes
26S proteasome regulatory subunit 8	PRS8_HUMAN	P62195	PSMC5	45.6	63	19	431	Yes
Annexin A4	ANXA4_HUMAN	P09525	ANXA4	35.9	58	22	430	Yes
Tenascin-X	TENX_HUMAN	P22105	TNXB	457.9	19	48	430	Yes
A-kinase anchor protein 12	AKA12_HUMAN	Q02952	AKAP12	191.4	32	42	430	Yes
Cofilin-1	COF1_HUMAN	P23528	CFL1	18.5	73	10	429	Yes
Inorganic pyrophosphatase 2, mitochondrial	IPYR2_HUMAN	Q9H2U2	PPA2	37.9	54	14	429	Yes
Complement factor B	CFAB_HUMAN	P00751	CFB	85.5	40	30	428	Yes
Serine/threonine-protein phosphatase 2A catalytic subunit alpha isoform	PP2AA_HUMAN	P67775	PPP2CA	35.6	67	2	428	Yes
Glyoxylate reductase/hydroxypyruvate reductase	GRHPR_HUMAN	Q9UBQ7	GRHPR	35.6	58	18	428	Yes
Rab GDP dissociation inhibitor alpha	GDIA_HUMAN	P31150	GDI1	50.6	61	14	426	Yes
Guanine nucleotide-binding protein G(I)/G(S)/G(T) subunit beta-1	GBB1_HUMAN	P62873	GNB1	37.4	45	7	425	Yes
Calcium-binding mitochondrial carrier protein SCaMC-1	SCMC1_HUMAN	Q6NUK1	SLC25A24	53.3	57	30	425	Yes
Alpha-2-macroglobulin receptor-associated protein	AMRP_HUMAN	P30533	LRPAP1	41.4	53	22	424	Yes
Programmed cell death 6-interacting protein	PDC6I_HUMAN	Q8WUM4	PDCD6IP	96	37	28	424	Yes
Peripherin	PERI_HUMAN	P41219	PRPH	53.6	26	9	420	No
Endoplasmic reticulum resident protein 29	ERP29_HUMAN	P30040	ERP29	29	52	15	419	Yes
Calcineurin B homologous protein 1	CHP1_HUMAN	Q99653	CHP1	22.4	86	14	417	Yes

NADH dehydrogenase [ubiquinone] iron-sulfur protein 4, mitochondrial	NDUS4_HUMAN	O43181	NDUFS4	20.1	34	9	416	Yes
Non-specific lipid-transfer protein	NLTP_HUMAN	P22307	SCP2	59	47	26	415	Yes
Alpha-1-syntrophin	SNTA1_HUMAN	Q13424	SNTA1	53.9	52	21	414	Yes
EH domain-containing protein 1	EHD1_HUMAN	Q9H4M9	EHD1	60.6	57	16	413	Yes
Protein NDRG2	NDRG2_HUMAN	Q9UN36	NDRG2	40.8	57	14	413	Yes
Four and a half LIM domains protein 1	FHL1_HUMAN	Q13642	FHL1	36.2	52	17	412	Yes
Cocaine esterase	EST2_HUMAN	O00748	CES2	61.8	37	18	408	Yes
Collagen alpha-1(XVIII) chain	COIA1_HUMAN	P39060	COL18A1	178.1	14	16	408	Yes
Alcohol dehydrogenase class-3	ADHX_HUMAN	P11766	ADH5	39.7	37	18	406	Yes
26S proteasome regulatory subunit 4	PRS4_HUMAN	P62191	PSMC1	49.2	52	22	406	Yes
Collagen alpha-1(XV) chain	COFA1_HUMAN	P39059	COL15A1	141.6	17	17	404	Yes
C-type mannose receptor 2	MRC2_HUMAN	Q9UBG0	MRC2	166.6	23	26	403	Yes
NADH-cytochrome b5 reductase 3	NB5R3_HUMAN	P00387	CYB5R3	34.2	75	16	402	Yes
Prolyl 4-hydroxylase subunit alpha-1	P4HA1_HUMAN	P13674	P4HA1	61	53	25	402	Yes
Cytochrome c1, heme protein, mitochondrial	CY1_HUMAN	P08574	CYC1	35.4	42	12	401	Yes
Puromycin-sensitive aminopeptidase	PSA_HUMAN	P55786	NPEPPS	103.2	35	32	401	Yes
26S proteasome regulatory subunit 10B	PRS10_HUMAN	P62333	PSMC6	44.1	69	25	401	Yes
Importin subunit beta-1	IMB1_HUMAN	Q14974	KPNB1	97.1	29	21	401	Yes
Complement component 1 Q subcomponent-binding protein, mitochondrial	C1QBP_HUMAN	Q07021	C1QBP	31.3	43	8	399	Yes
14 kDa phosphohistidine phosphatase	PHP14_HUMAN	Q9NRX4	PHPT1	13.8	70	11	399	Yes
cAMP-dependent protein kinase catalytic subunit alpha	KAPCA_HUMAN	P17612	PRKACA	40.6	43	10	398	Yes
T-complex protein 1 subunit delta	TCPD_HUMAN	P50991	CCT4	57.9	56	23	398	Yes
Torsin-1A-interacting protein 1	TOIP1_HUMAN	Q5JTV8	TOR1AIP1	66.2	46	25	398	Yes
Omega-amidase NIT2	NIT2_HUMAN	Q9NQR4	NIT2	30.6	80	22	398	Yes
Radixin	RADI_HUMAN	P35241	RDX	68.5	34	15	395	Yes
T-complex protein 1 subunit alpha	TCPA_HUMAN	P17987	TCP1	60.3	48	24	394	Yes
NADH dehydrogenase [ubiquinone] 1 alpha subcomplex subunit 5	NDUA5_HUMAN	Q16718	NDUFA5	13.5	72	9	394	Yes
2-methoxy-6-polyprenyl-1,4-benzoquinol methylase, mitochondrial	COQ5_HUMAN	Q5HYK3	COQ5	37.1	58	20	394	Yes
Calumenin	CALU_HUMAN	O43852	CALU	37.1	56	19	392	Yes
Nucleophosmin	NPM_HUMAN	P06748	NPM1	32.6	46	13	392	Yes
Endoplasmic reticulum resident protein 44	ERP44_HUMAN	Q9BS26	ERP44	46.9	46	17	392	Yes
60S acidic ribosomal protein P2	RLA2_HUMAN	P05387	RPLP2	11.7	89	7	391	Yes
Ubiquinone biosynthesis monooxygenase COQ6, mitochondrial	COQ6_HUMAN	Q9Y2Z9	COQ6	50.8	34	17	391	Yes

Ankyrin repeat domain-containing protein 2	ANKR2_HUMAN	Q9GZV1	ANKRD2	39.8	55	19	390	Yes
[Protein ADP-ribosylarginine] hydrolase-like protein 1	ARHL1_HUMAN	Q8NDY3	ADPRHL1	40.1	48	19	389	Yes
Epoxide hydrolase 1	HYEP_HUMAN	P07099	EPHX1	52.9	49	24	388	Yes
Serine/threonine-protein phosphatase 2A catalytic subunit beta isoform	PP2AB_HUMAN	P62714	PPP2CB	35.6	67	2	387	Yes
Stomatin-like protein 2, mitochondrial	STML2_HUMAN	Q9UJZ1	STOML2	38.5	38	19	387	Yes
Transketolase	TKT_HUMAN	P29401	TKT	67.8	43	23	386	Yes
26S proteasome regulatory subunit 7	PRS7_HUMAN	P35998	PSMC2	48.6	58	24	386	Yes
Guanine nucleotide-binding protein G(I)/G(S)/G(T) subunit beta-2	GBB2_HUMAN	P62879	GNB2	37.3	50	5	385	Yes
40S ribosomal protein S3a	RS3A_HUMAN	P61247	RPS3A	29.9	52	16	384	Yes
Heterogeneous nuclear ribonucleoprotein D0	HNRPD_HUMAN	Q14103	HNRNPD	38.4	43	14	384	Yes
Tubulin beta-6 chain	TBB6_HUMAN	Q9BUF5	TUBB6	49.8	36	6	384	No
Heme-binding protein 1	HEBP1_HUMAN	Q9NRV9	HEBP1	21.1	84	13	383	Yes
Mitochondrial import inner membrane translocase subunit TIM44	TIM44_HUMAN	O43615	TIMM44	51.3	41	22	382	Yes
Keratin, type II cuticular Hb6	KRT86_HUMAN	O43790	KRT86	53.5	52	2	382	No
Acylamino-acid-releasing enzyme	ACPH_HUMAN	P13798	APEH	81.2	35	22	382	Yes
Serine beta-lactamase-like protein LACTB, mitochondrial	LACTB_HUMAN	P83111	LACTB	60.7	46	27	382	Yes
3-ketoacyl-CoA thiolase, peroxisomal	THIK_HUMAN	P09110	ACAA1	44.3	58	21	380	Yes
Lysine--tRNA ligase	SYK_HUMAN	Q15046	KARS1	68	39	25	380	Yes
Glutathione S-transferase omega-1	GSTO1_HUMAN	P78417	GSTO1	27.5	39	13	379	Yes
Nucleosome assembly protein 1-like 4	NP1L4_HUMAN	Q99733	NAP1L4	42.8	51	16	379	Yes
Annexin A3	ANXA3_HUMAN	P12429	ANXA3	36.4	54	19	378	Yes
Septin-2	SEPT2_HUMAN	Q15019	SEPTIN2	41.5	73	19	377	Yes
NADH dehydrogenase [ubiquinone] iron-sulfur protein 5	NDUS5_HUMAN	O43920	NDUFS5	12.5	77	14	376	Yes
Echinoderm microtubule-associated protein-like 2	EMAL2_HUMAN	O95834	EML2	70.6	36	17	376	Yes
Carnitine O-palmitoyltransferase 1, muscle isoform	CPT1B_HUMAN	Q92523	CPT1B	87.7	32	22	376	Yes
NADH-cytochrome b5 reductase 1	NB5R1_HUMAN	Q9UHQ9	CYB5R1	34.1	50	15	375	Yes
Sodium/potassium-transporting ATPase subunit beta-1	AT1B1_HUMAN	P05026	ATP1B1	35	47	16	374	Yes
Methanethiol oxidase	SBP1_HUMAN	Q13228	SELENBP1	52.4	61	23	374	Yes
Mimecan	MIME_HUMAN	P20774	OGN	33.9	40	14	372	Yes
Myosin-14	MYH14_HUMAN	Q7Z406	MYH14	227.7	24	26	372	No
Ras-related protein Rab-1B	RAB1B_HUMAN	Q9H0U4	RAB1B	22.2	62	5	371	Yes
Transgelin-2	TAGL2_HUMAN	P37802	TAGLN2	22.4	80	18	370	Yes
NADH dehydrogenase [ubiquinone] iron-sulfur protein 6, mitochondrial	NDUS6_HUMAN	O75380	NDUFS6	13.7	61	7	369	Yes
cAMP-dependent protein kinase type II-beta regulatory subunit	KAP3_HUMAN	P31323	PRKAR2B	46.3	44	13	369	Yes

ATP synthase subunit e, mitochondrial	ATP5I_HUMAN	P56385	ATP5ME	7.9	68	8	369	Yes
Ras-related protein Rab-1A	RAB1A_HUMAN	P62820	RAB1A	22.7	62	6	369	Yes
General vesicular transport factor p115	USO1_HUMAN	O60763	USO1	107.8	32	24	368	Yes
Metaxin-2	MTX2_HUMAN	O75431	MTX2	29.7	46	14	368	Yes
Transaldolase	TALDO_HUMAN	P37837	TALDO1	37.5	38	15	368	Yes
ATPase family AAA domain-containing protein 3A	ATD3A_HUMAN	Q9NVI7	ATAD3A	71.3	42	10	368	Yes
NADH dehydrogenase [ubiquinone] iron-sulfur protein 7, mitochondrial	NDUS7_HUMAN	O75251	NDUFS7	23.5	26	7	367	Yes
Aspartyl/asparaginyl beta-hydroxylase	ASPH_HUMAN	Q12797	ASPH	85.8	33	20	367	Yes
Dihydropyrimidinase-related protein 3	DPYL3_HUMAN	Q14195	DPYSL3	61.9	58	20	367	Yes
Transforming growth factor-beta-induced protein ig-h3	BGH3_HUMAN	Q15582	TGFBI	74.6	40	26	367	Yes
Malonyl-CoA decarboxylase, mitochondrial	DCMC_HUMAN	O95822	MLYCD	55	41	17	364	Yes
Keratin, type I cytoskeletal 17	K1C17_HUMAN	Q04695	KRT17	48.1	55	15	363	Yes
Heterogeneous nuclear ribonucleoproteins C1/C2	HNRPC_HUMAN	P07910	HNRNPC	33.7	39	8	362	Yes
Dynein light chain 2, cytoplasmic	DYL2_HUMAN	Q96FJ2	DYNLL2	10.3	65	5	362	Yes
S-formylglutathione hydrolase	ESTD_HUMAN	P10768	ESD	31.4	57	14	361	Yes
Microtubule-associated protein 1B	MAP1B_HUMAN	P46821	MAP1B	270.5	26	45	361	Yes
Alpha-soluble NSF attachment protein	SNAA_HUMAN	P54920	NAPA	33.2	82	20	361	Yes
Heat shock protein beta-2	HSPB2_HUMAN	Q16082	HSPB2	20.2	79	9	361	Yes
Target of Nesh-SH3	TARSH_HUMAN	Q7Z7G0	ABI3BP	118.6	17	17	361	Yes
SPRY domain-containing protein 4	SPRY4_HUMAN	Q8WW59	SPRYD4	23.1	61	15	361	Yes
Supervillin	SVIL_HUMAN	O95425	SVIL	247.6	21	37	360	Yes
GrpE protein homolog 1, mitochondrial	GRPE1_HUMAN	Q9HAV7	GRPEL1	24.3	67	18	360	Yes
Dystrobrevin alpha	DTNA_HUMAN	Q9Y4J8	DTNA	83.8	29	23	359	Yes
Histone H2A type 1-B/E	H2A1B_HUMAN	P04908	H2AC4	14.1	41	3	358	Yes
N(G),N(G)-dimethylarginine dimethylaminohydrolase 2	DDAH2_HUMAN	O95865	DDAH2	29.6	60	14	357	Yes
60S acidic ribosomal protein P0	RLA0_HUMAN	P05388	RPLP0	34.3	52	15	356	Yes
Aldehyde dehydrogenase family 3 member A2	AL3A2_HUMAN	P51648	ALDH3A2	54.8	35	16	354	Yes
Desmoglein-2	DSG2_HUMAN	Q14126	DSG2	122.2	34	27	354	Yes
Fibulin-2	FBLN2_HUMAN	P98095	FBLN2	126.5	26	24	351	Yes
Collagen alpha-1(XIV) chain	COEA1_HUMAN	Q05707	COL14A1	193.4	18	26	350	Yes
Caveolae-associated protein 4	CAVN4_HUMAN	Q5BKX8	CAVIN4	41.9	48	21	350	Yes
X-ray repair cross-complementing protein 5	XRCC5_HUMAN	P13010	XRCC5	82.7	36	25	348	Yes
Neutral cholesterol ester hydrolase 1	NCEH1_HUMAN	Q6PIU2	NCEH1	45.8	37	11	348	Yes
Eukaryotic translation initiation factor 3 subunit C	EIF3C_HUMAN	Q99613	EIF3C	105.3	29	24	346	Yes
Dihydropteridine reductase	DHPR_HUMAN	P09417	QDPR	25.8	58	13	345	Yes

Retinol dehydrogenase 13	RDH13_HUMAN	Q8NBN7	RDH13	35.9	45	18	344	Yes
T-complex protein 1 subunit zeta	TCPZ_HUMAN	P40227	CCT6A	58	40	21	343	Yes
T-complex protein 1 subunit eta	TCPH_HUMAN	Q99832	CCT7	59.3	52	24	343	Yes
Vitronectin	VTNC_HUMAN	P04004	VTN	54.3	32	14	342	Yes
Cardiac phospholamban	PPLA_HUMAN	P26678	PLN	6.1	23	2	342	Yes
Heme oxygenase 2	HMOX2_HUMAN	P30519	HMOX2	36	64	16	342	Yes
Serine/threonine-protein phosphatase PP1-beta catalytic subunit	PP1B_HUMAN	P62140	PPP1CB	37.2	39	3	342	Yes
Src substrate cortactin	SRC8_HUMAN	Q14247	CTTN	61.5	45	22	340	Yes
Heterogeneous nuclear ribonucleoprotein U	HNRPU_HUMAN	Q00839	HNRNPU	90.5	33	27	339	Yes
Poly(rC)-binding protein 1	PCBP1_HUMAN	Q15365	PCBP1	37.5	44	7	339	Yes
Cytosolic non-specific dipeptidase	CNDP2_HUMAN	Q96KP4	CNDP2	52.8	53	21	339	Yes
Integrin-linked protein kinase	ILK_HUMAN	Q13418	ILK	51.4	43	18	337	Yes
Protein disulfide isomerase CRELD1	CREL1_HUMAN	Q96HD1	CRELD1	45.4	47	16	337	Yes
Histone H2AX	H2AX_HUMAN	P16104	H2AX	15.1	44	3	336	Yes
Acetolactate synthase-like protein	ILVBL_HUMAN	A1L0T0	ILVBL	67.8	37	17	335	Yes
Utrophin	UTRO_HUMAN	P46939	UTRN	394.2	16	43	334	Yes
Transcriptional activator protein Pur-alpha	PURA_HUMAN	Q00577	PURA	34.9	57	15	334	Yes
26S proteasome non-ATPase regulatory subunit 11	PSD11_HUMAN	O00231	PSMD11	47.4	58	28	333	Yes
Ras-related protein Rab-7a	RAB7A_HUMAN	P51149	RAB7A	23.5	66	12	333	Yes
Tight junction protein ZO-1	ZO1_HUMAN	Q07157	TJP1	195.3	21	28	333	Yes
Keratin, type II cytoskeletal 2 oral	K22O_HUMAN	Q01546	KRT76	65.8	15	2	332	No
Diablo homolog, mitochondrial	DBLOH_HUMAN	Q9NR28	DIABLO	27.1	44	11	332	Yes
Alpha-1B-glycoprotein	A1BG_HUMAN	P04217	A1BG	54.2	45	15	331	Yes
Cysteine desulfurase, mitochondrial	NFS1_HUMAN	Q9Y697	NFS1	50.2	57	20	331	Yes
Heterogeneous nuclear ribonucleoprotein H	HNRH1_HUMAN	P31943	HNRNPH1	49.2	39	6	330	Yes
Polyribonucleotide nucleotidyltransferase 1, mitochondrial	PNPT1_HUMAN	Q8TCS8	PNPT1	85.9	33	27	330	Yes
ATP-dependent RNA helicase DDX1	DDX1_HUMAN	Q92499	DDX1	82.4	34	20	330	Yes
RuvB-like 1	RUVB1_HUMAN	Q9Y265	RUVBL1	50.2	54	21	330	Yes
AP-2 complex subunit beta	AP2B1_HUMAN	P63010	AP2B1	104.5	22	12	329	Yes
Histidine triad nucleotide-binding protein 2, mitochondrial	HINT2_HUMAN	Q9BX68	HINT2	17.2	58	6	329	Yes
Complex I assembly factor ACAD9, mitochondrial	ACAD9_HUMAN	Q9H845	ACAD9	68.7	32	20	329	Yes
Alpha-2-HS-glycoprotein	FETUA_HUMAN	P02765	AHSG	39.3	35	12	328	Yes
NADH dehydrogenase [ubiquinone] 1 beta subcomplex subunit 7	NDUB7_HUMAN	P17568	NDUFB7	16.4	74	11	328	Yes
Proteasome subunit alpha type-5	PSA5_HUMAN	P28066	PSMA5	26.4	57	11	328	Yes
Glutathione peroxidase 3	GPX3_HUMAN	P22352	GPX3	25.5	42	9	326	Yes
Sepiapterin reductase	SPRE_HUMAN	P35270	SPR	28	59	13	325	Yes

Acyl-CoA-binding protein	ACBP_HUMAN	P07108	DBI	10	76	9	324	Yes
Thiosulfate sulfurtransferase	THTR_HUMAN	Q16762	TST	33.4	48	13	324	Yes
L-2-hydroxyglutarate dehydrogenase, mitochondrial	L2HDH_HUMAN	Q9H9P8	L2HGDH	50.3	42	18	324	Yes
Keratin, type II cytoskeletal 8	K2C8_HUMAN	P05787	KRT8	53.7	22	6	323	No
Beta-hexosaminidase subunit beta	HEXB_HUMAN	P07686	HEXB	63.1	34	18	323	Yes
5-demethoxyubiquinone hydroxylase, mitochondrial	COQ7_HUMAN	Q99807	COQ7	24.3	57	13	323	Yes
FUN14 domain-containing protein 2	FUND2_HUMAN	Q9BWH2	FUNDC2	20.7	24	8	323	Yes
EH domain-containing protein 3	EHD3_HUMAN	Q9NZN3	EHD3	60.8	59	12	322	No
Proteasome subunit beta type-4	PSB4_HUMAN	P28070	PSMB4	29.2	41	8	321	Yes
Glycine amidinotransferase, mitochondrial	GATM_HUMAN	P50440	GATM	48.4	47	17	321	Yes
NAD-dependent protein deacylase sirtuin-5, mitochondrial	SIR5_HUMAN	Q9NXA8	SIRT5	33.9	46	13	321	Yes
Elongation factor 1-beta	EF1B_HUMAN	P24534	EEF1B2	24.7	72	11	320	Yes
Synaptic vesicle membrane protein VAT-1 homolog	VAT1_HUMAN	Q99536	VAT1	41.9	49	16	320	Yes
Ubiquinone biosynthesis O-methyltransferase, mitochondrial	COQ3_HUMAN	Q9NZJ6	COQ3	41	36	11	319	Yes
Xin actin-binding repeat-containing protein 2	XIRP2_HUMAN	A4UGR9	XIRP2	382.1	16	46	318	Yes
Cartilage oligomeric matrix protein	COMP_HUMAN	P49747	COMP	82.8	35	14	318	Yes
Keratin, type II cuticular Hb5	KRT85_HUMAN	P78386	KRT85	55.8	57	12	317	No
ATP synthase mitochondrial F1 complex assembly factor 1	ATPF1_HUMAN	Q5TC12	ATPAF1	36.4	44	17	317	Yes
Vesicle-associated membrane protein-associated protein A	VAPA_HUMAN	Q9P0L0	VAPA	27.9	71	16	317	Yes
Plastin-3	PLST_HUMAN	P13797	PLS3	70.8	33	13	316	Yes
26S proteasome regulatory subunit 6B	PRS6B_HUMAN	P43686	PSMC4	47.3	56	22	315	Yes
14-3-3 protein sigma	1433S_HUMAN	P31947	SFN	27.8	40	4	314	No
Calcium-binding mitochondrial carrier protein Aralar2	CMC2_HUMAN	Q9UJS0	SLC25A13	74.1	18	4	314	No
Serine-threonine kinase receptor-associated protein	STRAP_HUMAN	Q9Y3F4	STRAP	38.4	61	17	313	Yes
40S ribosomal protein S19	RS19_HUMAN	P39019	RPS19	16.1	57	12	312	Yes
Cytoplasmic dynein 1 intermediate chain 1	DC1I1_HUMAN	O14576	DYNC1I1	72.9	40	19	311	Yes
Vesicle-associated membrane protein-associated protein B/C	VAPB_HUMAN	O95292	VAPB	27.2	56	13	311	Yes
Guanine nucleotide-binding protein G(s) subunit alpha isoforms short	GNAS2_HUMAN	P63092	GNAS	45.6	35	13	311	Yes
Nucleoside diphosphate kinase B	NDKB_HUMAN	P22392	NME2	17.3	61	4	309	Yes
Lactadherin	MFGM_HUMAN	Q08431	MFGE8	43.1	51	17	309	Yes
Acyl carrier protein, mitochondrial	ACPM_HUMAN	O14561	NDUFAB1	17.4	24	6	308	Yes
Troponin I, slow skeletal muscle	TNNI1_HUMAN	P19237	TNNI1	21.7	38	10	308	Yes
Peroxiredoxin-6	PRDX6_HUMAN	P30041	PRDX6	25	73	19	307	Yes
Lanosterol synthase	ERG7_HUMAN	P48449	LSS	83.3	38	24	307	Yes
Heterogeneous nuclear ribonucleoprotein A1	ROA1_HUMAN	P09651	HNRNPA1	38.7	46	14	305	Yes

Tumor protein D54	TPD54_HUMAN	O43399	TPD52L2	22.2	47	13	304	Yes
Aflatoxin B1 aldehyde reductase member 2	ARK72_HUMAN	O43488	AKR7A2	39.6	36	12	304	Yes
Core histone macro-H2A.1	H2AY_HUMAN	O75367	MACROH2A1	39.6	48	12	304	Yes
Ras-related protein Rab-2A	RAB2A_HUMAN	P61019	RAB2A	23.5	75	15	303	Yes
60S ribosomal protein L10a	RL10A_HUMAN	P62906	RPL10A	24.8	36	9	302	Yes
Heat shock protein beta-3	HSPB3_HUMAN	Q12988	HSPB3	17	53	11	302	Yes
All-trans-retinol dehydrogenase [NAD(+)] ADH1B	ADH1B_HUMAN	P00325	ADH1B	39.8	51	19	301	Yes
Sorcin	SORCN_HUMAN	P30626	SRI	21.7	47	9	301	Yes
NADH dehydrogenase [ubiquinone] 1 beta subcomplex subunit 9	NDUB9_HUMAN	Q9Y6M9	NDUFB9	21.8	64	12	301	Yes
Inter-alpha-trypsin inhibitor heavy chain H1	ITIH1_HUMAN	P19827	ITIH1	101.3	20	15	300	Yes
Peroxisomal multifunctional enzyme type 2	DHB4_HUMAN	P51659	HSD17B4	79.6	42	25	300	Yes
Serine/threonine-protein phosphatase PP1-alpha catalytic subunit	PP1A_HUMAN	P62136	PPP1CA	37.5	38	3	300	Yes
Desmoplakin	DESP_HUMAN	P15924	DSP	331.6	19	49	297	Yes
NADPH--cytochrome P450 reductase	NCPR_HUMAN	P16435	POR	76.6	42	26	296	Yes
Lamin-B1	LMNB1_HUMAN	P20700	LMNB1	66.4	40	18	296	Yes
Enoyl-CoA hydratase domain-containing protein 3, mitochondrial	ECHD3_HUMAN	Q96DC8	ECHDC3	32.6	50	11	296	Yes
Heterogeneous nuclear ribonucleoprotein Q	HNRPQ_HUMAN	O60506	SYNCRIP	69.6	38	13	294	Yes
Ras GTPase-activating-like protein IQGAP1	IQGA1_HUMAN	P46940	IQGAP1	189.1	25	35	294	Yes
Caveolae-associated protein 3	CAVN3_HUMAN	Q969G5	CAVIN3	27.7	50	17	293	Yes
CDK5 regulatory subunit-associated protein 3	CK5P3_HUMAN	Q96JB5	CDK5RAP3	56.9	30	15	293	Yes
Mitochondrial intermediate peptidase	MIPEP_HUMAN	Q99797	MIPEP	80.6	37	23	293	Yes
Immunoglobulin alpha-2 heavy chain	IGA2_HUMAN	P0DOX2	N/A	48.9	40	5	292	Yes
Proteasome subunit beta type-1	PSB1_HUMAN	P20618	PSMB1	26.5	46	12	292	Yes
Matrin-3	MATR3_HUMAN	P43243	MATR3	94.6	19	15	292	Yes
Cysteine-rich protein 2	CRIP2_HUMAN	P52943	CRIP2	22.5	52	9	292	Yes
Rab GDP dissociation inhibitor beta	GDIB_HUMAN	P50395	GDI2	50.6	54	12	291	Yes
NADH dehydrogenase [ubiquinone] 1 alpha subcomplex assembly factor 4	NDUF4_HUMAN	Q9P032	NDUFAF4	20.3	66	14	291	Yes
Oligoribonuclease, mitochondrial	ORN_HUMAN	Q9Y3B8	REXO2	26.8	49	11	290	Yes
Beta-centractin	ACTY_HUMAN	P42025	ACTR1B	42.3	49	6	289	No
RuvB-like 2	RUVB2_HUMAN	Q9Y230	RUVBL2	51.1	46	20	289	Yes
Destrin	DEST_HUMAN	P60981	DSTN	18.5	43	10	288	Yes
Gamma-sarcoglycan	SGCG_HUMAN	Q13326	SGCG	32.4	56	14	288	Yes
PDZ and LIM domain protein 3	PDLI3_HUMAN	Q53GG5	PDLIM3	39.2	45	10	288	Yes
Septin-11	SEP11_HUMAN	Q9NVA2	SEPTIN11	49.4	42	8	288	Yes
Inter-alpha-trypsin inhibitor heavy chain H2	ITIH2_HUMAN	P19823	ITIH2	106.4	32	23	287	Yes

26S proteasome non-ATPase regulatory subunit 4	PSMD4_HUMAN	P55036	PSMD4	40.7	36	12	287	Yes
Small glutamine-rich tetratricopeptide repeat-containing protein alpha	SGTA_HUMAN	O43765	SGTA	34	38	12	286	Yes
Peptidyl-prolyl cis-trans isomerase B	PPIB_HUMAN	P23284	PPIB	23.7	64	13	285	Yes
Tryptophan--tRNA ligase, cytoplasmic	SYWC_HUMAN	P23381	WARS1	53.1	44	18	284	Yes
Ankyrin-2	ANK2_HUMAN	Q01484	ANK2	433.4	10	22	284	Yes
Eukaryotic initiation factor 4A-II	IF4A2_HUMAN	Q14240	EIF4A2	46.4	40	6	284	Yes
Adipocyte enhancer-binding protein 1	AEBP1_HUMAN	Q8IUX7	AEBP1	130.8	18	16	284	Yes
[Pyruvate dehydrogenase [acetyl-transferring]]-phosphatase 1, mitochondrial	PDP1_HUMAN	Q9P0J1	PDP1	61	50	21	284	Yes
RNA transcription, translation and transport factor protein	RTRAF_HUMAN	Q9Y224	RTRAF	28.1	66	14	284	Yes
Protein transport protein Sec31A	SC31A_HUMAN	O94979	SEC31A	132.9	18	20	283	Yes
Cytochrome c oxidase subunit 2	COX2_HUMAN	P00403	MT-CO2	25.5	41	5	282	Yes
Glutathione S-transferase P	GSTP1_HUMAN	P09211	GSTP1	23.3	55	9	282	Yes
Versican core protein	CSPG2_HUMAN	P13611	VCAN	372.6	5	18	282	Yes
ATP synthase subunit g, mitochondrial	ATP5L_HUMAN	O75964	ATP5MG	11.4	52	6	281	Yes
Cathepsin B	CATB_HUMAN	P07858	CTSB	37.8	42	12	281	Yes
Platelet glycoprotein 4	CD36_HUMAN	P16671	CD36	53	18	9	281	Yes
Hsp90 co-chaperone Cdc37	CDC37_HUMAN	Q16543	CDC37	44.4	40	15	281	Yes
Ferritin light chain	FRIL_HUMAN	P02792	FTL	20	51	10	280	Yes
Cell surface glycoprotein MUC18	MUC18_HUMAN	P43121	MCAM	71.6	33	20	280	Yes
Alpha-1-antichymotrypsin	AACT_HUMAN	P01011	SERPINA3	47.6	27	14	277	Yes
Calpain-1 catalytic subunit	CAN1_HUMAN	P07384	CAPN1	81.8	27	18	277	Yes
Gamma-enolase	ENOG_HUMAN	P09104	ENO2	47.2	35	8	277	Yes
Platelet-activating factor acetylhydrolase IB subunit alpha	LIS1_HUMAN	P43034	PAFAH1B1	46.6	52	18	277	Yes
Protein FAM136A	F136A_HUMAN	Q96C01	FAM136A	15.6	91	15	277	Yes
Lupus La protein	LA_HUMAN	P05455	SSB	46.8	39	17	276	Yes
Delta-aminolevulinic acid dehydratase	HEM2_HUMAN	P13716	ALAD	36.3	44	15	275	Yes
Thymosin beta-4	TYB4_HUMAN	P62328	TMSB4X	5.1	57	6	275	Yes
COP9 signalosome complex subunit 4	CSN4_HUMAN	Q9BT78	COPS4	46.2	64	18	275	Yes
Protein NipSnap homolog 3A	NPS3A_HUMAN	Q9UFN0	NIPSNAP3A	28.4	65	11	275	Yes
Kelch-like protein 41	KLH41_HUMAN	O60662	KLHL41	68	29	22	274	Yes
NADH dehydrogenase [ubiquinone] 1 beta subcomplex subunit 8, mitochondrial	NDUB8_HUMAN	O95169	NDUFB8	21.8	46	8	274	Yes
Vacuolar protein sorting-associated protein 29	VPS29_HUMAN	Q9UBQ0	VPS29	20.5	52	9	274	Yes
Transthyretin	TTHY_HUMAN	P02766	TTR	15.9	69	11	273	Yes

Palladin	PALLD_HUMAN	Q8WX93	PALLD	150.5	21	23	273	Yes
Cytochrome b5	CYB5_HUMAN	P00167	CYB5A	15.3	49	6	271	Yes
Plasma membrane calcium-transporting ATPase 4	AT2B4_HUMAN	P23634	ATP2B4	137.8	26	18	271	Yes
Sodium/calcium exchanger 1	NAC1_HUMAN	P32418	SLC8A1	108.5	24	22	271	Yes
6-phosphogluconate dehydrogenase, decarboxylating	6PGD_HUMAN	P52209	PGD	53.1	34	15	271	Yes
Histone H3.1	H31_HUMAN	P68431	H3C1	15.4	39	2	271	Yes
Interleukin enhancer-binding factor 3	ILF3_HUMAN	Q12906	ILF3	95.3	24	19	271	Yes
Hydroxyacylglutathione hydrolase, mitochondrial	GLO2_HUMAN	Q16775	HAGH	33.8	42	11	271	Yes
Band 4.1-like protein 2	E41L2_HUMAN	O43491	EPB41L2	112.5	23	20	270	Yes
Alpha-aminoadipic semialdehyde synthase, mitochondrial	AASS_HUMAN	Q9UDR5	AASS	102.1	32	25	269	Yes
Mitochondrial import inner membrane translocase subunit Tim13	TIM13_HUMAN	Q9Y5L4	TIMM13	10.5	62	6	269	Yes
Alpha-1-acid glycoprotein 1	A1AG1_HUMAN	P02763	ORM1	23.5	43	6	266	Yes
Bifunctional purine biosynthesis protein PURH	PUR9_HUMAN	P31939	ATIC	64.6	46	22	266	Yes
Fermitin family homolog 2	FERM2_HUMAN	Q96AC1	FERMT2	77.8	33	20	266	Yes
PRA1 family protein 3	PRAF3_HUMAN	O75915	ARL6IP5	21.6	24	5	265	Yes
Collagen alpha-1(VI) chain	CO6A1_HUMAN	P12109	COL6A1	108.5	19	18	265	Yes
Thioredoxin reductase 1, cytoplasmic	TRXR1_HUMAN	Q16881	TXNRD1	70.9	30	20	264	Yes
Leucine-rich repeat-containing protein 47	LRC47_HUMAN	Q8N1G4	LRRC47	63.4	40	20	264	Yes
Far upstream element-binding protein 1	FUBP1_HUMAN	Q96AE4	FUBP1	67.5	37	16	264	Yes
Tripeptidyl-peptidase 1	TPP1_HUMAN	O14773	TPP1	61.2	20	7	263	Yes
Nucleobindin-2	NUCB2_HUMAN	P80303	NUCB2	50.2	46	17	263	Yes
Single-stranded DNA-binding protein, mitochondrial	SSBP_HUMAN	Q04837	SSBP1	17.2	59	12	262	Yes
Adenosylhomocysteinase	SAHH_HUMAN	P23526	AHCY	47.7	42	17	261	Yes
Guanine nucleotide-binding protein subunit beta-4	GBB4_HUMAN	Q9HAV0	GNB4	37.5	31	3	261	No
Glycine cleavage system H protein, mitochondrial	GCSH_HUMAN	P23434	GCSH	18.9	23	3	260	Yes
Palmitoyl-protein thioesterase 1	PPT1_HUMAN	P50897	PPT1	34.2	42	12	260	Yes
Mitochondrial fission 1 protein	FIS1_HUMAN	Q9Y3D6	FIS1	16.9	44	7	260	Yes
Methylmalonyl-CoA epimerase, mitochondrial	MCEE_HUMAN	Q96PE7	MCEE	18.7	66	9	258	Yes
Mitochondrial import inner membrane translocase subunit Tim9	TIM9_HUMAN	Q9Y5J7	TIMM9	10.4	74	7	258	Yes
Reticulon-4-interacting protein 1, mitochondrial	RT4I1_HUMAN	Q8WWV3	RTN4IP1	43.6	54	15	257	Yes
Elongin-C	ELOC_HUMAN	Q15369	ELOC	12.5	66	7	256	Yes
Proteasome subunit beta type-7	PSB7_HUMAN	Q99436	PSMB7	29.9	36	11	256	Yes
NSFL1 cofactor p47	NSF1C_HUMAN	Q9UNZ2	NSFL1C	40.5	58	19	256	Yes
Vesicle-trafficking protein SEC22b	SC22B_HUMAN	O75396	SEC22B	24.6	52	11	255	Yes
Proteasome subunit alpha type-2	PSA2_HUMAN	P25787	PSMA2	25.9	53	12	254	Yes
Ras-related protein Rab-10	RAB10_HUMAN	P61026	RAB10	22.5	50	7	254	Yes

Peptidyl-prolyl cis-trans isomerase FKBP9	FKBP9_HUMAN	O95302	FKBP9	63	28	17	253	Yes
Elongin-B	ELOB_HUMAN	Q15370	ELOB	13.1	86	10	253	Yes
Dynamin-1-like protein	DNM1L_HUMAN	O00429	DNM1L	81.8	42	25	252	Yes
Copine-3	CPNE3_HUMAN	O75131	CPNE3	60.1	43	21	252	Yes
28S ribosomal protein S22, mitochondrial	RT22_HUMAN	P82650	MRPS22	41.3	48	15	252	Yes
Clathrin light chain A	CLCA_HUMAN	P09496	CLTA	27.1	27	10	251	Yes
Estradiol 17-beta-dehydrogenase 8	DHB8_HUMAN	Q92506	HSD17B8	27	44	10	251	Yes
2-iminobutanoate/2-iminopropanoate deaminase	RIDA_HUMAN	P52758	RIDA	14.5	71	7	250	Yes
Thioredoxin	THIO_HUMAN	P10599	TXN	11.7	58	9	249	Yes
CD59 glycoprotein	CD59_HUMAN	P13987	CD59	14.2	28	7	249	Yes
Heterogeneous nuclear ribonucleoprotein M	HNRPM_HUMAN	P52272	HNRNPM	77.5	40	22	249	Yes
Methylglutaconyl-CoA hydratase, mitochondrial	AUHM_HUMAN	Q13825	AUH	35.6	37	15	249	Yes
Phosphatidylinositide phosphatase SAC1	SAC1_HUMAN	Q9NTJ5	SACM1L	66.9	30	19	249	Yes
ATP synthase subunit epsilon, mitochondrial	ATP5E_HUMAN	P56381	ATP5F1E	5.8	75	4	248	Yes
NADH dehydrogenase [ubiquinone] 1 beta subcomplex subunit 4	NDUB4_HUMAN	O95168	NDUFB4	15.2	50	9	246	Yes
Acidic leucine-rich nuclear phosphoprotein 32 family member A	AN32A_HUMAN	P39687	ANP32A	28.6	37	8	246	Yes
EF-hand domain-containing protein D1	EFHD1_HUMAN	Q9BUP0	EFHD1	26.9	65	14	246	Yes
Zinc-alpha-2-glycoprotein	ZA2G_HUMAN	P25311	AZGP1	34.2	52	14	245	Yes
Carbonic anhydrase 1	CAH1_HUMAN	P00915	CA1	28.9	51	10	244	Yes
Insulin-degrading enzyme	IDE_HUMAN	P14735	IDE	117.9	31	27	244	Yes
Muscular LMNA-interacting protein	MLIP_HUMAN	Q5VWP3	MLIP	50.4	52	19	244	Yes
A-kinase anchor protein 2	AKAP2_HUMAN	Q9Y2D5	AKAP2	94.6	37	24	244	Yes
Cytochrome c oxidase subunit NDUFA4	NDUA4_HUMAN	O00483	NDUFA4	9.4	56	7	243	Yes
Ras-related protein Rab-11B	RB11B_HUMAN	Q15907	RAB11B	24.5	47	10	243	Yes
Extended synaptotagmin-1	ESYT1_HUMAN	Q9BSJ8	ESYT1	122.8	28	25	242	Yes
Adipocyte plasma membrane-associated protein	APMAP_HUMAN	Q9HDC9	APMAP	46.5	35	14	242	Yes
Catenin delta-1	CTND1_HUMAN	O60716	CTNND1	108.1	33	25	241	Yes
Carboxymethylenebutenolidase homolog	CMBL_HUMAN	Q96DG6	CMBL	28	37	13	241	Yes
Protein S100-A13	S10AD_HUMAN	Q99584	S100A13	11.5	55	9	241	Yes
28S ribosomal protein S27, mitochondrial	RT27_HUMAN	Q92552	MRPS27	47.6	36	11	240	Yes
Septin-9	SEPT9_HUMAN	Q9UHD8	SEPTIN9	65.4	33	16	240	Yes
Coatomer subunit beta'	COPB2_HUMAN	P35606	COPB2	102.4	33	23	239	Yes
Transforming protein RhoA	RHOA_HUMAN	P61586	RHOA	21.8	50	4	239	Yes
Protein phosphatase 1 regulatory subunit 7	PP1R7_HUMAN	Q15435	PPP1R7	41.5	39	16	239	Yes
Isochorismatase domain-containing protein 1	ISOC1_HUMAN	Q96CN7	ISOC1	32.2	47	13	239	Yes
Alanine aminotransferase 1	ALAT1_HUMAN	P24298	GPT	54.6	44	15	238	Yes

Flavin reductase (NADPH)	BLVRB_HUMAN	P30043	BLVRB	22.1	60	10	238	Yes
Translationally-controlled tumor protein	TCTP_HUMAN	P13693	TPT1	19.6	34	7	237	Yes
Ras-related protein Rap-1A	RAP1A_HUMAN	P62834	RAP1A	21	53	2	237	Yes
Keratin, type II cytoskeletal 79	K2C79_HUMAN	Q5XKE5	KRT79	57.8	33	9	237	No
HLA class I histocompatibility antigen, A alpha chain	HLAA_HUMAN	P01892	HLA-A	40.9	55	2	236	Yes
Splicing factor, proline- and glutamine-rich	SFPQ_HUMAN	P23246	SFPQ	76.1	29	17	236	Yes
Heme-binding protein 2	HEBP2_HUMAN	Q9Y5Z4	HEBP2	22.9	53	11	236	Yes
ATPase ASNA1	ASNA_HUMAN	O43681	GET3	38.8	45	12	234	Yes
Proteasome subunit beta type-6	PSB6_HUMAN	P28072	PSMB6	25.3	47	7	234	Yes
Striatin-3	STRN3_HUMAN	Q13033	STRN3	87.2	22	13	234	Yes
ADP-ribose glycohydrolase MACROD1	MACD1_HUMAN	Q9BQ69	MACROD1	35.5	39	11	234	Yes
Protein AMBP	AMBP_HUMAN	P02760	AMBP	39	44	16	232	Yes
Hydroxymethylglutaryl-CoA lyase, mitochondrial	HMGCL_HUMAN	P35914	HMGCL	34.3	31	11	232	Yes
E3 ubiquitin-protein ligase HUWE1	HUWE1_HUMAN	Q7Z6Z7	HUWE1	481.6	8	25	232	Yes
Sialate O-acetylesterase	SIAE_HUMAN	Q9HAT2	SIAE	58.3	23	12	232	Yes
40S ribosomal protein SA	RSSA_HUMAN	P08865	RPSA	32.8	51	10	231	Yes
ATPase family AAA domain-containing protein 3B	ATD3B_HUMAN	Q5T9A4	ATAD3B	72.5	26	2	231	Yes
Alpha-1-acid glycoprotein 2	A1AG2_HUMAN	P19652	ORM2	23.6	29	5	230	Yes
Programmed cell death protein 5	PDCD5_HUMAN	O14737	PDCD5	14.3	65	10	229	Yes
Immunoglobulin heavy constant mu	IGHM_HUMAN	P01871	IGHM	49.4	38	17	229	Yes
Cytochrome c oxidase subunit 6C	COX6C_HUMAN	P09669	COX6C	8.8	52	8	229	Yes
Fibulin-1	FBLN1_HUMAN	P23142	FBLN1	77.2	26	12	229	Yes
S-phase kinase-associated protein 1	SKP1_HUMAN	P63208	SKP1	18.6	67	14	229	Yes
Enhancer of rudimentary homolog	ERH_HUMAN	P84090	ERH	12.3	52	7	229	Yes
Elongation factor G, mitochondrial	EFGM_HUMAN	Q96RP9	GFM1	83.4	26	19	229	Yes
BAG family molecular chaperone regulator 2	BAG2_HUMAN	O95816	BAG2	23.8	66	11	228	Yes
Ubiquitin carboxyl-terminal hydrolase 5	UBP5_HUMAN	P45974	USP5	95.7	28	17	227	Yes
Proteasome activator complex subunit 1	PSME1_HUMAN	Q06323	PSME1	28.7	49	11	227	Yes
Far upstream element-binding protein 2	FUBP2_HUMAN	Q92945	KHSRP	73.1	38	20	227	Yes
Fatty acid-binding protein, adipocyte	FABP4_HUMAN	P15090	FABP4	14.7	69	8	226	Yes
Catechol O-methyltransferase	COMT_HUMAN	P21964	COMT	30	67	16	226	Yes
Cytochrome c oxidase subunit 7C, mitochondrial	COX7C_HUMAN	P15954	COX7C	7.2	75	6	225	Yes
Inositol monophosphatase 1	IMPA1_HUMAN	P29218	IMPA1	30.2	43	10	225	Yes
Ras-related protein Rab-14	RAB14_HUMAN	P61106	RAB14	23.9	67	13	225	Yes
Nodal modulator 1	NOMO1_HUMAN	Q15155	NOMO1	134.2	19	18	225	Yes
Inter-alpha-trypsin inhibitor heavy chain H4	ITIH4_HUMAN	Q14624	ITIH4	103.3	26	21	224	Yes

Iron-sulfur cluster assembly 2 homolog, mitochondrial	ISCA2_HUMAN	Q86U28	ISCA2	16.5	62	7	224	Yes
Apolipoprotein A-II	APOA2_HUMAN	P02652	APOA2	11.2	47	7	223	Yes
Calpain-2 catalytic subunit	CAN2_HUMAN	P17655	CAPN2	79.9	33	17	223	Yes
NADH dehydrogenase [ubiquinone] 1 alpha subcomplex subunit 6	NDUA6_HUMAN	P56556	NDUFA6	17.9	42	7	223	Yes
Succinate dehydrogenase assembly factor 2, mitochondrial	SDHF2_HUMAN	Q9NX18	SDHAF2	19.6	29	7	223	Yes
Transforming acidic coiled-coil-containing protein 2	TACC2_HUMAN	O95359	TACC2	309.2	15	30	222	Yes
Heterogeneous nuclear ribonucleoprotein H2	HNRH2_HUMAN	P55795	HNRNPH2	49.2	28	4	222	No
Guanine nucleotide-binding protein G	GNAI1_HUMAN	P63096	GNAI1	40.3	31	3	222	No
Mitochondrial 2-oxoglutarate/malate carrier protein	M2OM_HUMAN	Q02978	SLC25A11	34	39	10	222	Yes
Beta-taxilin	TXLNB_HUMAN	Q8N3L3	TXLNB	76.5	35	19	222	Yes
Integrin alpha-V	ITAV_HUMAN	P06756	ITGAV	116	28	23	221	Yes
F-actin-capping protein subunit beta	CAPZB_HUMAN	P47756	CAPZB	31.3	54	13	221	Yes
NADH dehydrogenase [ubiquinone] 1 alpha subcomplex assembly factor 3	NDUF3_HUMAN	Q9BU61	NDUFAF3	20.3	52	7	221	Yes
Dipeptidyl peptidase 2	DPP2_HUMAN	Q9UHL4	DPP7	54.3	22	9	221	Yes
NFU1 iron-sulfur cluster scaffold homolog, mitochondrial	NFU1_HUMAN	Q9UMS0	NFU1	28.4	30	7	221	Yes
Cation-dependent mannose-6-phosphate receptor	MPRD_HUMAN	P20645	M6PR	31	35	9	220	Yes
AP-1 complex subunit beta-1	AP1B1_HUMAN	Q10567	AP1B1	104.6	19	7	220	No
Poly(rC)-binding protein 2	PCBP2_HUMAN	Q15366	PCBP2	38.6	23	3	220	Yes
Neudesin	NENF_HUMAN	Q9UMX5	NENF	18.8	61	9	220	Yes
Nucleoside diphosphate-linked moiety X motif 19	NUD19_HUMAN	A8MXV4	NUDT19	42.2	50	12	219	Yes
Mitochondrial peptide methionine sulfoxide reductase	MSRA_HUMAN	Q9UJ68	MSRA	26.1	37	8	218	Yes
Neuroplastin	NPTN_HUMAN	Q9Y639	NPTN	44.4	21	9	218	Yes
Cation-independent mannose-6-phosphate receptor	MPRI_HUMAN	P11717	IGF2R	274.2	13	29	217	Yes
NADH dehydrogenase [ubiquinone] 1 alpha subcomplex assembly factor 2	NDUF2_HUMAN	Q8N183	NDUFAF2	19.8	68	14	216	Yes
Apolipoprotein C-III	APOC3_HUMAN	P02656	APOC3	10.8	59	6	215	Yes
Ras-related protein Rap-1b	RAP1B_HUMAN	P61224	RAP1B	20.8	53	3	215	Yes
Guanine nucleotide-binding protein G	GNAI3_HUMAN	P08754	GNAI3	40.5	28	2	214	No
Probable 2-oxoglutarate dehydrogenase E1 component DHKTD1, mitochondrial	DHTK1_HUMAN	Q96HY7	DHTKD1	103	32	19	214	Yes
26S proteasome non-ATPase regulatory subunit 13	PSD13_HUMAN	Q9UNM6	PSMD13	42.9	57	20	214	Yes
Apolipoprotein L2	APOL2_HUMAN	Q9BQE5	APOL2	37.1	26	11	213	Yes
Mammalian ependymin-related protein 1	EPDR1_HUMAN	Q9UM22	EPDR1	25.4	24	5	213	Yes
Apolipoprotein D	APOD_HUMAN	P05090	APOD	21.3	41	10	212	Yes
Heterogeneous nuclear ribonucleoprotein H3	HNRH3_HUMAN	P31942	HNRNPH3	36.9	28	7	212	Yes

Fibromodulin	FMOD_HUMAN	Q06828	FMOD	43.2	30	8	212	Yes
Gamma-soluble NSF attachment protein	SNAG_HUMAN	Q99747	NAPG	34.7	46	15	212	Yes
Protein S100-A9	S10A9_HUMAN	P06702	S100A9	13.2	71	9	211	Yes
Actin-related protein 3	ARP3_HUMAN	P61158	ACTR3	47.3	50	16	211	Yes
Peptidyl-prolyl cis-trans isomerase FKBP1A	FKB1A_HUMAN	P62942	FKBP1A	11.9	46	5	211	Yes
Sec1 family domain-containing protein 1	SCFD1_HUMAN	Q8WVM8	SCFD1	72.3	36	17	211	Yes
NAD-dependent protein deacetylase sirtuin-3, mitochondrial	SIR3_HUMAN	Q9NTG7	SIRT3	43.5	34	10	211	Yes
26S proteasome non-ATPase regulatory subunit 3	PSMD3_HUMAN	O43242	PSMD3	60.9	49	21	210	Yes
Glutathione S-transferase Mu 2	GSTM2_HUMAN	P28161	GSTM2	25.7	50	6	210	Yes
2-oxoisovalerate dehydrogenase subunit beta, mitochondrial	ODBB_HUMAN	P21953	BCKDHB	43.1	40	10	209	Yes
G-rich sequence factor 1	GRSF1_HUMAN	Q12849	GRSF1	53.1	32	14	209	Yes
5-oxoprolinase	OPLA_HUMAN	O14841	OPLAH	137.4	24	22	208	Yes
26S proteasome non-ATPase regulatory subunit 7	PSMD7_HUMAN	P51665	PSMD7	37	49	12	208	Yes
Cell division control protein 42 homolog	CDC42_HUMAN	P60953	CDC42	21.2	45	9	208	Yes
Protein S100-A8	S10A8_HUMAN	P05109	S100A8	10.8	63	10	207	Yes
Adrenodoxin, mitochondrial	ADX_HUMAN	P10109	FDX1	19.4	31	9	207	Yes
Calcium/calmodulin-dependent protein kinase type II subunit delta	KCC2D_HUMAN	Q13557	CAMK2D	56.3	38	12	206	Yes
SUN domain-containing protein 2	SUN2_HUMAN	Q9UH99	SUN2	80.3	36	16	206	Yes
Striatin	STRN_HUMAN	O43815	STRN	86.1	42	20	205	Yes
Leukotriene A-4 hydrolase	LKHA4_HUMAN	P09960	LTA4H	69.2	33	19	205	Yes
Myosin light chain 6B	MYL6B_HUMAN	P14649	MYL6B	22.8	36	4	205	Yes
Splicing factor 3B subunit 3	SF3B3_HUMAN	Q15393	SF3B3	135.5	19	18	205	Yes
Putative keratin-87 protein	KR87P_HUMAN	A6NCN2	KRT87P	29.1	67	2	204	No
Histidine triad nucleotide-binding protein 1	HINT1_HUMAN	P49773	HINT1	13.8	79	8	204	Yes
Eukaryotic translation initiation factor 3 subunit B	EIF3B_HUMAN	P55884	EIF3B	92.4	24	15	204	Yes
Lactoylglutathione lyase	LGUL_HUMAN	Q04760	GLO1	20.8	45	10	204	Yes
Nucleoside diphosphate kinase A	NDKA_HUMAN	P15531	NME1	17.1	58	3	203	No
Heterogeneous nuclear ribonucleoprotein U-like protein 2	HNRL2_HUMAN	Q1KMD3	HNRNPUL2	85.1	28	17	203	Yes
Isochorismatase domain-containing protein 2	ISOC2_HUMAN	Q96AB3	ISOC2	22.3	65	8	203	Yes
Protein kinase C and casein kinase substrate in neurons protein 2	PACN2_HUMAN	Q9UNF0	PACSIN2	55.7	34	16	203	Yes
Carbonic anhydrase 3	CAH3_HUMAN	P07451	CA3	29.5	47	10	202	Yes
D-dopachrome decarboxylase	DOPD_HUMAN	P30046	DDT	12.7	57	8	201	Yes
Prolyl endopeptidase	PPCE_HUMAN	P48147	PREP	80.6	36	21	201	Yes
Growth factor receptor-bound protein 2	GRB2_HUMAN	P62993	GRB2	25.2	55	11	200	Yes
Mitochondrial import inner membrane translocase subunit TIM50	TIM50_HUMAN	Q3ZCQ8	TIMM50	39.6	27	9	200	Yes

SLC35A4 upstream open reading frame protein	S35U4_HUMAN	L0R6Q1	SLC35A4	11.1	57	6	199	Yes
Plasminogen	PLMN_HUMAN	P00747	PLG	90.5	27	19	199	Yes
Proteasome subunit beta type-3	PSB3_HUMAN	P49720	PSMB3	22.9	42	10	199	Yes
MICOS complex subunit MIC27	MIC27_HUMAN	Q6UXV4	APOOL	29.1	47	10	199	Yes
Serum amyloid P-component	SAMP_HUMAN	P02743	APCS	25.4	31	10	198	Yes
Aldo-keto reductase family 1 member A1	AK1A1_HUMAN	P14550	AKR1A1	36.6	66	13	198	Yes
Ragulator complex protein LAMTOR1	LTOR1_HUMAN	Q6IAA8	LAMTOR1	17.7	65	7	198	Yes
Rho-related GTP-binding protein RhoC	RHOC_HUMAN	P08134	RHOC	22	45	2	197	Yes
Histone H2A.Z	H2AZ_HUMAN	P0C0S5	H2AZ1	13.5	31	3	197	Yes
Protein S100-A1	S10A1_HUMAN	P23297	S100A1	10.5	59	8	197	Yes
Cytochrome b-c1 complex subunit 8	QCR8_HUMAN	O14949	UQCRQ	9.9	68	9	196	Yes
Glutathione S-transferase Mu 3	GSTM3_HUMAN	P21266	GSTM3	26.5	40	12	196	Yes
Ubiquitin carboxyl-terminal hydrolase 14	UBP14_HUMAN	P54578	USP14	56	35	14	196	Yes
Calponin-1	CNN1_HUMAN	P51911	CNN1	33.2	45	13	195	Yes
Cathepsin Z	CATZ_HUMAN	Q9UBR2	CTSZ	33.8	36	9	195	Yes
Paraplegin	SPG7_HUMAN	Q9UQ90	SPG7	88.2	30	19	195	Yes
Heterogeneous nuclear ribonucleoprotein R	HNRPR_HUMAN	O43390	HNRNPR	70.9	28	9	194	Yes
Protein S100-A10	S10AA_HUMAN	P60903	S100A10	11.2	63	6	194	Yes
39S ribosomal protein L12, mitochondrial	RM12_HUMAN	P52815	MRPL12	21.3	41	7	193	Yes
Eukaryotic initiation factor 4A-I	IF4A1_HUMAN	P60842	EIF4A1	46.1	39	4	193	No
Protein TFG	TFG_HUMAN	Q92734	TFG	43.4	33	9	193	Yes
Protein disulfide-isomerase TMX3	TMX3_HUMAN	Q96JJ7	TMX3	51.8	28	10	193	Yes
28S ribosomal protein S23, mitochondrial	RT23_HUMAN	Q9Y3D9	MRPS23	21.8	59	12	193	Yes
Eukaryotic translation initiation factor 3 subunit H	EIF3H_HUMAN	O15372	EIF3H	39.9	34	11	192	Yes
Nucleolar protein 3	NOL3_HUMAN	O60936	NOL3	22.6	48	8	192	Yes
Guanine nucleotide-binding protein subunit alpha-11	GNA11_HUMAN	P29992	GNA11	42.1	39	8	192	Yes
Nucleosome assembly protein 1-like 1	NP1L1_HUMAN	P55209	NAP1L1	45.3	21	7	192	Yes
Latent-transforming growth factor beta-binding protein 4	LTBP4_HUMAN	Q8N2S1	LTBP4	173.3	17	20	192	Yes
MICOS complex subunit MIC26	MIC26_HUMAN	Q9BUR5	APOO	22.3	35	6	192	Yes
Heterogeneous nuclear ribonucleoprotein L	HNRPL_HUMAN	P14866	HNRNPL	64.1	27	13	190	Yes
116 kDa U5 small nuclear ribonucleoprotein component	U5S1_HUMAN	Q15029	EFTUD2	109.4	27	22	190	Yes
S-adenosylmethionine synthase isoform type-2	METK2_HUMAN	P31153	MAT2A	43.6	30	13	189	Yes
Transmembrane protein 65	TMM65_HUMAN	Q6PI78	TMEM65	25.5	28	6	189	Yes
Acyl-coenzyme A thioesterase 13	ACO13_HUMAN	Q9NPJ3	ACOT13	15	41	5	189	Yes
LIM and calponin homology domains-containing protein 1	LIMC1_HUMAN	Q9UPQ0	LIMCH1	121.8	19	19	189	Yes
Glutathione hydrolase 5 proenzyme	GGT5_HUMAN	P36269	GGT5	62.2	22	10	188	Yes

Hematopoietic progenitor cell antigen CD34	CD34_HUMAN	P28906	CD34	40.7	14	9	187	Yes
Lysophospholipase-like protein 1	LYPL1_HUMAN	Q5VWZ2	LYPLAL1	26.3	44	9	187	Yes
Nucleoprotein TPR	TPR_HUMAN	P12270	TPR	267.1	14	29	186	Yes
Caveolin-1	CAV1_HUMAN	Q03135	CAV1	20.5	46	7	186	Yes
Cytochrome c oxidase assembly factor 6 homolog	COA6_HUMAN	Q5JTJ3	COA6	14.1	45	9	186	Yes
Myopalladin	MYPN_HUMAN	Q86TC9	MYPN	145.2	23	21	186	Yes
CDGSH iron-sulfur domain-containing protein 1	CISD1_HUMAN	Q9NZ45	CISD1	12.2	55	8	186	Yes
Leucyl-cystinyl aminopeptidase	LCAP_HUMAN	Q9UIQ6	LNPEP	117.3	18	16	186	Yes
Methionine-R-sulfoxide reductase B2, mitochondrial	MSRB2_HUMAN	Q9Y3D2	MSRB2	19.5	68	10	186	Yes
Cytoplasmic dynein 1 light intermediate chain 1	DC1L1_HUMAN	Q9Y6G9	DYNC1LI1	56.5	34	15	186	Yes
Starch-binding domain-containing protein 1	STBD1_HUMAN	O95210	STBD1	39	44	15	185	Yes
Protein S100-A4	S10A4_HUMAN	P26447	S100A4	11.7	28	4	185	Yes
Transmembrane protein 143	TM143_HUMAN	Q96AN5	TMEM143	51.7	31	10	185	Yes
Ubiquilin-1	UBQL1_HUMAN	Q9UMX0	UBQLN1	62.5	29	4	185	Yes
Plastin-2	PLSL_HUMAN	P13796	LCP1	70.2	40	16	184	Yes
Eukaryotic translation initiation factor 4B	IF4B_HUMAN	P23588	EIF4B	69.1	17	8	184	Yes
Protein flightless-1 homolog	FLII_HUMAN	Q13045	FLII	144.7	19	22	184	Yes
Chromobox protein homolog 3	CBX3_HUMAN	Q13185	CBX3	20.8	37	5	184	Yes
Frataxin, mitochondrial	FRDA_HUMAN	Q16595	FXN	23.1	39	9	184	Yes
Perilipin-1	PLIN1_HUMAN	O60240	PLIN1	56	48	19	182	No
Prostaglandin-H2 D-isomerase	PTGDS_HUMAN	P41222	PTGDS	21	32	6	182	Yes
COP9 signalosome complex subunit 2	CSN2_HUMAN	P61201	COPS2	51.6	30	13	182	Yes
Mitochondrial intermembrane space import and assembly protein 40	MIA40_HUMAN	Q8N4Q1	CHCHD4	16	51	8	182	Yes
Microtubule-associated protein tau	TAU_HUMAN	P10636	MAPT	78.9	14	8	181	Yes
SRA stem-loop-interacting RNA-binding protein, mitochondrial	SLIRP_HUMAN	Q9GZT3	SLIRP	12.3	80	9	181	Yes
Serine--tRNA ligase, mitochondrial	SYSM_HUMAN	Q9NP81	SARS2	58.2	33	15	181	Yes
Neutrophil defensin 1	DEF1_HUMAN	P59665	DEFA1	10.2	20	4	180	Yes
Fructosamine-3-kinase	FN3K_HUMAN	Q9H479	FN3K	35.1	35	9	180	Yes
Retinal dehydrogenase 2	AL1A2_HUMAN	O94788	ALDH1A2	56.7	18	6	179	No
Importin subunit alpha-3	IMA3_HUMAN	O00629	KPNA4	57.9	31	6	178	Yes
60S acidic ribosomal protein P1	RLA1_HUMAN	P05386	RPLP1	11.5	67	2	178	Yes
Glutathione synthetase	GSHB_HUMAN	P48637	GSS	52.4	35	15	178	Yes
28S ribosomal protein S29, mitochondrial	RT29_HUMAN	P51398	DAP3	45.5	35	13	178	Yes
DnaJ homolog subfamily A member 2	DNJA2_HUMAN	O60884	DNAJA2	45.7	23	9	177	Yes
Dynactin subunit 3	DCTN3_HUMAN	O75935	DCTN3	21.1	44	9	177	Yes

Scavenger receptor cysteine-rich type 1 protein M130	C163A_HUMAN	Q86VB7	CD163	125.4	27	24	177	No
Saccharopine dehydrogenase-like oxidoreductase	SCPDL_HUMAN	Q8NBX0	SCCPDH	47.1	27	10	177	Yes
Ras-related protein Rab-21	RAB21_HUMAN	Q9UL25	RAB21	24.3	31	7	177	Yes
Endophilin-B1	SHLB1_HUMAN	Q9Y371	SH3GLB1	40.8	44	15	177	Yes
Ragulator complex protein LAMTOR5	LTOR5_HUMAN	O43504	LAMTOR5	9.6	81	4	176	Yes
Cystatin-B	CYTB_HUMAN	P04080	CSTB	11.1	61	5	176	Yes
Reticulocalbin-1	RCN1_HUMAN	Q15293	RCN1	38.9	28	7	175	Yes
Heat shock 70 kDa protein 4L	HS74L_HUMAN	O95757	HSPA4L	94.5	30	17	174	No
LIM and SH3 domain protein 1	LASP1_HUMAN	Q14847	LASP1	29.7	36	11	174	Yes
ATP-dependent (S)-NAD(P)H-hydrate dehydratase	NNRD_HUMAN	Q8IW45	NAXD	36.6	44	12	174	Yes
Ethylmalonyl-CoA decarboxylase	ECHD1_HUMAN	Q9NTX5	ECHDC1	33.7	49	11	174	Yes
HLA class I histocompatibility antigen, C alpha chain	HLAC_HUMAN	P10321	HLA-C	40.6	38	2	173	No
Glyoxalase domain-containing protein 4	GLOD4_HUMAN	Q9HC38	GLOD4	34.8	47	12	173	Yes
Tubulin-specific chaperone A	TBCA_HUMAN	O75347	TBCA	12.8	71	13	172	Yes
Heat shock protein 105 kDa	HS105_HUMAN	Q92598	HSPH1	96.8	21	11	172	No
Iron-sulfur cluster assembly enzyme ISCU, mitochondrial	ISCU_HUMAN	Q9H1K1	ISCU	18	60	11	172	Yes
Heterogeneous nuclear ribonucleoprotein D-like	HNRDL_HUMAN	O14979	HNRNPDL	46.4	19	8	171	Yes
Valine--tRNA ligase	SYVC_HUMAN	P26640	VARS1	140.4	16	17	171	Yes
Adenylosuccinate synthetase isozyme 1	PURA1_HUMAN	Q8N142	ADSS1	50.2	37	15	170	Yes
Coiled-coil domain-containing protein 90B, mitochondrial	CC90B_HUMAN	Q9GZT6	CCDC90B	29.5	41	12	170	Yes
Guanine nucleotide-binding protein G	GNAO_HUMAN	P09471	GNAO1	40	36	8	169	No
40S ribosomal protein S10	RS10_HUMAN	P46783	RPS10	18.9	48	9	169	Yes
Vasodilator-stimulated phosphoprotein	VASP_HUMAN	P50552	VASP	39.8	39	12	169	Yes
Actin-related protein 2	ARP2_HUMAN	P61160	ACTR2	44.7	27	9	169	Yes
Regulator of microtubule dynamics protein 1	RMD1_HUMAN	Q96DB5	RMDN1	35.8	40	15	169	Yes
Histone H1.0	H10_HUMAN	P07305	H1-0	20.9	23	4	168	Yes
Glutaredoxin-related protein 5, mitochondrial	GLRX5_HUMAN	Q86SX6	GLRX5	16.6	34	6	168	Yes
Myoferlin	MYOF_HUMAN	Q9NZM1	MYOF	234.6	15	23	168	No
RNA-splicing ligase RtcB homolog	RTCB_HUMAN	Q9Y3I0	RTCB	55.2	47	20	168	Yes
N(G),N(G)-dimethylarginine dimethylaminohydrolase 1	DDAH1_HUMAN	O94760	DDAH1	31.1	36	7	167	Yes
40S ribosomal protein S12	RS12_HUMAN	P25398	RPS12	14.5	58	7	167	Yes
Rho GDP-dissociation inhibitor 1	GDIR1_HUMAN	P52565	ARHGDIA	23.2	41	7	167	Yes
Dehydrogenase/reductase SDR family member 4	DHRS4_HUMAN	Q9BTZ2	DHRS4	29.5	41	11	167	Yes
Enoyl-[acyl-carrier-protein] reductase, mitochondrial	MECR_HUMAN	Q9BV79	MECR	40.4	31	10	167	Yes
Membrane-associated progesterone receptor component 2	PGRC2_HUMAN	O15173	PGRMC2	23.8	48	8	166	Yes
Mitochondrial import inner membrane translocase subunit Tim8 A	TIM8A_HUMAN	O60220	TIMM8A	11	56	5	166	Yes

Glutathione peroxidase 1	GPX1_HUMAN	P07203	GPX1	22.1	51	8	166	Yes
Ras-related protein Rab-6A	RAB6A_HUMAN	P20340	RAB6A	23.6	49	5	166	Yes
Septin-8	SEPT8_HUMAN	Q92599	SEPTIN8	55.7	30	8	166	No
Fumarylacetoacetate hydrolase domain-containing protein 2A	FAH2A_HUMAN	Q96GK7	FAHD2A	34.6	42	11	166	Yes
Histone H1.5	H15_HUMAN	P16401	H1-5	22.6	28	5	165	No
Matrix-remodeling-associated protein 7	MXRA7_HUMAN	P84157	MXRA7	21.5	41	7	165	Yes
Citramalyl-CoA lyase, mitochondrial	CLYBL_HUMAN	Q8N0X4	CLYBL	37.3	45	13	165	Yes
Corrinoid adenosyltransferase	MMAB_HUMAN	Q96EY8	MMAB	27.4	38	8	165	Yes
Protein NipSnap homolog 1	NIPS1_HUMAN	Q9BPW8	NIPSNAP1	33.3	22	5	165	Yes
Haloacid dehalogenase-like hydrolase domain-containing 5	HDHD5_HUMAN	Q9BXW7	HDHD5	46.3	39	13	165	Yes
Pterin-4-alpha-carbinolamine dehydratase 2	PHS2_HUMAN	Q9H0N5	PCBD2	14.4	54	6	165	Yes
Syntaxin-7	STX7_HUMAN	O15400	STX7	29.8	43	9	164	No
Persulfide dioxygenase ETHE1, mitochondrial	ETHE1_HUMAN	O95571	ETHE1	27.9	52	9	164	Yes
Complement component C9	CO9_HUMAN	P02748	C9	63.1	26	13	164	Yes
ATP-dependent 6-phosphofructokinase, liver type	PFKAL_HUMAN	P17858	PFKL	85	15	4	164	Yes
Histone-binding protein RBBP7	RBBP7_HUMAN	Q16576	RBBP7	47.8	35	5	163	Yes
Pterin-4-alpha-carbinolamine dehydratase	PHS_HUMAN	P61457	PCBD1	12	51	4	162	Yes
Prefoldin subunit 6	PFD6_HUMAN	O15212	PFDN6	14.6	40	9	161	Yes
Extracellular superoxide dismutase [Cu-Zn]	SODE_HUMAN	P08294	SOD3	25.8	60	10	161	Yes
Histone-binding protein RBBP4	RBBP4_HUMAN	Q09028	RBBP4	47.6	29	3	161	Yes
Calcium/calmodulin-dependent protein kinase type II subunit beta	KCC2B_HUMAN	Q13554	CAMK2B	72.6	29	8	161	No
Keratin, type I cuticular Ha1	K1H1_HUMAN	Q15323	KRT31	47.2	44	4	161	No
Hornerin	HORN_HUMAN	Q86YZ3	HRNR	282.2	12	13	161	No
Protein NDRG4	NDRG4_HUMAN	Q9ULP0	NDRG4	38.4	41	9	161	Yes
NADH dehydrogenase [ubiquinone] 1 subunit C2	NDUC2_HUMAN	O95298	NDUFC2	14.2	30	5	160	Yes
Keratin, type I cytoskeletal 13	K1C13_HUMAN	P13646	KRT13	49.6	28	7	160	No
V-type proton ATPase catalytic subunit A	VATA_HUMAN	P38606	ATP6V1A	68.3	27	13	159	Yes
Prefoldin subunit 3	PFD3_HUMAN	P61758	VBP1	22.6	41	9	159	Yes
ATP-dependent RNA helicase A	DHX9_HUMAN	Q08211	DHX9	140.9	21	23	159	Yes
MICOS complex subunit MIC13	MIC13_HUMAN	Q5XKP0	MICOS13	13.1	61	6	159	Yes
Iron-sulfur protein NUBPL	NUBPL_HUMAN	Q8TB37	NUBPL	34.1	37	9	159	Yes
Alpha-parvin	PARVA_HUMAN	Q9NVD7	PARVA	42.2	25	12	159	Yes
Mitochondrial carnitine/acylcarnitine carrier protein	MCAT_HUMAN	O43772	SLC25A20	32.9	34	10	158	Yes
Retinol-binding protein 4	RET4_HUMAN	P02753	RBP4	23	36	6	158	Yes
GDH/6PGL endoplasmic bifunctional protein	G6PE_HUMAN	O95479	H6PD	88.8	28	18	157	Yes
Coiled-coil domain-containing protein 58	CCD58_HUMAN	Q4VC31	CCDC58	16.6	52	7	157	Yes

Acylpyruvase FAHD1, mitochondrial	FAHD1_HUMAN	Q6P587	FAHD1	24.8	62	10	157	Yes
Prostaglandin F2 receptor negative regulator	FPRP_HUMAN	Q9P2B2	PTGFRN	98.5	19	14	157	Yes
26S proteasome non-ATPase regulatory subunit 6	PSMD6_HUMAN	Q15008	PSMD6	45.5	31	10	156	Yes
Xaa-Pro aminopeptidase 3	XPP3_HUMAN	Q9NQH7	XPNPEP3	57	26	9	156	Yes
3-oxoacyl-[acyl-carrier-protein] synthase, mitochondrial	OXSM_HUMAN	Q9NWU1	OXSM	48.8	56	14	156	Yes
Cytoplasmic dynein 1 light intermediate chain 2	DC1L2_HUMAN	O43237	DYNC1LI2	54.1	37	15	155	Yes
Carbonyl reductase [NADPH] 1	CBR1_HUMAN	P16152	CBR1	30.4	68	14	155	Yes
TOM1-like protein 2	TM1L2_HUMAN	Q6ZVM7	TOM1L2	55.5	39	17	155	Yes
Spermatogenesis-associated protein 20	SPT20_HUMAN	Q8TB22	SPATA20	87.8	24	16	155	Yes
Protein preY, mitochondrial	PREY_HUMAN	Q96I23	PYURF	12.6	59	4	155	Yes
Fibulin-5	FBLN5_HUMAN	Q9UBX5	FBLN5	50.1	29	9	155	Yes
Long-chain fatty acid transport protein 6	S27A6_HUMAN	Q9Y2P4	SLC27A6	70.1	26	13	155	Yes
Alpha-adducin	ADDA_HUMAN	P35611	ADD1	80.9	29	15	154	Yes
Microfibril-associated glycoprotein 4	MFAP4_HUMAN	P55083	MFAP4	28.6	40	5	154	Yes
Rab GTPase-binding effector protein 1	RABE1_HUMAN	Q15276	RABEP1	99.2	20	16	154	Yes
26S proteasome non-ATPase regulatory subunit 12	PSD12_HUMAN	O00232	PSMD12	52.9	34	15	153	Yes
26S proteasome non-ATPase regulatory subunit 14	PSDE_HUMAN	O00487	PSMD14	34.6	34	10	153	Yes
Leukocyte elastase inhibitor	ILEU_HUMAN	P30740	SERPINB1	42.7	36	13	153	Yes
Protein S100-A11	S10AB_HUMAN	P31949	S100A11	11.7	31	4	153	Yes
Glutaredoxin-1	GLRX1_HUMAN	P35754	GLRX	11.8	56	6	153	Yes
Serine/threonine-protein phosphatase 2A 56 kDa regulatory subunit alpha isoform	2A5A_HUMAN	Q15172	PPP2R5A	56.2	17	8	153	Yes
Obscurin	OBSCN_HUMAN	Q5VST9	OBSCN	867.9	7	35	153	Yes
Junctophilin-2	JPH2_HUMAN	Q9BR39	JPH2	74.2	25	10	153	Yes
Tenascin	TENA_HUMAN	P24821	TNC	240.7	14	23	152	Yes
Eukaryotic translation initiation factor 3 subunit I	EIF3I_HUMAN	Q13347	EIF3I	36.5	42	10	152	Yes
Protein NDRG1	NDRG1_HUMAN	Q92597	NDRG1	42.8	46	9	152	Yes
Mitochondrial import inner membrane translocase subunit Tim8 B	TIM8B_HUMAN	Q9Y5J9	TIMM8B	9.3	45	4	152	Yes
40S ribosomal protein S5	RS5_HUMAN	P46782	RPS5	22.9	37	9	151	Yes
Serine/arginine-rich splicing factor 1	SRSF1_HUMAN	Q07955	SRSF1	27.7	36	8	151	Yes
Membrane primary amine oxidase	AOC3_HUMAN	Q16853	AOC3	84.6	16	11	151	Yes
Acyl-protein thioesterase 1	LYPA1_HUMAN	O75608	LYPLA1	24.7	37	6	150	Yes
Calpain small subunit 1	CPNS1_HUMAN	P04632	CAPNS1	28.3	50	8	150	Yes
Keratin, type I cytoskeletal 19	K1C19_HUMAN	P08727	KRT19	44.1	19	2	150	No
Cytochrome c-type heme lyase	CCHL_HUMAN	P53701	HCCS	30.6	36	14	150	Yes
NADH dehydrogenase [ubiquinone] flavoprotein 3, mitochondrial	NDUV3_HUMAN	P56181	NDUFV3	11.9	30	5	150	Yes

Prefoldin subunit 2	PFD2_HUMAN	Q9UHV9	PFDN2	16.6	54	8	150	Yes
B-cell receptor-associated protein 31	BAP31_HUMAN	P51572	BCAP31	28	35	11	149	Yes
Protein SET	SET_HUMAN	Q01105	SET	33.5	19	6	149	Yes
Myosin light chain kinase 3	MYLK3_HUMAN	Q32MK0	MYLK3	88.3	29	16	149	Yes
Coiled-coil domain-containing protein 47	CCD47_HUMAN	Q96A33	CCDC47	55.8	31	13	149	Yes
39S ribosomal protein L46, mitochondrial	RM46_HUMAN	Q9H2W6	MRPL46	31.7	37	9	149	Yes
Protein S100-A6	S10A6_HUMAN	P06703	S100A6	10.2	36	5	148	Yes
Stathmin	STMN1_HUMAN	P16949	STMN1	17.3	54	9	148	Yes
COP9 signalosome complex subunit 1	CSN1_HUMAN	Q13098	GPS1	55.5	26	12	148	Yes
Ryanodine receptor 2	RYR2_HUMAN	Q92736	RYR2	564.2	9	31	148	Yes
Medium-chain acyl-CoA ligase ACSF2, mitochondrial	ACSF2_HUMAN	Q96CM8	ACSF2	68.1	23	11	148	Yes
Transmembrane protein 109	TM109_HUMAN	Q9BVC6	TMEM109	26.2	13	5	148	Yes
Nicotinamide phosphoribosyltransferase	NAMPT_HUMAN	P43490	NAMPT	55.5	40	15	147	Yes
Interleukin enhancer-binding factor 2	ILF2_HUMAN	Q12905	ILF2	43	18	7	147	Yes
C->U-editing enzyme APOBEC-2	ABEC2_HUMAN	Q9Y235	APOBEC2	25.7	59	9	147	Yes
Peptidyl-prolyl cis-trans isomerase-like 1	PPIL1_HUMAN	Q9Y3C6	PPIL1	18.2	42	6	147	Yes
Paralemmin-1	PALM_HUMAN	O75781	PALM	42.1	20	8	146	Yes
Macrophage migration inhibitory factor	MIF_HUMAN	P14174	MIF	12.5	37	4	146	Yes
Cytochrome c oxidase subunit 7A2, mitochondrial	CX7A2_HUMAN	P14406	COX7A2	9.4	30	3	146	Yes
Integrin alpha-1	ITA1_HUMAN	P56199	ITGA1	130.8	11	12	146	No
Ras-related protein Rab-8A	RAB8A_HUMAN	P61006	RAB8A	23.7	29	2	146	No
Ubiquitin-associated protein 2-like	UBP2L_HUMAN	Q14157	UBAP2L	114.5	17	13	146	Yes
[Pyruvate dehydrogenase (acetyl-transferring)] kinase isozyme 1, mitochondrial	PDK1_HUMAN	Q15118	PDK1	49.2	45	13	146	Yes
Ras-related protein Rab-8B	RAB8B_HUMAN	Q92930	RAB8B	23.6	35	3	146	No
Clustered mitochondria protein homolog	CLU_HUMAN	O75153	CLUH	146.6	16	16	145	Yes
Peptidyl-glycine alpha-amidating monooxygenase	AMD_HUMAN	P19021	PAM	108.3	14	12	145	Yes
Keratin, type I cuticular Ha3-II	KT33B_HUMAN	Q14525	KRT33B	46.2	45	4	145	No
Putative deoxyribonuclease TATDN1	TATD1_HUMAN	Q6P1N9	TATDN1	33.6	46	16	145	Yes
Trimethyllysine dioxygenase, mitochondrial	TMLH_HUMAN	Q9NVH6	TMLHE	49.5	32	12	145	Yes
NADH dehydrogenase [ubiquinone] 1 beta subcomplex subunit 5, mitochondrial	NDUB5_HUMAN	O43674	NDUFB5	21.7	28	5	144	Yes
Insulin-like growth factor-binding protein 7	IBP7_HUMAN	Q16270	IGFBP7	29.1	49	10	144	Yes
GTP-binding protein SAR1a	SAR1A_HUMAN	Q9NR31	SAR1A	22.4	36	6	144	Yes
Endoplasmic reticulum aminopeptidase 1	ERAP1_HUMAN	Q9NZ08	ERAP1	107.2	20	16	144	Yes
ATPase family AAA domain-containing protein 1	ATAD1_HUMAN	Q8NBU5	ATAD1	40.7	32	7	143	Yes

Protein ABHD11	ABHDB_HUMAN	Q8NFV4	ABHD11	34.7	30	8	143	Yes
Guanine nucleotide-binding protein G(I)/G(S)/G(O) subunit gamma-12	GBG12_HUMAN	Q9UBI6	GNG12	8	71	5	143	Yes
Ras-related protein Rab-5B	RAB5B_HUMAN	P61020	RAB5B	23.7	43	5	142	No
LEM domain-containing protein 2	LEMD2_HUMAN	Q8NC56	LEMD2	56.9	26	12	142	Yes
RelA-associated inhibitor	IASPP_HUMAN	Q8WUF5	PPP1R13L	89	31	18	142	Yes
Multimerin-2	MMRN2_HUMAN	Q9H8L6	MMRN2	104.3	21	14	142	Yes
Chloride intracellular channel protein 1	CLIC1_HUMAN	O00299	CLIC1	26.9	60	12	141	Yes
60S ribosomal protein L13	RL13_HUMAN	P26373	RPL13	24.2	30	8	141	Yes
Protein SEC13 homolog	SEC13_HUMAN	P55735	SEC13	35.5	22	6	141	Yes
Raftlin	RFTN1_HUMAN	Q14699	RFTN1	63.1	28	12	141	No
COP9 signalosome complex subunit 3	CSN3_HUMAN	Q9UNS2	COPS3	47.8	39	13	141	Yes
Histidine-rich glycoprotein	HRG_HUMAN	P04196	HRG	59.5	36	14	140	Yes
Putative transferase CAF17, mitochondrial	CAF17_HUMAN	Q5T440	IBA57	38.1	37	9	140	Yes
Ras-related protein Rab-18	RAB18_HUMAN	Q9NP72	RAB18	23	46	8	140	Yes
Xaa-Pro aminopeptidase 1	XPP1_HUMAN	Q9NQW7	XPNPEP1	69.9	23	12	140	Yes
Beta-hexosaminidase subunit alpha	HEXA_HUMAN	P06865	HEXA	60.7	22	10	139	Yes
40S ribosomal protein S3	RS3_HUMAN	P23396	RPS3	26.7	50	13	138	Yes
Ubiquitin-conjugating enzyme E2 N	UBE2N_HUMAN	P61088	UBE2N	17.1	59	8	138	Yes
Splicing factor 3B subunit 2	SF3B2_HUMAN	Q13435	SF3B2	100.2	24	16	138	Yes
Thioredoxin, mitochondrial	THIOM_HUMAN	Q99757	TXN2	18.4	42	5	138	Yes
Tropomodulin-3	TMOD3_HUMAN	Q9NYL9	TMOD3	39.6	41	11	138	Yes
Complement component C7	CO7_HUMAN	P10643	C7	93.5	24	16	137	No
Coatomer subunit zeta-1	COPZ1_HUMAN	P61923	COPZ1	20.2	27	4	137	Yes
Spectrin alpha chain, erythrocytic 1	SPTA1_HUMAN	P02549	SPTA1	279.8	16	32	136	No
Complement factor H	CFAH_HUMAN	P08603	CFH	139	20	18	136	Yes
Coatomer subunit delta	COPD_HUMAN	P48444	ARCN1	57.2	28	12	136	Yes
Septin-6	SEPT6_HUMAN	Q14141	SEPTIN6	49.7	24	2	136	No
Protein transport protein Sec23A	SC23A_HUMAN	Q15436	SEC23A	86.1	22	13	136	Yes
Chloride intracellular channel protein 4	CLIC4_HUMAN	Q9Y696	CLIC4	28.8	40	8	136	Yes
NADH dehydrogenase [ubiquinone] 1 alpha subcomplex assembly factor 8	NDUF8_HUMAN	A1L188	NDUFAF8	7.8	45	4	135	Yes
Calmegin	CLGN_HUMAN	O14967	CLGN	70	22	7	135	Yes
Bifunctional glutamate/proline--tRNA ligase	SYEP_HUMAN	P07814	EPRS1	170.5	21	25	135	Yes
C-1-tetrahydrofolate synthase, cytoplasmic	C1TC_HUMAN	P11586	MTHFD1	101.5	20	16	135	Yes
Beta-2-microglobulin	B2MG_HUMAN	P61769	B2M	13.7	38	4	135	Yes

Eukaryotic translation initiation factor 3 subunit K	EIF3K_HUMAN	Q9UBQ5	EIF3K	25	29	6	135	Yes
NADP-dependent malic enzyme	MAOX_HUMAN	P48163	ME1	64.1	17	9	134	Yes
Aminomethyltransferase, mitochondrial	GCST_HUMAN	P48728	AMT	43.9	29	12	134	Yes
Dynamin-2	DYN2_HUMAN	P50570	DNM2	98	21	16	134	Yes
Trans-Golgi network integral membrane protein 2	TGON2_HUMAN	O43493	TGOLN2	51	15	6	133	Yes
Barrier-to-autointegration factor	BAF_HUMAN	O75531	BANF1	10.1	72	7	133	Yes
40S ribosomal protein S2	RS2_HUMAN	P15880	RPS2	31.3	26	7	133	Yes
Ras-related protein Rab-35	RAB35_HUMAN	Q15286	RAB35	23	21	2	133	No
Blood vessel epicardial substance	POPD1_HUMAN	Q8NE79	BVES	41.4	39	12	133	Yes
Aspartate--tRNA ligase, cytoplasmic	SYDC_HUMAN	P14868	DARS1	57.1	38	16	132	Yes
60S ribosomal protein L30	RL30_HUMAN	P62888	RPL30	12.8	67	7	132	Yes
Keratin, type II cytoskeletal 4	K2C4_HUMAN	P19013	KRT4	57.3	25	7	131	No
Heterogeneous nuclear ribonucleoprotein A3	ROA3_HUMAN	P51991	HNRNPA3	39.6	29	10	131	Yes
Astrocytic phosphoprotein PEA-15	PEA15_HUMAN	Q15121	PEA15	15	48	6	131	Yes
Aspartate--tRNA ligase, mitochondrial	SYDM_HUMAN	Q6PI48	DARS2	73.5	27	16	131	Yes
Coiled-coil domain-containing protein 141	CC141_HUMAN	Q6ZP82	CCDC141	166.2	14	18	131	Yes
Protein FAM162A	F162A_HUMAN	Q96A26	FAM162A	17.3	40	7	131	Yes
COP9 signalosome complex subunit 8	CSN8_HUMAN	Q99627	COPS8	23.2	35	5	131	Yes
Heat shock protein beta-8	HSPB8_HUMAN	Q9UJY1	HSPB8	21.6	37	11	131	Yes
Cytochrome c oxidase subunit 7A1, mitochondrial	CX7A1_HUMAN	P24310	COX7A1	9.1	34	4	130	Yes
Mitogen-activated protein kinase 1	MK01_HUMAN	P28482	MAPK1	41.4	33	8	130	Yes
60S ribosomal protein L12	RL12_HUMAN	P30050	RPL12	17.8	64	8	130	Yes
Cadherin-5	CADH5_HUMAN	P33151	CDH5	87.5	13	9	130	Yes
Serine hydroxymethyltransferase, mitochondrial	GLYM_HUMAN	P34897	SHMT2	56	27	11	130	Yes
Heterogeneous nuclear ribonucleoprotein F	HNRPF_HUMAN	P52597	HNRNPF	45.6	18	3	130	No
NPC intracellular cholesterol transporter 2	NPC2_HUMAN	P61916	NPC2	16.6	52	9	130	Yes
Protein phosphatase 1 regulatory subunit 12A	MYPT1_HUMAN	O14974	PPP1R12A	115.2	20	17	129	Yes
Splicing factor 3B subunit 1	SF3B1_HUMAN	O75533	SF3B1	145.7	11	9	129	Yes
Dihydroorotate dehydrogenase (quinone), mitochondrial	PYRD_HUMAN	Q02127	DHODH	42.8	52	14	129	Yes
Aminoacyl tRNA synthase complex-interacting multifunctional protein 1	AIMP1_HUMAN	Q12904	AIMP1	34.3	47	11	129	Yes
Proteasomal ubiquitin receptor ADRM1	ADRM1_HUMAN	Q16186	ADRM1	42.1	15	5	129	Yes
GTP-binding protein SAR1b	SAR1B_HUMAN	Q9Y6B6	SAR1B	22.4	39	6	129	Yes
Coagulation factor IX	FA9_HUMAN	P00740	F9	51.7	18	7	128	Yes
Cystatin-C	CYTC_HUMAN	P01034	CST3	15.8	37	6	128	Yes
Syntaxin-4	STX4_HUMAN	Q12846	STX4	34.2	28	7	128	Yes

Protein FAM177A1	F177A_HUMAN	Q8N128	FAM177A1	23.7	38	6	128	Yes
Probable serine carboxypeptidase CPVL	CPVL_HUMAN	Q9H3G5	CPVL	54.1	19	9	128	Yes
Glutaminase kidney isoform, mitochondrial	GLSK_HUMAN	O94925	GLS	73.4	27	13	127	Yes
Low molecular weight phosphotyrosine protein phosphatase	PPAC_HUMAN	P24666	ACP1	18	63	9	127	Yes
Heterogeneous nuclear ribonucleoprotein A/B	ROAA_HUMAN	Q99729	HNRNPAB	36.2	25	7	127	Yes
Optineurin	OPTN_HUMAN	Q96CV9	OPTN	65.9	24	12	126	Yes
Golgi reassembly-stacking protein 2	GORS2_HUMAN	Q9H8Y8	GORASP2	47.1	30	9	126	Yes
Metaxin-1	MTX1_HUMAN	Q13505	MTX1	51.4	26	10	125	Yes
Laminin subunit alpha-5	LAMA5_HUMAN	O15230	LAMA5	399.5	7	19	125	Yes
Ubiquitin carboxyl-terminal hydrolase isozyme L1	UCHL1_HUMAN	P09936	UCHL1	24.8	62	12	125	Yes
Ectonucleoside triphosphate diphosphohydrolase 1	ENTP1_HUMAN	P49961	ENTPD1	57.9	19	9	125	Yes
Thymosin beta-10	TYB10_HUMAN	P63313	TMSB10	5	48	3	125	No
Non-POU domain-containing octamer-binding protein	NONO_HUMAN	Q15233	NONO	54.2	30	13	125	Yes
Syntaxin-binding protein 3	STXB3_HUMAN	O00186	STXBP3	67.7	26	14	124	Yes
Actin-like protein 6A	ACL6A_HUMAN	O96019	ACTL6A	47.4	28	9	124	Yes
Dolichyl-diphosphooligosaccharide--protein glycosyltransferase 48 kDa subunit	OST48_HUMAN	P39656	DDOST	50.8	14	7	124	Yes
Dynein light chain 1, cytoplasmic	DYL1_HUMAN	P63167	DYNLL1	10.4	65	4	124	No
Fascin	FSCN1_HUMAN	Q16658	FSCN1	54.5	24	11	124	Yes
Ubiquinol-cytochrome-c reductase complex assembly factor 2	UQCC2_HUMAN	Q9BRT2	UQCC2	14.9	72	9	124	Yes
Epimerase family protein SDR39U1	D39U1_HUMAN	Q9NRG7	SDR39U1	34.7	27	6	124	Yes
Dynactin subunit 4	DCTN4_HUMAN	Q9UJW0	DCTN4	52.3	31	9	124	Yes
CD44 antigen	CD44_HUMAN	P16070	CD44	81.5	6	5	123	Yes
Atlastin-3	ATLA3_HUMAN	Q6DD88	ATL3	60.5	25	11	123	Yes
Protein enabled homolog	ENAH_HUMAN	Q8N8S7	ENAH	66.5	13	7	123	Yes
Pre-B-cell leukemia transcription factor-interacting protein 1	PBIP1_HUMAN	Q96AQ6	PBXIP1	80.6	25	17	123	No
Ras-related protein Rab-6B	RAB6B_HUMAN	Q9NRW1	RAB6B	23.4	38	2	123	No
60S ribosomal protein L17	RL17_HUMAN	P18621	RPL17	21.4	36	6	122	No
Progranulin	GRN_HUMAN	P28799	GRN	63.5	22	8	122	Yes
ADP-ribose pyrophosphatase, mitochondrial	NUDT9_HUMAN	Q9BW91	NUDT9	39.1	34	12	122	Yes
Proliferation-associated protein 2G4	PA2G4_HUMAN	Q9UQ80	PA2G4	43.8	37	12	122	No
Neuropilin-1	NRP1_HUMAN	O14786	NRP1	103.1	20	13	121	Yes
Arginine-hydroxylase NDUFAF5, mitochondrial	NDUF5_HUMAN	Q5TEU4	NDUFAF5	38.9	27	8	121	Yes
Carbonyl reductase family member 4	CBR4_HUMAN	Q8N4T8	CBR4	25.3	53	10	121	Yes
ATP synthase mitochondrial F1 complex assembly factor 2	ATPF2_HUMAN	Q8N5M1	ATPAF2	32.8	42	10	121	Yes
Proteasome activator complex subunit 2	PSME2_HUMAN	Q9UL46	PSME2	27.4	33	7	120	Yes

Endoglin	EGLN_HUMAN	P17813	ENG	70.5	14	7	119	Yes
Eukaryotic initiation factor 4A-III	IF4A3_HUMAN	P38919	EIF4A3	46.8	23	6	119	Yes
Nuclear transport factor 2	NTF2_HUMAN	P61970	NUTF2	14.5	51	4	119	Yes
Ras GTPase-activating protein-binding protein 1	G3BP1_HUMAN	Q13283	G3BP1	52.1	25	8	119	Yes
Microtubule-associated protein RP/EB family member 1	MARE1_HUMAN	Q15691	MAPRE1	30	36	7	119	Yes
HLA class II histocompatibility antigen, DRB1 beta chain	DRB1_HUMAN	P01911	HLA-DRB1	30	35	2	119	Yes
Dolichyl-diphosphooligosaccharide--protein glycosyltransferase subunit 2	RPN2_HUMAN	P04844	RPN2	69.2	28	13	118	Yes
SH3 domain-binding glutamic acid-rich protein	SH3BG_HUMAN	P55822	SH3BGR	26.1	26	6	118	Yes
Eukaryotic translation initiation factor 5A-1	IF5A1_HUMAN	P63241	EIF5A	16.8	25	5	118	Yes
SPARC-like protein 1	SPRL1_HUMAN	Q14515	SPARCL1	75.2	32	14	118	Yes
Echinoderm microtubule-associated protein-like 1	EMAL1_HUMAN	O00423	EML1	89.8	14	10	117	Yes
Ras-related protein Rab-5C	RAB5C_HUMAN	P51148	RAB5C	23.5	44	6	117	Yes
Cx9C motif-containing protein 4	CMC4_HUMAN	P56277	CMC4	7.7	78	7	117	Yes
Receptor of activated protein C kinase 1	RACK1_HUMAN	P63244	RACK1	35.1	41	12	117	Yes
Mitochondrial calcium uniporter regulator 1	MCUR1_HUMAN	Q96AQ8	MCUR1	39.7	23	9	117	Yes
Haloacid dehalogenase-like hydrolase domain-containing protein 3	HDHD3_HUMAN	Q9BSH5	HDHD3	28	41	7	117	Yes
Erlin-2	ERLN2_HUMAN	O94905	ERLIN2	37.8	29	11	116	Yes
Chromobox protein homolog 1	CBX1_HUMAN	P83916	CBX1	21.4	46	10	116	Yes
Protein sel-1 homolog 1	SE1L1_HUMAN	Q9UBV2	SEL1L	88.7	25	15	116	No
Complex I intermediate-associated protein 30, mitochondrial	CIA30_HUMAN	Q9Y375	NDUFAF1	37.7	40	9	116	Yes
V-type proton ATPase subunit B, brain isoform	VATB2_HUMAN	P21281	ATP6V1B2	56.5	35	14	115	Yes
Bis(5'-adenosyl)-triphosphatase	FHIT_HUMAN	P49789	FHIT	16.8	69	7	115	Yes
Coatomer subunit alpha	COPA_HUMAN	P53621	COPA	138.3	17	15	115	Yes
KN motif and ankyrin repeat domain-containing protein 2	KANK2_HUMAN	Q63ZY3	KANK2	91.1	23	15	115	Yes
Valacyclovir hydrolase	BPHL_HUMAN	Q86WA6	BPHL	32.5	23	7	115	Yes
Ubiquitin recognition factor in ER-associated degradation protein 1	UFD1_HUMAN	Q92890	UFD1	34.5	29	7	115	Yes
Ubiquitin thioesterase OTUB1	OTUB1_HUMAN	Q96FW1	OTUB1	31.3	35	9	115	Yes
39S ribosomal protein L11, mitochondrial	RM11_HUMAN	Q9Y3B7	MRPL11	20.7	64	12	115	Yes
Thioredoxin-like protein 1	TXNL1_HUMAN	O43396	TXNL1	32.2	53	12	114	Yes
Keratin, type I cuticular Ha4	KRT34_HUMAN	O76011	KRT34	49.4	29	2	114	No
60S ribosomal protein L7a	RL7A_HUMAN	P62424	RPL7A	30	37	9	114	Yes
Keratin, type II cytoskeletal 1b	K2C1B_HUMAN	Q7Z794	KRT77	61.9	13	4	114	No
Cytochrome c oxidase assembly factor 3 homolog, mitochondrial	COA3_HUMAN	Q9Y2R0	COA3	11.7	38	5	114	Yes
Serum amyloid A-1 protein	SAA1_HUMAN	P0DJI8	SAA1	13.5	51	3	113	Yes

Ras-related protein Ral-B	RALB_HUMAN	P11234	RALB	23.4	36	4	113	Yes
V-type proton ATPase subunit E 1	VATE1_HUMAN	P36543	ATP6V1E1	26.1	34	9	113	Yes
Cytochrome c oxidase assembly factor 4 homolog, mitochondrial	COA4_HUMAN	Q9NYJ1	COA4	10.1	66	5	113	Yes
SEC23-interacting protein	S23IP_HUMAN	Q9Y6Y8	SEC23IP	111	15	12	113	No
Apolipoprotein C-II	APOC2_HUMAN	P02655	APOC2	11.3	29	3	112	Yes
Core histone macro-H2A.2	H2AW_HUMAN	Q9P0M6	MACROH2A2	40	26	6	112	Yes
Ubiquilin-2	UBQL2_HUMAN	Q9UHD9	UBQLN2	65.7	22	6	112	Yes
Protein canopy homolog 2	CNPY2_HUMAN	Q9Y2B0	CNPY2	20.6	64	11	112	Yes
Ras-related protein R-Ras	RRAS_HUMAN	P10301	RRAS	23.5	56	7	111	Yes
Ras-related protein Ral-A	RALA_HUMAN	P11233	RALA	23.6	30	4	111	Yes
Peptidyl-prolyl cis-trans isomerase FKBP2	FKBP2_HUMAN	P26885	FKBP2	15.6	58	8	111	Yes
Deoxyuridine 5'-triphosphate nucleotidohydrolase, mitochondrial	DUT_HUMAN	P33316	DUT	26.5	35	8	111	Yes
Proteasome subunit beta type-2	PSB2_HUMAN	P49721	PSMB2	22.8	34	9	111	Yes
Dual specificity protein phosphatase 3	DUS3_HUMAN	P51452	DUSP3	20.5	51	7	111	Yes
Mitochondrial fission factor	MFF_HUMAN	Q9GZY8	MFF	38.4	35	7	111	Yes
Obg-like ATPase 1	OLA1_HUMAN	Q9NTK5	OLA1	44.7	24	9	111	Yes
Prothymosin alpha	PTMA_HUMAN	P06454	PTMA	12.2	15	5	110	Yes
3-mercaptopyruvate sulfurtransferase	THTM_HUMAN	P25325	MPST	33.2	41	9	110	Yes
Mitochondrial import inner membrane translocase subunit Tim10	TIM10_HUMAN	P62072	TIMM10	10.3	61	8	110	Yes
GTP-binding nuclear protein Ran	RAN_HUMAN	P62826	RAN	24.4	48	12	109	Yes
Mitochondrial potassium channel	MITOK_HUMAN	Q96ER9	CCDC51	45.8	23	7	109	Yes
Solute carrier family 2, facilitated glucose transporter member 4	GLUT4_HUMAN	P14672	SLC2A4	54.8	16	5	108	Yes
Protein PML	PML_HUMAN	P29590	PML	97.5	20	15	108	No
F-actin-capping protein subunit alpha-2	CAZA2_HUMAN	P47755	CAPZA2	32.9	40	8	108	Yes
Serine/threonine-protein kinase PAK 2	PAK2_HUMAN	Q13177	PAK2	58	32	12	108	Yes
Myeloid-derived growth factor	MYDGF_HUMAN	Q969H8	MYDGF	18.8	20	3	108	Yes
Nuclear migration protein nudC	NUDC_HUMAN	Q9Y266	NUDC	38.2	22	7	108	Yes
Flotillin-1	FLOT1_HUMAN	O75955	FLOT1	47.3	34	14	107	Yes
NADH dehydrogenase [ubiquinone] 1 alpha subcomplex subunit 3	NDUA3_HUMAN	O95167	NDUFA3	9.3	50	3	107	Yes
3'(2'),5'-bisphosphate nucleotidase 1	BPNT1_HUMAN	O95861	BPNT1	33.4	42	11	107	Yes
DDB1- and CUL4-associated factor 8	DCAF8_HUMAN	Q5TAQ9	DCAF8	66.8	21	10	107	Yes
Enoyl-CoA hydratase domain-containing protein 2, mitochondrial	ECHD2_HUMAN	Q86YB7	ECHDC2	31.1	35	7	107	Yes
Reticulocalbin-3	RCN3_HUMAN	Q96D15	RCN3	37.5	26	6	107	No
Pentatricopeptide repeat domain-containing protein 3, mitochondrial	PTCD3_HUMAN	Q96EY7	PTCD3	78.5	15	10	107	Yes
Resistin	RETN_HUMAN	Q9HD89	RETN	11.4	48	4	107	No

Adapter molecule crk	CRK_HUMAN	P46108	CRK	33.8	50	12	106	Yes
Vesicular integral-membrane protein VIP36	LMAN2_HUMAN	Q12907	LMAN2	40.2	44	11	106	Yes
Deaminated glutathione amidase	NIT1_HUMAN	Q86X76	NIT1	35.9	27	7	106	Yes
Keratin, type II cytoskeletal 78	K2C78_HUMAN	Q8N1N4	KRT78	56.8	8	2	106	No
Bifunctional epoxide hydrolase 2	HYES_HUMAN	P34913	EPHX2	62.6	22	9	105	Yes
60S ribosomal protein L5	RL5_HUMAN	P46777	RPL5	34.3	34	10	105	Yes
Cytochrome c oxidase copper chaperone	COX17_HUMAN	Q14061	COX17	6.9	86	6	105	Yes
Evolutionarily conserved signaling intermediate in Toll pathway, mitochondrial	ECSIT_HUMAN	Q9BQ95	ECSIT	49.1	35	10	105	Yes
Eukaryotic translation initiation factor 3 subunit F	EIF3F_HUMAN	O00303	EIF3F	37.5	20	6	104	Yes
Smoothelin	SMTN_HUMAN	P53814	SMTN	99	11	10	104	Yes
Polypyrimidine tract-binding protein 1	PTBP1_HUMAN	P26599	PTBP1	57.2	18	7	103	Yes
Protein S100-A7	S10A7_HUMAN	P31151	S100A7	11.5	39	8	103	Yes
Transmembrane emp24 domain-containing protein 10	TMEDA_HUMAN	P49755	TMED10	25	31	8	103	Yes
Guanine nucleotide-binding protein G	GNAQ_HUMAN	P50148	GNAQ	42.1	37	6	103	No
Prostaglandin reductase 2	PTGR2_HUMAN	Q8N8N7	PTGR2	38.5	23	7	103	Yes
Coatomer subunit epsilon	COPE_HUMAN	O14579	COPE	34.5	43	11	102	Yes
Apolipoprotein C-I	APOC1_HUMAN	P02654	APOC1	9.3	40	6	102	Yes
Collagen alpha-2(VI) chain	CO6A2_HUMAN	P12110	COL6A2	108.5	13	10	102	Yes
Inositol-3-phosphate synthase 1	INO1_HUMAN	Q9NPH2	ISYNA1	61	25	12	102	Yes
Tight junction protein ZO-2	ZO2_HUMAN	Q9UDY2	TJP2	133.9	17	16	102	No
Papilin	PPN_HUMAN	O95428	PAPLN	137.6	8	7	101	No
AP-2 complex subunit alpha-1	AP2A1_HUMAN	O95782	AP2A1	107.5	12	7	101	Yes
40S ribosomal protein S4, X isoform	RS4X_HUMAN	P62701	RPS4X	29.6	24	7	101	Yes
m-AAA protease-interacting protein 1, mitochondrial	MAIP1_HUMAN	Q8WWC4	MAIP1	32.5	14	4	101	Yes
EF-hand domain-containing protein D2	EFHD2_HUMAN	Q96C19	EFHD2	26.7	50	11	101	No
Beta,beta-carotene 9',10'-oxygenase	BCDO2_HUMAN	Q9BYV7	BCO2	65.6	25	12	101	Yes
DnaJ homolog subfamily B member 4	DNJB4_HUMAN	Q9UDY4	DNAJB4	37.8	42	12	101	Yes
Glucosamine-6-phosphate isomerase 1	GNPI1_HUMAN	P46926	GNPDA1	32.6	44	6	100	Yes
Histidine--tRNA ligase, mitochondrial	SYHM_HUMAN	P49590	HARS2	56.9	29	10	100	Yes
Adiponectin	ADIPO_HUMAN	Q15848	ADIPOQ	26.4	20	3	100	Yes
UPF0598 protein C8orf82	CH082_HUMAN	Q6P1X6	C8orf82	23.9	38	7	100	No
Staphylococcal nuclease domain-containing protein 1	SND1_HUMAN	Q7KZF4	SND1	101.9	26	20	100	Yes
Sialic acid synthase	SIAS_HUMAN	Q9NR45	NANS	40.3	32	9	100	Yes
Membrane-associated progesterone receptor component 1	PGRC1_HUMAN	O00264	PGRMC1	21.7	35	6	99	Yes
Peptidyl-prolyl cis-trans isomerase H	PPIH_HUMAN	O43447	PPIH	19.2	23	3	99	No

NADH dehydrogenase [ubiquinone] 1 beta subcomplex subunit 3	NDUB3_HUMAN	O43676	NDUFB3	11.4	20	3	99	Yes
EGF-containing fibulin-like extracellular matrix protein 1	FBLN3_HUMAN	Q12805	EFEMP1	54.6	27	9	99	No
[Pyruvate dehydrogenase	PDK2_HUMAN	Q15119	PDK2	46.1	26	6	99	No
Ras-related protein Rab-12	RAB12_HUMAN	Q6IQ22	RAB12	27.2	20	4	99	No
Keratin, type II cuticular Hb2	KRT82_HUMAN	Q9NSB4	KRT82	56.6	9	3	99	No
Coiled-coil-helix-coiled-coil-helix domain-containing protein 2	CHCH2_HUMAN	Q9Y6H1	CHCHD2	15.5	34	3	99	Yes
Ras-related protein Rab-4A	RAB4A_HUMAN	P20338	RAB4A	24.4	38	5	98	No
Flotillin-2	FLOT2_HUMAN	Q14254	FLOT2	47	43	16	98	No
Very-long-chain 3-oxoacyl-CoA reductase	DHB12_HUMAN	Q53GQ0	HSD17B12	34.3	30	8	98	Yes
C-Jun-amino-terminal kinase-interacting protein 4	JIP4_HUMAN	O60271	SPAG9	146.1	17	14	97	Yes
Ras-related protein Rab-5A	RAB5A_HUMAN	P20339	RAB5A	23.6	31	3	97	No
Ubiquitin-conjugating enzyme E2 variant 1	UB2V1_HUMAN	Q13404	UBE2V1	16.5	29	5	97	Yes
Ethanolamine-phosphate cytidylyltransferase	PCY2_HUMAN	Q99447	PCYT2	43.8	30	10	97	Yes
Gamma-adducin	ADDG_HUMAN	Q9UEY8	ADD3	79.1	21	11	97	No
Tryptase beta-2	TRYB2_HUMAN	P20231	TPSB2	30.5	33	7	96	Yes
Oxysterol-binding protein 1	OSBP1_HUMAN	P22059	OSBP	89.4	14	9	96	Yes
Tripeptidyl-peptidase 2	TPP2_HUMAN	P29144	TPP2	138.3	18	17	96	Yes
Serine/threonine-protein phosphatase 5	PPP5_HUMAN	P53041	PPP5C	56.8	29	13	96	Yes
Myosin regulatory light chain 2, atrial isoform	MLRA_HUMAN	Q01449	MYL7	19.4	49	7	96	Yes
Calcium/calmodulin-dependent 3',5'-cyclic nucleotide phosphodiesterase 1C	PDE1C_HUMAN	Q14123	PDE1C	80.7	13	9	96	No
Major vault protein	MVP_HUMAN	Q14764	MVP	99.3	32	22	96	No
Nuclear mitotic apparatus protein 1	NUMA1_HUMAN	Q14980	NUMA1	238.1	8	14	96	No
Scaffold attachment factor B1	SAFB1_HUMAN	Q15424	SAFB	102.6	13	8	96	Yes
ATP-dependent Clp protease proteolytic subunit, mitochondrial	CLPP_HUMAN	Q16740	CLPP	30.2	38	8	96	Yes
BTB/POZ domain-containing protein KCTD12	KCD12_HUMAN	Q96CX2	KCTD12	35.7	33	8	96	Yes
Monoglyceride lipase	MGLL_HUMAN	Q99685	MGLL	33.2	35	8	96	Yes
Serine protease HTRA2, mitochondrial	HTRA2_HUMAN	O43464	HTRA2	48.8	19	7	95	Yes
26S proteasome non-ATPase regulatory subunit 8	PSMD8_HUMAN	P48556	PSMD8	39.6	29	9	95	Yes
COP9 signalosome complex subunit 6	CSN6_HUMAN	Q7L5N1	COPS6	36.1	40	10	95	Yes
[F-actin]-monooxygenase MICAL3	MICA3_HUMAN	Q7RTP6	MICAL3	224.2	3	5	95	Yes
Sideroflexin-1	SFXN1_HUMAN	Q9H9B4	SFXN1	35.6	33	7	95	No
Carboxypeptidase Q	CBPQ_HUMAN	Q9Y646	CPQ	51.9	25	9	95	No
Synaptojanin-2-binding protein	SYJ2B_HUMAN	P57105	SYNJ2BP	15.9	63	6	94	Yes
Na(+)/H(+) exchange regulatory cofactor NHE-RF2	NHRF2_HUMAN	Q15599	SLC9A3R2	37.4	30	8	94	Yes
Probable glutamate--tRNA ligase, mitochondrial	SYEM_HUMAN	Q5JPH6	EARS2	58.7	18	11	94	No

Endoplasmic reticulum lectin 1	ERLEC_HUMAN	Q96DZ1	ERLEC1	54.8	31	12	94	Yes
Tetratricopeptide repeat protein 1	TTC1_HUMAN	Q99614	TTC1	33.5	30	8	94	Yes
Aminopeptidase B	AMPB_HUMAN	Q9H4A4	RNPEP	72.5	32	14	94	No
B-cell receptor-associated protein 29	BAP29_HUMAN	Q9UHQ4	BCAP29	28.3	21	7	94	Yes
Profilin-2	PROF2_HUMAN	P35080	PFN2	15	35	6	93	Yes
Afamin	AFAM_HUMAN	P43652	AFM	69	19	10	93	Yes
Emerin	EMD_HUMAN	P50402	EMD	29	30	6	93	Yes
F-actin-capping protein subunit alpha-1	CAZA1_HUMAN	P52907	CAPZA1	32.9	37	7	93	Yes
UV excision repair protein RAD23 homolog A	RD23A_HUMAN	P54725	RAD23A	39.6	27	5	93	No
60S ribosomal protein L31	RL31_HUMAN	P62899	RPL31	14.5	22	5	93	Yes
Peptidyl-prolyl cis-trans isomerase FKBP4	FKBP4_HUMAN	Q02790	FKBP4	51.8	44	14	93	No
Signal peptidase complex subunit 2	SPCS2_HUMAN	Q15005	SPCS2	25	34	6	93	No
Prostacyclin synthase	PTGIS_HUMAN	Q16647	PTGIS	57.1	19	7	93	Yes
AP-2 complex subunit mu	AP2M1_HUMAN	Q96CW1	AP2M1	49.6	17	7	93	Yes
Epidermal growth factor receptor substrate 15-like 1	EP15R_HUMAN	Q9UBC2	EPS15L1	94.2	14	10	93	Yes
Podocalyxin	PODXL_HUMAN	O00592	PODXL	58.6	9	5	92	Yes
ADP-ribosylation factor 3	ARF3_HUMAN	P61204	ARF3	20.6	48	4	92	Yes
Microfibrillar-associated protein 5	MFAP5_HUMAN	Q13361	MFAP5	19.6	21	5	92	Yes
Acyl-CoA synthetase short-chain family member 3, mitochondrial	ACSS3_HUMAN	Q9H6R3	ACSS3	74.7	20	12	92	Yes
rRNA methyltransferase 3, mitochondrial	MRM3_HUMAN	Q9HC36	MRM3	47	12	5	92	Yes
COP9 signalosome complex subunit 7a	CSN7A_HUMAN	Q9UBW8	COPS7A	30.3	36	8	92	Yes
High mobility group protein B1	HMGB1_HUMAN	P09429	HMGB1	24.9	42	8	91	Yes
Ras-related C3 botulinum toxin substrate 1	RAC1_HUMAN	P63000	RAC1	21.4	30	3	91	Yes
Sorting nexin-1	SNX1_HUMAN	Q13596	SNX1	59	26	10	91	Yes
Heterochromatin protein 1-binding protein 3	HP1B3_HUMAN	Q5SSJ5	HP1BP3	61.2	22	12	91	Yes
Cytochrome b-c1 complex subunit 9	QCR9_HUMAN	Q9UDW1	UQCR10	7.3	60	4	91	Yes
Argininosuccinate synthase	ASSY_HUMAN	P00966	ASS1	46.5	21	8	90	Yes
60 kDa SS-A/Ro ribonucleoprotein	RO60_HUMAN	P10155	RO60	60.6	25	12	90	Yes
Integrin alpha-6	ITA6_HUMAN	P23229	ITGA6	126.5	18	13	90	Yes
Microtubule-associated protein RP/EB family member 2	MARE2_HUMAN	Q15555	MAPRE2	37	33	7	90	Yes
NAD kinase 2, mitochondrial	NAKD2_HUMAN	Q4G0N4	NADK2	49.4	29	10	90	Yes
Dynein light chain roadblock-type 1	DLRB1_HUMAN	Q9NP97	DYNLRB1	10.9	51	3	90	Yes
BRISC and BRCA1-A complex member 1	BABA1_HUMAN	Q9NWV8	BABAM1	36.5	19	4	90	No
Dehydrogenase/reductase SDR family member 7	DHRS7_HUMAN	Q9Y394	DHRS7	38.3	20	6	90	Yes
40S ribosomal protein S9	RS9_HUMAN	P46781	RPS9	22.6	40	8	89	Yes
Hemoglobin subunit gamma-1	HBG1_HUMAN	P69891	HBG1	16.1	29	2	89	No

Eukaryotic translation initiation factor 4 gamma 1	IF4G1_HUMAN	Q04637	EIF4G1	175.4	14	18	89	Yes
Plexin domain-containing protein 2	PXDC2_HUMAN	Q6UX71	PLXDC2	59.5	9	4	89	Yes
Guanine nucleotide-binding protein subunit alpha-13	GNA13_HUMAN	Q14344	GNA13	44	20	5	88	No
Early endosome antigen 1	EEA1_HUMAN	Q15075	EEA1	162.4	15	16	88	Yes
Polymerase delta-interacting protein 2	PDIP2_HUMAN	Q9Y2S7	POLDIP2	42	27	9	88	Yes
Serum amyloid A-2 protein	SAA2_HUMAN	P0DJI9	SAA2	13.5	44	2	87	No
Serine/threonine-protein phosphatase 2A 55 kDa regulatory subunit B alpha isoform	2ABA_HUMAN	P63151	PPP2R2A	51.7	33	10	87	Yes
28S ribosomal protein S31, mitochondrial	RT31_HUMAN	Q92665	MRPS31	45.3	23	8	87	Yes
Olfactomedin-like protein 3	OLFL3_HUMAN	Q9NRN5	OLFML3	46	28	9	87	No
RNA-binding protein Raly	RALY_HUMAN	Q9UKM9	RALY	32.4	33	10	87	Yes
Carbonic anhydrase 2	CAH2_HUMAN	P00918	CA2	29.2	30	6	86	Yes
Threonine--tRNA ligase 1, cytoplasmic	SYTC_HUMAN	P26639	TARS1	83.4	15	10	86	No
Dematin	DEMA_HUMAN	Q08495	DMTN	45.5	12	5	86	No
Ferredoxin-2, mitochondrial	FDX2_HUMAN	Q6P4F2	FDX2	19.5	23	3	86	Yes
Prostaglandin reductase 1	PTGR1_HUMAN	Q14914	PTGR1	35.8	26	6	85	Yes
Plasminogen activator inhibitor 1 RNA-binding protein	PAIRB_HUMAN	Q8NC51	SERBP1	44.9	12	4	85	Yes
Lipoma-preferred partner	LPP_HUMAN	Q93052	LPP	65.7	14	7	85	Yes
Heterogeneous nuclear ribonucleoprotein U-like protein 1	HNRL1_HUMAN	Q9BUJ2	HNRNPUL1	95.7	22	13	85	Yes
GDP-fucose protein O-fucosyltransferase 1	OFUT1_HUMAN	Q9H488	POFUT1	43.9	37	11	85	Yes
E3 ubiquitin-protein ligase CHIP	CHIP_HUMAN	Q9UNE7	STUB1	34.8	42	11	85	No
Protein SCO1 homolog, mitochondrial	SCO1_HUMAN	O75880	SCO1	33.8	20	4	84	Yes
Reticulon-3	RTN3_HUMAN	O95197	RTN3	112.5	2	2	84	Yes
Thioredoxin domain-containing protein 12	TXD12_HUMAN	O95881	TXNDC12	19.2	22	3	84	Yes
60S ribosomal protein L7	RL7_HUMAN	P18124	RPL7	29.2	17	4	84	Yes
Ras-related protein Rab-3A	RAB3A_HUMAN	P20336	RAB3A	25	24	3	84	No
Signal recognition particle 9 kDa protein	SRP09_HUMAN	P49458	SRP9	10.1	40	4	84	Yes
Guanine nucleotide-binding protein G(I)/G(S)/G(O) subunit gamma-11	GBG11_HUMAN	P61952	GNG11	8.5	59	5	84	Yes
S-methyl-5'-thioadenosine phosphorylase	MTAP_HUMAN	Q13126	MTAP	31.2	54	10	84	Yes
Prostaglandin E synthase 3	TEBP_HUMAN	Q15185	PTGES3	18.7	31	5	84	Yes
Ribosome-recycling factor, mitochondrial	RRFM_HUMAN	Q96E11	MRRF	29.3	32	7	84	Yes
28S ribosomal protein S26, mitochondrial	RT26_HUMAN	Q9BYN8	MRPS26	24.2	32	8	84	Yes
Protein FAM114A2	F1142_HUMAN	Q9NRY5	FAM114A2	55.4	16	7	84	Yes
DnaJ homolog subfamily B member 6	DNJB6_HUMAN	O75190	DNAJB6	36.1	20	4	83	Yes
SUN domain-containing protein 1	SUN1_HUMAN	O94901	SUN1	90	15	8	83	No

Beta-2-glycoprotein 1	APOH_HUMAN	P02749	APOH	38.3	29	8	83	Yes
CD99 antigen	CD99_HUMAN	P14209	CD99	18.8	15	3	83	Yes
40S ribosomal protein S20	RS20_HUMAN	P60866	RPS20	13.4	31	5	83	Yes
28 kDa heat- and acid-stable phosphoprotein	HAP28_HUMAN	Q13442	PDAP1	20.6	39	8	83	Yes
Probable ATP-dependent RNA helicase DDX17	DDX17_HUMAN	Q92841	DDX17	80.2	30	10	83	No
Tubulin-folding cofactor B	TBCB_HUMAN	Q99426	TBCB	27.3	23	6	83	Yes
Toll-interacting protein	TOLIP_HUMAN	Q9H0E2	TOLLIP	30.3	32	7	83	No
Probable cysteine--tRNA ligase, mitochondrial	SYCM_HUMAN	Q9HA77	CARS2	62.2	16	8	83	Yes
OCIA domain-containing protein 1	OCAD1_HUMAN	Q9NX40	OCIAD1	27.6	32	6	83	Yes
28S ribosomal protein S7, mitochondrial	RT07_HUMAN	Q9Y2R9	MRPS7	28.1	41	11	83	No
Multifunctional procollagen lysine hydroxylase and glycosyltransferase LH3	PLOD3_HUMAN	O60568	PLOD3	84.7	11	7	82	Yes
Angiotensinogen	ANGT_HUMAN	P01019	AGT	53.1	11	5	82	Yes
Cathepsin L1	CATL1_HUMAN	P07711	CTSL	37.5	11	4	82	Yes
Thymidine phosphorylase	TYPH_HUMAN	P19971	TYMP	49.9	22	9	82	Yes
ATP synthase subunit f, mitochondrial	ATPK_HUMAN	P56134	ATP5MF	10.9	26	2	82	Yes
Secernin-1	SCRN1_HUMAN	Q12765	SCRN1	46.4	16	7	82	Yes
Septin-10	SEP10_HUMAN	Q9P0V9	SEPTIN10	52.6	22	6	82	No
Immunoglobulin heavy variable 1-2	HV102_HUMAN	P23083	IGHV1-2	13.1	23	3	81	Yes
Ras-related protein R-Ras2	RRAS2_HUMAN	P62070	RRAS2	23.4	41	5	81	No
Transmembrane emp24 domain-containing protein 2	TMED2_HUMAN	Q15363	TMED2	22.7	14	3	81	Yes
Endothelial cell-selective adhesion molecule	ESAM_HUMAN	Q96AP7	ESAM	41.2	25	6	81	Yes
Secernin-2	SCRN2_HUMAN	Q96FV2	SCRN2	46.6	20	5	81	No
Pigment epithelium-derived factor	PEDF_HUMAN	P36955	SERPINF1	46.3	23	9	80	No
Transport and Golgi organization protein 1 homolog	TGO1_HUMAN	Q5JRA6	MIA3	213.6	11	16	80	No
Protein O-glucosyltransferase 3	PLGT3_HUMAN	Q7Z4H8	POGLUT3	58.5	15	6	80	Yes
NHL repeat-containing protein 2	NHLC2_HUMAN	Q8NBF2	NHLRC2	79.4	17	9	80	Yes
Protein PRRC1	PRRC1_HUMAN	Q96M27	PRRC1	46.7	14	5	80	Yes
Importin subunit alpha-4	IMA4_HUMAN	O00505	KPNA3	57.8	13	3	79	No
Paralemmin-2	PALM2_HUMAN	Q8IXS6	PALM2	42.2	16	5	79	Yes
Thioredoxin-related transmembrane protein 4	TMX4_HUMAN	Q9H1E5	TMX4	38.9	21	9	79	Yes
Prefoldin subunit 4	PFD4_HUMAN	Q9NQP4	PFDN4	15.3	28	3	79	Yes
Integrin beta-5	ITB5_HUMAN	P18084	ITGB5	88	23	13	78	No
Protein ERGIC-53	LMAN1_HUMAN	P49257	LMAN1	57.5	22	7	78	No
Rho GDP-dissociation inhibitor 2	GDIR2_HUMAN	P52566	ARHGDIB	23	45	8	78	Yes
Very low-density lipoprotein receptor	VLDLR_HUMAN	P98155	VLDLR	96	17	11	78	No

Eukaryotic translation initiation factor 4H	IF4H_HUMAN	Q15056	EIF4H	27.4	28	5	78	Yes
Complex III assembly factor LYRM7	LYRM7_HUMAN	Q5U5X0	LYRM7	11.9	57	6	78	Yes
Transportin-1	TNPO1_HUMAN	Q92973	TNPO1	102.3	6	5	78	Yes
Sortilin	SORT_HUMAN	Q99523	SORT1	92	5	5	78	Yes
Transmembrane protein 120A	T120A_HUMAN	Q9BXJ8	TMEM120A	40.6	8	3	78	Yes
Telethonin	TELT_HUMAN	O15273	TCAP	19	47	7	77	Yes
60S ribosomal protein L4	RL4_HUMAN	P36578	RPL4	47.7	22	8	77	No
28S ribosomal protein S9, mitochondrial	RT09_HUMAN	P82933	MRPS9	45.8	25	8	77	No
Reticulocalbin-2	RCN2_HUMAN	Q14257	RCN2	36.9	21	6	77	Yes
NLR family member X1	NLRX1_HUMAN	Q86UT6	NLRX1	107.5	17	13	77	No
Midasin	MDN1_HUMAN	Q9NU22	MDN1	632.4	1	2	77	Yes
Immunoglobulin heavy variable 1-69D	HV69D_HUMAN	A0A0B4J2H0	IGHV1-69D	12.7	15	2	76	Yes
Protein CutA	CUTA_HUMAN	O60888	CUTA	19.1	41	4	76	Yes
Prefoldin subunit 1	PFD1_HUMAN	O60925	PFDN1	14.2	35	5	76	Yes
Cytosolic 10-formyltetrahydrofolate dehydrogenase	AL1L1_HUMAN	O75891	ALDH1L1	98.8	22	12	76	No
ATP-binding cassette sub-family A member 8	ABCA8_HUMAN	O94911	ABCA8	179.1	9	10	76	Yes
Leucine-rich alpha-2-glycoprotein	A2GL_HUMAN	P02750	LRG1	38.2	19	7	76	Yes
Proteasome subunit beta type-10	PSB10_HUMAN	P40306	PSMB10	28.9	19	5	76	Yes
Arginine--tRNA ligase, cytoplasmic	SYRC_HUMAN	P54136	RARS1	75.3	21	10	76	No
40S ribosomal protein S7	RS7_HUMAN	P62081	RPS7	22.1	20	4	76	Yes
Protein bicaudal D homolog 2	BICD2_HUMAN	Q8TD16	BICD2	93.5	13	9	76	No
Nicastrin	NICA_HUMAN	Q92542	NCSTN	78.4	10	8	76	Yes
Acidic leucine-rich nuclear phosphoprotein 32 family member B	AN32B_HUMAN	Q92688	ANP32B	28.8	21	2	76	No
Peroxiredoxin-like 2A	PXL2A_HUMAN	Q9BRX8	PRXL2A	25.7	48	12	76	Yes
DnaJ homolog subfamily B member 2	DNJB2_HUMAN	P25686	DNAJB2	35.6	23	6	75	No
3-ketodihydrosphingosine reductase	KDSR_HUMAN	Q06136	KDSR	36.2	15	4	75	Yes
A-kinase anchor protein SPHKAP	SPKAP_HUMAN	Q2M3C7	SPHKAP	186.3	12	15	75	No
Twinfilin-2	TWF2_HUMAN	Q6IBS0	TWF2	39.5	36	10	75	Yes
Protein FAM3C	FAM3C_HUMAN	Q92520	FAM3C	24.7	39	7	75	Yes
Stabilin-1	STAB1_HUMAN	Q9NY15	STAB1	275.3	17	27	75	No
Long-chain-fatty-acid--CoA ligase 3	ACSL3_HUMAN	O95573	ACSL3	80.4	15	6	74	Yes
U1 small nuclear ribonucleoprotein 70 kDa	RU17_HUMAN	P08621	SNRNP70	51.5	11	6	74	No
U1 small nuclear ribonucleoprotein A	SNRPA_HUMAN	P09012	SNRPA	31.3	14	5	74	Yes
Ribosyldihydronicotinamide dehydrogenase [quinone]	NQO2_HUMAN	P16083	NQO2	25.9	42	6	74	No
cGMP-inhibited 3',5'-cyclic phosphodiesterase A	PDE3A_HUMAN	Q14432	PDE3A	124.9	9	7	74	Yes
Structural maintenance of chromosomes protein 1A	SMC1A_HUMAN	Q14683	SMC1A	143.1	13	16	74	No

Coiled-coil domain-containing protein 127	CC127_HUMAN	Q96BQ5	CCDC127	30.8	27	8	74	No
DnaJ homolog subfamily A member 3, mitochondrial	DNJA3_HUMAN	Q96EY1	DNAJA3	52.5	29	10	74	No
Protein canopy homolog 3	CNPY3_HUMAN	Q9BT09	CNPY3	30.7	30	9	74	Yes
Transmembrane emp24 domain-containing protein 9	TMED9_HUMAN	Q9BVK6	TMED9	27.3	33	6	74	Yes
Endoplasmic reticulum junction formation protein lunapark	LNP_HUMAN	Q9C0E8	LNPK	47.7	22	8	74	No
Pyridoxal kinase	PDXK_HUMAN	O00764	PDXK	35.1	21	6	73	Yes
Hepatocyte growth factor-regulated tyrosine kinase substrate	HGS_HUMAN	O14964	HGS	86.1	10	8	73	Yes
NADH dehydrogenase [ubiquinone] 1 beta subcomplex subunit 6	NDUB6_HUMAN	O95139	NDUFB6	15.5	34	3	73	Yes
Mannose-6-phosphate isomerase	MPI_HUMAN	P34949	MPI	46.6	18	5	73	Yes
Phosphatidylinositol transfer protein beta isoform	PIPNB_HUMAN	P48739	PITPNB	31.5	27	3	73	No
Hepatoma-derived growth factor	HDGF_HUMAN	P51858	HDGF	26.8	27	5	73	No
Coatomer subunit beta	COPB_HUMAN	P53618	COPB1	107.1	15	9	73	No
40S ribosomal protein S18	RS18_HUMAN	P62269	RPS18	17.7	39	9	73	No
Follistatin-related protein 1	FSTL1_HUMAN	Q12841	FSTL1	35	24	8	73	No
Mitochondrial amidoxime reducing component 2	MARC2_HUMAN	Q969Z3	MTARC2	38	17	7	73	No
Prefoldin subunit 5	PFD5_HUMAN	Q99471	PFDN5	17.3	75	10	73	Yes
DnaJ homolog subfamily B member 11	DJB11_HUMAN	Q9UBS4	DNAJB11	40.5	30	8	73	Yes
Bis(5'-adenosyl)-triphosphatase ENPP4	ENPP4_HUMAN	Q9Y6X5	ENPP4	51.6	9	4	73	Yes
Vitamin K-dependent protein S	PROS_HUMAN	P07225	PROS1	75.1	21	12	72	Yes
Lysosomal acid phosphatase	PPAL_HUMAN	P11117	ACP2	48.3	15	5	72	Yes
Folate receptor beta	FOLR2_HUMAN	P14207	FOLR2	29.3	35	8	72	No
Caprin-1	CAPR1_HUMAN	Q14444	CAPRIN1	78.3	17	8	72	Yes
PGC-1 and ERR-induced regulator in muscle protein 1	PERM1_HUMAN	Q5SV97	PERM1	81.3	24	10	72	No
Endoplasmic reticulum-Golgi intermediate compartment protein 1	ERGI1_HUMAN	Q969X5	ERGIC1	32.6	26	6	72	Yes
Tripartite motif-containing protein 54	TRI54_HUMAN	Q9BYV2	TRIM54	40.3	43	9	72	No
Ubiquilin-4	UBQL4_HUMAN	Q9NRR5	UBQLN4	63.8	22	4	72	No
Mitochondrial import receptor subunit TOM22 homolog	TOM22_HUMAN	Q9NS69	TOMM22	15.5	51	5	72	No
Ragulator complex protein LAMTOR3	LTOR3_HUMAN	Q9UHA4	LAMTOR3	13.6	49	4	72	Yes
5'-AMP-activated protein kinase subunit beta-2	AAKB2_HUMAN	O43741	PRKAB2	30.3	39	7	72	Yes
Collagen alpha-1	CO4A1_HUMAN	P02462	COL4A1	160.5	2	3	71	No
Lipoprotein lipase	LIPL_HUMAN	P06858	LPL	53.1	21	8	71	Yes
Erythrocyte band 7 integral membrane protein	STOM_HUMAN	P27105	STOM	31.7	25	5	71	Yes
Peptidyl-prolyl cis-trans isomerase FKBP3	FKBP3_HUMAN	Q00688	FKBP3	25.2	53	11	71	No
Protein FAM98B	FA98B_HUMAN	Q52LJ0	FAM98B	37.2	25	6	71	No
Regulator of nonsense transcripts 1	RENT1_HUMAN	Q92900	UPF1	124.3	16	13	71	No
Endophilin-B2	SHLB2_HUMAN	Q9NR46	SH3GLB2	43.9	31	11	71	No

Coronin-1C	COR1C_HUMAN	Q9ULV4	CORO1C	53.2	27	10	71	Yes
Eukaryotic translation initiation factor 3 subunit D	EIF3D_HUMAN	O15371	EIF3D	63.9	25	9	70	No
Ankyrin-1	ANK1_HUMAN	P16157	ANK1	206.1	8	11	70	No
Multifunctional protein ADE2 [Includes: Phosphoribosylaminoimidazole-succinocarboxamide synthase	PUR6_HUMAN	P22234	PAICS	47	28	8	70	No
Glycine--tRNA ligase	GARS_HUMAN	P41250	GARS1	83.1	17	12	70	Yes
Lamina-associated polypeptide 2, isoforms beta/gamma	LAP2B_HUMAN	P42167	TMPO	50.6	29	9	70	No
Aminoacylase-1	ACY1_HUMAN	Q03154	ACY1	45.9	22	7	70	No
Cell cycle and apoptosis regulator protein 2	CCAR2_HUMAN	Q8N163	CCAR2	102.8	17	12	70	No
Vacuolar protein sorting-associated protein 35	VPS35_HUMAN	Q96QK1	VPS35	91.6	11	7	70	No
ATP synthase subunit s, mitochondrial	ATP5S_HUMAN	Q99766	DMAC2L	24.9	31	5	70	Yes
Glycerol-3-phosphate phosphatase	PGP_HUMAN	A6NDG6	PGP	34	21	6	69	Yes
Armadillo repeat protein deleted in velo-cardio-facial syndrome	ARVC_HUMAN	O00192	ARVCF	104.6	16	9	69	No
26S proteasome non-ATPase regulatory subunit 9	PSMD9_HUMAN	O00233	PSMD9	24.7	36	8	69	No
Pyridoxal phosphate homeostasis protein	PLPHP_HUMAN	O94903	PLPBP	30.3	18	5	69	Yes
Proteasome subunit beta type-9	PSB9_HUMAN	P28065	PSMB9	23.3	36	6	69	Yes
Alanine--tRNA ligase, cytoplasmic	SYAC_HUMAN	P49588	AARS1	106.7	11	10	69	No
Bleomycin hydrolase	BLMH_HUMAN	Q13867	BLMH	52.5	30	12	69	Yes
ER membrane protein complex subunit 2	EMC2_HUMAN	Q15006	EMC2	34.8	23	6	69	No
Inter-alpha-trypsin inhibitor heavy chain H5	ITIH5_HUMAN	Q86UX2	ITIH5	104.5	15	11	69	No
39S ribosomal protein L1, mitochondrial	RM01_HUMAN	Q9BYD6	MRPL1	36.9	18	6	69	Yes
Angiotensin-converting enzyme 2	ACE2_HUMAN	Q9BYF1	ACE2	92.4	18	12	69	Yes
Synaptic vesicle membrane protein VAT-1 homolog-like	VAT1L_HUMAN	Q9HCJ6	VAT1L	45.9	13	4	69	Yes
Complement component C1q receptor	C1QR1_HUMAN	Q9NPY3	CD93	68.5	13	5	69	Yes
Coatomer subunit gamma-1	COPG1_HUMAN	Q9Y678	COPG1	97.7	19	12	69	No
Acylphosphatase-2	ACYP2_HUMAN	P14621	ACYP2	11.1	55	6	68	No
5'-AMP-activated protein kinase catalytic subunit alpha-2	AAPK2_HUMAN	P54646	PRKAA2	62.3	27	6	68	No
CD81 antigen	CD81_HUMAN	P60033	CD81	25.8	18	2	68	Yes
Zyxin	ZYX_HUMAN	Q15942	ZYX	61.2	16	6	68	Yes
tRNA methyltransferase 10 homolog C	TM10C_HUMAN	Q7L0Y3	TRMT10C	47.3	30	9	68	No
Nucleoporin Nup37	NUP37_HUMAN	Q8NFH4	NUP37	36.7	20	6	68	Yes
WD repeat-containing protein 61	WDR61_HUMAN	Q9GZS3	WDR61	33.6	50	9	68	Yes
Methionine adenosyltransferase 2 subunit beta	MAT2B_HUMAN	Q9NZL9	MAT2B	37.5	37	11	68	Yes
Ubiquitin carboxyl-terminal hydrolase isozyme L5	UCHL5_HUMAN	Q9Y5K5	UCHL5	37.6	34	8	68	No
Peroxisomal membrane protein PEX14	PEX14_HUMAN	O75381	PEX14	41.2	18	6	67	Yes
Keratin, type I cuticular Ha6	KRT36_HUMAN	O76013	KRT36	52.2	13	3	67	No

Plasma protease C1 inhibitor	IC1_HUMAN	P05155	SERPING1	55.1	23	11	67	Yes
Thymidylate kinase	KTHY_HUMAN	P23919	DTYMK	23.8	35	7	67	No
60S ribosomal protein L11	RL11_HUMAN	P62913	RPL11	20.2	27	5	67	Yes
Y-box-binding protein 1	YBOX1_HUMAN	P67809	YBX1	35.9	43	6	67	No
Tyrosine-protein phosphatase non-receptor type 11	PTN11_HUMAN	Q06124	PTPN11	68.4	25	13	67	Yes
Transmembrane glycoprotein NMB	GPNMB_HUMAN	Q14956	GPNMB	63.9	6	3	67	Yes
ER membrane protein complex subunit 10	EMC10_HUMAN	Q5UCC4	EMC10	27.3	13	3	67	No
Chondroitin sulfate proteoglycan 4	CSPG4_HUMAN	Q6UVK1	CSPG4	250.4	11	16	67	No
LYR motif-containing protein 4	LYRM4_HUMAN	Q9HD34	LYRM4	10.8	44	4	67	Yes
Mitochondrial pyruvate carrier 1	MPC1_HUMAN	Q9Y5U8	MPC1	12.3	16	2	67	Yes
Wolframin	WFS1_HUMAN	O76024	WFS1	100.2	8	5	66	Yes
Immunoglobulin kappa variable 3-20	KV320_HUMAN	P01619	IGKV3-20	12.5	28	3	66	No
ATP synthase protein 8	ATP8_HUMAN	P03928	MT-ATP8	8	56	4	66	Yes
Alpha-2-antiplasmin	A2AP_HUMAN	P08697	SERPINF2	54.5	15	5	66	No
CDGSH iron-sulfur domain-containing protein 3, mitochondrial	CISD3_HUMAN	P0C7P0	CISD3	14.2	36	4	66	Yes
Myristoylated alanine-rich C-kinase substrate	MARCS_HUMAN	P29966	MARCKS	31.5	24	5	66	No
60S ribosomal protein L6	RL6_HUMAN	Q02878	RPL6	32.7	24	6	66	Yes
39S ribosomal protein L38, mitochondrial	RM38_HUMAN	Q96DV4	MRPL38	44.6	37	11	66	Yes
Sideroflexin-3	SFXN3_HUMAN	Q9BWM7	SFXN3	35.5	45	9	66	No
EMILIN-2	EMIL2_HUMAN	Q9BXX0	EMILIN2	115.6	9	7	66	Yes
Phenylalanine--tRNA ligase beta subunit	SYFB_HUMAN	Q9NSD9	FARSB	66.1	24	14	66	No
Golgi integral membrane protein 4	GOLI4_HUMAN	O00461	GOLIM4	81.8	15	8	65	Yes
Phytanoyl-CoA dioxygenase, peroxisomal	PAHX_HUMAN	O14832	PHYH	38.5	17	6	65	Yes
Asparagine--tRNA ligase, cytoplasmic	SYNC_HUMAN	O43776	NARS1	62.9	20	8	65	Yes
Immunoglobulin heavy variable 3-30	HV330_HUMAN	P01768	IGHV3-30	12.9	28	3	65	No
cAMP-dependent protein kinase inhibitor alpha	IPKA_HUMAN	P61925	PKIA	8	74	4	65	No
Transcription intermediary factor 1-beta	TIF1B_HUMAN	Q13263	TRIM28	88.5	26	14	65	No
Sterol-4-alpha-carboxylate 3-dehydrogenase, decarboxylating	NSDHL_HUMAN	Q15738	NSDHL	41.9	13	5	65	No
ER membrane protein complex subunit 1	EMC1_HUMAN	Q8N766	EMC1	111.7	18	12	65	No
Transcriptional activator protein Pur-beta	PURB_HUMAN	Q96QR8	PURB	33.2	26	6	65	No
MICOS complex subunit MIC25	MIC25_HUMAN	Q9BRQ6	CHCHD6	26.4	40	6	65	No
Neurolysin, mitochondrial	NEUL_HUMAN	Q9BYT8	NLN	80.6	24	12	65	No
Retinol dehydrogenase 14	RDH14_HUMAN	Q9HBH5	RDH14	36.8	33	12	65	No
39S ribosomal protein L39, mitochondrial	RM39_HUMAN	Q9NYK5	MRPL39	38.7	25	10	65	No
Drebrin-like protein	DBNL_HUMAN	Q9UJU6	DBNL	48.2	16	5	65	No
Microsomal glutathione S-transferase 3	MGST3_HUMAN	O14880	MGST3	16.5	21	2	64	Yes

Natriuretic peptides A	ANF_HUMAN	P01160	NPPA	16.7	19	3	64	Yes
Large proline-rich protein BAG6	BAG6_HUMAN	P46379	BAG6	119.3	14	9	64	No
Beta-1-syntrophin	SNTB1_HUMAN	Q13884	SNTB1	58	9	2	64	No
Translin	TSN_HUMAN	Q15631	TSN	26.2	25	6	64	Yes
CD109 antigen	CD109_HUMAN	Q6YHK3	CD109	161.6	7	8	64	No
Tripartite motif-containing protein 55	TRI55_HUMAN	Q9BYV6	TRIM55	60.4	23	10	64	No
Phospholipase A-2-activating protein	PLAP_HUMAN	Q9Y263	PLAA	87.1	21	15	64	No
28S ribosomal protein S18b, mitochondrial	RT18B_HUMAN	Q9Y676	MRPS18B	29.4	29	6	64	No
Wiskott-Aldrich syndrome protein family member 2	WASF2_HUMAN	Q9Y6W5	WASF2	54.3	12	5	64	Yes
Ribose-5-phosphate isomerase	RPIA_HUMAN	P49247	RPIA	33.2	27	7	64	Yes
Actin-related protein 2/3 complex subunit 2	ARPC2_HUMAN	O15144	ARPC2	34.3	25	7	63	No
Tapasin	TPSN_HUMAN	O15533	TAPBP	47.6	15	5	63	Yes
TP53-regulated inhibitor of apoptosis 1	TRIA1_HUMAN	O43715	TRIAP1	8.8	62	5	63	No
Arylsulfatase B	ARSB_HUMAN	P15848	ARSB	59.6	16	6	63	No
Serum paraoxonase/arylesterase 1	PON1_HUMAN	P27169	PON1	39.7	21	7	63	No
Transcriptional coactivator YAP1	YAP1_HUMAN	P46937	YAP1	54.4	29	8	63	Yes
Small nuclear ribonucleoprotein Sm D2	SMD2_HUMAN	P62316	SNRPD2	13.5	54	6	63	No
Cellular nucleic acid-binding protein	CNBP_HUMAN	P62633	CNBP	19.5	23	4	63	Yes
60S ribosomal protein L23a	RL23A_HUMAN	P62750	RPL23A	17.7	29	6	63	Yes
Spliceosome RNA helicase DDX39B	DX39B_HUMAN	Q13838	DDX39B	49	15	5	63	Yes
Protein disulfide isomerase CRELD2	CREL2_HUMAN	Q6UXH1	CRELD2	38.2	15	5	63	No
Perilipin-2	PLIN2_HUMAN	Q99541	PLIN2	48	52	17	63	No
[3-methyl-2-oxobutanoate dehydrogenase [lipoamide]] kinase, mitochondrial	BCKD_HUMAN	O14874	BCKDK	46.3	20	7	62	No
Lysosome-associated membrane glycoprotein 1	LAMP1_HUMAN	P11279	LAMP1	44.9	10	4	62	Yes
Parathymosin	PTMS_HUMAN	P20962	PTMS	11.5	24	3	62	Yes
RNA-binding protein FUS	FUS_HUMAN	P35637	FUS	53.4	12	4	62	No
Protein phosphatase inhibitor 2	IPP2_HUMAN	P41236	PPP1R2	23	43	7	62	Yes
Ribose-phosphate pyrophosphokinase 1	PRPS1_HUMAN	P60891	PRPS1	34.8	34	11	62	Yes
Eukaryotic translation initiation factor 3 subunit L	EIF3L_HUMAN	Q9Y262	EIF3L	66.7	17	10	62	Yes
MOB-like protein phocein	PHOCN_HUMAN	Q9Y3A3	MOB4	26	25	5	62	No
RNA-binding protein 8A	RBM8A_HUMAN	Q9Y5S9	RBM8A	19.9	21	3	62	Yes
Elongation factor Ts, mitochondrial	EFTS_HUMAN	P43897	TSFM	35.4	33	9	61	No
Transmembrane emp24 domain-containing protein 1	TMED1_HUMAN	Q13445	TMED1	25.2	22	4	61	Yes
Protein quaking	QKI_HUMAN	Q96PU8	QKI	37.6	19	5	61	Yes
Golgi-associated plant pathogenesis-related protein 1	GAPR1_HUMAN	Q9H4G4	GLIPR2	17.2	42	4	61	No

Hypoxanthine-guanine phosphoribosyltransferase	HPRT_HUMAN	P00492	HPRT1	24.6	25	5	60	No
Ubiquitin carboxyl-terminal hydrolase isozyme L3	UCHL3_HUMAN	P15374	UCHL3	26.2	51	8	60	No
Ganglioside GM2 activator	SAP3_HUMAN	P17900	GM2A	20.8	30	6	60	No
Serine--tRNA ligase, cytoplasmic	SYSC_HUMAN	P49591	SARS1	58.7	19	8	60	No
40S ribosomal protein S28	RS28_HUMAN	P62857	RPS28	7.8	52	4	60	No
Amyloid-like protein 2	APLP2_HUMAN	Q06481	APLP2	86.9	13	8	60	No
Beta-2-syntrophin	SNTB2_HUMAN	Q13425	SNTB2	57.9	19	7	60	No
ERO1-like protein alpha	ERO1A_HUMAN	Q96HE7	ERO1A	54.4	22	8	60	No
Structural maintenance of chromosomes protein 3	SMC3_HUMAN	Q9UQE7	SMC3	141.5	11	11	60	No
V-type proton ATPase subunit G 1	VATG1_HUMAN	O75348	ATP6V1G1	13.7	29	3	59	No
Laforin	EPM2A_HUMAN	O95278	EPM2A	37.1	25	5	59	No
Eukaryotic translation initiation factor 2 subunit 1	IF2A_HUMAN	P05198	EIF2S1	36.1	28	7	59	No
Gap junction alpha-1 protein	CXA1_HUMAN	P17302	GJA1	43	27	7	59	No
Methionine--tRNA ligase, cytoplasmic	SYMC_HUMAN	P56192	MARS1	101.1	11	8	59	No
40S ribosomal protein S21	RS21_HUMAN	P63220	RPS21	9.1	46	4	59	Yes
Splicing factor 3A subunit 1	SF3A1_HUMAN	Q15459	SF3A1	88.8	20	11	59	No
Filaggrin-2	FILA2_HUMAN	Q5D862	FLG2	247.9	9	9	59	No
FAST kinase domain-containing protein 4	FAKD4_HUMAN	Q969Z0	TBRG4	70.7	10	7	59	No
LIM domain and actin-binding protein 1	LIMA1_HUMAN	Q9UHB6	LIMA1	85.2	11	6	59	Yes
Ras GTPase-activating protein-binding protein 2	G3BP2_HUMAN	Q9UN86	G3BP2	54.1	13	5	59	No
Peptidyl-prolyl cis-trans isomerase E	PPIE_HUMAN	Q9UNP9	PPIE	33.4	13	3	59	No
UMP-CMP kinase	KCY_HUMAN	P30085	CMPK1	22.2	31	5	58	Yes
5'-AMP-activated protein kinase catalytic subunit alpha-1	AAPK1_HUMAN	Q13131	PRKAA1	64	15	2	58	No
Malectin	MLEC_HUMAN	Q14165	MLEC	32.2	9	2	58	No
Actin-related protein 2/3 complex subunit 1A	ARC1A_HUMAN	Q92747	ARPC1A	41.5	21	6	58	Yes
RNA-binding protein with multiple splicing	RBPMS_HUMAN	Q93062	RBPMS	21.8	25	3	58	Yes
ATP synthase membrane subunit DAPIT, mitochondrial	ATPMD_HUMAN	Q96IX5	ATP5MD	6.5	45	3	58	Yes
Serine/threonine-protein phosphatase CPPED1	CPPED_HUMAN	Q9BRF8	CPPED1	35.5	22	6	58	No
AP-2 complex subunit alpha-2	AP2A2_HUMAN	O94973	AP2A2	103.9	11	6	57	No
40S ribosomal protein S17	RS17_HUMAN	P08708	RPS17	15.5	39	5	57	Yes
Syndecan-2	SDC2_HUMAN	P34741	SDC2	22.1	12	2	57	Yes
Protein phosphatase 1A	PPM1A_HUMAN	P35813	PPM1A	42.4	29	6	57	No
RNA-binding motif protein, X chromosome	RBMX_HUMAN	P38159	RBMX	42.3	14	5	57	No
Translocon-associated protein subunit delta	SSRD_HUMAN	P51571	SSR4	19	36	5	57	Yes
Microtubule-associated protein 1A	MAP1A_HUMAN	P78559	MAP1A	305.3	8	15	57	No
Sodium channel protein type 5 subunit alpha	SCN5A_HUMAN	Q14524	SCN5A	226.8	4	6	57	No

Glycerol-3-phosphate acyltransferase 3	GPAT3_HUMAN	Q53EU6	GPAT3	48.7	20	6	57	No
La-related protein 1	LARP1_HUMAN	Q6PKG0	LARP1	123.4	6	5	57	No
Translation initiation factor IF-3, mitochondrial	IF3M_HUMAN	Q9H2K0	MTIF3	31.7	32	8	57	Yes
Mitogen-activated protein kinase kinase kinase 20	M3K20_HUMAN	Q9NYL2	MAP3K20	91.1	9	7	57	Yes
Basic leucine zipper and W2 domain-containing protein 2	BZW2_HUMAN	Q9Y6E2	BZW2	48.1	12	5	57	Yes
Monocyte differentiation antigen CD14	CD14_HUMAN	P08571	CD14	40.1	31	7	56	No
Arylsulfatase A	ARSA_HUMAN	P15289	ARSA	53.6	20	7	56	No
Y-box-binding protein 3	YBOX3_HUMAN	P16989	YBX3	40.1	52	8	56	No
28S ribosomal protein S35, mitochondrial	RT35_HUMAN	P82673	MRPS35	36.8	37	10	56	No
Isoamyl acetate-hydrolyzing esterase 1 homolog	IAH1_HUMAN	Q2TAA2	IAH1	27.6	33	7	56	Yes
Actin-related protein 2/3 complex subunit 3	ARPC3_HUMAN	O15145	ARPC3	20.5	25	5	55	Yes
Target of Myb protein 1	TOM1_HUMAN	O60784	TOM1	53.8	24	9	55	No
Endonuclease domain-containing 1 protein	ENDD1_HUMAN	O94919	ENDOD1	55	14	6	55	No
Pro-neuropeptide Y	NPY_HUMAN	P01303	NPY	10.8	16	2	55	Yes
Platelet-derived growth factor receptor beta	PGFRB_HUMAN	P09619	PDGFRB	123.9	11	9	55	No
Glycerol-3-phosphate dehydrogenase [NAD	GPDA_HUMAN	P21695	GPD1	37.5	17	4	55	No
Proteasome subunit beta type-8	PSB8_HUMAN	P28062	PSMB8	30.3	47	9	55	No
Actin-related protein 2/3 complex subunit 4	ARPC4_HUMAN	P59998	ARPC4	19.7	32	6	55	Yes
RNA-binding protein EWS	EWS_HUMAN	Q01844	EWSR1	68.4	12	5	55	No
Kinesin light chain 1	KLC1_HUMAN	Q07866	KLC1	65.3	19	7	55	No
39S ribosomal protein L49, mitochondrial	RM49_HUMAN	Q13405	MRPL49	19.2	29	4	55	No
Phosphatidylinositol-binding clathrin assembly protein	PICAL_HUMAN	Q13492	PICALM	70.7	10	7	55	No
Kynurenine--oxoglutarate transaminase 3	KAT3_HUMAN	Q6YP21	KYAT3	51.4	22	7	55	Yes
Nectin-2	NECT2_HUMAN	Q92692	NECTIN2	57.7	3	2	55	No
COP9 signalosome complex subunit 5	CSN5_HUMAN	Q92905	COPS5	37.6	23	9	55	Yes
Mitochondrial import inner membrane translocase subunit Tim21	TIM21_HUMAN	Q9BVV7	TIMM21	28.2	19	4	55	Yes
Poly	PUF60_HUMAN	Q9UHX1	PUF60	59.8	14	6	55	No
ER membrane protein complex subunit 8	EMC8_HUMAN	O43402	EMC8	23.8	34	5	54	No
NADH dehydrogenase [ubiquinone] 1 beta subcomplex subunit 1	NDUB1_HUMAN	O75438	NDUFB1	7	33	2	54	No
Non-histone chromosomal protein HMG-17	HMGN2_HUMAN	P05204	HMGN2	9.4	16	2	54	Yes
Hyaluronan and proteoglycan link protein 1	HPLN1_HUMAN	P10915	HAPLN1	40.1	22	6	54	No
60S ribosomal protein L22	RL22_HUMAN	P35268	RPL22	14.8	30	3	54	No
Eukaryotic translation initiation factor 3 subunit E	EIF3E_HUMAN	P60228	EIF3E	52.2	17	6	54	No
40S ribosomal protein S25	RS25_HUMAN	P62851	RPS25	13.7	24	3	54	No
Leiomodin-3	LMOD3_HUMAN	Q0VAK6	LMOD3	64.9	18	9	54	Yes
Cullin-3	CUL3_HUMAN	Q13618	CUL3	88.9	12	9	54	No

Cullin-4A	CUL4A_HUMAN	Q13619	CUL4A	87.6	8	6	54	No
Peptidyl-prolyl cis-trans isomerase FKBP8	FKBP8_HUMAN	Q14318	FKBP8	44.5	24	8	54	No
Serine/threonine-protein phosphatase 2A activator	PTPA_HUMAN	Q15257	PTPA	40.6	20	5	54	No
Zinc transporter 9	ZNT9_HUMAN	Q6PML9	SLC30A9	63.5	15	8	54	No
Bcl-2-like protein 13	B2L13_HUMAN	Q9BXK5	BCL2L13	52.7	29	8	54	Yes
Nuclear ubiquitous casein and cyclin-dependent kinase substrate 1	NUCKS_HUMAN	Q9H1E3	NUCKS1	27.3	37	7	54	No
StAR-related lipid transfer protein 7, mitochondrial	STAR7_HUMAN	Q9NQZ5	STARD7	43.1	21	6	54	Yes
Serine/threonine-protein kinase OSR1	OXSR1_HUMAN	O95747	OXSR1	58	8	4	53	No
Farnesyl pyrophosphate synthase	FPPS_HUMAN	P14324	FDPS	48.2	18	7	53	Yes
Macrophage mannose receptor 1	MRC1_HUMAN	P22897	MRC1	165.9	9	11	53	No
Vesicle-fusing ATPase	NSF_HUMAN	P46459	NSF	82.5	12	8	53	No
60S ribosomal protein L14	RL14_HUMAN	P50914	RPL14	23.4	27	5	53	Yes
Lysosome membrane protein 2	SCRB2_HUMAN	Q14108	SCARB2	54.3	7	4	53	No
Splicing factor 3A subunit 2	SF3A2_HUMAN	Q15428	SF3A2	49.2	11	4	53	No
Long-chain fatty acid transport protein 1	S27A1_HUMAN	Q6PCB7	SLC27A1	71.1	16	8	53	No
Protein phosphatase 1K, mitochondrial	PPM1K_HUMAN	Q8N3J5	PPM1K	41	9	4	53	No
Nicotinamide/nicotinic acid mononucleotide adenylyltransferase 3	NMNA3_HUMAN	Q96T66	NMNAT3	28.3	29	8	53	No
Cytosolic 5'-nucleotidase 3A	5NT3A_HUMAN	Q9H0P0	NT5C3A	37.9	25	8	53	No
39S ribosomal protein L17, mitochondrial	RM17_HUMAN	Q9NRX2	MRPL17	20	31	5	53	Yes
Small muscular protein	SMPX_HUMAN	Q9UHP9	SMPX	9.6	72	4	53	Yes
28S ribosomal protein S28, mitochondrial	RT28_HUMAN	Q9Y2Q9	MRPS28	20.8	30	6	53	No
Glutathione S-transferase LANCL1	LANC1_HUMAN	O43813	LANCL1	45.3	10	4	52	No
Programmed cell death protein 6	PDCD6_HUMAN	O75340	PDCD6	21.9	23	4	52	No
Adenylate cyclase type 5	ADCY5_HUMAN	O95622	ADCY5	138.8	5	4	52	No
Hippocalcin-like protein 1	HPCL1_HUMAN	P37235	HPCAL1	22.3	18	4	52	No
Peroxisomal biogenesis factor 19	PEX19_HUMAN	P40855	PEX19	32.8	33	7	52	No
Nucleoside diphosphate-linked moiety X motif 6	NUDT6_HUMAN	P53370	NUDT6	35.7	26	6	52	No
Eukaryotic translation initiation factor 4 gamma 2	IF4G2_HUMAN	P78344	EIF4G2	102.3	8	8	52	No
LRP chaperone MESD	MESD_HUMAN	Q14696	MESD	26.1	24	4	52	Yes
Cytochrome P450 20A1	CP20A_HUMAN	Q6UW02	CYP20A1	52.4	5	2	52	No
Probable aminopeptidase NPEPL1	PEPL1_HUMAN	Q8NDH3	NPEPL1	55.8	22	8	52	No
Plasmalemma vesicle-associated protein	PLVAP_HUMAN	Q9BX97	PLVAP	50.6	19	8	52	No
39S ribosomal protein L44, mitochondrial	RM44_HUMAN	Q9H9J2	MRPL44	37.5	27	7	52	Yes
Testis-expressed protein 264	TX264_HUMAN	Q9Y6I9	TEX264	34.2	13	3	52	No
6-phosphogluconolactonase	6PGL_HUMAN	O95336	PGLS	27.5	46	9	51	No

Integrin beta-2	ITB2_HUMAN	P05107	ITGB2	84.7	17	12	51	No
Poly [ADP-ribose] polymerase 1	PARP1_HUMAN	P09874	PARP1	113	17	15	51	No
N-acetylglucosamine-6-sulfatase	GNS_HUMAN	P15586	GNS	62	16	8	51	No
Mitogen-activated protein kinase 3	MK03_HUMAN	P27361	MAPK3	43.1	21	3	51	No
Afadin	AFAD_HUMAN	P55196	AFDN	206.7	3	5	51	No
40S ribosomal protein S8	RS8_HUMAN	P62241	RPS8	24.2	25	4	51	No
Ras-interacting protein 1	RAIN_HUMAN	Q5U651	RASIP1	103.4	11	6	51	No
39S ribosomal protein L53, mitochondrial	RM53_HUMAN	Q96EL3	MRPL53	12.1	36	4	51	Yes
NADH dehydrogenase [ubiquinone] 1 beta subcomplex subunit 11, mitochondrial	NDUBB_HUMAN	Q9NX14	NDUFB11	17.3	22	2	51	Yes
DCC-interacting protein 13-alpha	DP13A_HUMAN	Q9UKG1	APPL1	79.6	22	12	51	No
Sorting nexin-6	SNX6_HUMAN	Q9UNH7	SNX6	46.6	28	8	51	No
Sorting nexin-5	SNX5_HUMAN	Q9Y5X3	SNX5	46.8	27	9	51	No
Guanine nucleotide-binding protein G(I)/G(S)/G(O) subunit gamma-7	GBG7_HUMAN	O60262	GNG7	7.5	57	4	50	Yes
ATP-binding cassette sub-family B member 7, mitochondrial	ABCB7_HUMAN	O75027	ABCB7	82.6	9	8	50	No
Intercellular adhesion molecule 1	ICAM1_HUMAN	P05362	ICAM1	57.8	14	5	50	No
Carbonic anhydrase 4	CAH4_HUMAN	P22748	CA4	35	27	7	50	No
N-acetylgalactosamine-6-sulfatase	GALNS_HUMAN	P34059	GALNS	58	8	4	50	No
LIM and senescent cell antigen-like-containing domain protein 1	LIMS1_HUMAN	P48059	LIMS1	37.2	21	6	50	Yes
V-type proton ATPase subunit d 1	VA0D1_HUMAN	P61421	ATP6V0D1	40.3	11	4	50	Yes
40S ribosomal protein S13	RS13_HUMAN	P62277	RPS13	17.2	42	7	50	Yes
EARP and GARP complex-interacting protein 1	EIPR1_HUMAN	Q53HC9	EIPR1	43.6	34	9	50	No
Rab-like protein 3	RABL3_HUMAN	Q5HYI8	RABL3	26.4	9	2	50	No
UBX domain-containing protein 4	UBXN4_HUMAN	Q92575	UBXN4	56.7	17	5	50	No
Signal recognition particle subunit SRP68	SRP68_HUMAN	Q9UHB9	SRP68	70.7	7	2	50	No
Endoplasmic reticulum-Golgi intermediate compartment protein 3	ERGI3_HUMAN	Q9Y282	ERGIC3	43.2	10	5	50	No
Signal recognition particle receptor subunit beta	SRPRB_HUMAN	Q9Y5M8	SRPRB	29.7	24	5	50	No
Prolyl 4-hydroxylase subunit alpha-2	P4HA2_HUMAN	O15460	P4HA2	60.9	32	13	49	No
E3 UFM1-protein ligase 1	UFL1_HUMAN	O94874	UFL1	89.5	17	11	49	Yes
Amyloid-beta precursor protein	A4_HUMAN	P05067	APP	86.9	19	11	49	No
Phospholipid hydroperoxide glutathione peroxidase	GPX4_HUMAN	P36969	GPX4	22.2	33	5	49	No
CD151 antigen	CD151_HUMAN	P48509	CD151	28.3	10	3	49	Yes
60S ribosomal protein L34	RL34_HUMAN	P49207	RPL34	13.3	35	5	49	No
NEDD8-conjugating enzyme Ubc12	UBC12_HUMAN	P61081	UBE2M	20.9	33	8	49	No
Peptidyl-tRNA hydrolase ICT1, mitochondrial	ICT1_HUMAN	Q14197	MRPL58	23.6	33	6	49	No

EH domain-binding protein 1-like protein 1	EH1L1_HUMAN	Q8N3D4	EHBP1L1	161.8	9	7	49	No
Ketosamine-3-kinase	KT3K_HUMAN	Q9HA64	FN3KRP	34.4	25	6	49	Yes
Na	NHRF1_HUMAN	O14745	SLC9A3R1	38.8	23	6	48	No
Reticulon-2	RTN2_HUMAN	O75298	RTN2	59.2	5	3	48	Yes
Cold shock domain-containing protein E1	CSDE1_HUMAN	O75534	CSDE1	88.8	19	13	48	No
Complement component C6	CO6_HUMAN	P13671	C6	104.7	13	12	48	No
DnaJ homolog subfamily A member 1	DNJA1_HUMAN	P31689	DNAJA1	44.8	18	6	48	No
Crk-like protein	CRKL_HUMAN	P46109	CRKL	33.8	50	11	48	No
TAR DNA-binding protein 43	TADBP_HUMAN	Q13148	TARDBP	44.7	8	3	48	Yes
WASH complex subunit 2A	WAC2A_HUMAN	Q641Q2	WASHC2A	147.1	7	7	48	No
Procollagen galactosyltransferase 1	GT251_HUMAN	Q8NBJ5	COLGALT1	71.6	11	6	48	No
Peroxidasin homolog	PXDN_HUMAN	Q92626	PXDN	165.2	9	8	48	No
Armadillo repeat-containing protein 1	ARMC1_HUMAN	Q9NVT9	ARMC1	31.3	20	4	48	Yes
HCLS1-associated protein X-1	HAX1_HUMAN	O00165	HAX1	31.6	35	8	47	No
Monocarboxylate transporter 2	MOT2_HUMAN	O60669	SLC16A7	52.2	8	5	47	No
Cartilage intermediate layer protein 1	CILP1_HUMAN	O75339	CILP	132.5	8	6	47	No
Epidermal growth factor receptor	EGFR_HUMAN	P00533	EGFR	134.2	4	5	47	No
Xaa-Pro dipeptidase	PEPD_HUMAN	P12955	PEPD	54.5	19	6	47	No
Probable ATP-dependent RNA helicase DDX5	DDX5_HUMAN	P17844	DDX5	69.1	16	2	47	No
60S ribosomal protein L28	RL28_HUMAN	P46779	RPL28	15.7	30	5	47	No
Tumor protein D52	TPD52_HUMAN	P55327	TPD52	24.3	65	10	47	No
Pleckstrin homology-like domain family B member 1	PHLB1_HUMAN	Q86UU1	PHLDB1	151.1	3	5	47	No
D-2-hydroxyglutarate dehydrogenase, mitochondrial	D2HDH_HUMAN	Q8N465	D2HGDH	56.4	24	10	47	No
Transcription elongation factor A protein-like 3	TCAL3_HUMAN	Q969E4	TCEAL3	22.5	20	2	47	No
Protein mago nashi homolog 2	MGN2_HUMAN	Q96A72	MAGOHB	17.3	30	4	47	No
Caseinolytic peptidase B protein homolog	CLPB_HUMAN	Q9H078	CLPB	78.7	9	5	47	No
ATP-dependent RNA helicase DDX3X	DDX3X_HUMAN	O00571	DDX3X	73.2	16	7	46	No
ATP-dependent Clp protease ATP-binding subunit clpX-like, mitochondrial	CLPX_HUMAN	O76031	CLPX	69.2	18	8	46	No
Major prion protein	PRIO_HUMAN	P04156	PRNP	27.6	11	3	46	No
Mast cell carboxypeptidase A	CBPA3_HUMAN	P15088	CPA3	48.6	11	5	46	No
Nuclear autoantigenic sperm protein	NASP_HUMAN	P49321	NASP	85.2	12	8	46	No
40S ribosomal protein S14	RS14_HUMAN	P62263	RPS14	16.3	33	4	46	No
Procollagen C-endopeptidase enhancer 1	PCOC1_HUMAN	Q15113	PCOLCE	47.9	20	7	46	No
Calponin-3	CNN3_HUMAN	Q15417	CNN3	36.4	28	6	46	No
NEDD8	NEDD8_HUMAN	Q15843	NEDD8	9.1	54	5	46	Yes

ATP-dependent RNA helicase SUPV3L1, mitochondrial	SUV3_HUMAN	Q8IYB8	SUPV3L1	87.9	9	5	46	No
SWI/SNF complex subunit SMARCC2	SMRC2_HUMAN	Q8TAQ2	SMARCC2	132.8	8	8	46	No
Cytochrome c oxidase assembly factor 7	COA7_HUMAN	Q96BR5	COA7	25.7	40	9	46	No
SH3 domain-binding glutamic acid-rich-like protein 3	SH3L3_HUMAN	Q9H299	SH3BGRL3	10.4	39	3	46	Yes
Mth938 domain-containing protein	AAMDC_HUMAN	Q9H7C9	AAMDC	13.3	42	4	46	Yes
Ras-related GTP-binding protein C	RRAGC_HUMAN	Q9HB90	RRAGC	44.2	19	7	46	No
Popeye domain-containing protein 2	POPD2_HUMAN	Q9HBU9	POPDC2	40.4	10	3	46	No
Mitochondrial import inner membrane translocase subunit Tim10 B	T10B_HUMAN	Q9Y5J6	TIMM10B	11.6	50	4	46	No
Immunoglobulin heavy variable 5-51	HV551_HUMAN	A0A0C4DH38	IGHV5-51	12.7	53	4	45	No
Phosphoribosyl pyrophosphate synthase-associated protein 2	KPRB_HUMAN	O60256	PRPSAP2	40.9	25	7	45	Yes
Lysosomal protective protein	PPGB_HUMAN	P10619	CTSA	54.4	12	5	45	No
Aquaporin-1	AQP1_HUMAN	P29972	AQP1	28.5	12	3	45	No
Glycerol-3-phosphate dehydrogenase, mitochondrial	GPDM_HUMAN	P43304	GPD2	80.8	10	6	45	No
Dermcidin	DCD_HUMAN	P81605	DCD	11.3	30	3	45	Yes
MAP7 domain-containing protein 1	MA7D1_HUMAN	Q3KQU3	MAP7D1	92.8	5	4	45	Yes
Succinate dehydrogenase assembly factor 4, mitochondrial	SDHF4_HUMAN	Q5VUM1	SDHAF4	12.2	47	4	45	Yes
Atlastin-2	ATLA2_HUMAN	Q8NHH9	ATL2	66.2	11	4	45	No
Myocilin	MYOC_HUMAN	Q99972	MYOC	56.9	8	4	45	No
Serine/threonine-protein kinase WNK1	WNK1_HUMAN	Q9H4A3	WNK1	250.6	1	3	45	No
TBC1 domain family member 17	TBC17_HUMAN	Q9HA65	TBC1D17	72.7	10	6	45	No
N-acetyl-D-glucosamine kinase	NAGK_HUMAN	Q9UJ70	NAGK	37.4	33	8	45	No
Lipoyl synthase, mitochondrial	LIAS_HUMAN	O43766	LIAS	41.9	28	7	44	No
Long-chain-fatty-acid--CoA ligase 4	ACSL4_HUMAN	O60488	ACSL4	79.1	16	6	44	No
Transferrin receptor protein 1	TFR1_HUMAN	P02786	TFRC	84.8	13	7	44	No
Matrix Gla protein	MGP_HUMAN	P08493	MGP	12.3	32	3	44	No
2',3'-cyclic-nucleotide 3'-phosphodiesterase	CN37_HUMAN	P09543	CNP	47.5	22	7	44	Yes
Coronin-1A	COR1A_HUMAN	P31146	CORO1A	51	16	7	44	No
Glycerol kinase	GLPK_HUMAN	P32189	GK	61.2	17	8	44	No
40S ribosomal protein S23	RS23_HUMAN	P62266	RPS23	15.8	29	4	44	Yes
Rho-related GTP-binding protein RhoB	RHOB_HUMAN	P62745	RHOB	22.1	27	2	44	No
28S ribosomal protein S11, mitochondrial	RT11_HUMAN	P82912	MRPS11	20.6	36	4	44	Yes
Guanidinoacetate N-methyltransferase	GAMT_HUMAN	Q14353	GAMT	26.3	19	3	44	No
ELAV-like protein 1	ELAV1_HUMAN	Q15717	ELAVL1	36.1	19	5	44	No
Drebrin	DREB_HUMAN	Q16643	DBN1	71.4	9	4	44	No
Suprabasin	SBSN_HUMAN	Q6UWP8	SBSN	60.5	15	2	44	No

Filamin-A-interacting protein 1	FLIP1_HUMAN	Q7Z7B0	FILIP1	138	7	5	44	No
Nesprin-2	SYNE2_HUMAN	Q8WXH0	SYNE2	795.9	2	8	44	No
182 kDa tankyrase-1-binding protein	TB182_HUMAN	Q9C0C2	TNKS1BP1	181.7	7	7	44	No
Ragulator complex protein LAMTOR2	LTOR2_HUMAN	Q9Y2Q5	LAMTOR2	13.5	36	4	44	No
Mitochondrial carrier homolog 2	MTCH2_HUMAN	Q9Y6C9	MTCH2	33.3	25	6	44	No
Extended synaptotagmin-2	ESYT2_HUMAN	A0FGR8	ESYT2	102.3	18	11	43	No
Inositol polyphosphate 4-phosphatase type II	INP4B_HUMAN	O15327	INPP4B	104.7	11	6	43	No
Galactose-1-phosphate uridylyltransferase	GALT_HUMAN	P07902	GALT	43.3	10	3	43	No
Serpin B4	SPB4_HUMAN	P48594	SERPINB4	44.8	35	6	43	No
60S ribosomal protein L8	RL8_HUMAN	P62917	RPL8	28	26	5	43	No
Splicing factor 3A subunit 3	SF3A3_HUMAN	Q12874	SF3A3	58.8	12	4	43	No
Inactive phospholipase C-like protein 1	PLCL1_HUMAN	Q15111	PLCL1	122.6	10	8	43	No
MAP kinase-activated protein kinase 3	MAPK3_HUMAN	Q16644	MAPKAPK3	43	10	2	43	No
NAD-dependent protein deacetylase sirtuin-2	SIR2_HUMAN	Q8IXJ6	SIRT2	43.2	21	7	43	No
Nuclear pore complex protein Nup93	NUP93_HUMAN	Q8N1F7	NUP93	93.4	5	4	43	No
ATP-binding cassette sub-family F member 1	ABCF1_HUMAN	Q8NE71	ABCF1	95.9	5	4	43	No
DnaJ homolog subfamily A member 4	DNJA4_HUMAN	Q8WW22	DNAJA4	44.8	8	4	43	No
Uveal autoantigen with coiled-coil domains and ankyrin repeats	UACA_HUMAN	Q9BZF9	UACA	162.4	6	9	43	No
CXXC motif containing zinc binding protein	CZIB_HUMAN	Q9NWV4	CZIB	18	40	5	43	No
Band 4.1-like protein 3	E41L3_HUMAN	Q9Y2J2	EPB41L3	120.6	5	4	43	No
Pro-cathepsin H [Cleaved into: Cathepsin H mini chain; Cathepsin H	CATH_HUMAN	P09668	CTSH	37.4	20	6	42	No
Eukaryotic peptide chain release factor GTP-binding subunit ERF3A	ERF3A_HUMAN	P15170	GSPT1	55.7	10	5	42	No
High mobility group protein B2	HMGB2_HUMAN	P26583	HMGB2	24	26	4	42	No
Protein phosphatase 1F	PPM1F_HUMAN	P49593	PPM1F	49.8	18	5	42	No
Casein kinase II subunit beta	CSK2B_HUMAN	P67870	CSNK2B	24.9	21	4	42	No
Apoptosis regulator BAX	BAX_HUMAN	Q07812	BAX	21.2	29	4	42	No
ARF GTPase-activating protein GIT2	GIT2_HUMAN	Q14161	GIT2	84.5	14	7	42	No
Acyl-CoA-binding domain-containing protein 5	ACBD5_HUMAN	Q5T8D3	ACBD5	60.1	10	5	42	No
Transmembrane emp24 domain-containing protein 4	TMED4_HUMAN	Q7Z7H5	TMED4	25.9	33	6	42	No
Prostaglandin reductase 3	PTGR3_HUMAN	Q8N4Q0	ZADH2	40.1	18	5	42	No
Chloride intracellular channel protein 5	CLIC5_HUMAN	Q9NZA1	CLIC5	46.5	25	6	42	No
U6 snRNA-associated Sm-like protein LSm7	LSM7_HUMAN	Q9UK45	LSM7	11.6	22	2	42	No
Phenylalanine--tRNA ligase alpha subunit	SYFA_HUMAN	Q9Y285	FARSA	57.5	21	8	42	No
Cytochrome b5 type B	CYB5B_HUMAN	O43169	CYB5B	16.3	40	4	41	No
Trafficking protein particle complex subunit 3	TPPC3_HUMAN	O43617	TRAPPC3	20.3	12	2	41	No
Eukaryotic translation initiation factor 3 subunit G	EIF3G_HUMAN	O75821	EIF3G	35.6	26	8	41	No

NADPH:adrenodoxin oxidoreductase, mitochondrial	ADRO_HUMAN	P22570	FDXR	53.8	29	10	41	No
Serpin B3	SPB3_HUMAN	P29508	SERPINB3	44.5	32	7	41	No
Endothelin-converting enzyme 1	ECE1_HUMAN	P42892	ECE1	87.1	11	7	41	No
Protein OS-9	OS9_HUMAN	Q13438	OS9	75.5	15	6	41	No
Phosphoglucomutase-2	PGM2_HUMAN	Q96G03	PGM2	68.2	15	7	41	Yes
Haloacid dehalogenase-like hydrolase domain-containing protein 2	HDHD2_HUMAN	Q9H0R4	HDHD2	28.5	23	4	41	Yes
Spondin-1	SPON1_HUMAN	Q9HCB6	SPON1	90.9	4	2	41	No
Mitochondrial chaperone BCS1	BCS1_HUMAN	Q9Y276	BCS1L	47.5	26	8	41	No
Immunoglobulin heavy variable 3-72	HV372_HUMAN	A0A0B4J1Y9	IGHV3-72	13.2	15	2	40	No
Death-associated protein kinase 3	DAPK3_HUMAN	O43293	DAPK3	52.5	8	4	40	No
Epsilon-sarcoglycan	SGCE_HUMAN	O43556	SGCE	49.8	22	7	40	No
Complement C5	CO5_HUMAN	P01031	C5	188.2	3	5	40	No
Adenylosuccinate lyase	PUR8_HUMAN	P30566	ADSL	54.9	6	3	40	No
Translocon-associated protein subunit alpha	SSRA_HUMAN	P43307	SSR1	32.2	7	2	40	No
Protein transport protein Sec24C	SC24C_HUMAN	P53992	SEC24C	118.2	9	7	40	No
mRNA export factor	RAE1L_HUMAN	P78406	RAE1	40.9	28	9	40	No
Disks large homolog 1	DLG1_HUMAN	Q12959	DLG1	100.4	7	6	40	Yes
Stromal interaction molecule 1	STIM1_HUMAN	Q13586	STIM1	77.4	11	5	40	No
Cleavage and polyadenylation specificity factor subunit 6	CPSF6_HUMAN	Q16630	CPSF6	59.2	7	3	40	No
WD40 repeat-containing protein SMU1	SMU1_HUMAN	Q2TAY7	SMU1	57.5	4	2	40	No
Olfactomedin-like protein 1	OLFL1_HUMAN	Q6UWY5	OLFML1	45.9	21	7	40	No
Nucleoporin p54	NUP54_HUMAN	Q7Z3B4	NUP54	55.4	15	6	40	No
ELKS/Rab6-interacting/CAST family member 1	RB6I2_HUMAN	Q8IUD2	ERC1	128	10	8	40	No
Exocyst complex component 8	EXOC8_HUMAN	Q8IYI6	EXOC8	81.7	9	5	40	No
Golgi-specific brefeldin A-resistance guanine nucleotide exchange factor 1	GBF1_HUMAN	Q92538	GBF1	206.3	2	4	40	No
Importin-5	IPO5_HUMAN	O00410	IPO5	123.6	9	7	39	No
Integrin alpha-5	ITA5_HUMAN	P08648	ITGA5	114.5	10	7	39	No
Rho-related GTP-binding protein RhoG	RHOG_HUMAN	P84095	RHOG	21.3	26	2	39	No
Peroxisomal acyl-coenzyme A oxidase 1	ACOX1_HUMAN	Q15067	ACOX1	74.4	14	7	39	No
Myosin light chain kinase, smooth muscle	MYLK_HUMAN	Q15746	MYLK	210.6	5	8	39	No
COMM domain-containing protein 6	COMD6_HUMAN	Q7Z4G1	COMMD6	9.6	26	2	39	No
Protein phosphatase 1 regulatory subunit 14C	PP14C_HUMAN	Q8TAE6	PPP1R14C	17.8	33	3	39	No
Geranylgeranyl transferase type-2 subunit alpha	PGTA_HUMAN	Q92696	RABGGTA	65	10	5	39	No
Nicalin	NCLN_HUMAN	Q969V3	NCLN	62.9	17	7	39	No
STE20-like serine/threonine-protein kinase	SLK_HUMAN	Q9H2G2	SLK	142.6	12	13	39	No

Mitochondrial potassium channel ATP-binding subunit	MITOS_HUMAN	Q9NUT2	ABCB8	79.9	5	3	39	No
ADP-ribosylation factor-like protein 8B	ARL8B_HUMAN	Q9NVJ2	ARL8B	21.5	53	3	39	No
EMILIN-1	EMIL1_HUMAN	Q9Y6C2	EMILIN1	106.6	13	10	39	No
Cathepsin G	CATG_HUMAN	P08311	CTSG	28.8	24	4	38	No
CD63 antigen	CD63_HUMAN	P08962	CD63	25.6	21	4	38	No
40S ribosomal protein S15a	RS15A_HUMAN	P62244	RPS15A	14.8	38	5	38	No
Ubiquitin-conjugating enzyme E2 L3	UB2L3_HUMAN	P68036	UBE2L3	17.9	32	4	38	No
28S ribosomal protein S34, mitochondrial	RT34_HUMAN	P82930	MRPS34	25.6	24	4	38	No
60S ribosomal protein L24	RL24_HUMAN	P83731	RPL24	17.8	24	4	38	No
Inositol polyphosphate-5-phosphatase A	I5P1_HUMAN	Q14642	INPP5A	47.8	6	3	38	No
Sarcospan	SSPN_HUMAN	Q14714	SSPN	26.6	10	2	38	No
NADH dehydrogenase	NDUF6_HUMAN	Q330K2	NDUFAF6	38.2	14	4	38	No
Pre-mRNA-processing-splicing factor 8	PRP8_HUMAN	Q6P2Q9	PRPF8	273.4	3	6	38	No
C-type lectin domain family 14 member A	CLC14_HUMAN	Q86T13	CLEC14A	51.6	10	3	38	No
Histone-lysine N-methyltransferase SETD7	SETD7_HUMAN	Q8WTS6	SETD7	40.7	12	4	38	No
FAS-associated factor 2	FAF2_HUMAN	Q96CS3	FAF2	52.6	11	4	38	No
Histidine triad nucleotide-binding protein 3	HINT3_HUMAN	Q9NQE9	HINT3	20.3	55	7	38	No
Actin-related protein 10	ARP10_HUMAN	Q9NZ32	ACTR10	46.3	8	4	38	No
Serine/threonine-protein kinase 24	STK24_HUMAN	Q9Y6E0	STK24	49.3	17	7	38	No
Uncharacterized protein C16orf96	CP096_HUMAN	A6NNT2	C16orf96	125	1	2	37	No
Plexin-B2	PLXB2_HUMAN	O15031	PLXNB2	205	7	10	37	No
Sorting nexin-2	SNX2_HUMAN	O60749	SNX2	58.4	28	11	37	No
Cytochrome b-245 heavy chain	CY24B_HUMAN	P04839	CYBB	65.3	6	4	37	No
Complement decay-accelerating factor	DAF_HUMAN	P08174	CD55	41.4	15	7	37	No
Intercellular adhesion molecule 2	ICAM2_HUMAN	P13598	ICAM2	30.6	12	3	37	No
IST1 homolog	IST1_HUMAN	P53990	IST1	39.7	5	2	37	No
Sequestosome-1	SQSTM_HUMAN	Q13501	SQSTM1	47.7	12	4	37	No
Ecto-ADP-ribosyltransferase 3	NAR3_HUMAN	Q13508	ART3	43.9	18	6	37	No
DNL-type zinc finger protein	DNLZ_HUMAN	Q5SXM8	DNLZ	19.2	21	3	37	No
Vacuolar protein sorting-associated protein 13C	VP13C_HUMAN	Q709C8	VPS13C	422.1	1	2	37	No
Protein FAM98A	FA98A_HUMAN	Q8NCA5	FAM98A	55.4	7	2	37	No
Ubiquitin carboxyl-terminal hydrolase 13	UBP13_HUMAN	Q92995	USP13	97.3	14	6	37	No
Thioredoxin domain-containing protein 17	TXD17_HUMAN	Q9BRA2	TXNDC17	13.9	20	2	37	No
Methyltransferase-like protein 7A	MET7A_HUMAN	Q9H8H3	METTL7A	28.3	22	3	37	No
Gamma-synuclein	SYUG_HUMAN	O76070	SNCG	13.3	33	4	36	No
Adenosine deaminase	ADA_HUMAN	P00813	ADA	40.7	22	7	36	No

Immunoglobulin heavy variable 4-39	HV439_HUMAN	P01824	IGHV4-39	13.9	20	2	36	No
HLA class II histocompatibility antigen, DR alpha chain	DRA_HUMAN	P01903	HLA-DRA	28.6	28	5	36	Yes
Tetranectin	TETN_HUMAN	P05452	CLEC3B	22.5	9	2	36	No
Sulfotransferase 1A3	ST1A3_HUMAN	P0DMM9	SULT1A3	34.2	20	4	36	No
Protein 4.1	EPB41_HUMAN	P11171	EPB41	97	11	6	36	No
Importin subunit alpha-5	IMA5_HUMAN	P52294	KPNA1	60.2	13	2	36	No
ATP-citrate synthase	ACLY_HUMAN	P53396	ACLY	120.8	11	9	36	No
Monocarboxylate transporter 1	MOT1_HUMAN	P53985	SLC16A1	53.9	11	4	36	No
60S ribosomal protein L26	RL26_HUMAN	P61254	RPL26	17.2	28	6	36	No
40S ribosomal protein S16	RS16_HUMAN	P62249	RPS16	16.4	35	7	36	No
Casein kinase II subunit alpha	CSK21_HUMAN	P68400	CSNK2A1	45.1	26	7	36	Yes
Cullin-1	CUL1_HUMAN	Q13616	CUL1	89.6	9	7	36	No
Striated muscle preferentially expressed protein kinase	SPEG_HUMAN	Q15772	SPEG	354.1	4	7	36	No
2',5'-phosphodiesterase 12	PDE12_HUMAN	Q6L8Q7	PDE12	67.3	16	6	36	No
Leucine-rich repeat-containing protein 57	LRC57_HUMAN	Q8N9N7	LRRC57	26.7	13	3	36	No
AFG1-like ATPase	AFG1L_HUMAN	Q8WV93	AFG1L	54.8	4	3	36	No
Thioredoxin-related transmembrane protein 1	TMX1_HUMAN	Q9H3N1	TMX1	31.8	16	4	36	No
Golgi resident protein GCP60	GCP60_HUMAN	Q9H3P7	ACBD3	60.6	22	6	36	No
Dipeptidyl peptidase 3	DPP3_HUMAN	Q9NY33	DPP3	82.5	8	4	36	No
Glutamyl-tRNA	GATC_HUMAN	O43716	GATC	15.1	47	4	35	No
HIV Tat-specific factor 1	HTSF1_HUMAN	O43719	HTATSF1	85.8	8	6	35	No
Heat shock factor-binding protein 1	HSBP1_HUMAN	O75506	HSBP1	8.5	58	3	35	No
Band 3 anion transport protein	B3AT_HUMAN	P02730	SLC4A1	101.7	6	4	35	No
Melanotransferrin	TRFM_HUMAN	P08582	MELTF	80.2	20	8	35	No
Complement C1s subcomponent	C1S_HUMAN	P09871	C1S	76.6	10	3	35	No
Lipopolysaccharide-binding protein	LBP_HUMAN	P18428	LBP	53.4	7	4	35	No
Small nuclear ribonucleoprotein Sm D3	SMD3_HUMAN	P62318	SNRPD3	13.9	32	3	35	No
Platelet-activating factor acetylhydrolase IB subunit beta	PA1B2_HUMAN	P68402	PAFAH1B2	25.6	16	3	35	Yes
Dystonin	DYST_HUMAN	Q03001	DST	860.1	1	8	35	No
Clathrin interactor 1	EPN4_HUMAN	Q14677	CLINT1	68.2	9	4	35	No
CLIP-associating protein 1	CLAP1_HUMAN	Q7Z460	CLASP1	169.3	5	4	35	No
Actin-related protein 2/3 complex subunit 5-like protein	ARP5L_HUMAN	Q9BPX5	ARPC5L	16.9	33	4	35	No
Ribokinase	RBSK_HUMAN	Q9H477	RBKS	34.1	9	3	35	No
Exocyst complex component 1	EXOC1_HUMAN	Q9NV70	EXOC1	101.9	7	5	35	No
Switch-associated protein 70	SWP70_HUMAN	Q9UH65	SWAP70	69	16	8	35	No
Nuclease EXOG, mitochondrial	EXOG_HUMAN	Q9Y2C4	EXOG	41.1	13	3	35	No

Mannose-1-phosphate guanyltransferase beta	GMPPB_HUMAN	Q9Y5P6	GMPPB	39.8	20	5	35	No
Receptor-type tyrosine-protein phosphatase C	PTPRC_HUMAN	P08575	PTPRC	147.4	8	10	34	No
Alkyldihydroxyacetonephosphate synthase, peroxisomal	ADAS_HUMAN	O00116	AGPS	72.9	19	7	34	No
Choline-phosphate cytidylyltransferase A	PCY1A_HUMAN	P49585	PCYT1A	41.7	13	4	34	No
Mesencephalic astrocyte-derived neurotrophic factor	MANF_HUMAN	P55145	MANF	20.7	47	11	34	No
DnaJ homolog subfamily C member 3	DNJC3_HUMAN	Q13217	DNAJC3	57.5	13	5	34	No
Nucleoporin Nup43	NUP43_HUMAN	Q8NFH3	NUP43	42.1	15	4	34	No
Bcl2-associated agonist of cell death	BAD_HUMAN	Q92934	BAD	18.4	35	4	34	No
Ribosome-releasing factor 2, mitochondrial	RRF2M_HUMAN	Q969S9	GFM2	86.5	11	7	34	No
Coiled-coil-helix-coiled-coil-helix domain-containing protein 1	CHCH1_HUMAN	Q96BP2	CHCHD1	13.5	39	4	34	No
Coronin-1B	COR1B_HUMAN	Q9BR76	CORO1B	54.2	18	6	34	No
Vacuolar protein sorting-associated protein 28 homolog	VPS28_HUMAN	Q9UK41	VPS28	25.4	33	8	34	No
Aspartyl aminopeptidase	DNPEP_HUMAN	Q9ULA0	DNPEP	52.4	12	6	34	No
AH receptor-interacting protein	AIP_HUMAN	O00170	AIP	37.6	25	6	33	No
Cytochrome c oxidase subunit 7A-related protein, mitochondrial	COX7R_HUMAN	O14548	COX7A2L	12.6	56	5	33	No
Beta-galactosidase	BGAL_HUMAN	P16278	GLB1	76	8	4	33	No
Fumarylacetoacetase	FAAA_HUMAN	P16930	FAH	46.3	19	6	33	No
Cysteine and glycine-rich protein 1	CSRP1_HUMAN	P21291	CSRP1	20.6	36	5	33	No
NHP2-like protein 1	NH2L1_HUMAN	P55769	SNU13	14.2	29	4	33	Yes
60S ribosomal protein L15	RL15_HUMAN	P61313	RPL15	24.1	22	5	33	No
Brain acid soluble protein 1	BASP1_HUMAN	P80723	BASP1	22.7	57	6	33	No
UBX domain-containing protein 1	UBXN1_HUMAN	Q04323	UBXN1	33.3	21	4	33	No
CapZ-interacting protein	CPZIP_HUMAN	Q6JBY9	RCSD1	44.5	13	4	33	No
Thrombospondin type-1 domain-containing protein 4	THSD4_HUMAN	Q6ZMP0	THSD4	112.4	6	5	33	No
Syntaxin-12	STX12_HUMAN	Q86Y82	STX12	31.6	21	5	33	No
Phospholipase A2 group XV	PAG15_HUMAN	Q8NCC3	PLA2G15	46.6	10	3	33	No
Glucosamine-6-phosphate isomerase 2	GNPI2_HUMAN	Q8TDQ7	GNPDA2	31.1	37	3	33	No
Peptidyl-prolyl cis-trans isomerase FKBP10	FKB10_HUMAN	Q96AY3	FKBP10	64.2	11	5	33	No
Tumor susceptibility gene 101 protein	TS101_HUMAN	Q99816	TSG101	43.9	18	5	33	No
Translational activator of cytochrome c oxidase 1	TACO1_HUMAN	Q9BSH4	TACO1	32.5	28	6	33	No
28S ribosomal protein S17, mitochondrial	RT17_HUMAN	Q9Y2R5	MRPS17	14.5	38	3	33	No
Protein MEMO1	MEMO1_HUMAN	Q9Y316	MEMO1	33.7	16	3	33	No
U6 snRNA-associated Sm-like protein LSm2	LSM2_HUMAN	Q9Y333	LSM2	10.8	47	3	33	No
Immunoglobulin kappa variable 3D-11	KVD11_HUMAN	A0A0A0MRZ8	IGKV3D-11	12.6	26	2	32	No
Unconventional myosin-Ib	MYO1B_HUMAN	O43795	MYO1B	131.9	3	3	32	No
Phosphatidate cytidylyltransferase 2	CDS2_HUMAN	O95674	CDS2	51.4	11	3	32	No

Collagen alpha-1	CO1A1_HUMAN	P02452	COL1A1	138.9	6	8	32	No
Lysosomal acid glucosylceramidase	GLCM_HUMAN	P04062	GBA	59.7	8	4	32	No
Eukaryotic translation initiation factor 4E	IF4E_HUMAN	P06730	EIF4E	25.1	10	2	32	No
Mannosyl-oligosaccharide glucosidase	MOGS_HUMAN	Q13724	MOGS	91.9	5	4	32	No
KN motif and ankyrin repeat domain-containing protein 3	KANK3_HUMAN	Q6NY19	KANK3	88.4	7	5	32	No
Protein arginine methyltransferase NDUFAF7, mitochondrial	NDUF7_HUMAN	Q7L592	NDUFAF7	49.2	9	3	32	No
Junctional adhesion molecule C	JAM3_HUMAN	Q9BX67	JAM3	35	8	2	32	No
Constitutive coactivator of PPAR-gamma-like protein 1	F120A_HUMAN	Q9NZB2	FAM120A	121.8	8	8	32	No
Ubiquitin-fold modifier-conjugating enzyme 1	UFC1_HUMAN	Q9Y3C8	UFC1	19.4	20	4	32	No
Epsin-1	EPN1_HUMAN	Q9Y6I3	EPN1	60.3	15	6	32	No
4F2 cell-surface antigen heavy chain	4F2_HUMAN	P08195	SLC3A2	68	9	4	31	No
Mannan-binding lectin serine protease 1	MASP1_HUMAN	P48740	MASP1	79.2	13	5	31	No
Phosphatidylinositol transfer protein alpha isoform	PIPNA_HUMAN	Q00169	PITPNA	31.8	19	2	31	No
60S ribosomal protein L18	RL18_HUMAN	Q07020	RPL18	21.6	20	3	31	No
Endoribonuclease LACTB2	LACB2_HUMAN	Q53H82	LACTB2	32.8	39	8	31	No
Golgin subfamily A member 5	GOGA5_HUMAN	Q8TBA6	GOLGA5	83	11	7	31	No
Acyl-coenzyme A thioesterase 11	ACO11_HUMAN	Q8WXI4	ACOT11	68.4	3	2	31	No
Mitochondrial fission regulator 1-like	MFR1L_HUMAN	Q9H019	MTFR1L	31.9	19	4	31	No
5'-AMP-activated protein kinase subunit gamma-2	AAKG2_HUMAN	Q9UGJ0	PRKAG2	63	7	3	31	No
FAS-associated factor 1	FAF1_HUMAN	Q9UNN5	FAF1	73.9	8	5	31	No
Importin subunit alpha-7	IMA7_HUMAN	O60684	KPNA6	60	16	3	30	No
CAP-Gly domain-containing linker protein 1	CLIP1_HUMAN	P30622	CLIP1	162.1	4	5	30	No
Eukaryotic translation initiation factor 2 subunit 3	IF2G_HUMAN	P41091	EIF2S3	51.1	16	5	30	No
Secernin-3	SCRN3_HUMAN	Q0VDG4	SCRN3	48.5	8	3	30	No
Adipogenesis regulatory factor	ADIRF_HUMAN	Q15847	ADIRF	7.9	80	6	30	No
Magnesium-dependent phosphatase 1	MGDP1_HUMAN	Q86V88	MDP1	20.1	19	3	30	No
Immunity-related GTPase family Q protein	IRGQ_HUMAN	Q8WZA9	IRGQ	62.7	6	3	30	No
WW domain-binding protein 2	WBP2_HUMAN	Q969T9	WBP2	28.1	10	2	30	No
Chloride intracellular channel protein 6	CLIC6_HUMAN	Q96NY7	CLIC6	73	20	5	30	No
FYVE and coiled-coil domain-containing protein 1	FYCO1_HUMAN	Q9BQS8	FYCO1	166.9	6	6	30	No
PDZ and LIM domain protein 7	PDLI7_HUMAN	Q9NR12	PDLIM7	49.8	8	3	30	No
Vacuolar protein sorting-associated protein 45	VPS45_HUMAN	Q9NRW7	VPS45	65	4	2	30	No
Protein lin-7 homolog C	LIN7C_HUMAN	Q9NUP9	LIN7C	21.8	14	3	30	No
Actin-related protein 2/3 complex subunit 1B	ARC1B_HUMAN	O15143	ARPC1B	40.9	19	5	29	No
Actin-related protein 2/3 complex subunit 5	ARPC5_HUMAN	O15511	ARPC5	16.3	44	3	29	No
Eukaryotic translation initiation factor 1b	EIF1B_HUMAN	O60739	EIF1B	12.8	28	2	29	Yes

Protein cordon-bleu	COBL_HUMAN	O75128	COBL	135.5	4	4	29	No
C-reactive protein [Cleaved into: C-reactive protein	CRP_HUMAN	P02741	CRP	25	16	3	29	No
Complement factor I	CFAI_HUMAN	P05156	CFI	65.7	10	5	29	No
Protein PET100 homolog, mitochondrial	PT100_HUMAN	P0DJ07	PET100	9.1	23	2	29	No
60S ribosomal protein L29	RL29_HUMAN	P47914	RPL29	17.7	14	2	29	No
Fragile X mental retardation syndrome-related protein 2	FXR2_HUMAN	P51116	FXR2	74.2	16	7	29	No
28S ribosomal protein S25, mitochondrial	RT25_HUMAN	P82663	MRPS25	20.1	43	5	29	No
Guanylate cyclase soluble subunit beta-1	GCYB1_HUMAN	Q02153	GUCY1B1	70.5	3	2	29	No
Aminoacyl tRNA synthase complex-interacting multifunctional protein 2	AIMP2_HUMAN	Q13155	AIMP2	35.3	14	3	29	No
Protein CASP	CASP_HUMAN	Q13948	CUX1	77.4	19	10	29	No
Mitochondrial 10-formyltetrahydrofolate dehydrogenase	AL1L2_HUMAN	Q3SY69	ALDH1L2	101.7	7	3	29	No
Dipeptidyl peptidase 9	DPP9_HUMAN	Q86TI2	DPP9	98.2	5	4	29	No
Malonyl-CoA-acyl carrier protein transacylase, mitochondrial	FABD_HUMAN	Q8IVS2	MCAT	42.9	14	3	29	No
Ubiquitin-associated domain-containing protein 1	UBAC1_HUMAN	Q9BSL1	UBAC1	45.3	16	4	29	No
Transmembrane protein 43	TMM43_HUMAN	Q9BTV4	TMEM43	44.8	14	4	29	No
Target of rapamycin complex subunit LST8	LST8_HUMAN	Q9BVC4	MLST8	35.9	9	2	29	No
Ubiquinol-cytochrome-c reductase complex assembly factor 1	UQCC1_HUMAN	Q9NVA1	UQCC1	34.6	13	3	29	No
Peflin	PEF1_HUMAN	Q9UBV8	PEF1	30.4	7	3	29	No
Protein CDV3 homolog	CDV3_HUMAN	Q9UKY7	CDV3	27.3	37	4	29	No
Serine/threonine-protein phosphatase 6 catalytic subunit	PPP6_HUMAN	O00743	PPP6C	35.1	16	5	28	No
LYR motif-containing protein 1	LYRM1_HUMAN	O43325	LYRM1	14.3	34	4	28	No
Gamma-interferon-inducible lysosomal thiol reductase	GILT_HUMAN	P13284	IFI30	27.9	8	2	28	No
Small nuclear ribonucleoprotein-associated proteins B and B'	RSMB_HUMAN	P14678	SNRPB	24.6	23	6	28	No
Guanylate-binding protein 1	GBP1_HUMAN	P32455	GBP1	67.9	5	3	28	No
ADP-ribosylation factor-like protein 3	ARL3_HUMAN	P36405	ARL3	20.4	31	5	28	No
Ran-specific GTPase-activating protein	RANG_HUMAN	P43487	RANBP1	23.3	10	2	28	No
Galectin-7	LEG7_HUMAN	P47929	LGALS7	15.1	63	6	28	No
Junctional adhesion molecule B	JAM2_HUMAN	P57087	JAM2	33.2	13	3	28	No
Protocadherin-1	PCDH1_HUMAN	Q08174	PCDH1	114.7	2	2	28	No
Cytoskeleton-associated protein 5	CKAP5_HUMAN	Q14008	CKAP5	225.4	3	5	28	No
Triokinase/FMN cyclase	TKFC_HUMAN	Q3LXA3	TKFC	58.9	6	3	28	No
Beta-1,3-glucosyltransferase	B3GLT_HUMAN	Q6Y288	B3GLCT	56.5	12	5	28	No
Protein Hook homolog 3	HOOK3_HUMAN	Q86VS8	HOOK3	83.1	9	5	28	No
Nesprin-1	SYNE1_HUMAN	Q8NF91	SYNE1	1010.5	1	5	28	No
Putative phospholipase B-like 2	PLBL2_HUMAN	Q8NHP8	PLBD2	65.4	7	4	28	No

Pyridoxal phosphate phosphatase	PLPP_HUMAN	Q96GD0	PDXP	31.7	20	4	28	No
RWD domain-containing protein 1	RWDD1_HUMAN	Q9H446	RWDD1	27.9	12	3	28	No
39S ribosomal protein L15, mitochondrial	RM15_HUMAN	Q9P015	MRPL15	33.4	23	5	28	No
Microtubule-actin cross-linking factor 1, isoforms 1/2/3/5	MACF1_HUMAN	Q9UPN3	MACF1	837.8	1	2	28	No
Lysosomal alpha-mannosidase	MA2B1_HUMAN	O00754	MAN2B1	113.7	5	4	27	No
Transforming growth factor beta-1-induced transcript 1 protein	TGFI1_HUMAN	O43294	TGFB1I1	49.8	13	4	27	No
Coiled-coil domain-containing protein 22	CCD22_HUMAN	O60826	CCDC22	70.7	18	7	27	No
Protein phosphatase 1B	PPM1B_HUMAN	O75688	PPM1B	52.6	14	3	27	No
Bisphosphoglycerate mutase	PMGE_HUMAN	P07738	BPGM	30	23	5	27	No
Cytochrome c oxidase subunit 6A1, mitochondrial	CX6A1_HUMAN	P12074	COX6A1	12.1	43	2	27	No
Neuromodulin	NEUM_HUMAN	P17677	GAP43	24.8	8	2	27	No
Filaggrin	FILA_HUMAN	P20930	FLG	434.9	4	5	27	No
Trifunctional purine biosynthetic protein adenosine-3 [Includes: Phosphoribosylamine--glycine ligase	PUR2_HUMAN	P22102	GART	107.7	6	5	27	No
60S ribosomal protein L3	RL3_HUMAN	P39023	RPL3	46.1	7	2	27	No
Eukaryotic translation initiation factor 5	IF5_HUMAN	P55010	EIF5	49.2	11	4	27	No
Desmocollin-2	DSC2_HUMAN	Q02487	DSC2	99.9	5	3	27	Yes
Voltage-dependent L-type calcium channel subunit alpha-1C	CAC1C_HUMAN	Q13936	CACNA1C	248.8	1	2	27	No
Rho guanine nucleotide exchange factor 7	ARHG7_HUMAN	Q14155	ARHGEF7	90	9	5	27	No
Deoxyguanosine kinase, mitochondrial	DGUOK_HUMAN	Q16854	DGUOK	32	26	5	27	No
Sickle tail protein homolog	SKT_HUMAN	Q5T5P2	KIAA1217	214	2	3	27	No
Acyl-CoA dehydrogenase family member 10	ACD10_HUMAN	Q6JQN1	ACAD10	118.8	5	4	27	No
Nicotinate phosphoribosyltransferase	PNCB_HUMAN	Q6XQN6	NAPRT	57.5	15	5	27	No
Mitochondrial antiviral-signaling protein	MAVS_HUMAN	Q7Z434	MAVS	56.5	15	4	27	No
Methionine-R-sulfoxide reductase B3	MSRB3_HUMAN	Q8IXL7	MSRB3	20.7	43	4	27	No
Leucine-rich repeat-containing protein 59	LRC59_HUMAN	Q96AG4	LRRC59	34.9	18	5	27	No
Calcineurin B homologous protein 3	CHP3_HUMAN	Q96BS2	TESC	24.7	31	5	27	No
Charged multivesicular body protein 6	CHMP6_HUMAN	Q96FZ7	CHMP6	23.5	25	4	27	No
Mitochondrial import inner membrane translocase subunit TIM16	TIM16_HUMAN	Q9Y3D7	PAM16	13.8	21	3	27	No
U6 snRNA-associated Sm-like protein LSm8	LSM8_HUMAN	O95777	LSM8	10.4	36	3	26	No
Lactotransferrin	TRFL_HUMAN	P02788	LTF	78.1	10	7	26	No
Arginase-1	ARGI1_HUMAN	P05089	ARG1	34.7	8	3	26	No
ATP-dependent DNA helicase Q1	RECQ1_HUMAN	P46063	RECQL	73.4	7	5	26	No
Carnitine O-palmitoyltransferase 1, liver isoform	CPT1A_HUMAN	P50416	CPT1A	88.3	8	5	26	No
IgG receptor FcRn large subunit p51	FCGRN_HUMAN	P55899	FCGRT	39.7	11	3	26	No
Nucleoside diphosphate kinase 3	NDK3_HUMAN	Q13232	NME3	19	39	6	26	No

Cullin-2	CUL2_HUMAN	Q13617	CUL2	86.9	4	3	26	No
MAM domain-containing protein 2	MAMC2_HUMAN	Q7Z304	MAMDC2	77.5	9	5	26	No
Protein LYRIC	LYRIC_HUMAN	Q86UE4	MTDH	63.8	9	3	26	No
39S ribosomal protein L50, mitochondrial	RM50_HUMAN	Q8N5N7	MRPL50	18.3	41	4	26	No
Protein phosphatase PTC7 homolog	PPTC7_HUMAN	Q8NI37	PPTC7	32.6	25	5	26	No
Zinc transporter SLC39A7	S39A7_HUMAN	Q92504	SLC39A7	50.1	7	4	26	No
SWI/SNF-related matrix-associated actin-dependent regulator of chromatin subfamily E member 1	SMCE1_HUMAN	Q969G3	SMARCE1	46.6	18	6	26	No
Far upstream element-binding protein 3	FUBP3_HUMAN	Q96I24	FUBP3	61.6	14	3	26	No
Acidic leucine-rich nuclear phosphoprotein 32 family member E	AN32E_HUMAN	Q9BTT0	ANP32E	30.7	17	3	26	No
ATP-binding cassette sub-family B member 10, mitochondrial	ABCBA_HUMAN	Q9NRK6	ABCB10	79.1	6	4	26	No
Leucine--tRNA ligase, cytoplasmic	SYLC_HUMAN	Q9P2J5	LARS1	134.4	2	3	26	No
E3 ubiquitin-protein ligase HECTD1	HECD1_HUMAN	Q9ULT8	HECTD1	289.2	2	4	26	No
Thyroid hormone receptor-associated protein 3	TR150_HUMAN	Q9Y2W1	THRAP3	108.6	3	4	26	No
Chromatin target of PRMT1 protein	CHTOP_HUMAN	Q9Y3Y2	CHTOP	26.4	16	3	26	No
Syntenin-1	SDCB1_HUMAN	O00560	SDCBP	32.4	7	2	25	No
Sorting nexin-3	SNX3_HUMAN	O60493	SNX3	18.8	36	5	25	No
6-phosphofructo-2-kinase/fructose-2,6-bisphosphatase 2	F262_HUMAN	O60825	PFKFB2	58.4	10	4	25	No
Cullin-associated NEDD8-dissociated protein 2	CAND2_HUMAN	O75155	CAND2	135.2	5	5	25	No
Synaptosomal-associated protein 29	SNP29_HUMAN	O95721	SNAP29	29	16	3	25	No
Phosphatidylserine lipase ABHD16A	ABHGA_HUMAN	O95870	ABHD16A	63.2	4	3	25	No
Chymase	CMA1_HUMAN	P23946	CMA1	27.3	22	3	25	No
Protein farnesyltransferase/geranylgeranyltransferase type-1 subunit alpha	FNTA_HUMAN	P49354	FNTA	44.4	12	4	25	No
Protoporphyrinogen oxidase	PPOX_HUMAN	P50336	PPOX	50.7	19	8	25	No
Arf-GAP domain and FG repeat-containing protein 1	AGFG1_HUMAN	P52594	AGFG1	58.2	8	4	25	No
Lysozyme C	LYSC_HUMAN	P61626	LYZ	16.5	14	2	25	No
39S ribosomal protein L28, mitochondrial	RM28_HUMAN	Q13084	MRPL28	30.1	10	2	25	No
60S ribosomal protein L3-like	RL3L_HUMAN	Q92901	RPL3L	46.3	7	2	25	No
Leucine-rich repeat-containing protein 39	LRC39_HUMAN	Q96DD0	LRRC39	38.8	15	5	25	No
Vacuolar protein-sorting-associated protein 25	VPS25_HUMAN	Q9BRG1	VPS25	20.7	20	3	25	No
Protein arginine N-methyltransferase 1	ANM1_HUMAN	Q99873	PRMT1	42.4	23	7	24	No
Ubiquitin-like modifier-activating enzyme 6	UBA6_HUMAN	A0AVT1	UBA6	117.9	2	3	24	No
Charged multivesicular body protein 2a	CHM2A_HUMAN	O43633	CHMP2A	25.1	17	4	24	No
Immunoglobulin kappa variable 3-15	KV315_HUMAN	P01624	IGKV3-15	12.5	26	2	24	No
X-box-binding protein 1	XBP1_HUMAN	P17861	XBP1	28.7	13	2	24	No

Splicing factor U2AF 65 kDa subunit	U2AF2_HUMAN	P26368	U2AF2	53.5	5	2	24	No
Translation initiation factor IF-2, mitochondrial	IF2M_HUMAN	P46199	MTIF2	81.3	8	4	24	No
Triadin	TRDN_HUMAN	Q13061	TRDN	81.5	6	4	24	No
Phosphorylase b kinase gamma catalytic chain, skeletal muscle/heart isoform	PHKG1_HUMAN	Q16816	PHKG1	45	21	5	24	No
AP2-associated protein kinase 1	AAK1_HUMAN	Q2M2I8	AAK1	103.8	5	3	24	No
FK506-binding protein 15	FKB15_HUMAN	Q5T1M5	FKBP15	133.5	8	7	24	No
Coronin-6	CORO6_HUMAN	Q6QEF8	CORO6	52.7	12	4	24	No
Protein FAM185A	F185A_HUMAN	Q8N0U4	FAM185A	42.3	11	3	24	No
Nuclear protein localization protein 4 homolog	NPL4_HUMAN	Q8TAT6	NPLOC4	68.1	8	4	24	No
Serine/threonine-protein kinase Nek9	NEK9_HUMAN	Q8TD19	NEK9	107.1	10	6	24	No
SH3 domain-containing kinase-binding protein 1	SH3K1_HUMAN	Q96B97	SH3KBP1	73.1	6	3	24	No
Sorting nexin-27	SNX27_HUMAN	Q96L92	SNX27	61.2	5	2	24	No
Copine-1	CPNE1_HUMAN	Q99829	CPNE1	59	7	3	24	No
CD209 antigen	CD209_HUMAN	Q9NNX6	CD209	45.7	22	4	24	No
Complex I assembly factor TIMMDC1, mitochondrial	TIDC1_HUMAN	Q9NPL8	TIMMDC1	32.2	20	6	24	No
L-aminoadipate-semialdehyde dehydrogenase-phosphopantetheinyl transferase	ADPPT_HUMAN	Q9NRN7	AASDHPPT	35.8	8	2	24	No
DnaJ homolog subfamily C member 11	DJC11_HUMAN	Q9NVH1	DNAJC11	63.2	9	5	24	No
Cytosolic iron-sulfur assembly component 2B	CIA2B_HUMAN	Q9Y3D0	CIAO2B	17.7	39	3	24	No
Deoxynucleoside triphosphate triphosphohydrolase SAMHD1	SAMH1_HUMAN	Q9Y3Z3	SAMHD1	72.2	10	6	24	No
Galectin-8	LEG8_HUMAN	O00214	LGALS8	35.8	15	5	23	No
Copper chaperone for superoxide dismutase	CCS_HUMAN	O14618	CCS	29	22	4	23	No
Golgi SNAP receptor complex member 2	GOSR2_HUMAN	O14653	GOSR2	24.8	21	3	23	No
Vinexin	VINEX_HUMAN	O60504	SORBS3	75.3	11	5	23	No
Keratin, type I cuticular Ha8	KRT38_HUMAN	O76015	KRT38	50.4	12	2	23	No
Protein transport protein Sec24D	SC24D_HUMAN	O94855	SEC24D	112.9	2	2	23	No
Coagulation factor XII	FA12_HUMAN	P00748	F12	67.7	11	5	23	No
Matrix metalloproteinase-9	MMP9_HUMAN	P14780	MMP9	78.4	7	3	23	No
Poliovirus receptor	PVR_HUMAN	P15151	PVR	45.3	5	2	23	No
Replication protein A 70 kDa DNA-binding subunit	RFA1_HUMAN	P27694	RPA1	68.1	6	3	23	No
Cleavage stimulation factor subunit 2	CSTF2_HUMAN	P33240	CSTF2	60.9	3	2	23	No
60S ribosomal protein L13a	RL13A_HUMAN	P40429	RPL13A	23.6	15	3	23	No
KH domain-containing, RNA-binding, signal transduction-associated protein 1	KHDR1_HUMAN	Q07666	KHDRBS1	48.2	6	2	23	No
A-kinase anchor protein 13	AKP13_HUMAN	Q12802	AKAP13	307.4	2	5	23	No

Mitogen-activated protein kinase 14	MK14_HUMAN	Q16539	MAPK14	41.3	32	6	23	No
Prolyl 3-hydroxylase 1	P3H1_HUMAN	Q32P28	P3H1	83.3	9	6	23	No
von Willebrand factor A domain-containing protein 1	VWA1_HUMAN	Q6PCB0	VWA1	46.8	16	4	23	No
Protein BRICK1	BRK1_HUMAN	Q8WUW1	BRK1	8.7	47	4	23	No
GTPase IMAP family member 1	GIMA1_HUMAN	Q8WWP7	GIMAP1	34.3	18	4	23	No
Probable asparagine--tRNA ligase, mitochondrial	SYNM_HUMAN	Q96I59	NARS2	54.1	4	2	23	No
Caspase recruitment domain-containing protein 19	CAR19_HUMAN	Q96LW7	CARD19	25.6	14	3	23	No
Chloride channel CLIC-like protein 1	CLCC1_HUMAN	Q96S66	CLCC1	62	9	2	23	No
Protein C10	C10_HUMAN	Q99622	C12orf57	13.2	29	3	23	No
Vacuolar protein sorting-associated protein VTA1 homolog	VTA1_HUMAN	Q9NP79	VTA1	33.9	10	2	23	No
N-lysine methyltransferase SMYD2	SMYD2_HUMAN	Q9NRG4	SMYD2	49.7	8	3	23	No
Pre-mRNA-processing factor 19	PRP19_HUMAN	Q9UMS4	PRPF19	55.1	21	7	23	No
Protocadherin-7	PCDH7_HUMAN	O60245	PCDH7	116	3	2	22	No
Tetraspanin-9	TSN9_HUMAN	O75954	TSPAN9	26.8	9	2	22	No
Receptor-type tyrosine-protein phosphatase mu	PTPRM_HUMAN	P28827	PTPRM	163.6	7	6	22	No
Signal recognition particle 14 kDa protein	SRP14_HUMAN	P37108	SRP14	14.6	34	5	22	No
Methionine aminopeptidase 2	MAP2_HUMAN	P50579	METAP2	52.9	10	3	22	No
60S ribosomal protein L27	RL27_HUMAN	P61353	RPL27	15.8	21	3	22	No
60S ribosomal protein L23	RL23_HUMAN	P62829	RPL23	14.9	40	4	22	No
Immunoglobulin-binding protein 1	IGBP1_HUMAN	P78318	IGBP1	39.2	11	2	22	No
Cytochrome c oxidase assembly protein COX19	COX19_HUMAN	Q49B96	COX19	10.4	22	3	22	No
Electron transfer flavoprotein regulatory factor 1	ETFR1_HUMAN	Q6IPR1	ETFRF1	10.9	40	4	22	No
Butyrophilin-like protein 9	BTNL9_HUMAN	Q6UXG8	BTNL9	59.7	4	2	22	No
Choline transporter-like protein 2	CTL2_HUMAN	Q8IWA5	SLC44A2	80.1	4	3	22	No
N-acylneuraminate cytidylyltransferase	NEUA_HUMAN	Q8NFW8	CMAS	48.3	7	3	22	No
45 kDa calcium-binding protein	CAB45_HUMAN	Q9BRK5	SDF4	41.8	6	2	22	No
Uncharacterized protein C1orf198	CA198_HUMAN	Q9H425	C1orf198	36.3	9	2	22	No
Gephyrin [Includes: Molybdopterin adenylyltransferase	GEPH_HUMAN	Q9NQX3	GPHN	79.7	4	2	22	No
Apoptotic chromatin condensation inducer in the nucleus	ACINU_HUMAN	Q9UKV3	ACIN1	151.8	8	9	22	No
V-type proton ATPase subunit D	VATD_HUMAN	Q9Y5K8	ATP6V1D	28.2	14	3	22	No
Selenoprotein F	SEP15_HUMAN	O60613	SELENOF	18.1	23	4	21	No
Synaptosomal-associated protein 23	SNP23_HUMAN	O00161	SNAP23	23.3	13	2	21	No
Vacuolar protein sorting-associated protein 26A	VP26A_HUMAN	O75436	VPS26A	38.1	8	2	21	No
Tyrosine-protein kinase Yes	YES_HUMAN	P07947	YES1	60.8	6	2	21	No
Lysosome-associated membrane glycoprotein 2	LAMP2_HUMAN	P13473	LAMP2	44.9	6	3	21	No
Natriuretic peptides B	ANFB_HUMAN	P16860	NPPB	14.7	22	3	21	No

Peptidyl-prolyl cis-trans isomerase NIMA-interacting 1	PIN1_HUMAN	Q13526	PIN1	18.2	34	3	21	No
Nicotinate-nucleotide pyrophosphorylase [carboxylating]	NADC_HUMAN	Q15274	QPRT	30.8	10	3	21	No
Lysocardiolipin acyltransferase 1	LCLT1_HUMAN	Q6UWP7	LCLAT1	48.9	8	4	21	No
5'-3' exonuclease PLD3	PLD3_HUMAN	Q8IV08	PLD3	54.7	11	4	21	No
FUN14 domain-containing protein 1	FUND1_HUMAN	Q8IVP5	FUNDC1	17.2	23	2	21	No
ADP-ribosylation factor GTPase-activating protein 2	ARFG2_HUMAN	Q8N6H7	ARFGAP2	56.7	7	3	21	No
Torsin-1A-interacting protein 2	TOIP2_HUMAN	Q8NFQ8	TOR1AIP2	51.2	12	2	21	No
Growth arrest and DNA damage-inducible proteins-interacting protein 1	G45IP_HUMAN	Q8TAE8	GADD45GIP1	25.4	22	5	21	No
TATA-binding protein-associated factor 2N	RBP56_HUMAN	Q92804	TAF15	61.8	7	2	21	No
RNA-binding protein Musashi homolog 2	MSI2H_HUMAN	Q96DH6	MSI2	35.2	23	6	21	No
Peroxisomal acyl-coenzyme A oxidase 2	ACOX2_HUMAN	Q99424	ACOX2	76.8	11	5	21	No
Coiled-coil-helix-coiled-coil-helix domain-containing protein 5	CHCH5_HUMAN	Q9BSY4	CHCHD5	12.4	25	2	21	No
Protein PBDC1	PBDC1_HUMAN	Q9BVG4	PBDC1	26	21	4	21	No
Chitinase domain-containing protein 1	CHID1_HUMAN	Q9BWS9	CHID1	44.9	9	3	21	No
Netrin-4	NET4_HUMAN	Q9HB63	NTN4	70	7	3	21	No
4'-phosphopantetheine phosphatase	PANK4_HUMAN	Q9NVE7	PANK4	85.9	3	2	21	No
Coatomer subunit zeta-2	COPZ2_HUMAN	Q9P299	COPZ2	23.5	14	3	21	No
Angiopoietin-related protein 2	ANGL2_HUMAN	Q9UKU9	ANGPTL2	57.1	10	4	21	No
Serine/arginine repetitive matrix protein 2	SRRM2_HUMAN	Q9UQ35	SRRM2	299.4	1	3	21	No
Charged multivesicular body protein 2b	CHM2B_HUMAN	Q9UQN3	CHMP2B	23.9	9	2	21	No
Protein SGT1 homolog	SGT1_HUMAN	Q9Y2Z0	SUGT1	41	16	4	21	No
Sorting and assembly machinery component 50 homolog	SAM50_HUMAN	Q9Y512	SAMM50	51.9	16	5	21	No
Plexin-B1	PLXB1_HUMAN	O43157	PLXNB1	232.2	3	5	20	No
Cleavage and polyadenylation specificity factor subunit 5	CPSF5_HUMAN	O43809	NUDT21	26.2	13	3	20	No
TBC1 domain family member 4	TBCD4_HUMAN	O60343	TBC1D4	146.5	3	3	20	No
Eukaryotic translation initiation factor 5B	IF2P_HUMAN	O60841	EIF5B	138.7	3	2	20	No
Unconventional myosin-Id	MYO1D_HUMAN	O94832	MYO1D	116.1	4	3	20	No
Apolipoprotein M	APOM_HUMAN	O95445	APOM	21.2	10	2	20	No
Heparin cofactor 2	HEP2_HUMAN	P05546	SERPIND1	57	8	4	20	No
60S ribosomal protein L9	RL9_HUMAN	P32969	RPL9	21.9	23	4	20	No
Chromobox protein homolog 5	CBX5_HUMAN	P45973	CBX5	22.2	17	3	20	No
Ubiquitin-conjugating enzyme E2 K	UBE2K_HUMAN	P61086	UBE2K	22.4	29	5	20	No
U6 snRNA-associated Sm-like protein LSm6	LSM6_HUMAN	P62312	LSM6	9.1	25	2	20	No
60S ribosomal protein L32	RL32_HUMAN	P62910	RPL32	15.9	33	4	20	No
Guanine nucleotide-binding protein G	GBG5_HUMAN	P63218	GNG5	7.3	37	4	20	No

Aldo-keto reductase family 1 member C1	AK1C1_HUMAN	Q04828	AKR1C1	36.8	19	2	20	No
cGMP-dependent protein kinase 1	KGP1_HUMAN	Q13976	PRKG1	76.3	7	4	20	No
Latent-transforming growth factor beta-binding protein 1	LTBP1_HUMAN	Q14766	LTBP1	186.7	3	4	20	No
BRISC complex subunit Abraxas 2	ABRX2_HUMAN	Q15018	ABRAXAS2	46.9	21	6	20	No
Coiled-coil domain-containing protein 93	CCD93_HUMAN	Q567U6	CCDC93	73.2	9	4	20	No
Rho GTPase-activating protein 17	RHG17_HUMAN	Q68EM7	ARHGAP17	95.4	5	4	20	No
Anamorsin	CPIN1_HUMAN	Q6FI81	CIAPIN1	33.6	13	3	20	No
Hepatoma-derived growth factor-related protein 2	HDGR2_HUMAN	Q7Z4V5	HDGFL2	74.3	4	2	20	No
Coiled-coil domain-containing protein 50	CCD50_HUMAN	Q8IVM0	CCDC50	35.8	6	2	20	No
5'	NT5C_HUMAN	Q8TCD5	NT5C	23.4	40	6	20	No
Signal transducing adapter molecule 1	STAM1_HUMAN	Q92783	STAM	59.1	10	4	20	No
Rab-interacting lysosomal protein	RILP_HUMAN	Q96NA2	RILP	44.2	11	5	20	No
GPI transamidase component PIG-S	PIGS_HUMAN	Q96S52	PIGS	61.6	11	4	20	No
Protein cereblon	CRBN_HUMAN	Q96SW2	CRBN	50.5	4	2	20	No
Dickkopf-related protein 3	DKK3_HUMAN	Q9UBP4	DKK3	38.4	20	5	20	No
Vacuolar protein sorting-associated protein 4A	VPS4A_HUMAN	Q9UN37	VPS4A	48.9	8	3	20	No
AP-3 complex subunit beta-1	AP3B1_HUMAN	O00203	AP3B1	121.2	2	2	19	No
Thy-1 membrane glycoprotein	THY1_HUMAN	P04216	THY1	17.9	25	4	19	No
Argininosuccinate lyase	ARLY_HUMAN	P04424	ASL	51.6	5	2	19	No
Eukaryotic translation initiation factor 2 subunit 2	IF2B_HUMAN	P20042	EIF2S2	38.4	13	4	19	No
60S ribosomal protein L35	RL35_HUMAN	P42766	RPL35	14.5	17	2	19	No
Activated RNA polymerase II transcriptional coactivator p15	TCP4_HUMAN	P53999	SUB1	14.4	46	6	19	No
Eukaryotic translation initiation factor 6	IF6_HUMAN	P56537	EIF6	26.6	25	4	19	No
Disintegrin and metalloproteinase domain-containing protein 17	ADA17_HUMAN	P78536	ADAM17	93	3	3	19	No
SNW domain-containing protein 1	SNW1_HUMAN	Q13573	SNW1	61.5	7	3	19	No
Serine/threonine-protein phosphatase 2A 56 kDa regulatory subunit epsilon isoform	2A5E_HUMAN	Q16537	PPP2R5E	54.7	11	4	19	No
Transmembrane protein 119	TM119_HUMAN	Q4V9L6	TMEM119	29.2	13	2	19	No
Ras-related GTP-binding protein B	RRAGB_HUMAN	Q5VZM2	RRAGB	43.2	7	3	19	No
Cytoplasmic FMR1-interacting protein 1	CYFP1_HUMAN	Q7L576	CYFIP1	145.1	5	3	19	No
Protein LZIC	LZIC_HUMAN	Q8WZA0	LZIC	21.5	18	4	19	No
Melanoma inhibitory activity protein 2	MIA2_HUMAN	Q96PC5	MIA2	159.7	3	4	19	No
N-terminal Xaa-Pro-Lys N-methyltransferase 1	NTM1A_HUMAN	Q9BV86	NTMT1	25.4	13	3	19	No
SUMO-activating enzyme subunit 2	SAE2_HUMAN	Q9UBT2	UBA2	71.2	8	5	19	No
Melanoma-associated antigen D2	MAGD2_HUMAN	Q9UNF1	MAGED2	64.9	17	6	19	No
28S ribosomal protein S16, mitochondrial	RT16_HUMAN	Q9Y3D3	MRPS16	15.3	19	2	19	No

60S ribosomal protein L36	RL36_HUMAN	Q9Y3U8	RPL36	12.2	29	3	19	No
Myc box-dependent-interacting protein 1	BIN1_HUMAN	O00499	BIN1	64.7	5	2	18	No
S-adenosylhomocysteine hydrolase-like protein 1	SAHH2_HUMAN	O43865	AHCYL1	58.9	11	6	18	No
Apolipoprotein B-100	APOB_HUMAN	P04114	APOB	515.3	2	9	18	No
Signal recognition particle receptor subunit alpha	SRPRA_HUMAN	P08240	SRPRA	69.8	6	3	18	No
Integrin alpha-IIb	ITA2B_HUMAN	P08514	ITGA2B	113.3	13	8	18	No
Ubiquitin-like protein 4A	UBL4A_HUMAN	P11441	UBL4A	17.8	34	5	18	No
Oxygen-dependent coproporphyrinogen-III oxidase, mitochondrial	HEM6_HUMAN	P36551	CPOX	50.1	17	5	18	No
60S ribosomal protein L21	RL21_HUMAN	P46778	RPL21	18.6	9	2	18	No
Delta-1-pyrroline-5-carboxylate synthase	P5CS_HUMAN	P54886	ALDH18A1	87.2	4	3	18	No
Serine/arginine-rich splicing factor 3	SRSF3_HUMAN	P84103	SRSF3	19.3	18	2	18	No
Kynurenine--oxoglutarate transaminase 1	KAT1_HUMAN	Q16773	KYAT1	47.8	9	2	18	No
Proteasome adapter and scaffold protein ECM29	ECM29_HUMAN	Q5VYK3	ECPAS	204.2	4	5	18	No
ADAMTS-like protein 5	ATL5_HUMAN	Q6ZMM2	ADAMTSL5	53.2	10	2	18	No
Podocan	PODN_HUMAN	Q7Z5L7	PODN	68.9	10	5	18	No
NADH dehydrogenase [ubiquinone] 1 alpha subcomplex subunit 11	NDUAB_HUMAN	Q86Y39	NDUFA11	14.8	24	2	18	No
Metalloendopeptidase OMA1, mitochondrial	OMA1_HUMAN	Q96E52	OMA1	60.1	14	5	18	No
Coenzyme Q-binding protein COQ10 homolog A, mitochondrial	CQ10A_HUMAN	Q96MF6	COQ10A	27.7	8	2	18	No
N-acetylmuramoyl-L-alanine amidase	PGRP2_HUMAN	Q96PD5	PGLYRP2	62.2	14	5	18	No
TRIO and F-actin-binding protein	TARA_HUMAN	Q9H2D6	TRIOBP	261.2	4	7	18	No
von Willebrand factor A domain-containing protein 8	VWA8_HUMAN	A3KMH1	VWA8	214.7	2	4	17	No
NADH dehydrogenase [ubiquinone] 1 beta subcomplex subunit 2, mitochondrial	NDUB2_HUMAN	O95178	NDUFB2	12.1	9	2	17	No
CUGBP Elav-like family member 2	CELF2_HUMAN	O95319	CELF2	54.3	6	2	17	No
Collagen alpha-2	CO4A2_HUMAN	P08572	COL4A2	167.4	3	4	17	No
Caspase-14	CASPE_HUMAN	P31944	CASP14	27.7	15	4	17	No
Replication protein A 14 kDa subunit	RFA3_HUMAN	P35244	RPA3	13.6	36	3	17	No
Lysosomal Pro-X carboxypeptidase	PCP_HUMAN	P42785	PRCP	55.8	4	2	17	No
Cathelicidin antimicrobial peptide	CAMP_HUMAN	P49913	CAMP	19.3	17	3	17	No
40S ribosomal protein S24	RS24_HUMAN	P62847	RPS24	15.4	14	2	17	No
Bcl-2-like protein 1	B2CL1_HUMAN	Q07817	BCL2L1	26	8	2	17	No
26S proteasome non-ATPase regulatory subunit 5	PSMD5_HUMAN	Q16401	PSMD5	56.2	8	3	17	No
Tumor protein D53	TPD53_HUMAN	Q16890	TPD52L1	22.4	22	4	17	No
Acylglycerol kinase, mitochondrial	AGK_HUMAN	Q53H12	AGK	47.1	11	4	17	No
Methylmalonic aciduria type A protein, mitochondrial	MMAA_HUMAN	Q8IVH4	MMAA	46.5	6	2	17	No
Ras-related protein Rab-24	RAB24_HUMAN	Q969Q5	RAB24	23.1	10	2	17	No

Phosphotriesterase-related protein	PTER_HUMAN	Q96BW5	PTER	39	6	2	17	No
Septin-5	SEPT5_HUMAN	Q99719	SEPTIN5	42.8	26	8	17	No
Latexin	LXN_HUMAN	Q9BS40	LXN	25.7	12	2	17	No
Threonine--tRNA ligase, mitochondrial	SYTM_HUMAN	Q9BW92	TARS2	81	3	2	17	No
Serrate RNA effector molecule homolog	SRRT_HUMAN	Q9BXP5	SRRT	100.6	5	3	17	No
Tubulointerstitial nephritis antigen-like	TINAL_HUMAN	Q9GZM7	TINAGL1	52.4	12	4	17	No
5'-3' exoribonuclease 2	XRN2_HUMAN	Q9H0D6	XRN2	108.5	4	3	17	No
Ester hydrolase C11orf54	CK054_HUMAN	Q9H0W9	C11orf54	35.1	21	4	17	No
Cell cycle control protein 50A	CC50A_HUMAN	Q9NV96	TMEM30A	40.7	6	2	17	No
Mitochondrial transcription rescue factor 1	MRES1_HUMAN	Q9P0P8	MTRES1	27.9	13	2	17	No
Ribosome maturation protein SBDS	SBDS_HUMAN	Q9Y3A5	SBDS	28.7	11	3	17	No
Ubiquitin carboxyl-terminal hydrolase 15	UBP15_HUMAN	Q9Y4E8	USP15	112.3	9	6	17	No
WAS protein family homolog 2	WASH2_HUMAN	Q6VEQ5	WASH2P	50.3	13	4	16	No
Exocyst complex component 5	EXOC5_HUMAN	O00471	EXOC5	81.8	3	2	16	No
Dihydropyrimidinase-related protein 4	DPYL4_HUMAN	O14531	DPYSL4	61.8	8	3	16	No
Eukaryotic translation initiation factor 1A, Y-chromosomal	IF1AY_HUMAN	O14602	EIF1AY	16.4	25	3	16	No
Ras-related protein M-Ras	RASM_HUMAN	O14807	MRAS	23.8	13	3	16	No
Secretory carrier-associated membrane protein 2	SCAM2_HUMAN	O15127	SCAMP2	36.6	16	4	16	No
HLA class II histocompatibility antigen gamma chain	HG2A_HUMAN	P04233	CD74	33.5	11	4	16	No
Collagen alpha-2	CO1A2_HUMAN	P08123	COL1A2	129.2	4	4	16	No
72 kDa type IV collagenase	MMP2_HUMAN	P08253	MMP2	73.8	7	4	16	No
Alpha-N-acetylgalactosaminidase	NAGAB_HUMAN	P17050	NAGA	46.5	16	5	16	No
E3 ubiquitin-protein ligase TRIM21	RO52_HUMAN	P19474	TRIM21	54.1	4	2	16	No
Glutamine--tRNA ligase	SYQ_HUMAN	P47897	QARS1	87.7	10	5	16	No
DNA-directed RNA polymerases I, II, and III subunit RPABC3	RPAB3_HUMAN	P52434	POLR2H	17.1	21	2	16	No
C-terminal-binding protein 1	CTBP1_HUMAN	Q13363	CTBP1	47.5	9	4	16	No
Hyaluronan-binding protein 2	HABP2_HUMAN	Q14520	HABP2	62.6	6	3	16	No
Protein disulfide-isomerase A5	PDIA5_HUMAN	Q14554	PDIA5	59.6	9	3	16	No
Malonate--CoA ligase ACSF3, mitochondrial	ACSF3_HUMAN	Q4G176	ACSF3	64.1	4	2	16	No
Acyl-coenzyme A thioesterase THEM4	THEM4_HUMAN	Q5T1C6	THEM4	27.1	18	4	16	No
GRB10-interacting GYF protein 2	GGYF2_HUMAN	Q6Y7W6	GIGYF2	150	2	2	16	No
Charged multivesicular body protein 1b	CHM1B_HUMAN	Q7LBR1	CHMP1B	22.1	8	2	16	No
COX assembly mitochondrial protein homolog	COXM1_HUMAN	Q7Z7K0	CMC1	12.5	19	2	16	No
Reticulophagy regulator 3	RETR3_HUMAN	Q86VR2	RETREG3	51.4	4	2	16	No
DnaJ homolog subfamily C member 10	DJC10_HUMAN	Q8IXB1	DNAJC10	91	6	4	16	No

Pyridine nucleotide-disulfide oxidoreductase domain-containing protein 2	PYRD2_HUMAN	Q8N2H3	PYROXD2	63	6	3	16	No
Serine/threonine-protein phosphatase PGAM5, mitochondrial	PGAM5_HUMAN	Q96HS1	PGAM5	32	14	4	16	No
Transmembrane protein 126A	T126A_HUMAN	Q9H061	TMEM126A	21.5	14	3	16	No
Translation initiation factor eIF-2B subunit gamma	EI2BG_HUMAN	Q9NR50	EIF2B3	50.2	8	2	16	No
Matrix-remodeling-associated protein 5	MXRA5_HUMAN	Q9NR99	MXRA5	312	2	5	16	No
LIM and cysteine-rich domains protein 1	LMCD1_HUMAN	Q9NZU5	LMCD1	40.8	12	4	16	No
Plexin-A1	PLXA1_HUMAN	Q9UIW2	PLXNA1	210.9	2	2	16	No
Exocyst complex component 7	EXOC7_HUMAN	Q9UPT5	EXOC7	83.3	11	6	16	No
Proline-rich basic protein 1	PROB1_HUMAN	E7EW31	PROB1	106.9	6	5	15	No
DnaJ homolog subfamily C member 8	DNJC8_HUMAN	O75937	DNAJC8	29.8	13	3	15	No
Probable bifunctional dTTP/UTP pyrophosphatase/methyltransferase protein [Includes: dTTP/UTP pyrophosphatase	ASML_HUMAN	O95671	ASMTL	68.8	11	5	15	No
Immunoglobulin J chain	IGJ_HUMAN	P01591	JCHAIN	18.1	19	3	15	No
Tissue alpha-L-fucosidase	FUCO_HUMAN	P04066	FUCA1	53.7	6	3	15	No
Alpha-galactosidase A	AGAL_HUMAN	P06280	GLA	48.7	12	4	15	No
Immunoglobulin kappa variable 4-1	KV401_HUMAN	P06312	IGKV4-1	13.4	20	2	15	No
Thrombospondin-1	TSP1_HUMAN	P07996	THBS1	129.3	9	8	15	No
Glutathione S-transferase theta-1	GSTT1_HUMAN	P30711	GSTT1	27.3	13	3	15	No
Alpha-taxilin	TXLNA_HUMAN	P40222	TXLNA	61.9	6	2	15	No
40S ribosomal protein S27	RS27_HUMAN	P42677	RPS27	9.5	25	2	15	No
Arfaptin-1	ARFP1_HUMAN	P53367	ARFIP1	41.7	13	4	15	No
Protein transport protein Sec61 subunit beta	SC61B_HUMAN	P60468	SEC61B	10	27	2	15	No
40S ribosomal protein S6	RS6_HUMAN	P62753	RPS6	28.7	20	4	15	No
Nucleolysin TIAR	TIAR_HUMAN	Q01085	TIAL1	41.6	14	2	15	No
Desmoglein-1	DSG1_HUMAN	Q02413	DSG1	113.7	8	6	15	No
Probable leucine--tRNA ligase, mitochondrial	SYLM_HUMAN	Q15031	LARS2	101.9	5	4	15	No
PDZ domain-containing protein 11	PDZ11_HUMAN	Q5EBL8	PDZD11	16.1	51	5	15	No
EGF domain-specific O-linked N-acetylglucosamine transferase	EOGT_HUMAN	Q5NDL2	EOGT	62	4	2	15	No
Exosome complex component MTR3	EXOS6_HUMAN	Q5RKV6	EXOSC6	28.2	16	2	15	No
Tumor protein p63-regulated gene 1-like protein	TPRGL_HUMAN	Q5T0D9	TPRG1L	30.2	44	5	15	No
Serine/threonine/tyrosine-interacting-like protein 2	STYL2_HUMAN	Q5VZP5	STYXL2	130.1	7	4	15	No
PHD finger-like domain-containing protein 5A	PHF5A_HUMAN	Q7RTV0	PHF5A	12.4	25	2	15	No
Nucleoporin NUP35	NUP35_HUMAN	Q8NFH5	NUP35	34.8	8	2	15	No
Ataxin-2-like protein	ATX2L_HUMAN	Q8WWM7	ATXN2L	113.3	2	2	15	No

Proteasome inhibitor PI31 subunit	PSMF1_HUMAN	Q92530	PSMF1	29.8	9	2	15	No
Synapse-associated protein 1	SYAP1_HUMAN	Q96A49	SYAP1	39.9	19	4	15	No
Rho guanine nucleotide exchange factor 17	ARHGH_HUMAN	Q96PE2	ARHGEF17	221.5	1	2	15	No
Rab GTPase-binding effector protein 2	RABE2_HUMAN	Q9H5N1	RABEP2	63.5	5	3	15	No
Calmodulin-like protein 5	CALL5_HUMAN	Q9NZT1	CALML5	15.9	69	7	15	No
FERM, ARHGEF and pleckstrin domain-containing protein 1	FARP1_HUMAN	Q9Y4F1	FARP1	118.6	7	5	15	No
U6 snRNA-associated Sm-like protein LSm4	LSM4_HUMAN	Q9Y4Z0	LSM4	15.3	14	2	15	No
AP-1 complex subunit gamma-1	AP1G1_HUMAN	O43747	AP1G1	91.3	2	2	14	No
Protein diaphanous homolog 1	DIAP1_HUMAN	O60610	DIAPH1	141.3	7	8	14	No
SH3 domain-binding glutamic acid-rich-like protein	SH3L1_HUMAN	O75368	SH3BGRL	12.8	30	3	14	No
U5 small nuclear ribonucleoprotein 200 kDa helicase	U520_HUMAN	O75643	SNRNP200	244.4	3	6	14	No
Angiogenin	ANGI_HUMAN	P03950	ANG	16.5	17	2	14	No
Ribonuclease pancreatic	RNAS1_HUMAN	P07998	RNASE1	17.6	28	2	14	No
Probable ATP-dependent RNA helicase DDX6	DDX6_HUMAN	P26196	DDX6	54.4	8	2	14	No
Syntaxin-binding protein 1	STXB1_HUMAN	P61764	STXBP1	67.5	10	5	14	No
Procollagen-lysine,2-oxoglutarate 5-dioxygenase 1	PLOD1_HUMAN	Q02809	PLOD1	83.5	9	5	14	No
Complement factor H-related protein 1	FHR1_HUMAN	Q03591	CFHR1	37.6	11	2	14	No
Anoctamin-6	ANO6_HUMAN	Q4KMQ2	ANO6	106.1	5	4	14	No
Liprin-beta-1	LIPB1_HUMAN	Q86W92	PPFIBP1	114	2	2	14	No
Serine/arginine repetitive matrix protein 1	SRRM1_HUMAN	Q8IYB3	SRRM1	102.3	6	4	14	No
1-acylglycerol-3-phosphate O-acyltransferase ABHD5	ABHD5_HUMAN	Q8WTS1	ABHD5	39.1	14	3	14	No
Unconventional myosin-XVIIIa	MY18A_HUMAN	Q92614	MYO18A	233	1	2	14	No
A-kinase anchor protein 1, mitochondrial	AKAP1_HUMAN	Q92667	AKAP1	97.3	9	6	14	No
Golgin subfamily A member 1	GOGA1_HUMAN	Q92805	GOLGA1	88.1	3	2	14	No
Immunoglobulin superfamily member 8	IGSF8_HUMAN	Q969P0	IGSF8	65	8	3	14	No
Galactose mutarotase	GALM_HUMAN	Q96C23	GALM	37.7	12	3	14	No
Protein Niban 2	NIBA2_HUMAN	Q96TA1	NIBAN2	84.1	3	3	14	No
Acetyl-CoA acetyltransferase, cytosolic	THIC_HUMAN	Q9BWD1	ACAT2	41.3	8	3	14	No
39S ribosomal protein L4, mitochondrial	RM04_HUMAN	Q9BYD3	MRPL4	34.9	19	4	14	No
Myozenin-1	MYOZ1_HUMAN	Q9NP98	MYOZ1	31.7	28	3	14	No
SAFB-like transcription modulator	SLTM_HUMAN	Q9NWH9	SLTM	117.1	3	2	14	No
Voltage-dependent calcium channel subunit alpha-2/delta-2	CA2D2_HUMAN	Q9NY47	CACNA2D2	129.7	1	2	14	No
Translocation protein SEC63 homolog	SEC63_HUMAN	Q9UGP8	SEC63	87.9	2	2	14	No
V-type proton ATPase subunit H	VATH_HUMAN	Q9UI12	ATP6V1H	55.8	6	2	14	No
Sorting nexin-9	SNX9_HUMAN	Q9Y5X1	SNX9	66.6	13	6	14	No
Peptidyl-prolyl cis-trans isomerase FKBP7	FKBP7_HUMAN	Q9Y680	FKBP7	25.8	11	2	13	No

Double-stranded RNA-binding protein Staufen homolog 2	STAU2_HUMAN	Q9NUL3	STAU2	62.6	5	2	13	No
Deoxyribonuclease-2-alpha	DNS2A_HUMAN	O00115	DNASE2	39.6	10	3	13	No
DNA fragmentation factor subunit alpha	DFFA_HUMAN	O00273	DFFA	36.5	9	2	13	No
Sulfhydryl oxidase 1	QSOX1_HUMAN	O00391	QSOX1	82.5	3	2	13	No
Dynactin subunit 6	DCTN6_HUMAN	O00399	DCTN6	20.7	19	3	13	No
2'-deoxynucleoside 5'-phosphate N-hydrolase 1	DNPH1_HUMAN	O43598	DNPH1	19.1	25	3	13	No
Acyl-protein thioesterase 2	LYPA2_HUMAN	O95372	LYPLA2	24.7	7	2	13	No
Double-stranded RNA-binding protein Staufen homolog 1	STAU1_HUMAN	O95793	STAU1	63.1	9	4	13	No
NAD	NQO1_HUMAN	P15559	NQO1	30.8	10	3	13	No
Centrin-2	CETN2_HUMAN	P41208	CETN2	19.7	20	3	13	No
Fragile X mental retardation syndrome-related protein 1	FXR1_HUMAN	P51114	FXR1	69.7	8	3	13	No
Tricarboxylate transport protein, mitochondrial	TXTP_HUMAN	P53007	SLC25A1	34	13	4	13	No
Myotrophin	MTPN_HUMAN	P58546	MTPN	12.9	17	2	13	No
ATP-binding cassette sub-family E member 1	ABCE1_HUMAN	P61221	ABCE1	67.3	4	2	13	No
TSC22 domain family protein 1	T22D1_HUMAN	Q15714	TSC22D1	109.6	5	2	13	No
Intersectin-1	ITSN1_HUMAN	Q15811	ITSN1	195.3	3	4	13	No
Vesicle-associated membrane protein 3	VAMP3_HUMAN	Q15836	VAMP3	11.3	40	2	13	No
Bcl-2 homologous antagonist/killer	BAK_HUMAN	Q16611	BAK1	23.4	21	2	13	No
Protein phosphatase 1 regulatory subunit 3A	PPR3A_HUMAN	Q16821	PPP1R3A	125.7	5	4	13	No
Cullin-associated NEDD8-dissociated protein 1	CAND1_HUMAN	Q86VP6	CAND1	136.3	6	6	13	No
Vacuolar protein sorting-associated protein 37A	VP37A_HUMAN	Q8NEZ2	VPS37A	44.3	12	3	13	No
Histone H1.10	H1X_HUMAN	Q92522	H1-10	22.5	17	3	13	No
Translin-associated protein X	TSNAX_HUMAN	Q99598	TSNAX	33.1	9	2	13	No
Suppressor of IKBKE 1	SIKE1_HUMAN	Q9BRV8	SIKE1	23.7	12	2	13	No
ER membrane protein complex subunit 3	EMC3_HUMAN	Q9P0I2	EMC3	29.9	16	4	13	No
Protein NDRG3	NDRG3_HUMAN	Q9UGV2	NDRG3	41.4	7	2	13	No
Epididymis-specific alpha-mannosidase	MA2B2_HUMAN	Q9Y2E5	MAN2B2	113.9	5	4	13	No
Soluble scavenger receptor cysteine-rich domain-containing protein SSC5D	SRCRL_HUMAN	A1L4H1	SSC5D	165.6	3	3	12	No
Periplakin	PEPL_HUMAN	O60437	PPL	204.6	2	3	12	No
Huntingtin-interacting protein 1-related protein	HIP1R_HUMAN	O75146	HIP1R	119.3	2	2	12	No
Phosphatase and actin regulator 2	PHAR2_HUMAN	O75167	PHACTR2	69.7	4	2	12	No
Glypican-4	GPC4_HUMAN	O75487	GPC4	62.4	14	5	12	No
Reversion-inducing cysteine-rich protein with Kazal motifs	RECK_HUMAN	O95980	RECK	106.4	4	4	12	No
SPARC	SPRC_HUMAN	P09486	SPARC	34.6	16	4	12	No
Integrin alpha-M	ITAM_HUMAN	P11215	ITGAM	127.1	11	10	12	No

Glucose-6-phosphate 1-dehydrogenase	G6PD_HUMAN	P11413	G6PD	59.2	12	4	12	No
DnaJ homolog subfamily B member 1	DNJB1_HUMAN	P25685	DNAJB1	38	12	2	12	No
Protein DEK	DEK_HUMAN	P35659	DEK	42.6	13	5	12	No
Aldo-keto reductase family 1 member C3	AK1C3_HUMAN	P42330	AKR1C3	36.8	16	2	12	No
40S ribosomal protein S11	RS11_HUMAN	P62280	RPS11	18.4	18	3	12	No
CMP-N-acetylneuraminate-beta-galactosamide-alpha-2,3-sialyltransferase 1	SIA4A_HUMAN	Q11201	ST3GAL1	39.1	12	3	12	No
Serine/threonine-protein phosphatase 2A 56 kDa regulatory subunit gamma isoform	2A5G_HUMAN	Q13362	PPP2R5C	61	4	2	12	No
Leucine-rich repeat flightless-interacting protein 1	LRRF1_HUMAN	Q32MZ4	LRRFIP1	89.2	12	7	12	No
Protein LSM12 homolog	LSM12_HUMAN	Q3MHD2	LSM12	21.7	9	2	12	No
Queuosine salvage protein	QSPP_HUMAN	Q5T6V5	C9orf64	39	5	2	12	No
Peptidase inhibitor 16	PI16_HUMAN	Q6UXB8	PI16	49.4	9	3	12	No
Catechol O-methyltransferase domain-containing protein 1	CMTD1_HUMAN	Q86VU5	COMTD1	28.8	20	3	12	No
Iron-sulfur cluster co-chaperone protein HscB	HSC20_HUMAN	Q8IWL3	HSCB	27.4	27	4	12	No
DDB1- and CUL4-associated factor 11	DCA11_HUMAN	Q8TEB1	DCAF11	61.6	5	2	12	No
Protein POF1B	POF1B_HUMAN	Q8WVV4	POF1B	68	4	2	12	No
Rho guanine nucleotide exchange factor 2	ARHG2_HUMAN	Q92974	ARHGEF2	111.5	3	3	12	No
Cullin-5	CUL5_HUMAN	Q93034	CUL5	90.9	7	4	12	No
V-type proton ATPase 116 kDa subunit a1	VPP1_HUMAN	Q93050	ATP6V0A1	96.4	7	5	12	No
Cilia- and flagella-associated protein 57	CFA57_HUMAN	Q96MR6	CFAP57	144.9	1	2	12	No
Ataxin-2	ATX2_HUMAN	Q99700	ATXN2	140.2	4	4	12	No
Programmed cell death protein 10	PDC10_HUMAN	Q9BUL8	PDCD10	24.7	16	2	12	No
Charged multivesicular body protein 4b	CHM4B_HUMAN	Q9H444	CHMP4B	24.9	9	2	12	No
Optic atrophy 3 protein	OPA3_HUMAN	Q9H6K4	OPA3	20	9	2	12	No
Protein-cysteine N-palmitoyltransferase HHAT-like protein	HHATL_HUMAN	Q9HCP6	HHATL	56.7	6	2	12	No
GMP reductase 2	GMPR2_HUMAN	Q9P2T1	GMPR2	37.9	8	2	12	No
F-box only protein 40	FBX40_HUMAN	Q9UH90	FBXO40	79.7	4	2	12	No
Splicing factor 3B subunit 6	SF3B6_HUMAN	Q9Y3B4	SF3B6	14.6	18	2	12	No
Serum paraoxonase/arylesterase 2	PON2_HUMAN	Q15165	PON2	39.4	6	2	11	No
D-3-phosphoglycerate dehydrogenase	SERA_HUMAN	O43175	PHGDH	56.6	7	3	11	No
TIP41-like protein	TIPRL_HUMAN	O75663	TIPRL	31.4	14	3	11	No
CAAX prenyl protease 1 homolog	FACE1_HUMAN	O75844	ZMPSTE24	54.8	5	3	11	No
Complement factor D	CFAD_HUMAN	P00746	CFD	27	35	4	11	No
Ornithine aminotransferase, mitochondrial	OAT_HUMAN	P04181	OAT	48.5	10	3	11	No
Semenogelin-1	SEMG1_HUMAN	P04279	SEMG1	52.1	17	6	11	No

5'-nucleotidase	5NTD_HUMAN	P21589	NT5E	63.3	11	5	11	No
Calmodulin-like protein 3	CALL3_HUMAN	P27482	CALML3	16.9	27	3	11	No
Leiomodin-1	LMOD1_HUMAN	P29536	LMOD1	67	7	4	11	No
Serum amyloid A-4 protein	SAA4_HUMAN	P35542	SAA4	14.7	29	4	11	No
Dual specificity mitogen-activated protein kinase kinase 2	MP2K2_HUMAN	P36507	MAP2K2	44.4	20	3	11	No
Histamine N-methyltransferase	HNMT_HUMAN	P50135	HNMT	33.3	11	3	11	No
Ras-related protein Rab-9A	RAB9A_HUMAN	P51151	RAB9A	22.8	12	2	11	No
Signal recognition particle 54 kDa protein	SRP54_HUMAN	P61011	SRP54	55.7	8	4	11	No
60S ribosomal protein L19	RL19_HUMAN	P84098	RPL19	23.5	13	2	11	No
Vigilin	VIGLN_HUMAN	Q00341	HDLBP	141.4	7	6	11	No
Serine/arginine-rich splicing factor 2	SRSF2_HUMAN	Q01130	SRSF2	25.5	11	2	11	No
Leukocyte surface antigen CD47	CD47_HUMAN	Q08722	CD47	35.2	6	2	11	No
V-type proton ATPase subunit S1	VAS1_HUMAN	Q15904	ATP6AP1	52	6	3	11	No
Keratinocyte proline-rich protein	KPRP_HUMAN	Q5T749	KPRP	64.1	9	4	11	No
5'-nucleotidase domain-containing protein 1	NT5D1_HUMAN	Q5TFE4	NT5DC1	51.8	3	2	11	No
Eukaryotic translation initiation factor 3 subunit M	EIF3M_HUMAN	Q7L2H7	EIF3M	42.5	15	4	11	No
Pleckstrin homology-like domain family B member 2	PHLB2_HUMAN	Q86SQ0	PHLDB2	142.1	2	2	11	No
Stimulator of interferon genes protein	STING_HUMAN	Q86WV6	STING1	42.2	10	2	11	No
NudC domain-containing protein 3	NUDC3_HUMAN	Q8IVD9	NUDCD3	40.8	11	2	11	No
Armadillo repeat-containing protein 10	ARM10_HUMAN	Q8N2F6	ARMC10	37.5	6	2	11	No
E3 ubiquitin-protein ligase TRIM63	TRI63_HUMAN	Q969Q1	TRIM63	40.2	8	2	11	No
Protein dpy-30 homolog	DPY30_HUMAN	Q9C005	DPY30	11.2	27	2	11	No
Plasminogen receptor	PLRKT_HUMAN	Q9HBL7	PLGRKT	17.2	16	2	11	No
ER membrane protein complex subunit 7	EMC7_HUMAN	Q9NPA0	EMC7	26.5	22	3	11	No
39S ribosomal protein L40, mitochondrial	RM40_HUMAN	Q9NQ50	MRPL40	24.5	19	4	11	No
Nck-associated protein 1	NCKP1_HUMAN	Q9Y2A7	NCKAP1	128.7	2	2	11	No
28S ribosomal protein S2, mitochondrial	RT02_HUMAN	Q9Y399	MRPS2	33.2	6	2	11	No
TSC22 domain family protein 4	T22D4_HUMAN	Q9Y3Q8	TSC22D4	41	12	2	11	No
CD2-associated protein	CD2AP_HUMAN	Q9Y5K6	CD2AP	71.4	4	2	11	No
CDP-diacylglycerol--inositol 3-phosphatidyltransferase	CDIPT_HUMAN	O14735	CDIPT	23.5	10	2	10	No
Protein arginine N-methyltransferase 5	ANM5_HUMAN	O14744	PRMT5	72.6	8	4	10	No
Peripheral plasma membrane protein CASK	CSKP_HUMAN	O14936	CASK	105.1	2	2	10	No
TSC22 domain family protein 2	T22D2_HUMAN	O75157	TSC22D2	79.2	3	2	10	No
Glia-derived nexin	GDN_HUMAN	P07093	SERPINE2	44	6	2	10	No
Complement component C8 alpha chain	CO8A_HUMAN	P07357	C8A	65.1	10	4	10	No
Beta-glucuronidase	BGLR_HUMAN	P08236	GUSB	74.7	3	2	10	No

U2 small nuclear ribonucleoprotein A'	RU2A_HUMAN	P09661	SNRPA1	28.4	13	3	10	No
Tyrosine-protein phosphatase non-receptor type 1	PTN1_HUMAN	P18031	PTPN1	49.9	7	3	10	No
Macrophage-capping protein	CAPG_HUMAN	P40121	CAPG	38.5	18	6	10	No
Methyl-CpG-binding protein 2	MECP2_HUMAN	P51608	MECP2	52.4	10	4	10	No
Thimet oligopeptidase	THOP1_HUMAN	P52888	THOP1	78.8	4	2	10	No
Adenosine kinase	ADK_HUMAN	P55263	ADK	40.5	16	4	10	No
Anthrax toxin receptor 2	ANTR2_HUMAN	P58335	ANTXR2	53.6	4	2	10	No
DNA-dependent protein kinase catalytic subunit	PRKDC_HUMAN	P78527	PRKDC	468.8	2	5	10	No
SAP domain-containing ribonucleoprotein	SARNP_HUMAN	P82979	SARNP	23.7	9	2	10	No
Perilipin-5	PLIN5_HUMAN	Q00G26	PLIN5	50.8	15	4	10	No
Focal adhesion kinase 1	FAK1_HUMAN	Q05397	PTK2	119.2	4	3	10	No
Serine/threonine-protein phosphatase 2B catalytic subunit alpha isoform	PP2BA_HUMAN	Q08209	PPP3CA	58.7	6	2	10	No
Golgin subfamily B member 1	GOGB1_HUMAN	Q14789	GOLGB1	375.8	1	2	10	No
Splicing factor 1	SF01_HUMAN	Q15637	SF1	68.3	5	3	10	No
39S ribosomal protein L52, mitochondrial	RM52_HUMAN	Q86TS9	MRPL52	13.7	34	2	10	No
Mitofusin-1	MFN1_HUMAN	Q8IWA4	MFN1	84.1	8	5	10	No
Uncharacterized protein FAM241A	F241A_HUMAN	Q8N8J7	FAM241A	14.6	30	2	10	No
Protein THEM6	THEM6_HUMAN	Q8WUY1	THEM6	23.9	20	2	10	No
DAZ-associated protein 1	DAZP1_HUMAN	Q96EP5	DAZAP1	43.4	8	2	10	No
DnaJ homolog subfamily C member 7	DNJC7_HUMAN	Q99615	DNAJC7	56.4	4	3	10	No
Iron-sulfur cluster assembly 1 homolog, mitochondrial	ISCA1_HUMAN	Q9BUE6	ISCA1	14.2	19	2	10	No
3-hydroxybutyrate dehydrogenase type 2	BDH2_HUMAN	Q9BUT1	BDH2	26.7	10	2	10	No
PITH domain-containing protein 1	PITH1_HUMAN	Q9GZP4	PITHD1	24.2	28	4	10	No
Diphthine methyl ester synthase	DPH5_HUMAN	Q9H2P9	DPH5	31.6	11	2	10	No
V-type immunoglobulin domain-containing suppressor of T-cell activation	VISTA_HUMAN	Q9H7M9	VSIR	33.9	11	2	10	No
Ufm1-specific protease 2	UFSP2_HUMAN	Q9NUQ7	UFSP2	53.2	7	2	10	No
Golgi-resident adenosine 3',5'-bisphosphate 3'-phosphatase	IMPA3_HUMAN	Q9NX62	BPNT2	38.7	10	4	10	No
Rabankyrin-5	ANFY1_HUMAN	Q9P2R3	ANKFY1	128.3	4	3	10	No
Peptide chain release factor 1-like, mitochondrial	RF1ML_HUMAN	Q9UGC7	MTRF1L	43.6	8	2	10	No
AP-3 complex subunit delta-1	AP3D1_HUMAN	O14617	AP3D1	130.1	2	2	9	No
Peroxisomal acyl-coenzyme A oxidase 3	ACOX3_HUMAN	O15254	ACOX3	77.6	9	4	9	No
Synaptobrevin homolog YKT6	YKT6_HUMAN	O15498	YKT6	22.4	9	2	9	No
Protein CREG1	CREG1_HUMAN	O75629	CREG1	24.1	15	2	9	No
C4b-binding protein alpha chain	C4BPA_HUMAN	P04003	C4BPA	67	14	6	9	No

Replication protein A 32 kDa subunit	RFA2_HUMAN	P15927	RPA2	29.2	13	2	9	No
Integrin beta-4	ITB4_HUMAN	P16144	ITGB4	202	1	2	9	No
Cysteine--tRNA ligase, cytoplasmic	SYCC_HUMAN	P49589	CARS1	85.4	8	5	9	No
Ras-related protein Rap-2b	RAP2B_HUMAN	P61225	RAP2B	20.5	36	5	9	No
Small nuclear ribonucleoprotein F	RUXF_HUMAN	P62306	SNRPF	9.7	24	2	9	No
Butyrophilin subfamily 3 member A2	BT3A2_HUMAN	P78410	BTN3A2	36.4	11	3	9	No
Inter-alpha-trypsin inhibitor heavy chain H3	ITIH3_HUMAN	Q06033	ITIH3	99.8	4	2	9	No
Extracellular matrix protein 1	ECM1_HUMAN	Q16610	ECM1	60.6	6	2	9	No
E3 ubiquitin-protein ligase RNF123	RN123_HUMAN	Q5XPI4	RNF123	148.4	4	3	9	No
Inhibitor of nuclear factor kappa-B kinase-interacting protein	IKIP_HUMAN	Q70UQ0	IKBIP	39.3	9	3	9	No
ADP-ribosylation factor GTPase-activating protein 1	ARFG1_HUMAN	Q8N6T3	ARFGAP1	44.6	7	2	9	No
Inactive C-alpha-formylglycine-generating enzyme 2	SUMF2_HUMAN	Q8NBJ7	SUMF2	33.8	14	3	9	No
Selenoprotein M	SELM_HUMAN	Q8WWX9	SELENOM	16.2	21	2	9	No
Serine protease HTRA1	HTRA1_HUMAN	Q92743	HTRA1	51.3	14	6	9	No
c-Myc-binding protein	MYCBP_HUMAN	Q99417	MYCBP	12	29	3	9	No
Regulation of nuclear pre-mRNA domain-containing protein 1B	RPR1B_HUMAN	Q9NQG5	RPRD1B	36.9	10	2	9	No
DnaJ homolog subfamily B member 12	DJB12_HUMAN	Q9NXW2	DNAJB12	41.8	10	3	9	No
FACT complex subunit SPT16	SP16H_HUMAN	Q9Y5B9	SUPT16H	119.8	2	2	9	No
Leucine-rich repeat flightless-interacting protein 2	LRRF2_HUMAN	Q9Y608	LRRFIP2	82.1	10	5	9	No
Junctional adhesion molecule A	JAM1_HUMAN	Q9Y624	F11R	32.6	11	3	9	No
Ribonuclease T2	RNT2_HUMAN	O00584	RNASET2	29.5	16	3	8	No
AT-rich interactive domain-containing protein 1A	ARI1A_HUMAN	O14497	ARID1A	241.9	1	2	8	No
Rho-associated protein kinase 2	ROCK2_HUMAN	O75116	ROCK2	160.8	3	4	8	No
Eukaryotic translation initiation factor 3 subunit J	EIF3J_HUMAN	O75822	EIF3J	29	12	3	8	No
26S proteasome non-ATPase regulatory subunit 10	PSD10_HUMAN	O75832	PSMD10	24.4	22	3	8	No
Complement C1r subcomponent	C1R_HUMAN	P00736	C1R	80.1	5	3	8	No
Myeloperoxidase	PERM_HUMAN	P05164	MPO	83.8	8	4	8	No
Complement C2	CO2_HUMAN	P06681	C2	83.2	4	3	8	No
Low affinity immunoglobulin gamma Fc region receptor III-A	FCG3A_HUMAN	P08637	FCGR3A	29.1	13	3	8	No
Nuclear factor NF-kappa-B p105 subunit	NFKB1_HUMAN	P19838	NFKB1	105.3	5	3	8	No
DNA-directed RNA polymerase II subunit RPB1	RPB1_HUMAN	P24928	POLR2A	217	1	2	8	No
ADP-ribosyl cyclase/cyclic ADP-ribose hydrolase 1	CD38_HUMAN	P28907	CD38	34.3	8	2	8	No
Glutamate--cysteine ligase catalytic subunit	GSH1_HUMAN	P48506	GCLC	72.7	5	2	8	No
Serpin B9	SPB9_HUMAN	P50453	SERPINB9	42.4	6	2	8	No
N-sulphoglucosamine sulphohydrolase	SPHM_HUMAN	P51688	SGSH	56.7	7	3	8	No
Spermine synthase	SPSY_HUMAN	P52788	SMS	41.2	8	3	8	No

Dipeptidyl peptidase 1	CATC_HUMAN	P53634	CTSC	51.8	9	4	8	No
GTP-binding protein RAD	RAD_HUMAN	P55042	RRAD	33.2	10	2	8	No
Phospholipid transfer protein	PLTP_HUMAN	P55058	PLTP	54.7	5	2	8	No
Peptidyl-prolyl cis-trans isomerase FKBP5	FKBP5_HUMAN	Q13451	FKBP5	51.2	5	2	8	No
Filamin A-interacting protein 1-like	FIL1L_HUMAN	Q4L180	FILIP1L	130.3	3	3	8	No
Rab GTPase-activating protein 1-like	RBG1L_HUMAN	Q5R372	RABGAP1L	92.5	5	3	8	No
Valine--tRNA ligase, mitochondrial	SYVM_HUMAN	Q5ST30	VARS2	118.4	2	2	8	No
Metalloreductase STEAP4	STEA4_HUMAN	Q687X5	STEAP4	51.9	6	2	8	No
Transport and Golgi organization protein 2 homolog	TNG2_HUMAN	Q6ICL3	TANGO2	30.9	17	4	8	No
Threonine synthase-like 1	THNS1_HUMAN	Q8IYQ7	THNSL1	83	7	4	8	No
Phosphatidylinositol 5-phosphate 4-kinase type-2 gamma	PI42C_HUMAN	Q8TBX8	PIP4K2C	47.3	5	2	8	No
Trafficking protein particle complex subunit 12	TPC12_HUMAN	Q8WVT3	TRAPPC12	79.3	3	2	8	No
Mitochondrial import inner membrane translocase subunit TIM14	TIM14_HUMAN	Q96DA6	DNAJC19	12.5	39	3	8	No
Methylthioribulose-1-phosphate dehydratase	MTNB_HUMAN	Q96GX9	APIP	27.1	10	2	8	No
Vacuolar-sorting protein SNF8	SNF8_HUMAN	Q96H20	SNF8	28.8	14	3	8	No
Sorting nexin-18	SNX18_HUMAN	Q96RF0	SNX18	68.9	4	2	8	No
Endophilin-A1	SH3G2_HUMAN	Q99962	SH3GL2	39.9	19	4	8	No
NIF3-like protein 1	NIF3L_HUMAN	Q9GZT8	NIF3L1	41.9	7	3	8	No
Probable bifunctional methylenetetrahydrofolate dehydrogenase/cyclohydrolase 2	MTD2L_HUMAN	Q9H903	MTHFD2L	37.3	7	2	8	No
Charged multivesicular body protein 1a	CHM1A_HUMAN	Q9HD42	CHMP1A	21.7	8	2	8	No
Baculoviral IAP repeat-containing protein 6	BIRC6_HUMAN	Q9NR09	BIRC6	529.9	1	2	8	No
Alpha-mannosidase 2C1	MA2C1_HUMAN	Q9NTJ4	MAN2C1	115.8	5	3	8	No
Spliceosome-associated protein CWC15 homolog	CWC15_HUMAN	Q9P013	CWC15	26.6	9	2	8	No
Protein argonaute-2	AGO2_HUMAN	Q9UKV8	AGO2	97.1	5	3	8	No
Phosphoenolpyruvate carboxykinase [GTP], mitochondrial	PCKGM_HUMAN	Q16822	PCK2	70.7	6	4	7	No
Immunoglobulin superfamily containing leucine-rich repeat protein	ISLR_HUMAN	O14498	ISLR	46	7	2	7	No
Disintegrin and metalloproteinase domain-containing protein 10	ADA10_HUMAN	O14672	ADAM10	84.1	6	3	7	No
Apolipoprotein L1	APOL1_HUMAN	O14791	APOL1	43.9	8	3	7	No
Heat shock 70 kDa protein 12A	HS12A_HUMAN	O43301	HSPA12A	74.9	4	3	7	No
Retinal rod rhodopsin-sensitive cGMP 3',5'-cyclic phosphodiesterase subunit delta	PDE6D_HUMAN	O43924	PDE6D	17.4	21	2	7	No
Phosphoacetylglucosamine mutase	AGM1_HUMAN	O95394	PGM3	59.8	6	3	7	No
Prostaglandin G/H synthase 1	PGH1_HUMAN	P23219	PTGS1	68.6	6	2	7	No
Syndecan-4	SDC4_HUMAN	P31431	SDC4	21.6	11	2	7	No

Glia maturation factor beta	GMFB_HUMAN	P60983	GMFB	16.7	14	2	7	No
40S ribosomal protein S26	RS26_HUMAN	P62854	RPS26	13	21	2	7	No
28S ribosomal protein S6, mitochondrial	RT06_HUMAN	P82932	MRPS6	14.2	10	2	7	No
Galectin-10	LEG10_HUMAN	Q05315	CLC	16.4	10	2	7	No
Synaptic functional regulator FMR1	FMR1_HUMAN	Q06787	FMR1	71.1	4	2	7	No
Chromodomain-helicase-DNA-binding protein 4	CHD4_HUMAN	Q14839	CHD4	217.9	1	2	7	No
Coiled-coil domain-containing protein 6	CCDC6_HUMAN	Q16204	CCDC6	53.3	4	2	7	No
Inverted formin-2	INF2_HUMAN	Q27J81	INF2	135.5	2	3	7	No
Prolyl endopeptidase-like	PPCEL_HUMAN	Q4J6C6	PREPL	83.9	5	3	7	No
E3 ubiquitin-protein ligase UBR4	UBR4_HUMAN	Q5T4S7	UBR4	573.5	1	4	7	No
GRAM domain-containing protein 4	GRAM4_HUMAN	Q6IC98	GRAMD4	66.4	3	2	7	No
Oxidation resistance protein 1	OXR1_HUMAN	Q8N573	OXR1	97.9	3	2	7	No
NEDD8-activating enzyme E1 catalytic subunit	UBA3_HUMAN	Q8TBC4	UBA3	51.8	7	3	7	No
PRKC apoptosis WT1 regulator protein	PAWR_HUMAN	Q96IZ0	PAWR	36.5	8	3	7	No
Heat shock 70 kDa protein 12B	HS12B_HUMAN	Q96MM6	HSPA12B	75.6	3	2	7	No
Splicing factor 3B subunit 5	SF3B5_HUMAN	Q9BWJ5	SF3B5	10.1	41	3	7	No
AP-1 complex subunit mu-1	AP1M1_HUMAN	Q9BXS5	AP1M1	48.6	5	2	7	No
UBX domain-containing protein 6	UBXN6_HUMAN	Q9BZV1	UBXN6	49.7	7	2	7	No
Phospholysine phosphohistidine inorganic pyrophosphate phosphatase	LHPP_HUMAN	Q9H008	LHPP	29.1	8	2	7	No
Peptidyl-prolyl cis-trans isomerase-like 3	PPIL3_HUMAN	Q9H2H8	PPIL3	18.1	14	2	7	No
Damage-control phosphatase ARMT1	ARMT1_HUMAN	Q9H993	ARMT1	51.1	8	3	7	No
Calcium-independent phospholipase A2-gamma	PLPL8_HUMAN	Q9NP80	PNPLA8	88.4	3	2	7	No
GTPase IMAP family member 4	GIMA4_HUMAN	Q9NUV9	GIMAP4	37.5	6	2	7	No
BRISC and BRCA1-A complex member 2	BABA2_HUMAN	Q9NXR7	BABAM2	43.5	6	2	7	No
COMM domain-containing protein 3	COMD3_HUMAN	Q9UBI1	COMMD3	22.1	10	2	7	No
BAG family molecular chaperone regulator 5	BAG5_HUMAN	Q9UL15	BAG5	51.2	6	2	7	No
Two pore calcium channel protein 1	TPC1_HUMAN	Q9ULQ1	TPCN1	94.1	3	2	7	No
GDP-fucose protein O-fucosyltransferase 2	OFUT2_HUMAN	Q9Y2G5	POFUT2	49.9	5	2	7	No
Tyrosine--tRNA ligase, mitochondrial	SYYM_HUMAN	Q9Y2Z4	YARS2	53.2	6	2	7	No
Transmembrane emp24 domain-containing protein 5	TMED5_HUMAN	Q9Y3A6	TMED5	26	7	2	7	No
Pericentriolar material 1 protein	PCM1_HUMAN	Q15154	PCM1	228.4	2	3	6	No
Maleylacetoacetate isomerase	MAAI_HUMAN	O43708	GSTZ1	24.2	14	2	6	No
CLIP-associating protein 2	CLAP2_HUMAN	O75122	CLASP2	141	3	2	6	No
E3 ubiquitin-protein ligase ARIH2	ARI2_HUMAN	O95376	ARIH2	57.8	6	2	6	No
Heme oxygenase 1	HMOX1_HUMAN	P09601	HMOX1	32.8	18	5	6	No

Non-secretory ribonuclease	RNAS2_HUMAN	P10153	RNASE2	18.3	12	2	6	No
Membrane cofactor protein	MCP_HUMAN	P15529	CD46	43.7	6	2	6	No
Transcription factor BTF3	BTF3_HUMAN	P20290	BTF3	22.2	13	2	6	No
Interferon-induced GTP-binding protein Mx2	MX2_HUMAN	P20592	MX2	82	4	2	6	No
DNA-	APEX1_HUMAN	P27695	APEX1	35.5	11	3	6	No
Adhesion G protein-coupled receptor E5	AGRE5_HUMAN	P48960	ADGRE5	91.8	6	4	6	No
Double-strand break repair protein MRE11	MRE11_HUMAN	P49959	MRE11	80.5	2	2	6	No
Nuclear pore complex protein Nup98-Nup96	NUP98_HUMAN	P52948	NUP98	197.5	2	3	6	No
Cytosolic Fe-S cluster assembly factor NUBP1	NUBP1_HUMAN	P53384	NUBP1	34.5	13	2	6	No
Calcineurin subunit B type 1	CANB1_HUMAN	P63098	PPP3R1	19.3	21	2	6	No
Golgin subfamily A member 3	GOGA3_HUMAN	Q08378	GOLGA3	167.3	2	2	6	No
Nuclear factor 1 A-type	NFIA_HUMAN	Q12857	NFIA	55.9	5	2	6	No
Protein scribble homolog	SCRIB_HUMAN	Q14160	SCRIB	174.8	1	2	6	No
Inactive hydroxysteroid dehydrogenase-like protein 1	HSDL1_HUMAN	Q3SXM5	HSDL1	37	5	2	6	No
Putative oxidoreductase GLYR1	GLYR1_HUMAN	Q49A26	GLYR1	60.5	5	2	6	No
BolA-like protein 3	BOLA3_HUMAN	Q53S33	BOLA3	12.1	30	2	6	No
DDB1- and CUL4-associated factor 6	DCAF6_HUMAN	Q58WW2	DCAF6	96.2	4	3	6	No
Autophagy-related protein 16-1	A16L1_HUMAN	Q676U5	ATG16L1	68.2	2	2	6	No
Dehydrogenase/reductase SDR family member 11	DHR11_HUMAN	Q6UWP2	DHRS11	28.3	10	2	6	No
G protein-regulated inducer of neurite outgrowth 3	GRIN3_HUMAN	Q6ZVF9	GPRIN3	82.4	4	2	6	No
Mitochondrial Rho GTPase 1	MIRO1_HUMAN	Q8IXI2	RHOT1	70.7	7	3	6	No
1-phosphatidylinositol 4,5-bisphosphate phosphodiesterase delta-3	PLCD3_HUMAN	Q8N3E9	PLCD3	89.2	4	2	6	No
Choline dehydrogenase, mitochondrial	CHDH_HUMAN	Q8NE62	CHDH	65.3	6	3	6	No
Leucine-rich repeat-containing protein 20	LRC20_HUMAN	Q8TCA0	LRRC20	20.5	13	2	6	No
RalBP1-associated Eps domain-containing protein 1	REPS1_HUMAN	Q96D71	REPS1	86.6	5	2	6	No
Ubiquitin carboxyl-terminal hydrolase 47	UBP47_HUMAN	Q96K76	USP47	157.2	2	3	6	No
Alpha-protein kinase 3	ALPK3_HUMAN	Q96L96	ALPK3	201.1	2	3	6	No
39S ribosomal protein L45, mitochondrial	RM45_HUMAN	Q9BRJ2	MRPL45	35.3	10	2	6	No
Nucleoporin p58/p45	NUP58_HUMAN	Q9BVL2	NUP58	60.9	3	2	6	No
BolA-like protein 2	BOLA2_HUMAN	Q9H3K6	BOLA2	10.1	41	2	6	No
Tyrosine-protein phosphatase non-receptor type 23	PTN23_HUMAN	Q9H3S7	PTPN23	178.9	2	2	6	No
Fibronectin type III and SPRY domain-containing protein 2	FSD2_HUMAN	A1L4K1	FSD2	85.3	3	2	5	No
Syntaxin-16	STX16_HUMAN	O14662	STX16	37	6	2	5	No
Secretory carrier-associated membrane protein 1	SCAM1_HUMAN	O15126	SCAMP1	37.9	17	3	5	No

Interferon-inducible double-stranded RNA-dependent protein kinase activator A	PRKRA_HUMAN	O75569	PRKRA	34.4	6	2	5	No
Integrin beta-3	ITB3_HUMAN	P05106	ITGB3	87	8	4	5	No
Eosinophil peroxidase	PERE_HUMAN	P11678	EPX	81	7	4	5	No
Protein S100-A2	S10A2_HUMAN	P29034	S100A2	11.1	28	3	5	No
SHC-transforming protein 1	SHC1_HUMAN	P29353	SHC1	62.8	3	2	5	No
Phosphorylase b kinase regulatory subunit alpha, skeletal muscle isoform	KPB1_HUMAN	P46020	PHKA1	137.2	4	2	5	No
Dolichyl-diphosphooligosaccharide--protein glycosyltransferase subunit STT3A	STT3A_HUMAN	P46977	STT3A	80.5	2	2	5	No
Fatty acid synthase	FAS_HUMAN	P49327	FASN	273.3	2	4	5	No
Tyrosine--tRNA ligase, cytoplasmic	SYYC_HUMAN	P54577	YARS1	59.1	10	4	5	No
Eukaryotic peptide chain release factor subunit 1	ERF1_HUMAN	P62495	ETF1	49	6	2	5	No
40S ribosomal protein S15	RS15_HUMAN	P62841	RPS15	17	9	2	5	No
Polypeptide N-acetylgalactosaminyltransferase 2	GALT2_HUMAN	Q10471	GALNT2	64.7	3	2	5	No
NEDD8-activating enzyme E1 regulatory subunit	ULA1_HUMAN	Q13564	NAE1	60.2	5	2	5	No
[Pyruvate dehydrogenase	PDK3_HUMAN	Q15120	PDK3	46.9	9	3	5	No
RILP-like protein 1	RIPL1_HUMAN	Q5EBL4	RILPL1	47.1	8	3	5	No
Collectin-12	COL12_HUMAN	Q5KU26	COLEC12	81.5	3	2	5	No
Putative DENN domain-containing protein 10 B	DE10B_HUMAN	Q6NSW5	DENND10P1	40.5	10	2	5	No
Long-chain fatty acid transport protein 4	S27A4_HUMAN	Q6P1M0	SLC27A4	72	6	2	5	No
Acyl-CoA dehydrogenase family member 11	ACD11_HUMAN	Q709F0	ACAD11	87.2	4	3	5	No
Armadillo repeat-containing X-linked protein 2	ARMX2_HUMAN	Q7L311	ARMCX2	65.6	8	2	5	No
Abl interactor 1	ABI1_HUMAN	Q8IZP0	ABI1	55	6	2	5	No
CD99 antigen-like protein 2	C99L2_HUMAN	Q8TCZ2	CD99L2	28	18	2	5	No
Nuclear pore complex protein Nup133	NU133_HUMAN	Q8WUM0	NUP133	128.9	3	2	5	No
Ubiquitin carboxyl-terminal hydrolase 7	UBP7_HUMAN	Q93009	USP7	128.2	3	3	5	No
39S ribosomal protein L24, mitochondrial	RM24_HUMAN	Q96A35	MRPL24	24.9	10	2	5	No
39S ribosomal protein L48, mitochondrial	RM48_HUMAN	Q96GC5	MRPL48	23.9	11	2	5	No
Coiled-coil domain-containing protein 115	CC115_HUMAN	Q96NT0	CCDC115	19.7	15	2	5	No
Endophilin-A2	SH3G1_HUMAN	Q99961	SH3GL1	41.5	8	2	5	No
Methylosome protein 50	MEP50_HUMAN	Q9BQA1	WDR77	36.7	13	3	5	No
tRNA	TR61B_HUMAN	Q9BVS5	TRMT61B	52.9	10	3	5	No
Complement factor H-related protein 5	FHR5_HUMAN	Q9BXR6	CFHR5	64.4	13	4	5	No
Ubiquitin-like modifier-activating enzyme 5	UBA5_HUMAN	Q9GZZ9	UBA5	44.8	9	2	5	No
Pleckstrin homology domain-containing family A member 5	PKHA5_HUMAN	Q9HAU0	PLEKHA5	127.4	3	2	5	No

Succinate dehydrogenase assembly factor 3, mitochondrial	SDHF3_HUMAN	Q9NRP4	SDHAF3	14.6	37	4	5	No
Bcl-2-associated transcription factor 1	BCLF1_HUMAN	Q9NYF8	BCLAF1	106.1	4	3	5	No
E3 ubiquitin-protein ligase KCMF1	KCMF1_HUMAN	Q9P0J7	KCMF1	41.9	14	3	5	No
Tryptophan--tRNA ligase, mitochondrial	SYWM_HUMAN	Q9UGM6	WARS2	40.1	6	2	5	No
Short coiled-coil protein	SCOC_HUMAN	Q9UIL1	SCOC	18	18	2	5	No
C-type lectin domain family 11 member A	CLC11_HUMAN	Q9Y240	CLEC11A	35.7	7	2	5	No
Collagen alpha-6	CO6A6_HUMAN	A6NMZ7	COL6A6	247	1	2	4	No
Alpha-2-macroglobulin-like protein 1	A2ML1_HUMAN	A8K2U0	A2ML1	161	5	4	4	No
MIF4G domain-containing protein	MI4GD_HUMAN	A9UHW6	MIF4GD	25.4	11	2	4	No
Protein SCO2 homolog, mitochondrial	SCO2_HUMAN	O43819	SCO2	29.8	8	2	4	No
UDP-glucose 6-dehydrogenase	UGDH_HUMAN	O60701	UGDH	55	4	2	4	No
2-amino-3-ketobutyrate coenzyme A ligase, mitochondrial	KBL_HUMAN	O75600	GCAT	45.3	13	3	4	No
Ly6/PLAUR domain-containing protein 3	LYPD3_HUMAN	O95274	LYPD3	35.9	5	2	4	No
Activator of 90 kDa heat shock protein ATPase homolog 1	AHSA1_HUMAN	O95433	AHSA1	38.3	11	3	4	No
HLA class II histocompatibility antigen, DP beta 1 chain	DPB1_HUMAN	P04440	HLA-DPB1	29.1	15	2	4	No
Keratin, type I cytoskeletal 18	K1C18_HUMAN	P05783	KRT18	48	6	2	4	No
Complement component C8 gamma chain	CO8G_HUMAN	P07360	C8G	22.3	25	3	4	No
39S ribosomal protein L3, mitochondrial	RM03_HUMAN	P09001	MRPL3	38.6	12	3	4	No
Lymphocyte-specific protein 1	LSP1_HUMAN	P33241	LSP1	37.2	19	4	4	No
Glycogen synthase kinase-3 alpha	GSK3A_HUMAN	P49840	GSK3A	50.9	12	2	4	No
Selenide, water dikinase 1	SPS1_HUMAN	P49903	SEPHS1	42.9	8	2	4	No
Selenocysteine-specific elongation factor	SELB_HUMAN	P57772	EEFSEC	65.3	5	2	4	No
DDB1- and CUL4-associated factor 7	DCAF7_HUMAN	P61962	DCAF7	38.9	8	2	4	No
Visinin-like protein 1	VISL1_HUMAN	P62760	VSNL1	22.1	12	2	4	No
Desmocollin-1	DSC1_HUMAN	Q08554	DSC1	99.9	5	3	4	No
Protein disulfide-isomerase A2	PDIA2_HUMAN	Q13087	PDIA2	58.2	4	2	4	No
Tubulin--tyrosine ligase-like protein 12	TTL12_HUMAN	Q14166	TTLL12	74.4	6	2	4	No
Translation initiation factor eIF-2B subunit alpha	EI2BA_HUMAN	Q14232	EIF2B1	33.7	8	2	4	No
Fibroleukin	FGL2_HUMAN	Q14314	FGL2	50.2	8	2	4	No
Microtubule-associated tumor suppressor candidate 2	MTUS2_HUMAN	Q5JR59	MTUS2	150.1	2	2	4	No
Tetratricopeptide repeat protein 38	TTC38_HUMAN	Q5R3I4	TTC38	52.8	6	2	4	No
Ribulose-phosphate 3-epimerase	RPE_HUMAN	Q96AT9	RPE	24.9	4	2	4	No
Dehydrogenase/reductase SDR family member 1	DHRS1_HUMAN	Q96LJ7	DHRS1	33.9	13	2	4	No
Small proline-rich protein 4	SPRR4_HUMAN	Q96PI1	SPRR4	8.8	30	3	4	No
F-box-like/WD repeat-containing protein TBL1XR1	TBL1R_HUMAN	Q9BZK7	TBL1XR1	55.6	10	3	4	No
Protein FAM234A	F234A_HUMAN	Q9H0X4	FAM234A	59.6	3	2	4	No

WD repeat-containing protein 26	WDR26_HUMAN	Q9H7D7	WDR26	72.1	3	2	4	No
MYG1 exonuclease	MYG1_HUMAN	Q9HB07	MYG1	42.4	6	2	4	No
Stromal cell-derived factor 2-like protein 1	SDF2L_HUMAN	Q9HCN8	SDF2L1	23.6	14	2	4	No
ADP-ribose glycohydrolase ARH3	ADPRS_HUMAN	Q9NX46	ADPRS	38.9	8	2	4	No
ADP-ribosylation factor-like protein 15	ARL15_HUMAN	Q9NXU5	ARL15	22.9	14	2	4	No
SUMO-activating enzyme subunit 1	SAE1_HUMAN	Q9UBE0	SAE1	38.4	8	3	4	No
Peptidyl-tRNA hydrolase 2, mitochondrial	PTH2_HUMAN	Q9Y3E5	PTRH2	19.2	27	3	4	No
HBS1-like protein	HBS1L_HUMAN	Q9Y450	HBS1L	75.4	3	2	4	No
Plexin-D1	PLXD1_HUMAN	Q9Y4D7	PLXND1	211.9	2	2	4	No
Protein phosphatase methylesterase 1	PPME1_HUMAN	Q9Y570	PPME1	42.3	9	3	4	No
Platelet endothelial cell adhesion molecule	PECA1_HUMAN	P16284	PECAM1	82.5	3	2	3	No
Huntingtin-interacting protein 1	HIP1_HUMAN	O00291	HIP1	116.1	3	2	3	No
Exocyst complex component 3	EXOC3_HUMAN	O60645	EXOC3	86.8	4	2	3	No
Gamma-glutamylcyclotransferase	GGCT_HUMAN	O75223	GGCT	21	12	2	3	No
Glutamyl-tRNA	GATB_HUMAN	O75879	GATB	61.8	4	2	3	No
cGMP-specific 3',5'-cyclic phosphodiesterase	PDE5A_HUMAN	O76074	PDE5A	99.9	3	2	3	No
Metalloproteinase inhibitor 1	TIMP1_HUMAN	P01033	TIMP1	23.2	19	2	3	No
Uroporphyrinogen decarboxylase	DCUP_HUMAN	P06132	UROD	40.8	10	2	3	No
Myocardial zonula adherens protein	MYZAP_HUMAN	P0CAP1	MYZAP	54.2	5	2	3	No
Osteopontin	OSTP_HUMAN	P10451	SPP1	35.4	14	3	3	No
Pyruvate carboxylase, mitochondrial	PYC_HUMAN	P11498	PC	129.6	4	3	3	No
Inosine-5'-monophosphate dehydrogenase 2	IMDH2_HUMAN	P12268	IMPDH2	55.8	6	2	3	No
Carboxypeptidase M	CBPM_HUMAN	P14384	CPM	50.5	7	2	3	No
Cytoplasmic protein NCK1	NCK1_HUMAN	P16333	NCK1	42.8	5	2	3	No
Insulin-like growth factor-binding protein 2	IBP2_HUMAN	P18065	IGFBP2	34.8	9	2	3	No
Spermidine synthase	SPEE_HUMAN	P19623	SRM	33.8	11	2	3	No
Small proline-rich protein 2E	SPR2E_HUMAN	P22531	SPRR2E	7.9	36	2	3	No
Methylosome subunit pICln	ICLN_HUMAN	P54105	CLNS1A	26.2	33	3	3	No
RNA cytosine C	NSUN2_HUMAN	Q08J23	NSUN2	86.4	3	2	3	No
Dihydropyrimidine dehydrogenase [NADP	DPYD_HUMAN	Q12882	DPYD	111.3	2	2	3	No
Secreted phosphoprotein 24	SPP24_HUMAN	Q13103	SPP2	24.3	11	2	3	No
Rho guanine nucleotide exchange factor 6	ARHG6_HUMAN	Q15052	ARHGEF6	87.4	4	2	3	No
Cdc42-interacting protein 4	CIP4_HUMAN	Q15642	TRIP10	68.3	6	2	3	No
Phytanoyl-CoA dioxygenase domain-containing protein 1	PHYD1_HUMAN	Q5SRE7	PHYHD1	32.4	9	2	3	No
Putative hydroxypyruvate isomerase	HYI_HUMAN	Q5T013	HYI	30.4	16	3	3	No
Microtubule-associated protein 1S	MAP1S_HUMAN	Q66K74	MAP1S	112.1	3	2	3	No

Kinesin-like protein KIF21A	KI21A_HUMAN	Q7Z4S6	KIF21A	187.1	1	2	3	No
Exopolyphosphatase PRUNE1	PRUN1_HUMAN	Q86TP1	PRUNE1	50.2	6	2	3	No
Actin-histidine N-methyltransferase	SETD3_HUMAN	Q86TU7	SETD3	67.2	4	2	3	No
Prolyl 3-hydroxylase 3	P3H3_HUMAN	Q8IVL6	P3H3	81.8	4	2	3	No
Ubiquitin carboxyl-terminal hydrolase MINDY-1	MINY1_HUMAN	Q8N5J2	MINDY1	51.7	9	2	3	No
39S ribosomal protein L43, mitochondrial	RM43_HUMAN	Q8N983	MRPL43	23.4	10	2	3	No
Pleckstrin homology domain-containing family O member 2	PKHO2_HUMAN	Q8TD55	PLEKHO2	53.3	4	2	3	No
Biogenesis of lysosome-related organelles complex 1 subunit 5	BL1S5_HUMAN	Q8TDH9	BLOC1S5	21.6	10	2	3	No
Cytoglobin	CYGB_HUMAN	Q8WWM9	CYGB	21.4	9	2	3	No
Rab11 family-interacting protein 5	RFIP5_HUMAN	Q9BXF6	RAB11FIP5	70.4	6	3	3	No
Polyadenylate-binding protein-interacting protein 1	PAIP1_HUMAN	Q9H074	PAIP1	53.5	5	2	3	No
Cdc42 effector protein 4	BORG4_HUMAN	Q9H3Q1	CDC42EP4	38	14	2	3	No
Enolase-phosphatase E1	ENOPH_HUMAN	Q9UHY7	ENOPH1	28.9	10	2	3	No
Adseverin	ADSV_HUMAN	Q9Y6U3	SCIN	80.4	6	3	3	No
Pre-mRNA-splicing factor ATP-dependent RNA helicase DHX15	DHX15_HUMAN	O43143	DHX15	90.9	3	2	2	No
Proline dehydrogenase 1, mitochondrial	PROD_HUMAN	O43272	PRODH	68	4	2	2	No
Protein diaphanous homolog 2	DIAP2_HUMAN	O60879	DIAPH2	125.5	2	2	2	No
Secretogranin-1	SCG1_HUMAN	P05060	CHGB	78.2	4	2	2	No
Adenine phosphoribosyltransferase	APT_HUMAN	P07741	APRT	19.6	10	2	2	No
Protein 4.2	EPB42_HUMAN	P16452	EPB42	77	3	2	2	No
Lymphocyte function-associated antigen 3	LFA3_HUMAN	P19256	CD58	28.1	5	2	2	No
Integrin alpha-X	ITAX_HUMAN	P20702	ITGAX	127.7	2	2	2	No
Myelin protein P0	MYP0_HUMAN	P25189	MPZ	27.5	13	2	2	No
Insulin-like growth factor-binding protein complex acid labile subunit	ALS_HUMAN	P35858	IGFALS	66	4	2	2	No
Signal transducer and activator of transcription 1-alpha/beta	STAT1_HUMAN	P42224	STAT1	87.3	3	2	2	No
Lipopolysaccharide-responsive and beige-like anchor protein	LRBA_HUMAN	P50851	LRBA	318.9	1	2	2	No
Splicing factor U2AF 35 kDa subunit	U2AF1_HUMAN	Q01081	U2AF1	27.9	9	2	2	No
Bifunctional coenzyme A synthase	COASY_HUMAN	Q13057	COASY	62.3	5	2	2	No
Disintegrin and metalloproteinase domain-containing protein 9	ADAM9_HUMAN	Q13443	ADAM9	90.5	3	2	2	No
Vacuolar protein sorting-associated protein 26B	VP26B_HUMAN	Q4G0F5	VPS26B	39.1	9	2	2	No
Protein phosphatase 1 regulatory subunit 21	PPR21_HUMAN	Q6ZMI0	PPP1R21	88.3	3	2	2	No
Stromal membrane-associated protein 1	SMAP1_HUMAN	Q8IYB5	SMAP1	50.4	5	2	2	No
Marginal zone B- and B1-cell-specific protein	MZB1_HUMAN	Q8WU39	MZB1	20.7	16	2	2	No
Kallikrein-6	KLK6_HUMAN	Q92876	KLK6	26.8	9	2	2	No
Plasma alpha-L-fucosidase	FUCO2_HUMAN	Q9BTY2	FUCA2	54	4	2	2	No

Calcyclin-binding protein	CYBP_HUMAN	Q9HB71	CACYBP	26.2	8	2	2	No
Serine protease inhibitor Kazal-type 5	ISK5_HUMAN	Q9NQ38	SPINK5	120.6	2	2	2	No
Beta-catenin-interacting protein 1	CNBP1_HUMAN	Q9NSA3	CTNNBIP1	9.2	38	2	2	No
Glucose-fructose oxidoreductase domain-containing protein 1	GFOD1_HUMAN	Q9NXC2	GFOD1	43.1	5	2	2	No
CDKN2A-interacting protein	CARF_HUMAN	Q9NXV6	CDKN2AIP	61.1	4	2	2	No
Testin	TES_HUMAN	Q9UGI8	TES	48	5	2	2	No
Coiled-coil domain-containing protein 9	CCDC9_HUMAN	Q9Y3X0	CCDC9	59.7	6	2	2	No
Solute carrier family 12 member 7	S12A7_HUMAN	Q9Y666	SLC12A7	119	2	2	2	No

Supplemental Table VI. Quantification of proteins identified by proteomics in the GuHCl extracts of the human ischaemic heart failure cohort according to medication usage and presence of comorbidities.

Protein Information				Statins			Diabetes			ACE Inhibitors			Antiarrhythmics			Anticoagulants			ARBs			Atrial Fibrilation			Beta Blockers			Hypertension			Antiplatelet Therapy		
				yes=29 vs no=36			yes=27 vs no=38			yes=49 vs no=16			yes=28 vs no=37			yes=41 vs no=24			yes=13 vs no=52			yes=23 vs no=42			yes=44 vs no=21			yes=42 vs no=23			yes=29 vs no=36		
Protein Name	Accession number	Uniprot ID	Gene name	FC	P-value	BH Adjusted Pvalue	FC	P-value	BH Adjusted Pvalue	FC	P-value	BH Adjusted Pvalue	FC	P-value	BH Adjusted Pvalue	FC	P-value	BH Adjusted Pvalue	FC	P-value	BH Adjusted Pvalue	FC	P-value	BH Adjusted Pvalue	FC	P-value	BH Adjusted Pvalue	FC	P-value	BH Adjusted Pvalue	FC	P-value	BH Adjusted Pvalue
Adipocyte enhancer-binding protein 1	AEBP1_HUMAN	Q8IUX7	AEBP1	1.08	0.7354	0.9547	0.91	0.6804	0.9288	0.82	0.4762	0.8140	1.07	0.7643	0.9382	0.98	0.9229	0.9901	0.88	0.6627	0.9159	0.88	0.6253	0.9754	0.74	0.2610	0.6503	1.01	0.9740	0.9988	1.27	0.3120	0.9456
Agrin	AGRIN_HUMAN	O00468	AGRN	0.92	0.4643	0.8774	0.85	0.1642	0.9130	0.83	0.1860	0.7872	0.98	0.8542	0.9423	0.85	0.2053	0.9132	1.15	0.3379	0.9159	0.95	0.6882	0.9754	0.92	0.5396	0.8270	1.06	0.6222	0.9988	1.07	0.5741	0.9456
Alpha-1-acid glycoprotein 1	A1AG1_HUMAN	P02763	ORM1	0.95	0.8348	0.9735	1.17	0.5496	0.9288	1.39	0.2811	0.7872	0.82	0.4653	0.7848	0.87	0.6215	0.9701	0.70	0.2783	0.9159	1.09	0.7530	0.9754	0.90	0.7356	0.9223	1.17	0.5667	0.9988	1.16	0.5838	0.9456
Alpha-1-antichymotrypsin	AACT_HUMAN	P01011	SERPINA3	0.84	0.3517	0.8774	1.16	0.4416	0.9288	1.10	0.6563	0.8730	1.23	0.2705	0.7212	0.75	0.1319	0.9132	0.89	0.6200	0.9159	0.74	0.1382	0.9754	0.64	0.0323	0.3105	1.06	0.7741	0.9988	0.97	0.8600	0.9768
Alpha-1-antitrypsin	A1AT_HUMAN	P01009	SERPINA1	0.90	0.4312	0.8774	1.00	0.9720	0.9746	0.97	0.8696	0.9474	0.98	0.8689	0.9423	0.94	0.6592	0.9701	1.00	0.9820	0.9985	0.94	0.6848	0.9754	0.69	0.0151	0.2897	1.02	0.9068	0.9988	1.11	0.4519	0.9456
Alpha-1B-glycoprotein	A1BG_HUMAN	P04217	A1BG	0.90	0.4900	0.8774	0.85	0.2785	0.9130	0.89	0.5225	0.8140	1.05	0.7478	0.9382	0.90	0.5274	0.9701	1.08	0.6912	0.9159	0.94	0.6827	0.9754	0.80	0.1792	0.5464	1.02	0.9050	0.9988	1.09	0.5682	0.9456
Alpha-2-macroglobulin	A2MG_HUMAN	P01023	A2M	1.13	0.6064	0.8865	1.37	0.1966	0.9130	1.16	0.5922	0.8366	1.34	0.2221	0.6806	1.23	0.4158	0.9132	1.00	0.9981	0.9985	1.52	0.1058	0.9754	0.85	0.5472	0.8272	1.21	0.4497	0.9988	1.02	0.9306	0.9768
Angiogenin	ANGI_HUMAN	P03950	ANG	0.90	0.6216	0.8865	1.49	0.0728	0.9130	0.80	0.3941	0.8140	1.08	0.7384	0.9382	0.95	0.8387	0.9701	1.45	0.1729	0.9159	1.43	0.1258	0.9754	0.60	0.0396	0.3105	1.07	0.7617	0.9988	0.94	0.7787	0.9631
Annexin A1	ANXA1_HUMAN	P04083	ANXA1	1.07	0.7243	0.9544	0.88	0.5339	0.9288	0.93	0.7342	0.9081	1.04	0.8261	0.9423	1.25	0.2755	0.9132	1.07	0.7807	0.9409	1.09	0.6689	0.9754	0.87	0.5335	0.8270	0.79	0.2390	0.9988	1.37	0.1064	0.9456
Annexin A11	ANX11_HUMAN	P50995	ANXA11	0.89	0.2362	0.8774	0.89	0.2606	0.9130	0.88	0.2678	0.7872	1.03	0.7886	0.9423	0.95	0.6254	0.9701	1.15	0.2715	0.9159	0.99	0.8912	0.9754	1.04	0.7181	0.9223	1.05	0.6169	0.9988	1.03	0.7444	0.9456
Annexin A2	ANXA2_HUMAN	P07355	ANXA2	0.82	0.1410	0.8568	1.13	0.3755	0.9130	0.90	0.5130	0.8140	1.04	0.7786	0.9423	0.77	0.0625	0.9132	1.21	0.2497	0.9159	0.95	0.7265	0.9754	0.99	0.9416	0.9762	1.11	0.4297	0.9988	0.94	0.6615	0.9456
Annexin A6	ANXA6_HUMAN	P08133	ANXA6	0.77	0.1886	0.8774	1.02	0.9144	0.9694	0.91	0.6714	0.8766	0.76	0.1580	0.6806	0.78	0.2376	0.9132	1.29	0.2946	0.9159	0.73	0.1409	0.9754	0.91	0.6831	0.9223	1.05	0.7972	0.9988	1.08	0.6958	0.9456
Antithrombin-III	ANT3_HUMAN	P01008	SERPINC1	0.87	0.5924	0.8793	0.93	0.7815	0.9404	0.78	0.4120	0.8140	0.76	0.2843	0.7212	0.79	0.3903	0.9132	1.36	0.3467	0.9159	0.76	0.3205	0.9754	0.78	0.4054	0.7212	1.21	0.4866	0.9988	1.23	0.4203	0.9456
Apolipoprotein A-I	APOA1_HUMAN	P02647	APOA1	1.05	0.7516	0.9547	0.85	0.2830	0.9130	0.73	0.0704	0.6812	0.83	0.2052	0.6806	1.13	0.4450	0.9364	1.43	0.0549	0.8916	0.77	0.1016	0.9754	0.86	0.3659	0.7212	1.03	0.8584	0.9988	1.34	0.0503	0.9456
Apolipoprotein A-II	APOA2_HUMAN	P02652	APOA2	1.11	0.5176	0.8777	0.75	0.0797	0.9130	0.75	0.1168	0.7192	0.89	0.4541	0.7848	1.01	0.9542	0.9901	1.38	0.1029	0.8916	0.72	0.0578	0.9754	1.29	0.1575	0.5289	0.93	0.6761	0.9988	0.96	0.8158	0.9768
Apolipoprotein A-IV	APOA4_HUMAN	P06727	APOA4	1.02	0.9467	0.9865	1.19	0.5041	0.9288	0.84	0.5628	0.8140	1.21	0.4484	0.7848	1.09	0.7559	0.9701	1.25	0.4733	0.9159	1.03	0.9192	0.9754	0.81	0.4467	0.7498	0.90	0.6818	0.9988	0.94	0.7931	0.9724
Apolipoprotein C-III	APOC3_HUMAN	P02656	APOC3	1.09	0.5821	0.8793	0.96	0.8130	0.9404	0.84	0.3569	0.8140	0.97	0.8360	0.9423	1.02	0.9243	0.9901	1.20	0.3419	0.9159	0.96	0.8090	0.9754	1.04	0.8286	0.9422	0.97	0.8364	0.9988	0.88	0.4220	0.9456
Apolipoprotein E	APOE_HUMAN	P02649	APOE	0.67	0.0427	0.6326	1.09	0.6594	0.9288	0.65	0.0626	0.6812	0.97	0.8970	0.9474	0.65	0.0360	0.9132	1.40	0.1734	0.9159	0.86	0.4934	0.9754	0.53	0.0030	0.2125	0.91	0.6359	0.9988	0.75	0.1473	0.9456
Asporin	ASPN_HUMAN	Q9BXN1	ASPN	0.84	0.4741	0.8774	0.80	0.3533	0.9130	0.78	0.3805	0.8140	1.22	0.4120	0.7848	0.96	0.8737	0.9701	1.11	0.7204	0.9203	1.14	0.6181	0.9754	0.59	0.0481	0.3205	1.21	0.4534	0.9988	1.21	0.4328	0.9456
Basal cell adhesion molecule	BCAM_HUMAN	P50895	BCAM	0.93	0.4252	0.8774	0.98	0.7803	0.9404	1.08	0.4842	0.8140	0.94	0.4847	0.7900	0.90	0.2579	0.9132	0.89	0.2671	0.9159	1.02	0.8568	0.9754	1.09	0.3908	0.7212	0.87	0.1151	0.9988	0.90	0.2117	0.9456
Basement membrane-specific heparan sulfate proteoglycan core protein	PGBM_HUMAN	P98160	HSPG2	1.04	0.5723	0.8777	1.02	0.7238	0.9404	0.95	0.5486	0.8140	1.07	0.2790	0.7212	1.02	0.7749	0.9701	1.05	0.5897	0.9159	1.04	0.5612	0.9754	1.01	0.9194	0.9762	0.97	0.7070	0.9988	1.03	0.6875	0.9456
Beta-2-glycoprotein 1	APOH_HUMAN	P02749	APOH	0.74	0.0529	0.6326	0.90	0.5026	0.9288	0.71	0.0618	0.6812	1.22	0.2068	0.6806	0.73	0.0519	0.9132	1.45	0.0516	0.8916	1.13	0.4674	0.9754	1.00	0.9774	0.9915	0.95	0.7351	0.9988	0.85	0.2915	0.9456
Beta-2-microglobulin	B2MG_HUMAN	P61769	B2M	0.83	0.2365	0.8774	1.07	0.6637	0.9288	0.93	0.6788	0.8781	1.23	0.1763	0.6806	0.76	0.0919	0.9132	0.96	0.8430	0.9593	1.11	0.5466	0.9754	0.90	0.5394	0.8270	1.04	0.8111	0.9988	0.88	0.4277	0.9456
Biglycan	PGS1_HUMAN	P21810	BGN	1.08	0.7443	0.9547	0.86	0.4953	0.9288	0.67	0.1208	0.7192	1.39	0.1458	0.6806	1.19	0.4725	0.9496	1.27	0.3918	0.9159	1.06	0.8158	0.9754	0.51	0.0067	0.2155	1.00	0.9894	0.9988	1.30	0.2523	0.9456
Cadherin-13	CAD13_HUMAN	P55290	CDH13	0.93	0.5414	0.8777	0.88	0.3073	0.9130	0.98	0.8869	0.9474	0.94	0.6158	0.8861	0.92	0.5201	0.9701	0.97	0.8556	0.9593	1.18	0.2012	0.9754	1.05	0.7078	0.9223	0.91	0.4451	0.9988	0.83	0.1102	0.9456
Calpastatin	ICAL_HUMAN	P20810	CAST	0.93	0.5899	0.8793	0.86	0.2628	0.9130	1.03	0.8321	0.9345	0.94	0.6663	0.9382	0.80	0.1216	0.9132	0.91	0.5563	0.9159	0.71	0.0183	0.9754	0.96	0.7876	0.9254	0.79	0.0881	0.9988	0.87	0.3195	0.9456
Cartilage intermediate layer protein 1	CILP1_HUMAN	O75339	CILP	0.86	0.5222	0.8777	0.88	0.5826	0.9288	1.10	0.7119	0.9043	0.86	0.4986	0.7900	0.72	0.1703	0.9132	1.00	0.9932	0.9985	1.25	0.3533	0.9754	0.93	0.7643	0.9229	1.09	0.7207	0.9988	0.87	0.5323	0.9456
Cathepsin B	CATB_HUMAN	P07858	CTSB	0.98	0.8873	0.9865	1.17	0.3262	0.9130	0.82	0.2822	0.7872	1.00	0.9873	0.9920	0.84	0.2904	0.9132	1.33	0.1401	0.9159	0.93	0.6789	0.9754	0.81	0.2326	0.6308	0.92	0.6170	0.9988	0.80	0.1422	0.9456
Cathepsin D	CATD_HUMAN	P07339	CTSD	1.12	0.3027	0.8774	1.10	0.3984	0.9288	0.81	0.0979	0.7192	1.01	0.9216	0.9555	1.01	0.9507	0.9901	1.33	0.0313	0.8916	0.86	0.1943	0.9754	1.02	0.8967	0.9762	0.89	0.2732	0.9988	0.97	0.7516	0.9463
Cathepsin G	CATG_HUMAN	P08311	CTSG	0.66	0.0165	0.6326	1.13	0.4856	0.9288	1.38	0.1105	0.7192	1.08	0.6570	0.9358	0.76	0.1180	0.9132	0.78	0.2567	0.9159	1.22	0.2872	0.9754	0.71	0.0756	0.3554	1.11	0.5449	0.9988	0.86	0.3736	0.9456
CD59 glycoprotein	CD59_HUMAN	P13987	CD59	1.08	0.8122	0.9735	0.69	0.2363	0.9130	0.51	0.0663	0.6812	0.51	0.0308	0.6806	0.73	0.3445	0.9132	1.98	0.0784	0.8916	0.95	0.8860	0.9754	1.03	0.9235	0.9762	1.39	0.3158	0.9988	0.73	0.3195	0.9456
Cell surface glycoprotein MUC18	MUC18_HUMAN	P43121	MCAM	1.01	0.9589	0.9865	0.85	0.2818	0.9130	1.13	0.5064	0.8140	0.78	0.1005	0.6806	0.87	0.3756	0.9132	0.77	0.1676	0.9159	1.07	0.6658	0.9754	1.22	0.2543	0.6503	0.98	0.9017	0.9988	0.87	0.3840	0.9456
Chondroitin sulfate proteoglycan 4	CSPG4_HUMAN	Q6UVK1	CSPG4	0.99	0.9466	0.9865	0.89	0.5344	0.9288	1.50	0.0610	0.6812	0.96	0.8421	0.9423	0.78	0.2045	0.9132	0.61	0.0295	0.8916	1.20	0.3529	0.9754	0.82	0.3392	0.7138	1.11	0.5949	0.9988	0.84	0.3546	0.9456
Chymase	CMA1_HUMAN	P23946	CMA1	0.78	0.0624	0.6771	1.13	0.3695	0.9130	1.34	0.0598	0.6812	1.22	0.1408	0.6806	0.95	0.6997	0.9701	0.87	0.4081	0.9159	1.12	0.4394	0.9754	0.90	0.4932	0.7902	1.28	0.0794	0.9988	0.95	0.7175	0.9456
Clusterin	CLUS_HUMAN	P10909	CLU	0.89	0.4553	0.8774	1.07	0.6953	0.9288	0.90	0.5933	0.8366	1.27	0.1353	0.6806	0.89	0.4703	0.9496	1.02	0.9051	0.9855	1.09	0.6344	0.9754	0.65	0.0140	0.2897	0.94	0.7181	0.9988	0.97	0.8609	0.9768
Collagen alpha-1(I) chain	CO1A1_HUMAN	P02452	COL1A1	0.77	0.2116	0.8774	0.78	0.2510	0.9130	0.89	0.6402	0.8645	1.17	0.4588	0.7848	0.91	0.6561	0.9701	1.05	0.8416	0.9593	0.73	0.1622	0.9754	0.66	0.0749	0.3554	1.02	0.9266	0.9988	1.18	0.4366	0.9456
Collagen alpha-1(III) chain	CO3A1_HUMAN	P02461	COL3A1	0.76	0.1458	0.8568	0.82	0.3120	0.9130	1.03	0.9051	0.9596	1.08	0.6985	0.9382	0.88	0.5011	0.9701	0.90	0.6574	0.9159	0.96	0.8441	0.9754	0.71	0.1104	0.4325	1.13	0.5196	0.9988	1.08	0.6855	0.9456
Collagen alpha-1(IV) chain	CO4A1_HUMAN	P02462	COL4A1	0.71	0.0242	0.6326	1.15	0.3556	0.9130	1.12	0.5167	0.8140	1.13	0.4174	0.7848	0.71	0.0280	0.9132	0.74	0.1163	0.9112	1.14	0.4159	0.9754	0.88	0.4449	0.7498	0.90	0.5084	0.9988	0.72	0.0309	0.9456
Collagen alpha-1(V) chain	CO5A1_HUMAN	P20908	COL5A1	0.82	0.3884	0.8774	0.72	0.1621	0.9130	0.67	0.1355	0.7348	1.36	0.1805	0.6806	0.95	0.8181	0.9701	1.30	0.3589	0.9159	0.87	0.5640	0.9754	0.64	0.0897	0.3847	0.96	0.8707	0.9988	1.19	0.4629	0.9456
Collagen alpha-1(VI) chain	CO6A1_HUMAN	P12109	COL6A1	0.89	0.4360	0.8774	1.07	0.6697	0.9288	1.04	0.8069	0.9335	0.92	0.5825	0.8737	0.81	0.1786	0.9132	0.94	0.7376	0.9285	1.01	0.9361	0.9754	0.74	0.0643	0.3554	1.13	0.4272	0.9988	1.00	0.9807	0.9978
Collagen alpha-1(XII) chain	COCA1_HUMAN	Q99715	COL12A1	1.23	0.3711	0.8774	0.88	0.5766	0.9288	1.27	0.3775	0.8140	0.72	0.1579	0.6806	1.24	0.3730	0.9132	0.87	0.6271	0.9159	0.76	0.2578	0.9754	0.96	0.8871	0.9762	0.70	0.1315	0.9988	1.41	0.1376	0.9456
Collagen alpha-1(XIV) chain	COEA1_HUMAN	Q05707	COL14A1	0.87	0.5622	0.8777	0.99	0.9651	0.9746	0.88	0.6438	0.8645	1.10	0.6945	0.9382	0.88	0.6147	0.9701	1.18	0.5786	0.9159	0.84	0.5004	0.9754	0.61	0.0696	0.3554	1.13	0.6294	0.9988	1.14	0.5978	0.9456
Collagen alpha-1(XV) chain	COFA1_HUMAN	P39059	COL15A1	0.77	0.0977	0.8545	1.02	0.8856	0.9532	0.96	0.8262	0.9345	1.12	0.4731	0.7848	0.86	0.3713	0.9132	1.06	0.7547	0.9409	0.99	0.9318	0.9754	0.88	0.4522	0.7502	0.84	0.2734	0.9988	0.98	0.8753	0.9768
Collagen alpha-1(XVIII) chain	COIA1_HUMAN	P39060	COL18A1	1.05	0.7938	0.9733	1.18	0.3194	0.9130	1.00	0.9946	0.9988	1.41	0.0392	0.6806	1.02	0.9028	0.9868	0.90	0.6096	0.9159	0.88	0.4607	0.9754	0.66	0.0238	0.3105	1.01	0.9724	0.9988	1.10	0.5760	0.9456
Collagen alpha-2(I) chain	CO1A2_HUMAN	P08123	COL1A2	0.80	0.3003	0.8774	0.77	0.2258	0.9130	0.83	0.4501	0.8140	1.13	0.5654	0.8572	0.92	0.7063	0.9701	1.11	0.6958	0.9159	0.73	0.1623	0.9754	0.63	0.0469	0.3205	0.95	0.8287	0.9988	1.18	0.4380	0.9456
Collagen alpha-2(IV) chain	CO4A2_HUMAN	P08572	COL4A2	0.84	0.1253	0.8545	1.14	0.2575	0.9130	1.00	0.9811	0.9988	1.04	0.7562	0.9382	0.86	0.1947	0.9132	0.90	0.4804	0.9159	1.07	0.5852	0.9754	0.89	0.3546	0.7212	0.98	0.8913	0.9988	0.94	0.5724	0.9456
Collagen alpha-2(V) chain	CO5A2_HUMAN	P05997	COL5A2	0.76	0.3156	0.8774	0.71	0.2053	0.9130	0.72	0.2810	0.7872	1.28	0.3551	0.7703	0.98	0.9534	0.9901	1.28	0.4618	0.9159	0.78	0.3888	0.9754	0.57	0.0599	0.3537	1.02	0.9538	0.9988	1.26	0.3883	0.9456
Collagen alpha-2(VI) chain	CO6A2_HUMAN	P12110	COL6A2	0.98	0.9179	0.9865	1.13	0.5373	0.9288	0.81	0.3672	0.8140	0.94	0.7322	0.9382	0.81	0.3097	0.9132	1.19	0.4779	0.9159	1.08	0.7155	0.9754	0.71	0.1151	0.4387	1.27	0.2393	0.9988	1.07	0.7204	0.9456
Collagen alpha-3(V) chain	CO5A3_HUMAN	P25940	COL5A3	0.82	0.4586	0.8774	0.96	0.8721	0.9532	0.91	0.7553	0.9171	1.32	0.2806	0.7212	0.88	0.6313	0.9701	1.00	0.9985	0.9985	1.19	0.5238	0.9754	0.76	0.3371	0.7138	0.90	0.6988	0.9988	0.96	0.8708	0.9768
Collagen alpha-3(VI) chain	CO6A3_HUMAN	P12111	COL6A3	0.92	0.6224	0.8865	1.06	0.7289	0.9404	0.99	0.9675	0.9957	0.93	0.6841	0.9382	0.83	0.2849	0.9132	0.99	0.9624	0.9905	1.02	0.9005	0.9754	0.78	0.1785	0.5464	1.03	0.8509	0.9988	1.00	0.9907	0.9978
Collagen alpha-6(VI) chain	CO6A6_HUMAN	A6NMZ7	COL6A6	0.83	0.3586	0.8774	1.12	0.5739	0.9288	0.85	0.5030	0.8140	1.37	0.1207	0.6806	0.73	0.1349	0.9132	1.15	0.5869	0.9159	1.06	0.7981	0.9754	1.08	0.7451	0.9223	1.22	0.3505	0.9988	0.73	0.1270	0.9456
Cystatin-B	CYTB_HUMAN	P04080	CSTB	0.99	0.9007	0.9865	0.94	0.6108	0.9288	0.82	0.1443	0.7486	1.00	0.9894	0.9920	0.86	0.2089	0.9132	1.16	0.3100	0.9159	0.81	0.0894	0.9754	0.93	0.6059	0.8807	0.82	0.1007	0.9988	0.85	0.1649	0.9456
Cystatin-C	CYTC_HUMAN	P01034	CST3	1.09	0.3916	0.8774	1.01	0.9565	0.9746	0.83	0.1100	0.7192	1.24	0.0263	0.6806	1.15	0.1714	0.9132	1.22	0.1048	0.8916	1.03	0.7616	0.9754	0.96	0.7276	0.9223	0.92	0.4168	0.9988	0.96	0.6730	0.9456
Cysteine-rich protein 2	CRIP2_HUMAN	P52943	CRIP2	1.07	0.4036	0.8774	0.84	0.0371	0.9130	0.97	0.7681	0.9171	0.97	0.7114	0.9382	1.01	0.8736	0.9701	1.07	0.5096	0.9159	0.93	0.4372	0.9754	1.08	0.3968	0.7212	0.89	0.1887	0.9988	0.95	0.5667	0.9456
Decorin	PGS2_HUMAN	P07585	DCN	0.87	0.4899	0.8774	0.88	0.5211	0.9288	0.87	0.5467	0.8140	1.29	0.1842	0.6806	1.00	0.9901	0.9901	1.06	0.8181	0.9593	1.19	0.4088	0.9754	0.68	0.0706	0.3554	1.04	0.8472	0.9988	1.18	0.3972	0.9456
Dermatopontin	DERM_HUMAN	Q07507	DPT	0.76	0.2155	0.8774	0.83	0.3945	0.9288	0.84	0.4972	0.8140	1.25	0.3030	0.7212	0.89	0.6138	0.9701	1.11	0.6926	0.9159	1.14	0.5659	0.9754	0.63	0.0602	0.3537	1.08	0.7219	0.9988	1.18	0.4461	0.9456
Dermcidin	DCD_HUMAN	P81605	DCD	0.95	0.7653	0.9621	1.04	0.8104	0.9404	0.70	0.0776	0.6812	1.30	0.1303	0.6806	0.93	0.7139	0.9701	1.50	0.0602	0.8916	1.05	0.8108	0.9754	0.93	0.7204	0.9223	1.06	0.7384	0.9988	0.90	0.5430	0.9456
Dystroglycan	DAG1_HUMAN	Q14118	DAG1	1.06	0.4145	0.8774	0.95	0.4224	0.9288	1.00	0.9988	0.9988	1.02	0.7391	0.9382	1.02	0.8087	0.9701	0.96	0.6321	0.9159	0.95	0.5021	0.9754	1.00	0.9901	0.9971	0.93	0.3113	0.9988	0.92	0.2526	0.9456
EMILIN-1	EMIL1_HUMAN	Q9Y6C2	EMILIN1	0.67	0.0453	0.6326	1.18	0.4200	0.9288	1.16	0.5349	0.8140	1.11	0.6030	0.8766	0.68	0.0662	0.9132	0.84	0.4965	0.9159	0.97	0.8930	0.9754	0.51	0.0020	0.2125	1.00	0.9988	0.9988	0.88	0.5400	0.9456
Extracellular superoxide dismutase [Cu-Zn]	SODE_HUMAN	P08294	SOD3	0.89	0.5561	0.8777	0.78	0.2248	0.9130	0.95	0.8448	0.9345	1.37	0.1183	0.6806	1.14	0.5431	0.9701	0.90	0.6942	0.9159	0.93	0.7474	0.9754	0.82	0.3909	0.7212	0.92	0.6792	0.9988	1.13	0.5496	0.9456
Fibrillin-1	FBN1_HUMAN	P35555	FBN1	0.82	0.2128	0.8774	0.83	0.2178	0.9130	0.91	0.6022	0.8408	0.86	0.3425	0.7545	0.79	0.1335	0.9132	1.07	0.7155	0.9203	1.11	0.5433	0.9754	0.80	0.1906	0.5599	1.05	0.7415	0.9988	1.12	0.4615	0.9456
Fibromodulin	FMOD_HUMAN	Q06828	FMOD	1.04	0.9149	0.9865	0.59	0.1926	0.9130	0.44	0.0827	0.6812	1.02	0.9594	0.9803	1.03	0.9368	0.9901	1.81	0.2407	0.9159	0.97	0.9528	0.9754	0.68	0.4042	0.7212	0.92	0.8537	0.9988	1.29	0.5377	0.9456
Fibronectin	FINC_HUMAN	P02751	FN1	0.91	0.5041	0.8774	0.94	0.6712	0.9288	0.93	0.6291	0.8611	1.30	0.0462	0.6806	0.97	0.8401	0.9701	0.97	0.8562	0.9593	0.96	0.7580	0.9754	0.72	0.0269	0.3105	1.04	0.7530	0.9988	1.17	0.2495	0.9456
Fibulin-2	FBLN2_HUMAN	P98095	FBLN2	0.77	0.2685	0.8774	0.96	0.8816	0.9532	0.90	0.7082	0.9043	1.19	0.4671	0.7848	0.82	0.4274	0.9132	0.90	0.7245	0.9203	1.31	0.2849	0.9754	0.48	0.0049	0.2155	1.20	0.4588	0.9988	1.05	0.8379	0.9768
Galectin-1	LEG1_HUMAN	P09382	LGALS1	0.84	0.1113	0.8545	0.80	0.0474	0.9130	0.96	0.7740	0.9171	1.08	0.4963	0.7900	0.86	0.2097	0.9132	1.06	0.6719	0.9159	0.92	0.5012	0.9754	0.94	0.6468	0.9120	0.83	0.1008	0.9988	0.95	0.6400	0.9456
Galectin-3	LEG3_HUMAN	P17931	LGALS3	1.06	0.4950	0.8774	1.05	0.6017	0.9288	0.92	0.3947	0.8140	1.26	0.0070	0.4924	1.09	0.3524	0.9132	1.09	0.4496	0.9159	1.00	0.9739	0.9805	0.92	0.4002	0.7212	0.88	0.1649	0.9988	0.99	0.8923	0.9768
Galectin-3-binding protein	LG3BP_HUMAN	Q08380	LGALS3BP	1.23	0.1722	0.8774	1.33	0.0595	0.9130	0.82	0.2779	0.7872	0.95	0.7251	0.9382	0.95	0.7359	0.9701	1.16	0.4374	0.9159	0.77	0.1150	0.9754	1.06	0.7408	0.9223	0.99	0.9384	0.9988	0.82	0.2014	0.9456
Gelsolin	GELS_HUMAN	P06396	GSN	1.06	0.3227	0.8774	0.95	0.3641	0.9130	1.01	0.8483	0.9345	1.06	0.3113	0.7212	1.08	0.2336	0.9132	0.95	0.5436	0.9159	0.96	0.5204	0.9754	1.05	0.4617	0.7570	0.98	0.8091	0.9988	1.03	0.6815	0.9456
Hepatoma-derived growth factor	HDGF_HUMAN	P51858	HDGF	0.95	0.7000	0.9311	0.89	0.3560	0.9130	1.18	0.2568	0.7872	1.23	0.1078	0.6806	1.01	0.9573	0.9901	0.82	0.2073	0.9159	0.81	0.1210	0.9754	0.87	0.3282	0.7119	1.04	0.7518	0.9988	1.14	0.3007	0.9456
Hepatoma-derived growth factor-related protein 3	HDGR3_HUMAN	Q9Y3E1	HDGFL3	1.39	0.0278	0.6326	0.88	0.4144	0.9288	0.73	0.0666	0.6812	1.12	0.4678	0.7848	1.18	0.2849	0.9132	1.31	0.1406	0.9159	0.85	0.3082	0.9754	1.07	0.6991	0.9223	0.72	0.0308	0.9988	1.19	0.2456	0.9456
Hyaluronan and proteoglycan link protein 1	HPLN1_HUMAN	P10915	HAPLN1	1.08	0.8360	0.9735	0.51	0.0712	0.9130	0.70	0.4103	0.8140	1.58	0.2202	0.6806	1.43	0.3627	0.9132	0.74	0.5156	0.9159	1.90	0.1072	0.9754	0.64	0.2842	0.6570	1.12	0.7682	0.9988	1.69	0.1585	0.9456
Insulin-like growth factor-binding protein 7	IBP7_HUMAN	Q16270	IGFBP7	1.11	0.4851	0.8774	0.96	0.7672	0.9404	0.71	0.0429	0.6812	1.17	0.2911	0.7212	1.01	0.9698	0.9901	1.35	0.0911	0.8916	0.92	0.5961	0.9754	0.83	0.2461	0.6503	0.99	0.9232	0.9988	1.14	0.3725	0.9456
Integrin alpha-1	ITA1_HUMAN	P56199	ITGA1	0.78	0.1321	0.8545	1.11	0.5314	0.9288	1.07	0.7189	0.9051	0.86	0.3809	0.7848	0.81	0.2320	0.9132	0.87	0.4909	0.9159	1.17	0.3817	0.9754	0.73	0.0974	0.3923	1.26	0.1729	0.9988	0.97	0.8404	0.9768
Integrin alpha-V	ITAV_HUMAN	P06756	ITGAV	1.00	0.9987	0.9987	0.83	0.2078	0.9130	0.91	0.5578	0.8140	0.88	0.3891	0.7848	1.00	0.9856	0.9901	1.07	0.7001	0.9159	1.08	0.6257	0.9754	0.97	0.8591	0.9691	1.07	0.6577	0.9988	1.20	0.2013	0.9456
Integrin beta-1	ITB1_HUMAN	P05556	ITGB1	1.16	0.4064	0.8774	1.11	0.5545	0.9288	0.80	0.2800	0.7872	0.88	0.4479	0.7848	0.95	0.7636	0.9701	1.11	0.6284	0.9159	0.98	0.9164	0.9754	1.00	0.9980	0.9980	1.05	0.7970	0.9988	0.91	0.5993	0.9456
Inter-alpha-trypsin inhibitor heavy chain H1	ITIH1_HUMAN	P19827	ITIH1	1.09	0.7486	0.9547	1.12	0.6800	0.9288	0.49	0.0186	0.6812	1.34	0.2700	0.7212	0.80	0.4244	0.9132	2.10	0.0239	0.8916	0.95	0.8702	0.9754	0.72	0.2821	0.6570	1.89	0.0195	0.9988	1.03	0.9108	0.9768
Inter-alpha-trypsin inhibitor heavy chain H2	ITIH2_HUMAN	P19823	ITIH2	0.82	0.3464	0.8774	0.85	0.4453	0.9288	0.85	0.4975	0.8140	0.96	0.8541	0.9423	1.08	0.7398	0.9701	0.97	0.9156	0.9855	0.93	0.7442	0.9754	0.72	0.1560	0.5289	0.97	0.8978	0.9988	0.91	0.6718	0.9456
Kininogen-1	KNG1_HUMAN	P01042	KNG1	1.07	0.6486	0.9050	1.04	0.7895	0.9404	0.85	0.3389	0.8140	0.98	0.8680	0.9423	1.11	0.5109	0.9701	1.15	0.4456	0.9159	0.91	0.5368	0.9754	0.80	0.1677	0.5464	0.93	0.6153	0.9988	1.27	0.0999	0.9456
Lactadherin	MFGM_HUMAN	Q08431	MFGE8	1.10	0.6559	0.9050	0.82	0.3601	0.9130	0.74	0.2364	0.7872	1.16	0.4982	0.7900	1.07	0.7610	0.9701	1.36	0.2492	0.9159	0.98	0.9322	0.9754	0.95	0.8230	0.9422	1.04	0.8522	0.9988	1.41	0.1103	0.9456
Laminin subunit alpha-2	LAMA2_HUMAN	P24043	LAMA2	1.09	0.3783	0.8774	1.10	0.3618	0.9130	0.97	0.7818	0.9186	1.19	0.0891	0.6806	1.03	0.7954	0.9701	1.04	0.7681	0.9409	1.07	0.5413	0.9754	1.10	0.3924	0.7212	1.01	0.9207	0.9988	0.99	0.9438	0.9768
Laminin subunit alpha-4	LAMA4_HUMAN	Q16363	LAMA4	0.86	0.2402	0.8774	1.03	0.8261	0.9404	0.88	0.3872	0.8140	1.02	0.9020	0.9474	0.79	0.0682	0.9132	1.12	0.4695	0.9159	1.02	0.9099	0.9754	1.05	0.7318	0.9223	1.07	0.6013	0.9988	0.88	0.2829	0.9456
Laminin subunit alpha-5	LAMA5_HUMAN	O15230	LAMA5	0.86	0.2275	0.8774	1.02	0.8510	0.9532	0.92	0.5657	0.8140	0.93	0.5978	0.8766	0.78	0.0647	0.9132	0.98	0.9090	0.9855	1.05	0.7074	0.9754	1.01	0.9382	0.9762	0.99	0.9491	0.9988	0.94	0.6353	0.9456
Laminin subunit beta-1	LAMB1_HUMAN	P07942	LAMB1	1.03	0.7933	0.9733	1.06	0.5640	0.9288	0.85	0.1757	0.7872	1.21	0.0744	0.6806	0.98	0.8612	0.9701	1.15	0.2785	0.9159	0.99	0.9076	0.9754	1.05	0.6782	0.9223	1.00	0.9963	0.9988	1.07	0.5153	0.9456
Laminin subunit beta-2	LAMB2_HUMAN	P55268	LAMB2	0.93	0.4591	0.8774	0.99	0.9543	0.9746	0.96	0.7559	0.9171	0.94	0.5073	0.7947	0.88	0.2011	0.9132	1.03	0.8283	0.9593	1.07	0.5472	0.9754	1.10	0.4184	0.7212	0.95	0.5847	0.9988	0.91	0.3702	0.9456
Laminin subunit gamma-1	LAMC1_HUMAN	P11047	LAMC1	0.91	0.3253	0.8774	1.02	0.8270	0.9404	1.03	0.8143	0.9335	0.98	0.8256	0.9423	0.87	0.1751	0.9132	0.96	0.7617	0.9409	1.05	0.6700	0.9754	1.10	0.4019	0.7212	0.94	0.5702	0.9988	0.91	0.3498	0.9456
Latent-transforming growth factor beta-binding protein 2	LTBP2_HUMAN	Q14767	LTBP2	1.17	0.4989	0.8774	0.69	0.1138	0.9130	0.94	0.8140	0.9335	1.26	0.3311	0.7411	1.19	0.4782	0.9496	0.86	0.5931	0.9159	1.12	0.6523	0.9754	0.67	0.1272	0.4721	0.90	0.6505	0.9988	1.10	0.6718	0.9456
Latent-transforming growth factor beta-binding protein 4	LTBP4_HUMAN	Q8N2S1	LTBP4	0.90	0.3269	0.8774	0.96	0.6983	0.9288	1.09	0.5156	0.8140	1.05	0.6776	0.9382	0.90	0.3460	0.9132	0.88	0.3750	0.9159	0.93	0.5444	0.9754	0.78	0.0431	0.3197	1.06	0.5864	0.9988	0.94	0.5919	0.9456
Leukocyte elastase inhibitor	ILEU_HUMAN	P30740	SERPINB1	0.71	0.1192	0.8545	1.38	0.1504	0.9130	1.33	0.2727	0.7872	1.38	0.1473	0.6806	0.83	0.4249	0.9132	0.83	0.5098	0.9159	0.85	0.5014	0.9754	0.59	0.0333	0.3105	1.14	0.5706	0.9988	1.00	0.9857	0.9978
Lumican	LUM_HUMAN	P51884	LUM	1.00	0.9905	0.9976	0.92	0.6980	0.9288	0.73	0.2217	0.7872	1.26	0.3048	0.7212	1.08	0.7486	0.9701	1.24	0.4286	0.9159	1.16	0.5232	0.9754	0.59	0.0297	0.3105	1.01	0.9728	0.9988	1.19	0.4369	0.9456
Lysozyme C	LYSC_HUMAN	P61626	LYZ	0.91	0.5727	0.8777	1.24	0.1996	0.9130	1.09	0.6639	0.8749	1.37	0.0555	0.6806	0.86	0.3824	0.9132	0.92	0.7015	0.9159	1.48	0.0240	0.9754	0.88	0.4771	0.7732	1.27	0.1580	0.9988	0.88	0.4469	0.9456
Macrophage migration inhibitory factor	MIF_HUMAN	P14174	MIF	1.02	0.9203	0.9865	0.99	0.9646	0.9746	0.84	0.3961	0.8140	0.86	0.4018	0.7848	0.93	0.6960	0.9701	1.31	0.2199	0.9159	0.76	0.1449	0.9754	0.95	0.7819	0.9254	0.98	0.9198	0.9988	1.19	0.3317	0.9456
Mast cell carboxypeptidase A	CBPA3_HUMAN	P15088	CPA3	0.90	0.6611	0.9050	1.37	0.1983	0.9130	1.04	0.8850	0.9474	1.57	0.0608	0.6806	1.06	0.8254	0.9701	1.18	0.5793	0.9159	1.03	0.9183	0.9754	0.70	0.1821	0.5464	1.78	0.0197	0.9988	1.03	0.9181	0.9768

Matrix-remodeling-associated protein 5	MXRA5_HUMAN	Q9NR99	MXRA5	0.85	0.1170	0.8545	1.06	0.5382	0.9288	1.40	0.0030	0.4258	1.08	0.4507	0.7848	0.91	0.3785	0.9132	0.70	0.0038	0.5338	0.98	0.8795	0.9754	0.93	0.5515	0.8272	1.03	0.7513	0.9988	0.79	0.0149	0.9456
Mesencephalic astrocyte-derived neurotrophic factor	MANF_HUMAN	P55145	MANF	1.11	0.5344	0.8777	0.84	0.2986	0.9130	0.79	0.2130	0.7872	1.26	0.1598	0.6806	1.05	0.7682	0.9701	1.14	0.5314	0.9159	0.99	0.9761	0.9805	1.16	0.4194	0.7212	0.85	0.3391	0.9988	0.95	0.7425	0.9456
Microfibril-associated glycoprotein 4	MFAP4_HUMAN	P55083	MFAP4	0.88	0.4702	0.8774	0.75	0.0838	0.9130	0.79	0.2248	0.7872	1.18	0.3249	0.7388	0.96	0.8355	0.9701	1.24	0.3120	0.9159	1.01	0.9546	0.9754	0.98	0.9312	0.9762	0.82	0.2661	0.9988	1.26	0.1671	0.9456
Microfibrillar-associated protein 5	MFAP5_HUMAN	Q13361	MFAP5	0.85	0.1549	0.8737	0.81	0.0635	0.9130	1.01	0.9356	0.9845	1.03	0.8187	0.9423	0.86	0.2192	0.9132	0.97	0.8441	0.9593	1.04	0.7330	0.9754	0.89	0.3523	0.7212	1.10	0.4150	0.9988	1.01	0.9124	0.9768
Mimecan	MIME_HUMAN	P20774	OGN	0.81	0.3073	0.8774	0.84	0.4226	0.9288	0.74	0.2117	0.7872	1.31	0.1957	0.6806	0.94	0.7936	0.9701	1.26	0.3797	0.9159	1.02	0.9436	0.9754	0.62	0.0393	0.3105	1.00	0.9820	0.9988	1.21	0.3568	0.9456
Multimerin-2	MMRN2_HUMAN	Q9H8L6	MMRN2	1.00	0.9655	0.9865	1.17	0.1779	0.9130	0.92	0.5295	0.8140	0.94	0.5616	0.8572	0.97	0.7662	0.9701	1.20	0.1880	0.9159	0.87	0.2536	0.9754	0.90	0.4113	0.7212	0.99	0.9394	0.9988	0.94	0.6057	0.9456
Neutral alpha-glucosidase AB	GANAB_HUMAN	Q14697	GANAB	1.01	0.9371	0.9865	0.95	0.6941	0.9288	0.96	0.7622	0.9171	0.91	0.4263	0.7848	0.86	0.2443	0.9132	0.97	0.8573	0.9593	0.95	0.7092	0.9754	1.02	0.9052	0.9762	1.10	0.4695	0.9988	1.02	0.8863	0.9768
Neutrophil defensin 1	DEF1_HUMAN	P59665	DEFA1	0.92	0.8149	0.9735	0.65	0.2307	0.9130	0.59	0.2049	0.7872	1.04	0.9071	0.9474	0.72	0.3776	0.9132	1.49	0.3666	0.9159	1.29	0.5111	0.9754	0.84	0.6625	0.9223	0.78	0.5051	0.9988	1.03	0.9417	0.9768
Neutrophil elastase	ELNE_HUMAN	P08246	ELANE	0.96	0.8733	0.9865	1.38	0.2005	0.9130	1.02	0.9590	0.9957	1.00	0.9920	0.9920	0.81	0.4072	0.9132	0.87	0.6644	0.9159	1.20	0.4918	0.9754	0.97	0.9103	0.9762	1.32	0.2814	0.9988	0.96	0.8770	0.9768
Nidogen-1	NID1_HUMAN	P14543	NID1	0.92	0.3160	0.8774	1.14	0.1023	0.9130	1.18	0.0829	0.6812	1.13	0.1229	0.6806	0.91	0.2439	0.9132	0.85	0.1075	0.8916	1.02	0.7815	0.9754	1.00	0.9661	0.9871	0.99	0.8975	0.9988	0.91	0.2407	0.9456
Nidogen-2	NID2_HUMAN	Q14112	NID2	0.99	0.9340	0.9865	1.00	0.9746	0.9746	0.93	0.4513	0.8140	1.19	0.0347	0.6806	1.03	0.7608	0.9701	1.03	0.8157	0.9593	0.98	0.8087	0.9754	0.98	0.8667	0.9699	0.90	0.2514	0.9988	1.03	0.7422	0.9456
Olfactomedin-like protein 1	OLFL1_HUMAN	Q6UWY5	OLFML1	0.83	0.5031	0.8774	0.89	0.6849	0.9288	0.57	0.0780	0.6812	1.04	0.8754	0.9423	1.11	0.7259	0.9701	1.73	0.1043	0.8916	0.91	0.7597	0.9754	0.51	0.0285	0.3105	0.95	0.8469	0.9988	1.40	0.2173	0.9456
Papilin	PPN_HUMAN	O95428	PAPLN	1.01	0.9347	0.9865	1.18	0.3132	0.9130	0.87	0.4545	0.8140	1.24	0.1898	0.6806	1.03	0.8818	0.9713	1.06	0.7773	0.9409	0.97	0.8693	0.9754	0.72	0.0667	0.3554	1.05	0.7840	0.9988	1.12	0.4861	0.9456
Periostin	POSTN_HUMAN	Q15063	POSTN	1.23	0.3778	0.8774	0.69	0.1104	0.9130	0.67	0.1323	0.7348	1.04	0.8635	0.9423	1.01	0.9767	0.9901	1.29	0.3679	0.9159	0.98	0.9496	0.9754	0.76	0.3004	0.6831	1.05	0.8478	0.9988	1.27	0.3052	0.9456
Peroxiredoxin-1	PRDX1_HUMAN	Q06830	PRDX1	0.92	0.3885	0.8774	0.97	0.7443	0.9404	0.98	0.8782	0.9474	1.07	0.4466	0.7848	0.91	0.3500	0.9132	1.01	0.9552	0.9905	0.99	0.9470	0.9754	0.90	0.3133	0.6992	0.94	0.5242	0.9988	1.04	0.6829	0.9456
Peroxiredoxin-2	PRDX2_HUMAN	P32119	PRDX2	1.17	0.1979	0.8774	1.02	0.8847	0.9532	0.83	0.1963	0.7872	1.03	0.8138	0.9423	1.05	0.6904	0.9701	1.18	0.2806	0.9159	0.99	0.9387	0.9754	1.03	0.8159	0.9422	0.94	0.6125	0.9988	1.15	0.2521	0.9456
Platelet glycoprotein 4	CD36_HUMAN	P16671	CD36	0.93	0.7710	0.9621	0.88	0.6188	0.9288	1.19	0.5553	0.8140	0.70	0.1587	0.6806	0.77	0.3331	0.9132	0.83	0.5495	0.9159	1.19	0.5227	0.9754	1.10	0.7522	0.9223	0.85	0.5262	0.9988	0.78	0.3360	0.9456
Plexin-B2	PLXB2_HUMAN	O15031	PLXNB2	1.02	0.8915	0.9865	1.12	0.4524	0.9288	0.99	0.9614	0.9957	1.38	0.0295	0.6806	0.92	0.5924	0.9701	0.92	0.6590	0.9159	1.10	0.5685	0.9754	0.86	0.3710	0.7212	0.89	0.4522	0.9988	0.95	0.7354	0.9456
Procathepsin L	CATL1_HUMAN	P07711	CTSL	1.09	0.6152	0.8865	0.99	0.9310	0.9746	0.81	0.2847	0.7872	1.63	0.0029	0.4074	1.03	0.8677	0.9701	1.19	0.3964	0.9159	1.03	0.8727	0.9754	0.95	0.8072	0.9406	1.03	0.8676	0.9988	0.93	0.6569	0.9456
Prolargin	PRELP_HUMAN	P51888	PRELP	0.99	0.9655	0.9865	0.83	0.4460	0.9288	0.59	0.0600	0.6812	1.34	0.2298	0.6894	1.07	0.8003	0.9701	1.44	0.2281	0.9159	0.99	0.9805	0.9805	0.55	0.0259	0.3105	0.96	0.8638	0.9988	1.34	0.2332	0.9456
Prosaposin	SAP_HUMAN	P07602	PSAP	0.93	0.6819	0.9246	0.77	0.1655	0.9130	0.63	0.0283	0.6812	1.21	0.2955	0.7212	0.96	0.8504	0.9701	1.59	0.0386	0.8916	1.07	0.7329	0.9754	1.23	0.3174	0.6992	0.74	0.1062	0.9988	0.92	0.6306	0.9456
Prostaglandin-H2 D-isomerase	PTGDS_HUMAN	P41222	PTGDS	1.09	0.3311	0.8774	1.01	0.9101	0.9694	0.85	0.1224	0.7192	0.94	0.4508	0.7848	1.07	0.4527	0.9386	1.19	0.1074	0.8916	0.90	0.2539	0.9754	1.12	0.2629	0.6503	0.94	0.5038	0.9988	0.99	0.9071	0.9768
Protein AMBP	AMBP_HUMAN	P02760	AMBP	0.53	0.0010	0.1449	1.16	0.4659	0.9288	1.00	0.9874	0.9988	0.98	0.9056	0.9474	0.66	0.0476	0.9132	0.98	0.9379	0.9905	1.08	0.7198	0.9754	0.88	0.5887	0.8732	0.79	0.2658	0.9988	0.75	0.1545	0.9456
Protein disulfide-isomerase	PDIA1_HUMAN	P07237	P4HB	0.99	0.9504	0.9865	1.16	0.3629	0.9130	0.83	0.3090	0.8140	0.78	0.1068	0.6806	0.85	0.3314	0.9132	1.15	0.4662	0.9159	0.87	0.3895	0.9754	0.98	0.9307	0.9762	1.07	0.6896	0.9988	0.94	0.7005	0.9456
Protein S100-A10	S10AA_HUMAN	P60903	S100A10	0.96	0.8148	0.9735	1.23	0.2312	0.9130	0.79	0.2253	0.7872	1.38	0.0529	0.6806	0.93	0.6940	0.9701	1.22	0.3417	0.9159	0.96	0.8266	0.9754	0.76	0.1545	0.5289	1.00	0.9937	0.9988	1.11	0.5457	0.9456
Protein S100-A13	S10AD_HUMAN	Q99584	S100A13	1.45	0.1077	0.8545	0.79	0.3077	0.9130	0.80	0.4010	0.8140	1.05	0.8470	0.9423	1.40	0.1595	0.9132	1.27	0.4127	0.9159	0.72	0.1805	0.9754	1.54	0.0927	0.3847	0.80	0.3573	0.9988	1.07	0.7595	0.9477
Protein S100-A7	S10A7_HUMAN	P31151	S100A7	0.58	0.0538	0.6326	1.15	0.6270	0.9288	0.74	0.3585	0.8140	1.35	0.2894	0.7212	0.95	0.8610	0.9701	1.34	0.4139	0.9159	1.19	0.5721	0.9754	0.65	0.1821	0.5464	1.36	0.2925	0.9988	0.98	0.9491	0.9768
Protein S100-A9	S10A9_HUMAN	P06702	S100A9	0.64	0.0071	0.5015	1.11	0.5508	0.9288	0.87	0.4887	0.8140	1.19	0.3005	0.7212	0.74	0.0895	0.9132	1.17	0.4553	0.9159	0.92	0.6278	0.9754	0.73	0.0928	0.3847	1.08	0.6788	0.9988	0.93	0.6837	0.9456
Secreted frizzled-related protein 1	SFRP1_HUMAN	Q8N474	SFRP1	0.89	0.4668	0.8774	1.08	0.6251	0.9288	1.14	0.4918	0.8140	0.95	0.7652	0.9382	0.91	0.5612	0.9701	1.03	0.8691	0.9649	0.94	0.7060	0.9754	1.06	0.7658	0.9229	0.75	0.0892	0.9988	1.02	0.8945	0.9768
Serine protease HTRA1	HTRA1_HUMAN	Q92743	HTRA1	1.01	0.9829	0.9971	1.05	0.8257	0.9404	0.80	0.4035	0.8140	1.57	0.0498	0.6806	0.89	0.6329	0.9701	1.03	0.9124	0.9855	1.15	0.5810	0.9754	0.73	0.2252	0.6227	0.97	0.8969	0.9988	1.15	0.5412	0.9456
Serpin B6	SPB6_HUMAN	P35237	SERPINB6	0.73	0.0466	0.6326	0.94	0.6910	0.9288	1.26	0.2182	0.7872	0.95	0.7285	0.9382	0.87	0.3985	0.9132	0.85	0.4203	0.9159	0.85	0.3299	0.9754	0.82	0.2607	0.6503	1.11	0.5259	0.9988	0.98	0.9237	0.9768
Serpin H1	SERPH_HUMAN	P50454	SERPINH1	1.49	0.0525	0.6326	1.05	0.8084	0.9404	0.79	0.3354	0.8140	1.18	0.4276	0.7848	1.27	0.2766	0.9132	1.22	0.4468	0.9159	0.84	0.4361	0.9754	1.12	0.6169	0.8875	0.86	0.4853	0.9988	1.18	0.4173	0.9456
Serum amyloid P-component	SAMP_HUMAN	P02743	APCS	0.80	0.2507	0.8774	1.07	0.7116	0.9377	0.85	0.4838	0.8140	1.18	0.4011	0.7848	0.75	0.1547	0.9132	1.16	0.5323	0.9159	1.18	0.4180	0.9754	0.76	0.2078	0.5979	1.13	0.5384	0.9988	0.80	0.2557	0.9456
Signal recognition particle 14 kDa protein	SRP14_HUMAN	P37108	SRP14	1.04	0.8345	0.9735	1.22	0.2513	0.9130	0.87	0.4699	0.8140	0.99	0.9519	0.9797	0.90	0.5326	0.9701	1.20	0.3926	0.9159	0.77	0.1409	0.9754	0.93	0.7228	0.9223	0.86	0.3767	0.9988	0.81	0.2035	0.9456
Somatomedin-B and thrombospondin type-1 domain-containing protein	SBSPO_HUMAN	Q8IVN8	SBSPON	0.83	0.3853	0.8774	0.94	0.7614	0.9404	1.25	0.3586	0.8140	1.08	0.7259	0.9382	0.74	0.1719	0.9132	0.86	0.5737	0.9159	1.08	0.7153	0.9754	0.93	0.7726	0.9231	0.75	0.1832	0.9988	0.91	0.6489	0.9456
Target of Nesh-SH3	TARSH_HUMAN	Q7Z7G0	ABI3BP	0.88	0.5612	0.8777	0.76	0.2017	0.9130	0.74	0.2261	0.7872	1.31	0.2116	0.6806	1.00	0.9834	0.9901	1.17	0.5544	0.9159	0.90	0.6444	0.9754	0.66	0.0811	0.3690	1.01	0.9493	0.9988	1.32	0.1927	0.9456
Tenascin	TENA_HUMAN	P24821	TNC	0.87	0.6905	0.9273	1.17	0.6437	0.9288	1.69	0.1771	0.7872	1.41	0.3120	0.7212	0.73	0.3677	0.9132	0.80	0.5937	0.9159	1.06	0.8748	0.9754	1.13	0.7487	0.9223	1.08	0.8213	0.9988	0.89	0.7362	0.9456
Tenascin-X	TENX_HUMAN	P22105	TNXB	1.05	0.6593	0.9050	0.83	0.0774	0.9130	0.92	0.5186	0.8140	1.13	0.2736	0.7212	1.04	0.7079	0.9701	1.08	0.5679	0.9159	0.91	0.4066	0.9754	0.88	0.2779	0.6570	1.02	0.8932	0.9988	1.08	0.4734	0.9456
Thioredoxin	THIO_HUMAN	P10599	TXN	1.11	0.1804	0.8774	0.96	0.6204	0.9288	0.88	0.1487	0.7486	1.02	0.8436	0.9423	1.02	0.7886	0.9701	1.15	0.1468	0.9159	0.94	0.4628	0.9754	1.10	0.2839	0.6570	0.86	0.0474	0.9988	1.03	0.6669	0.9456
Thrombospondin type-1 domain-containing protein 4	THSD4_HUMAN	Q6ZMP0	THSD4	0.88	0.2869	0.8774	0.83	0.1334	0.9130	1.09	0.5455	0.8140	0.94	0.6031	0.8766	0.83	0.1553	0.9132	0.93	0.6378	0.9159	0.86	0.2733	0.9754	0.94	0.6401	0.9117	0.96	0.7363	0.9988	0.89	0.3643	0.9456
Transforming growth factor-beta-induced protein ig-h3	BGH3_HUMAN	Q15582	TGFBI	1.16	0.2865	0.8774	1.01	0.9619	0.9746	0.95	0.7302	0.9081	1.19	0.2112	0.6806	1.07	0.6160	0.9701	0.99	0.9573	0.9905	0.96	0.7934	0.9754	0.67	0.0076	0.2155	1.14	0.3562	0.9988	1.21	0.1674	0.9456
Transgelin-2	TAGL2_HUMAN	P37802	TAGLN2	1.18	0.2430	0.8774	1.02	0.8850	0.9532	0.72	0.0450	0.6812	1.29	0.0773	0.6806	1.29	0.0931	0.9132	1.46	0.0345	0.8916	1.02	0.8911	0.9754	0.82	0.2169	0.6117	1.02	0.9207	0.9988	1.06	0.7100	0.9456
Tryptase beta-2	TRYB2_HUMAN	P20231	TPSB2	0.66	0.0402	0.6326	1.05	0.7964	0.9404	1.49	0.0870	0.6812	1.18	0.4270	0.7848	0.82	0.3408	0.9132	0.66	0.1002	0.8916	1.04	0.8724	0.9754	0.64	0.0500	0.3205	1.11	0.6170	0.9988	1.05	0.8270	0.9768
Tubulointerstitial nephritis antigen-like	TINAL_HUMAN	Q9GZM7	TINAGL1	0.98	0.8423	0.9735	0.97	0.7535	0.9404	0.95	0.6093	0.8423	1.13	0.1800	0.6806	0.98	0.8719	0.9701	1.05	0.6768	0.9159	1.06	0.5782	0.9754	1.06	0.5945	0.8732	0.94	0.5509	0.9988	1.00	0.9988	0.9988
Versican core protein	CSPG2_HUMAN	P13611	VCAN	0.87	0.5703	0.8777	0.74	0.2210	0.9130	0.84	0.5462	0.8140	1.16	0.5340	0.8274	0.88	0.6230	0.9701	0.98	0.9334	0.9905	0.86	0.5437	0.9754	0.53	0.0164	0.2897	1.10	0.7075	0.9988	1.32	0.2472	0.9456
Vinculin	VINC_HUMAN	P18206	VCL	1.17	0.1333	0.8545	0.98	0.8592	0.9532	0.88	0.2998	0.8129	0.87	0.1860	0.6806	1.07	0.5057	0.9701	1.12	0.3657	0.9159	0.93	0.5238	0.9754	0.99	0.9554	0.9833	0.94	0.5781	0.9988	1.10	0.3355	0.9456
Vitronectin	VTNC_HUMAN	P04004	VTN	0.89	0.4857	0.8774	1.04	0.8198	0.9404	0.81	0.2593	0.7872	1.30	0.1054	0.6806	0.91	0.5801	0.9701	1.15	0.4971	0.9159	1.15	0.4212	0.9754	0.69	0.0390	0.3105	0.94	0.6952	0.9988	0.97	0.8565	0.9768
von Willebrand factor A domain-containing protein 1	VWA1_HUMAN	Q6PCB0	VWA1	1.20	0.0966	0.8545	1.23	0.0592	0.9130	0.98	0.8478	0.9345	1.15	0.1905	0.6806	1.15	0.2124	0.9132	0.99	0.9576	0.9905	0.92	0.4781	0.9754	0.84	0.1575	0.5289	1.03	0.8037	0.9988	1.15	0.2008	0.9456
Y-box-binding protein 1	YBOX1_HUMAN	P67809	YBX1	1.06	0.5495	0.8777	0.96	0.6233	0.9288	1.09	0.3877	0.8140	1.12	0.2156	0.6806	1.03	0.7710	0.9701	0.92	0.4719	0.9159	0.91	0.3115	0.9754	1.07	0.5161	0.8177	0.92	0.3471	0.9988	0.90	0.2246	0.9456

Supplemental Table VII. Quantification of proteins identified by proteomics in the SDS extracts of the human ischaemic heart failure cohort according to medication usage and presence of comorbidities.

Protein Information			Statins			Diabetes			ACE Inhibitors			Antiarrhythmics			Anticoagulants			ARBs			Atrial Fibrilation			Beta Blockers			Hypertension			Antiplatelet Therapy		
			yes=29 vs no=36			yes=27 vs no=38			yes=49 vs no=16			yes=28 vs no=37			yes=41 vs no=24			yes=13 vs no=52			yes=23 vs no=42			yes=44 vs no=21			yes=42 vs no=23			yes=29 vs no=36		
Protein Name	Accession number	Uniprot ID	FC	P-value	BH Adjusted Pvalue	FC	P-value	BH Adjusted Pvalue	FC	P-value	BH Adjusted Pvalue	FC	P-value	BH Adjusted Pvalue	FC	P-value	BH Adjusted Pvalue	FC	P-value	BH Adjusted Pvalue	FC	P-value	BH Adjusted Pvalue	FC	P-value	BH Adjusted Pvalue	FC	P-value	BH Adjusted Pvalue	FC	P-value	BH Adjusted Pvalue
[F-actin]-monooxygenase MICAL3	MICA3_HUMAN	Q7RTP6	1.12	0.1283	0.3940	1.04	0.5897	0.9982	0.94	0.4781	0.9960	1.00	0.9772	0.9961	1.01	0.9284	0.9951	1.01	0.9218	0.9959	0.91	0.2497	0.7187	1.10	0.2564	0.5217	1.07	0.4096	0.9889	1.06	0.4420	0.9607
[Protein ADP-ribosylarginine] hydrolase-like protein 1	ARHL1_HUMAN	Q8NDY3	1.11	0.2183	0.4911	0.98	0.8614	0.9982	0.95	0.6502	0.9960	0.99	0.8886	0.9939	0.98	0.8463	0.9951	1.07	0.5343	0.9856	0.86	0.0962	0.6824	1.26	0.0151	0.1606	0.99	0.8834	0.9889	1.02	0.7816	0.9881
[Pyruvate dehydrogenase (acetyl-transferring)] kinase isozyme 1, mitocho	PDK1_HUMAN	Q15118	1.06	0.4847	0.7201	1.19	0.0243	0.9982	0.98	0.8708	0.9960	1.06	0.4670	0.9939	1.01	0.9466	0.9951	0.94	0.5663	0.9856	1.00	0.9986	0.9992	0.92	0.3662	0.5972	1.11	0.1870	0.9889	0.98	0.8434	0.9881
[Pyruvate dehydrogenase [acetyl-transferring]]-phosphatase 1, mitochonc	PDP1_HUMAN	Q9P0J1	1.08	0.2102	0.4843	1.08	0.1897	0.9982	0.95	0.4519	0.9960	0.99	0.8160	0.9939	1.03	0.6771	0.9951	1.04	0.5590	0.9856	1.01	0.9316	0.9901	1.00	0.9477	0.9708	1.10	0.1303	0.9889	1.05	0.4272	0.9607
1,4-alpha-glucan-branching enzyme	GLGB_HUMAN	Q04446	1.18	0.1363	0.3997	0.93	0.5397	0.9982	1.02	0.8864	0.9960	0.92	0.4427	0.9939	0.99	0.9236	0.9951	1.11	0.4445	0.9856	0.82	0.0830	0.6824	1.42	0.0032	0.1090	0.93	0.4893	0.9889	1.00	0.9864	0.9992
10 kDa heat shock protein, mitochondrial	CH10_HUMAN	P61604	1.09	0.1632	0.4357	1.00	0.9808	0.9985	0.99	0.9419	0.9960	1.02	0.7066	0.9939	0.99	0.9222	0.9951	1.02	0.8181	0.9959	0.99	0.9355	0.9903	1.05	0.4544	0.6687	1.00	0.9609	0.9961	0.99	0.8373	0.9881
116 kDa U5 small nuclear ribonucleoprotein component	U5S1_HUMAN	Q15029	0.94	0.2350	0.5075	1.02	0.7429	0.9982	1.07	0.2612	0.9960	0.98	0.6349	0.9939	0.98	0.6795	0.9951	0.94	0.3018	0.9856	1.05	0.3571	0.7644	0.93	0.2223	0.4903	0.96	0.4343	0.9889	1.01	0.9134	0.9938
14 kDa phosphohistidine phosphatase	PHP14_HUMAN	Q9NRX4	1.14	0.2514	0.5219	0.99	0.9141	0.9982	0.97	0.7980	0.9960	0.93	0.5292	0.9939	1.00	0.9663	0.9951	1.18	0.2298	0.9856	0.80	0.0568	0.6824	1.36	0.0139	0.1581	0.95	0.6865	0.9889	1.03	0.8197	0.9881
14-3-3 protein beta/alpha	1433B_HUMAN	P31946	0.98	0.8131	0.9114	0.94	0.3659	0.9982	1.13	0.1249	0.9960	0.90	0.1231	0.9874	0.87	0.0512	0.9951	0.95	0.5609	0.9856	0.97	0.6376	0.8888	1.03	0.6830	0.8225	0.93	0.2872	0.9889	0.85	0.0143	0.9304
14-3-3 protein epsilon	1433E_HUMAN	P62258	1.09	0.2696	0.5388	1.00	0.9954	0.9985	1.04	0.7004	0.9960	0.92	0.2506	0.9939	0.93	0.3880	0.9951	0.99	0.9090	0.9959	0.82	0.0169	0.6779	1.18	0.0476	0.2309	0.93	0.3673	0.9889	0.94	0.3945	0.9607
14-3-3 protein eta	1433F_HUMAN	Q04917	1.12	0.0484	0.2898	1.00	0.9487	0.9982	1.03	0.7225	0.9960	1.01	0.8189	0.9939	1.06	0.3295	0.9951	1.01	0.8454	0.9959	0.92	0.2046	0.7149	1.04	0.5456	0.7330	1.00	0.9810	0.9976	1.08	0.2048	0.9607
14-3-3 protein gamma	1433G_HUMAN	P61981	1.09	0.3048	0.5744	0.97	0.7328	0.9982	0.99	0.9325	0.9960	0.93	0.3873	0.9939	0.95	0.5173	0.9951	1.02	0.8068	0.9959	0.80	0.0059	0.6779	1.23	0.0200	0.1655	0.89	0.1657	0.9889	0.93	0.3688	0.9607
14-3-3 protein theta	1433T_HUMAN	P27348	0.94	0.3504	0.6124	1.01	0.9046	0.9982	1.11	0.1387	0.9960	0.91	0.1231	0.9874	0.88	0.0491	0.9951	0.91	0.1844	0.9856	0.93	0.2505	0.7187	0.99	0.8263	0.9067	0.92	0.1722	0.9889	0.86	0.0133	0.9304
14-3-3 protein zeta/delta	1433Z_HUMAN	P63104	1.07	0.3069	0.5769	1.01	0.9158	0.9982	1.07	0.4362	0.9960	0.89	0.1000	0.9874	0.93	0.2963	0.9951	0.99	0.9105	0.9959	0.89	0.1260	0.6868	1.08	0.3141	0.5582	0.91	0.1785	0.9889	0.92	0.2540	0.9607
2-iminobutanoate/2-iminopropanoate deaminase	RIDA_HUMAN	P52758	1.17	0.0891	0.3410	1.15	0.1420	0.9982	0.93	0.5135	0.9960	0.99	0.9247	0.9939	0.99	0.8855	0.9951	1.05	0.6827	0.9856	0.93	0.4860	0.8365	1.04	0.7401	0.8574	0.99	0.9269	0.9889	1.00	0.9774	0.9992
2-methoxy-6-polyprenyl-1,4-benzoquinol methylase, mitochondrial	COQ5_HUMAN	Q5HYK3	1.15	0.0804	0.3290	1.06	0.4360	0.9982	0.96	0.6889	0.9960	1.09	0.2921	0.9939	1.07	0.4273	0.9951	0.99	0.9066	0.9959	1.00	0.9724	0.9937	1.08	0.3686	0.5979	1.07	0.3921	0.9889	1.16	0.0628	0.9607
2-oxoglutarate dehydrogenase-like, mitochondrial	OGDHL_HUMAN	Q9ULD0	1.08	0.6074	0.7989	1.07	0.6572	0.9982	1.28	0.1363	0.9960	1.25	0.1234	0.9874	0.89	0.4521	0.9951	0.79	0.1978	0.9856	1.33	0.0656	0.6824	1.01	0.9277	0.9585	1.05	0.7252	0.9889	0.87	0.3260	0.9607
2-oxoglutarate dehydrogenase, mitochondrial	ODO1_HUMAN	Q02218	1.18	0.0136	0.2710	1.06	0.3612	0.9982	1.02	0.8120	0.9960	1.03	0.6244	0.9939	1.06	0.4219	0.9951	0.96	0.6088	0.9856	0.93	0.3233	0.7400	1.07	0.3441	0.5780	1.09	0.2259	0.9889	1.08	0.2276	0.9607
2-oxoisovalerate dehydrogenase subunit alpha, mitochondrial	ODBA_HUMAN	P12694	1.18	0.0335	0.2873	1.04	0.6591	0.9982	1.05	0.6287	0.9960	1.01	0.8558	0.9939	1.07	0.4052	0.9951	0.91	0.3157	0.9856	0.95	0.5581	0.8601	1.03	0.7778	0.8813	1.12	0.1731	0.9889	1.13	0.1274	0.9607
2-oxoisovalerate dehydrogenase subunit beta, mitochondrial	ODBB_HUMAN	P21953	1.06	0.5095	0.7292	1.05	0.5360	0.9982	1.16	0.1213	0.9960	1.00	0.9594	0.9942	1.07	0.4116	0.9951	0.84	0.0924	0.9856	0.93	0.4262	0.8072	0.97	0.7624	0.8704	1.10	0.2578	0.9889	1.04	0.6729	0.9881
2,4-dienoyl-CoA reductase, mitochondrial	DECR_HUMAN	Q16698	1.25	0.0122	0.2656	1.03	0.7334	0.9982	0.89	0.2766	0.9960	0.97	0.7011	0.9939	1.05	0.5790	0.9951	1.16	0.1947	0.9856	0.91	0.3299	0.7411	1.23	0.0436	0.2215	0.97	0.7795	0.9889	1.04	0.6507	0.9881
2',3'-cyclic-nucleotide 3'-phosphodiesterase	CN37_HUMAN	P09543	0.87	0.0601	0.3008	0.96	0.5453	0.9982	0.99	0.8761	0.9960	1.02	0.7815	0.9939	0.92	0.2603	0.9951	1.15	0.1271	0.9856	1.00	0.9524	0.9923	1.13	0.1428	0.3873	1.08	0.3284	0.9889	0.99	0.8942	0.9938
26S proteasome non-ATPase regulatory subunit 1	PSMD1_HUMAN	Q99460	0.97	0.5870	0.7845	1.00	0.9341	0.9982	1.06	0.3655	0.9960	1.00	0.9261	0.9939	0.95	0.3207	0.9951	0.94	0.3573	0.9856	0.93	0.1732	0.7012	1.12	0.0556	0.2468	0.96	0.4457	0.9889	0.96	0.4458	0.9607
26S proteasome non-ATPase regulatory subunit 11	PSD11_HUMAN	O00231	1.02	0.6956	0.8548	1.07	0.2854	0.9982	1.09	0.2112	0.9960	1.12	0.0623	0.9874	1.05	0.4082	0.9951	0.89	0.0988	0.9856	1.10	0.1396	0.6871	0.94	0.3430	0.5778	0.99	0.8270	0.9889	1.00	0.9814	0.9992
26S proteasome non-ATPase regulatory subunit 12	PSD12_HUMAN	O00232	0.99	0.8231	0.9186	1.05	0.3833	0.9982	1.04	0.5398	0.9960	1.07	0.2136	0.9939	0.99	0.9266	0.9951	0.99	0.8549	0.9959	0.98	0.7642	0.9289	1.03	0.6125	0.7814	1.02	0.7850	0.9889	0.99	0.8431	0.9881
26S proteasome non-ATPase regulatory subunit 13	PSD13_HUMAN	Q9UNM6	1.02	0.7180	0.8715	1.06	0.2860	0.9982	1.11	0.0962	0.9960	1.11	0.0568	0.9874	0.98	0.7896	0.9951	0.86	0.0324	0.9411	0.98	0.6769	0.8962	0.95	0.4040	0.6299	0.96	0.4549	0.9889	0.98	0.7092	0.9881
26S proteasome non-ATPase regulatory subunit 14	PSDE_HUMAN	O00487	0.95	0.4053	0.6612	1.07	0.2613	0.9982	1.10	0.1859	0.9960	1.10	0.1044	0.9874	1.02	0.7382	0.9951	0.87	0.0654	0.9641	1.05	0.4164	0.7974	0.91	0.1535	0.4038	1.03	0.6539	0.9889	0.97	0.5927	0.9881
26S proteasome non-ATPase regulatory subunit 2	PSMD2_HUMAN	Q13200	1.03	0.6121	0.8022	0.98	0.7306	0.9982	1.07	0.2902	0.9960	1.03	0.6496	0.9939	0.98	0.7932	0.9951	0.94	0.4045	0.9856	0.92	0.1622	0.6896	1.14	0.0350	0.2017	1.04	0.4962	0.9889	1.01	0.8144	0.9881
26S proteasome non-ATPase regulatory subunit 3	PSMD3_HUMAN	O43242	1.04	0.5187	0.7355	1.01	0.8894	0.9982	1.15	0.0771	0.9960	1.00	0.9597	0.9942	0.99	0.8356	0.9951	0.85	0.0415	0.9411	0.95	0.4632	0.8244	1.10	0.1995	0.4593	0.95	0.4173	0.9889	1.02	0.7419	0.9881
26S proteasome non-ATPase regulatory subunit 4	PSMD4_HUMAN	P55036	0.94	0.2056	0.4823	1.00	0.9288	0.9982	1.10	0.0947	0.9960	1.10	0.0601	0.9874	0.98	0.7209	0.9951	0.91	0.1465	0.9856	1.03	0.5786	0.8632	1.02	0.6940	0.8265	1.02	0.7622	0.9889	0.98	0.7307	0.9881
26S proteasome non-ATPase regulatory subunit 6	PSMD6_HUMAN	Q15008	1.21	0.0411	0.2873	1.10	0.3233	0.9982	1.20	0.0885	0.9960	1.11	0.2863	0.9939	1.19	0.0690	0.9951	0.83	0.1059	0.9856	1.08	0.4255	0.8072	0.97	0.7463	0.8613	1.03	0.7759	0.9889	1.10	0.3156	0.9607
26S proteasome non-ATPase regulatory subunit 7	PSMD7_HUMAN	P51665	0.97	0.6086	0.7996	1.02	0.6706	0.9982	1.06	0.3710	0.9960	1.11	0.0555	0.9874	0.98	0.6936	0.9951	0.97	0.5981	0.9856	1.04	0.4890	0.8385	1.03	0.6177	0.7841	1.00	0.9882	0.9976	0.99	0.8199	0.9881
26S proteasome non-ATPase regulatory subunit 8	PSMD8_HUMAN	P48556	0.96	0.4956	0.7237	1.03	0.6054	0.9982	1.09	0.2432	0.9960	1.07	0.2373	0.9939	1.01	0.8526	0.9951	0.89	0.1401	0.9856	0.98	0.7218	0.9201	0.95	0.4464	0.6635	1.02	0.7164	0.9889	1.01	0.8293	0.9881
26S proteasome regulatory subunit 10B	PRS10_HUMAN	P62333	1.03	0.6548	0.8297	1.01	0.8539	0.9982	1.07	0.2824	0.9960	1.11	0.0616	0.9874	1.03	0.5607	0.9951	0.96	0.6000	0.9856	1.00	0.9542	0.9923	0.99	0.9213	0.9569	1.06	0.3217	0.9889	1.07	0.2447	0.9607
26S proteasome regulatory subunit 4	PRS4_HUMAN	P62191	0.98	0.7480	0.8824	1.02	0.7640	0.9982	1.01	0.8909	0.9960	1.05	0.3321	0.9939	0.99	0.9282	0.9951	1.01	0.9289	0.9959	0.92	0.1618	0.6896	1.08	0.2036	0.4651	1.05	0.3754	0.9889	1.03	0.5359	0.9881
26S proteasome regulatory subunit 6A	PRS6A_HUMAN	P17980	0.97	0.5242	0.7378	0.97	0.5293	0.9982	1.05	0.3146	0.9960	1.04	0.3816	0.9939	1.00	0.9732	0.9951	0.96	0.4931	0.9856	0.99	0.8194	0.9481	1.04	0.4725	0.6780	1.03	0.5914	0.9889	1.01	0.7429	0.9881
26S proteasome regulatory subunit 6B	PRS6B_HUMAN	P43686	1.03	0.6328	0.8170	1.01	0.8774	0.9982	1.06	0.3989	0.9960	1.12	0.0464	0.9874	1.10	0.1285	0.9951	0.97	0.7092	0.9857	1.03	0.6559	0.8915	0.95	0.4477	0.6635	1.01	0.8388	0.9889	1.10	0.0977	0.9607
26S proteasome regulatory subunit 7	PRS7_HUMAN	P35998	0.98	0.6499	0.8297	1.04	0.4554	0.9982	1.06	0.3693	0.9960	1.06	0.2784	0.9939	1.00	0.9447	0.9951	0.96	0.5326	0.9856	0.97	0.6458	0.8897	0.99	0.8740	0.9308	1.06	0.2935	0.9889	1.02	0.6505	0.9881
26S proteasome regulatory subunit 8	PRS8_HUMAN	P62195	0.99	0.8829	0.9389	1.01	0.9082	0.9982	1.04	0.5520	0.9960	1.06	0.2883	0.9939	1.02	0.7449	0.9951	0.96	0.5883	0.9856	0.96	0.5609	0.8601	1.06	0.3691	0.5979	1.05	0.4074	0.9889	1.01	0.8551	0.9881
28 kDa heat- and acid-stable phosphoprotein	HAP28_HUMAN	Q13442	0.93	0.5577	0.7603	0.90	0.3583	0.9982	1.04	0.7979	0.9960	0.86	0.1959	0.9939	0.88	0.3124	0.9951	1.01	0.9595	0.9967	0.86	0.2258	0.7187	1.43	0.0063	0.1323	0.93	0.5763	0.9889	0.88	0.2674	0.9607
28S ribosomal protein S11, mitochondrial	RT11_HUMAN	P82912	1.01	0.9188	0.9543	1.02	0.8070	0.9982	0.95	0.4907	0.9960	0.89	0.0759	0.9874	0.98	0.7634	0.9951	1.08	0.3717	0.9856	1.05	0.4714	0.8308	0.99	0.9116	0.9526	1.20	0.0054	0.9889	1.07	0.2863	0.9607
28S ribosomal protein S22, mitochondrial	RT22_HUMAN	P82650	0.99	0.8384	0.9204	1.00	0.9500	0.9982	0.98	0.7217	0.9960	1.03	0.5536	0.9939	1.03	0.5500	0.9951	0.97	0.6533	0.9856	0.98	0.7547	0.9282	1.04	0.5524	0.7377	1.12	0.0411	0.9889	1.07	0.2178	0.9607
28S ribosomal protein S23, mitochondrial	RT23_HUMAN	Q9Y3D9	1.15	0.0123	0.2656	1.05	0.4433	0.9982	0.91	0.1773	0.9960	1.05	0.4069	0.9939	1.10	0.1024	0.9951	1.05	0.5063	0.9856	0.93	0.2655	0.7187	1.06	0.3801	0.6113	1.08	0.1823	0.9889	1.13	0.0266	0.9607
28S ribosomal protein S26, mitochondrial	RT26_HUMAN	Q9BYN8	1.00	0.9986	0.9993	1.02	0.7079	0.9982	0.96	0.5813	0.9960	0.94	0.2973	0.9939	1.01	0.8532	0.9951	1.00	0.9603	0.9967	1.00	0.9528	0.9923	0.95	0.4820	0.6855	1.07	0.2628	0.9889	1.07	0.2301	0.9607
28S ribosomal protein S27, mitochondrial	RT27_HUMAN	Q92552	1.15	0.0415	0.2873	1.02	0.8084	0.9982	0.99	0.9433	0.9960	1.01	0.8296	0.9939	1.08	0.2834	0.9951	0.97	0.6738	0.9856	0.97	0.6303	0.8874	0.96	0.6295	0.7896	1.10	0.1802	0.9889	1.10	0.1642	0.9607
28S ribosomal protein S29, mitochondrial	RT29_HUMAN	P51398	1.04	0.6590	0.8336	1.05	0.5744	0.9982	0.99	0.8787	0.9960	0.89	0.1376	0.9874	0.94	0.4419	0.9951	0.96	0.6395	0.9856	0.93	0.4074	0.7885	1.10	0.2819	0.5346	1.14	0.1049	0.9889	1.04	0.5738	0.9881
28S ribosomal protein S31, mitochondrial	RT31_HUMAN	Q92665	0.99	0.8803	0.9384	1.07	0.2644	0.9982	1.00	0.9690	0.9960	0.91	0.1107	0.9874	0.93	0.2530	0.9951	0.97	0.6605	0.9856	1.07	0.3234	0.7400	1.00	0.9754	0.9860	1.10	0.1310	0.9889	0.99	0.9014	0.9938
28S ribosomal protein S36, mitochondrial	RT36_HUMAN	P82909	1.06	0.2664	0.5351	0.98	0.6907	0.9982	1.04	0.5074	0.9960	1.13	0.0257	0.9874	1.07	0.2334	0.9951	0.90	0.1052	0.9856	1.05	0.4241	0.8063	0.96	0.5382	0.7261	1.14	0.0162	0.9889	1.07	0.2125	0.9607
3-hydroxyacyl-CoA dehydrogenase type-2	HCD2_HUMAN	Q99714	1.01	0.8435	0.9230	1.05	0.3413	0.9982	1.03	0.5557	0.9960	0.99	0.8377	0.9939	0.98	0.6226	0.9951	0.95	0.3981	0.9856	1.02	0.6322	0.8882	0.91	0.0663	0.2648	1.06	0.2487	0.9889	0.97	0.5203	0.9846
3-hydroxyisobutyrate dehydrogenase, mitochondrial	3HIDH_HUMAN	P31937	1.18	0.0323	0.2873	1.06	0.4521	0.9982	0.89	0.2151	0.9960	0.98	0.7937	0.9939	1.01	0.9098	0.9951	1.13	0.2061	0.9856	0.89	0.1627	0.6896	1.09	0.3486	0.5817	1.03	0.7057	0.9889	1.02	0.7706	0.9881
3-hydroxyisobutyryl-CoA hydrolase, mitochondrial	HIBCH_HUMAN	Q6NVY1	1.14	0.1456	0.4103	0.91	0.3165	0.9982	0.94	0.5617	0.9960	0.90	0.2524	0.9939	0.98	0.8699	0.9951	1.03	0.7757	0.9875	0.91	0.3007	0.7349	1.18	0.0941	0.3118	0.90	0.2340	0.9889	0.98	0.7908	0.9881
3-ketoacyl-CoA thiolase, mitochondrial	THIM_HUMAN	P42765	1.19	0.0250	0.2873	1.03	0.6630	0.9982	0.93	0.4537	0.9960	1.13	0.1233	0.9874	1.07	0.3819	0.9951	1.08	0.4184	0.9856	0.99	0.8689	0.9677	1.10	0.2780	0.5337	1.01	0.9135	0.9889	0.98	0.8302	0.9881
3-ketoacyl-CoA thiolase, peroxisomal	THIK_HUMAN	P09110	1.00	0.9894	0.9926	0.94	0.2047	0.9982	0.92	0.1527	0.9960	1.04	0.4422	0.9939	1.03	0.5509	0.9951	1.04	0.5158	0.9856	1.03	0.5578	0.8601	1.00	0.9588	0.9773	1.00	0.9431	0.9922	1.00	0.9595	0.9946
3-ketodihydrosphingosine reductase	KDSR_HUMAN	Q06136	0.92	0.1448	0.4093	0.90	0.0756	0.9982	0.98	0.8216	0.9960	0.97	0.6309	0.9939	0.93	0.2177	0.9951	0.99	0.9168	0.9959	1.04	0.5392	0.8573	0.99	0.8973	0.9431	0.98	0.7374	0.9889	0.93	0.2188	0.9607
3-mercaptopyruvate sulfurtransferase	THTM_HUMAN	P25325	1.32	0.0003	0.1653	1.09	0.2594	0.9982	0.97	0.7142	0.9960	1.02	0.7592	0.9939	1.21	0.0206	0.9951	1.05	0.6458	0.9856	0.97	0.7388	0.9218	0.95	0.5919	0.7646	1.00	0.9584	0.9961	1.17	0.0443	0.9607
3-oxoacyl-[acyl-carrier-protein] synthase, mitochondrial	OXSM_HUMAN	Q9NWU1	1.09	0.2309	0.5050	0.92	0.2832	0.9982	0.88	0.1411	0.9960	0.88	0.0685	0.9874	1.00	0.9853	0.9964	1.15	0.1291	0.9856	0.99	0.8505	0.9579	1.09	0.2845	0.5383	1.06	0.4271	0.9889	0.97	0.6693	0.9881
3'(2'),5'-bisphosphate nucleotidase 1	BPNT1_HUMAN	O95861	1.22	0.0207	0.2873	1.03	0.7530	0.9982	0.96	0.7064	0.9960	0.96	0.6167	0.9939	1.07	0.4777	0.9951	1.07	0.5168	0.9856	0.81	0.0213	0.6804	1.22	0.0431	0.2209	0.95	0.5642	0.9889	1.09	0.3285	0.9607
39S ribosomal protein L1, mitochondrial	RM01_HUMAN	Q9BYD6	1.06	0.4864	0.7205	1.01	0.9309	0.9982	0.89	0.2417	0.9960	0.92	0.3126	0.9939	0.96	0.6603	0.9951	1.09	0.4149	0.9856	0.84	0.0440	0.6824	1.06	0.5422	0.7297	1.01	0.9228	0.9889	1.04	0.6581	0.9881
39S ribosomal protein L11, mitochondrial	RM11_HUMAN	Q9Y3B7	1.14	0.0293	0.2873	1.05	0.4196	0.9982	1.01	0.9181	0.9960	0.97	0.6523	0.9939	1.03	0.6102	0.9951	0.95	0.4471	0.9856	0.94	0.2953	0.7286	1.07	0.3285	0.5681	1.04	0.5372	0.9889	1.04	0.5029	0.9818
39S ribosomal protein L12, mitochondrial	RM12_HUMAN	P52815	1.02	0.7112	0.8664	0.95	0.4049	0.9982	0.94	0.3529	0.9960	0.91	0.0989	0.9874	0.86	0.0176	0.9951	1.03	0.6730	0.9856	0.90	0.0855	0.6824	1.12	0.0881	0.2995	1.08	0.2459	0.9889	0.94	0.2920	0.9607
39S ribosomal protein L17, mitochondrial	RM17_HUMAN	Q9NRX2	1.00	0.9954	0.9973	1.05	0.3631	0.9982	0.95	0.4670	0.9960	1.01	0.7968	0.9939	0.95	0.4131	0.9951	1.03	0.6323	0.9856	0.97	0.6130	0.8738	0.96	0.5023	0.7008	1.08	0.2072	0.9889	0.98	0.6857	0.9881
39S ribosomal protein L38, mitochondrial	RM38_HUMAN	Q96DV4	1.04	0.5195	0.7355	1.03	0.6271	0.9982	0.92	0.1740	0.9960	0.93	0.1843	0.9939	0.99	0.8879	0.9951	1.03	0.6358	0.9856	0.93	0.2199	0.7187	1.07	0.2949	0.5464	1.07	0.2145	0.9889	1.05	0.3535	0.9607
39S ribosomal protein L44, mitochondrial	RM44_HUMAN	Q9H9J2	1.16	0.0242	0.2873	0.98	0.7951	0.9982	0.98	0.8142	0.9960	0.95	0.4690	0.9939	1.10	0.1872	0.9951	0.99	0.8597	0.9959	0.96	0.5908	0.8646	1.01	0.9358	0.9616	1.03	0.6334	0.9889	1.13	0.0575	0.9607
39S ribosomal protein L46, mitochondrial	RM46_HUMAN	Q9H2W6	1.13	0.0955	0.3548	1.05	0.5293	0.9982	0.88	0.1485	0.9960	0.87	0.0638	0.9874	0.92	0.2681	0.9951	1.03	0.7065	0.9857	0.88	0.0992	0.6824	0.98	0.7875	0.8860	1.09	0.2573	0.9889	0.97	0.6337	0.9881
39S ribosomal protein L53, mitochondrial	RM53_HUMAN	Q96EL3	1.06	0.3305	0.5968	1.05	0.4801	0.9982	0.93	0.3232	0.9960	0.94	0.3277	0.9939	0.94	0.3790	0.9951	1.03	0.7057	0.9857	0.97	0.6348	0.8887	1.11	0.1475	0.3939	1.01	0.9092	0.9889	1.02	0.7786	0.9881
4-aminobutyrate aminotransferase, mitochondrial	GABT_HUMAN	P80404	1.08	0.6232	0.8128	1.16	0.3439	0.9982	1.12	0.5175	0.9960	0.93	0.6165	0.9939	0.93	0.6472	0.9951	0.92	0.6571	0.9856	0.81	0.1942	0.7149	0.93	0.6842	0.8231	1.34	0.0637	0.9889	1.07	0.6631	0.9881
4-trimethylaminobutyraldehyde dehydrogenase	AL9A1_HUMAN	P49189	1.05	0.4536	0.6965	0.97	0.6448	0.9982	0.96	0.6369	0.9960	0.93	0.3105	0.9939	0.93	0.2872	0.9951	1.03	0.7395	0.9857	0.88	0.0698	0.6824	1.02	0.8087	0.8963	1.00	0.9443	0.9922	1.00	0.9481	0.9938
40S ribosomal protein S10	RS10_HUMAN	P46783	0.92	0.1664	0.4371	1.02	0.7231	0.9982	0.97	0.6871	0.9960	1.06	0.3654	0.9939	0.98	0.8104	0.9951	1.03	0.7548	0.9857	1.20	0.0063	0.6779	0.96	0.5703	0.7499	0.92	0.1946	0.9889	0.92	0.1713	0.9607
40S ribosomal protein S12	RS12_HUMAN	P25398	0.86	0.0193	0.2873	0.98	0.8176	0.9982	1.07	0.3657	0.9960	1.01	0.8649	0.9939	0.94	0.4012	0.9951	0.96	0.6386	0.9856	1.13	0.0981	0.6824	0.97	0.7333	0.8540	0.92	0.2339	0.9889	0.99	0.8785	0.9938
40S ribosomal protein S13	RS13_HUMAN	P62277	0.96	0.5118	0.7292	0.95	0.4779	0.9982	1.01	0.9078	0.9960	1.02	0.8173	0.9939	1.07	0.3082	0.9951	0.97	0.6753	0.9856	1.11	0.1233	0.6868	0.85	0.0250	0.1799	0.89	0.0777	0.9889	1.03	0.6859	0.9881
40S ribosomal protein S17	RS17_HUMAN	P08708	0.85	0.0721	0.3224	0.96	0.6895	0.9982	1.13	0.2485	0.9960	1.02	0.8129	0.9939	0.92	0.3656	0.9951	0.94	0.5677	0.9856	1.12	0.2410	0.7187	0.92	0.3796	0.6113	0.89	0.1871	0.9889	0.88	0.1438	0.9607
40S ribosomal protein S19	RS19_HUMAN	P39019	0.94	0.2785	0.5509	0.97	0.5819	0.9982	0.97	0.6441	0.9960	0.94	0.3039	0.9939	1.03	0.6639	0.9951	1.00	0.9965	0.9992	1.10	0.1235	0.6868	0.96	0.5317	0.7227	0.95	0.3562	0.9889	0.98	0.6799	0.9881
40S ribosomal protein S2	RS2_HUMAN	P15880	0.98	0.8255	0.9186	1.01	0.8957	0.9982	1.03	0.7533	0.9960	1.04	0.6277	0.9939	1.09	0.2681	0.9951	0.92	0.3655	0.9856	1.15	0.0847	0.6824	0.81	0.0105	0.1455	0.97	0.6620	0.9889	1.03	0.7014	0.9881
40S ribosomal protein S20	RS20_HUMAN	P60866	0.92	0.1578	0.4265	0.96	0.5657	0.9982	1.04	0.5941	0.9960	0.98	0.7003	0.9939	0.94	0.3181	0.9951	0.99	0.9237	0.9959	0.98	0.8163	0.9481	1.01	0.8909	0.9398	0.89	0.0574	0.9889	0.94	0.3536	0.9607
40S ribosomal protein S21	RS21_HUMAN	P63220	1.03	0.5766	0.7759	1.13	0.0352	0.9982	1.02	0.7298	0.9960	1.10	0.1137	0.9874	1.05	0.4053	0.9951	0.99	0.8502	0.9959	1.05	0.4384	0.8116	0.99	0.8644	0.9252	0.98	0.6963	0.9889	0.97	0.6534	0.9881
40S ribosomal protein S23	RS23_HUMAN	P62266	0.96	0.5421	0.7503	0.94	0.3686	0.9982	0.97	0.6383	0.9960	1.04	0.5663	0.9939	1.04	0.6067	0.9951	0.96	0.6291	0.9856	1.10	0.1647	0.6896	0.99	0.8348	0.9130	0.96	0.5051	0.9889	1.00	0.9553	0.9938
40S ribosomal protein S3	RS3_HUMAN	P23396	0.92	0.2056	0.4823	1.02	0.7323	0.9982	0.97	0.6939	0.9960	0.95	0.4570	0.9939	0.95	0.5201	0.9951	0.97	0.7559	0.9857	0.96	0.5669	0.8601	0.94	0.4147	0.6399	1.01	0.8809	0.9889	0.95	0.4915	0.9712
40S ribosomal protein S3a	RS3A_HUMAN	P61247	0.89	0.0490	0.2898	0.97	0.6527	0.9982	1.04	0.6107	0.9960	1.02	0.7656	0.9939	1.01	0.9093	0.9951	0.96	0.6106	0.9856	1.18	0.0073	0.6779	0.90	0.1206	0.3537	0.93	0.2100	0.9889	1.01	0.9223	0.9938
40S ribosomal protein S4, X isoform	RS4X_HUMAN	P62701	0.85	0.0797	0.3281	0.84	0.0497	0.9982	1.21	0.0726	0.9960	1.13	0.1872	0.9939	1.01	0.9148	0.9951	0.79	0.0337	0.9411	1.26	0.0178	0.6779	0.94	0.5311	0.7227	0.97	0.7386	0.9889	0.93	0.4062	0.9607
40S ribosomal protein S5	RS5_HUMAN	P46782	0.98	0.7504	0.8824	1.01	0.8256	0.9982	1.00	0.9910	0.9960	1.02	0.8075	0.9939	1.07	0.3479	0.9951	1.00	0.9618	0.9967	1.16	0.0337	0.6824	0.86	0.0436	0.2215	0.92	0.2316	0.9889	1.00	0.9592	0.9946
40S ribosomal protein S7	RS7_HUMAN	P62081	0.87	0.0294	0.2873	1.06	0.3413	0.9982	0.91	0.1946	0.9960	0.89	0.0829	0.9874	0.86	0.0193	0.9951	1.08	0.3539	0.9856	1.05	0.4741	0.8329	1.00	0.9759	0.9860	0.83	0.0039	0.9889	0.87	0.0312	0.9607
40S ribosomal protein S9	RS9_HUMAN	P46781	0.88	0.0579	0.2973	0.97	0.6746	0.9982	1.01	0.8628	0.9960	0.94	0.3349	0.9939	0.91	0.1940	0.9951	0.97	0.7242	0.9857	1.07	0.3234	0.7400	0.94	0.4104	0.6358	0.93	0.2995	0.9889	0.91	0.1564	0.9607
40S ribosomal protein SA	RSSA_HUMAN	P08865	1.07	0.2629	0.5333	1.01	0.8105	0.9982	1.04	0.5452	0.9960	0.99	0.9290	0.9939	1.01	0.8339	0.9951	0.98	0.7933	0.9921	1.02	0.7365	0.9218	1.10	0.1746	0.4260	1.01	0.9053	0.9889	1.00	0.9721	0.9992
5-demethoxyubiquinone hydroxylase, mitochondrial	COQ7_HUMAN	Q99807	1.14	0.0347	0.2873	1.07	0.3114	0.9982	0.97	0.6669	0.9960	1.01	0.8709	0.9939	1.03	0.6356	0.9951	0.97	0.6868	0.9856	0.98	0.7488	0.9272	1.07	0.3259	0.5681	1.03	0.6873	0.9889	1.03	0.6761	0.9881
5-oxoprolinase	OPLA_HUMAN	O14841	1.08	0.3584	0.6214	1.00	0.9634	0.9982	0.90	0.3180	0.9960	1.01	0.8845	0.9939	1.02	0.8056	0.9951	1.24	0.0423	0.9411	0.82	0.0307	0.6824	1.21	0.0479	0.2315	1.16	0.0922	0.9889	1.08	0.3628	0.9607
5'-AMP-activated protein kinase subunit beta-2	AAKB2_HUMAN	O43741	1.18	0.0543	0.2939	0.98	0.8417	0.9982	1.00	0.9883	0.9960	1.15	0.1151	0.9874	1.16	0.0929	0.9951	1.04	0.7135	0.9857	1.13	0.1987	0.7149	1.12	0.2550	0.5197	1.06	0.5212	0.9889	1.09	0.3352	0.9607
6-phosphogluconate dehydrogenase, decarboxylating	6PGD_HUMAN	P52209	1.01	0.8817	0.9384	0.96	0.5592	0.9982	0.90	0.2183	0.9960	0.98	0.8398	0.9939	0.98	0.7580	0.9951	1.21	0.0379	0.9411	1.08	0.3363	0.7459	1.02	0.7875	0.8860	0.90	0.1574	0.9889	0.89	0.1112	0.9607
60 kDa heat shock protein, mitochondrial	CH60_HUMAN	P10809	1.08	0.1562	0.4253	0.98	0.7233	0.9982	0.94	0.3663	0.9960	0.97	0.5985	0.9939	0.97	0.5869	0.9951	1.08	0.2640	0.9856	0.90	0.0915	0.6824	1.12	0.0746	0.2814	1.06	0.3623	0.9889	0.99	0.8523	0.9881
60 kDa SS-A/Ro ribonucleoprotein	RO60_HUMAN	P10155	1.11	0.1863	0.4611	1.15	0.0892	0.9982	1.00	0.9659	0.9960	1.00	0.9644	0.9947	0.97	0.6977	0.9951	0.99	0.8861	0.9959	0.89	0.1796	0.7130	0.93	0.4260	0.6490	0.96	0.5995	0.9889	0.96	0.6396	0.9881
60S acidic ribosomal protein P0	RLA0_HUMAN	P05388	1.02	0.6233	0.8128	1.05	0.2880	0.9982	1.12	0.0174	0.9960	1.07	0.1302	0.9874	1.01	0.8318	0.9951	0.87	0.0071	0.9411	0.99	0.8870	0.9753	0.99	0.8630	0.9252	0.97	0.4384	0.9889	0.98	0.7170	0.9881
60S acidic ribosomal protein P1	RLA1_HUMAN	P05386	1.03	0.5216	0.7363	0.99	0.8582	0.9982	1.09	0.1316	0.9960	1.06	0.1957	0.9939	1.03	0.4884	0.9951	0.91	0.0900	0.9856	1.05	0.3360	0.7459	1.00	0.9786	0.9881	0.98	0.6493	0.9889	1.03	0.5331	0.9881
60S acidic ribosomal protein P2	RLA2_HUMAN	P05387	0.94	0.2431	0.5171	0.98	0.7089	0.9982	0.98	0.7454	0.9960	1.02	0.6811	0.9939	0.93	0.1968	0.9951	0.99	0.8358	0.9959	0.92	0.1434	0.6871	1.06	0.3049	0.5503	1.05	0.4258	0.9889	0.97	0.6100	0.9881
60S ribosomal protein L10a	RL10A_HUMAN	P62906	1.03	0.6105	0.8008	1.03	0.5960	0.9982	0.99	0.8255	0.9960	1.04	0.4936	0.9939	1.03	0.6262	0.9951	0.99	0.8230	0.9959	1.03	0.5688	0.8601	0.94	0.3077	0.5505	1.08	0.1487	0.9889	1.06	0.2757	0.9607

60S ribosomal protein L11	RL11_HUMAN	P62913	0.95	0.3109	0.5811	1.03	0.5809	0.9982	1.03	0.6798	0.9960	1.01	0.8399	0.9939	0.97	0.6371	0.9951	0.94	0.4101	0.9856	1.11	0.0860	0.6824	0.93	0.2212	0.4893	0.98	0.7468	0.9889	0.94	0.2935	0.9607
60S ribosomal protein L12	RL12_HUMAN	P30050	0.92	0.0664	0.3105	0.96	0.3785	0.9982	1.06	0.2385	0.9960	1.01	0.8062	0.9939	0.94	0.2105	0.9951	0.93	0.1626	0.9856	1.02	0.6045	0.8683	0.98	0.6237	0.7880	0.95	0.2322	0.9889	0.95	0.2340	0.9607
60S ribosomal protein L13	RL13_HUMAN	P26373	0.84	0.0035	0.2012	0.95	0.4153	0.9982	0.99	0.8850	0.9960	0.98	0.7255	0.9939	0.92	0.1689	0.9951	1.03	0.6747	0.9856	1.08	0.2347	0.7187	0.95	0.4660	0.6760	0.95	0.4077	0.9889	0.90	0.0885	0.9607
60S ribosomal protein L14	RL14_HUMAN	P50914	0.93	0.3440	0.6081	0.95	0.4888	0.9982	1.06	0.5057	0.9960	0.96	0.5481	0.9939	1.04	0.5746	0.9951	0.98	0.8265	0.9959	1.15	0.0747	0.6824	0.90	0.2293	0.4943	0.96	0.5764	0.9889	1.01	0.8769	0.9938
60S ribosomal protein L23a	RL23A_HUMAN	P62750	0.89	0.1265	0.3932	1.12	0.1219	0.9982	1.11	0.2123	0.9960	0.94	0.3713	0.9939	0.87	0.0617	0.9951	0.93	0.4318	0.9856	0.98	0.8397	0.9537	1.02	0.8420	0.9149	0.98	0.8298	0.9889	0.93	0.2946	0.9607
60S ribosomal protein L30	RL30_HUMAN	P62888	0.98	0.6888	0.8496	1.13	0.0309	0.9982	1.07	0.3290	0.9960	1.07	0.2509	0.9939	0.97	0.6109	0.9951	0.95	0.4886	0.9856	1.07	0.2596	0.7187	0.96	0.5135	0.7082	0.97	0.5613	0.9889	0.99	0.8552	0.9881
60S ribosomal protein L31	RL31_HUMAN	P62899	0.75	0.0015	0.2005	0.89	0.2209	0.9982	0.94	0.5699	0.9960	0.87	0.1467	0.9874	0.86	0.1180	0.9951	1.03	0.8073	0.9959	1.08	0.4550	0.8208	1.05	0.6180	0.7841	0.86	0.1250	0.9889	0.79	0.0121	0.9304
60S ribosomal protein L5	RL5_HUMAN	P46777	1.02	0.6703	0.8396	1.07	0.2207	0.9982	1.08	0.2134	0.9960	1.13	0.0242	0.9874	1.05	0.3576	0.9951	0.87	0.0435	0.9411	1.12	0.0511	0.6824	0.88	0.0340	0.2017	1.03	0.6618	0.9889	1.00	0.9523	0.9938
60S ribosomal protein L6	RL6_HUMAN	Q02878	1.02	0.7509	0.8824	1.14	0.0477	0.9982	0.93	0.3677	0.9960	1.07	0.2995	0.9939	1.08	0.2394	0.9951	1.06	0.4359	0.9856	1.11	0.1153	0.6824	0.85	0.0192	0.1651	1.14	0.0533	0.9889	1.04	0.5225	0.9846
60S ribosomal protein L7	RL7_HUMAN	P18124	0.89	0.1132	0.3748	1.00	0.9698	0.9982	0.98	0.7934	0.9960	1.00	0.9544	0.9939	0.97	0.6642	0.9951	1.02	0.8482	0.9959	1.09	0.2869	0.7212	0.97	0.7499	0.8625	1.03	0.6848	0.9889	1.01	0.8564	0.9881
60S ribosomal protein L7a	RL7A_HUMAN	P62424	0.95	0.5127	0.7297	0.89	0.1063	0.9982	0.95	0.5230	0.9960	0.97	0.6401	0.9939	0.98	0.8005	0.9951	0.97	0.7041	0.9857	0.98	0.8167	0.9481	0.99	0.9181	0.9562	1.05	0.5200	0.9889	1.03	0.7302	0.9881
A-kinase anchor protein 12	AKA12_HUMAN	Q02952	0.85	0.0644	0.3091	0.94	0.4933	0.9982	0.97	0.7488	0.9960	1.06	0.5004	0.9939	0.89	0.2037	0.9951	1.04	0.7029	0.9857	1.22	0.0315	0.6824	0.84	0.0787	0.2863	0.97	0.7611	0.9889	0.92	0.3144	0.9607
A-kinase anchor protein 2	AKAP2_HUMAN	Q9Y2D5	0.93	0.2960	0.5677	0.97	0.6966	0.9982	1.13	0.1116	0.9960	1.03	0.6988	0.9939	0.97	0.6530	0.9951	0.90	0.2162	0.9856	1.02	0.8275	0.9492	1.04	0.6130	0.7814	1.05	0.5104	0.9889	0.96	0.4906	0.9712
Acetolactate synthase-like protein	ILVBL_HUMAN	A1L0T0	0.99	0.8636	0.9348	0.96	0.5585	0.9982	1.02	0.7670	0.9960	1.00	0.9988	0.9995	1.02	0.7659	0.9951	0.92	0.3492	0.9856	0.98	0.7928	0.9431	1.04	0.6274	0.7896	1.03	0.7029	0.9889	1.07	0.3143	0.9607
Acetyl-CoA acetyltransferase, mitochondrial	THIL_HUMAN	P24752	1.03	0.6136	0.8030	0.94	0.2968	0.9982	0.98	0.7337	0.9960	0.97	0.6146	0.9939	0.95	0.3386	0.9951	1.01	0.8476	0.9959	0.98	0.7223	0.9201	1.14	0.0404	0.2167	1.03	0.6577	0.9889	0.97	0.6364	0.9881
Acetyl-coenzyme A synthetase 2-like, mitochondrial	ACS2L_HUMAN	Q9NUB1	1.26	0.0195	0.2873	1.04	0.7037	0.9982	0.89	0.3228	0.9960	1.00	0.9896	0.9981	1.03	0.7741	0.9951	1.12	0.3483	0.9856	0.91	0.3693	0.7673	1.19	0.1223	0.3555	1.06	0.5455	0.9889	1.00	0.9937	0.9992
Acid ceramidase	ASAH1_HUMAN	Q13510	1.07	0.4245	0.6724	1.00	0.9963	0.9985	0.99	0.8797	0.9960	1.11	0.2026	0.9939	1.05	0.5357	0.9951	0.95	0.5718	0.9856	0.95	0.5410	0.8573	1.22	0.0244	0.1768	0.94	0.4778	0.9889	0.95	0.5398	0.9881
Acidic leucine-rich nuclear phosphoprotein 32 family member A	AN32A_HUMAN	P39687	1.07	0.4135	0.6657	0.99	0.9371	0.9982	0.95	0.6067	0.9960	0.89	0.1679	0.9874	0.93	0.4109	0.9951	1.16	0.1526	0.9856	0.81	0.0149	0.6779	1.19	0.0548	0.2454	0.99	0.9029	0.9889	0.96	0.5937	0.9881
Aconitate hydratase, mitochondrial	ACON_HUMAN	Q99798	1.17	0.0407	0.2873	1.05	0.5435	0.9982	0.87	0.1032	0.9960	0.92	0.3090	0.9939	0.98	0.8056	0.9951	1.19	0.0640	0.9641	0.92	0.2804	0.7212	0.98	0.8249	0.9067	1.01	0.9008	0.9889	1.03	0.7156	0.9881
Actin-binding LIM protein 1	ABLM1_HUMAN	O14639	0.99	0.8279	0.9186	1.02	0.7804	0.9982	1.05	0.4990	0.9960	1.04	0.5950	0.9939	1.02	0.8115	0.9951	0.89	0.1379	0.9856	1.02	0.7811	0.9362	0.90	0.1548	0.4044	1.05	0.4521	0.9889	1.04	0.5427	0.9881
Actin-like protein 6A	ACL6A_HUMAN	O96019	1.12	0.0466	0.2898	0.98	0.7858	0.9982	0.92	0.1914	0.9960	1.10	0.1033	0.9874	1.14	0.0259	0.9951	1.06	0.4238	0.9856	1.01	0.9151	0.9848	0.89	0.0826	0.2936	1.06	0.3007	0.9889	1.12	0.0453	0.9607
Actin-related protein 2	ARP2_HUMAN	P61160	0.85	0.0573	0.2961	1.00	0.9641	0.9982	1.00	0.9723	0.9960	1.00	0.9753	0.9961	0.89	0.2172	0.9951	1.00	0.9950	0.9992	1.12	0.2240	0.7187	0.87	0.1579	0.4067	0.93	0.4362	0.9889	0.87	0.1129	0.9607
Actin-related protein 2/3 complex subunit 1A	ARC1A_HUMAN	Q92747	0.96	0.6879	0.8496	1.10	0.2864	0.9982	1.05	0.6281	0.9960	0.95	0.6004	0.9939	0.86	0.1220	0.9951	1.00	0.9696	0.9967	1.00	0.9779	0.9952	1.13	0.2508	0.5152	1.20	0.0465	0.9889	0.89	0.2236	0.9607
Actin-related protein 2/3 complex subunit 3	ARPC3_HUMAN	O15145	0.87	0.1314	0.3958	1.07	0.4979	0.9982	1.08	0.4636	0.9960	0.99	0.9248	0.9939	0.91	0.3452	0.9951	0.91	0.4205	0.9856	1.19	0.0819	0.6824	0.81	0.0415	0.2169	0.95	0.6227	0.9889	0.85	0.0692	0.9607
Actin-related protein 2/3 complex subunit 4	ARPC4_HUMAN	P59998	0.89	0.0486	0.2898	1.07	0.2361	0.9982	0.98	0.7841	0.9960	1.07	0.2457	0.9939	0.91	0.1331	0.9951	1.04	0.6372	0.9856	1.07	0.3098	0.7378	0.96	0.5176	0.7125	1.05	0.4325	0.9889	0.93	0.2277	0.9607
Actin-related protein 3	ARP3_HUMAN	P61158	0.87	0.1295	0.3958	1.10	0.3248	0.9982	0.99	0.9299	0.9960	0.97	0.7506	0.9939	0.87	0.1549	0.9951	1.03	0.7808	0.9897	1.19	0.0859	0.6824	0.90	0.3084	0.5505	0.90	0.2692	0.9889	0.88	0.1583	0.9607
Actin, alpha skeletal muscle	ACTS_HUMAN	P68133	0.91	0.3331	0.5975	0.96	0.6795	0.9982	1.18	0.1497	0.9960	1.06	0.5654	0.9939	1.01	0.9112	0.9951	0.78	0.0424	0.9411	1.15	0.1954	0.7149	0.86	0.1879	0.4466	1.06	0.5699	0.9889	0.98	0.8384	0.9881
Actin, aortic smooth muscle	ACTA_HUMAN	P62736	0.97	0.6644	0.8375	1.01	0.8461	0.9982	1.00	0.9965	0.9983	1.04	0.5193	0.9939	1.03	0.7089	0.9951	0.96	0.6505	0.9856	1.08	0.2565	0.7187	0.91	0.1831	0.4380	1.05	0.4986	0.9889	1.07	0.3034	0.9607
Actin, cytoplasmic 1	ACTB_HUMAN	P60709	0.76	0.0140	0.2728	1.04	0.7223	0.9982	0.99	0.9167	0.9960	0.87	0.2401	0.9939	0.81	0.0714	0.9951	1.05	0.7376	0.9857	1.16	0.2350	0.7187	0.76	0.0356	0.2045	0.91	0.4023	0.9889	0.88	0.2611	0.9607
Acyl carrier protein, mitochondrial	ACPM_HUMAN	O14561	1.12	0.2483	0.5210	0.98	0.8232	0.9982	0.98	0.8794	0.9960	0.86	0.1252	0.9874	0.95	0.5824	0.9951	1.04	0.7391	0.9857	0.91	0.3630	0.7668	1.30	0.0169	0.1636	1.04	0.6764	0.9889	1.05	0.6510	0.9881
Acyl-CoA synthetase short-chain family member 3, mitochondrial	ACSS3_HUMAN	Q9H6R3	1.03	0.7319	0.8760	0.93	0.4610	0.9982	1.11	0.3194	0.9960	0.90	0.2432	0.9939	0.94	0.4852	0.9951	0.90	0.3434	0.9856	0.98	0.8227	0.9481	1.32	0.0052	0.1317	0.89	0.1941	0.9889	0.95	0.5726	0.9881
Acyl-CoA-binding protein	ACBP_HUMAN	P07108	1.02	0.8777	0.9384	1.04	0.7563	0.9982	1.06	0.6739	0.9960	0.93	0.5627	0.9939	0.92	0.5166	0.9951	1.11	0.4984	0.9856	0.92	0.5347	0.8573	1.42	0.0070	0.1326	0.95	0.6881	0.9889	0.86	0.2050	0.9607
Acyl-coenzyme A thioesterase 1	ACOT1_HUMAN	Q86TX2	1.09	0.4476	0.6928	1.02	0.8514	0.9982	0.80	0.0971	0.9960	0.89	0.2915	0.9939	0.91	0.4181	0.9951	1.27	0.0932	0.9856	0.82	0.1004	0.6824	1.23	0.1092	0.3360	1.21	0.1026	0.9889	1.05	0.6420	0.9881
Acyl-coenzyme A thioesterase 13	ACO13_HUMAN	Q9NPJ3	1.09	0.2067	0.4832	1.02	0.8150	0.9982	0.93	0.3655	0.9960	0.91	0.1650	0.9874	0.95	0.4459	0.9951	1.11	0.2123	0.9856	1.00	0.9520	0.9923	1.08	0.3146	0.5585	1.00	0.9524	0.9952	0.94	0.3867	0.9607
Acyl-coenzyme A thioesterase 2, mitochondrial	ACOT2_HUMAN	P49753	1.23	0.0644	0.3091	1.06	0.5885	0.9982	0.93	0.5858	0.9960	0.97	0.8119	0.9939	1.03	0.7754	0.9951	1.13	0.3751	0.9856	0.89	0.3191	0.7400	1.21	0.1340	0.3740	1.06	0.5885	0.9889	1.00	0.9878	0.9992
Acyl-coenzyme A thioesterase 9, mitochondrial	ACOT9_HUMAN	Q9Y305	1.01	0.8344	0.9199	1.01	0.8273	0.9982	1.00	0.9502	0.9960	1.04	0.5243	0.9939	0.99	0.8860	0.9951	0.97	0.7001	0.9857	0.92	0.2187	0.7187	1.01	0.8370	0.9133	1.10	0.1598	0.9889	1.01	0.8993	0.9938
Acyl-protein thioesterase 1	LYPA1_HUMAN	O75608	1.21	0.0080	0.2487	1.05	0.5389	0.9982	0.89	0.1825	0.9960	0.93	0.3027	0.9939	1.06	0.4591	0.9951	1.14	0.1524	0.9856	0.96	0.5639	0.8601	1.08	0.3651	0.5972	1.00	0.9670	0.9964	1.09	0.2540	0.9607
Acylamino-acid-releasing enzyme	ACPH_HUMAN	P13798	1.09	0.2411	0.5162	0.99	0.9072	0.9982	0.95	0.5631	0.9960	0.97	0.6832	0.9939	0.95	0.5269	0.9951	1.09	0.3802	0.9856	0.89	0.1452	0.6871	1.20	0.0330	0.2017	0.89	0.1553	0.9889	0.93	0.3620	0.9607
Acylpyruvase FAHD1, mitochondrial	FAHD1_HUMAN	Q6P587	1.07	0.2597	0.5306	1.04	0.5557	0.9982	0.93	0.3217	0.9960	1.00	0.9436	0.9939	1.00	0.9746	0.9951	1.05	0.5588	0.9856	0.90	0.1363	0.6871	0.95	0.4681	0.6776	0.95	0.4534	0.9889	0.99	0.8868	0.9938
Adapter molecule crk	CRK_HUMAN	P46108	1.09	0.3172	0.5859	1.01	0.8968	0.9982	0.90	0.3055	0.9960	0.90	0.1986	0.9939	0.97	0.7284	0.9951	1.18	0.1172	0.9856	0.90	0.2406	0.7187	1.01	0.9214	0.9569	1.01	0.9347	0.9909	1.02	0.8087	0.9881
Adenosylhomocysteinase	SAHH_HUMAN	P23526	1.15	0.0566	0.2942	0.97	0.6531	0.9982	0.94	0.4439	0.9960	0.93	0.3191	0.9939	1.05	0.5281	0.9951	1.18	0.0561	0.9641	0.99	0.9275	0.9880	1.22	0.0127	0.1544	0.99	0.9056	0.9889	1.05	0.4735	0.9611
Adenylate kinase 2, mitochondrial	KAD2_HUMAN	P54819	1.16	0.0188	0.2873	1.02	0.7754	0.9982	0.93	0.3590	0.9960	0.91	0.1365	0.9874	1.03	0.6681	0.9951	1.09	0.2682	0.9856	0.93	0.3235	0.7400	1.00	0.9643	0.9796	1.00	0.9433	0.9922	1.00	0.9505	0.9938
Adenylate kinase 4, mitochondrial	KAD4_HUMAN	P27144	1.01	0.8723	0.9360	1.00	0.9387	0.9982	0.99	0.8976	0.9960	1.00	0.9515	0.9939	0.99	0.8251	0.9951	0.99	0.9424	0.9959	1.04	0.5341	0.8573	0.99	0.8226	0.9062	1.04	0.4952	0.9889	0.97	0.6552	0.9881
Adenylate kinase isoenzyme 1	KAD1_HUMAN	P00568	1.11	0.1427	0.4055	0.98	0.8096	0.9982	0.98	0.8553	0.9960	0.94	0.3724	0.9939	0.96	0.6030	0.9951	1.07	0.4428	0.9856	0.90	0.1745	0.7019	1.19	0.0347	0.2017	1.05	0.5036	0.9889	1.04	0.6257	0.9881
Adenylosuccinate synthetase isozyme 1	PURA1_HUMAN	Q8N142	1.22	0.0841	0.3334	1.27	0.0368	0.9982	0.89	0.3882	0.9960	1.02	0.8756	0.9939	1.04	0.7621	0.9951	1.11	0.4600	0.9856	1.06	0.6370	0.8888	0.84	0.1616	0.4114	1.03	0.7967	0.9889	0.99	0.9457	0.9938
Adenylyl cyclase-associated protein 1	CAP1_HUMAN	Q01518	0.94	0.3239	0.5917	1.00	0.9796	0.9985	1.02	0.8302	0.9960	0.97	0.5748	0.9939	0.92	0.1773	0.9951	1.01	0.8935	0.9959	1.04	0.5910	0.8646	0.95	0.4423	0.6607	0.95	0.4096	0.9889	0.91	0.1034	0.9607
Adenylyl cyclase-associated protein 2	CAP2_HUMAN	P40123	1.04	0.5002	0.7246	0.94	0.3133	0.9982	1.05	0.5117	0.9960	0.99	0.8474	0.9939	0.95	0.4368	0.9951	0.99	0.9165	0.9959	0.88	0.0681	0.6824	1.15	0.0539	0.2446	0.96	0.5722	0.9889	0.99	0.9073	0.9938
Adipocyte enhancer-binding protein 1	AEBP1_HUMAN	Q8IUX7	0.73	0.0709	0.3218	0.83	0.3021	0.9982	1.09	0.6739	0.9960	1.13	0.4900	0.9939	0.83	0.3090	0.9951	0.83	0.4152	0.9856	0.92	0.6579	0.8915	0.73	0.1143	0.3452	0.82	0.2834	0.9889	0.88	0.4625	0.9607
Adipocyte plasma membrane-associated protein	APMAP_HUMAN	Q9HDC9	0.87	0.0682	0.3121	0.97	0.6544	0.9982	1.08	0.3908	0.9960	1.07	0.3808	0.9939	1.00	0.9631	0.9951	0.87	0.1489	0.9856	1.23	0.0101	0.6779	0.86	0.0844	0.2956	0.88	0.1032	0.9889	0.93	0.3497	0.9607
Adiponectin	ADIPO_HUMAN	Q15848	1.05	0.5043	0.7271	0.94	0.4587	0.9982	1.11	0.2624	0.9960	0.91	0.2405	0.9939	0.94	0.4396	0.9951	0.87	0.1465	0.9856	0.96	0.6556	0.8915	0.94	0.4605	0.6738	0.90	0.1681	0.9889	0.96	0.6192	0.9881
ADP-ribose glycohydrolase MACROD1	MACD1_HUMAN	Q9BQ69	1.15	0.0301	0.2873	1.07	0.3362	0.9982	0.93	0.3324	0.9960	0.91	0.1470	0.9874	0.98	0.7941	0.9951	1.10	0.2571	0.9856	0.86	0.0328	0.6824	1.07	0.3596	0.5943	1.03	0.6618	0.9889	0.99	0.9058	0.9938
ADP-ribose pyrophosphatase, mitochondrial	NUDT9_HUMAN	Q9BW91	1.17	0.0417	0.2873	0.98	0.7935	0.9982	0.94	0.5085	0.9960	1.04	0.6073	0.9939	1.08	0.3426	0.9951	1.07	0.4927	0.9856	0.96	0.6362	0.8888	1.05	0.5896	0.7646	1.05	0.5072	0.9889	1.04	0.6145	0.9881
ADP-ribosylation factor 3	ARF3_HUMAN	P61204	0.98	0.7604	0.8882	1.07	0.2858	0.9982	0.99	0.8883	0.9960	1.04	0.5239	0.9939	1.02	0.7077	0.9951	0.98	0.8023	0.9959	1.05	0.4602	0.8243	0.91	0.1532	0.4037	1.09	0.1715	0.9889	1.07	0.2860	0.9607
ADP/ATP translocase 1	ADT1_HUMAN	P12235	1.14	0.1655	0.4371	1.11	0.2815	0.9982	1.04	0.7151	0.9960	1.02	0.8258	0.9939	1.07	0.4940	0.9951	0.92	0.4742	0.9856	0.93	0.5000	0.8476	1.09	0.4251	0.6483	1.05	0.6386	0.9889	1.11	0.2720	0.9607
ADP/ATP translocase 2	ADT2_HUMAN	P05141	0.93	0.4095	0.6635	1.07	0.4146	0.9982	1.11	0.2793	0.9960	1.05	0.5850	0.9939	1.00	0.9900	0.9969	0.85	0.0955	0.9856	1.04	0.6913	0.9047	0.91	0.2937	0.5464	1.02	0.8013	0.9889	0.97	0.7083	0.9881
Adrenodoxin, mitochondrial	ADX_HUMAN	P10109	1.15	0.0387	0.2873	1.05	0.4656	0.9982	0.89	0.1193	0.9960	1.00	0.9545	0.9939	1.08	0.2854	0.9951	1.13	0.1347	0.9856	0.98	0.7627	0.9282	0.94	0.3962	0.6232	0.98	0.8148	0.9889	1.03	0.6798	0.9881
Afamin	AFAM_HUMAN	P43652	0.82	0.0521	0.2928	1.01	0.9349	0.9982	0.94	0.6372	0.9960	1.02	0.8740	0.9939	0.95	0.6681	0.9951	1.07	0.6217	0.9856	1.07	0.5675	0.8601	0.85	0.1531	0.4037	1.00	0.9986	0.9990	0.85	0.1258	0.9607
AFG3-like protein 2	AFG32_HUMAN	Q9Y4W6	1.06	0.3380	0.6021	1.02	0.7753	0.9982	1.01	0.8787	0.9960	1.07	0.2726	0.9939	1.05	0.4629	0.9951	0.93	0.3636	0.9856	0.94	0.3755	0.7723	1.05	0.4960	0.6959	1.03	0.6753	0.9889	1.04	0.5359	0.9881
Aflatoxin B1 aldehyde reductase member 2	ARK72_HUMAN	O43488	1.13	0.0516	0.2912	1.03	0.6393	0.9982	0.96	0.5411	0.9960	0.93	0.2452	0.9939	1.01	0.8231	0.9951	1.08	0.3186	0.9856	0.93	0.2469	0.7187	1.08	0.2590	0.5237	1.05	0.4840	0.9889	1.02	0.7290	0.9881
Agrin	AGRIN_HUMAN	O00468	0.73	0.0074	0.2487	1.14	0.2874	0.9982	1.21	0.1719	0.9960	0.90	0.3673	0.9939	0.79	0.0552	0.9951	0.88	0.3800	0.9856	1.10	0.4622	0.8243	0.80	0.0953	0.3123	1.12	0.3761	0.9889	0.80	0.0570	0.9607
Alanine aminotransferase 1	ALAT1_HUMAN	P24298	1.22	0.1208	0.3822	1.09	0.5084	0.9982	0.91	0.5250	0.9960	0.99	0.9252	0.9939	0.96	0.7613	0.9951	1.18	0.2844	0.9856	0.92	0.5466	0.8600	1.07	0.6526	0.8015	0.94	0.6257	0.9889	0.95	0.7003	0.9881
Alcohol dehydrogenase class-3	ADHX_HUMAN	P11766	1.12	0.1749	0.4501	0.90	0.1985	0.9982	0.96	0.6502	0.9960	0.90	0.1952	0.9939	1.04	0.6960	0.9951	1.12	0.2853	0.9856	0.89	0.2151	0.7173	1.34	0.0015	0.0774	0.92	0.3239	0.9889	1.01	0.9349	0.9938
Aldehyde dehydrogenase family 3 member A2	AL3A2_HUMAN	P51648	0.91	0.2335	0.5075	0.94	0.4475	0.9982	1.14	0.1257	0.9960	1.11	0.1841	0.9939	0.95	0.5360	0.9951	0.81	0.0231	0.9411	1.05	0.5823	0.8635	0.96	0.6514	0.8015	0.96	0.6189	0.9889	0.93	0.3321	0.9607
Aldehyde dehydrogenase X, mitochondrial	AL1B1_HUMAN	P30837	0.96	0.7823	0.8982	1.25	0.0864	0.9982	0.83	0.2050	0.9960	1.22	0.1209	0.9874	1.03	0.8143	0.9951	1.08	0.6350	0.9856	0.93	0.5885	0.8645	0.68	0.0066	0.1326	0.99	0.9239	0.9889	0.95	0.6911	0.9881
Aldehyde dehydrogenase, mitochondrial	ALDH2_HUMAN	P05091	1.17	0.0454	0.2898	1.13	0.1288	0.9982	0.94	0.4929	0.9960	0.90	0.1841	0.9939	0.95	0.5240	0.9951	1.08	0.4463	0.9856	0.91	0.2303	0.7187	1.07	0.4146	0.6399	1.06	0.4510	0.9889	1.03	0.7273	0.9881
Aldo-keto reductase family 1 member A1	AK1A1_HUMAN	P14550	1.03	0.7151	0.8705	1.08	0.2880	0.9982	0.98	0.8247	0.9960	0.88	0.0507	0.9874	1.01	0.8852	0.9951	1.08	0.3553	0.9856	0.96	0.5857	0.8645	1.06	0.4913	0.6922	0.98	0.8115	0.9889	0.93	0.2657	0.9607
Aldo-keto reductase family 1 member B1	ALDR_HUMAN	P15121	1.18	0.0672	0.3105	0.99	0.9570	0.9982	0.85	0.1405	0.9960	0.90	0.2688	0.9939	1.02	0.8427	0.9951	1.26	0.0431	0.9411	0.81	0.0359	0.6824	1.24	0.0400	0.2167	1.07	0.4674	0.9889	1.09	0.3689	0.9607
All-trans-retinol dehydrogenase [NAD(+)] ADH1B	ADH1B_HUMAN	P00325	0.76	0.0595	0.3008	1.17	0.2844	0.9982	0.99	0.9569	0.9960	0.88	0.3700	0.9939	0.72	0.0267	0.9951	1.03	0.8559	0.9959	1.03	0.8447	0.9558	0.88	0.4357	0.6555	1.00	0.9881	0.9976	0.82	0.1707	0.9607
Alpha-1-acid glycoprotein 1	A1AG1_HUMAN	P02763	0.88	0.3866	0.6444	1.13	0.4326	0.9982	1.12	0.5099	0.9960	1.15	0.3656	0.9939	1.12	0.4854	0.9951	0.91	0.6092	0.9856	1.03	0.8362	0.9531	0.67	0.0188	0.1650	1.00	0.9843	0.9976	1.05	0.7679	0.9881
Alpha-1-acid glycoprotein 2	A1AG2_HUMAN	P19652	0.85	0.1344	0.3972	1.00	0.9917	0.9985	1.06	0.6385	0.9960	1.13	0.2575	0.9939	1.02	0.8519	0.9951	0.93	0.5772	0.9856	1.07	0.5517	0.8601	0.89	0.3401	0.5756	1.00	0.9974	0.9990	0.97	0.7697	0.9881
Alpha-1-antichymotrypsin	AACT_HUMAN	P01011	0.85	0.2812	0.5543	1.17	0.3188	0.9982	1.16	0.4167	0.9960	1.18	0.2811	0.9939	1.07	0.6676	0.9951	0.77	0.1661	0.9856	1.34	0.0755	0.6824	0.59	0.0015	0.0773	0.87	0.3890	0.9889	0.92	0.5734	0.9881
Alpha-1-antitrypsin	A1AT_HUMAN	P01009	0.88	0.2909	0.5638	1.09	0.4669	0.9982	1.13	0.3657	0.9960	1.01	0.9112	0.9939	1.02	0.8851	0.9951	0.87	0.3196	0.9856	1.10	0.4504	0.8183	0.71	0.0061	0.1317	1.03	0.8146	0.9889	0.90	0.3670	0.9607
Alpha-1-syntrophin	SNTA1_HUMAN	Q13424	1.05	0.3276	0.5952	1.02	0.6875	0.9982	1.04	0.5359	0.9960	1.06	0.2602	0.9939	1.03	0.5792	0.9951	0.92	0.2000	0.9856	0.95	0.3516	0.7593	0.97	0.5784	0.7549	1.03	0.5323	0.9889	0.99	0.8649	0.9894
Alpha-1B-glycoprotein	A1BG_HUMAN	P04217	0.83	0.0258	0.2873	1.08	0.3568	0.9982	1.07	0.4638	0.9960	1.00	0.9760	0.9961	0.95	0.5269	0.9951	0.91	0.3508	0.9856	1.11	0.2460	0.7187	0.77	0.0039	0.1242	0.98	0.7861	0.9889	0.93	0.3841	0.9607
Alpha-2-HS-glycoprotein	FETUA_HUMAN	P02765	0.80	0.0405	0.2873	1.08	0.5092	0.9982	0.98	0.8935	0.9960	0.97	0.7621	0.9939	0.92	0.4564	0.9951	1.06	0.6903	0.9856	1.00	0.9811	0.9971	0.88	0.2966	0.5464	1.06	0.6039	0.9889	0.87	0.1878	0.9607
Alpha-2-macroglobulin	A2MG_HUMAN	P01023	0.97	0.8066	0.9060	1.17	0.1782	0.9982	1.06	0.6684	0.9960	1.10	0.3812	0.9939	1.12	0.3410	0.9951	0.88	0.3806	0.9856	1.23	0.0838	0.6824	0.73	0.0130	0.1544	0.95	0.6711	0.9889	0.99	0.9377	0.9938
Alpha-2-macroglobulin receptor-associated protein	AMRP_HUMAN	P30533	0.91	0.1302	0.3958	0.91	0.1275	0.9982	1.05	0.4929	0.9960	1.02	0.8029	0.9939	0.93	0.2861	0.9951	0.94	0.4309	0.9856	1.05	0.5022	0.8476	0.99	0.9225	0.9569	1.00	0.9893	0.9976	0.92	0.1810	0.9607
Alpha-actinin-1	ACTN1_HUMAN	P12814	0.93	0.2138	0.4853	0.94	0.2965	0.9982	1.04	0.5376	0.9960	0.94	0.2269	0.9939	0.87	0.0130	0.9951	0.97	0.6839	0.9856	0.93	0.2536	0.7187	1.07	0.2934	0.5464	0.99	0.9074	0.9889	0.93	0.1664	0.9607
Alpha-actinin-2	ACTN2_HUMAN	P35609	1.17	0.0277	0.2873	1.01	0.9249	0.9982	1.00	0.9553	0.9960	1.04	0.5995	0.9939	1.05	0.5280	0.9951	0.97	0.7522	0.9857	0.93	0.3682	0.7673	1.17	0.0405	0.2167	1.06	0.4182	0.9889	1.08	0.2737	0.9607
Alpha-actinin-4	ACTN4_HUMAN	O43707	0.93	0.1544	0.4236	0.99	0.8614	0.9982	0.97	0.5868	0.9960	0.94	0.2173	0.9939	0.90	0.0594	0.9951	1.09	0.1948	0.9856	1.00	0.9864	0.9992	0.94	0.2829	0.5358	0.99	0.8292	0.9889	0.94	0.2200	0.9607
Alpha-adducin	ADDA_HUMAN	P35611	1.00	0.9686	0.9819	1.08	0.4001	0.9982	1.04	0.6981	0.9960	0.89	0.1755	0.9907	0.89	0.1973	0.9951	0.99	0.9282	0.9959	1.05	0.6095	0.8715	0.91	0.3288	0.5681	0.94	0.4712	0.9889	0.93	0.4166	0.9607
Alpha-aminoadipic semialdehyde dehydrogenase	AL7A1_HUMAN	P49419	1.23	0.0284	0.2873	1.07	0.4552	0.9982	0.87	0.2221	0.9960	0.96	0.6596	0.9939	1.06	0.5268	0.9951	1.21	0.0955	0.9856	0.86	0.1304	0.6868	1.13	0.2521	0.5154	1.04	0.7093	0.9889	1.01	0.9070	0.9938
Alpha-aminoadipic semialdehyde synthase, mitochondrial	AASS_HUMAN	Q9UDR5	1.03	0.6847	0.8481	0.97	0.7394	0.9982	1.13	0.1993	0.9960	0.96	0.6591	0.9939	1.01	0.8929	0.9951	0.89	0.2326	0.9856	0.88	0.1249	0.6868	1.07	0.4577	0.6717	1.12	0.1936	0.9889	0.99	0.9344	0.9938
Alpha-centractin	ACTZ_HUMAN	P61163	0.99	0.8651	0.9348	1.03	0.5051	0.9982	0.99	0.8863	0.9960	1.07	0.1099	0.9874	1.02	0.6388	0.9951	0.96	0.4242	0.9856	1.06	0.1955	0.7149	0.97	0.5627	0.7438	0.94	0.1908	0.9889	1.01	0.8752	0.9938
Alpha-crystallin B chain	CRYAB_HUMAN	P02511	0.99	0.8947	0.9442	1.02	0.7403	0.9982	1.04	0.6000	0.9960	1.16	0.0273	0.9874	0.97	0.6656	0.9951	0.97	0.6943	0.9856	1.04	0.6156	0.8745	1.02	0.7845	0.8852	1.00	0.9852	0.9976	1.00	0.9682	0.9985
Alpha-enolase	ENOA_HUMAN	P06733	0.89	0.0095	0.2530	0.96	0.3327	0.9982	0.99	0.8175	0.9960	1.04	0.3922	0.9939	0.96	0.3802	0.9951	0.98	0.7841	0.9897	1.03	0.5806	0.8633	1.05	0.3372	0.5738	0.98	0.6934	0.9889	0.95	0.2851	0.9607
Alpha-parvin	PARVA_HUMAN	Q9NVD7	1.06	0.5196	0.7355	1.12	0.2174	0.9982	0.95	0.6131	0.9960	1.15	0.1139	0.9874	1.14	0.1528	0.9951	1.01	0.9371	0.9959	0.97	0.7861	0.9379	0.89	0.2471	0.5122	1.02	0.8421	0.9889	1.05	0.5974	0.9881
Alpha-sarcoglycan	SGCA_HUMAN	Q16586	1.08	0.3436	0.6081	1.03	0.6866	0.9982	1.02	0.8017	0.9960	1.07	0.4250	0.9939	1.19	0.0374	0.9951	0.91	0.3446	0.9856	1.06	0.4843	0.8365	0.81	0.0176	0.1636	0.98	0.7868	0.9889	1.09	0.3011	0.9607
Alpha-soluble NSF attachment protein	SNAA_HUMAN	P54920	0.95	0.3165	0.5859	1.00	0.9624	0.9982	1.09	0.1591	0.9960	0.95	0.3405	0.9939	0.92	0.1288	0.9951	0.89	0.0710	0.9641	0.98	0.7585	0.9282	1.02	0.7912	0.8865	0.92	0.0906	0.9889	0.92	0.0882	0.9607
Amine oxidase [flavin-containing] A	AOFA_HUMAN	P21397	1.06	0.5663	0.7665	1.16	0.1347	0.9982	1.15	0.2131	0.9960	1.07	0.4783	0.9939	1.04	0.6995	0.9951	0.78	0.0407	0.9411	0.87	0.2014	0.7149	0.88	0.2408	0.5050	1.01	0.9476	0.9935	1.08	0.4230	0.9607
Amine oxidase [flavin-containing] B	AOFB_HUMAN	P27338	1.17	0.1163	0.3780	1.17	0.1059	0.9982	1.15	0.2328	0.9960	1.05	0.5942	0.9939	1.12	0.2856	0.9951	0.80	0.0721	0.9641	0.90	0.3167	0.7400	0.95	0.6435	0.7988	0.98	0.8549	0.9889	1.13	0.2202	0.9607
Aminoacyl tRNA synthase complex-interacting multifunctional protein 1	AIMP1_HUMAN	Q12904	0.98	0.7217	0.8735	1.06	0.3798	0.9982	1.02	0.7725	0.9960	1.12	0.0740	0.9874	1.01	0.9369	0.9951	0.99	0.8865	0.9959	1.04	0.5652	0.8601	0.99	0.9338	0.9616	1.02	0.7500	0.9889	1.00	0.9814	0.9992
Aminomethyltransferase, mitochondrial	GCST_HUMAN	P48728	1.10	0.2349	0.5075	1.13	0.1307	0.9982	0.96	0.6957	0.9960	0.95	0.5533	0.9939	0.95	0.5092	0.9951	1.09	0.3756	0.9856	0.95	0.5292	0.8563	1.08	0.4093	0.6358	1.11	0.2038	0.9889	1.02	0.7815	0.9881
Angiotensin-converting enzyme 2	ACE2_HUMAN	Q9BYF1	0.81	0.0816	0.3290	0.93	0.5456	0.9982	1.28	0.0783	0.9960	0.95	0.6995	0.9939	0.91	0.4520	0.9951	0.77	0.0860	0.9856	0.79	0.0638	0.6824	1.25	0.1035	0.3276	0.99	0.9333	0.9907	0.86	0.2012	0.9607
Angiotensinogen	ANGT_HUMAN	P01019	0.86	0.1058	0.3654	0.97	0.7169	0.9982	1.11	0.3275	0.9960	1.15	0.1279	0.9874	1.11	0.2964	0.9951	0.89	0.3042	0.9856	1.13	0.2130	0.7171	0.90	0.2959	0.5464	1.03	0.7931	0.9889	1.08	0.4353	0.9607
Ankyrin repeat domain-containing protein 1	ANKR1_HUMAN	Q15327	0.94	0.4770	0.7141	0.95	0.5733	0.9982	1.13	0.2543	0.9960	1.17	0.0881	0.9874	0.93	0.4386	0.9951	0.84	0.1157	0.9856	0.98	0.8030	0.9456	1.23	0.0404	0.2167	0.90	0.2591	0.9889	0.89	0.2108	0.9607
Ankyrin repeat domain-containing protein 2	ANKR2_HUMAN	Q9GZV1	1.18	0.2644	0.5347	0.99	0.9517	0.9982	1.16	0.3912	0.9960	1.24	0.1443	0.9874	1.22	0.2013	0.9951	0.70	0.0514	0.9621	0.94	0.7153	0.9159	1.27	0.1523	0.4027	0.96	0.8015	0.9889	1.14	0.3962	0.9607
Ankyrin-2	ANK2_HUMAN	Q01484	1.05	0.4340	0.6812	1.07	0.3220	0.9982	1.02	0.7873	0.9960	0.96	0.4762	0.9939	0.97	0.6782	0.9951	0.99	0.8796	0.9959	0.99	0.9267	0.9880	1.06	0.4274	0.6497	1.04	0.5101	0.9889	1.03	0.6746	0.9881
Ankyrin-3	ANK3_HUMAN	Q12955	1.02	0.7855	0.8986	0.97	0.6455	0.9982	1.08	0.2495	0.9960	1.00	0.9576	0.9939	0.96	0.5233	0.9951	0.90	0.1463	0.9856	0.89	0.0726	0.6824	1.10	0.1427	0.3873	1.04	0.5308	0.9889	1.00	0.9391	0.9938

Annexin A1	ANXA1_HUMAN	P04083	0.87	0.1386	0.4015	1.06	0.5559	0.9982	1.00	0.9844	0.9960	1.05	0.6095	0.9939	0.90	0.2726	0.9951	1.04	0.7630	0.9857	1.27	0.0154	0.6779	0.80	0.0345	0.2017	0.86	0.1302	0.9889	0.84	0.0720	0.9607
Annexin A11	ANX11_HUMAN	P50995	1.06	0.2101	0.4843	1.05	0.3278	0.9982	1.08	0.1771	0.9960	1.04	0.4206	0.9939	1.04	0.4554	0.9951	0.91	0.1285	0.9856	1.02	0.7466	0.9270	1.00	0.9352	0.9616	0.98	0.6789	0.9889	1.02	0.6952	0.9881
Annexin A2	ANXA2_HUMAN	P07355	0.90	0.1632	0.4357	1.03	0.7031	0.9982	0.95	0.5482	0.9960	1.03	0.6902	0.9939	0.96	0.6333	0.9951	1.10	0.3069	0.9856	1.19	0.0252	0.6824	0.88	0.1316	0.3699	0.95	0.5148	0.9889	0.94	0.3893	0.9607
Annexin A3	ANXA3_HUMAN	P12429	1.14	0.0711	0.3218	1.09	0.2597	0.9982	1.03	0.6984	0.9960	0.95	0.4571	0.9939	1.02	0.7889	0.9951	0.98	0.8208	0.9959	0.88	0.0972	0.6824	1.04	0.6640	0.8089	1.03	0.7348	0.9889	0.98	0.7902	0.9881
Annexin A4	ANXA4_HUMAN	P09525	0.96	0.6225	0.8128	1.06	0.4435	0.9982	0.98	0.8251	0.9960	0.99	0.8500	0.9939	0.96	0.6086	0.9951	1.00	0.9863	0.9992	1.04	0.5714	0.8612	0.79	0.0032	0.1090	0.97	0.7035	0.9889	1.00	0.9872	0.9992
Annexin A5	ANXA5_HUMAN	P08758	0.92	0.2524	0.5219	1.06	0.4548	0.9982	0.99	0.9279	0.9960	0.93	0.3098	0.9939	0.90	0.1975	0.9951	1.03	0.7236	0.9857	0.99	0.8975	0.9786	0.83	0.0236	0.1763	0.95	0.5230	0.9889	0.89	0.1373	0.9607
Annexin A6	ANXA6_HUMAN	P08133	1.06	0.3647	0.6275	1.09	0.1668	0.9982	0.96	0.5884	0.9960	0.93	0.2632	0.9939	0.91	0.1422	0.9951	1.09	0.2654	0.9856	0.88	0.0587	0.6824	1.07	0.3166	0.5601	0.90	0.1198	0.9889	0.87	0.0238	0.9607
Annexin A7	ANXA7_HUMAN	P20073	1.11	0.0615	0.3038	1.09	0.1299	0.9982	0.95	0.4854	0.9960	1.00	0.9912	0.9981	1.01	0.8146	0.9951	1.06	0.3918	0.9856	0.97	0.5907	0.8646	0.98	0.7166	0.8381	0.98	0.7007	0.9889	1.02	0.7310	0.9881
Antithrombin-III	ANT3_HUMAN	P01008	0.86	0.0819	0.3290	1.04	0.6830	0.9982	1.14	0.2007	0.9960	0.99	0.8984	0.9939	0.97	0.7275	0.9951	0.91	0.3640	0.9856	1.02	0.8595	0.9636	0.79	0.0143	0.1589	0.97	0.7275	0.9889	0.92	0.3047	0.9607
AP-2 complex subunit alpha-1	AP2A1_HUMAN	O95782	1.09	0.2518	0.5219	1.04	0.5769	0.9982	0.96	0.6695	0.9960	1.12	0.1174	0.9874	1.07	0.3552	0.9951	1.00	0.9753	0.9967	0.92	0.3048	0.7378	1.06	0.4458	0.6635	1.03	0.7333	0.9889	1.05	0.5131	0.9846
AP-2 complex subunit beta	AP2B1_HUMAN	P63010	1.03	0.4362	0.6830	1.00	0.9179	0.9982	1.08	0.0922	0.9960	0.97	0.4830	0.9939	0.99	0.7746	0.9951	0.92	0.1163	0.9856	0.99	0.8411	0.9538	1.03	0.5673	0.7480	0.96	0.3445	0.9889	0.96	0.3746	0.9607
AP-2 complex subunit mu	AP2M1_HUMAN	Q96CW1	0.96	0.6243	0.8134	0.97	0.6910	0.9982	1.14	0.1973	0.9960	1.07	0.4531	0.9939	1.02	0.8540	0.9951	0.87	0.1929	0.9856	0.90	0.2628	0.7187	1.09	0.3882	0.6174	0.98	0.8383	0.9889	1.05	0.5756	0.9881
Apolipoprotein A-I	APOA1_HUMAN	P02647	0.88	0.2230	0.4953	1.04	0.7187	0.9982	1.09	0.4638	0.9960	0.89	0.2794	0.9939	0.96	0.6769	0.9951	0.88	0.3491	0.9856	1.21	0.0944	0.6824	0.81	0.0711	0.2734	1.03	0.7991	0.9889	0.96	0.6866	0.9881
Apolipoprotein A-II	APOA2_HUMAN	P02652	0.78	0.0527	0.2939	1.01	0.9170	0.9982	1.03	0.8204	0.9960	0.88	0.3403	0.9939	0.89	0.3822	0.9951	0.92	0.5983	0.9856	1.20	0.1797	0.7130	0.91	0.5358	0.7254	0.99	0.9694	0.9971	0.89	0.3695	0.9607
Apolipoprotein A-IV	APOA4_HUMAN	P06727	0.90	0.2797	0.5519	0.90	0.3116	0.9982	1.29	0.0273	0.9960	1.03	0.7758	0.9939	1.07	0.5101	0.9951	0.82	0.1246	0.9856	1.18	0.1334	0.6871	1.02	0.8928	0.9399	0.92	0.4139	0.9889	0.96	0.6668	0.9881
Apolipoprotein C-I	APOC1_HUMAN	P02654	0.73	0.0401	0.2873	1.15	0.3677	0.9982	1.00	0.9882	0.9960	0.91	0.5593	0.9939	0.86	0.3442	0.9951	0.90	0.5669	0.9856	1.21	0.2435	0.7187	0.58	0.0012	0.0763	1.05	0.7813	0.9889	0.94	0.7071	0.9881
Apolipoprotein C-II	APOC2_HUMAN	P02655	0.75	0.0279	0.2873	1.12	0.4033	0.9982	0.97	0.8592	0.9960	0.96	0.7369	0.9939	0.96	0.7568	0.9951	0.94	0.6951	0.9856	1.22	0.1690	0.6935	0.62	0.0010	0.0763	0.99	0.9303	0.9901	0.88	0.3435	0.9607
Apolipoprotein C-III	APOC3_HUMAN	P02656	0.81	0.0844	0.3334	1.18	0.1860	0.9982	1.16	0.3078	0.9960	1.13	0.3228	0.9939	1.01	0.9226	0.9951	0.84	0.2546	0.9856	1.33	0.0293	0.6824	0.73	0.0239	0.1763	1.03	0.8053	0.9889	0.95	0.6566	0.9881
Apolipoprotein D	APOD_HUMAN	P05090	0.85	0.1318	0.3958	0.95	0.6461	0.9982	1.16	0.2466	0.9960	1.06	0.6027	0.9939	0.93	0.5011	0.9951	0.76	0.0408	0.9411	1.22	0.0849	0.6824	0.79	0.0503	0.2382	0.89	0.2876	0.9889	0.82	0.0642	0.9607
Apolipoprotein E	APOE_HUMAN	P02649	0.88	0.3880	0.6461	1.28	0.0951	0.9982	0.88	0.4738	0.9960	0.86	0.3065	0.9939	0.97	0.8671	0.9951	1.00	0.9882	0.9992	1.21	0.2205	0.7187	0.60	0.0013	0.0763	1.13	0.4436	0.9889	0.93	0.6348	0.9881
Apolipoprotein L2	APOL2_HUMAN	Q9BQE5	1.05	0.5010	0.7246	1.02	0.7997	0.9982	1.04	0.6106	0.9960	1.23	0.0037	0.8235	1.05	0.5299	0.9951	0.92	0.3330	0.9856	1.07	0.3792	0.7749	0.98	0.8407	0.9140	0.95	0.5282	0.9889	0.98	0.7481	0.9881
Apoptosis-inducing factor 1, mitochondrial	AIFM1_HUMAN	O95831	1.11	0.0904	0.3428	1.01	0.8251	0.9982	1.00	0.9854	0.9960	0.98	0.7809	0.9939	0.97	0.6901	0.9951	1.01	0.8738	0.9959	0.91	0.1443	0.6871	1.12	0.0940	0.3118	1.02	0.8056	0.9889	1.02	0.8038	0.9881
Arginine-hydroxylase NDUFAF5, mitochondrial	NDUF5_HUMAN	Q5TEU4	1.14	0.1088	0.3697	0.98	0.7710	0.9982	0.98	0.8182	0.9960	0.92	0.3066	0.9939	0.99	0.8767	0.9951	1.07	0.4839	0.9856	0.85	0.0693	0.6824	1.24	0.0189	0.1650	1.13	0.1567	0.9889	1.02	0.7970	0.9881
Argininosuccinate synthase	ASSY_HUMAN	P00966	1.11	0.2129	0.4846	1.09	0.3205	0.9982	0.90	0.3062	0.9960	1.08	0.3946	0.9939	1.10	0.2993	0.9951	1.19	0.1100	0.9856	1.15	0.1398	0.6871	0.87	0.1509	0.4002	0.88	0.1567	0.9889	1.10	0.2938	0.9607
Armadillo repeat-containing protein 1	ARMC1_HUMAN	Q9NVT9	0.93	0.5114	0.7292	0.93	0.4923	0.9982	1.12	0.3963	0.9960	0.92	0.4431	0.9939	0.90	0.3558	0.9951	0.89	0.4153	0.9856	0.82	0.0987	0.6824	1.16	0.2241	0.4912	1.24	0.0627	0.9889	0.98	0.8574	0.9881
Asparagine--tRNA ligase, cytoplasmic	SYNC_HUMAN	O43776	1.06	0.4775	0.7142	1.04	0.6415	0.9982	0.94	0.5138	0.9960	1.09	0.3000	0.9939	1.10	0.2424	0.9951	1.09	0.3677	0.9856	1.02	0.8516	0.9580	1.00	0.9645	0.9796	0.89	0.1514	0.9889	1.02	0.8260	0.9881
Aspartate aminotransferase, cytoplasmic	AATC_HUMAN	P17174	1.22	0.0659	0.3105	0.98	0.8826	0.9982	0.90	0.4026	0.9960	0.89	0.2750	0.9939	1.01	0.9284	0.9951	1.24	0.1088	0.9856	0.82	0.0928	0.6824	1.22	0.1035	0.3276	0.97	0.7579	0.9889	1.06	0.5979	0.9881
Aspartate aminotransferase, mitochondrial	AATM_HUMAN	P00505	1.18	0.0509	0.2898	1.09	0.3432	0.9982	0.89	0.2672	0.9960	0.96	0.6550	0.9939	1.00	0.9868	0.9964	1.15	0.1941	0.9856	0.91	0.3134	0.7400	1.11	0.2733	0.5288	1.03	0.7328	0.9889	1.02	0.8201	0.9881
Aspartate--tRNA ligase, cytoplasmic	SYDC_HUMAN	P14868	1.08	0.4322	0.6798	1.16	0.1247	0.9982	0.96	0.7147	0.9960	1.30	0.0054	0.9874	1.18	0.0980	0.9951	1.07	0.5708	0.9856	1.18	0.1013	0.6824	0.86	0.1557	0.4055	1.09	0.4101	0.9889	1.13	0.1966	0.9607
Aspartate--tRNA ligase, mitochondrial	SYDM_HUMAN	Q6PI48	0.98	0.7816	0.8981	1.01	0.9334	0.9982	0.98	0.7434	0.9960	1.10	0.1649	0.9874	1.02	0.7637	0.9951	1.02	0.8552	0.9959	0.95	0.4346	0.8109	0.95	0.5340	0.7236	1.23	0.0022	0.9889	1.00	0.9401	0.9938
Aspartyl/asparaginyl beta-hydroxylase	ASPH_HUMAN	Q12797	0.77	0.0032	0.2005	0.98	0.8161	0.9982	0.98	0.8850	0.9960	1.03	0.7023	0.9939	0.92	0.3501	0.9951	0.99	0.9445	0.9959	1.26	0.0147	0.6779	0.89	0.2692	0.5288	0.87	0.1160	0.9889	0.83	0.0359	0.9607
Asporin	ASPN_HUMAN	Q9BXN1	0.75	0.2508	0.5219	1.00	0.9989	0.9989	0.63	0.1162	0.9960	0.96	0.8748	0.9939	0.87	0.6030	0.9951	1.35	0.3297	0.9856	1.22	0.4590	0.8243	0.52	0.0172	0.1636	1.31	0.2994	0.9889	1.03	0.9199	0.9938
Astrocytic phosphoprotein PEA-15	PEA15_HUMAN	Q15121	0.99	0.9484	0.9699	0.97	0.6935	0.9982	0.96	0.6643	0.9960	0.97	0.7254	0.9939	0.95	0.6030	0.9951	1.21	0.0743	0.9641	0.94	0.4761	0.8339	1.11	0.2609	0.5247	0.93	0.4174	0.9889	0.97	0.7004	0.9881
Atlastin-3	ATLA3_HUMAN	Q6DD88	0.84	0.0391	0.2873	1.01	0.8815	0.9982	1.03	0.7400	0.9960	0.94	0.4853	0.9939	0.89	0.1731	0.9951	0.93	0.4853	0.9856	1.12	0.1928	0.7149	0.82	0.0391	0.2155	0.93	0.4159	0.9889	0.93	0.4002	0.9607
ATP synthase F(0) complex subunit B1, mitochondrial	AT5F1_HUMAN	P24539	1.02	0.7657	0.8882	1.01	0.9174	0.9982	0.95	0.5678	0.9960	0.99	0.8769	0.9939	1.01	0.9446	0.9951	0.99	0.9313	0.9959	1.00	0.9569	0.9923	1.11	0.2175	0.4836	1.02	0.8367	0.9889	1.07	0.3369	0.9607
ATP synthase membrane subunit DAPIT, mitochondrial	ATPMD_HUMAN	Q96IX5	0.99	0.8801	0.9384	1.07	0.2974	0.9982	0.95	0.5493	0.9960	0.94	0.3763	0.9939	1.00	0.9824	0.9964	1.00	0.9597	0.9967	1.05	0.5296	0.8563	1.02	0.7660	0.8737	1.01	0.8477	0.9889	1.05	0.4669	0.9607
ATP synthase mitochondrial F1 complex assembly factor 1	ATPF1_HUMAN	Q5TC12	1.20	0.0661	0.3105	1.10	0.3581	0.9982	0.93	0.5421	0.9960	0.90	0.2858	0.9939	0.95	0.6083	0.9951	1.09	0.4807	0.9856	0.84	0.1035	0.6824	1.19	0.1217	0.3555	1.04	0.6780	0.9889	1.03	0.7884	0.9881
ATP synthase mitochondrial F1 complex assembly factor 2	ATPF2_HUMAN	Q8N5M1	1.26	0.0223	0.2873	1.05	0.6178	0.9982	0.84	0.1561	0.9960	1.03	0.7657	0.9939	1.04	0.7396	0.9951	1.15	0.2814	0.9856	0.97	0.8071	0.9460	1.17	0.1714	0.4222	1.05	0.6485	0.9889	1.05	0.6671	0.9881
ATP synthase protein 8	ATP8_HUMAN	P03928	0.92	0.2763	0.5477	1.03	0.7108	0.9982	0.87	0.1191	0.9960	0.94	0.4130	0.9939	0.88	0.1193	0.9951	1.10	0.3023	0.9856	1.03	0.7317	0.9218	1.05	0.5468	0.7334	1.03	0.7564	0.9889	0.97	0.6868	0.9881
ATP synthase subunit alpha, mitochondrial	ATPA_HUMAN	P25705	1.14	0.0820	0.3290	1.07	0.4098	0.9982	0.96	0.6520	0.9960	1.09	0.2472	0.9939	1.09	0.2765	0.9951	0.97	0.7632	0.9857	0.97	0.7077	0.9121	1.10	0.2686	0.5288	1.05	0.5186	0.9889	1.12	0.1345	0.9607
ATP synthase subunit beta, mitochondrial	ATPB_HUMAN	P06576	1.19	0.0163	0.2873	1.05	0.4874	0.9982	0.96	0.6457	0.9960	1.12	0.1025	0.9874	1.13	0.0937	0.9951	1.00	0.9679	0.9967	1.05	0.5211	0.8558	1.07	0.4042	0.6299	1.02	0.8093	0.9889	1.14	0.0624	0.9607
ATP synthase subunit d, mitochondrial	ATP5H_HUMAN	O75947	1.02	0.7495	0.8824	0.98	0.8015	0.9982	0.92	0.2777	0.9960	1.02	0.7857	0.9939	1.00	0.9812	0.9964	1.05	0.5705	0.9856	0.94	0.4345	0.8109	1.20	0.0127	0.1544	1.10	0.1647	0.9889	1.08	0.2322	0.9607
ATP synthase subunit delta, mitochondrial	ATPD_HUMAN	P30049	1.09	0.1763	0.4521	1.00	0.9886	0.9985	0.97	0.7221	0.9960	1.00	0.9480	0.9939	1.02	0.7432	0.9951	0.99	0.9399	0.9959	0.96	0.5983	0.8646	1.10	0.2018	0.4628	1.03	0.6570	0.9889	1.06	0.3869	0.9607
ATP synthase subunit e, mitochondrial	ATP5I_HUMAN	P56385	1.09	0.2070	0.4833	1.03	0.7158	0.9982	1.02	0.7613	0.9960	1.12	0.1113	0.9874	1.10	0.2059	0.9951	0.91	0.2636	0.9856	0.97	0.6641	0.8929	1.06	0.4700	0.6780	1.06	0.4399	0.9889	1.13	0.0762	0.9607
ATP synthase subunit epsilon, mitochondrial	ATP5E_HUMAN	P56381	1.13	0.0996	0.3570	1.05	0.5211	0.9982	0.93	0.3596	0.9960	1.04	0.5556	0.9939	1.05	0.5453	0.9951	1.07	0.4519	0.9856	0.98	0.8122	0.9478	1.11	0.1947	0.4557	1.07	0.3423	0.9889	1.13	0.0848	0.9607
ATP synthase subunit f, mitochondrial	ATPK_HUMAN	P56134	1.02	0.8260	0.9186	1.09	0.2926	0.9982	0.99	0.9181	0.9960	1.07	0.4096	0.9939	1.01	0.8909	0.9951	0.93	0.4697	0.9856	0.96	0.6465	0.8897	1.02	0.8645	0.9252	1.12	0.2071	0.9889	1.06	0.4648	0.9607
ATP synthase subunit g, mitochondrial	ATP5L_HUMAN	O75964	1.06	0.4499	0.6950	1.00	0.9882	0.9985	0.94	0.4254	0.9960	1.07	0.3634	0.9939	1.07	0.3671	0.9951	1.02	0.8365	0.9959	1.03	0.6635	0.8929	1.13	0.1163	0.3468	1.03	0.6984	0.9889	1.10	0.1776	0.9607
ATP synthase subunit gamma, mitochondrial	ATPG_HUMAN	P36542	1.07	0.4140	0.6659	1.08	0.3496	0.9982	0.91	0.3589	0.9960	1.04	0.6385	0.9939	1.05	0.5868	0.9951	1.02	0.8537	0.9959	1.00	0.9579	0.9923	1.04	0.6809	0.8218	1.07	0.4387	0.9889	1.10	0.2687	0.9607
ATP synthase subunit O, mitochondrial	ATPO_HUMAN	P48047	1.10	0.1664	0.4371	1.03	0.7166	0.9982	0.95	0.5064	0.9960	1.05	0.4508	0.9939	1.09	0.2522	0.9951	1.01	0.9431	0.9959	1.00	0.9991	0.9992	1.09	0.2707	0.5288	1.05	0.4712	0.9889	1.12	0.0882	0.9607
ATP synthase subunit s, mitochondrial	ATP5S_HUMAN	Q99766	1.09	0.2630	0.5333	0.96	0.6426	0.9982	0.96	0.6574	0.9960	0.96	0.5561	0.9939	0.95	0.5294	0.9951	1.03	0.7844	0.9897	0.92	0.2871	0.7212	1.31	0.0014	0.0763	1.05	0.5747	0.9889	1.01	0.9382	0.9938
ATP synthase-coupling factor 6, mitochondrial	ATP5J_HUMAN	P18859	1.06	0.4634	0.7039	0.99	0.8653	0.9982	0.93	0.4401	0.9960	1.03	0.7125	0.9939	0.98	0.7872	0.9951	1.04	0.6812	0.9856	0.94	0.4382	0.8116	1.18	0.0451	0.2257	1.05	0.5200	0.9889	1.05	0.5351	0.9881
ATP-binding cassette sub-family A member 8	ABCA8_HUMAN	O94911	0.82	0.0395	0.2873	1.13	0.2147	0.9982	1.05	0.6627	0.9960	0.91	0.3276	0.9939	0.76	0.0050	0.9951	0.90	0.3663	0.9856	1.06	0.5416	0.8573	0.90	0.3049	0.5503	0.88	0.2110	0.9889	0.76	0.0036	0.9304
ATP-dependent (S)-NAD(P)H-hydrate dehydratase	NNRD_HUMAN	Q8IW45	1.13	0.1254	0.3922	0.98	0.7529	0.9982	0.92	0.3657	0.9960	0.95	0.5291	0.9939	0.98	0.8511	0.9951	1.23	0.0295	0.9411	0.89	0.1650	0.6896	1.24	0.0151	0.1606	0.99	0.9019	0.9889	1.05	0.5132	0.9846
ATP-dependent 6-phosphofructokinase, liver type	PFKAL_HUMAN	P17858	1.00	0.9876	0.9915	1.05	0.6110	0.9982	1.10	0.3504	0.9960	1.08	0.3833	0.9939	1.06	0.5607	0.9951	0.92	0.4548	0.9856	1.02	0.8034	0.9456	0.86	0.1356	0.3764	0.96	0.6414	0.9889	0.98	0.8567	0.9881
ATP-dependent 6-phosphofructokinase, muscle type	PFKAM_HUMAN	P08237	1.07	0.3560	0.6186	1.09	0.2667	0.9982	1.05	0.5469	0.9960	1.01	0.9139	0.9939	0.98	0.8204	0.9951	0.96	0.6371	0.9856	0.97	0.6692	0.8929	1.10	0.2670	0.5288	1.00	0.9876	0.9976	1.01	0.8883	0.9938
ATP-dependent 6-phosphofructokinase, platelet type	PFKAP_HUMAN	Q01813	1.02	0.7898	0.8986	1.03	0.6602	0.9982	1.01	0.8328	0.9960	1.06	0.3466	0.9939	1.01	0.9326	0.9951	0.99	0.9442	0.9959	1.02	0.7942	0.9431	1.04	0.5721	0.7504	1.06	0.3225	0.9889	1.00	0.9465	0.9938
ATP-dependent Clp protease proteolytic subunit, mitochondrial	CLPP_HUMAN	Q16740	1.09	0.0953	0.3548	1.04	0.4869	0.9982	0.89	0.0565	0.9960	1.02	0.7227	0.9939	1.05	0.3791	0.9951	1.12	0.0947	0.9856	0.97	0.5699	0.8607	1.02	0.7854	0.8852	1.01	0.8650	0.9889	1.03	0.5647	0.9881
ATP-dependent RNA helicase A	DHX9_HUMAN	Q08211	0.83	0.1860	0.4611	1.19	0.2055	0.9982	1.01	0.9726	0.9960	0.96	0.7893	0.9939	0.84	0.2386	0.9951	0.98	0.9200	0.9959	1.16	0.3205	0.7400	0.87	0.3665	0.5972	0.93	0.6402	0.9889	0.77	0.0652	0.9607
ATP-dependent RNA helicase DDX1	DDX1_HUMAN	Q92499	0.91	0.0622	0.3044	0.97	0.5693	0.9982	1.09	0.1161	0.9960	0.99	0.7924	0.9939	0.98	0.7120	0.9951	0.93	0.2077	0.9856	1.05	0.3265	0.7401	0.97	0.6298	0.7896	0.98	0.6411	0.9889	0.99	0.8002	0.9881
ATPase ASNA1	ASNA_HUMAN	O43681	1.06	0.1943	0.4726	0.99	0.8774	0.9982	1.04	0.3744	0.9960	0.99	0.8712	0.9939	1.03	0.5682	0.9951	0.97	0.5631	0.9856	0.95	0.2952	0.7286	1.01	0.8750	0.9313	0.96	0.2899	0.9889	1.02	0.7198	0.9881
ATPase family AAA domain-containing protein 1	ATAD1_HUMAN	Q8NBU5	1.11	0.3600	0.6228	1.14	0.2528	0.9982	0.92	0.5185	0.9960	1.22	0.0690	0.9874	1.22	0.0911	0.9951	1.06	0.6585	0.9856	1.01	0.9411	0.9903	0.89	0.3390	0.5744	1.10	0.3959	0.9889	1.21	0.0795	0.9607
ATPase family AAA domain-containing protein 3A	ATD3A_HUMAN	Q9NVI7	1.07	0.2700	0.5390	1.02	0.7278	0.9982	0.96	0.5581	0.9960	1.03	0.6294	0.9939	1.10	0.1317	0.9951	1.01	0.8871	0.9959	1.03	0.6698	0.8929	1.07	0.3053	0.5503	0.97	0.6818	0.9889	1.09	0.1523	0.9607
ATPase family AAA domain-containing protein 3B	ATD3B_HUMAN	Q5T9A4	0.86	0.0269	0.2873	1.05	0.4911	0.9982	0.93	0.3564	0.9960	1.02	0.7802	0.9939	1.00	0.9661	0.9951	1.04	0.6349	0.9856	1.14	0.0705	0.6824	0.88	0.0868	0.2982	1.06	0.4168	0.9889	1.00	0.9482	0.9938
ATPase inhibitor, mitochondrial	ATIF1_HUMAN	Q9UII2	1.09	0.2115	0.4843	1.00	0.9531	0.9982	0.91	0.2439	0.9960	0.98	0.7405	0.9939	1.00	0.9606	0.9951	1.11	0.1908	0.9856	0.93	0.3069	0.7378	1.21	0.0089	0.1369	0.99	0.8459	0.9889	0.97	0.6831	0.9881
Atypical kinase COQ8A, mitochondrial	COQ8A_HUMAN	Q8NI60	1.12	0.0816	0.3290	1.05	0.4463	0.9982	0.95	0.4636	0.9960	1.10	0.1326	0.9874	1.05	0.4735	0.9951	1.00	0.9656	0.9967	1.09	0.2281	0.7187	1.03	0.6470	0.7998	1.05	0.5018	0.9889	1.07	0.2883	0.9607
B-cell receptor-associated protein 29	BAP29_HUMAN	Q9UHQ4	0.82	0.0096	0.2530	0.91	0.2027	0.9982	1.03	0.7418	0.9960	1.03	0.7460	0.9939	0.92	0.3203	0.9951	0.96	0.6561	0.9856	1.06	0.4805	0.8352	1.02	0.7766	0.8813	0.93	0.3263	0.9889	0.93	0.3637	0.9607
B-cell receptor-associated protein 31	BAP31_HUMAN	P51572	0.83	0.0265	0.2873	1.07	0.4480	0.9982	1.01	0.9602	0.9960	0.94	0.4354	0.9939	0.96	0.6248	0.9951	0.98	0.8404	0.9959	1.03	0.7281	0.9213	0.85	0.0905	0.3039	1.01	0.9449	0.9922	0.95	0.5311	0.9881
BAG family molecular chaperone regulator 2	BAG2_HUMAN	O95816	0.86	0.0538	0.2939	1.00	0.9839	0.9985	0.99	0.9258	0.9960	1.02	0.7723	0.9939	0.95	0.5230	0.9951	0.95	0.6350	0.9856	1.10	0.2501	0.7187	0.85	0.0569	0.2478	1.06	0.4930	0.9889	0.96	0.6407	0.9881
BAG family molecular chaperone regulator 3	BAG3_HUMAN	O95817	0.98	0.6735	0.8415	1.01	0.8984	0.9982	0.98	0.7800	0.9960	0.96	0.4880	0.9939	0.96	0.4952	0.9951	1.04	0.5796	0.9856	0.91	0.1036	0.6824	1.02	0.7508	0.8627	1.10	0.0648	0.9889	1.05	0.3962	0.9607
Band 4.1-like protein 2	E41L2_HUMAN	O43491	0.86	0.0359	0.2873	0.95	0.4915	0.9982	1.04	0.6418	0.9960	0.96	0.5788	0.9939	0.91	0.2105	0.9951	1.00	0.9707	0.9967	1.10	0.2222	0.7187	0.86	0.0553	0.2467	0.96	0.5892	0.9889	0.92	0.2250	0.9607
Barrier-to-autointegration factor	BAF_HUMAN	O75531	0.80	0.0204	0.2873	1.02	0.8537	0.9982	1.09	0.4566	0.9960	0.85	0.0936	0.9874	0.74	0.0029	0.9389	0.90	0.3930	0.9856	0.96	0.7002	0.9088	0.91	0.4018	0.6294	1.08	0.4571	0.9889	0.85	0.1022	0.9607
Basal cell adhesion molecule	BCAM_HUMAN	P50895	0.99	0.8362	0.9201	1.05	0.4303	0.9982	0.98	0.8109	0.9960	0.99	0.9306	0.9939	1.02	0.7516	0.9951	0.97	0.7378	0.9857	1.08	0.2610	0.7187	0.96	0.5379	0.7261	0.97	0.6260	0.9889	0.97	0.6827	0.9881
Basement membrane-specific heparan sulfate proteoglycan core protein	PGBM_HUMAN	P98160	0.87	0.0560	0.2942	1.00	0.9893	0.9985	1.10	0.2660	0.9960	0.96	0.5240	0.9939	0.88	0.0794	0.9951	0.95	0.5746	0.9856	1.02	0.7951	0.9431	0.87	0.0883	0.2997	1.01	0.9224	0.9889	0.89	0.1036	0.9607
Basic leucine zipper and W2 domain-containing protein 2	BZW2_HUMAN	Q9Y6E2	1.05	0.4191	0.6686	0.95	0.3971	0.9982	1.00	0.9734	0.9960	0.97	0.6940	0.9939	1.03	0.6261	0.9951	1.00	0.9584	0.9967	0.95	0.5019	0.8476	1.12	0.1277	0.3636	1.02	0.7716	0.9889	1.06	0.3723	0.9607
Basigin	BASI_HUMAN	P35613	0.94	0.3990	0.6574	0.90	0.1553	0.9982	1.14	0.1072	0.9960	1.04	0.5641	0.9939	1.04	0.5632	0.9951	0.81	0.0166	0.9411	1.05	0.4806	0.8352	0.88	0.1151	0.3456	0.90	0.1684	0.9889	0.95	0.4377	0.9607
Bcl-2-like protein 13	B2L13_HUMAN	Q9BXK5	1.17	0.0885	0.3410	1.06	0.5195	0.9982	0.92	0.4235	0.9960	0.85	0.0839	0.9874	1.01	0.9344	0.9951	1.06	0.5991	0.9856	0.80	0.0211	0.6804	1.01	0.9269	0.9583	0.94	0.4897	0.9889	1.00	0.9848	0.9992
Beta-2-glycoprotein 1	APOH_HUMAN	P02749	0.88	0.2393	0.5133	1.11	0.3732	0.9982	0.98	0.8897	0.9960	0.94	0.6156	0.9939	0.84	0.1458	0.9951	1.01	0.9609	0.9967	1.08	0.5403	0.8573	0.84	0.1693	0.4217	0.95	0.6789	0.9889	0.84	0.1288	0.9607
Beta-2-microglobulin	B2MG_HUMAN	P61769	0.95	0.6258	0.8147	0.94	0.5475	0.9982	1.21	0.1187	0.9960	0.98	0.8465	0.9939	1.04	0.7031	0.9951	0.87	0.2692	0.9856	1.17	0.1513	0.6871	0.79	0.0475	0.2309	0.93	0.5117	0.9889	1.02	0.8213	0.9881
Beta-enolase	ENOB_HUMAN	P13929	0.99	0.9432	0.9668	0.98	0.8365	0.9982	1.00	0.9685	0.9960	0.93	0.4241	0.9939	0.91	0.2773	0.9951	1.02	0.8756	0.9959	0.85	0.0721	0.6824	1.22	0.0366	0.2073	1.04	0.6701	0.9889	0.98	0.8562	0.9881
Beta-hexosaminidase subunit alpha	HEXA_HUMAN	P06865	1.04	0.6515	0.8297	1.05	0.5838	0.9982	1.11	0.2973	0.9960	1.04	0.6716	0.9939	0.96	0.6326	0.9951	0.86	0.1650	0.9856	0.99	0.8906	0.9753	1.12	0.2472	0.5122	0.91	0.3144	0.9889	0.92	0.3447	0.9607
Beta-hexosaminidase subunit beta	HEXB_HUMAN	P07686	1.12	0.1054	0.3654	0.94	0.3618	0.9982	1.16	0.0746	0.9960	1.06	0.4239	0.9939	1.08	0.3225	0.9951	0.82	0.0248	0.9411	1.09	0.2779	0.7212	1.03	0.7429	0.8595	0.93	0.3119	0.9889	0.97	0.6723	0.9881
Beta-sarcoglycan	SGCB_HUMAN	Q16585	0.95	0.4282	0.6776	0.96	0.5497	0.9982	1.11	0.2078	0.9960	1.03	0.6616	0.9939	1.03	0.6668	0.9951	0.89	0.1596	0.9856	1.02	0.7568	0.9282	0.83	0.0130	0.1544	0.95	0.4744	0.9889	1.00	0.9675	0.9985
Beta-taxilin	TXLNB_HUMAN	Q8N3L3	1.04	0.6308	0.8170	0.97	0.6319	0.9982	1.02	0.8198	0.9960	1.14	0.0557	0.9874	1.12	0.1384	0.9951	1.00	0.9557	0.9967	0.95	0.4932	0.8421	1.10	0.2127	0.4786	0.99	0.9176	0.9889	1.02	0.7569	0.9881
Beta,beta-carotene 9',10'-oxygenase	BCDO2_HUMAN	Q9BYV7	0.90	0.3117	0.5811	0.91	0.3642	0.9982	1.11	0.3786	0.9960	0.91	0.3622	0.9939	0.96	0.7135	0.9951	0.88	0.3030	0.9856	0.83	0.0955	0.6824	1.09	0.4507	0.6657	0.91	0.3556	0.9889	1.00	0.9619	0.9956
Bifunctional epoxide hydrolase 2	HYES_HUMAN	P34913	1.01	0.8705	0.9356	1.00	0.9699	0.9982	0.97	0.7576	0.9960	1.05	0.5836	0.9939	1.14	0.1517	0.9951	1.01	0.9387	0.9959	1.04	0.6610	0.8929	0.97	0.7852	0.8852	0.99	0.8872	0.9889	1.00	0.9834	0.9992
Bifunctional glutamate/proline--tRNA ligase	SYEP_HUMAN	P07814	0.89	0.1310	0.3958	0.99	0.9277	0.9982	1.17	0.0754	0.9960	1.02	0.8090	0.9939	0.99	0.8848	0.9951	0.87	0.1328	0.9856	1.00	0.9884	0.9992	1.12	0.1807	0.4355	1.11	0.1858	0.9889	0.99	0.9395	0.9938
Bifunctional purine biosynthesis protein PURH	PUR9_HUMAN	P31939	1.18	0.0960	0.3559	0.99	0.9266	0.9982	0.96	0.7393	0.9960	0.93	0.4722	0.9939	1.03	0.7968	0.9951	1.16	0.2221	0.9856	0.91	0.3890	0.7835	1.08	0.5047	0.7017	0.89	0.2677	0.9889	1.03	0.7632	0.9881
Biglycan	PGS1_HUMAN	P21810	0.65	0.0512	0.2898	1.05	0.8199	0.9982	0.71	0.1764	0.9960	0.91	0.6685	0.9939	0.82	0.3790	0.9951	1.21	0.4878	0.9856	1.15	0.5646	0.8601	0.53	0.0084	0.1361	1.18	0.4776	0.9889	0.96	0.8600	0.9881
Bis(5'-adenosyl)-triphosphatase	FHIT_HUMAN	P49789	1.31	0.0028	0.2005	1.05	0.6294	0.9982	1.02	0.8536	0.9960	0.93	0.4373	0.9939	1.07	0.4543	0.9951	0.97	0.7941	0.9923	0.86	0.1232	0.6868	1.13	0.2474	0.5122	0.99	0.8835	0.9889	1.07	0.4782	0.9632
Bis(5'-adenosyl)-triphosphatase ENPP4	ENPP4_HUMAN	Q9Y6X5	1.04	0.5459	0.7505	1.01	0.8789	0.9982	1.12	0.0931	0.9960	1.02	0.6834	0.9939	1.02	0.7913	0.9951	0.85	0.0221	0.9411	0.96	0.5230	0.8558	1.04	0.5490	0.7350	0.99	0.8366	0.9889	1.07	0.2781	0.9607
Bleomycin hydrolase	BLMH_HUMAN	Q13867	1.00	0.9848	0.9915	0.95	0.3939	0.9982	0.94	0.4069	0.9960	1.00	0.9974	0.9993	0.99	0.8395	0.9951	1.10	0.2338	0.9856	0.97	0.6441	0.8897	1.14	0.0644	0.2631	0.93	0.2808	0.9889	0.98	0.7788	0.9881
Blood vessel epicardial substance	POPD1_HUMAN	Q8NE79	1.04	0.6658	0.8376	0.91	0.2511	0.9982	1.18	0.0868	0.9960	1.06	0.4903	0.9939	1.15	0.1162	0.9951	0.78	0.0167	0.9411	0.94	0.5175	0.8554	0.99	0.9342	0.9616	0.95	0.5534	0.9889	1.08	0.3387	0.9607
Branched-chain-amino-acid aminotransferase, mitochondrial	BCAT2_HUMAN	O15382	1.19	0.0350	0.2873	1.08	0.3572	0.9982	0.95	0.6016	0.9960	0.99	0.8822	0.9939	1.02	0.8609	0.9951	1.03	0.7514	0.9857	0.90	0.2219	0.7187	1.07	0.4549	0.6689	1.04	0.6593	0.9889	1.06	0.4722	0.9607
BTB/POZ domain-containing protein KCTD12	KCD12_HUMAN	Q96CX2	1.02	0.8254	0.9186	1.03	0.7286	0.9982	1.10	0.3002	0.9960	0.98	0.8317	0.9939	0.94	0.4788	0.9951	0.95	0.5905	0.9856	0.96	0.5990	0.8646	0.93	0.4354	0.6555	0.93	0.3829	0.9889	0.91	0.2566	0.9607
C->U-editing enzyme APOBEC-2	ABEC2_HUMAN	Q9Y235	1.02	0.7651	0.8882	1.13	0.1254	0.9982	1.06	0.5270	0.9960	1.12	0.1679	0.9874	0.89	0.1665	0.9951	0.95	0.6406	0.9856	0.98	0.8240	0.9482	1.10	0.3093	0.5510	1.04	0.5944	0.9889	0.92	0.2974	0.9607
C-1-tetrahydrofolate synthase, cytoplasmic	C1TC_HUMAN	P11586	1.03	0.6810	0.8455	1.00	0.9632	0.9982	1.02	0.8151	0.9960	1.00	0.9738	0.9961	0.98	0.8096	0.9951	1.02	0.8404	0.9959	1.05	0.5190	0.8558	1.01	0.8863	0.9377	0.94	0.3901	0.9889	0.98	0.7493	0.9881
C-Jun-amino-terminal kinase-interacting protein 4	JIP4_HUMAN	O60271	1.01	0.9015	0.9479	1.05	0.3059	0.9982	0.98	0.7044	0.9960	1.04	0.4018	0.9939	0.95	0.2915	0.9951	0.98	0.7815	0.9897	1.03	0.6003	0.8646	0.93	0.1710	0.4219	1.04	0.4486	0.9889	1.01	0.8788	0.9938
C-type mannose receptor 2	MRC2_HUMAN	Q9UBG0	0.85	0.1955	0.4726	1.02	0.8500	0.9982	0.93	0.6163	0.9960	1.13	0.3165	0.9939	0.97	0.8120	0.9951	1.16	0.3379	0.9856	1.22	0.1266	0.6868	0.74	0.0259	0.1832	0.98	0.8873	0.9889	0.98	0.8780	0.9938
Cadherin-13	CAD13_HUMAN	P55290	1.01	0.8308	0.9186	0.95	0.4371	0.9982	0.94	0.4190	0.9960	1.08	0.2354	0.9939	1.00	0.9682	0.9951	1.04	0.6444	0.9856	1.06	0.3852	0.7810	1.02	0.8014	0.8919	0.96	0.5613	0.9889	1.01	0.8400	0.9881

Cadherin-2	CADH2_HUMAN	P19022	1.01	0.9228	0.9555	0.95	0.3753	0.9982	1.06	0.3880	0.9960	0.99	0.8142	0.9939	1.03	0.6197	0.9951	0.90	0.1334	0.9856	0.93	0.2649	0.7187	1.00	0.9486	0.9708	1.02	0.6933	0.9889	1.05	0.3535	0.9607
Cadherin-5	CADH5_HUMAN	P33151	1.06	0.2465	0.5201	0.96	0.4015	0.9982	0.99	0.8571	0.9960	1.00	0.9819	0.9969	1.10	0.0496	0.9951	1.03	0.6645	0.9856	1.01	0.8293	0.9494	1.00	0.9682	0.9827	0.94	0.1931	0.9889	1.03	0.5152	0.9846
Calcineurin B homologous protein 1	CHP1_HUMAN	Q99653	1.00	0.9687	0.9819	0.95	0.3881	0.9982	1.00	0.9886	0.9960	0.99	0.8474	0.9939	0.99	0.8332	0.9951	0.97	0.6572	0.9856	0.93	0.2338	0.7187	1.00	0.9800	0.9889	0.98	0.7495	0.9889	1.02	0.6975	0.9881
Calcium-binding mitochondrial carrier protein Aralar1	CMC1_HUMAN	O75746	1.16	0.0439	0.2873	1.10	0.1970	0.9982	0.95	0.5403	0.9960	1.07	0.3915	0.9939	1.11	0.1787	0.9951	0.99	0.9335	0.9959	0.99	0.9054	0.9840	1.07	0.4396	0.6595	1.10	0.2401	0.9889	1.15	0.0572	0.9607
Calcium-binding mitochondrial carrier protein SCaMC-1	SCMC1_HUMAN	Q6NUK1	0.98	0.7691	0.8891	1.06	0.3440	0.9982	1.02	0.8095	0.9960	1.06	0.3576	0.9939	1.04	0.4991	0.9951	0.97	0.6772	0.9856	1.00	0.9897	0.9992	0.95	0.4896	0.6911	0.92	0.1701	0.9889	0.98	0.7479	0.9881
Calcium/calmodulin-dependent protein kinase type II subunit delta	KCC2D_HUMAN	Q13557	0.98	0.7232	0.8735	0.94	0.3720	0.9982	1.09	0.2717	0.9960	1.02	0.7757	0.9939	1.02	0.7152	0.9951	0.97	0.7285	0.9857	0.98	0.7353	0.9218	1.25	0.0013	0.0763	1.05	0.4714	0.9889	1.03	0.5987	0.9881
Caldesmon	CALD1_HUMAN	Q05682	0.88	0.1267	0.3932	0.98	0.8035	0.9982	0.95	0.6148	0.9960	0.90	0.2121	0.9939	0.87	0.0999	0.9951	1.11	0.2990	0.9856	0.95	0.5633	0.8601	0.93	0.4039	0.6299	0.97	0.7437	0.9889	0.94	0.4500	0.9607
Calmegin	CLGN_HUMAN	O14967	0.81	0.0025	0.2005	0.96	0.5573	0.9982	1.13	0.1583	0.9960	0.88	0.0881	0.9874	0.83	0.0119	0.9951	0.99	0.8948	0.9959	0.93	0.3832	0.7787	1.04	0.6468	0.7998	1.03	0.7070	0.9889	0.92	0.2617	0.9607
Calmodulin-1	CALM1_HUMAN	P0DP23	0.95	0.3916	0.6493	0.99	0.8351	0.9982	0.93	0.3500	0.9960	0.96	0.5082	0.9939	0.93	0.3004	0.9951	1.07	0.3618	0.9856	0.91	0.1654	0.6896	1.01	0.8708	0.9284	0.92	0.2002	0.9889	0.92	0.1950	0.9607
Calnexin	CALX_HUMAN	P27824	0.84	0.0023	0.2005	0.94	0.2581	0.9982	1.05	0.5060	0.9960	1.02	0.6978	0.9939	0.97	0.6446	0.9951	0.93	0.3091	0.9856	1.07	0.2746	0.7196	0.95	0.4756	0.6794	0.97	0.6111	0.9889	0.97	0.5882	0.9881
Calpain small subunit 1	CPNS1_HUMAN	P04632	0.99	0.8703	0.9356	0.99	0.8170	0.9982	0.99	0.8701	0.9960	0.90	0.1111	0.9874	0.90	0.1161	0.9951	1.05	0.5183	0.9856	0.84	0.0096	0.6779	1.17	0.0229	0.1743	0.97	0.6591	0.9889	0.93	0.2313	0.9607
Calpain-1 catalytic subunit	CAN1_HUMAN	P07384	1.06	0.3733	0.6312	1.10	0.1218	0.9982	1.03	0.6677	0.9960	0.96	0.5092	0.9939	0.93	0.2212	0.9951	1.07	0.3794	0.9856	0.87	0.0353	0.6824	1.15	0.0437	0.2215	1.02	0.7080	0.9889	0.95	0.4199	0.9607
Calpain-2 catalytic subunit	CAN2_HUMAN	P17655	1.02	0.7430	0.8802	0.93	0.1655	0.9982	1.01	0.9178	0.9960	0.92	0.1171	0.9874	0.97	0.5387	0.9951	1.01	0.8364	0.9959	0.97	0.6477	0.8897	1.06	0.3421	0.5771	0.93	0.2148	0.9889	0.97	0.6141	0.9881
Calpastatin	ICAL_HUMAN	P20810	1.02	0.7891	0.8986	0.99	0.9106	0.9982	0.95	0.4983	0.9960	0.98	0.8078	0.9939	0.93	0.3046	0.9951	1.15	0.1186	0.9856	0.89	0.1113	0.6824	1.16	0.0613	0.2551	1.02	0.8135	0.9889	0.95	0.5044	0.9818
Calponin-1	CNN1_HUMAN	P51911	1.06	0.6376	0.8225	1.06	0.6857	0.9982	0.89	0.4363	0.9960	0.81	0.1012	0.9874	0.86	0.2612	0.9951	1.23	0.1993	0.9856	0.99	0.9180	0.9848	0.94	0.6612	0.8074	0.91	0.4935	0.9889	1.02	0.8590	0.9881
Calreticulin	CALR_HUMAN	P27797	0.96	0.4594	0.7012	0.97	0.5986	0.9982	1.06	0.3837	0.9960	1.01	0.8490	0.9939	0.96	0.5667	0.9951	0.95	0.4945	0.9856	0.99	0.8931	0.9765	0.98	0.7188	0.8395	0.90	0.0862	0.9889	0.88	0.0425	0.9607
Calsequestrin-2	CASQ2_HUMAN	O14958	1.09	0.4004	0.6584	0.94	0.5217	0.9982	1.15	0.2265	0.9960	0.98	0.8536	0.9939	0.94	0.5269	0.9951	0.83	0.1221	0.9856	0.83	0.0765	0.6824	1.29	0.0159	0.1634	0.98	0.8713	0.9889	0.93	0.4691	0.9607
Calumenin	CALU_HUMAN	O43852	0.91	0.2309	0.5050	0.93	0.3694	0.9982	0.97	0.6900	0.9960	0.98	0.7513	0.9939	0.91	0.2412	0.9951	1.06	0.5397	0.9856	1.06	0.4429	0.8171	1.01	0.8883	0.9384	0.92	0.3009	0.9889	0.91	0.2138	0.9607
cAMP-dependent protein kinase catalytic subunit alpha	KAPCA_HUMAN	P17612	1.04	0.4921	0.7220	0.97	0.5664	0.9982	0.98	0.7888	0.9960	1.02	0.7598	0.9939	1.01	0.9010	0.9951	1.01	0.8392	0.9959	1.05	0.3461	0.7593	1.13	0.0332	0.2017	0.95	0.3045	0.9889	0.99	0.8456	0.9881
cAMP-dependent protein kinase type I-alpha regulatory subunit	KAP0_HUMAN	P10644	1.12	0.1602	0.4314	1.05	0.5657	0.9982	0.94	0.4818	0.9960	1.02	0.8358	0.9939	0.99	0.9096	0.9951	1.15	0.1500	0.9856	0.93	0.4330	0.8109	1.20	0.0449	0.2255	0.92	0.3379	0.9889	0.97	0.6778	0.9881
cAMP-dependent protein kinase type II-alpha regulatory subunit	KAP2_HUMAN	P13861	1.16	0.0100	0.2530	1.03	0.5798	0.9982	0.94	0.3691	0.9960	1.00	0.9914	0.9981	1.09	0.1688	0.9951	1.05	0.5445	0.9856	1.00	0.9571	0.9923	1.05	0.4609	0.6738	0.97	0.5793	0.9889	1.09	0.1577	0.9607
cAMP-dependent protein kinase type II-beta regulatory subunit	KAP3_HUMAN	P31323	1.03	0.7285	0.8741	1.02	0.8183	0.9982	1.04	0.6785	0.9960	1.19	0.0339	0.9874	1.11	0.2098	0.9951	1.01	0.9207	0.9959	1.15	0.1106	0.6824	1.02	0.8406	0.9140	1.04	0.6131	0.9889	1.11	0.2058	0.9607
Caprin-1	CAPR1_HUMAN	Q14444	0.99	0.8447	0.9230	0.97	0.5709	0.9982	1.01	0.8612	0.9960	1.01	0.7687	0.9939	1.01	0.7862	0.9951	1.03	0.5761	0.9856	0.97	0.6142	0.8741	1.13	0.0230	0.1743	1.01	0.7807	0.9889	1.00	0.9940	0.9992
Carbonic anhydrase 1	CAH1_HUMAN	P00915	1.09	0.4127	0.6652	0.93	0.5045	0.9982	1.08	0.5301	0.9960	0.96	0.6836	0.9939	1.15	0.1679	0.9951	1.01	0.9378	0.9959	1.09	0.4110	0.7917	1.16	0.1947	0.4557	1.02	0.8748	0.9889	1.13	0.2164	0.9607
Carbonic anhydrase 2	CAH2_HUMAN	P00918	1.10	0.3736	0.6312	0.93	0.5125	0.9982	1.08	0.5382	0.9960	0.85	0.1321	0.9874	1.04	0.7098	0.9951	1.00	0.9967	0.9992	1.04	0.7674	0.9291	1.16	0.2156	0.4816	0.93	0.4961	0.9889	0.90	0.3410	0.9607
Carbonic anhydrase 3	CAH3_HUMAN	P07451	0.86	0.5246	0.7378	1.18	0.4785	0.9982	0.80	0.3991	0.9960	1.09	0.7182	0.9939	1.05	0.8515	0.9951	0.96	0.8952	0.9959	1.48	0.1055	0.6824	1.26	0.3690	0.5979	1.13	0.6114	0.9889	0.76	0.2343	0.9607
Carbonyl reductase [NADPH] 1	CBR1_HUMAN	P16152	1.18	0.0109	0.2535	1.07	0.3327	0.9982	0.96	0.5515	0.9960	1.03	0.6287	0.9939	1.10	0.1741	0.9951	1.10	0.2378	0.9856	1.00	0.9599	0.9924	1.00	0.9822	0.9891	1.01	0.8327	0.9889	1.04	0.5900	0.9881
Carbonyl reductase family member 4	CBR4_HUMAN	Q8N4T8	1.06	0.4875	0.7214	1.03	0.6990	0.9982	0.99	0.8974	0.9960	1.00	0.9621	0.9947	0.94	0.4443	0.9951	1.03	0.7581	0.9857	0.90	0.2351	0.7187	1.22	0.0233	0.1754	1.19	0.0336	0.9889	0.94	0.4070	0.9607
Carboxymethylenebutenolidase homolog	CMBL_HUMAN	Q96DG6	1.14	0.2482	0.5210	0.97	0.7778	0.9982	1.03	0.8467	0.9960	0.92	0.4819	0.9939	0.99	0.9580	0.9951	0.98	0.9030	0.9959	0.77	0.0269	0.6824	1.39	0.0098	0.1402	1.02	0.8755	0.9889	1.00	0.9723	0.9992
Cardiac phospholamban	PPLA_HUMAN	P26678	1.08	0.3953	0.6526	1.11	0.2536	0.9982	1.01	0.9090	0.9960	1.14	0.1601	0.9874	0.99	0.8792	0.9951	0.95	0.6549	0.9856	0.99	0.9187	0.9848	1.08	0.4523	0.6671	1.01	0.8989	0.9889	0.99	0.9497	0.9938
Cardiomyopathy-associated protein 5	CMYA5_HUMAN	Q8N3K9	1.02	0.8255	0.9186	0.96	0.6079	0.9982	1.05	0.6441	0.9960	0.95	0.5373	0.9939	0.99	0.8711	0.9951	1.02	0.8373	0.9959	0.82	0.0189	0.6779	1.29	0.0043	0.1244	1.04	0.6482	0.9889	1.00	0.9991	0.9999
Carnitine O-acetyltransferase	CACP_HUMAN	P43155	1.22	0.0192	0.2873	1.10	0.2917	0.9982	0.88	0.2049	0.9960	1.03	0.7088	0.9939	1.07	0.4521	0.9951	1.11	0.3443	0.9856	0.96	0.6673	0.8929	1.07	0.4829	0.6860	1.11	0.2359	0.9889	1.08	0.3492	0.9607
Carnitine O-palmitoyltransferase 1, muscle isoform	CPT1B_HUMAN	Q92523	1.13	0.2267	0.5006	1.04	0.7305	0.9982	1.00	0.9744	0.9960	1.03	0.7899	0.9939	1.09	0.4293	0.9951	0.98	0.8701	0.9959	0.88	0.2080	0.7149	1.31	0.0129	0.1544	1.05	0.6062	0.9889	1.10	0.3499	0.9607
Carnitine O-palmitoyltransferase 2, mitochondrial	CPT2_HUMAN	P23786	1.15	0.0738	0.3253	1.11	0.1761	0.9982	0.95	0.5432	0.9960	1.00	0.9944	0.9986	1.04	0.6679	0.9951	1.01	0.9341	0.9959	0.99	0.9104	0.9848	1.15	0.1158	0.3460	1.09	0.3107	0.9889	1.07	0.4085	0.9607
Cartilage oligomeric matrix protein	COMP_HUMAN	P49747	0.75	0.2496	0.5210	0.89	0.6448	0.9982	0.51	0.0213	0.9960	0.96	0.8568	0.9939	0.82	0.4523	0.9951	1.95	0.0304	0.9411	1.20	0.5015	0.8476	0.66	0.1392	0.3817	1.69	0.0423	0.9889	1.00	0.9995	0.9999
Casein kinase II subunit alpha	CSK21_HUMAN	P68400	1.00	0.9540	0.9721	0.98	0.7040	0.9982	1.02	0.7947	0.9960	1.06	0.2692	0.9939	0.97	0.5661	0.9951	0.92	0.2033	0.9856	0.99	0.8907	0.9753	1.07	0.2738	0.5288	0.93	0.2000	0.9889	0.92	0.1251	0.9607
Catalase	CATA_HUMAN	P04040	1.04	0.5615	0.7643	1.01	0.9349	0.9982	0.88	0.0930	0.9960	1.02	0.7784	0.9939	1.12	0.1001	0.9951	1.10	0.2354	0.9856	1.21	0.0058	0.6779	0.92	0.2979	0.5473	0.98	0.7978	0.9889	1.03	0.6461	0.9881
Catechol O-methyltransferase	COMT_HUMAN	P21964	0.97	0.7107	0.8664	1.03	0.7327	0.9982	1.16	0.1050	0.9960	1.04	0.6370	0.9939	0.96	0.6660	0.9951	0.81	0.0299	0.9411	1.00	0.9656	0.9931	0.89	0.1931	0.4535	0.96	0.5934	0.9889	0.93	0.3412	0.9607
Catenin alpha-1	CTNA1_HUMAN	P35221	1.03	0.6446	0.8286	0.86	0.0168	0.9982	1.00	0.9600	0.9960	1.01	0.8809	0.9939	1.11	0.1248	0.9951	0.92	0.3180	0.9856	0.94	0.3677	0.7673	1.06	0.4035	0.6299	0.97	0.6488	0.9889	1.09	0.2057	0.9607
Catenin alpha-3	CTNA3_HUMAN	Q9UI47	1.04	0.5420	0.7503	1.02	0.7517	0.9982	1.05	0.5333	0.9960	1.05	0.4688	0.9939	1.02	0.7336	0.9951	0.98	0.7716	0.9857	0.90	0.1181	0.6868	1.07	0.3955	0.6232	1.00	0.9456	0.9922	1.05	0.4839	0.9652
Catenin beta-1	CTNB1_HUMAN	P35222	1.00	0.9584	0.9734	0.94	0.3721	0.9982	1.10	0.2366	0.9960	0.94	0.3871	0.9939	1.01	0.9197	0.9951	0.86	0.0786	0.9856	0.88	0.1040	0.6824	1.08	0.3561	0.5923	1.05	0.5189	0.9889	1.06	0.4360	0.9607
Catenin delta-1	CTND1_HUMAN	O60716	1.08	0.1805	0.4557	1.03	0.6507	0.9982	0.88	0.0698	0.9960	1.10	0.1068	0.9874	1.12	0.0760	0.9951	1.04	0.6371	0.9856	1.09	0.1563	0.6871	0.95	0.4708	0.6780	1.06	0.3491	0.9889	1.17	0.0068	0.9304
Cathepsin B	CATB_HUMAN	P07858	1.13	0.2739	0.5446	1.08	0.4875	0.9982	1.12	0.3815	0.9960	0.84	0.1137	0.9874	1.03	0.7831	0.9951	0.92	0.5320	0.9856	0.97	0.8271	0.9492	0.71	0.0043	0.1244	0.85	0.1701	0.9889	0.97	0.7530	0.9881
Cathepsin D	CATD_HUMAN	P07339	1.24	0.0023	0.2005	1.14	0.0686	0.9982	0.96	0.6180	0.9960	0.97	0.6479	0.9939	1.07	0.3801	0.9951	1.02	0.8385	0.9959	0.90	0.1557	0.6871	0.98	0.8035	0.8927	0.95	0.4747	0.9889	1.03	0.7084	0.9881
Cathepsin L1	CATL1_HUMAN	P07711	1.09	0.2117	0.4843	1.04	0.5754	0.9982	1.03	0.7473	0.9960	1.25	0.0011	0.8235	1.15	0.0529	0.9951	0.97	0.7125	0.9857	1.07	0.3701	0.7673	0.91	0.2327	0.4959	1.00	0.9952	0.9990	1.02	0.7396	0.9881
Cathepsin Z	CATZ_HUMAN	Q9UBR2	0.99	0.8779	0.9384	1.01	0.8811	0.9982	1.14	0.2333	0.9960	0.87	0.1367	0.9874	0.87	0.1561	0.9951	0.91	0.4148	0.9856	0.87	0.1585	0.6871	1.07	0.5524	0.7377	0.86	0.1329	0.9889	0.82	0.0372	0.9607
Cation-dependent mannose-6-phosphate receptor	MPRD_HUMAN	P20645	0.94	0.4924	0.7220	1.12	0.1833	0.9982	1.10	0.3337	0.9960	0.94	0.4646	0.9939	0.96	0.6125	0.9951	0.89	0.2631	0.9856	1.01	0.8760	0.9720	0.80	0.0132	0.1561	1.03	0.6949	0.9889	0.98	0.8401	0.9881
Cation-independent mannose-6-phosphate receptor	MPRI_HUMAN	P11717	0.96	0.5997	0.7947	1.13	0.1122	0.9982	1.04	0.6693	0.9960	1.00	0.9964	0.9990	1.09	0.2934	0.9951	0.94	0.4913	0.9856	1.06	0.4722	0.8308	0.83	0.0262	0.1832	0.93	0.3305	0.9889	1.04	0.6472	0.9881
Caveolae-associated protein 1	CAVN1_HUMAN	Q6NZI2	0.87	0.0966	0.3563	1.00	0.9906	0.9985	1.06	0.5535	0.9960	1.10	0.2379	0.9939	0.99	0.8868	0.9951	0.92	0.4172	0.9856	1.22	0.0175	0.6779	0.79	0.0092	0.1378	0.98	0.8350	0.9889	0.98	0.7583	0.9881
Caveolae-associated protein 2	CAVN2_HUMAN	O95810	0.89	0.0301	0.2873	1.04	0.5120	0.9982	1.03	0.6562	0.9960	1.07	0.2647	0.9939	0.98	0.7303	0.9951	0.94	0.3423	0.9856	1.10	0.1269	0.6868	0.83	0.0022	0.0852	1.06	0.3364	0.9889	0.98	0.6590	0.9881
Caveolae-associated protein 3	CAVN3_HUMAN	Q969G5	0.97	0.7604	0.8882	0.97	0.7103	0.9982	0.86	0.1272	0.9960	1.03	0.7155	0.9939	1.05	0.5701	0.9951	1.07	0.5356	0.9856	1.16	0.1008	0.6824	0.83	0.0440	0.2224	0.96	0.6289	0.9889	1.07	0.4434	0.9607
Caveolae-associated protein 4	CAVN4_HUMAN	Q5BKX8	0.93	0.4507	0.6954	0.95	0.5622	0.9982	1.21	0.0773	0.9960	1.10	0.3291	0.9939	1.03	0.7977	0.9951	0.75	0.0150	0.9411	0.91	0.3733	0.7708	0.93	0.4992	0.6976	0.82	0.0372	0.9889	0.93	0.4645	0.9607
Caveolin-1	CAV1_HUMAN	Q03135	0.85	0.0335	0.2873	0.98	0.7477	0.9982	1.15	0.1248	0.9960	0.94	0.4121	0.9939	0.87	0.0780	0.9951	0.81	0.0237	0.9411	0.91	0.2587	0.7187	0.93	0.4291	0.6500	0.86	0.0514	0.9889	0.87	0.0626	0.9607
CD151 antigen	CD151_HUMAN	P48509	0.87	0.2530	0.5225	1.06	0.6167	0.9982	1.33	0.0398	0.9960	0.88	0.2926	0.9939	0.92	0.4980	0.9951	0.76	0.0590	0.9641	1.02	0.8636	0.9639	0.71	0.0085	0.1361	0.89	0.3704	0.9889	0.90	0.3626	0.9607
CD44 antigen	CD44_HUMAN	P16070	0.82	0.1139	0.3748	1.11	0.4181	0.9982	0.98	0.8952	0.9960	1.08	0.5374	0.9939	0.94	0.6436	0.9951	1.00	0.9865	0.9992	1.26	0.0789	0.6824	0.65	0.0012	0.0763	0.89	0.3432	0.9889	0.95	0.6922	0.9881
CD59 glycoprotein	CD59_HUMAN	P13987	0.98	0.7326	0.8760	0.99	0.8993	0.9982	1.21	0.0023	0.9960	1.02	0.7381	0.9939	0.99	0.9177	0.9951	0.79	0.0004	0.3206	0.99	0.8208	0.9481	0.91	0.1447	0.3898	0.94	0.2798	0.9889	0.96	0.4915	0.9712
CD81 antigen	CD81_HUMAN	P60033	0.77	0.0130	0.2710	1.00	0.9978	0.9985	1.05	0.7160	0.9960	1.00	0.9749	0.9961	0.87	0.1959	0.9951	0.94	0.6276	0.9856	1.24	0.0583	0.6824	0.84	0.1378	0.3791	0.94	0.5465	0.9889	0.79	0.0226	0.9607
CD99 antigen	CD99_HUMAN	P14209	0.94	0.6521	0.8297	0.89	0.3615	0.9982	0.77	0.0783	0.9960	1.15	0.2526	0.9939	1.07	0.6326	0.9951	1.19	0.2743	0.9856	1.04	0.7754	0.9323	0.99	0.9612	0.9782	1.10	0.4621	0.9889	1.08	0.5589	0.9881
CDGSH iron-sulfur domain-containing protein 1	CISD1_HUMAN	Q9NZ45	1.02	0.8039	0.9060	1.04	0.5433	0.9982	1.03	0.7647	0.9960	0.99	0.9411	0.9939	1.02	0.8377	0.9951	0.95	0.5244	0.9856	0.89	0.1193	0.6868	1.01	0.9490	0.9708	1.01	0.8849	0.9889	1.04	0.5381	0.9881
CDGSH iron-sulfur domain-containing protein 3, mitochondrial	CISD3_HUMAN	P0C7P0	1.01	0.8922	0.9437	1.07	0.2646	0.9982	1.10	0.1908	0.9960	1.02	0.7136	0.9939	1.01	0.8969	0.9951	0.95	0.5211	0.9856	0.96	0.5586	0.8601	1.00	0.9585	0.9773	1.03	0.6557	0.9889	1.02	0.7145	0.9881
CDK5 regulatory subunit-associated protein 3	CK5P3_HUMAN	Q96JB5	1.00	0.9499	0.9699	1.01	0.8899	0.9982	1.04	0.3934	0.9960	1.02	0.6759	0.9939	0.98	0.6662	0.9951	0.94	0.2204	0.9856	0.97	0.5559	0.8601	1.05	0.3274	0.5681	0.98	0.6974	0.9889	0.99	0.7928	0.9881
Cell division control protein 42 homolog	CDC42_HUMAN	P60953	0.82	0.0032	0.2005	0.98	0.7350	0.9982	1.06	0.4826	0.9960	0.97	0.6765	0.9939	0.91	0.1697	0.9951	0.93	0.3780	0.9856	1.11	0.1683	0.6934	0.89	0.1166	0.3471	0.90	0.1521	0.9889	0.91	0.1869	0.9607
Cell surface glycoprotein MUC18	MUC18_HUMAN	P43121	0.87	0.0977	0.3563	0.99	0.8584	0.9982	1.01	0.8820	0.9960	0.93	0.4157	0.9939	0.89	0.2010	0.9951	0.95	0.6451	0.9856	1.14	0.1361	0.6871	0.88	0.1592	0.4083	0.85	0.0597	0.9889	0.87	0.0807	0.9607
Cellular nucleic acid-binding protein	CNBP_HUMAN	P62633	0.88	0.1224	0.3866	0.93	0.4171	0.9982	0.95	0.6266	0.9960	0.82	0.0114	0.9874	0.87	0.1102	0.9951	1.14	0.1943	0.9856	0.84	0.0377	0.6824	1.19	0.0624	0.2584	0.98	0.7710	0.9889	0.98	0.7790	0.9881
Ceruloplasmin	CERU_HUMAN	P00450	0.89	0.3465	0.6090	1.19	0.1586	0.9982	1.06	0.6621	0.9960	1.08	0.5084	0.9939	1.01	0.9570	0.9951	0.92	0.5593	0.9856	1.14	0.3108	0.7378	0.70	0.0068	0.1326	0.97	0.7947	0.9889	0.90	0.3753	0.9607
cGMP-inhibited 3',5'-cyclic phosphodiesterase A	PDE3A_HUMAN	Q14432	1.12	0.2018	0.4813	1.04	0.7065	0.9982	1.04	0.6874	0.9960	0.85	0.0771	0.9874	0.96	0.6864	0.9951	1.00	0.9710	0.9967	0.86	0.1082	0.6824	1.00	0.9811	0.9891	1.04	0.7124	0.9889	1.11	0.2659	0.9607
Chloride intracellular channel protein 1	CLIC1_HUMAN	O00299	0.98	0.8205	0.9184	1.01	0.8434	0.9982	0.92	0.3012	0.9960	0.95	0.4939	0.9939	0.97	0.6505	0.9951	1.10	0.2693	0.9856	1.00	0.9909	0.9992	0.93	0.3848	0.6145	1.06	0.4631	0.9889	0.97	0.6532	0.9881
Chloride intracellular channel protein 4	CLIC4_HUMAN	Q9Y696	1.07	0.2295	0.5032	0.99	0.8266	0.9982	0.95	0.4750	0.9960	0.96	0.4822	0.9939	0.97	0.5780	0.9951	1.07	0.3559	0.9856	0.98	0.7569	0.9282	0.96	0.5556	0.7394	1.02	0.7189	0.9889	0.99	0.8924	0.9938
Chromobox protein homolog 1	CBX1_HUMAN	P83916	1.07	0.3827	0.6407	0.94	0.3904	0.9982	0.99	0.8962	0.9960	0.98	0.8217	0.9939	1.05	0.5317	0.9951	1.08	0.4074	0.9856	0.93	0.3778	0.7741	1.10	0.2271	0.4943	0.92	0.2676	0.9889	1.02	0.8002	0.9881
Chromobox protein homolog 3	CBX3_HUMAN	Q13185	0.97	0.6766	0.8429	0.96	0.5199	0.9982	0.99	0.8935	0.9960	0.90	0.1174	0.9874	0.93	0.2908	0.9951	1.10	0.2525	0.9856	0.90	0.1180	0.6868	1.10	0.1836	0.4384	0.90	0.1038	0.9889	0.91	0.1393	0.9607
Citramalyl-CoA lyase, mitochondrial	CLYBL_HUMAN	Q8N0X4	0.98	0.8617	0.9348	1.02	0.8425	0.9982	0.89	0.3104	0.9960	0.89	0.2244	0.9939	0.90	0.3118	0.9951	1.11	0.4117	0.9856	0.95	0.6330	0.8882	1.11	0.3572	0.5925	1.08	0.4827	0.9889	1.18	0.0888	0.9607
Citrate synthase, mitochondrial	CISY_HUMAN	O75390	1.19	0.0598	0.3008	1.05	0.6184	0.9982	0.89	0.2721	0.9960	0.93	0.4097	0.9939	0.94	0.5206	0.9951	1.15	0.2148	0.9856	0.86	0.1301	0.6868	1.22	0.0538	0.2446	1.15	0.1412	0.9889	1.03	0.7132	0.9881
Clathrin heavy chain 1	CLH1_HUMAN	Q00610	1.06	0.4627	0.7039	1.01	0.9135	0.9982	1.17	0.1026	0.9960	0.97	0.6987	0.9939	1.01	0.9081	0.9951	0.89	0.2859	0.9856	0.91	0.2876	0.7212	1.24	0.0242	0.1763	1.06	0.5274	0.9889	1.05	0.5739	0.9881
Clathrin light chain A	CLCA_HUMAN	P09496	0.83	0.0927	0.3485	1.04	0.7409	0.9982	0.99	0.9462	0.9960	1.06	0.5920	0.9939	0.98	0.8318	0.9951	1.02	0.8842	0.9959	1.22	0.1039	0.6824	0.73	0.0135	0.1571	1.05	0.6884	0.9889	0.95	0.6679	0.9881
Clathrin light chain B	CLCB_HUMAN	P09497	0.94	0.1947	0.4726	0.96	0.4422	0.9982	1.00	0.9971	0.9983	1.06	0.2838	0.9939	0.96	0.4052	0.9951	0.95	0.4394	0.9856	1.00	0.9480	0.9913	0.97	0.6444	0.7988	1.06	0.2435	0.9889	1.01	0.8995	0.9938
Clustered mitochondria protein homolog	CLU_HUMAN	O75153	1.12	0.0827	0.3309	0.96	0.4817	0.9982	0.96	0.6084	0.9960	1.07	0.2705	0.9939	1.07	0.2861	0.9951	1.01	0.8671	0.9959	0.95	0.4797	0.8352	1.21	0.0060	0.1317	1.06	0.3962	0.9889	1.07	0.2622	0.9607
Clusterin	CLUS_HUMAN	P10909	0.80	0.1947	0.4726	1.26	0.1688	0.9982	0.96	0.8333	0.9960	0.85	0.3349	0.9939	0.93	0.6597	0.9951	0.94	0.7640	0.9857	1.20	0.3125	0.7400	0.56	0.0018	0.0815	1.04	0.8103	0.9889	0.90	0.5492	0.9881
Coagulation factor IX	FA9_HUMAN	P00740	0.90	0.3096	0.5800	1.04	0.6917	0.9982	1.17	0.2033	0.9960	0.91	0.3623	0.9939	0.86	0.1535	0.9951	0.86	0.2564	0.9856	0.93	0.5505	0.8601	0.85	0.1779	0.4315	1.06	0.6042	0.9889	0.94	0.5446	0.9881
Coagulation factor XIII A chain	F13A_HUMAN	P00488	0.99	0.9138	0.9536	1.29	0.0259	0.9982	0.98	0.8592	0.9960	1.09	0.4420	0.9939	0.92	0.4748	0.9951	1.00	0.9752	0.9967	1.26	0.0585	0.6824	0.73	0.0113	0.1499	1.03	0.7868	0.9889	0.84	0.1164	0.9607
Coatomer subunit alpha	COPA_HUMAN	P53621	1.11	0.1028	0.3639	1.05	0.4277	0.9982	0.99	0.9067	0.9960	1.06	0.3529	0.9939	1.03	0.6238	0.9951	1.03	0.6751	0.9856	1.00	0.9881	0.9992	1.03	0.6829	0.8225	1.03	0.6347	0.9889	1.03	0.6699	0.9881
Coatomer subunit beta'	COPB2_HUMAN	P35606	1.01	0.8371	0.9202	1.07	0.2163	0.9982	1.06	0.3585	0.9960	1.08	0.1376	0.9874	1.05	0.4143	0.9951	0.95	0.4476	0.9856	1.03	0.6572	0.8915	0.96	0.4649	0.6760	0.96	0.4499	0.9889	0.98	0.6947	0.9881
Coatomer subunit delta	COPD_HUMAN	P48444	1.05	0.4403	0.6862	1.10	0.1072	0.9982	1.06	0.3941	0.9960	0.91	0.1221	0.9874	0.92	0.2205	0.9951	0.95	0.5031	0.9856	0.91	0.1487	0.6871	1.12	0.0861	0.2982	1.01	0.8466	0.9889	0.97	0.6215	0.9881
Coatomer subunit epsilon	COPE_HUMAN	O14579	1.01	0.7966	0.9014	0.99	0.8739	0.9982	1.07	0.2585	0.9960	1.07	0.1827	0.9939	1.04	0.4714	0.9951	0.93	0.2974	0.9856	1.00	0.9464	0.9908	0.95	0.3665	0.5972	0.95	0.3697	0.9889	1.03	0.5811	0.9881
Coatomer subunit zeta-1	COPZ1_HUMAN	P61923	1.09	0.0586	0.2994	1.00	0.9893	0.9985	0.96	0.4250	0.9960	0.99	0.7593	0.9939	1.04	0.3989	0.9951	1.04	0.4360	0.9856	0.94	0.1800	0.7130	0.99	0.8406	0.9140	0.96	0.3905	0.9889	1.01	0.7991	0.9881
Cocaine esterase	EST2_HUMAN	O00748	1.08	0.3942	0.6515	1.12	0.2215	0.9982	1.02	0.8539	0.9960	1.08	0.4264	0.9939	0.99	0.8810	0.9951	0.96	0.7161	0.9857	0.89	0.2221	0.7187	1.15	0.1675	0.4206	0.87	0.1335	0.9889	0.92	0.3757	0.9607
Cofilin-1	COF1_HUMAN	P23528	0.88	0.0531	0.2939	0.97	0.6260	0.9982	1.03	0.6583	0.9960	0.95	0.4636	0.9939	0.87	0.0346	0.9951	1.04	0.6599	0.9856	1.00	0.9992	0.9992	1.07	0.3594	0.5943	0.92	0.2019	0.9889	0.83	0.0021	0.9304
Cofilin-2	COF2_HUMAN	Q9Y281	1.02	0.7907	0.8986	0.93	0.3439	0.9982	1.04	0.6329	0.9960	0.95	0.5208	0.9939	0.89	0.1060	0.9951	1.01	0.9136	0.9959	0.89	0.1158	0.6824	1.31	0.0005	0.0621	0.93	0.3042	0.9889	0.90	0.1643	0.9607
Coiled-coil domain-containing protein 141	CC141_HUMAN	Q6ZP82	1.26	0.0023	0.2005	1.01	0.8700	0.9982	0.89	0.1893	0.9960	1.10	0.2480	0.9939	1.15	0.0791	0.9951	1.04	0.6975	0.9856	0.95	0.5522	0.8601	1.13	0.1737	0.4257	1.11	0.1843	0.9889	1.16	0.0547	0.9607
Coiled-coil domain-containing protein 47	CCD47_HUMAN	Q96A33	1.00	0.9407	0.9668	0.99	0.9282	0.9982	0.93	0.2707	0.9960	1.05	0.3824	0.9939	1.10	0.1265	0.9951	1.02	0.8353	0.9959	1.08	0.2477	0.7187	0.86	0.0197	0.1655	0.97	0.5860	0.9889	1.06	0.3353	0.9607
Coiled-coil domain-containing protein 58	CCD58_HUMAN	Q4VC31	1.17	0.0365	0.2873	1.11	0.1782	0.9982	1.06	0.4726	0.9960	1.02	0.7868	0.9939	1.03	0.7505	0.9951	0.99	0.9354	0.9959	0.96	0.6372	0.8888	0.95	0.5702	0.7499	0.98	0.7667	0.9889	0.94	0.4016	0.9607
Coiled-coil domain-containing protein 90B, mitochondrial	CC90B_HUMAN	Q9GZT6	1.02	0.6456	0.8286	0.98	0.7409	0.9982	0.92	0.1676	0.9960	1.02	0.7731	0.9939	1.00	0.9562	0.9951	1.02	0.7114	0.9857	1.05	0.3373	0.7460	1.06	0.3386	0.5744	1.02	0.6800	0.9889	1.09	0.0831	0.9607
Coiled-coil-helix-coiled-coil-helix domain-containing protein 2	CHCH2_HUMAN	Q9Y6H1	0.98	0.7749	0.8924	1.04	0.6583	0.9982	1.05	0.5928	0.9960	0.95	0.4962	0.9939	0.94	0.4240	0.9951	1.04	0.7120	0.9857	0.92	0.3473	0.7593	1.23	0.0187	0.1650	1.02	0.8504	0.9889	0.96	0.6071	0.9881
Collagen alpha-1(VI) chain	CO6A1_HUMAN	P12109	0.88	0.2464	0.5201	1.11	0.3482	0.9982	1.07	0.5878	0.9960	0.89	0.3166	0.9939	0.90	0.3824	0.9951	0.86	0.2738	0.9856	1.12	0.3640	0.7671	0.72	0.0082	0.1361	1.02	0.8715	0.9889	0.89	0.3192	0.9607
Collagen alpha-1(XIV) chain	COEA1_HUMAN	Q05707	0.74	0.0873	0.3401	0.83	0.2896	0.9982	0.92	0.6667	0.9960	0.92	0.6583	0.9939	0.79	0.1938	0.9951	1.08	0.7239	0.9857	1.05	0.7928	0.9431	0.69	0.0577	0.2494	0.94	0.7358	0.9889	0.80	0.2069	0.9607
Collagen alpha-1(XV) chain	COFA1_HUMAN	P39059	0.94	0.4906	0.7219	0.94	0.4841	0.9982	1.05	0.5979	0.9960	1.05	0.5301	0.9939	0.89	0.1860	0.9951	1.03	0.7703	0.9857	1.07	0.4488	0.8181	1.14	0.1545	0.4044	1.04	0.6571	0.9889	0.90	0.1904	0.9607
Collagen alpha-1(XVIII) chain	COIA1_HUMAN	P39060	0.80	0.0312	0.2873	1.04	0.7334	0.9982	1.16	0.2168	0.9960	1.08	0.4525	0.9939	0.85	0.1253	0.9951	0.82	0.1285	0.9856	1.16	0.1645	0.6896	0.78	0.0333	0.2017	0.97	0.7965	0.9889	0.88	0.2125	0.9607
Collagen alpha-2(VI) chain	CO6A2_HUMAN	P12110	0.87	0.2967	0.5682	0.93	0.5887	0.9982	1.13	0.4347	0.9960	0.96	0.7832	0.9939	0.98	0.8719	0.9951	0.83	0.2524	0.9856	1.18	0.2250	0.7187	0.66	0.0031	0.1090	1.09	0.5368	0.9889	1.02	0.8574	0.9881
Collagen alpha-3(VI) chain	CO6A3_HUMAN	P12111	0.78	0.0326	0.2873	0.97	0.7976	0.9982	1.04	0.8004	0.9960	0.92	0.5072	0.9939	0.85	0.2019	0.9951	0.89	0.4415	0.9856	0.93	0.5591	0.8601	0.75	0.0295	0.1909	1.04	0.7540	0.9889	0.92	0.4919	0.9712
Complement C3	CO3_HUMAN	P01024	0.81	0.0900	0.3428	1.14	0.3062	0.9982	1.12	0.4219	0.9960	0.99	0.9312	0.9939	0.88	0.3415	0.9951	0.90	0.4835	0.9856	1.14	0.3256	0.7400	0.72	0.0170	0.1636	1.02	0.9011	0.9889	0.86	0.2135	0.9607

Complement C4-B	CO4B_HUMAN	P0C0L5	0.80	0.0877	0.3401	1.12	0.3802	0.9982	1.09	0.5504	0.9960	0.99	0.9405	0.9939	0.89	0.4058	0.9951	0.91	0.5368	0.9856	1.17	0.2446	0.7187	0.65	0.0021	0.0830	1.06	0.6724	0.9889	0.91	0.4717	0.9607
Complement component 1 Q subcomponent-binding protein, mitochond	C1QBP_HUMAN	Q07021	1.03	0.7076	0.8642	1.07	0.3159	0.9982	0.94	0.4800	0.9960	0.95	0.4655	0.9939	0.97	0.6426	0.9951	1.06	0.5267	0.9856	0.88	0.0719	0.6824	1.08	0.3189	0.5613	0.99	0.9279	0.9889	0.95	0.4701	0.9607
Complement component C1q receptor	C1QR1_HUMAN	Q9NPY3	1.00	0.9663	0.9808	1.00	0.9701	0.9982	0.92	0.2675	0.9960	0.91	0.1804	0.9939	0.96	0.5760	0.9951	1.18	0.0424	0.9411	1.11	0.1480	0.6871	1.02	0.7774	0.8813	1.02	0.8152	0.9889	0.98	0.7252	0.9881
Complement component C9	CO9_HUMAN	P02748	0.79	0.0645	0.3091	1.22	0.1299	0.9982	1.04	0.7753	0.9960	1.04	0.7586	0.9939	0.89	0.3906	0.9951	0.90	0.5083	0.9856	1.01	0.9376	0.9903	0.59	0.0002	0.0621	1.04	0.7976	0.9889	0.82	0.1304	0.9607
Complement factor B	CFAB_HUMAN	P00751	0.81	0.1056	0.3654	1.10	0.4570	0.9982	1.18	0.2594	0.9960	1.05	0.6831	0.9939	0.90	0.4430	0.9951	0.87	0.3615	0.9856	1.11	0.4353	0.8109	0.71	0.0144	0.1589	1.01	0.9204	0.9889	0.86	0.2418	0.9607
Complement factor H	CFAH_HUMAN	P08603	0.94	0.7632	0.8882	1.07	0.7397	0.9982	0.90	0.6443	0.9960	1.14	0.5212	0.9939	1.15	0.5173	0.9951	1.18	0.5140	0.9856	1.40	0.1154	0.6824	0.63	0.0382	0.2132	1.23	0.3147	0.9889	1.06	0.7591	0.9881
Complex I assembly factor ACAD9, mitochondrial	ACAD9_HUMAN	Q9H845	1.09	0.3235	0.5917	1.07	0.4367	0.9982	1.01	0.9020	0.9960	0.94	0.4186	0.9939	0.98	0.8009	0.9951	0.92	0.4120	0.9856	0.90	0.2571	0.7187	1.11	0.2616	0.5247	1.08	0.3652	0.9889	1.02	0.7872	0.9881
Complex I intermediate-associated protein 30, mitochondrial	CIA30_HUMAN	Q9Y375	1.18	0.1105	0.3718	1.15	0.1724	0.9982	0.96	0.7315	0.9960	0.88	0.2214	0.9939	1.06	0.5763	0.9951	0.99	0.9578	0.9967	0.90	0.3508	0.7593	1.00	0.9920	0.9952	1.01	0.9391	0.9922	1.15	0.1669	0.9607
Complex III assembly factor LYRM7	LYRM7_HUMAN	Q5U5X0	1.17	0.0416	0.2873	1.06	0.4146	0.9982	1.10	0.2877	0.9960	0.94	0.4258	0.9939	1.02	0.7917	0.9951	0.90	0.2590	0.9856	0.94	0.4673	0.8278	1.03	0.6993	0.8299	0.95	0.4831	0.9889	0.96	0.5670	0.9881
COP9 signalosome complex subunit 1	CSN1_HUMAN	Q13098	1.10	0.1246	0.3905	1.09	0.1973	0.9982	1.00	0.9826	0.9960	0.94	0.3631	0.9939	0.95	0.4021	0.9951	0.98	0.7752	0.9875	0.91	0.1734	0.7012	1.10	0.2000	0.4593	0.99	0.8984	0.9889	0.98	0.7764	0.9881
COP9 signalosome complex subunit 2	CSN2_HUMAN	P61201	1.09	0.1894	0.4651	1.09	0.1840	0.9982	1.08	0.3060	0.9960	1.04	0.5431	0.9939	0.98	0.7199	0.9951	0.94	0.4638	0.9856	0.93	0.2897	0.7212	1.07	0.3414	0.5766	0.95	0.4903	0.9889	0.96	0.4843	0.9652
COP9 signalosome complex subunit 3	CSN3_HUMAN	Q9UNS2	0.97	0.6719	0.8410	0.93	0.3332	0.9982	1.01	0.9110	0.9960	1.00	0.9917	0.9981	0.99	0.8627	0.9951	1.00	0.9857	0.9992	1.09	0.2807	0.7212	1.07	0.3963	0.6232	0.91	0.2150	0.9889	0.92	0.2778	0.9607
COP9 signalosome complex subunit 4	CSN4_HUMAN	Q9BT78	1.04	0.4951	0.7237	1.01	0.7940	0.9982	1.13	0.0525	0.9960	0.97	0.6320	0.9939	0.95	0.3687	0.9951	0.94	0.3546	0.9856	0.99	0.9094	0.9848	1.07	0.2714	0.5288	0.97	0.5810	0.9889	0.98	0.7033	0.9881
COP9 signalosome complex subunit 5	CSN5_HUMAN	Q92905	0.97	0.6915	0.8511	1.00	0.9539	0.9982	0.92	0.4224	0.9960	1.07	0.4487	0.9939	0.99	0.8804	0.9951	1.09	0.3888	0.9856	0.85	0.0626	0.6824	1.08	0.4430	0.6607	1.06	0.5170	0.9889	1.05	0.5623	0.9881
COP9 signalosome complex subunit 6	CSN6_HUMAN	Q7L5N1	1.14	0.0559	0.2942	1.13	0.0853	0.9982	1.16	0.0637	0.9960	1.03	0.6574	0.9939	1.02	0.8037	0.9951	0.89	0.1569	0.9856	0.93	0.3187	0.7400	1.05	0.5292	0.7227	0.95	0.4601	0.9889	1.02	0.7391	0.9881
COP9 signalosome complex subunit 7a	CSN7A_HUMAN	Q9UBW8	1.10	0.1469	0.4117	1.13	0.0707	0.9982	0.98	0.7890	0.9960	1.00	0.9521	0.9939	1.01	0.9044	0.9951	1.03	0.7225	0.9857	0.92	0.2105	0.7157	1.02	0.7972	0.8904	1.07	0.3104	0.9889	1.03	0.6076	0.9881
COP9 signalosome complex subunit 8	CSN8_HUMAN	Q99627	1.00	0.9508	0.9699	0.98	0.7018	0.9982	1.00	0.9581	0.9960	1.03	0.5641	0.9939	0.99	0.8352	0.9951	1.00	0.9605	0.9967	0.97	0.6419	0.8892	1.12	0.0595	0.2519	0.98	0.7617	0.9889	1.03	0.5748	0.9881
Copine-3	CPNE3_HUMAN	O75131	1.14	0.0135	0.2710	1.02	0.7049	0.9982	1.02	0.7044	0.9960	0.97	0.5720	0.9939	1.04	0.4963	0.9951	0.99	0.8787	0.9959	0.90	0.0704	0.6824	1.11	0.0863	0.2982	0.95	0.3408	0.9889	1.02	0.7303	0.9881
Core histone macro-H2A.1	H2AY_HUMAN	O75367	0.86	0.0786	0.3281	1.04	0.6210	0.9982	0.96	0.6684	0.9960	0.98	0.7698	0.9939	0.94	0.4529	0.9951	1.02	0.8628	0.9959	1.11	0.2699	0.7187	0.78	0.0093	0.1378	1.00	0.9603	0.9961	0.98	0.8317	0.9881
Core histone macro-H2A.2	H2AW_HUMAN	Q9P0M6	0.82	0.0431	0.2873	1.04	0.6872	0.9982	0.81	0.0645	0.9960	1.07	0.4989	0.9939	0.93	0.4714	0.9951	1.19	0.1662	0.9856	1.06	0.6113	0.8732	0.79	0.0287	0.1909	0.97	0.7368	0.9889	0.97	0.7473	0.9881
Coronin-1C	COR1C_HUMAN	Q9ULV4	0.88	0.1431	0.4056	1.01	0.9351	0.9982	1.05	0.6288	0.9960	1.07	0.4591	0.9939	0.98	0.8383	0.9951	1.00	0.9651	0.9967	1.08	0.4085	0.7891	0.84	0.0790	0.2866	0.96	0.6389	0.9889	1.00	0.9888	0.9992
Corrinoid adenosyltransferase	MMAB_HUMAN	Q96EY8	1.08	0.4518	0.6961	1.02	0.8767	0.9982	0.84	0.1129	0.9960	1.10	0.3424	0.9939	1.07	0.5260	0.9951	1.16	0.2037	0.9856	0.94	0.5125	0.8518	0.92	0.4684	0.6776	1.17	0.1073	0.9889	1.04	0.6874	0.9881
Creatine kinase B-type	KCRB_HUMAN	P12277	1.31	0.0054	0.2406	1.06	0.5381	0.9982	0.81	0.0554	0.9960	0.93	0.4813	0.9939	1.13	0.2184	0.9951	1.34	0.0150	0.9411	0.96	0.7241	0.9205	1.09	0.4170	0.6425	1.02	0.8476	0.9889	1.18	0.0843	0.9607
Creatine kinase M-type	KCRM_HUMAN	P06732	1.18	0.0471	0.2898	1.00	0.9789	0.9985	0.85	0.0929	0.9960	0.99	0.8605	0.9939	1.04	0.6982	0.9951	1.25	0.0362	0.9411	0.97	0.7381	0.9218	1.30	0.0056	0.1317	1.02	0.8138	0.9889	1.07	0.4117	0.9607
Creatine kinase S-type, mitochondrial	KCRS_HUMAN	P17540	1.22	0.0044	0.2034	1.01	0.8696	0.9982	0.94	0.4622	0.9960	1.06	0.4000	0.9939	1.08	0.2768	0.9951	1.11	0.2356	0.9856	1.00	0.9938	0.9992	1.16	0.0563	0.2474	1.04	0.6341	0.9889	1.07	0.3616	0.9607
Cx9C motif-containing protein 4	CMC4_HUMAN	P56277	1.12	0.1662	0.4371	1.02	0.8114	0.9982	0.97	0.7507	0.9960	0.98	0.7784	0.9939	1.04	0.6307	0.9951	1.21	0.0549	0.9641	0.91	0.2866	0.7212	1.23	0.0185	0.1650	1.04	0.6541	0.9889	1.04	0.6030	0.9881
Cystatin-B	CYTB_HUMAN	P04080	1.00	0.9513	0.9699	1.01	0.8353	0.9982	1.06	0.4989	0.9960	0.91	0.1885	0.9939	0.98	0.8333	0.9951	0.99	0.9136	0.9959	1.06	0.4359	0.8109	1.03	0.6666	0.8098	0.92	0.2672	0.9889	0.90	0.1367	0.9607
Cystatin-C	CYTC_HUMAN	P01034	1.16	0.0199	0.2873	1.01	0.8385	0.9982	0.94	0.3894	0.9960	1.18	0.0083	0.9874	1.19	0.0083	0.9951	1.04	0.5945	0.9856	1.11	0.1200	0.6868	0.88	0.0599	0.2522	1.02	0.7813	0.9889	1.17	0.0139	0.9304
Cysteine and glycine-rich protein 3	CSRP3_HUMAN	P50461	0.89	0.1072	0.3670	0.93	0.3710	0.9982	1.14	0.1219	0.9960	1.02	0.8337	0.9939	0.91	0.2400	0.9951	0.85	0.0833	0.9856	0.87	0.0781	0.6824	1.12	0.1890	0.4471	1.12	0.1562	0.9889	0.97	0.7041	0.9881
Cysteine desulfurase, mitochondrial	NFS1_HUMAN	Q9Y697	1.05	0.3092	0.5800	1.00	0.9260	0.9982	1.02	0.7623	0.9960	0.99	0.8976	0.9939	0.99	0.7973	0.9951	0.98	0.7589	0.9857	0.95	0.3588	0.7644	1.02	0.6485	0.7998	1.05	0.3051	0.9889	1.01	0.8306	0.9881
Cysteine-rich protein 2	CRIP2_HUMAN	P52943	1.06	0.2346	0.5075	0.99	0.9047	0.9982	0.99	0.8762	0.9960	0.97	0.5696	0.9939	0.97	0.6233	0.9951	1.04	0.4898	0.9856	0.89	0.0363	0.6824	1.04	0.4983	0.6970	1.12	0.0284	0.9889	1.08	0.1452	0.9607
Cytochrome b-c1 complex subunit 1, mitochondrial	QCR1_HUMAN	P31930	1.19	0.0245	0.2873	1.02	0.8484	0.9982	0.97	0.7609	0.9960	0.95	0.5259	0.9939	1.02	0.7788	0.9951	1.02	0.8551	0.9959	0.89	0.1490	0.6871	1.08	0.3968	0.6232	1.09	0.2963	0.9889	1.12	0.1531	0.9607
Cytochrome b-c1 complex subunit 2, mitochondrial	QCR2_HUMAN	P22695	1.11	0.0876	0.3401	1.02	0.7467	0.9982	0.99	0.8631	0.9960	0.94	0.3244	0.9939	1.02	0.7242	0.9951	1.00	0.9920	0.9992	1.01	0.9284	0.9880	0.98	0.8263	0.9067	1.01	0.8706	0.9889	1.08	0.1870	0.9607
Cytochrome b-c1 complex subunit 6, mitochondrial	QCR6_HUMAN	P07919	1.04	0.6472	0.8293	1.02	0.8623	0.9982	1.08	0.4709	0.9960	0.94	0.5124	0.9939	0.91	0.3270	0.9951	0.92	0.4433	0.9856	0.87	0.1386	0.6871	1.16	0.1463	0.3921	1.07	0.4734	0.9889	1.03	0.7280	0.9881
Cytochrome b-c1 complex subunit 7	QCR7_HUMAN	P14927	1.10	0.2031	0.4813	1.07	0.3724	0.9982	0.93	0.3721	0.9960	1.00	0.9501	0.9939	0.97	0.6987	0.9951	1.04	0.7024	0.9857	0.94	0.4356	0.8109	1.08	0.3282	0.5681	1.14	0.0702	0.9889	1.07	0.3273	0.9607
Cytochrome b-c1 complex subunit 8	QCR8_HUMAN	O14949	1.10	0.2569	0.5263	1.11	0.2139	0.9982	1.01	0.9400	0.9960	0.96	0.6601	0.9939	1.06	0.5013	0.9951	0.97	0.7611	0.9857	0.95	0.5539	0.8601	0.90	0.2493	0.5144	1.05	0.5766	0.9889	1.08	0.3686	0.9607
Cytochrome b-c1 complex subunit 9	QCR9_HUMAN	Q9UDW1	1.00	0.9745	0.9855	1.01	0.9332	0.9982	1.09	0.3376	0.9960	0.88	0.0865	0.9874	0.94	0.4540	0.9951	0.89	0.1853	0.9856	0.89	0.1337	0.6871	0.95	0.5744	0.7520	1.05	0.5191	0.9889	1.06	0.4496	0.9607
Cytochrome b-c1 complex subunit Rieske, mitochondrial	UCRI_HUMAN	P47985	1.14	0.0376	0.2873	1.07	0.2852	0.9982	0.96	0.5787	0.9960	1.05	0.4841	0.9939	1.06	0.3589	0.9951	1.01	0.9422	0.9959	0.95	0.5020	0.8476	1.05	0.4960	0.6959	1.10	0.1514	0.9889	1.13	0.0634	0.9607
Cytochrome b5	CYB5_HUMAN	P00167	0.94	0.3597	0.6228	0.98	0.7653	0.9982	1.03	0.6480	0.9960	0.97	0.6017	0.9939	0.95	0.4723	0.9951	0.93	0.3357	0.9856	1.13	0.0618	0.6824	1.03	0.7170	0.8381	0.95	0.4374	0.9889	1.01	0.9078	0.9938
Cytochrome c	CYC_HUMAN	P99999	1.12	0.1234	0.3880	1.00	0.9745	0.9982	0.99	0.9144	0.9960	0.95	0.4381	0.9939	0.92	0.2798	0.9951	1.08	0.4157	0.9856	0.92	0.2935	0.7277	1.12	0.1502	0.3999	1.04	0.5801	0.9889	0.98	0.7592	0.9881
Cytochrome c oxidase assembly factor 3 homolog, mitochondrial	COA3_HUMAN	Q9Y2R0	0.96	0.4359	0.6830	1.00	0.9611	0.9982	0.98	0.7073	0.9960	1.03	0.5366	0.9939	1.04	0.4552	0.9951	1.01	0.8717	0.9959	1.05	0.4061	0.7885	0.97	0.6484	0.7998	1.01	0.8268	0.9889	1.05	0.3540	0.9607
Cytochrome c oxidase assembly factor 4 homolog, mitochondrial	COA4_HUMAN	Q9NYJ1	1.06	0.4525	0.6961	1.13	0.1162	0.9982	1.01	0.9545	0.9960	0.94	0.4684	0.9939	1.02	0.8334	0.9951	1.06	0.5570	0.9856	0.93	0.4035	0.7880	1.14	0.1571	0.4056	1.11	0.2023	0.9889	1.00	0.9757	0.9992
Cytochrome c oxidase assembly factor 6 homolog	COA6_HUMAN	Q5JTJ3	1.13	0.0546	0.2939	0.97	0.5919	0.9982	0.95	0.4425	0.9960	1.01	0.8362	0.9939	1.06	0.3740	0.9951	1.07	0.4179	0.9856	0.92	0.2023	0.7149	1.07	0.3405	0.5756	0.99	0.9229	0.9889	0.97	0.6278	0.9881
Cytochrome c oxidase copper chaperone	COX17_HUMAN	Q14061	1.03	0.8304	0.9186	1.02	0.8546	0.9982	1.04	0.7921	0.9960	0.79	0.0570	0.9874	0.80	0.0741	0.9951	1.11	0.4868	0.9856	0.73	0.0152	0.6779	1.27	0.0845	0.2956	0.94	0.6318	0.9889	0.85	0.1844	0.9607
Cytochrome c oxidase subunit 2	COX2_HUMAN	P00403	1.21	0.0719	0.3224	1.12	0.2773	0.9982	0.89	0.3515	0.9960	0.97	0.7547	0.9939	1.16	0.1797	0.9951	1.08	0.5529	0.9856	1.12	0.3239	0.7400	0.90	0.3874	0.6167	1.13	0.2622	0.9889	1.20	0.0860	0.9607
Cytochrome c oxidase subunit 4 isoform 1, mitochondrial	COX41_HUMAN	P13073	1.11	0.1528	0.4219	1.05	0.5181	0.9982	0.87	0.0945	0.9960	0.96	0.5245	0.9939	1.08	0.3097	0.9951	1.10	0.2969	0.9856	1.05	0.5632	0.8601	0.96	0.6184	0.7841	1.13	0.1027	0.9889	1.12	0.1103	0.9607
Cytochrome c oxidase subunit 5A, mitochondrial	COX5A_HUMAN	P20674	1.23	0.0206	0.2873	1.02	0.8363	0.9982	0.90	0.3153	0.9960	0.88	0.1627	0.9874	1.00	0.9612	0.9951	1.07	0.5489	0.9856	0.90	0.2454	0.7187	1.21	0.0587	0.2503	1.12	0.2090	0.9889	1.10	0.2775	0.9607
Cytochrome c oxidase subunit 5B, mitochondrial	COX5B_HUMAN	P10606	1.19	0.0375	0.2873	1.00	0.9610	0.9982	0.89	0.2343	0.9960	0.94	0.4836	0.9939	1.04	0.6349	0.9951	1.09	0.4232	0.9856	0.92	0.3468	0.7593	1.18	0.0773	0.2846	1.13	0.1687	0.9889	1.14	0.1343	0.9607
Cytochrome c oxidase subunit 6B1	CX6B1_HUMAN	P14854	1.25	0.0098	0.2530	1.10	0.2963	0.9982	0.88	0.2226	0.9960	1.00	0.9634	0.9947	1.03	0.7374	0.9951	1.10	0.3710	0.9856	0.89	0.2213	0.7187	1.09	0.3921	0.6210	1.09	0.3582	0.9889	1.13	0.1720	0.9607
Cytochrome c oxidase subunit 6C	COX6C_HUMAN	P09669	1.18	0.1023	0.3628	1.04	0.6820	0.9982	0.92	0.5074	0.9960	0.88	0.1926	0.9939	1.09	0.4258	0.9951	1.06	0.6410	0.9856	0.87	0.1879	0.7149	1.04	0.7500	0.8625	1.08	0.4605	0.9889	1.18	0.1062	0.9607
Cytochrome c oxidase subunit 7A1, mitochondrial	CX7A1_HUMAN	P24310	1.21	0.0770	0.3266	1.09	0.4259	0.9982	0.92	0.5237	0.9960	0.90	0.3079	0.9939	1.00	0.9730	0.9951	1.01	0.9149	0.9959	0.89	0.2890	0.7212	1.20	0.1283	0.3638	1.06	0.5985	0.9889	1.12	0.2814	0.9607
Cytochrome c oxidase subunit 7A2, mitochondrial	CX7A2_HUMAN	P14406	1.18	0.0586	0.2994	1.05	0.5730	0.9982	1.04	0.7327	0.9960	0.96	0.6021	0.9939	1.04	0.6278	0.9951	0.92	0.4349	0.9856	0.92	0.3811	0.7767	1.14	0.1705	0.4217	1.17	0.0709	0.9889	1.13	0.1458	0.9607
Cytochrome c oxidase subunit 7C, mitochondrial	COX7C_HUMAN	P15954	1.11	0.2658	0.5347	1.05	0.6340	0.9982	0.88	0.2279	0.9960	0.88	0.1653	0.9874	0.93	0.4623	0.9951	1.09	0.4602	0.9856	0.82	0.0535	0.6824	1.23	0.0517	0.2413	1.12	0.2526	0.9889	1.04	0.7172	0.9881
Cytochrome c oxidase subunit NDUFA4	NDUA4_HUMAN	O00483	1.13	0.1915	0.4689	1.07	0.4535	0.9982	0.91	0.3888	0.9960	0.96	0.6328	0.9939	1.02	0.8401	0.9951	1.05	0.6804	0.9856	0.86	0.1245	0.6868	1.08	0.4618	0.6745	1.06	0.5303	0.9889	1.09	0.3538	0.9607
Cytochrome c-type heme lyase	CCHL_HUMAN	P53701	1.04	0.6327	0.8170	0.97	0.6651	0.9982	0.96	0.6956	0.9960	0.94	0.4338	0.9939	1.02	0.7912	0.9951	1.05	0.6214	0.9856	0.97	0.6827	0.9001	0.94	0.4531	0.6674	1.05	0.5170	0.9889	1.15	0.0812	0.9607
Cytochrome c1, heme protein, mitochondrial	CY1_HUMAN	P08574	1.05	0.6033	0.7965	1.07	0.4312	0.9982	1.04	0.6675	0.9960	0.92	0.3172	0.9939	1.03	0.7119	0.9951	0.96	0.7008	0.9857	1.03	0.7517	0.9282	0.86	0.1079	0.3341	1.04	0.6650	0.9889	1.04	0.6749	0.9881
Cytoplasmic aconitate hydratase	ACOC_HUMAN	P21399	1.22	0.0347	0.2873	1.02	0.8152	0.9982	0.90	0.3170	0.9960	0.85	0.0880	0.9874	0.98	0.8126	0.9951	1.22	0.0905	0.9856	0.88	0.1877	0.7149	1.19	0.1079	0.3341	0.99	0.9045	0.9889	1.03	0.7576	0.9881
Cytoplasmic dynein 1 heavy chain 1	DYHC1_HUMAN	Q14204	1.04	0.5091	0.7292	0.98	0.7676	0.9982	1.15	0.0687	0.9960	1.05	0.4165	0.9939	1.04	0.5745	0.9951	0.89	0.1479	0.9856	1.01	0.9155	0.9848	1.12	0.1184	0.3513	1.05	0.4579	0.9889	1.05	0.4505	0.9607
Cytoplasmic dynein 1 intermediate chain 1	DC1I1_HUMAN	O14576	1.04	0.5692	0.7686	1.01	0.8687	0.9982	0.94	0.3827	0.9960	1.13	0.0584	0.9874	1.02	0.7704	0.9951	1.01	0.8884	0.9959	1.02	0.7453	0.9262	1.11	0.1548	0.4044	1.01	0.8323	0.9889	1.00	0.9468	0.9938
Cytoplasmic dynein 1 intermediate chain 2	DC1I2_HUMAN	Q13409	0.93	0.1329	0.3961	0.95	0.3391	0.9982	1.05	0.3922	0.9960	1.09	0.0834	0.9874	0.99	0.7940	0.9951	0.94	0.3265	0.9856	0.97	0.5681	0.8601	1.01	0.8369	0.9133	0.97	0.5687	0.9889	0.95	0.3643	0.9607
Cytoplasmic dynein 1 light intermediate chain 1	DC1L1_HUMAN	Q9Y6G9	1.06	0.3373	0.6020	0.98	0.7682	0.9982	1.04	0.5859	0.9960	1.10	0.0923	0.9874	1.05	0.3933	0.9951	0.93	0.3220	0.9856	0.92	0.1423	0.6871	1.07	0.2717	0.5288	0.96	0.4439	0.9889	1.05	0.3419	0.9607
Cytoplasmic dynein 1 light intermediate chain 2	DC1L2_HUMAN	O43237	0.91	0.1421	0.4043	1.01	0.9312	0.9982	0.96	0.5478	0.9960	1.12	0.0654	0.9874	1.01	0.8656	0.9951	1.06	0.4677	0.9856	1.13	0.0640	0.6824	0.94	0.3708	0.6002	0.98	0.7691	0.9889	0.95	0.4076	0.9607
Cytoskeleton-associated protein 4	CKAP4_HUMAN	Q07065	0.80	0.0390	0.2873	1.09	0.4575	0.9982	0.98	0.8750	0.9960	1.01	0.9049	0.9939	0.95	0.6316	0.9951	1.02	0.9099	0.9959	1.15	0.2344	0.7187	0.66	0.0006	0.0690	1.03	0.8155	0.9889	0.95	0.6738	0.9881
Cytosol aminopeptidase	AMPL_HUMAN	P28838	1.03	0.5463	0.7505	1.03	0.5236	0.9982	0.94	0.2838	0.9960	1.05	0.3215	0.9939	1.06	0.2839	0.9951	1.11	0.1016	0.9856	1.11	0.0596	0.6824	0.96	0.4662	0.6760	1.01	0.9074	0.9889	0.98	0.7433	0.9881
Cytosolic non-specific dipeptidase	CNDP2_HUMAN	Q96KP4	1.08	0.2103	0.4843	1.00	0.9865	0.9985	1.01	0.8368	0.9960	0.97	0.6260	0.9939	1.00	0.9484	0.9951	1.02	0.8376	0.9959	0.99	0.8767	0.9720	1.05	0.4821	0.6855	0.94	0.3369	0.9889	0.95	0.4240	0.9607
D-beta-hydroxybutyrate dehydrogenase, mitochondrial	BDH_HUMAN	Q02338	1.09	0.3801	0.6372	1.02	0.8302	0.9982	1.09	0.4354	0.9960	1.01	0.9167	0.9939	1.02	0.8748	0.9951	0.88	0.2759	0.9856	0.90	0.3062	0.7378	1.23	0.0556	0.2468	1.07	0.5180	0.9889	1.01	0.9064	0.9938
D-dopachrome decarboxylase	DOPD_HUMAN	P30046	1.11	0.3297	0.5968	1.02	0.8824	0.9982	1.00	0.9845	0.9960	0.83	0.0735	0.9874	0.92	0.4492	0.9951	1.07	0.6099	0.9856	0.89	0.2862	0.7212	1.31	0.0200	0.1655	0.89	0.3033	0.9889	0.92	0.4336	0.9607
D-glutamate cyclase, mitochondrial	GLUCM_HUMAN	Q7Z3D6	1.18	0.3416	0.6072	0.72	0.0545	0.9982	0.96	0.8332	0.9960	1.04	0.7963	0.9939	1.15	0.4189	0.9951	1.03	0.8937	0.9959	0.82	0.2866	0.7212	1.33	0.1291	0.3656	0.84	0.3140	0.9889	1.20	0.2897	0.9607
DDB1- and CUL4-associated factor 8	DCAF8_HUMAN	Q5TAQ9	1.09	0.0987	0.3566	1.06	0.2685	0.9982	0.96	0.5364	0.9960	1.02	0.7532	0.9939	0.98	0.7126	0.9951	1.04	0.5389	0.9856	0.88	0.0254	0.6824	1.05	0.4271	0.6497	1.01	0.9169	0.9889	1.02	0.7071	0.9881
Deaminated glutathione amidase	NIT1_HUMAN	Q86X76	1.03	0.6552	0.8297	0.89	0.1138	0.9982	1.06	0.4791	0.9960	1.01	0.8508	0.9939	1.02	0.7994	0.9951	0.98	0.8182	0.9959	0.95	0.5266	0.8558	1.11	0.2107	0.4775	0.93	0.3136	0.9889	1.02	0.7443	0.9881
Decorin	PGS2_HUMAN	P07585	0.48	0.0084	0.2530	1.06	0.8269	0.9982	0.64	0.1764	0.9960	0.82	0.4837	0.9939	0.66	0.1571	0.9951	1.38	0.3536	0.9856	1.66	0.0891	0.6824	0.50	0.0267	0.1844	1.14	0.6591	0.9889	0.87	0.6335	0.9881
Dehydrogenase/reductase SDR family member 4	DHRS4_HUMAN	Q9BTZ2	1.05	0.4968	0.7242	0.94	0.3770	0.9982	1.06	0.4482	0.9960	1.01	0.8953	0.9939	0.99	0.9255	0.9951	0.93	0.4163	0.9856	1.03	0.6738	0.8951	0.95	0.5584	0.7400	0.99	0.9373	0.9922	1.12	0.1082	0.9607
Dehydrogenase/reductase SDR family member 7	DHRS7_HUMAN	Q9Y394	0.78	0.0022	0.2005	1.00	0.9582	0.9982	1.08	0.4032	0.9960	1.03	0.6826	0.9939	0.93	0.3711	0.9951	0.88	0.1936	0.9856	1.08	0.3607	0.7659	0.81	0.0175	0.1636	0.94	0.4910	0.9889	0.91	0.2252	0.9607
Delta-1-pyrroline-5-carboxylate dehydrogenase, mitochondrial	AL4A1_HUMAN	P30038	1.06	0.4955	0.7237	1.00	0.9889	0.9985	0.99	0.8765	0.9960	1.12	0.1789	0.9939	1.05	0.5715	0.9951	1.04	0.6957	0.9856	0.97	0.7749	0.9323	1.16	0.1118	0.3401	0.98	0.8271	0.9889	1.01	0.9154	0.9938
Delta-aminolevulinic acid dehydratase	HEM2_HUMAN	P13716	1.05	0.6318	0.8170	0.91	0.3343	0.9982	1.17	0.1615	0.9960	1.29	0.0064	0.9874	1.06	0.5780	0.9951	0.95	0.6725	0.9856	0.92	0.3930	0.7856	1.08	0.4902	0.6913	0.88	0.1932	0.9889	0.99	0.9098	0.9938
Delta-sarcoglycan	SGCD_HUMAN	Q92629	0.90	0.0891	0.3410	0.97	0.6489	0.9982	1.07	0.3918	0.9960	1.04	0.4896	0.9939	1.00	0.9842	0.9964	0.91	0.2371	0.9856	1.02	0.7839	0.9373	0.89	0.0880	0.2995	0.97	0.6090	0.9889	0.97	0.6394	0.9881
Delta(3,5)-Delta(2,4)-dienoyl-CoA isomerase, mitochondrial	ECH1_HUMAN	Q13011	1.24	0.0362	0.2873	1.14	0.1991	0.9982	0.93	0.5521	0.9960	1.14	0.1945	0.9939	1.11	0.3570	0.9951	1.22	0.1198	0.9856	1.06	0.6120	0.8735	1.11	0.3555	0.5920	0.95	0.6256	0.9889	1.06	0.5662	0.9881
Deoxyuridine 5'-triphosphate nucleotidohydrolase, mitochondrial	DUT_HUMAN	P33316	1.15	0.0493	0.2898	1.06	0.4459	0.9982	0.98	0.8530	0.9960	0.92	0.2320	0.9939	1.01	0.8763	0.9951	1.00	0.9554	0.9967	1.00	0.9469	0.9908	0.88	0.1114	0.3394	0.98	0.7582	0.9889	1.01	0.8488	0.9881
Dermcidin	DCD_HUMAN	P81605	1.04	0.7405	0.8802	1.07	0.6173	0.9982	1.11	0.4783	0.9960	1.02	0.8909	0.9939	0.94	0.6337	0.9951	0.84	0.2482	0.9856	0.82	0.1354	0.6871	1.12	0.4213	0.6447	1.00	0.9837	0.9976	1.03	0.8272	0.9881
Desmin	DESM_HUMAN	P17661	0.81	0.0224	0.2873	1.06	0.5567	0.9982	1.03	0.8108	0.9960	0.99	0.9326	0.9939	0.91	0.3155	0.9951	0.92	0.4976	0.9856	0.95	0.6061	0.8689	0.81	0.0410	0.2167	1.10	0.3337	0.9889	1.04	0.6787	0.9881
Desmocollin-2	DSC2_HUMAN	Q02487	0.93	0.4180	0.6675	0.99	0.9339	0.9982	1.08	0.4936	0.9960	0.96	0.6224	0.9939	0.99	0.8761	0.9951	0.96	0.6886	0.9856	0.95	0.6012	0.8652	1.14	0.1990	0.4593	1.12	0.2353	0.9889	1.03	0.7508	0.9881
Desmoglein-2	DSG2_HUMAN	Q14126	1.05	0.4736	0.7121	1.06	0.3723	0.9982	0.98	0.8175	0.9960	1.00	0.9697	0.9961	1.07	0.3426	0.9951	0.96	0.6088	0.9856	0.92	0.2797	0.7212	0.95	0.5119	0.7066	1.10	0.1759	0.9889	1.12	0.0893	0.9607
Desmoplakin	DESP_HUMAN	P15924	1.00	0.9877	0.9915	1.25	0.0348	0.9982	1.08	0.5114	0.9960	0.99	0.9133	0.9939	0.94	0.6069	0.9951	0.86	0.2675	0.9856	0.85	0.1479	0.6871	0.83	0.1206	0.3537	1.14	0.2285	0.9889	1.10	0.3591	0.9607
Destrin	DEST_HUMAN	P60981	0.92	0.1789	0.4545	1.07	0.2789	0.9982	1.05	0.4533	0.9960	0.93	0.2211	0.9939	0.82	0.0016	0.9389	1.01	0.9191	0.9959	0.86	0.0183	0.6779	1.13	0.0704	0.2713	0.94	0.3303	0.9889	0.88	0.0332	0.9607
Diablo homolog, mitochondrial	DBLOH_HUMAN	Q9NR28	1.08	0.2250	0.4984	1.04	0.5690	0.9982	0.96	0.5613	0.9960	1.04	0.5626	0.9939	1.01	0.8592	0.9951	1.04	0.6307	0.9856	0.88	0.0459	0.6824	1.06	0.4346	0.6551	1.07	0.3031	0.9889	1.05	0.4390	0.9607
Dihydrolipoyl dehydrogenase, mitochondrial	DLDH_HUMAN	P09622	1.14	0.0793	0.3281	1.03	0.7197	0.9982	0.93	0.4091	0.9960	1.04	0.5693	0.9939	1.03	0.6751	0.9951	1.06	0.5304	0.9856	0.94	0.4609	0.8243	1.11	0.2177	0.4836	1.09	0.2673	0.9889	1.06	0.4579	0.9607
Dihydrolipoyllysine-residue acetyltransferase component of pyruvate deh	ODP2_HUMAN	P10515	1.00	0.9987	0.9993	1.03	0.6236	0.9982	1.03	0.6708	0.9960	0.97	0.6008	0.9939	0.98	0.7938	0.9951	0.91	0.2270	0.9856	0.96	0.5100	0.8517	1.05	0.5327	0.7227	1.08	0.2234	0.9889	1.04	0.5154	0.9846
Dihydrolipoyllysine-residue succinyltransferase component of 2-oxogluta	ODO2_HUMAN	P36957	0.95	0.3040	0.5744	1.00	0.9853	0.9985	0.99	0.8091	0.9960	0.98	0.6394	0.9939	0.94	0.2572	0.9951	0.99	0.8580	0.9959	1.00	0.9972	0.9992	1.03	0.6271	0.7896	1.17	0.0018	0.9889	1.01	0.8509	0.9881
Dihydroorotate dehydrogenase (quinone), mitochondrial	PYRD_HUMAN	Q02127	1.06	0.3622	0.6254	1.11	0.1181	0.9982	1.14	0.0808	0.9960	0.95	0.4265	0.9939	1.03	0.6340	0.9951	0.87	0.0816	0.9856	0.95	0.4721	0.8308	0.96	0.5844	0.7608	1.05	0.4792	0.9889	1.07	0.2985	0.9607
Dihydropteridine reductase	DHPR_HUMAN	P09417	1.21	0.0251	0.2873	1.10	0.2895	0.9982	0.93	0.5024	0.9960	0.93	0.3793	0.9939	0.98	0.8066	0.9951	1.12	0.2803	0.9856	0.88	0.1656	0.6896	1.11	0.2737	0.5288	1.00	0.9746	0.9976	1.00	0.9760	0.9992
Dihydropyrimidinase-related protein 2	DPYL2_HUMAN	Q16555	1.03	0.6094	0.8000	1.03	0.6203	0.9982	1.01	0.8507	0.9960	1.05	0.4387	0.9939	0.98	0.7940	0.9951	1.03	0.6975	0.9856	1.06	0.3478	0.7593	0.95	0.4881	0.6896	1.01	0.8916	0.9889	0.98	0.7004	0.9881
Dihydropyrimidinase-related protein 3	DPYL3_HUMAN	Q14195	1.04	0.7263	0.8738	0.96	0.7417	0.9982	0.90	0.4220	0.9960	1.06	0.6308	0.9939	1.01	0.9603	0.9951	1.14	0.3661	0.9856	1.07	0.5871	0.8645	0.79	0.0675	0.2660	1.01	0.9180	0.9889	0.99	0.9193	0.9938
Dipeptidyl peptidase 2	DPP2_HUMAN	Q9UHL4	1.16	0.1572	0.4259	1.11	0.3148	0.9982	0.86	0.2245	0.9960	0.77	0.0112	0.9874	0.88	0.2441	0.9951	1.17	0.2251	0.9856	1.03	0.8140	0.9478	1.06	0.6415	0.7985	0.97	0.7889	0.9889	0.98	0.8520	0.9881
Disks large homolog 1	DLG1_HUMAN	Q12959	0.99	0.8677	0.9356	0.94	0.3698	0.9982	1.18	0.0419	0.9960	0.92	0.2419	0.9939	0.97	0.6478	0.9951	0.82	0.0250	0.9411	1.04	0.6481	0.8897	1.05	0.5329	0.7227	1.08	0.3004	0.9889	0.94	0.3918	0.9607
DNA damage-binding protein 1	DDB1_HUMAN	Q16531	1.09	0.1280	0.3939	1.06	0.3275	0.9982	1.03	0.6154	0.9960	0.97	0.5469	0.9939	0.96	0.5026	0.9951	0.97	0.6120	0.9856	0.91	0.0973	0.6824	1.11	0.1002	0.3230	0.97	0.5454	0.9889	0.99	0.8204	0.9881
DnaJ homolog subfamily A member 2	DNJA2_HUMAN	O60884	0.95	0.3960	0.6532	0.99	0.8717	0.9982	0.96	0.6120	0.9960	0.97	0.6753	0.9939	1.02	0.7431	0.9951	1.03	0.7054	0.9857	1.00	0.9420	0.9903	1.01	0.9014	0.9448	1.04	0.5352	0.9889	1.11	0.0767	0.9607
DnaJ homolog subfamily B member 11	DJB11_HUMAN	Q9UBS4	0.88	0.0314	0.2873	0.97	0.6011	0.9982	1.04	0.5284	0.9960	1.03	0.6000	0.9939	0.97	0.6721	0.9951	0.96	0.5434	0.9856	1.06	0.3677	0.7673	0.95	0.3852	0.6146	0.89	0.0456	0.9889	0.94	0.3116	0.9607
DnaJ homolog subfamily B member 4	DNJB4_HUMAN	Q9UDY4	0.89	0.0994	0.3570	0.97	0.6875	0.9982	1.00	0.9739	0.9960	0.99	0.9129	0.9939	0.90	0.1465	0.9951	0.90	0.2492	0.9856	0.99	0.9186	0.9848	0.89	0.1669	0.4197	1.02	0.8263	0.9889	0.95	0.4822	0.9652
DnaJ homolog subfamily B member 6	DNJB6_HUMAN	O75190	0.93	0.2202	0.4926	1.07	0.2800	0.9982	1.01	0.8876	0.9960	0.99	0.8674	0.9939	0.91	0.1420	0.9951	0.96	0.5899	0.9856	1.00	0.9560	0.9923	1.03	0.7126	0.8360	0.98	0.6920	0.9889	0.98	0.6903	0.9881

Dolichyl-diphosphooligosaccharide--protein glycosyltransferase 48 kDa s	OST48_HUMAN	P39656	0.89	0.1598	0.4312	1.09	0.3121	0.9982	0.96	0.6562	0.9960	0.96	0.6475	0.9939	0.94	0.4522	0.9951	0.97	0.8022	0.9959	1.10	0.3067	0.7378	0.85	0.0831	0.2940	0.97	0.6846	0.9889	0.94	0.4687	0.9607
Dolichyl-diphosphooligosaccharide--protein glycosyltransferase subunit	RPN1_HUMAN	P04843	0.91	0.1604	0.4314	0.99	0.9093	0.9982	1.07	0.4008	0.9960	1.07	0.3184	0.9939	1.03	0.6579	0.9951	0.90	0.2179	0.9856	1.08	0.2700	0.7187	0.89	0.1129	0.3426	0.96	0.5196	0.9889	0.99	0.8630	0.9881
Dolichyl-diphosphooligosaccharide--protein glycosyltransferase subunit	RPN2_HUMAN	P04844	0.81	0.0155	0.2873	1.03	0.7453	0.9982	1.10	0.3535	0.9960	0.99	0.9267	0.9939	0.91	0.3285	0.9951	0.88	0.2559	0.9856	1.07	0.4633	0.8244	0.85	0.0997	0.3221	0.97	0.7757	0.9889	0.91	0.2689	0.9607
Dual specificity protein phosphatase 3	DUS3_HUMAN	P51452	1.11	0.1050	0.3654	1.09	0.2136	0.9982	0.96	0.6216	0.9960	0.96	0.5450	0.9939	1.06	0.3961	0.9951	1.07	0.4362	0.9856	1.02	0.8029	0.9456	0.96	0.6233	0.7880	0.94	0.3311	0.9889	0.99	0.8462	0.9881
Dynactin subunit 1	DCTN1_HUMAN	Q14203	1.01	0.8068	0.9060	1.02	0.6836	0.9982	1.04	0.3552	0.9960	1.03	0.4184	0.9939	1.00	0.9320	0.9951	0.93	0.1418	0.9856	0.98	0.6417	0.8892	0.99	0.7395	0.8574	0.98	0.6126	0.9889	1.00	0.9332	0.9938
Dynactin subunit 2	DCTN2_HUMAN	Q13561	1.01	0.8357	0.9201	0.95	0.2048	0.9982	1.05	0.3567	0.9960	1.03	0.4492	0.9939	0.99	0.7566	0.9951	0.94	0.2460	0.9856	0.95	0.2590	0.7187	1.04	0.3827	0.6130	0.95	0.2231	0.9889	1.00	0.9342	0.9938
Dynactin subunit 3	DCTN3_HUMAN	O75935	1.07	0.3655	0.6279	1.01	0.9094	0.9982	1.07	0.4311	0.9960	1.01	0.9122	0.9939	0.95	0.5217	0.9951	0.86	0.0880	0.9856	0.89	0.1419	0.6871	1.11	0.1806	0.4355	0.91	0.1900	0.9889	0.99	0.8411	0.9881
Dynactin subunit 4	DCTN4_HUMAN	Q9UJW0	0.92	0.1162	0.3780	0.92	0.1396	0.9982	1.17	0.0083	0.9960	1.09	0.1159	0.9874	1.03	0.6176	0.9951	0.82	0.0016	0.6489	1.02	0.6845	0.9004	0.98	0.7344	0.8540	0.98	0.6482	0.9889	1.03	0.6073	0.9881
Dynamin-1-like protein	DNM1L_HUMAN	O00429	1.04	0.5452	0.7505	1.02	0.7220	0.9982	1.03	0.6675	0.9960	1.01	0.8834	0.9939	0.96	0.5213	0.9951	0.96	0.6168	0.9856	0.93	0.2649	0.7187	1.07	0.2805	0.5337	1.01	0.9109	0.9889	0.93	0.2420	0.9607
Dynamin-2	DYN2_HUMAN	P50570	0.97	0.7228	0.8735	1.19	0.0507	0.9982	0.97	0.7622	0.9960	1.01	0.9433	0.9939	0.87	0.1316	0.9951	1.01	0.9605	0.9967	0.95	0.5866	0.8645	0.85	0.0952	0.3123	1.11	0.2295	0.9889	0.84	0.0408	0.9607
Dynamin-like 120 kDa protein, mitochondrial	OPA1_HUMAN	O60313	1.07	0.4034	0.6612	0.99	0.8970	0.9982	1.00	0.9996	0.9996	1.02	0.8209	0.9939	1.07	0.3727	0.9951	0.97	0.7522	0.9857	0.92	0.3103	0.7378	1.17	0.0671	0.2650	1.10	0.2437	0.9889	1.12	0.1296	0.9607
Dynein light chain 2, cytoplasmic	DYL2_HUMAN	Q96FJ2	1.12	0.0889	0.3410	1.10	0.1771	0.9982	1.05	0.5396	0.9960	0.96	0.5720	0.9939	0.96	0.5348	0.9951	0.95	0.5282	0.9856	0.85	0.0297	0.6824	1.13	0.1224	0.3555	0.98	0.7924	0.9889	1.00	0.9941	0.9992
Dynein light chain roadblock-type 1	DLRB1_HUMAN	Q9NP97	1.11	0.0815	0.3290	1.07	0.2904	0.9982	1.10	0.1796	0.9960	1.04	0.5427	0.9939	1.02	0.7391	0.9951	0.90	0.1649	0.9856	1.00	0.9401	0.9903	0.97	0.7132	0.8360	0.94	0.3473	0.9889	0.99	0.8504	0.9881
Dysferlin	DYSF_HUMAN	O75923	0.89	0.1051	0.3654	0.96	0.6216	0.9982	1.26	0.0042	0.9960	1.02	0.7810	0.9939	0.93	0.3072	0.9951	0.81	0.0144	0.9411	0.94	0.3874	0.7824	1.09	0.2962	0.5464	0.89	0.1039	0.9889	0.89	0.1109	0.9607
Dystrobrevin alpha	DTNA_HUMAN	Q9Y4J8	1.08	0.1918	0.4689	1.03	0.6257	0.9982	1.04	0.5288	0.9960	1.09	0.1581	0.9874	1.01	0.8530	0.9951	0.92	0.2178	0.9856	0.94	0.3078	0.7378	0.95	0.4805	0.6845	1.04	0.5557	0.9889	1.02	0.7701	0.9881
Dystroglycan	DAG1_HUMAN	Q14118	0.90	0.0917	0.3466	0.92	0.2006	0.9982	1.14	0.0785	0.9960	0.95	0.4561	0.9939	0.96	0.5238	0.9951	0.86	0.0646	0.9641	1.00	0.9754	0.9939	0.92	0.2802	0.5337	0.92	0.2220	0.9889	0.92	0.2352	0.9607
Dystrophin	DMD_HUMAN	P11532	1.02	0.7490	0.8824	1.03	0.7012	0.9982	1.09	0.2778	0.9960	0.90	0.1214	0.9874	0.91	0.1837	0.9951	0.93	0.3370	0.9856	0.80	0.0008	0.6779	1.13	0.0959	0.3130	1.03	0.6254	0.9889	1.02	0.8152	0.9881
E3 ubiquitin-protein ligase HUWE1	HUWE1_HUMAN	Q7Z6Z7	0.95	0.4540	0.6965	1.01	0.9371	0.9982	1.13	0.1746	0.9960	0.94	0.3743	0.9939	0.93	0.3548	0.9951	0.88	0.1865	0.9856	1.08	0.3342	0.7458	1.01	0.9362	0.9616	0.98	0.7485	0.9889	0.90	0.1672	0.9607
E3 UFM1-protein ligase 1	UFL1_HUMAN	O94874	0.98	0.7499	0.8824	0.92	0.2698	0.9982	0.93	0.4297	0.9960	1.11	0.1707	0.9874	1.11	0.1991	0.9951	1.04	0.6601	0.9856	1.09	0.2900	0.7212	1.17	0.0655	0.2631	0.87	0.0717	0.9889	1.05	0.5116	0.9846
Early endosome antigen 1	EEA1_HUMAN	Q15075	0.93	0.4670	0.7074	1.00	0.9694	0.9982	1.06	0.5778	0.9960	1.14	0.1393	0.9874	0.99	0.9436	0.9951	0.94	0.5834	0.9856	1.06	0.5271	0.8558	0.86	0.1306	0.3685	1.11	0.2672	0.9889	1.04	0.6472	0.9881
Echinoderm microtubule-associated protein-like 1	EMAL1_HUMAN	O00423	1.08	0.3711	0.6312	0.91	0.2666	0.9982	1.07	0.4695	0.9960	1.10	0.2199	0.9939	1.00	0.9861	0.9964	1.03	0.7524	0.9857	1.05	0.6001	0.8646	1.19	0.0548	0.2454	0.93	0.3790	0.9889	0.96	0.6106	0.9881
Echinoderm microtubule-associated protein-like 2	EMAL2_HUMAN	O95834	1.19	0.0502	0.2898	1.10	0.2775	0.9982	1.00	0.9922	0.9960	1.02	0.7959	0.9939	1.04	0.7002	0.9951	1.04	0.7531	0.9857	0.90	0.2826	0.7212	1.10	0.3437	0.5779	0.98	0.8416	0.9889	1.03	0.7552	0.9881
Ectonucleoside triphosphate diphosphohydrolase 1	ENTP1_HUMAN	P49961	0.86	0.0966	0.3563	1.10	0.2883	0.9982	0.98	0.8850	0.9960	0.96	0.6760	0.9939	0.90	0.2801	0.9951	1.03	0.7710	0.9857	1.17	0.1086	0.6824	0.77	0.0081	0.1361	0.95	0.5852	0.9889	0.91	0.2868	0.9607
EF-hand domain-containing protein D1	EFHD1_HUMAN	Q9BUP0	1.12	0.2219	0.4949	1.06	0.5526	0.9982	0.97	0.7596	0.9960	0.99	0.9395	0.9939	1.05	0.6062	0.9951	1.03	0.7815	0.9897	1.08	0.4466	0.8181	1.02	0.8447	0.9165	1.02	0.8580	0.9889	1.13	0.1859	0.9607
EH domain-containing protein 1	EHD1_HUMAN	Q9H4M9	1.01	0.9210	0.9555	0.94	0.3258	0.9982	0.95	0.4700	0.9960	1.01	0.8840	0.9939	1.02	0.7679	0.9951	1.01	0.9033	0.9959	0.97	0.6497	0.8898	1.15	0.0340	0.2017	0.87	0.0156	0.9889	0.96	0.4833	0.9652
EH domain-containing protein 2	EHD2_HUMAN	Q9NZN4	1.02	0.7854	0.8986	1.00	0.9703	0.9982	0.96	0.5904	0.9960	1.00	0.9461	0.9939	1.04	0.5701	0.9951	1.01	0.9087	0.9959	1.04	0.5764	0.8630	1.08	0.2663	0.5282	0.97	0.6477	0.9889	1.03	0.6453	0.9881
EH domain-containing protein 4	EHD4_HUMAN	Q9H223	1.02	0.7707	0.8895	0.94	0.2608	0.9982	1.04	0.5857	0.9960	1.06	0.3420	0.9939	1.08	0.2161	0.9951	0.89	0.1055	0.9856	0.98	0.7731	0.9317	1.05	0.4185	0.6429	0.94	0.3435	0.9889	1.02	0.6733	0.9881
Electron transfer flavoprotein subunit alpha, mitochondrial	ETFA_HUMAN	P13804	1.17	0.0372	0.2873	1.06	0.4448	0.9982	0.91	0.2734	0.9960	1.00	0.9527	0.9939	0.97	0.7135	0.9951	1.11	0.2575	0.9856	0.95	0.5375	0.8573	1.07	0.4210	0.6447	1.03	0.6709	0.9889	0.99	0.9142	0.9938
Electron transfer flavoprotein subunit beta	ETFB_HUMAN	P38117	1.17	0.0450	0.2894	1.06	0.4340	0.9982	0.89	0.1827	0.9960	1.01	0.9466	0.9939	0.96	0.6613	0.9951	1.14	0.1632	0.9856	0.94	0.4299	0.8104	1.09	0.3064	0.5503	1.07	0.3823	0.9889	1.00	0.9875	0.9992
Electron transfer flavoprotein-ubiquinone oxidoreductase, mitochondria	ETFD_HUMAN	Q16134	1.11	0.1565	0.4253	1.15	0.0525	0.9982	1.05	0.5751	0.9960	1.12	0.1156	0.9874	1.03	0.6646	0.9951	0.88	0.1638	0.9856	1.06	0.4474	0.8181	0.97	0.7540	0.8652	1.01	0.8566	0.9889	1.03	0.6976	0.9881
Elongation factor 1-alpha 1	EF1A1_HUMAN	P68104	0.79	0.0270	0.2873	1.00	0.9727	0.9982	1.01	0.9184	0.9960	1.04	0.7271	0.9939	0.90	0.3546	0.9951	1.02	0.8906	0.9959	1.13	0.2774	0.7212	0.88	0.3064	0.5503	1.08	0.4866	0.9889	0.94	0.5612	0.9881
Elongation factor 1-alpha 2	EF1A2_HUMAN	Q05639	0.92	0.0997	0.3570	1.01	0.8935	0.9982	1.06	0.2815	0.9960	0.96	0.4080	0.9939	0.90	0.0342	0.9951	0.96	0.5102	0.9856	0.98	0.6744	0.8951	1.07	0.2444	0.5087	0.96	0.4372	0.9889	0.89	0.0200	0.9607
Elongation factor 1-beta	EF1B_HUMAN	P24534	1.04	0.4379	0.6839	1.02	0.7599	0.9982	1.05	0.3785	0.9960	1.09	0.0947	0.9874	1.06	0.2698	0.9951	0.94	0.3068	0.9856	1.00	0.9793	0.9960	0.94	0.3043	0.5503	1.02	0.6895	0.9889	1.02	0.7028	0.9881
Elongation factor 1-delta	EF1D_HUMAN	P29692	1.05	0.3310	0.5968	1.00	0.9384	0.9982	1.05	0.3909	0.9960	1.13	0.0220	0.9874	1.12	0.0421	0.9951	0.94	0.3621	0.9856	1.04	0.5143	0.8536	0.89	0.0521	0.2413	1.01	0.9010	0.9889	1.06	0.2689	0.9607
Elongation factor 1-gamma	EF1G_HUMAN	P26641	1.03	0.5932	0.7894	1.00	0.9334	0.9982	1.08	0.1685	0.9960	1.11	0.0287	0.9874	1.04	0.4681	0.9951	0.92	0.1761	0.9856	1.04	0.3868	0.7822	0.98	0.6891	0.8252	1.00	0.9899	0.9976	1.00	0.9429	0.9938
Elongation factor 2	EF2_HUMAN	P13639	0.99	0.9075	0.9509	0.99	0.8789	0.9982	1.07	0.2046	0.9960	0.99	0.7987	0.9939	0.94	0.1597	0.9951	0.97	0.6373	0.9856	1.01	0.9027	0.9831	1.05	0.3277	0.5681	0.93	0.0999	0.9889	0.93	0.1016	0.9607
Elongation factor G, mitochondrial	EFGM_HUMAN	Q96RP9	1.06	0.3688	0.6289	0.98	0.7310	0.9982	0.88	0.1018	0.9960	1.00	0.9700	0.9961	1.02	0.7995	0.9951	1.14	0.1128	0.9856	0.96	0.5709	0.8612	0.99	0.8442	0.9165	1.08	0.2318	0.9889	1.05	0.4383	0.9607
Elongation factor Tu, mitochondrial	EFTU_HUMAN	P49411	1.07	0.2543	0.5233	0.95	0.3722	0.9982	0.95	0.4586	0.9960	1.01	0.9281	0.9939	1.09	0.1848	0.9951	1.00	0.9751	0.9967	1.01	0.9165	0.9848	1.03	0.6557	0.8033	1.13	0.0421	0.9889	1.12	0.0625	0.9607
Elongin-B	ELOB_HUMAN	Q15370	1.08	0.2323	0.5066	0.98	0.7079	0.9982	0.94	0.4254	0.9960	1.02	0.8161	0.9939	1.04	0.6158	0.9951	1.09	0.2853	0.9856	0.84	0.0095	0.6779	1.19	0.0145	0.1589	0.98	0.7682	0.9889	1.06	0.3625	0.9607
Elongin-C	ELOC_HUMAN	Q15369	1.21	0.0339	0.2873	0.92	0.3777	0.9982	1.07	0.5166	0.9960	1.02	0.8215	0.9939	1.20	0.0481	0.9951	0.99	0.8959	0.9959	0.96	0.7069	0.9121	0.95	0.6351	0.7937	0.88	0.1482	0.9889	1.18	0.0642	0.9607
Emerin	EMD_HUMAN	P50402	0.89	0.0851	0.3348	1.01	0.8495	0.9982	1.05	0.5242	0.9960	0.91	0.2038	0.9939	0.93	0.2987	0.9951	0.92	0.3555	0.9856	1.13	0.0946	0.6824	0.89	0.1238	0.3569	0.93	0.3239	0.9889	0.98	0.7681	0.9881
EMILIN-2	EMIL2_HUMAN	Q9BXX0	1.01	0.9160	0.9536	1.00	0.9847	0.9985	1.11	0.4895	0.9960	0.99	0.9240	0.9939	1.07	0.6036	0.9951	0.77	0.0974	0.9856	0.86	0.2803	0.7212	1.01	0.9598	0.9773	0.87	0.2824	0.9889	0.97	0.8203	0.9881
Endoglin	EGLN_HUMAN	P17813	0.94	0.4857	0.7203	1.02	0.8209	0.9982	0.94	0.5839	0.9960	1.02	0.8463	0.9939	1.05	0.5819	0.9951	1.02	0.8544	0.9959	1.15	0.1528	0.6871	0.76	0.0056	0.1317	1.03	0.7917	0.9889	1.07	0.4770	0.9621
Endophilin-B1	SHLB1_HUMAN	Q9Y371	0.96	0.5445	0.7505	0.94	0.3057	0.9982	1.10	0.1747	0.9960	1.16	0.0110	0.9874	1.09	0.1712	0.9951	0.90	0.1647	0.9856	1.12	0.0730	0.6824	1.11	0.1028	0.3273	0.97	0.6609	0.9889	1.03	0.6640	0.9881
Endoplasmic reticulum aminopeptidase 1	ERAP1_HUMAN	Q9NZ08	1.07	0.4498	0.6950	1.08	0.3664	0.9982	1.07	0.5069	0.9960	1.08	0.3574	0.9939	1.07	0.4168	0.9951	1.02	0.8153	0.9959	1.11	0.2682	0.7187	1.08	0.4176	0.6425	1.04	0.6210	0.9889	0.91	0.2852	0.9607
Endoplasmic reticulum chaperone BiP	BIP_HUMAN	P11021	0.96	0.3732	0.6312	0.95	0.2852	0.9982	1.07	0.2010	0.9960	1.04	0.3883	0.9939	0.96	0.4595	0.9951	0.93	0.2460	0.9856	1.01	0.8972	0.9786	1.05	0.3296	0.5682	0.98	0.7472	0.9889	0.93	0.1093	0.9607
Endoplasmic reticulum lectin 1	ERLEC_HUMAN	Q96DZ1	1.04	0.5960	0.7918	0.98	0.7951	0.9982	1.13	0.2092	0.9960	1.04	0.5924	0.9939	1.07	0.4374	0.9951	0.84	0.0921	0.9856	1.04	0.6388	0.8892	0.91	0.3301	0.5682	1.01	0.9511	0.9945	1.00	0.9938	0.9992
Endoplasmic reticulum resident protein 29	ERP29_HUMAN	P30040	0.94	0.2340	0.5075	0.97	0.6121	0.9982	1.03	0.5744	0.9960	1.01	0.8218	0.9939	0.97	0.6133	0.9951	0.98	0.7693	0.9857	1.13	0.0205	0.6804	0.96	0.5264	0.7205	0.96	0.4897	0.9889	0.93	0.1639	0.9607
Endoplasmic reticulum resident protein 44	ERP44_HUMAN	Q9BS26	1.05	0.2926	0.5638	1.03	0.5623	0.9982	1.03	0.6442	0.9960	0.99	0.7877	0.9939	0.98	0.7242	0.9951	1.00	0.9826	0.9992	0.97	0.5793	0.8632	0.94	0.2969	0.5464	1.02	0.7008	0.9889	0.96	0.4712	0.9607
Endoplasmic reticulum-Golgi intermediate compartment protein 1	ERGI1_HUMAN	Q969X5	0.96	0.6875	0.8496	0.99	0.8878	0.9982	1.01	0.8920	0.9960	1.02	0.8147	0.9939	1.05	0.6164	0.9951	0.94	0.6096	0.9856	1.18	0.0918	0.6824	0.79	0.0239	0.1763	1.02	0.8733	0.9889	1.06	0.5088	0.9846
Endoplasmin	ENPL_HUMAN	P14625	0.94	0.2996	0.5710	0.97	0.6327	0.9982	1.06	0.3661	0.9960	1.08	0.1453	0.9874	0.98	0.6690	0.9951	0.93	0.2918	0.9856	1.09	0.1434	0.6871	0.96	0.5469	0.7334	0.95	0.3588	0.9889	0.91	0.0977	0.9607
Endothelial cell-selective adhesion molecule	ESAM_HUMAN	Q96AP7	1.05	0.3493	0.6117	1.03	0.5793	0.9982	0.97	0.5691	0.9960	1.05	0.3452	0.9939	1.10	0.0681	0.9951	1.07	0.2679	0.9856	1.09	0.1056	0.6824	0.89	0.0379	0.2131	1.05	0.3544	0.9889	1.07	0.1430	0.9607
Enhancer of rudimentary homolog	ERH_HUMAN	P84090	1.06	0.1845	0.4603	0.98	0.6773	0.9982	0.96	0.4034	0.9960	0.96	0.3663	0.9939	1.02	0.6473	0.9951	1.07	0.2018	0.9856	0.93	0.1281	0.6868	1.03	0.5999	0.7719	0.96	0.3992	0.9889	1.03	0.5236	0.9846
Enoyl-[acyl-carrier-protein] reductase, mitochondrial	MECR_HUMAN	Q9BV79	1.09	0.1771	0.4521	1.06	0.3913	0.9982	1.05	0.4904	0.9960	1.10	0.1131	0.9874	1.02	0.7330	0.9951	0.98	0.7529	0.9857	1.08	0.2610	0.7187	0.96	0.5980	0.7702	1.03	0.6301	0.9889	1.02	0.7419	0.9881
Enoyl-CoA delta isomerase 1, mitochondrial	ECI1_HUMAN	P42126	1.23	0.0339	0.2873	1.07	0.4885	0.9982	0.93	0.5038	0.9960	0.92	0.4035	0.9939	1.00	0.9626	0.9951	1.10	0.4169	0.9856	0.81	0.0431	0.6824	1.11	0.3337	0.5705	1.00	0.9624	0.9961	1.02	0.8032	0.9881
Enoyl-CoA delta isomerase 2, mitochondrial	ECI2_HUMAN	O75521	1.05	0.3684	0.6289	1.02	0.7777	0.9982	0.98	0.7447	0.9960	1.03	0.6081	0.9939	0.99	0.9315	0.9951	1.00	0.9722	0.9967	0.98	0.7975	0.9431	1.11	0.1312	0.3695	1.08	0.2062	0.9889	1.05	0.3871	0.9607
Enoyl-CoA hydratase domain-containing protein 2, mitochondrial	ECHD2_HUMAN	Q86YB7	1.15	0.1662	0.4371	0.97	0.7582	0.9982	1.05	0.6573	0.9960	1.02	0.8189	0.9939	0.98	0.8715	0.9951	0.99	0.9140	0.9959	0.94	0.5418	0.8573	1.23	0.0654	0.2631	0.96	0.6911	0.9889	0.97	0.7703	0.9881
Enoyl-CoA hydratase domain-containing protein 3, mitochondrial	ECHD3_HUMAN	Q96DC8	1.00	0.9910	0.9936	0.94	0.5543	0.9982	1.10	0.4459	0.9960	1.10	0.3952	0.9939	0.98	0.8868	0.9951	0.94	0.6657	0.9856	0.90	0.3641	0.7671	1.06	0.6086	0.7788	1.11	0.3544	0.9889	1.05	0.6664	0.9881
Enoyl-CoA hydratase, mitochondrial	ECHM_HUMAN	P30084	1.15	0.0771	0.3266	1.02	0.7655	0.9982	0.89	0.2024	0.9960	0.97	0.7271	0.9939	0.98	0.7950	0.9951	1.08	0.4475	0.9856	0.94	0.4537	0.8208	1.08	0.3738	0.6037	1.01	0.9035	0.9889	1.00	0.9559	0.9938
Epidermal growth factor receptor substrate 15-like 1	EP15R_HUMAN	Q9UBC2	0.96	0.4163	0.6668	1.02	0.7198	0.9982	1.05	0.4500	0.9960	1.11	0.0457	0.9874	1.02	0.7511	0.9951	1.00	0.9973	0.9992	1.14	0.0189	0.6779	0.99	0.9014	0.9448	1.04	0.5137	0.9889	0.97	0.5736	0.9881
Epimerase family protein SDR39U1	D39U1_HUMAN	Q9NRG7	1.03	0.7512	0.8824	0.87	0.1083	0.9982	1.05	0.6367	0.9960	1.05	0.5292	0.9939	1.01	0.8711	0.9951	0.90	0.3127	0.9856	0.98	0.8093	0.9473	1.24	0.0201	0.1655	0.93	0.3709	0.9889	1.00	0.9535	0.9938
Epoxide hydrolase 1	HYEP_HUMAN	P07099	0.84	0.0815	0.3290	1.06	0.5445	0.9982	1.09	0.4845	0.9960	1.11	0.2891	0.9939	0.92	0.4519	0.9951	0.82	0.1200	0.9856	1.33	0.0068	0.6779	0.76	0.0134	0.1569	0.85	0.1208	0.9889	0.88	0.1949	0.9607
Erlin-2	ERLN2_HUMAN	O94905	0.71	0.0003	0.1653	1.05	0.6276	0.9982	1.07	0.5611	0.9960	0.94	0.5044	0.9939	0.82	0.0580	0.9951	0.92	0.5244	0.9856	1.13	0.2429	0.7187	0.82	0.0823	0.2933	1.04	0.7267	0.9889	0.86	0.1227	0.9607
Erythrocyte band 7 integral membrane protein	STOM_HUMAN	P27105	0.85	0.1380	0.4015	1.11	0.3197	0.9982	1.08	0.5410	0.9960	0.90	0.3078	0.9939	0.97	0.8083	0.9951	0.88	0.3373	0.9856	1.17	0.1569	0.6871	0.75	0.0130	0.1544	0.99	0.9013	0.9889	0.92	0.4469	0.9607
Estradiol 17-beta-dehydrogenase 8	DHB8_HUMAN	Q92506	1.03	0.6320	0.8170	1.02	0.7096	0.9982	1.14	0.0616	0.9960	0.94	0.2712	0.9939	0.93	0.2181	0.9951	0.87	0.0519	0.9621	0.96	0.5690	0.8601	1.02	0.7565	0.8668	1.11	0.0826	0.9889	0.96	0.4709	0.9607
Ethanolamine-phosphate cytidylyltransferase	PCY2_HUMAN	Q99447	1.07	0.4161	0.6668	1.01	0.9280	0.9982	0.88	0.1937	0.9960	0.90	0.2573	0.9939	1.04	0.6515	0.9951	1.30	0.0144	0.9411	1.08	0.4217	0.8037	1.11	0.2804	0.5337	1.00	0.9578	0.9961	1.04	0.6507	0.9881
Ethylmalonyl-CoA decarboxylase	ECHD1_HUMAN	Q9NTX5	1.25	0.0066	0.2487	1.01	0.8844	0.9982	0.85	0.0949	0.9960	0.94	0.4940	0.9939	1.09	0.3026	0.9951	1.08	0.4667	0.9856	0.87	0.1069	0.6824	1.06	0.5496	0.7352	1.00	0.9984	0.9990	1.10	0.2330	0.9607
Eukaryotic initiation factor 4A-II	IF4A2_HUMAN	Q14240	1.05	0.3303	0.5968	1.01	0.7905	0.9982	1.03	0.6880	0.9960	0.97	0.5997	0.9939	0.98	0.6885	0.9951	0.99	0.8886	0.9959	1.01	0.8397	0.9537	1.07	0.2928	0.5464	0.91	0.0775	0.9889	0.97	0.6083	0.9881
Eukaryotic initiation factor 4A-III	IF4A3_HUMAN	P38919	0.97	0.4540	0.6965	1.06	0.1646	0.9982	1.11	0.0354	0.9960	1.03	0.4683	0.9939	0.97	0.4675	0.9951	0.91	0.0893	0.9856	1.02	0.6554	0.8915	1.01	0.8870	0.9377	1.06	0.2330	0.9889	0.96	0.3016	0.9607
Eukaryotic translation initiation factor 1b	EIF1B_HUMAN	O60739	1.06	0.5013	0.7246	0.89	0.1425	0.9982	0.92	0.3673	0.9960	1.00	0.9910	0.9981	1.06	0.4982	0.9951	1.16	0.1573	0.9856	0.94	0.4860	0.8365	1.32	0.0023	0.0880	1.10	0.2530	0.9889	1.06	0.4756	0.9617
Eukaryotic translation initiation factor 3 subunit A	EIF3A_HUMAN	Q14152	1.04	0.4049	0.6612	1.04	0.4374	0.9982	1.03	0.5662	0.9960	1.12	0.0129	0.9874	1.12	0.0253	0.9951	0.97	0.5842	0.9856	0.98	0.7296	0.9214	0.95	0.3390	0.5744	1.05	0.3724	0.9889	1.10	0.0457	0.9607
Eukaryotic translation initiation factor 3 subunit B	EIF3B_HUMAN	P55884	1.06	0.3323	0.5968	1.12	0.0686	0.9982	1.12	0.1204	0.9960	1.05	0.4560	0.9939	1.00	0.9698	0.9951	0.92	0.2626	0.9856	1.03	0.6190	0.8770	0.97	0.6859	0.8240	1.01	0.8843	0.9889	0.99	0.9350	0.9938
Eukaryotic translation initiation factor 3 subunit C	EIF3C_HUMAN	Q99613	1.03	0.5901	0.7870	1.04	0.3616	0.9982	1.00	0.9273	0.9960	1.12	0.0132	0.9874	1.03	0.5580	0.9951	1.03	0.6437	0.9856	0.98	0.7544	0.9282	1.03	0.5085	0.7043	1.04	0.4216	0.9889	1.04	0.4127	0.9607
Eukaryotic translation initiation factor 3 subunit F	EIF3F_HUMAN	O00303	0.98	0.7935	0.9011	1.06	0.3352	0.9982	1.18	0.0179	0.9960	1.09	0.1583	0.9874	0.98	0.7129	0.9951	0.85	0.0307	0.9411	1.04	0.5479	0.8600	0.98	0.8209	0.9052	0.91	0.1109	0.9889	0.97	0.6366	0.9881
Eukaryotic translation initiation factor 3 subunit H	EIF3H_HUMAN	O15372	1.00	0.9582	0.9734	1.01	0.8812	0.9982	1.09	0.1568	0.9960	1.02	0.7020	0.9939	0.98	0.6763	0.9951	0.89	0.0726	0.9641	0.92	0.1317	0.6871	1.02	0.6947	0.8265	0.99	0.8034	0.9889	0.99	0.8798	0.9938
Eukaryotic translation initiation factor 3 subunit I	EIF3I_HUMAN	Q13347	1.07	0.4576	0.6991	0.99	0.8982	0.9982	1.04	0.6572	0.9960	1.09	0.2937	0.9939	1.14	0.1348	0.9951	0.98	0.8278	0.9959	0.90	0.2542	0.7187	0.98	0.7996	0.8912	1.07	0.4107	0.9889	1.07	0.4145	0.9607
Eukaryotic translation initiation factor 3 subunit K	EIF3K_HUMAN	Q9UBQ5	0.97	0.4687	0.7086	0.99	0.8927	0.9982	1.03	0.5420	0.9960	1.00	0.9409	0.9939	0.94	0.1255	0.9951	0.96	0.4359	0.9856	0.94	0.1882	0.7149	1.02	0.6906	0.8252	0.95	0.2646	0.9889	0.98	0.5705	0.9881
Eukaryotic translation initiation factor 3 subunit L	EIF3L_HUMAN	Q9Y262	1.08	0.3225	0.5917	1.11	0.1722	0.9982	1.11	0.2708	0.9960	1.05	0.5502	0.9939	1.01	0.9366	0.9951	0.88	0.1757	0.9856	0.98	0.7727	0.9317	1.04	0.6253	0.7890	0.98	0.8501	0.9889	0.97	0.6519	0.9881
Eukaryotic translation initiation factor 4 gamma 1	IF4G1_HUMAN	Q04637	1.12	0.1275	0.3938	1.03	0.6990	0.9982	0.99	0.9520	0.9960	1.08	0.3310	0.9939	1.18	0.0332	0.9951	0.96	0.6492	0.9856	1.00	0.9981	0.9992	0.92	0.3253	0.5681	0.98	0.8176	0.9889	1.09	0.2490	0.9607
Eukaryotic translation initiation factor 4B	IF4B_HUMAN	P23588	1.09	0.2765	0.5477	0.97	0.6737	0.9982	1.08	0.3777	0.9960	0.96	0.6245	0.9939	1.07	0.4011	0.9951	1.03	0.7843	0.9897	0.97	0.7361	0.9218	1.10	0.2804	0.5337	0.95	0.5656	0.9889	1.00	0.9712	0.9992
Eukaryotic translation initiation factor 4H	IF4H_HUMAN	Q15056	1.06	0.4570	0.6991	0.94	0.4163	0.9982	0.97	0.7356	0.9960	1.01	0.8831	0.9939	1.02	0.7861	0.9951	1.11	0.3032	0.9856	0.94	0.4745	0.8329	1.08	0.3782	0.6095	0.96	0.6262	0.9889	0.98	0.8235	0.9881
Eukaryotic translation initiation factor 5A-1	IF5A1_HUMAN	P63241	1.05	0.4909	0.7219	0.96	0.5103	0.9982	1.07	0.3415	0.9960	0.93	0.2884	0.9939	0.93	0.3081	0.9951	1.00	0.9566	0.9967	0.94	0.3472	0.7593	1.16	0.0363	0.2067	0.94	0.3436	0.9889	0.89	0.0807	0.9607
Evolutionarily conserved signaling intermediate in Toll pathway, mitochc	ECSIT_HUMAN	Q9BQ95	1.22	0.0612	0.3033	1.07	0.5059	0.9982	0.94	0.6322	0.9960	0.94	0.5669	0.9939	1.14	0.2245	0.9951	1.05	0.6924	0.9856	0.81	0.0500	0.6824	1.09	0.4872	0.6890	0.98	0.8713	0.9889	1.33	0.0055	0.9304
Extended synaptotagmin-1	ESYT1_HUMAN	Q9BSJ8	0.86	0.0303	0.2873	1.04	0.5396	0.9982	1.10	0.2591	0.9960	0.94	0.4110	0.9939	0.91	0.1823	0.9951	0.92	0.3713	0.9856	1.11	0.1479	0.6871	0.89	0.1283	0.3638	0.96	0.6088	0.9889	0.92	0.2683	0.9607
Extracellular superoxide dismutase [Cu-Zn]	SODE_HUMAN	P08294	0.76	0.0175	0.2873	0.86	0.2125	0.9982	1.11	0.4214	0.9960	1.13	0.3040	0.9939	0.90	0.3803	0.9951	0.93	0.6090	0.9856	1.16	0.2397	0.7187	0.90	0.4035	0.6299	0.96	0.7137	0.9889	0.92	0.4471	0.9607
Ezrin	EZRI_HUMAN	P15311	0.95	0.3900	0.6473	0.96	0.4653	0.9982	1.04	0.4948	0.9960	0.91	0.0901	0.9874	0.88	0.0206	0.9951	0.98	0.7540	0.9857	0.98	0.6660	0.8929	0.97	0.6679	0.8105	0.97	0.5702	0.9889	0.93	0.2129	0.9607
F-actin-capping protein subunit alpha-1	CAZA1_HUMAN	P52907	0.84	0.1056	0.3654	0.92	0.4349	0.9982	1.01	0.9555	0.9960	0.97	0.7837	0.9939	1.08	0.4841	0.9951	1.01	0.9439	0.9959	1.22	0.0744	0.6824	0.83	0.1219	0.3555	0.94	0.5594	0.9889	1.06	0.5780	0.9881
F-actin-capping protein subunit alpha-2	CAZA2_HUMAN	P47755	0.88	0.0987	0.3566	1.08	0.3326	0.9982	1.10	0.3130	0.9960	0.99	0.9230	0.9939	0.96	0.6057	0.9951	0.88	0.2177	0.9856	1.10	0.2690	0.7187	0.81	0.0175	0.1636	0.89	0.1631	0.9889	0.97	0.6831	0.9881
F-actin-capping protein subunit beta	CAPZB_HUMAN	P47756	0.89	0.1470	0.4117	1.03	0.7512	0.9982	1.03	0.7356	0.9960	1.02	0.8189	0.9939	1.01	0.8889	0.9951	0.97	0.7230	0.9857	1.17	0.0600	0.6824	0.86	0.0785	0.2861	0.95	0.5116	0.9889	1.00	0.9571	0.9941
Far upstream element-binding protein 1	FUBP1_HUMAN	Q96AE4	1.09	0.1563	0.4253	1.04	0.5084	0.9982	0.96	0.5559	0.9960	0.97	0.6469	0.9939	0.98	0.6985	0.9951	1.09	0.2835	0.9856	0.92	0.2137	0.7171	1.09	0.2096	0.4758	0.98	0.7437	0.9889	0.95	0.4386	0.9607
Far upstream element-binding protein 2	FUBP2_HUMAN	Q92945	0.97	0.5395	0.7500	0.96	0.4488	0.9982	0.96	0.4959	0.9960	0.96	0.4520	0.9939	0.99	0.8672	0.9951	1.07	0.3103	0.9856	1.00	0.9440	0.9903	1.04	0.5296	0.7227	1.00	0.9791	0.9976	0.96	0.4669	0.9607
Farnesyl pyrophosphate synthase	FPPS_HUMAN	P14324	1.23	0.0237	0.2873	1.16	0.0990	0.9982	0.91	0.4051	0.9960	0.98	0.8646	0.9939	1.07	0.4846	0.9951	1.12	0.3048	0.9856	0.96	0.6762	0.8962	1.08	0.4524	0.6671	0.88	0.1882	0.9889	1.01	0.8796	0.9938
Fascin	FSCN1_HUMAN	Q16658	0.95	0.5664	0.7665	0.94	0.4745	0.9982	0.97	0.7859	0.9960	0.94	0.5100	0.9939	0.95	0.5493	0.9951	1.12	0.2952	0.9856	1.07	0.4827	0.8361	0.98	0.8763	0.9320	0.94	0.4801	0.9889	0.85	0.0578	0.9607
Fatty acid-binding protein 5	FABP5_HUMAN	Q01469	1.16	0.1150	0.3763	1.09	0.3744	0.9982	1.02	0.8580	0.9960	0.85	0.0851	0.9874	1.02	0.8272	0.9951	1.08	0.4997	0.9856	0.89	0.2634	0.7187	1.05	0.6493	0.7998	0.97	0.7341	0.9889	0.97	0.7209	0.9881
Fatty acid-binding protein, adipocyte	FABP4_HUMAN	P15090	1.05	0.6685	0.8389	0.89	0.3661	0.9982	0.94	0.6490	0.9960	0.83	0.1216	0.9874	0.97	0.8334	0.9951	1.16	0.3460	0.9856	1.08	0.5783	0.8632	1.03	0.8264	0.9067	0.81	0.0943	0.9889	0.88	0.2908	0.9607
Fatty acid-binding protein, heart	FABPH_HUMAN	P05413	1.24	0.0849	0.3346	0.99	0.9553	0.9982	0.91	0.5430	0.9960	0.94	0.6471	0.9939	1.02	0.8796	0.9951	1.20	0.2409	0.9856	0.79	0.0766	0.6824	1.37	0.0269	0.1844	0.98	0.8678	0.9889	1.05	0.7032	0.9881
Fermitin family homolog 2	FERM2_HUMAN	Q96AC1	0.94	0.1875	0.4611	1.00	0.9583	0.9982	1.08	0.1705	0.9960	1.02	0.7141	0.9939	0.93	0.1267	0.9951	0.91	0.1229	0.9856	0.98	0.6786	0.8977	0.98	0.6666	0.8098	1.01	0.8312	0.9889	0.93	0.1500	0.9607
Ferredoxin-2, mitochondrial	FDX2_HUMAN	Q6P4F2	1.09	0.2884	0.5627	1.18	0.0397	0.9982	0.85	0.0900	0.9960	1.06	0.5010	0.9939	0.94	0.4471	0.9951	1.22	0.0525	0.9621	0.96	0.6152	0.8745	0.92	0.3956	0.6232	1.00	0.9961	0.9990	0.92	0.2812	0.9607
Ferritin heavy chain	FRIH_HUMAN	P02794	1.10	0.3668	0.6279	0.94	0.5546	0.9982	0.94	0.6545	0.9960	0.95	0.6477	0.9939	1.03	0.8180	0.9951	1.11	0.4507	0.9856	0.95	0.6662	0.8929	0.91	0.4388	0.6588	0.80	0.0413	0.9889	0.91	0.3768	0.9607

Ferritin light chain	FRIL_HUMAN	P02792	0.87	0.4995	0.7246	1.03	0.8777	0.9982	0.99	0.9603	0.9960	1.01	0.9760	0.9961	0.91	0.6573	0.9951	1.01	0.9720	0.9967	0.98	0.9424	0.9903	0.72	0.1617	0.4114	0.72	0.1228	0.9889	0.77	0.2189	0.9607
Ferrochelatase, mitochondrial	HEMH_HUMAN	P22830	0.98	0.8003	0.9037	1.06	0.3149	0.9982	0.98	0.8284	0.9960	1.06	0.3701	0.9939	1.01	0.9069	0.9951	0.96	0.6321	0.9856	0.98	0.7029	0.9105	0.94	0.3667	0.5972	1.08	0.2318	0.9889	1.05	0.4179	0.9607
Fibrillin-1	FBN1_HUMAN	P35555	1.06	0.5686	0.7685	1.05	0.6079	0.9982	1.15	0.2344	0.9960	0.83	0.0588	0.9874	1.04	0.7317	0.9951	0.98	0.8538	0.9959	0.93	0.5232	0.8558	0.94	0.5917	0.7646	1.19	0.0882	0.9889	1.13	0.2416	0.9607
Fibrinogen alpha chain	FIBA_HUMAN	P02671	0.77	0.0766	0.3266	1.05	0.7580	0.9982	1.01	0.9349	0.9960	1.12	0.4282	0.9939	0.98	0.9030	0.9951	1.00	0.9898	0.9992	1.33	0.0679	0.6824	0.64	0.0054	0.1317	1.15	0.3681	0.9889	0.94	0.6929	0.9881
Fibrinogen beta chain	FIBB_HUMAN	P02675	0.73	0.0461	0.2898	1.05	0.7413	0.9982	1.06	0.7367	0.9960	1.09	0.6006	0.9939	0.92	0.6023	0.9951	0.98	0.9201	0.9959	1.32	0.0994	0.6824	0.62	0.0072	0.1331	1.13	0.4655	0.9889	0.89	0.4793	0.9642
Fibrinogen gamma chain	FIBG_HUMAN	P02679	0.73	0.0439	0.2873	1.07	0.6844	0.9982	1.09	0.6198	0.9960	1.08	0.6201	0.9939	0.91	0.5685	0.9951	0.93	0.7170	0.9857	1.30	0.1132	0.6824	0.64	0.0082	0.1361	1.11	0.5293	0.9889	0.88	0.3988	0.9607
Fibromodulin	FMOD_HUMAN	Q06828	0.78	0.3437	0.6081	0.93	0.7723	0.9982	0.63	0.1295	0.9960	1.02	0.9333	0.9939	0.95	0.8420	0.9951	1.25	0.4874	0.9856	1.29	0.3585	0.7644	0.44	0.0034	0.1118	0.92	0.7552	0.9889	0.98	0.9523	0.9938
Fibronectin	FINC_HUMAN	P02751	0.80	0.0737	0.3253	1.07	0.5980	0.9982	1.05	0.7426	0.9960	0.98	0.8814	0.9939	0.87	0.2958	0.9951	0.93	0.6627	0.9856	1.08	0.5676	0.8601	0.73	0.0301	0.1916	1.05	0.7397	0.9889	0.87	0.2977	0.9607
Fibulin-1	FBLN1_HUMAN	P23142	0.80	0.2845	0.5580	1.19	0.4021	0.9982	0.91	0.6794	0.9960	1.09	0.6753	0.9939	1.01	0.9730	0.9951	0.98	0.9519	0.9967	1.41	0.1116	0.6824	0.47	0.0005	0.0621	0.95	0.8000	0.9889	0.92	0.6795	0.9881
Fibulin-2	FBLN2_HUMAN	P98095	0.83	0.2047	0.4823	0.93	0.6375	0.9982	0.96	0.7987	0.9960	0.98	0.9155	0.9939	0.95	0.7129	0.9951	1.08	0.6672	0.9856	1.16	0.3350	0.7458	0.77	0.1061	0.3316	1.05	0.7295	0.9889	0.90	0.4685	0.9607
Fibulin-5	FBLN5_HUMAN	Q9UBX5	0.78	0.1409	0.4031	1.24	0.2192	0.9982	1.06	0.7728	0.9960	1.00	0.9954	0.9986	0.79	0.1965	0.9951	1.02	0.9262	0.9959	1.10	0.6134	0.8738	0.66	0.0268	0.1844	1.04	0.8307	0.9889	0.99	0.9544	0.9938
Filamin-A	FLNA_HUMAN	P21333	0.76	0.0193	0.2873	1.04	0.7601	0.9982	0.96	0.7464	0.9960	0.87	0.2355	0.9939	0.83	0.1255	0.9951	1.14	0.3784	0.9856	1.06	0.6515	0.8906	0.78	0.0518	0.2413	0.92	0.5169	0.9889	0.93	0.5299	0.9881
Filamin-B	FLNB_HUMAN	O75369	0.94	0.3005	0.5713	0.98	0.7277	0.9982	1.01	0.9353	0.9960	1.03	0.6155	0.9939	1.03	0.6396	0.9951	1.04	0.6492	0.9856	1.07	0.3297	0.7411	0.92	0.2126	0.4786	0.98	0.7185	0.9889	0.96	0.5282	0.9881
Filamin-C	FLNC_HUMAN	Q14315	0.91	0.1797	0.4557	0.88	0.0554	0.9982	1.14	0.1126	0.9960	1.10	0.1521	0.9874	0.96	0.5719	0.9951	0.86	0.0692	0.9641	0.97	0.7072	0.9121	1.25	0.0039	0.1242	0.99	0.9081	0.9889	0.95	0.4215	0.9607
Flavin reductase (NADPH)	BLVRB_HUMAN	P30043	1.27	0.0117	0.2608	1.02	0.8572	0.9982	0.98	0.8481	0.9960	0.94	0.4969	0.9939	1.16	0.1326	0.9951	1.10	0.4042	0.9856	0.93	0.5001	0.8476	1.05	0.6545	0.8025	0.92	0.4065	0.9889	1.11	0.2829	0.9607
Flotillin-1	FLOT1_HUMAN	O75955	1.06	0.6076	0.7989	1.17	0.1528	0.9982	0.98	0.8413	0.9960	0.97	0.7838	0.9939	0.95	0.6603	0.9951	1.03	0.8351	0.9959	0.99	0.9546	0.9923	0.93	0.5717	0.7504	1.01	0.9636	0.9961	1.09	0.4387	0.9607
Four and a half LIM domains protein 1	FHL1_HUMAN	Q13642	0.84	0.0367	0.2873	0.90	0.2028	0.9982	1.13	0.2011	0.9960	1.02	0.8039	0.9939	0.88	0.1403	0.9951	0.89	0.2642	0.9856	0.95	0.5448	0.8590	1.13	0.1949	0.4557	1.12	0.1744	0.9889	0.99	0.9258	0.9938
Four and a half LIM domains protein 2	FHL2_HUMAN	Q14192	1.04	0.6840	0.8479	1.01	0.9518	0.9982	1.02	0.9044	0.9960	0.97	0.7478	0.9939	1.27	0.0319	0.9951	0.95	0.7163	0.9857	0.90	0.3485	0.7593	0.97	0.7857	0.8852	1.13	0.2620	0.9889	1.34	0.0046	0.9304
Frataxin, mitochondrial	FRDA_HUMAN	Q16595	1.14	0.0976	0.3563	1.08	0.3577	0.9982	0.95	0.6119	0.9960	0.91	0.2591	0.9939	0.97	0.7519	0.9951	1.09	0.3806	0.9856	0.92	0.3440	0.7576	1.16	0.1066	0.3325	1.09	0.2864	0.9889	0.97	0.6673	0.9881
Fructosamine-3-kinase	FN3K_HUMAN	Q9H479	1.18	0.0079	0.2487	1.08	0.2417	0.9982	0.95	0.5145	0.9960	1.13	0.0559	0.9874	1.10	0.1428	0.9951	1.02	0.7840	0.9897	0.97	0.6965	0.9074	1.01	0.8837	0.9373	1.03	0.6961	0.9889	1.12	0.0678	0.9607
Fructose-bisphosphate aldolase A	ALDOA_HUMAN	P04075	1.04	0.5666	0.7665	0.99	0.8405	0.9982	1.01	0.8888	0.9960	0.96	0.5312	0.9939	0.90	0.1036	0.9951	1.05	0.5035	0.9856	1.00	0.9423	0.9903	1.11	0.1424	0.3873	1.01	0.8758	0.9889	0.96	0.4670	0.9607
Fructose-bisphosphate aldolase C	ALDOC_HUMAN	P09972	1.02	0.7906	0.8986	1.00	0.9761	0.9984	0.98	0.8142	0.9960	0.99	0.9452	0.9939	0.92	0.2781	0.9951	1.11	0.2728	0.9856	0.98	0.8218	0.9481	1.17	0.0740	0.2796	1.08	0.3194	0.9889	1.02	0.7603	0.9881
Fumarate hydratase, mitochondrial	FUMH_HUMAN	P07954	1.15	0.1001	0.3575	1.01	0.9079	0.9982	0.89	0.2730	0.9960	0.94	0.4644	0.9939	0.96	0.6739	0.9951	1.13	0.2781	0.9856	0.86	0.1073	0.6824	1.17	0.1057	0.3312	1.06	0.5230	0.9889	1.02	0.8420	0.9881
Fumarylacetoacetate hydrolase domain-containing protein 2A	FAH2A_HUMAN	Q96GK7	1.08	0.2724	0.5423	1.07	0.3275	0.9982	1.00	0.9850	0.9960	1.02	0.8024	0.9939	0.99	0.8422	0.9951	0.97	0.7469	0.9857	0.88	0.0747	0.6824	0.97	0.6442	0.7988	0.98	0.7394	0.9889	1.01	0.8984	0.9938
FUN14 domain-containing protein 2	FUND2_HUMAN	Q9BWH2	1.12	0.0426	0.2873	1.04	0.5350	0.9982	0.97	0.6914	0.9960	1.02	0.7571	0.9939	1.01	0.8390	0.9951	0.97	0.6297	0.9856	0.90	0.0821	0.6824	1.02	0.7243	0.8448	0.91	0.1059	0.9889	1.05	0.4293	0.9607
G-rich sequence factor 1	GRSF1_HUMAN	Q12849	1.08	0.1816	0.4568	1.02	0.7609	0.9982	1.01	0.9036	0.9960	1.03	0.5893	0.9939	1.00	0.9414	0.9951	1.01	0.9168	0.9959	0.95	0.4481	0.8181	1.01	0.9299	0.9594	1.08	0.1900	0.9889	1.02	0.7851	0.9881
Galectin-1	LEG1_HUMAN	P09382	1.01	0.8265	0.9186	0.91	0.0804	0.9982	1.00	0.9818	0.9960	1.07	0.1923	0.9939	0.99	0.8914	0.9951	0.96	0.5800	0.9856	1.04	0.4954	0.8449	1.07	0.2801	0.5337	0.93	0.2256	0.9889	0.93	0.1645	0.9607
Galectin-3	LEG3_HUMAN	P17931	0.95	0.4676	0.7076	1.09	0.1766	0.9982	0.89	0.1417	0.9960	1.13	0.0568	0.9874	1.04	0.5706	0.9951	1.09	0.3053	0.9856	1.16	0.0350	0.6824	0.78	0.0004	0.0621	0.95	0.4919	0.9889	1.05	0.4653	0.9607
Galectin-3-binding protein	LG3BP_HUMAN	Q08380	1.04	0.6303	0.8170	1.12	0.1195	0.9982	1.10	0.2543	0.9960	1.14	0.0575	0.9874	1.07	0.3362	0.9951	0.89	0.1708	0.9856	0.99	0.9399	0.9903	0.94	0.4429	0.6607	0.97	0.6906	0.9889	0.97	0.6572	0.9881
Gamma-enolase	ENOG_HUMAN	P09104	0.91	0.3793	0.6371	0.91	0.3559	0.9982	1.09	0.4984	0.9960	0.91	0.3501	0.9939	0.82	0.0657	0.9951	0.90	0.4394	0.9856	0.76	0.0146	0.6779	1.24	0.0650	0.2631	1.00	0.9976	0.9990	0.90	0.3011	0.9607
Gamma-sarcoglycan	SGCG_HUMAN	Q13326	0.90	0.1106	0.3718	1.00	0.9712	0.9982	1.07	0.3805	0.9960	1.03	0.6974	0.9939	0.99	0.8370	0.9951	0.89	0.1624	0.9856	1.00	0.9935	0.9992	0.88	0.1078	0.3341	0.94	0.3801	0.9889	0.98	0.7785	0.9881
Gamma-soluble NSF attachment protein	SNAG_HUMAN	Q99747	0.88	0.0471	0.2898	0.95	0.4243	0.9982	1.12	0.1354	0.9960	0.96	0.5789	0.9939	0.94	0.3402	0.9951	0.89	0.1546	0.9856	1.07	0.3563	0.7644	1.08	0.2790	0.5337	0.98	0.8125	0.9889	0.95	0.3892	0.9607
GDH/6PGL endoplasmic bifunctional protein	G6PE_HUMAN	O95479	0.99	0.8686	0.9356	1.03	0.6596	0.9982	0.95	0.4837	0.9960	1.05	0.4761	0.9939	0.92	0.2597	0.9951	1.06	0.4713	0.9856	1.00	0.9629	0.9924	1.05	0.5110	0.7066	1.08	0.2416	0.9889	0.94	0.3357	0.9607
GDP-fucose protein O-fucosyltransferase 1	OFUT1_HUMAN	Q9H488	1.15	0.0279	0.2873	1.13	0.0565	0.9982	1.13	0.1154	0.9960	1.02	0.7433	0.9939	1.05	0.4645	0.9951	0.91	0.2289	0.9856	0.93	0.2710	0.7196	0.98	0.7588	0.8675	0.99	0.9274	0.9889	1.09	0.1940	0.9607
Gelsolin	GELS_HUMAN	P06396	0.85	0.0266	0.2873	0.96	0.5982	0.9982	1.00	0.9946	0.9978	0.99	0.9295	0.9939	0.94	0.4348	0.9951	1.00	0.9634	0.9967	1.09	0.2635	0.7187	0.90	0.2355	0.4981	0.98	0.7667	0.9889	0.98	0.7589	0.9881
General vesicular transport factor p115	USO1_HUMAN	O60763	1.09	0.1826	0.4577	1.04	0.5577	0.9982	1.04	0.5858	0.9960	0.99	0.8958	0.9939	0.98	0.7825	0.9951	1.02	0.7894	0.9903	0.97	0.6332	0.8882	1.17	0.0300	0.1912	1.00	0.9695	0.9971	0.98	0.8068	0.9881
Glucosamine-6-phosphate isomerase 1	GNPI1_HUMAN	P46926	1.17	0.0746	0.3261	1.13	0.1473	0.9982	0.97	0.7999	0.9960	0.92	0.3397	0.9939	0.96	0.6384	0.9951	1.05	0.6318	0.9856	1.00	0.9667	0.9935	1.09	0.3690	0.5979	0.92	0.3248	0.9889	0.91	0.2685	0.9607
Glucose-6-phosphate isomerase	G6PI_HUMAN	P06744	1.17	0.0943	0.3537	1.00	0.9947	0.9985	0.89	0.3002	0.9960	0.89	0.2085	0.9939	1.01	0.9391	0.9951	1.24	0.0581	0.9641	0.89	0.2630	0.7187	1.18	0.1113	0.3394	0.93	0.4826	0.9889	1.03	0.7659	0.9881
Glucosidase 2 subunit beta	GLU2B_HUMAN	P14314	0.97	0.4924	0.7220	0.95	0.2731	0.9982	1.04	0.4985	0.9960	1.03	0.4952	0.9939	0.99	0.8145	0.9951	0.97	0.6173	0.9856	1.07	0.1397	0.6871	0.99	0.7730	0.8789	0.98	0.6022	0.9889	0.96	0.3704	0.9607
Glutamate dehydrogenase 1, mitochondrial	DHE3_HUMAN	P00367	1.06	0.2322	0.5066	0.95	0.3225	0.9982	1.01	0.8665	0.9960	0.96	0.4760	0.9939	0.96	0.4303	0.9951	1.02	0.7647	0.9857	0.93	0.2089	0.7149	1.10	0.0906	0.3039	0.99	0.7919	0.9889	0.94	0.2485	0.9607
Glutaminase kidney isoform, mitochondrial	GLSK_HUMAN	O94925	1.13	0.1196	0.3810	1.09	0.3019	0.9982	0.99	0.9179	0.9960	1.04	0.6677	0.9939	1.11	0.2292	0.9951	0.96	0.6804	0.9856	1.22	0.0185	0.6779	0.84	0.0583	0.2503	0.97	0.6921	0.9889	1.04	0.6623	0.9881
Glutamine amidotransferase-like class 1 domain-containing protein 3A, m	GAL3A_HUMAN	P0DPI2	1.29	0.0102	0.2530	1.07	0.4745	0.9982	0.82	0.0797	0.9960	0.92	0.4090	0.9939	1.01	0.9290	0.9951	1.25	0.0636	0.9641	0.93	0.4781	0.8352	1.13	0.2713	0.5288	1.01	0.9240	0.9889	1.06	0.5742	0.9881
Glutaredoxin-1	GLRX1_HUMAN	P35754	1.10	0.2031	0.4813	0.96	0.5782	0.9982	0.94	0.5320	0.9960	0.99	0.9223	0.9939	0.99	0.9505	0.9951	1.12	0.2222	0.9856	0.92	0.3220	0.7400	1.35	0.0004	0.0621	1.07	0.3688	0.9889	0.99	0.9246	0.9938
Glutaredoxin-related protein 5, mitochondrial	GLRX5_HUMAN	Q86SX6	1.00	0.9747	0.9855	1.04	0.6538	0.9982	0.99	0.9261	0.9960	0.93	0.3836	0.9939	0.90	0.2425	0.9951	1.15	0.1813	0.9856	0.92	0.3402	0.7512	1.25	0.0170	0.1636	1.05	0.5779	0.9889	0.92	0.3087	0.9607
Glutaryl-CoA dehydrogenase, mitochondrial	GCDH_HUMAN	Q92947	1.16	0.0309	0.2873	0.97	0.6690	0.9982	0.96	0.6327	0.9960	0.97	0.6191	0.9939	1.04	0.5553	0.9951	1.04	0.6170	0.9856	0.94	0.4077	0.7885	1.07	0.3909	0.6204	1.10	0.1656	0.9889	1.10	0.1527	0.9607
Glutathione hydrolase 5 proenzyme	GGT5_HUMAN	P36269	0.69	0.0175	0.2873	1.08	0.6292	0.9982	0.84	0.3293	0.9960	1.10	0.5351	0.9939	0.96	0.7864	0.9951	1.19	0.3772	0.9856	1.37	0.0540	0.6824	0.64	0.0088	0.1369	0.97	0.8670	0.9889	0.97	0.8548	0.9881
Glutathione peroxidase 1	GPX1_HUMAN	P07203	1.02	0.8027	0.9057	0.99	0.9214	0.9982	0.97	0.7529	0.9960	1.02	0.7830	0.9939	1.09	0.3127	0.9951	1.04	0.6880	0.9856	1.03	0.7617	0.9282	0.92	0.3943	0.6230	1.02	0.8107	0.9889	1.03	0.7342	0.9881
Glutathione peroxidase 3	GPX3_HUMAN	P22352	1.12	0.1157	0.3778	1.04	0.6074	0.9982	0.97	0.7696	0.9960	1.02	0.7829	0.9939	1.03	0.6895	0.9951	0.94	0.5029	0.9856	0.92	0.2693	0.7187	0.91	0.2413	0.5050	1.02	0.8400	0.9889	1.11	0.1759	0.9607
Glutathione reductase, mitochondrial	GSHR_HUMAN	P00390	1.14	0.0191	0.2873	1.04	0.5147	0.9982	0.98	0.7552	0.9960	0.95	0.3374	0.9939	1.02	0.7648	0.9951	1.04	0.5987	0.9856	0.99	0.9187	0.9848	0.99	0.9209	0.9569	1.02	0.7224	0.9889	1.00	0.9399	0.9938
Glutathione S-transferase kappa 1	GSTK1_HUMAN	Q9Y2Q3	1.12	0.0724	0.3231	1.08	0.1996	0.9982	0.94	0.3529	0.9960	0.96	0.4990	0.9939	0.98	0.7355	0.9951	1.05	0.5616	0.9856	0.92	0.1892	0.7149	1.04	0.5486	0.7350	1.01	0.8791	0.9889	1.00	0.9538	0.9938
Glutathione S-transferase Mu 2	GSTM2_HUMAN	P28161	1.20	0.0680	0.3121	1.10	0.3374	0.9982	0.87	0.2322	0.9960	0.91	0.3488	0.9939	1.03	0.7726	0.9951	1.30	0.0309	0.9411	0.90	0.3021	0.7361	1.33	0.0083	0.1361	1.13	0.2289	0.9889	1.06	0.5681	0.9881
Glutathione S-transferase Mu 3	GSTM3_HUMAN	P21266	1.15	0.2107	0.4843	1.11	0.3578	0.9982	0.95	0.7029	0.9960	0.97	0.7945	0.9939	1.02	0.8376	0.9951	1.14	0.3431	0.9856	0.82	0.0811	0.6824	1.27	0.0517	0.2413	0.96	0.7114	0.9889	1.05	0.6873	0.9881
Glutathione S-transferase omega-1	GSTO1_HUMAN	P78417	1.16	0.1325	0.3961	0.95	0.6302	0.9982	0.99	0.9112	0.9960	0.82	0.0527	0.9874	0.95	0.6038	0.9951	1.08	0.5449	0.9856	0.79	0.0225	0.6824	1.34	0.0079	0.1361	0.97	0.7346	0.9889	0.97	0.8019	0.9881
Glutathione S-transferase P	GSTP1_HUMAN	P09211	1.10	0.1806	0.4557	1.05	0.5344	0.9982	0.95	0.5292	0.9960	0.96	0.5788	0.9939	0.95	0.5264	0.9951	1.16	0.0964	0.9856	0.88	0.0818	0.6824	1.10	0.2198	0.4875	0.94	0.4036	0.9889	0.93	0.3478	0.9607
Glutathione synthetase	GSHB_HUMAN	P48637	1.16	0.0672	0.3105	1.05	0.5357	0.9982	0.89	0.2275	0.9960	1.01	0.9445	0.9939	1.10	0.2581	0.9951	1.23	0.0433	0.9411	0.96	0.6671	0.8929	1.04	0.7137	0.8360	0.91	0.2482	0.9889	1.05	0.5479	0.9881
Glyceraldehyde-3-phosphate dehydrogenase	G3P_HUMAN	P04406	1.11	0.1433	0.4056	1.01	0.8902	0.9982	0.93	0.4251	0.9960	0.93	0.3403	0.9939	0.97	0.7031	0.9951	1.19	0.0523	0.9621	1.04	0.5770	0.8630	1.13	0.1334	0.3740	1.04	0.5887	0.9889	1.01	0.9301	0.9938
Glycerol-3-phosphate dehydrogenase 1-like protein	GPD1L_HUMAN	Q8N335	1.19	0.0259	0.2873	1.15	0.0844	0.9982	0.91	0.3403	0.9960	0.92	0.2950	0.9939	1.01	0.8675	0.9951	1.14	0.1909	0.9856	0.97	0.7003	0.9088	1.03	0.7740	0.8791	1.09	0.3264	0.9889	1.11	0.2116	0.9607
Glycerol-3-phosphate phosphatase	PGP_HUMAN	A6NDG6	1.10	0.1740	0.4492	0.97	0.6541	0.9982	0.98	0.7901	0.9960	0.99	0.8985	0.9939	1.05	0.4968	0.9951	1.01	0.9128	0.9959	0.96	0.5822	0.8635	1.22	0.0089	0.1369	1.06	0.4192	0.9889	1.10	0.1616	0.9607
Glycine amidinotransferase, mitochondrial	GATM_HUMAN	P50440	1.07	0.6636	0.8373	0.78	0.0823	0.9982	1.13	0.4813	0.9960	1.00	0.9863	0.9981	1.03	0.8421	0.9951	0.78	0.1742	0.9856	0.93	0.6572	0.8915	1.14	0.4369	0.6566	0.94	0.6680	0.9889	0.88	0.3721	0.9607
Glycine cleavage system H protein, mitochondrial	GCSH_HUMAN	P23434	1.26	0.0282	0.2873	1.11	0.3453	0.9982	0.89	0.3498	0.9960	1.00	0.9835	0.9969	1.06	0.6072	0.9951	1.17	0.2287	0.9856	0.90	0.3711	0.7673	1.14	0.2627	0.5247	0.98	0.8637	0.9889	1.05	0.6355	0.9881
Glycine--tRNA ligase	GARS_HUMAN	P41250	1.15	0.2418	0.5165	1.15	0.2531	0.9982	0.82	0.1618	0.9960	1.24	0.0703	0.9874	1.21	0.1330	0.9951	1.27	0.1052	0.9856	1.02	0.8826	0.9740	0.94	0.6593	0.8070	1.18	0.1759	0.9889	1.10	0.4381	0.9607
Glycogen [starch] synthase, muscle	GYS1_HUMAN	P13807	1.14	0.1300	0.3958	0.96	0.6200	0.9982	0.99	0.8918	0.9960	1.01	0.8665	0.9939	1.06	0.5319	0.9951	0.95	0.6383	0.9856	0.91	0.2887	0.7212	1.21	0.0396	0.2161	1.08	0.3958	0.9889	1.13	0.1343	0.9607
Glycogen debranching enzyme	GDE_HUMAN	P35573	1.18	0.0275	0.2873	1.05	0.5168	0.9982	1.02	0.8436	0.9960	1.02	0.8136	0.9939	1.06	0.4894	0.9951	0.97	0.7711	0.9857	0.87	0.0891	0.6824	1.10	0.2597	0.5245	1.04	0.5917	0.9889	1.12	0.1195	0.9607
Glycogen phosphorylase, brain form	PYGB_HUMAN	P11216	1.12	0.1784	0.4545	1.01	0.8853	0.9982	0.96	0.7156	0.9960	1.07	0.4126	0.9939	1.01	0.9052	0.9951	1.05	0.6681	0.9856	0.90	0.2474	0.7187	1.22	0.0357	0.2045	1.03	0.7310	0.9889	1.10	0.2411	0.9607
Glycogen phosphorylase, muscle form	PYGM_HUMAN	P11217	1.22	0.0358	0.2873	1.14	0.1602	0.9982	1.00	0.9890	0.9960	1.05	0.6119	0.9939	1.03	0.7851	0.9951	1.00	0.9960	0.9992	0.97	0.7620	0.9282	1.24	0.0426	0.2201	0.91	0.3410	0.9889	1.03	0.7319	0.9881
Glycogenin-1	GLYG_HUMAN	P46976	1.08	0.3306	0.5968	1.03	0.7116	0.9982	0.97	0.7759	0.9960	0.99	0.8587	0.9939	0.98	0.8253	0.9951	0.95	0.5898	0.9856	0.91	0.2985	0.7319	0.83	0.0461	0.2280	0.97	0.7148	0.9889	1.03	0.7009	0.9881
Glyoxalase domain-containing protein 4	GLOD4_HUMAN	Q9HC38	1.13	0.0831	0.3318	0.99	0.9105	0.9982	1.04	0.6416	0.9960	0.98	0.7530	0.9939	1.06	0.4449	0.9951	1.05	0.5460	0.9856	0.91	0.2226	0.7187	1.01	0.8863	0.9377	0.94	0.4124	0.9889	1.07	0.3305	0.9607
Glyoxylate reductase/hydroxypyruvate reductase	GRHPR_HUMAN	Q9UBQ7	1.17	0.0102	0.2530	1.01	0.9256	0.9982	0.88	0.0774	0.9960	0.91	0.1304	0.9874	1.02	0.8009	0.9951	1.15	0.0678	0.9641	0.96	0.5162	0.8541	1.02	0.7900	0.8865	1.04	0.4942	0.9889	1.06	0.3179	0.9607
Glypican-1	GPC1_HUMAN	P35052	1.08	0.2230	0.4953	0.94	0.3020	0.9982	1.14	0.0840	0.9960	1.10	0.1507	0.9874	1.01	0.8455	0.9951	0.83	0.0164	0.9411	0.92	0.2350	0.7187	1.07	0.3538	0.5899	0.90	0.1249	0.9889	0.98	0.7658	0.9881
Golgi apparatus protein 1	GSLG1_HUMAN	Q92896	0.88	0.0287	0.2873	0.95	0.4138	0.9982	1.06	0.4093	0.9960	1.05	0.4374	0.9939	0.95	0.3949	0.9951	0.95	0.5277	0.9856	1.01	0.8616	0.9637	0.91	0.1703	0.4217	0.95	0.3800	0.9889	0.95	0.3688	0.9607
Golgi integral membrane protein 4	GOLI4_HUMAN	O00461	0.74	0.0037	0.2034	1.06	0.5948	0.9982	1.01	0.9292	0.9960	0.98	0.8489	0.9939	0.87	0.2049	0.9951	0.96	0.7394	0.9857	0.99	0.9637	0.9924	0.81	0.0780	0.2849	1.09	0.4364	0.9889	0.94	0.5637	0.9881
Golgi reassembly-stacking protein 2	GORS2_HUMAN	Q9H8Y8	1.05	0.4705	0.7095	0.94	0.3085	0.9982	0.98	0.8217	0.9960	1.03	0.6676	0.9939	1.10	0.1595	0.9951	1.03	0.7519	0.9857	1.07	0.2960	0.7293	1.02	0.8094	0.8963	1.01	0.8292	0.9889	1.04	0.5212	0.9846
Growth factor receptor-bound protein 2	GRB2_HUMAN	P62993	0.95	0.4147	0.6663	0.89	0.0474	0.9982	0.99	0.8844	0.9960	0.95	0.3408	0.9939	0.93	0.2501	0.9951	1.06	0.3882	0.9856	0.90	0.0886	0.6824	1.10	0.1446	0.3898	0.89	0.0571	0.9889	0.92	0.1547	0.9607
GrpE protein homolog 1, mitochondrial	GRPE1_HUMAN	Q9HAV7	1.14	0.0472	0.2898	0.99	0.8881	0.9982	0.93	0.3165	0.9960	0.97	0.6802	0.9939	1.00	0.9445	0.9951	1.09	0.2886	0.9856	0.96	0.5630	0.8601	1.11	0.1392	0.3817	1.08	0.2335	0.9889	1.10	0.1391	0.9607
GTP-binding nuclear protein Ran	RAN_HUMAN	P62826	0.79	0.0006	0.1679	0.96	0.6056	0.9982	1.12	0.1583	0.9960	0.92	0.2190	0.9939	0.80	0.0019	0.9389	0.94	0.4805	0.9856	1.03	0.7265	0.9205	0.96	0.6527	0.8015	0.87	0.0612	0.9889	0.76	<0.0001	0.0634
GTP-binding protein SAR1a	SAR1A_HUMAN	Q9NR31	1.01	0.9239	0.9555	1.00	0.9796	0.9985	1.06	0.4853	0.9960	1.05	0.5304	0.9939	1.01	0.9369	0.9951	0.91	0.2878	0.9856	1.02	0.8473	0.9567	0.99	0.8667	0.9262	1.01	0.8853	0.9889	1.03	0.6450	0.9881
GTP-binding protein SAR1b	SAR1B_HUMAN	Q9Y6B6	1.00	0.9454	0.9684	0.98	0.7341	0.9982	1.09	0.2855	0.9960	0.97	0.6778	0.9939	0.97	0.6900	0.9951	0.86	0.0770	0.9856	0.93	0.3096	0.7378	1.14	0.0932	0.3107	0.93	0.3205	0.9889	1.00	0.9684	0.9985
GTP:AMP phosphotransferase AK3, mitochondrial	KAD3_HUMAN	Q9UIJ7	1.10	0.1771	0.4521	1.07	0.3505	0.9982	0.90	0.1713	0.9960	0.95	0.4740	0.9939	0.99	0.8409	0.9951	1.09	0.2847	0.9856	1.02	0.8035	0.9456	0.98	0.7817	0.8845	1.01	0.9102	0.9889	1.01	0.8628	0.9881
Guanine nucleotide-binding protein G(i) subunit alpha-2	GNAI2_HUMAN	P04899	0.82	0.0238	0.2873	1.03	0.7719	0.9982	1.03	0.7835	0.9960	0.98	0.8511	0.9939	0.90	0.2497	0.9951	0.95	0.6779	0.9856	1.12	0.2382	0.7187	0.82	0.0409	0.2167	0.92	0.3536	0.9889	0.90	0.2623	0.9607
Guanine nucleotide-binding protein G(I)/G(S)/G(O) subunit gamma-11	GBG11_HUMAN	P61952	0.99	0.8644	0.9348	0.95	0.4669	0.9982	1.00	0.9876	0.9960	1.01	0.8965	0.9939	0.97	0.6706	0.9951	0.96	0.6154	0.9856	0.92	0.2480	0.7187	1.11	0.1473	0.3939	1.09	0.1904	0.9889	1.00	0.9407	0.9938
Guanine nucleotide-binding protein G(I)/G(S)/G(O) subunit gamma-12	GBG12_HUMAN	Q9UBI6	0.88	0.2486	0.5210	1.00	0.9951	0.9985	1.03	0.8219	0.9960	1.03	0.8012	0.9939	0.96	0.7185	0.9951	0.98	0.8809	0.9959	1.07	0.5929	0.8646	0.74	0.0170	0.1636	0.87	0.2402	0.9889	0.99	0.9199	0.9938
Guanine nucleotide-binding protein G(I)/G(S)/G(O) subunit gamma-7	GBG7_HUMAN	O60262	1.02	0.7331	0.8760	0.96	0.5207	0.9982	1.10	0.1439	0.9960	0.97	0.6049	0.9939	1.00	0.9545	0.9951	0.87	0.0492	0.9568	1.10	0.1188	0.6868	0.98	0.7948	0.8884	0.96	0.5146	0.9889	1.05	0.4220	0.9607
Guanine nucleotide-binding protein G(I)/G(S)/G(T) subunit beta-1	GBB1_HUMAN	P62873	0.90	0.1501	0.4195	1.06	0.4619	0.9982	1.09	0.3426	0.9960	1.00	0.9834	0.9969	0.96	0.5999	0.9951	0.89	0.2252	0.9856	1.16	0.0527	0.6824	0.79	0.0048	0.1308	0.91	0.1940	0.9889	0.94	0.3896	0.9607
Guanine nucleotide-binding protein G(I)/G(S)/G(T) subunit beta-2	GBB2_HUMAN	P62879	0.89	0.1625	0.4354	1.17	0.0680	0.9982	1.04	0.7301	0.9960	0.99	0.9178	0.9939	0.92	0.3749	0.9951	0.98	0.8273	0.9959	1.14	0.1458	0.6871	0.74	0.0011	0.0763	0.97	0.7610	0.9889	0.92	0.3159	0.9607
Guanine nucleotide-binding protein G(s) subunit alpha isoforms short	GNAS2_HUMAN	P63092	0.94	0.3171	0.5859	1.04	0.5521	0.9982	0.96	0.6315	0.9960	1.04	0.5233	0.9939	1.00	0.9880	0.9967	1.03	0.7161	0.9857	1.03	0.6261	0.8830	1.02	0.8038	0.8927	1.01	0.8774	0.9889	0.98	0.7098	0.9881
Guanine nucleotide-binding protein subunit alpha-11	GNA11_HUMAN	P29992	0.96	0.5059	0.7279	1.04	0.4931	0.9982	0.94	0.3982	0.9960	1.01	0.8768	0.9939	1.04	0.5366	0.9951	1.00	0.9756	0.9967	1.10	0.1306	0.6868	0.80	0.0009	0.0763	0.98	0.7250	0.9889	1.05	0.3855	0.9607
Haloacid dehalogenase-like hydrolase domain-containing 5	HDHD5_HUMAN	Q9BXW7	1.17	0.0463	0.2898	1.06	0.4956	0.9982	0.84	0.0475	0.9960	0.96	0.6353	0.9939	1.04	0.6451	0.9951	1.13	0.1921	0.9856	0.91	0.2614	0.7187	1.08	0.3757	0.6062	1.13	0.1216	0.9889	1.04	0.6303	0.9881
Haloacid dehalogenase-like hydrolase domain-containing protein 2	HDHD2_HUMAN	Q9H0R4	1.06	0.5003	0.7246	0.95	0.5803	0.9982	1.05	0.6583	0.9960	0.83	0.0436	0.9874	0.91	0.3512	0.9951	1.04	0.7332	0.9857	0.88	0.1972	0.7149	1.22	0.0564	0.2474	0.94	0.4941	0.9889	0.94	0.4972	0.9765
Haloacid dehalogenase-like hydrolase domain-containing protein 3	HDHD3_HUMAN	Q9BSH5	1.19	0.0455	0.2898	1.15	0.1139	0.9982	0.90	0.3077	0.9960	0.98	0.8139	0.9939	1.05	0.6037	0.9951	1.03	0.7697	0.9857	0.89	0.2362	0.7187	0.89	0.2471	0.5122	1.04	0.6926	0.9889	1.15	0.1279	0.9607
Haptoglobin	HPT_HUMAN	P00738	1.03	0.8895	0.9421	1.01	0.9648	0.9982	1.10	0.7276	0.9960	1.12	0.6209	0.9939	1.29	0.2957	0.9951	0.84	0.5543	0.9856	1.42	0.1494	0.6871	0.73	0.2325	0.4959	0.92	0.7192	0.9889	1.06	0.7956	0.9881
Heat shock 70 kDa protein 1A	HS71A_HUMAN	P0DMV8	0.97	0.5051	0.7276	0.99	0.7951	0.9982	1.06	0.2368	0.9960	1.06	0.2247	0.9939	0.95	0.3235	0.9951	0.93	0.1981	0.9856	0.99	0.9056	0.9840	1.05	0.3640	0.5972	0.96	0.3584	0.9889	0.94	0.1465	0.9607
Heat shock 70 kDa protein 4	HSP74_HUMAN	P34932	0.94	0.3362	0.6019	0.94	0.3061	0.9982	0.99	0.8912	0.9960	0.96	0.4920	0.9939	0.90	0.0938	0.9951	1.03	0.6861	0.9856	0.85	0.0131	0.6779	1.15	0.0456	0.2274	0.95	0.3964	0.9889	0.89	0.0744	0.9607
Heat shock cognate 71 kDa protein	HSP7C_HUMAN	P11142	0.97	0.4435	0.6892	0.96	0.3345	0.9982	1.01	0.8737	0.9960	1.01	0.7602	0.9939	0.94	0.1390	0.9951	1.01	0.7818	0.9897	0.96	0.2779	0.7212	1.09	0.0336	0.2017	1.00	0.9850	0.9976	0.95	0.2017	0.9607
Heat shock protein 75 kDa, mitochondrial	TRAP1_HUMAN	Q12931	1.19	0.0112	0.2537	1.11	0.1242	0.9982	0.97	0.7029	0.9960	0.99	0.9034	0.9939	1.05	0.4664	0.9951	1.02	0.7904	0.9903	1.01	0.9163	0.9848	1.06	0.4853	0.6882	1.12	0.1223	0.9889	1.07	0.3707	0.9607
Heat shock protein beta-1	HSPB1_HUMAN	P04792	0.84	0.0089	0.2530	0.99	0.8700	0.9982	1.10	0.2314	0.9960	1.15	0.0327	0.9874	0.88	0.0594	0.9951	0.90	0.2296	0.9856	1.04	0.5946	0.8646	1.01	0.9102	0.9518	0.94	0.4049	0.9889	0.88	0.0571	0.9607
Heat shock protein beta-2	HSPB2_HUMAN	Q16082	1.01	0.9583	0.9734	0.80	0.0654	0.9982	0.88	0.3422	0.9960	0.93	0.5436	0.9939	1.04	0.7519	0.9951	1.18	0.2655	0.9856	0.90	0.3886	0.7835	1.40	0.0110	0.1498	1.03	0.7860	0.9889	1.09	0.4827	0.9652
Heat shock protein beta-3	HSPB3_HUMAN	Q12988	0.98	0.9109	0.9528	0.82	0.1509	0.9982	0.84	0.2680	0.9960	0.85	0.2306	0.9939	0.98	0.9021	0.9951	1.21	0.2556	0.9856	0.86	0.3077	0.7378	1.50	0.0058	0.1317	1.11	0.4523	0.9889	1.08	0.5611	0.9881
Heat shock protein beta-6	HSPB6_HUMAN	O14558	0.91	0.3548	0.6179	0.96	0.7108	0.9982	1.26	0.0475	0.9960	1.37	0.0017	0.8235	1.01	0.9547	0.9951	0.75	0.0198	0.9411	1.16	0.1833	0.7149	1.00	0.9885	0.9937	0.98	0.8368	0.9889	0.97	0.7916	0.9881
Heat shock protein beta-7	HSPB7_HUMAN	Q9UBY9	0.99	0.9016	0.9479	0.86	0.0382	0.9982	1.15	0.0895	0.9960	1.08	0.2943	0.9939	1.02	0.8228	0.9951	0.87	0.1167	0.9856	0.99	0.8825	0.9740	1.19	0.0283	0.1908	0.87	0.0521	0.9889	0.95	0.4762	0.9617

Heat shock protein beta-8	HSPB8_HUMAN	Q9UJY1	0.89	0.0795	0.3281	0.96	0.5486	0.9982	1.01	0.9371	0.9960	1.02	0.7618	0.9939	0.88	0.0613	0.9951	0.98	0.7746	0.9875	0.97	0.6205	0.8783	1.04	0.6117	0.7814	1.02	0.7350	0.9889	0.95	0.4670	0.9607
Heat shock protein HSP 90-alpha	HS90A_HUMAN	P07900	1.01	0.8709	0.9356	0.99	0.9195	0.9982	1.05	0.4672	0.9960	0.97	0.5542	0.9939	0.91	0.1070	0.9951	0.98	0.7852	0.9899	0.90	0.0998	0.6824	1.08	0.2503	0.5149	0.93	0.2102	0.9889	0.91	0.1017	0.9607
Heat shock protein HSP 90-beta	HS90B_HUMAN	P08238	1.05	0.3937	0.6514	1.00	0.9611	0.9982	1.04	0.4980	0.9960	1.01	0.8348	0.9939	0.95	0.3918	0.9951	0.99	0.8678	0.9959	0.94	0.2380	0.7187	1.08	0.1899	0.4480	0.97	0.5392	0.9889	0.94	0.2055	0.9607
Heat shock-related 70 kDa protein 2	HSP72_HUMAN	P54652	0.97	0.8049	0.9060	0.86	0.1809	0.9982	1.01	0.9669	0.9960	1.10	0.3833	0.9939	0.95	0.6734	0.9951	0.92	0.5469	0.9856	1.02	0.8967	0.9786	1.14	0.3061	0.5503	1.04	0.7562	0.9889	0.97	0.7866	0.9881
Hematopoietic progenitor cell antigen CD34	CD34_HUMAN	P28906	0.85	0.0391	0.2873	1.02	0.8475	0.9982	0.96	0.6611	0.9960	1.01	0.9012	0.9939	0.95	0.5637	0.9951	1.06	0.5386	0.9856	1.12	0.1979	0.7149	0.94	0.4661	0.6760	1.05	0.5274	0.9889	1.02	0.7766	0.9881
Heme oxygenase 2	HMOX2_HUMAN	P30519	0.96	0.4365	0.6830	1.04	0.4957	0.9982	1.00	0.9765	0.9960	1.08	0.1535	0.9874	1.03	0.5880	0.9951	0.94	0.4078	0.9856	0.99	0.8551	0.9606	0.88	0.0428	0.2201	1.05	0.3972	0.9889	1.11	0.0713	0.9607
Heme-binding protein 1	HEBP1_HUMAN	Q9NRV9	1.12	0.0601	0.3008	0.95	0.3944	0.9982	0.97	0.6251	0.9960	1.00	0.9737	0.9961	1.08	0.2072	0.9951	0.99	0.9456	0.9959	0.95	0.4131	0.7921	1.12	0.0841	0.2956	1.01	0.9118	0.9889	1.14	0.0268	0.9607
Heme-binding protein 2	HEBP2_HUMAN	Q9Y5Z4	1.05	0.5977	0.7927	0.95	0.5781	0.9982	0.98	0.8846	0.9960	0.94	0.5198	0.9939	0.96	0.6884	0.9951	1.08	0.4848	0.9856	0.89	0.2145	0.7171	1.26	0.0242	0.1763	0.94	0.5456	0.9889	0.96	0.6911	0.9881
Hemoglobin subunit alpha	HBA_HUMAN	P69905	1.01	0.9014	0.9479	0.77	0.0154	0.9982	1.01	0.9578	0.9960	0.81	0.0570	0.9874	1.06	0.6291	0.9951	1.12	0.3982	0.9856	1.10	0.4231	0.8053	1.25	0.0656	0.2631	0.92	0.4814	0.9889	1.01	0.9338	0.9938
Hemoglobin subunit beta	HBB_HUMAN	P68871	0.97	0.7674	0.8890	0.76	0.0114	0.9982	1.07	0.6027	0.9960	0.75	0.0090	0.9874	0.96	0.7008	0.9951	1.04	0.7617	0.9857	1.08	0.5246	0.8558	1.33	0.0229	0.1743	0.90	0.3724	0.9889	0.93	0.5136	0.9846
Hemoglobin subunit delta	HBD_HUMAN	P02042	0.98	0.8440	0.9230	0.87	0.2220	0.9982	1.09	0.4822	0.9960	0.79	0.0316	0.9874	0.89	0.3098	0.9951	0.97	0.8502	0.9959	1.04	0.7332	0.9218	1.39	0.0065	0.1326	0.94	0.5741	0.9889	0.83	0.0818	0.9607
Hemopexin	HEMO_HUMAN	P02790	0.93	0.5780	0.7764	1.19	0.1775	0.9982	1.30	0.0779	0.9960	0.99	0.9372	0.9939	0.94	0.6413	0.9951	0.78	0.1160	0.9856	1.12	0.4046	0.7880	0.77	0.0668	0.2648	0.87	0.2886	0.9889	0.86	0.2195	0.9607
Hepatocyte growth factor-regulated tyrosine kinase substrate	HGS_HUMAN	O14964	0.98	0.8243	0.9186	1.04	0.5978	0.9982	1.00	0.9729	0.9960	1.06	0.4097	0.9939	1.01	0.8912	0.9951	1.04	0.6763	0.9856	1.06	0.4767	0.8339	0.93	0.3599	0.5943	0.95	0.5069	0.9889	1.07	0.3255	0.9607
Heterochromatin protein 1-binding protein 3	HP1B3_HUMAN	Q5SSJ5	0.89	0.0786	0.3281	0.99	0.8799	0.9982	1.05	0.5762	0.9960	0.94	0.3385	0.9939	0.90	0.1304	0.9951	0.91	0.2727	0.9856	1.12	0.1218	0.6868	0.97	0.6524	0.8015	1.00	0.9984	0.9990	0.96	0.5256	0.9848
Heterogeneous nuclear ribonucleoprotein A/B	ROAA_HUMAN	Q99729	1.07	0.2979	0.5691	0.96	0.5395	0.9982	0.95	0.4323	0.9960	0.97	0.6327	0.9939	0.99	0.8475	0.9951	1.05	0.5269	0.9856	0.89	0.0771	0.6824	0.99	0.9309	0.9599	0.95	0.4504	0.9889	1.01	0.8692	0.9925
Heterogeneous nuclear ribonucleoprotein A1	ROA1_HUMAN	P09651	1.01	0.8856	0.9400	1.04	0.6114	0.9982	1.18	0.0524	0.9960	1.00	0.9899	0.9981	0.95	0.5292	0.9951	0.92	0.3796	0.9856	0.91	0.2419	0.7187	1.03	0.6824	0.8225	1.04	0.5797	0.9889	0.92	0.2506	0.9607
Heterogeneous nuclear ribonucleoprotein A3	ROA3_HUMAN	P51991	1.02	0.7287	0.8741	1.03	0.6217	0.9982	0.92	0.2081	0.9960	0.97	0.6288	0.9939	1.02	0.7911	0.9951	1.07	0.3522	0.9856	1.07	0.2699	0.7187	0.94	0.3228	0.5647	1.07	0.2721	0.9889	1.03	0.6431	0.9881
Heterogeneous nuclear ribonucleoprotein D-like	HNRDL_HUMAN	O14979	0.99	0.8886	0.9418	1.05	0.4401	0.9982	0.99	0.8708	0.9960	0.97	0.6777	0.9939	0.89	0.0881	0.9951	0.99	0.9170	0.9959	1.00	0.9981	0.9992	1.05	0.5217	0.7170	1.04	0.5871	0.9889	0.89	0.0720	0.9607
Heterogeneous nuclear ribonucleoprotein D0	HNRPD_HUMAN	Q14103	0.98	0.6729	0.8415	0.98	0.7594	0.9982	0.97	0.6374	0.9960	0.91	0.0961	0.9874	0.89	0.0507	0.9951	1.12	0.1109	0.9856	0.97	0.5931	0.8646	1.06	0.3569	0.5925	1.09	0.1624	0.9889	0.93	0.2084	0.9607
Heterogeneous nuclear ribonucleoprotein H	HNRH1_HUMAN	P31943	1.05	0.2669	0.5351	1.02	0.7124	0.9982	1.03	0.5948	0.9960	0.98	0.7104	0.9939	1.03	0.4480	0.9951	0.99	0.8250	0.9959	1.01	0.8073	0.9460	0.95	0.2694	0.5288	1.03	0.5432	0.9889	1.00	0.9242	0.9938
Heterogeneous nuclear ribonucleoprotein H3	HNRH3_HUMAN	P31942	1.03	0.6429	0.8286	1.02	0.6939	0.9982	1.01	0.9185	0.9960	0.95	0.3094	0.9939	0.93	0.2199	0.9951	0.98	0.7998	0.9954	0.95	0.3353	0.7458	1.03	0.5894	0.7646	0.99	0.7917	0.9889	0.98	0.7603	0.9881
Heterogeneous nuclear ribonucleoprotein K	HNRPK_HUMAN	P61978	1.05	0.3449	0.6081	1.05	0.3321	0.9982	0.96	0.4927	0.9960	0.93	0.1360	0.9874	1.00	0.9964	0.9996	1.07	0.2327	0.9856	0.96	0.4527	0.8205	1.02	0.6424	0.7988	0.96	0.3567	0.9889	0.95	0.2733	0.9607
Heterogeneous nuclear ribonucleoprotein L	HNRPL_HUMAN	P14866	1.01	0.9126	0.9536	1.05	0.4454	0.9982	1.04	0.5759	0.9960	0.97	0.6811	0.9939	0.95	0.4153	0.9951	0.93	0.3971	0.9856	1.09	0.2163	0.7187	0.93	0.2993	0.5483	0.92	0.2155	0.9889	0.95	0.3882	0.9607
Heterogeneous nuclear ribonucleoprotein M	HNRPM_HUMAN	P52272	0.88	0.0378	0.2873	0.93	0.2691	0.9982	1.09	0.2314	0.9960	0.89	0.0625	0.9874	0.94	0.3165	0.9951	0.90	0.1561	0.9856	0.97	0.6833	0.9001	0.88	0.0651	0.2631	0.90	0.0891	0.9889	0.95	0.3956	0.9607
Heterogeneous nuclear ribonucleoprotein Q	HNRPQ_HUMAN	O60506	0.93	0.1041	0.3654	1.00	0.9437	0.9982	1.03	0.6174	0.9960	0.99	0.9050	0.9939	0.91	0.0428	0.9951	1.00	0.9724	0.9967	0.96	0.4492	0.8181	1.03	0.5577	0.7400	1.05	0.3527	0.9889	0.93	0.1197	0.9607
Heterogeneous nuclear ribonucleoprotein R	HNRPR_HUMAN	O43390	0.94	0.3105	0.5811	1.05	0.4036	0.9982	0.94	0.3672	0.9960	0.87	0.0141	0.9874	0.89	0.0453	0.9951	1.12	0.0966	0.9856	1.01	0.8383	0.9534	1.03	0.6945	0.8265	1.04	0.4795	0.9889	0.91	0.1074	0.9607
Heterogeneous nuclear ribonucleoprotein U	HNRPU_HUMAN	Q00839	0.87	0.0245	0.2873	0.99	0.8370	0.9982	1.06	0.4273	0.9960	0.97	0.5688	0.9939	0.91	0.1338	0.9951	0.97	0.6445	0.9856	0.99	0.8607	0.9636	1.00	0.9874	0.9937	0.90	0.0983	0.9889	0.89	0.0606	0.9607
Heterogeneous nuclear ribonucleoprotein U-like protein 1	HNRL1_HUMAN	Q9BUJ2	1.17	0.1675	0.4377	1.06	0.6252	0.9982	0.92	0.5383	0.9960	0.84	0.1075	0.9874	0.96	0.7015	0.9951	1.15	0.3232	0.9856	1.08	0.5405	0.8573	0.83	0.1406	0.3837	1.07	0.5414	0.9889	1.03	0.8222	0.9881
Heterogeneous nuclear ribonucleoprotein U-like protein 2	HNRL2_HUMAN	Q1KMD3	1.05	0.4838	0.7195	1.00	0.9684	0.9982	1.02	0.7873	0.9960	1.07	0.3254	0.9939	0.97	0.6546	0.9951	0.96	0.6020	0.9856	0.92	0.2492	0.7187	1.04	0.5637	0.7445	1.10	0.1554	0.9889	1.07	0.3017	0.9607
Heterogeneous nuclear ribonucleoproteins A2/B1	ROA2_HUMAN	P22626	0.98	0.8004	0.9037	0.97	0.6607	0.9982	1.16	0.0478	0.9960	0.90	0.0961	0.9874	0.91	0.1533	0.9951	0.96	0.5832	0.9856	0.96	0.5332	0.8567	1.04	0.6388	0.7958	0.95	0.4865	0.9889	0.90	0.1061	0.9607
Heterogeneous nuclear ribonucleoproteins C1/C2	HNRPC_HUMAN	P07910	0.94	0.2248	0.4984	0.96	0.4270	0.9982	1.00	0.9779	0.9960	0.93	0.1659	0.9874	0.89	0.0350	0.9951	0.98	0.8177	0.9959	0.95	0.3819	0.7773	1.04	0.5063	0.7026	0.97	0.6250	0.9889	0.90	0.0436	0.9607
Hexokinase-1	HXK1_HUMAN	P19367	1.15	0.0311	0.2873	1.13	0.0660	0.9982	0.96	0.5941	0.9960	1.03	0.6670	0.9939	1.08	0.2529	0.9951	1.06	0.4826	0.9856	0.96	0.5914	0.8646	1.03	0.6600	0.8072	1.12	0.1002	0.9889	1.15	0.0271	0.9607
High mobility group protein B1	HMGB1_HUMAN	P09429	0.99	0.9169	0.9536	1.04	0.5793	0.9982	1.07	0.4329	0.9960	0.98	0.7748	0.9939	0.91	0.2388	0.9951	1.02	0.7994	0.9954	0.97	0.6765	0.8962	1.01	0.8969	0.9431	0.93	0.3422	0.9889	0.95	0.4928	0.9712
Histidine triad nucleotide-binding protein 1	HINT1_HUMAN	P49773	1.13	0.1748	0.4501	0.97	0.7261	0.9982	0.99	0.9233	0.9960	0.92	0.3699	0.9939	1.01	0.8867	0.9951	1.07	0.5112	0.9856	0.88	0.1942	0.7149	1.27	0.0154	0.1606	0.94	0.4959	0.9889	1.01	0.9100	0.9938
Histidine triad nucleotide-binding protein 2, mitochondrial	HINT2_HUMAN	Q9BX68	1.24	0.0111	0.2537	1.06	0.4821	0.9982	0.96	0.6587	0.9960	0.99	0.9023	0.9939	1.05	0.5955	0.9951	1.04	0.6975	0.9856	0.92	0.3702	0.7673	1.02	0.8386	0.9140	0.97	0.7418	0.9889	1.05	0.5997	0.9881
Histidine--tRNA ligase, mitochondrial	SYHM_HUMAN	P49590	1.15	0.0394	0.2873	1.01	0.9298	0.9982	0.98	0.7576	0.9960	0.98	0.7645	0.9939	1.10	0.1726	0.9951	0.97	0.6835	0.9856	0.91	0.1985	0.7149	1.03	0.7341	0.8540	1.18	0.0144	0.9889	1.13	0.0693	0.9607
Histidine-rich glycoprotein	HRG_HUMAN	P04196	0.93	0.5216	0.7363	1.05	0.6674	0.9982	1.12	0.3829	0.9960	0.99	0.9242	0.9939	0.96	0.7465	0.9951	0.93	0.5884	0.9856	1.02	0.8669	0.9661	0.85	0.1840	0.4384	0.99	0.9159	0.9889	1.02	0.8229	0.9881
Histone H1.0	H10_HUMAN	P07305	0.86	0.0790	0.3281	1.15	0.1095	0.9982	0.95	0.6279	0.9960	0.95	0.5116	0.9939	0.86	0.0791	0.9951	1.01	0.9107	0.9959	1.14	0.1640	0.6896	0.81	0.0289	0.1909	1.00	0.9652	0.9961	0.92	0.3405	0.9607
Histone H1.2	H12_HUMAN	P16403	0.94	0.3463	0.6090	1.03	0.6452	0.9982	1.05	0.5530	0.9960	0.87	0.0338	0.9874	0.91	0.1664	0.9951	0.94	0.4590	0.9856	1.00	0.9729	0.9937	0.94	0.3809	0.6115	0.93	0.2848	0.9889	0.97	0.6013	0.9881
Histone H1.4	H14_HUMAN	P10412	0.90	0.0667	0.3105	1.04	0.4684	0.9982	1.04	0.5833	0.9960	0.91	0.1196	0.9874	0.92	0.1589	0.9951	0.96	0.5920	0.9856	1.04	0.5151	0.8536	0.91	0.1569	0.4056	0.93	0.2536	0.9889	0.95	0.3383	0.9607
Histone H2A type 1-B/E	H2A1B_HUMAN	P04908	0.70	0.0101	0.2530	1.00	0.9959	0.9985	0.85	0.3302	0.9960	0.91	0.5319	0.9939	0.85	0.2619	0.9951	1.22	0.2611	0.9856	1.15	0.3491	0.7593	0.81	0.1827	0.4377	0.96	0.7974	0.9889	0.94	0.6568	0.9881
Histone H2A type 2-C	H2A2C_HUMAN	Q16777	0.69	0.0010	0.1794	1.03	0.8244	0.9982	0.95	0.7016	0.9960	0.94	0.6241	0.9939	0.82	0.1019	0.9951	1.01	0.9580	0.9967	1.24	0.0799	0.6824	0.75	0.0287	0.1909	0.97	0.7767	0.9889	0.89	0.3028	0.9607
Histone H2A.Z	H2AZ_HUMAN	P0C0S5	0.78	0.0072	0.2487	1.04	0.6910	0.9982	1.05	0.6785	0.9960	0.98	0.8525	0.9939	0.90	0.2698	0.9951	0.94	0.5710	0.9856	1.24	0.0338	0.6824	0.75	0.0055	0.1317	0.88	0.1957	0.9889	0.88	0.1582	0.9607
Histone H2AX	H2AX_HUMAN	P16104	0.70	0.0006	0.1679	1.00	0.9642	0.9982	0.90	0.3756	0.9960	0.94	0.5446	0.9939	0.82	0.0702	0.9951	1.06	0.6614	0.9856	1.13	0.3040	0.7378	0.77	0.0275	0.1861	0.94	0.5888	0.9889	0.83	0.0913	0.9607
Histone H2B type 1-C/E/F/G/I	H2B1C_HUMAN	P62807	0.73	0.0006	0.1679	0.93	0.4623	0.9982	1.02	0.8255	0.9960	0.89	0.2251	0.9939	0.82	0.0390	0.9951	0.96	0.7237	0.9857	1.18	0.0942	0.6824	0.83	0.0684	0.2676	0.91	0.3060	0.9889	0.87	0.1311	0.9607
Histone H3.1	H31_HUMAN	P68431	0.77	0.0107	0.2530	1.10	0.3763	0.9982	1.04	0.7719	0.9960	0.89	0.2793	0.9939	0.82	0.0700	0.9951	0.90	0.3981	0.9856	1.14	0.2390	0.7187	0.77	0.0201	0.1655	0.90	0.3324	0.9889	0.85	0.1273	0.9607
Histone H4	H4_HUMAN	P62805	0.74	0.0017	0.2005	1.05	0.6278	0.9982	1.03	0.8240	0.9960	0.95	0.6168	0.9939	0.80	0.0350	0.9951	0.93	0.5391	0.9856	1.18	0.1305	0.6868	0.79	0.0328	0.2017	0.87	0.1860	0.9889	0.84	0.0901	0.9607
Histone-binding protein RBBP4	RBBP4_HUMAN	Q09028	1.02	0.7179	0.8715	0.93	0.2234	0.9982	0.97	0.6755	0.9960	1.01	0.8997	0.9939	1.00	0.9918	0.9969	1.02	0.7332	0.9857	1.01	0.9294	0.9884	1.04	0.5312	0.7227	0.94	0.3038	0.9889	0.99	0.8284	0.9881
Histone-binding protein RBBP7	RBBP7_HUMAN	Q16576	0.96	0.4615	0.7031	0.91	0.0666	0.9982	0.98	0.7644	0.9960	0.89	0.0193	0.9874	1.01	0.8113	0.9951	1.01	0.8606	0.9959	0.95	0.3811	0.7767	1.07	0.2614	0.5247	0.93	0.1796	0.9889	1.02	0.7195	0.9881
Histone-lysine N-methyltransferase SMYD1	SMYD1_HUMAN	Q8NB12	0.98	0.8249	0.9186	0.96	0.6210	0.9982	1.06	0.5038	0.9960	1.08	0.3031	0.9939	0.97	0.6705	0.9951	0.88	0.1622	0.9856	0.95	0.5669	0.8601	1.26	0.0060	0.1317	0.93	0.3591	0.9889	0.98	0.7434	0.9881
HLA class I histocompatibility antigen, A alpha chain	HLAA_HUMAN	P01892	0.80	0.0718	0.3224	1.15	0.2546	0.9982	1.25	0.1158	0.9960	1.09	0.4774	0.9939	0.91	0.4758	0.9951	0.75	0.0562	0.9641	1.01	0.9326	0.9903	0.72	0.0154	0.1606	0.96	0.7525	0.9889	0.89	0.3607	0.9607
HLA class II histocompatibility antigen, DR alpha chain	DRA_HUMAN	P01903	0.93	0.4743	0.7121	1.12	0.2978	0.9982	0.93	0.5831	0.9960	1.06	0.6145	0.9939	0.93	0.5362	0.9951	1.01	0.9498	0.9967	1.15	0.2265	0.7187	0.94	0.5919	0.7646	0.82	0.0825	0.9889	0.93	0.4744	0.9617
HLA class II histocompatibility antigen, DRB1 beta chain	DRB1_HUMAN	Q5Y7A7	1.13	0.4221	0.6720	1.07	0.6467	0.9982	0.77	0.1512	0.9960	1.10	0.5382	0.9939	1.33	0.0740	0.9951	1.25	0.2437	0.9856	1.33	0.0861	0.6824	0.84	0.3003	0.5487	0.84	0.2656	0.9889	1.10	0.5208	0.9846
Hsc70-interacting protein	F10A1_HUMAN	P50502	1.04	0.5736	0.7732	0.97	0.6644	0.9982	1.01	0.9337	0.9960	0.94	0.3161	0.9939	0.95	0.4189	0.9951	1.06	0.4958	0.9856	0.90	0.1362	0.6871	1.11	0.1685	0.4217	0.93	0.2919	0.9889	0.95	0.4602	0.9607
Hsp90 co-chaperone Cdc37	CDC37_HUMAN	Q16543	0.97	0.6275	0.8160	0.96	0.5017	0.9982	1.02	0.7708	0.9960	0.99	0.8534	0.9939	0.96	0.4404	0.9951	1.02	0.7679	0.9857	0.96	0.4373	0.8116	1.13	0.0463	0.2280	0.90	0.0638	0.9889	0.88	0.0166	0.9607
Hydroxyacid-oxoacid transhydrogenase, mitochondrial	HOT_HUMAN	Q8IWW8	1.30	0.0312	0.2873	0.94	0.6262	0.9982	1.02	0.9076	0.9960	1.13	0.3146	0.9939	1.05	0.7270	0.9951	0.99	0.9492	0.9967	0.96	0.7375	0.9218	1.31	0.0496	0.2366	1.06	0.6704	0.9889	1.01	0.9422	0.9938
Hydroxyacyl-coenzyme A dehydrogenase, mitochondrial	HCDH_HUMAN	Q16836	1.20	0.0335	0.2873	1.01	0.8804	0.9982	0.87	0.1657	0.9960	0.96	0.6489	0.9939	0.99	0.9343	0.9951	1.17	0.1302	0.9856	0.95	0.5381	0.8573	1.10	0.3331	0.5705	1.00	0.9691	0.9971	1.06	0.5162	0.9846
Hydroxyacylglutathione hydrolase, mitochondrial	GLO2_HUMAN	Q16775	1.16	0.0212	0.2873	1.05	0.4974	0.9982	0.93	0.3096	0.9960	0.95	0.4791	0.9939	1.04	0.5577	0.9951	1.11	0.1954	0.9856	0.94	0.3611	0.7659	1.10	0.1958	0.4564	1.10	0.1682	0.9889	1.08	0.2391	0.9607
Hydroxymethylglutaryl-CoA lyase, mitochondrial	HMGCL_HUMAN	P35914	0.96	0.5153	0.7328	0.99	0.8758	0.9982	0.93	0.3112	0.9960	0.91	0.1013	0.9874	0.93	0.2529	0.9951	1.09	0.2098	0.9856	0.94	0.2891	0.7212	1.07	0.3087	0.5505	1.03	0.6394	0.9889	0.94	0.3129	0.9607
Hydroxysteroid dehydrogenase-like protein 2	HSDL2_HUMAN	Q6YN16	1.26	0.0010	0.1794	1.08	0.2981	0.9982	0.94	0.4888	0.9960	1.02	0.8023	0.9939	1.11	0.1845	0.9951	1.05	0.6129	0.9856	0.93	0.3231	0.7400	1.10	0.2368	0.5003	1.04	0.6426	0.9889	1.10	0.1861	0.9607
Hypoxia up-regulated protein 1	HYOU1_HUMAN	Q9Y4L1	0.93	0.1873	0.4611	0.97	0.6374	0.9982	1.03	0.7128	0.9960	1.10	0.1014	0.9874	0.98	0.7484	0.9951	0.96	0.5827	0.9856	1.07	0.2522	0.7187	1.00	0.9739	0.9853	0.95	0.4178	0.9889	0.94	0.2925	0.9607
Immunoglobulin alpha-2 heavy chain	IGA2_HUMAN	P0DOX2	0.92	0.6789	0.8437	0.90	0.6014	0.9982	0.95	0.8422	0.9960	1.18	0.4230	0.9939	1.24	0.3202	0.9951	1.10	0.7097	0.9857	1.33	0.1884	0.7149	0.80	0.3303	0.5682	1.30	0.2176	0.9889	1.23	0.3169	0.9607
Immunoglobulin gamma-1 heavy chain	IGG1_HUMAN	P0DOX5	0.82	0.2167	0.4882	1.01	0.9369	0.9982	1.06	0.7479	0.9960	0.95	0.7339	0.9939	1.02	0.8907	0.9951	0.99	0.9577	0.9967	1.33	0.2219	0.7187	0.65	0.0162	0.1636	1.09	0.6002	0.9889	1.01	0.9508	0.9938
Immunoglobulin heavy constant alpha 1	IGHA1_HUMAN	P01876	0.86	0.4179	0.6675	0.90	0.5521	0.9982	1.02	0.9135	0.9960	1.08	0.6596	0.9939	1.04	0.8402	0.9951	0.97	0.8928	0.9959	1.27	0.2275	0.7187	0.80	0.2853	0.5390	1.27	0.2128	0.9889	0.98	0.9232	0.9938
Immunoglobulin heavy constant gamma 2	IGHG2_HUMAN	P01859	0.87	0.4754	0.7131	1.03	0.8604	0.9982	1.08	0.7418	0.9960	1.02	0.9369	0.9939	1.01	0.9408	0.9951	0.87	0.5691	0.9856	1.23	0.3105	0.7378	0.74	0.1582	0.4067	1.07	0.7367	0.9889	0.98	0.9248	0.9938
Immunoglobulin heavy constant gamma 3	IGHG3_HUMAN	P01860	0.98	0.8568	0.9307	0.94	0.6215	0.9982	1.06	0.7267	0.9960	0.95	0.7100	0.9939	1.11	0.4631	0.9951	0.95	0.7470	0.9857	1.05	0.7552	0.9282	0.78	0.0964	0.3134	0.97	0.8085	0.9889	1.05	0.6924	0.9881
Immunoglobulin heavy constant gamma 4	IGHG4_HUMAN	P01861	0.91	0.6674	0.8387	0.95	0.8293	0.9982	1.30	0.3177	0.9960	0.91	0.6728	0.9939	1.06	0.8158	0.9951	0.83	0.5072	0.9856	1.26	0.3349	0.7458	0.74	0.2314	0.4953	1.15	0.5462	0.9889	1.00	0.9999	0.9999
Immunoglobulin heavy constant mu	IGHM_HUMAN	P01871	1.02	0.8796	0.9384	1.17	0.3212	0.9982	1.15	0.4382	0.9960	1.07	0.6533	0.9939	1.19	0.3019	0.9951	0.91	0.6330	0.9856	1.21	0.2669	0.7187	0.61	0.0050	0.1313	0.96	0.7913	0.9889	1.02	0.8814	0.9938
Immunoglobulin heavy variable 1-2	HV102_HUMAN	P23083	0.82	0.2447	0.5184	0.99	0.9458	0.9982	1.06	0.7590	0.9960	0.86	0.3557	0.9939	0.97	0.8562	0.9951	0.90	0.6268	0.9856	1.17	0.3671	0.7673	0.73	0.0843	0.2956	1.00	0.9808	0.9976	0.93	0.6770	0.9881
Immunoglobulin heavy variable 1-69D	HV69D_HUMAN	A0A0B4J2H0	0.82	0.3166	0.5859	0.98	0.9084	0.9982	1.22	0.3880	0.9960	0.77	0.1787	0.9939	0.84	0.3988	0.9951	0.91	0.7095	0.9857	0.97	0.8856	0.9752	0.74	0.1723	0.4237	1.15	0.4929	0.9889	0.95	0.8004	0.9881
Immunoglobulin kappa constant	IGKC_HUMAN	P01834	0.82	0.2043	0.4823	1.02	0.9125	0.9982	1.02	0.9075	0.9960	0.98	0.8905	0.9939	1.00	0.9909	0.9969	0.97	0.8800	0.9959	1.18	0.3265	0.7401	0.75	0.1030	0.3273	1.09	0.6050	0.9889	1.00	0.9794	0.9992
Immunoglobulin kappa light chain	IGK_HUMAN	P0DOX7	0.88	0.4901	0.7219	1.01	0.9722	0.9982	1.02	0.9171	0.9960	0.96	0.8432	0.9939	1.14	0.5006	0.9951	1.02	0.9193	0.9959	1.23	0.3026	0.7361	0.63	0.0222	0.1739	1.03	0.8682	0.9889	1.08	0.6836	0.9881
Immunoglobulin lambda constant 2	IGLC2_HUMAN	P0DOY2	0.84	0.2792	0.5517	0.99	0.9319	0.9982	1.06	0.7555	0.9960	0.97	0.8388	0.9939	1.05	0.7765	0.9951	0.92	0.6875	0.9856	1.25	0.1873	0.7149	0.67	0.0222	0.1739	1.02	0.9248	0.9889	0.97	0.8436	0.9881
Immunoglobulin lambda-1 light chain	IGL1_HUMAN	P0DOX8	0.85	0.2921	0.5638	0.96	0.7741	0.9982	1.11	0.5667	0.9960	1.00	0.9916	0.9981	1.00	0.9989	0.9996	0.89	0.5498	0.9856	1.23	0.2236	0.7187	0.68	0.0258	0.1832	0.99	0.9639	0.9961	0.96	0.8017	0.9881
Importin subunit alpha-3	IMA3_HUMAN	O00629	0.98	0.7622	0.8882	0.93	0.3125	0.9982	1.03	0.7478	0.9960	0.99	0.8779	0.9939	0.90	0.1498	0.9951	0.96	0.5974	0.9856	0.87	0.0558	0.6824	1.19	0.0192	0.1651	0.89	0.1077	0.9889	0.87	0.0450	0.9607
Importin subunit beta-1	IMB1_HUMAN	Q14974	1.14	0.0813	0.3290	1.01	0.9289	0.9982	1.07	0.4706	0.9960	1.04	0.5746	0.9939	1.08	0.3461	0.9951	0.96	0.7020	0.9857	0.95	0.5115	0.8518	1.18	0.0500	0.2372	0.99	0.9124	0.9889	1.03	0.7431	0.9881
Inorganic pyrophosphatase 2, mitochondrial	IPYR2_HUMAN	Q9H2U2	1.08	0.2987	0.5699	0.98	0.8276	0.9982	0.95	0.5400	0.9960	0.94	0.4385	0.9939	1.04	0.5897	0.9951	1.06	0.5015	0.9856	0.89	0.1478	0.6871	1.05	0.5259	0.7205	1.03	0.7118	0.9889	1.00	0.9828	0.9992
Inositol monophosphatase 1	IMPA1_HUMAN	P29218	1.16	0.1383	0.4015	0.97	0.7577	0.9982	0.93	0.5426	0.9960	0.85	0.1008	0.9874	0.96	0.6634	0.9951	1.20	0.1326	0.9856	0.81	0.0446	0.6824	1.23	0.0646	0.2631	0.92	0.4227	0.9889	1.06	0.5895	0.9881
Inositol-3-phosphate synthase 1	INO1_HUMAN	Q9NPH2	1.00	0.9581	0.9734	1.01	0.8632	0.9982	0.96	0.6143	0.9960	1.00	0.9855	0.9981	0.94	0.3815	0.9951	1.07	0.3521	0.9856	0.94	0.3939	0.7856	1.06	0.4191	0.6429	1.02	0.8130	0.9889	0.97	0.6805	0.9881
Insulin-degrading enzyme	IDE_HUMAN	P14735	1.14	0.0926	0.3485	1.06	0.4720	0.9982	0.89	0.2101	0.9960	0.97	0.6787	0.9939	0.98	0.8186	0.9951	1.15	0.1367	0.9856	0.89	0.1416	0.6871	1.14	0.1257	0.3597	1.01	0.9394	0.9922	1.03	0.7305	0.9881
Insulin-like growth factor-binding protein 7	IBP7_HUMAN	Q16270	0.97	0.7495	0.8824	1.09	0.3541	0.9982	0.90	0.2913	0.9960	1.07	0.4175	0.9939	0.96	0.6490	0.9951	1.16	0.1696	0.9856	1.11	0.2748	0.7196	0.82	0.0409	0.2167	1.07	0.4282	0.9889	1.01	0.9057	0.9938
Integrin alpha-6	ITA6_HUMAN	P23229	0.84	0.0369	0.2873	0.95	0.5583	0.9982	1.00	0.9630	0.9960	0.90	0.2214	0.9939	0.95	0.5683	0.9951	0.94	0.5542	0.9856	1.15	0.1130	0.6824	0.88	0.1698	0.4217	0.90	0.2122	0.9889	0.86	0.0696	0.9607
Integrin alpha-7	ITA7_HUMAN	Q13683	0.93	0.4046	0.6612	1.05	0.5833	0.9982	1.18	0.0770	0.9960	1.00	0.9564	0.9939	1.04	0.6278	0.9951	0.78	0.0125	0.9411	1.11	0.2266	0.7187	0.76	0.0020	0.0815	0.93	0.3569	0.9889	0.97	0.7067	0.9881
Integrin alpha-V	ITAV_HUMAN	P06756	0.88	0.1829	0.4579	1.17	0.0970	0.9982	0.99	0.9478	0.9960	1.01	0.8860	0.9939	0.93	0.4555	0.9951	0.94	0.6003	0.9856	1.11	0.3129	0.7400	0.72	0.0013	0.0763	1.03	0.7423	0.9889	0.95	0.5578	0.9881
Integrin beta-1	ITB1_HUMAN	P05556	0.84	0.0371	0.2873	1.00	0.9766	0.9984	1.08	0.4122	0.9960	0.94	0.4263	0.9939	0.93	0.3886	0.9951	0.92	0.3932	0.9856	1.06	0.4887	0.8385	0.79	0.0097	0.1392	0.94	0.4706	0.9889	0.94	0.4472	0.9607
Integrin-linked protein kinase	ILK_HUMAN	Q13418	0.94	0.2451	0.5184	0.97	0.6384	0.9982	1.04	0.4983	0.9960	1.02	0.7074	0.9939	0.99	0.8449	0.9951	0.93	0.2601	0.9856	1.04	0.5322	0.8567	1.00	0.9727	0.9847	0.98	0.7108	0.9889	0.99	0.8067	0.9881
Inter-alpha-trypsin inhibitor heavy chain H1	ITIH1_HUMAN	P19827	0.83	0.0742	0.3253	1.07	0.5175	0.9982	0.97	0.8011	0.9960	1.06	0.5731	0.9939	0.87	0.2157	0.9951	1.03	0.8209	0.9959	1.25	0.0491	0.6824	0.83	0.1143	0.3452	1.15	0.2107	0.9889	0.88	0.2119	0.9607
Inter-alpha-trypsin inhibitor heavy chain H2	ITIH2_HUMAN	P19823	0.81	0.0600	0.3008	1.10	0.3968	0.9982	1.10	0.4590	0.9960	1.17	0.1575	0.9874	0.96	0.7495	0.9951	0.92	0.5438	0.9856	1.27	0.0413	0.6824	0.75	0.0197	0.1655	1.05	0.6699	0.9889	0.89	0.2927	0.9607
Inter-alpha-trypsin inhibitor heavy chain H4	ITIH4_HUMAN	Q14624	0.85	0.1634	0.4357	1.12	0.3233	0.9982	0.98	0.8530	0.9960	0.93	0.5544	0.9939	0.93	0.5311	0.9951	1.02	0.8663	0.9959	1.15	0.2505	0.7187	0.70	0.0042	0.1244	1.03	0.7786	0.9889	0.89	0.2991	0.9607
Interleukin enhancer-binding factor 2	ILF2_HUMAN	Q12905	1.03	0.6138	0.8030	1.10	0.1686	0.9982	1.06	0.4658	0.9960	0.97	0.6024	0.9939	0.90	0.1404	0.9951	0.94	0.4841	0.9856	1.01	0.9348	0.9903	0.95	0.4866	0.6890	0.98	0.7276	0.9889	0.95	0.4007	0.9607
Interleukin enhancer-binding factor 3	ILF3_HUMAN	Q12906	1.00	0.9804	0.9886	1.07	0.1962	0.9982	1.01	0.8499	0.9960	0.96	0.3734	0.9939	0.95	0.3513	0.9951	1.01	0.8414	0.9959	1.02	0.7422	0.9230	0.96	0.5047	0.7017	1.00	0.9268	0.9889	0.96	0.3920	0.9607
Iron-sulfur cluster assembly 2 homolog, mitochondrial	ISCA2_HUMAN	Q86U28	1.16	0.0346	0.2873	1.02	0.8177	0.9982	0.87	0.1035	0.9960	1.04	0.5855	0.9939	1.07	0.3551	0.9951	1.19	0.0480	0.9535	0.97	0.6807	0.8997	1.12	0.1787	0.4326	1.14	0.0744	0.9889	1.12	0.1277	0.9607
Iron-sulfur cluster assembly enzyme ISCU, mitochondrial	ISCU_HUMAN	Q9H1K1	1.05	0.3375	0.6020	1.06	0.3136	0.9982	0.97	0.5751	0.9960	1.02	0.6608	0.9939	0.98	0.6909	0.9951	1.02	0.7470	0.9857	0.99	0.8497	0.9579	0.96	0.4719	0.6780	1.06	0.2568	0.9889	1.01	0.8217	0.9881
Iron-sulfur protein NUBPL	NUBPL_HUMAN	Q8TB37	1.13	0.0878	0.3401	0.93	0.3346	0.9982	0.95	0.5250	0.9960	1.00	0.9493	0.9939	1.14	0.0936	0.9951	1.07	0.4861	0.9856	0.92	0.2747	0.7196	1.21	0.0202	0.1659	1.11	0.1680	0.9889	1.11	0.1673	0.9607
Isoamyl acetate-hydrolyzing esterase 1 homolog	IAH1_HUMAN	Q2TAA2	1.06	0.4108	0.6642	1.05	0.4838	0.9982	1.03	0.6551	0.9960	1.00	0.9421	0.9939	1.02	0.7846	0.9951	1.11	0.1885	0.9856	0.96	0.5681	0.8601	1.03	0.6684	0.8105	1.03	0.6772	0.9889	1.03	0.6594	0.9881
Isobutyryl-CoA dehydrogenase, mitochondrial	ACAD8_HUMAN	Q9UKU7	1.17	0.0131	0.2710	1.09	0.1934	0.9982	0.99	0.9235	0.9960	0.95	0.4279	0.9939	1.02	0.7542	0.9951	1.06	0.4789	0.9856	0.89	0.0966	0.6824	1.10	0.2149	0.4815	1.04	0.5483	0.9889	1.05	0.5050	0.9818
Isochorismatase domain-containing protein 1	ISOC1_HUMAN	Q96CN7	1.13	0.1521	0.4208	0.96	0.6023	0.9982	0.95	0.6401	0.9960	1.00	0.9730	0.9961	0.98	0.8067	0.9951	1.10	0.3921	0.9856	0.87	0.1300	0.6868	1.25	0.0189	0.1650	0.91	0.3072	0.9889	0.97	0.7521	0.9881
Isochorismatase domain-containing protein 2	ISOC2_HUMAN	Q96AB3	1.03	0.6503	0.8297	1.03	0.7093	0.9982	1.03	0.7412	0.9960	0.95	0.5284	0.9939	0.90	0.1707	0.9951	1.01	0.8775	0.9959	0.90	0.1614	0.6896	1.11	0.1897	0.4480	0.91	0.2030	0.9889	0.90	0.1616	0.9607
Isocitrate dehydrogenase [NAD] subunit alpha, mitochondrial	IDH3A_HUMAN	P50213	1.10	0.1959	0.4729	1.05	0.5146	0.9982	0.91	0.2513	0.9960	0.90	0.1745	0.9907	0.95	0.5081	0.9951	1.16	0.1115	0.9856	0.86	0.0654	0.6824	1.09	0.3193	0.5613	1.04	0.5857	0.9889	1.04	0.5735	0.9881

Isocitrate dehydrogenase [NAD] subunit beta, mitochondrial	IDH3B_HUMAN	O43837	1.16	0.0450	0.2894	1.06	0.4202	0.9982	0.93	0.4101	0.9960	0.93	0.3449	0.9939	1.02	0.8317	0.9951	1.11	0.2441	0.9856	0.87	0.0779	0.6824	1.11	0.2287	0.4943	1.09	0.2475	0.9889	1.08	0.2747	0.9607
Isocitrate dehydrogenase [NAD] subunit gamma, mitochondrial	IDH3G_HUMAN	P51553	1.10	0.1680	0.4381	1.06	0.4000	0.9982	0.93	0.3569	0.9960	0.94	0.4109	0.9939	0.97	0.6394	0.9951	1.10	0.2582	0.9856	0.90	0.1693	0.6935	1.13	0.1264	0.3604	1.08	0.2925	0.9889	1.03	0.6986	0.9881
Isocitrate dehydrogenase [NADP] cytoplasmic	IDHC_HUMAN	O75874	0.99	0.9380	0.9653	1.05	0.5367	0.9982	0.98	0.8575	0.9960	0.92	0.3063	0.9939	1.00	0.9730	0.9951	1.06	0.5724	0.9856	1.06	0.4556	0.8211	0.97	0.6959	0.8265	0.91	0.2305	0.9889	0.94	0.4135	0.9607
Isocitrate dehydrogenase [NADP], mitochondrial	IDHP_HUMAN	P48735	1.22	0.0212	0.2873	1.12	0.2073	0.9982	0.95	0.6148	0.9960	1.03	0.7221	0.9939	1.05	0.5605	0.9951	1.03	0.7548	0.9857	0.94	0.5325	0.8567	1.15	0.1594	0.4083	1.03	0.7480	0.9889	1.05	0.6046	0.9881
Isoleucine--tRNA ligase, mitochondrial	SYIM_HUMAN	Q9NSE4	1.09	0.0641	0.3091	1.02	0.6682	0.9982	0.96	0.4840	0.9960	1.04	0.4304	0.9939	1.06	0.2685	0.9951	1.04	0.4736	0.9856	0.99	0.8952	0.9781	1.06	0.2982	0.5473	1.07	0.1876	0.9889	1.04	0.4092	0.9607
Isovaleryl-CoA dehydrogenase, mitochondrial	IVD_HUMAN	P26440	0.98	0.7218	0.8735	0.96	0.5457	0.9982	1.00	0.9901	0.9960	0.91	0.1686	0.9874	0.90	0.1186	0.9951	1.03	0.7682	0.9857	0.95	0.5216	0.8558	0.97	0.7105	0.8360	1.05	0.4895	0.9889	0.97	0.7098	0.9881
Junction plakoglobin	PLAK_HUMAN	P14923	1.11	0.0795	0.3281	1.04	0.5577	0.9982	0.97	0.6085	0.9960	0.94	0.2555	0.9939	1.00	0.9615	0.9951	0.99	0.8987	0.9959	0.90	0.0883	0.6824	1.01	0.9225	0.9569	1.03	0.5863	0.9889	1.08	0.1702	0.9607
Junctophilin-2	JPH2_HUMAN	Q9BR39	0.88	0.1196	0.3810	0.95	0.5595	0.9982	1.15	0.1557	0.9960	1.01	0.8961	0.9939	0.95	0.5880	0.9951	0.87	0.1738	0.9856	0.94	0.4618	0.8243	0.98	0.7899	0.8865	0.97	0.7022	0.9889	0.99	0.8565	0.9881
Kelch-like protein 41	KLH41_HUMAN	O60662	1.03	0.6892	0.8496	0.94	0.3586	0.9982	1.07	0.4046	0.9960	1.10	0.1577	0.9874	1.00	0.9642	0.9951	0.86	0.0739	0.9641	0.98	0.8196	0.9481	1.16	0.0562	0.2474	0.95	0.4298	0.9889	0.97	0.6980	0.9881
Keratin, type I cytoskeletal 10	K1C10_HUMAN	P13645	0.80	0.2689	0.5381	1.10	0.6254	0.9982	1.86	0.0064	0.9960	1.62	0.0134	0.9874	0.92	0.6755	0.9951	0.55	0.0129	0.9411	0.97	0.9047	0.9840	1.17	0.4761	0.6794	0.93	0.7080	0.9889	0.85	0.4280	0.9607
Keratin, type I cytoskeletal 16	K1C16_HUMAN	P08779	0.98	0.8813	0.9384	1.09	0.6114	0.9982	0.98	0.9202	0.9960	1.04	0.7967	0.9939	0.88	0.4514	0.9951	1.09	0.6562	0.9856	1.05	0.7935	0.9431	1.29	0.1562	0.4056	1.07	0.6693	0.9889	0.81	0.2032	0.9607
Keratin, type I cytoskeletal 17	K1C17_HUMAN	Q04695	0.89	0.4415	0.6867	1.09	0.5583	0.9982	1.19	0.3302	0.9960	1.27	0.1166	0.9874	0.87	0.3681	0.9951	0.87	0.4510	0.9856	0.99	0.9733	0.9937	1.17	0.3666	0.5972	0.85	0.3176	0.9889	0.80	0.1358	0.9607
Keratin, type I cytoskeletal 9	K1C9_HUMAN	P35527	1.05	0.8105	0.9092	0.72	0.0883	0.9982	1.31	0.2298	0.9960	1.36	0.1109	0.9874	1.34	0.1386	0.9951	0.84	0.4770	0.9856	0.92	0.6861	0.9014	1.41	0.1089	0.3358	0.80	0.2749	0.9889	1.13	0.5393	0.9881
Keratin, type II cytoskeletal 1	K2C1_HUMAN	P04264	0.89	0.5382	0.7494	0.94	0.7392	0.9982	1.52	0.0446	0.9960	1.52	0.0186	0.9874	1.09	0.6655	0.9951	0.75	0.1999	0.9856	1.00	0.9983	0.9992	1.34	0.1509	0.4002	0.89	0.5282	0.9889	0.97	0.8707	0.9925
Keratin, type II cytoskeletal 2 epidermal	K22E_HUMAN	P35908	0.92	0.6990	0.8576	1.09	0.6712	0.9982	1.41	0.1459	0.9960	1.86	0.0017	0.8235	1.00	0.9914	0.9969	0.73	0.2229	0.9856	1.17	0.4806	0.8352	1.32	0.2249	0.4919	1.05	0.8168	0.9889	0.99	0.9529	0.9938
Keratin, type II cytoskeletal 5	K2C5_HUMAN	P13647	0.91	0.5891	0.7867	1.24	0.2342	0.9982	1.26	0.2657	0.9960	1.66	0.0032	0.8235	1.01	0.9715	0.9951	0.87	0.5360	0.9856	1.17	0.4048	0.7880	1.26	0.2523	0.5154	1.15	0.4559	0.9889	0.91	0.5945	0.9881
Keratin, type II cytoskeletal 6A	K2C6A_HUMAN	P02538	0.94	0.7267	0.8738	1.27	0.2100	0.9982	0.99	0.9574	0.9960	1.19	0.3695	0.9939	1.02	0.9394	0.9951	1.08	0.7507	0.9857	1.33	0.1569	0.6871	1.18	0.4290	0.6500	1.18	0.3897	0.9889	0.92	0.6531	0.9881
Ketimine reductase mu-crystallin	CRYM_HUMAN	Q14894	1.26	0.0843	0.3334	1.01	0.9175	0.9982	0.89	0.4741	0.9960	0.74	0.0233	0.9874	0.90	0.4774	0.9951	1.23	0.2171	0.9856	0.72	0.0196	0.6779	1.30	0.0813	0.2916	1.12	0.4153	0.9889	1.10	0.4973	0.9765
Ketosamine-3-kinase	KT3K_HUMAN	Q9HA64	1.06	0.2414	0.5162	0.97	0.5086	0.9982	1.00	0.9420	0.9960	0.94	0.2096	0.9939	1.09	0.1068	0.9951	1.03	0.6874	0.9856	0.98	0.6612	0.8929	1.01	0.9251	0.9573	0.99	0.8205	0.9889	1.08	0.1171	0.9607
Kinectin	KTN1_HUMAN	Q86UP2	0.99	0.7602	0.8882	0.96	0.3141	0.9982	1.04	0.4143	0.9960	1.02	0.5627	0.9939	1.01	0.8007	0.9951	0.95	0.3476	0.9856	0.94	0.1572	0.6871	0.99	0.8492	0.9195	1.03	0.4921	0.9889	1.04	0.3012	0.9607
Kinesin-1 heavy chain	KINH_HUMAN	P33176	0.90	0.0654	0.3105	0.91	0.0815	0.9982	1.00	0.9845	0.9960	1.06	0.3079	0.9939	0.96	0.5141	0.9951	1.07	0.3117	0.9856	0.93	0.2146	0.7171	1.15	0.0241	0.1763	1.05	0.3730	0.9889	1.00	0.9805	0.9992
Kininogen-1	KNG1_HUMAN	P01042	0.84	0.1201	0.3810	1.14	0.2515	0.9982	1.17	0.2276	0.9960	1.00	0.9730	0.9961	0.95	0.6795	0.9951	0.87	0.3391	0.9856	1.08	0.5040	0.8476	0.72	0.0069	0.1326	0.96	0.7255	0.9889	0.89	0.3031	0.9607
KN motif and ankyrin repeat domain-containing protein 2	KANK2_HUMAN	Q63ZY3	0.95	0.5754	0.7750	1.06	0.5163	0.9982	0.94	0.5530	0.9960	1.09	0.3514	0.9939	1.02	0.8146	0.9951	1.01	0.9471	0.9964	1.12	0.2550	0.7187	0.86	0.1447	0.3898	0.94	0.5283	0.9889	1.02	0.8087	0.9881
Kynurenine--oxoglutarate transaminase 3	KAT3_HUMAN	Q6YP21	1.10	0.2157	0.4877	1.09	0.2981	0.9982	0.90	0.2764	0.9960	1.05	0.5600	0.9939	1.03	0.7246	0.9951	1.13	0.2078	0.9856	1.06	0.5196	0.8558	1.02	0.8368	0.9133	1.09	0.3086	0.9889	1.03	0.7485	0.9881
L-2-hydroxyglutarate dehydrogenase, mitochondrial	L2HDH_HUMAN	Q9H9P8	1.23	0.0090	0.2530	1.08	0.3749	0.9982	0.94	0.5420	0.9960	1.08	0.3721	0.9939	1.17	0.0665	0.9951	1.05	0.6077	0.9856	1.00	0.9677	0.9937	1.07	0.4738	0.6786	1.11	0.2229	0.9889	1.13	0.1264	0.9607
L-lactate dehydrogenase A chain	LDHA_HUMAN	P00338	1.20	0.0552	0.2942	1.20	0.0477	0.9982	0.95	0.6650	0.9960	0.97	0.7490	0.9939	1.02	0.8466	0.9951	1.09	0.4708	0.9856	1.05	0.5963	0.8646	0.77	0.0128	0.1544	0.97	0.7189	0.9889	1.09	0.3426	0.9607
L-lactate dehydrogenase B chain	LDHB_HUMAN	P07195	1.15	0.1543	0.4236	1.01	0.8871	0.9982	0.93	0.5352	0.9960	0.90	0.2850	0.9939	0.96	0.6557	0.9951	1.16	0.2257	0.9856	0.89	0.2377	0.7187	1.23	0.0598	0.2522	0.94	0.5437	0.9889	1.00	0.9893	0.9992
L-xylulose reductase	DCXR_HUMAN	Q7Z4W1	1.18	0.0471	0.2898	1.00	0.9699	0.9982	1.05	0.6006	0.9960	0.92	0.3141	0.9939	1.02	0.8224	0.9951	0.96	0.6829	0.9856	1.00	0.9684	0.9937	1.06	0.5542	0.7387	1.02	0.8360	0.9889	1.04	0.6736	0.9881
Lactadherin	MFGM_HUMAN	Q08431	0.92	0.5565	0.7594	1.10	0.4956	0.9982	0.90	0.4955	0.9960	1.05	0.7355	0.9939	1.01	0.9277	0.9951	1.00	0.9899	0.9992	1.10	0.5041	0.8476	0.67	0.0057	0.1317	1.17	0.2553	0.9889	1.15	0.2864	0.9607
Lactoylglutathione lyase	LGUL_HUMAN	Q04760	1.14	0.1036	0.3650	0.98	0.8486	0.9982	1.00	0.9844	0.9960	0.94	0.4577	0.9939	1.05	0.5470	0.9951	1.10	0.3540	0.9856	0.91	0.2785	0.7212	1.19	0.0524	0.2413	0.93	0.3903	0.9889	1.05	0.5757	0.9881
Lamin-B1	LMNB1_HUMAN	P20700	0.81	0.0148	0.2807	1.09	0.3023	0.9982	1.03	0.7468	0.9960	0.89	0.1918	0.9939	0.89	0.1987	0.9951	1.02	0.8380	0.9959	1.05	0.6167	0.8752	0.84	0.0629	0.2592	0.91	0.3009	0.9889	0.87	0.1016	0.9607
Lamin-B2	LMNB2_HUMAN	Q03252	0.84	0.0186	0.2873	1.03	0.7061	0.9982	1.01	0.9486	0.9960	0.98	0.7951	0.9939	0.93	0.3073	0.9951	1.00	0.9683	0.9967	1.10	0.1999	0.7149	0.82	0.0150	0.1606	1.02	0.7919	0.9889	0.99	0.8603	0.9881
Laminin subunit alpha-2	LAMA2_HUMAN	P24043	0.99	0.8712	0.9356	0.93	0.2522	0.9982	1.15	0.0510	0.9960	1.01	0.8308	0.9939	0.99	0.8959	0.9951	0.90	0.1591	0.9856	0.96	0.5451	0.8590	1.05	0.4689	0.6776	0.95	0.4503	0.9889	0.92	0.1554	0.9607
Laminin subunit alpha-4	LAMA4_HUMAN	Q16363	0.88	0.0617	0.3038	0.96	0.5238	0.9982	1.10	0.2074	0.9960	0.99	0.9221	0.9939	0.90	0.1302	0.9951	0.93	0.3809	0.9856	1.12	0.1041	0.6824	1.06	0.4079	0.6344	0.90	0.1368	0.9889	0.84	0.0106	0.9304
Laminin subunit alpha-5	LAMA5_HUMAN	O15230	0.96	0.6446	0.8286	1.12	0.2032	0.9982	1.11	0.3066	0.9960	0.91	0.2699	0.9939	0.96	0.6393	0.9951	0.91	0.3780	0.9856	1.08	0.4034	0.7880	0.88	0.1748	0.4260	0.97	0.7259	0.9889	0.90	0.2286	0.9607
Laminin subunit beta-1	LAMB1_HUMAN	P07942	0.91	0.1403	0.4030	0.93	0.2504	0.9982	1.10	0.1782	0.9960	1.03	0.6463	0.9939	0.95	0.4726	0.9951	0.93	0.3247	0.9856	1.06	0.3902	0.7838	1.02	0.7919	0.8865	0.92	0.2052	0.9889	0.87	0.0210	0.9607
Laminin subunit beta-2	LAMB2_HUMAN	P55268	0.98	0.6976	0.8566	0.95	0.3096	0.9982	1.18	0.0083	0.9960	0.96	0.4395	0.9939	0.99	0.8181	0.9951	0.87	0.0370	0.9411	0.99	0.8848	0.9751	1.07	0.2626	0.5247	1.00	0.9934	0.9985	0.99	0.9157	0.9938
Laminin subunit gamma-1	LAMC1_HUMAN	P11047	0.89	0.0545	0.2939	0.92	0.1675	0.9982	1.13	0.0856	0.9960	0.99	0.8255	0.9939	0.91	0.1710	0.9951	0.92	0.2777	0.9856	1.03	0.6425	0.8893	1.05	0.4717	0.6780	0.93	0.2563	0.9889	0.85	0.0101	0.9304
Lanosterol synthase	ERG7_HUMAN	P48449	1.16	0.1058	0.3654	1.14	0.1387	0.9982	1.14	0.1981	0.9960	1.05	0.5756	0.9939	1.02	0.7942	0.9951	0.82	0.0735	0.9641	0.93	0.4313	0.8109	1.26	0.0209	0.1683	1.00	0.9865	0.9976	1.07	0.4353	0.9607
Latent-transforming growth factor beta-binding protein 2	LTBP2_HUMAN	Q14767	0.90	0.5410	0.7503	1.00	0.9955	0.9985	0.94	0.7583	0.9960	1.04	0.8453	0.9939	1.00	0.9980	0.9996	1.01	0.9764	0.9969	1.05	0.7962	0.9431	0.62	0.0157	0.1622	1.01	0.9505	0.9945	1.09	0.6280	0.9881
Latent-transforming growth factor beta-binding protein 4	LTBP4_HUMAN	Q8N2S1	0.90	0.4321	0.6798	1.23	0.1117	0.9982	1.06	0.7051	0.9960	1.00	0.9780	0.9961	0.90	0.4483	0.9951	0.93	0.6509	0.9856	1.01	0.9523	0.9923	0.72	0.0195	0.1655	0.97	0.8409	0.9889	1.04	0.7335	0.9881
Leiomodin-2	LMOD2_HUMAN	Q6P5Q4	0.93	0.2857	0.5588	0.94	0.3868	0.9982	1.02	0.8349	0.9960	1.11	0.1246	0.9874	1.01	0.8419	0.9951	0.90	0.2104	0.9856	1.02	0.7411	0.9230	1.03	0.6793	0.8205	1.04	0.5639	0.9889	0.99	0.8434	0.9881
Leiomodin-3	LMOD3_HUMAN	Q0VAK6	1.11	0.1508	0.4208	1.02	0.7968	0.9982	1.05	0.5882	0.9960	1.13	0.0873	0.9874	1.20	0.0145	0.9951	0.91	0.3157	0.9856	0.97	0.7286	0.9213	1.04	0.6620	0.8078	0.95	0.5153	0.9889	1.15	0.0509	0.9607
LEM domain-containing protein 2	LEMD2_HUMAN	Q8NC56	0.88	0.1101	0.3718	0.96	0.5988	0.9982	1.04	0.6828	0.9960	0.88	0.1186	0.9874	0.86	0.0596	0.9951	0.94	0.5549	0.9856	0.90	0.2387	0.7187	0.88	0.1688	0.4217	0.93	0.4125	0.9889	0.98	0.8421	0.9881
Leucine-rich alpha-2-glycoprotein	A2GL_HUMAN	P02750	0.98	0.9039	0.9491	1.28	0.0527	0.9982	1.08	0.6117	0.9960	1.14	0.2894	0.9939	1.20	0.1609	0.9951	0.92	0.5826	0.9856	1.33	0.0355	0.6824	0.64	0.0014	0.0763	0.84	0.1908	0.9889	0.95	0.6751	0.9881
Leucine-rich PPR motif-containing protein, mitochondrial	LPPRC_HUMAN	P42704	1.22	0.0168	0.2873	1.05	0.5888	0.9982	0.98	0.8095	0.9960	1.02	0.8227	0.9939	1.05	0.6018	0.9951	1.01	0.9146	0.9959	0.97	0.7555	0.9282	1.10	0.2948	0.5464	1.11	0.2297	0.9889	1.10	0.2589	0.9607
Leucine-rich repeat-containing protein 47	LRC47_HUMAN	Q8N1G4	0.92	0.1543	0.4236	0.97	0.6444	0.9982	1.04	0.5693	0.9960	0.99	0.8275	0.9939	0.97	0.5581	0.9951	1.02	0.8216	0.9959	0.94	0.2665	0.7187	0.99	0.8596	0.9237	1.12	0.0473	0.9889	0.99	0.8942	0.9938
Leucyl-cystinyl aminopeptidase	LCAP_HUMAN	Q9UIQ6	1.02	0.7889	0.8986	1.06	0.3529	0.9982	1.04	0.5824	0.9960	1.10	0.1129	0.9874	1.06	0.3604	0.9951	0.91	0.2092	0.9856	1.03	0.6832	0.9001	0.96	0.5266	0.7205	1.07	0.3209	0.9889	1.09	0.1496	0.9607
Leukocyte elastase inhibitor	ILEU_HUMAN	P30740	0.97	0.6512	0.8297	1.03	0.6711	0.9982	1.07	0.4416	0.9960	0.98	0.7561	0.9939	0.92	0.3039	0.9951	0.97	0.7074	0.9857	0.90	0.1899	0.7149	1.01	0.8592	0.9237	0.91	0.2294	0.9889	0.80	0.0021	0.9304
Leukotriene A-4 hydrolase	LKHA4_HUMAN	P09960	1.17	0.0542	0.2939	1.01	0.8753	0.9982	0.94	0.5294	0.9960	0.95	0.5050	0.9939	1.05	0.5509	0.9951	1.17	0.1253	0.9856	0.93	0.4389	0.8116	1.19	0.0511	0.2403	0.94	0.4856	0.9889	1.01	0.8992	0.9938
LIM and calponin homology domains-containing protein 1	LIMC1_HUMAN	Q9UPQ0	1.28	0.0041	0.2034	1.10	0.2615	0.9982	1.00	0.9750	0.9960	0.96	0.6667	0.9939	1.07	0.4354	0.9951	1.01	0.9360	0.9959	0.83	0.0385	0.6824	1.03	0.7842	0.8852	1.07	0.4462	0.9889	1.18	0.0505	0.9607
LIM and senescent cell antigen-like-containing domain protein 1	LIMS1_HUMAN	P48059	1.11	0.2192	0.4919	1.17	0.0594	0.9982	0.95	0.5782	0.9960	1.06	0.5045	0.9939	1.10	0.2569	0.9951	1.00	0.9694	0.9967	1.14	0.1391	0.6871	0.83	0.0469	0.2295	1.08	0.3732	0.9889	1.11	0.2233	0.9607
LIM and SH3 domain protein 1	LASP1_HUMAN	Q14847	1.07	0.3038	0.5744	1.18	0.0075	0.9982	0.93	0.3594	0.9960	1.00	0.9995	0.9995	1.03	0.7011	0.9951	1.10	0.2503	0.9856	1.03	0.6618	0.8929	0.81	0.0025	0.0888	1.01	0.8711	0.9889	0.99	0.9322	0.9938
LIM domain and actin-binding protein 1	LIMA1_HUMAN	Q9UHB6	1.02	0.8490	0.9270	1.06	0.4819	0.9982	0.76	0.0046	0.9960	0.96	0.6051	0.9939	1.04	0.6548	0.9951	1.24	0.0385	0.9411	1.08	0.3788	0.7749	0.88	0.1777	0.4315	1.01	0.9254	0.9889	1.07	0.4029	0.9607
LIM domain only protein 7	LMO7_HUMAN	Q8WWI1	0.99	0.8936	0.9439	1.05	0.3751	0.9982	1.05	0.4266	0.9960	0.92	0.1311	0.9874	0.91	0.0932	0.9951	0.96	0.5766	0.9856	0.84	0.0014	0.6779	1.05	0.3935	0.6226	1.00	0.9557	0.9961	0.95	0.3515	0.9607
LIM domain-binding protein 3	LDB3_HUMAN	O75112	1.13	0.0392	0.2873	1.02	0.6967	0.9982	0.96	0.5232	0.9960	1.08	0.1718	0.9874	1.11	0.0886	0.9951	1.02	0.7605	0.9857	0.99	0.8122	0.9478	1.06	0.3994	0.6263	1.05	0.4702	0.9889	1.11	0.0747	0.9607
Lipoamide acyltransferase component of branched-chain alpha-keto acid	ODB2_HUMAN	P11182	1.01	0.9215	0.9555	1.01	0.8866	0.9982	1.01	0.8942	0.9960	0.99	0.8062	0.9939	1.01	0.8878	0.9951	0.94	0.4159	0.9856	0.97	0.6921	0.9047	1.08	0.2807	0.5337	1.09	0.1629	0.9889	1.05	0.4406	0.9607
Lipoma-preferred partner	LPP_HUMAN	Q93052	0.95	0.5371	0.7490	1.06	0.4253	0.9982	1.07	0.4545	0.9960	0.86	0.0434	0.9874	0.88	0.0970	0.9951	1.01	0.9273	0.9959	0.87	0.0823	0.6824	0.91	0.2514	0.5154	0.99	0.9414	0.9922	1.02	0.8155	0.9881
Lipoprotein lipase	LIPL_HUMAN	P06858	0.82	0.1331	0.3961	0.78	0.0702	0.9982	1.40	0.0307	0.9960	1.11	0.4606	0.9939	0.88	0.3686	0.9951	0.67	0.0144	0.9411	1.03	0.8334	0.9513	1.39	0.0296	0.1909	0.99	0.9457	0.9922	0.83	0.1803	0.9607
Liver carboxylesterase 1	EST1_HUMAN	P23141	1.20	0.0489	0.2898	0.99	0.8900	0.9982	0.95	0.6395	0.9960	1.16	0.1060	0.9874	1.05	0.5787	0.9951	1.01	0.9189	0.9959	1.06	0.5529	0.8601	1.13	0.2243	0.4912	0.89	0.2182	0.9889	0.99	0.9264	0.9938
Lon protease homolog, mitochondrial	LONM_HUMAN	P36776	1.13	0.0326	0.2873	1.03	0.6133	0.9982	0.99	0.8795	0.9960	1.01	0.9163	0.9939	1.07	0.2187	0.9951	0.96	0.5870	0.9856	0.95	0.3554	0.7644	0.97	0.6009	0.7719	1.09	0.1511	0.9889	1.06	0.3229	0.9607
Long-chain fatty acid transport protein 6	S27A6_HUMAN	Q9Y2P4	1.06	0.6545	0.8297	0.91	0.5132	0.9982	0.80	0.1479	0.9960	0.95	0.6974	0.9939	0.95	0.7131	0.9951	1.24	0.1952	0.9856	0.94	0.6886	0.9041	1.31	0.0732	0.2781	1.11	0.4474	0.9889	0.94	0.6393	0.9881
Long-chain-fatty-acid--CoA ligase 1	ACSL1_HUMAN	P33121	1.11	0.1675	0.4377	1.05	0.5349	0.9982	0.98	0.8307	0.9960	1.02	0.7823	0.9939	1.05	0.5257	0.9951	0.97	0.7665	0.9857	1.02	0.7851	0.9374	1.16	0.0893	0.3023	0.99	0.9137	0.9889	1.06	0.4533	0.9607
Long-chain-fatty-acid--CoA ligase 3	ACSL3_HUMAN	O95573	0.93	0.2565	0.5261	0.97	0.6226	0.9982	1.07	0.3231	0.9960	1.19	0.0036	0.8235	1.05	0.4384	0.9951	0.91	0.2164	0.9856	1.11	0.1082	0.6824	0.97	0.6492	0.7998	1.04	0.5618	0.9889	1.02	0.7364	0.9881
Low molecular weight phosphotyrosine protein phosphatase	PPAC_HUMAN	P24666	1.19	0.0073	0.2487	0.98	0.7475	0.9982	0.96	0.6036	0.9960	1.01	0.8565	0.9939	1.09	0.2347	0.9951	1.04	0.6778	0.9856	0.97	0.6932	0.9047	1.13	0.0950	0.3123	0.98	0.8030	0.9889	1.05	0.4446	0.9607
LRP chaperone MESD	MESD_HUMAN	Q14696	0.95	0.5429	0.7505	1.10	0.2391	0.9982	1.05	0.5978	0.9960	1.12	0.1581	0.9874	1.03	0.7618	0.9951	0.99	0.9401	0.9959	1.09	0.2914	0.7236	0.88	0.1641	0.4153	0.92	0.2776	0.9889	0.91	0.2225	0.9607
Lumican	LUM_HUMAN	P51884	0.64	0.0431	0.2873	0.87	0.5322	0.9982	0.73	0.2288	0.9960	0.92	0.6893	0.9939	0.77	0.2638	0.9951	1.21	0.4951	0.9856	1.15	0.5654	0.8601	0.60	0.0348	0.2017	1.10	0.6770	0.9889	0.95	0.8156	0.9881
Lupus La protein	LA_HUMAN	P05455	1.05	0.4960	0.7237	0.94	0.3415	0.9982	0.95	0.5061	0.9960	0.90	0.1180	0.9874	0.97	0.6694	0.9951	1.14	0.1177	0.9856	0.89	0.0990	0.6824	1.17	0.0424	0.2201	0.95	0.4776	0.9889	0.96	0.5475	0.9881
LYR motif-containing protein 4	LYRM4_HUMAN	Q9HD34	1.05	0.4993	0.7246	1.13	0.1027	0.9982	0.91	0.3109	0.9960	1.04	0.5846	0.9939	1.01	0.9252	0.9951	1.10	0.3249	0.9856	0.93	0.4030	0.7880	1.06	0.4918	0.6924	1.12	0.1352	0.9889	1.10	0.2327	0.9607
Lysine--tRNA ligase	SYK_HUMAN	Q15046	1.11	0.0203	0.2873	1.07	0.1667	0.9982	1.02	0.7263	0.9960	1.07	0.1276	0.9874	1.04	0.4062	0.9951	1.01	0.8621	0.9959	1.01	0.8661	0.9660	1.01	0.8117	0.8963	1.02	0.6925	0.9889	1.03	0.4758	0.9617
Lysophospholipase-like protein 1	LYPL1_HUMAN	Q5VWZ2	1.24	0.0136	0.2710	1.05	0.5839	0.9982	0.86	0.1598	0.9960	0.96	0.6787	0.9939	1.03	0.7619	0.9951	1.13	0.2556	0.9856	0.88	0.1593	0.6871	1.19	0.0770	0.2846	1.04	0.6461	0.9889	1.10	0.2871	0.9607
Lysosomal acid phosphatase	PPAL_HUMAN	P11117	0.98	0.8071	0.9060	0.98	0.7683	0.9982	1.05	0.5401	0.9960	1.07	0.2989	0.9939	1.08	0.2232	0.9951	0.87	0.0682	0.9641	1.14	0.0472	0.6824	0.83	0.0069	0.1326	0.96	0.5271	0.9889	0.96	0.5138	0.9846
Lysosomal alpha-glucosidase	LYAG_HUMAN	P10253	1.11	0.1193	0.3810	1.02	0.8069	0.9982	0.91	0.2488	0.9960	1.05	0.4598	0.9939	1.09	0.2450	0.9951	1.13	0.1412	0.9856	1.09	0.2209	0.7187	1.01	0.9055	0.9475	0.93	0.3125	0.9889	1.03	0.6992	0.9881
Lysosome-associated membrane glycoprotein 1	LAMP1_HUMAN	P11279	0.90	0.3544	0.6179	1.09	0.4670	0.9982	0.96	0.7760	0.9960	1.06	0.6375	0.9939	1.07	0.5852	0.9951	0.97	0.8170	0.9959	1.14	0.2873	0.7212	0.63	0.0003	0.0621	0.98	0.8734	0.9889	1.04	0.7674	0.9881
m-AAA protease-interacting protein 1, mitochondrial	MAIP1_HUMAN	Q8WWC4	1.10	0.1849	0.4606	1.05	0.4918	0.9982	0.98	0.7765	0.9960	1.06	0.4020	0.9939	1.07	0.3606	0.9951	0.99	0.9181	0.9959	0.99	0.8783	0.9720	1.13	0.1244	0.3578	1.07	0.3624	0.9889	1.05	0.4823	0.9652
Macrophage migration inhibitory factor	MIF_HUMAN	P14174	1.23	0.0394	0.2873	1.15	0.1621	0.9982	0.89	0.3415	0.9960	0.96	0.7078	0.9939	1.00	0.9912	0.9969	1.18	0.1953	0.9856	0.85	0.1275	0.6868	0.99	0.9254	0.9573	0.97	0.7510	0.9889	0.98	0.8428	0.9881
Malate dehydrogenase, cytoplasmic	MDHC_HUMAN	P40925	1.18	0.1180	0.3810	0.96	0.7084	0.9982	0.92	0.4960	0.9960	0.94	0.5537	0.9939	0.99	0.9251	0.9951	1.19	0.1740	0.9856	0.86	0.1902	0.7149	1.35	0.0094	0.1378	0.98	0.8552	0.9889	1.03	0.7869	0.9881
Malate dehydrogenase, mitochondrial	MDHM_HUMAN	P40926	1.14	0.1149	0.3763	1.07	0.4290	0.9982	0.92	0.3679	0.9960	1.04	0.6277	0.9939	0.99	0.9021	0.9951	1.12	0.2623	0.9856	0.93	0.4457	0.8181	1.10	0.3202	0.5613	1.06	0.5243	0.9889	1.01	0.8625	0.9881
Malonyl-CoA decarboxylase, mitochondrial	DCMC_HUMAN	O95822	1.23	0.0059	0.2484	1.08	0.3059	0.9982	0.99	0.9042	0.9960	0.97	0.7070	0.9939	1.05	0.5653	0.9951	1.00	0.9833	0.9992	0.95	0.5260	0.8558	1.05	0.5585	0.7400	1.08	0.3209	0.9889	1.09	0.2467	0.9607
Mammalian ependymin-related protein 1	EPDR1_HUMAN	Q9UM22	1.32	0.0080	0.2487	1.17	0.1264	0.9982	1.06	0.6345	0.9960	1.03	0.8091	0.9939	1.10	0.3694	0.9951	0.92	0.5042	0.9856	0.82	0.0832	0.6824	1.01	0.9239	0.9571	1.01	0.9541	0.9961	1.10	0.3686	0.9607
Mannose-6-phosphate isomerase	MPI_HUMAN	P34949	1.12	0.2021	0.4813	0.98	0.7851	0.9982	0.86	0.1574	0.9960	1.15	0.1250	0.9874	1.08	0.4482	0.9951	1.22	0.0721	0.9641	0.91	0.3231	0.7400	1.15	0.1810	0.4355	1.02	0.8568	0.9889	1.01	0.9132	0.9938
MAP7 domain-containing protein 1	MA7D1_HUMAN	Q3KQU3	0.99	0.8559	0.9307	1.04	0.5997	0.9982	1.14	0.0964	0.9960	0.95	0.4262	0.9939	0.97	0.6802	0.9951	0.89	0.1871	0.9856	0.94	0.4414	0.8152	0.96	0.6389	0.7958	1.05	0.4745	0.9889	0.93	0.3166	0.9607
Matrin-3	MATR3_HUMAN	P43243	0.92	0.0742	0.3253	0.97	0.4988	0.9982	1.01	0.9041	0.9960	0.95	0.3255	0.9939	0.97	0.5850	0.9951	0.98	0.7288	0.9857	0.93	0.1552	0.6871	1.00	0.9994	0.9994	0.95	0.3181	0.9889	1.01	0.8825	0.9938
Matrix-remodeling-associated protein 7	MXRA7_HUMAN	P84157	0.93	0.3116	0.5811	1.00	0.9627	0.9982	0.95	0.5705	0.9960	0.99	0.9376	0.9939	0.92	0.2813	0.9951	1.01	0.9328	0.9959	0.97	0.7372	0.9218	0.95	0.5750	0.7520	1.00	0.9652	0.9961	0.94	0.3889	0.9607
Medium-chain acyl-CoA ligase ACSF2, mitochondrial	ACSF2_HUMAN	Q96CM8	1.02	0.7844	0.8986	1.07	0.3929	0.9982	0.89	0.2359	0.9960	1.04	0.6541	0.9939	1.01	0.9296	0.9951	1.07	0.5166	0.9856	1.15	0.1146	0.6824	0.94	0.5233	0.7179	0.94	0.4831	0.9889	1.00	0.9871	0.9992
Medium-chain specific acyl-CoA dehydrogenase, mitochondrial	ACADM_HUMAN	P11310	1.21	0.0578	0.2973	0.93	0.4787	0.9982	0.87	0.2296	0.9960	1.02	0.8799	0.9939	0.99	0.9064	0.9951	1.19	0.1654	0.9856	0.93	0.5001	0.8476	1.31	0.0184	0.1650	1.05	0.6235	0.9889	1.06	0.5652	0.9881
Membrane primary amine oxidase	AOC3_HUMAN	Q16853	0.81	0.1279	0.3939	1.04	0.7705	0.9982	1.03	0.8510	0.9960	0.84	0.2187	0.9939	0.80	0.1308	0.9951	0.92	0.6374	0.9856	1.06	0.6905	0.9047	0.75	0.0667	0.2648	0.81	0.1492	0.9889	0.87	0.3044	0.9607
Membrane-associated progesterone receptor component 1	PGRC1_HUMAN	O00264	0.88	0.0997	0.3570	1.04	0.6054	0.9982	0.99	0.8949	0.9960	1.13	0.0924	0.9874	1.01	0.8665	0.9951	1.00	0.9788	0.9982	1.23	0.0093	0.6779	0.86	0.0729	0.2778	1.00	0.9927	0.9985	0.96	0.6221	0.9881
Membrane-associated progesterone receptor component 2	PGRC2_HUMAN	O15173	0.83	0.0353	0.2873	0.90	0.2346	0.9982	1.02	0.8620	0.9960	0.98	0.8360	0.9939	0.82	0.0290	0.9951	0.93	0.4973	0.9856	0.99	0.9268	0.9880	1.17	0.1199	0.3534	0.88	0.1659	0.9889	0.87	0.1219	0.9607
Metaxin-1	MTX1_HUMAN	Q13505	1.22	0.0218	0.2873	1.16	0.1032	0.9982	0.92	0.4245	0.9960	1.17	0.0710	0.9874	1.22	0.0304	0.9951	1.06	0.6006	0.9856	0.99	0.9446	0.9903	1.01	0.8976	0.9431	1.10	0.3104	0.9889	1.24	0.0130	0.9304
Metaxin-2	MTX2_HUMAN	O75431	1.02	0.7737	0.8916	1.00	0.9503	0.9982	1.03	0.7773	0.9960	0.94	0.4062	0.9939	0.95	0.5170	0.9951	0.92	0.4119	0.9856	0.95	0.5749	0.8630	1.08	0.3643	0.5972	1.08	0.3312	0.9889	1.04	0.6635	0.9881
Methanethiol oxidase	SBP1_HUMAN	Q13228	1.09	0.4057	0.6612	1.04	0.6689	0.9982	1.07	0.5462	0.9960	0.94	0.5206	0.9939	0.97	0.7888	0.9951	1.07	0.5595	0.9856	0.93	0.4661	0.8274	1.22	0.0715	0.2736	0.95	0.6044	0.9889	0.97	0.7487	0.9881
Methionine adenosyltransferase 2 subunit beta	MAT2B_HUMAN	Q9NZL9	1.01	0.9233	0.9555	1.05	0.5701	0.9982	1.00	0.9707	0.9960	0.98	0.8331	0.9939	0.95	0.6254	0.9951	0.99	0.9265	0.9959	0.92	0.4274	0.8085	0.91	0.3618	0.5967	1.12	0.2231	0.9889	0.99	0.9136	0.9938
Methionine-R-sulfoxide reductase B2, mitochondrial	MSRB2_HUMAN	Q9Y3D2	0.98	0.7906	0.8986	0.97	0.7074	0.9982	0.99	0.9539	0.9960	1.05	0.5402	0.9939	0.99	0.9437	0.9951	1.03	0.7700	0.9857	1.03	0.7818	0.9363	1.13	0.1975	0.4593	0.90	0.2318	0.9889	0.95	0.5169	0.9846
Methylcrotonoyl-CoA carboxylase beta chain, mitochondrial	MCCB_HUMAN	Q9HCC0	1.15	0.0486	0.2898	1.01	0.8986	0.9982	0.96	0.6400	0.9960	0.97	0.7131	0.9939	1.01	0.8717	0.9951	1.03	0.6951	0.9856	0.95	0.4479	0.8181	1.18	0.0296	0.1909	1.14	0.0620	0.9889	1.08	0.2488	0.9607
Methylcrotonoyl-CoA carboxylase subunit alpha, mitochondrial	MCCA_HUMAN	Q96RQ3	1.19	0.0754	0.3266	0.94	0.5543	0.9982	0.83	0.1127	0.9960	0.99	0.9565	0.9939	1.11	0.3201	0.9951	1.16	0.2328	0.9856	1.12	0.2980	0.7319	1.21	0.0901	0.3035	1.13	0.2262	0.9889	1.10	0.3501	0.9607
Methylglutaconyl-CoA hydratase, mitochondrial	AUHM_HUMAN	Q13825	1.09	0.1344	0.3972	1.07	0.2046	0.9982	0.95	0.4353	0.9960	1.04	0.5080	0.9939	1.02	0.7324	0.9951	1.05	0.4777	0.9856	0.93	0.2596	0.7187	1.04	0.5753	0.7520	1.13	0.0400	0.9889	1.06	0.2962	0.9607
Methylmalonate-semialdehyde dehydrogenase [acylating], mitochondrial	MMSA_HUMAN	Q02252	1.12	0.2119	0.4843	0.97	0.7547	0.9982	1.03	0.7543	0.9960	0.87	0.1395	0.9874	0.94	0.4998	0.9951	0.97	0.7716	0.9857	0.88	0.2075	0.7149	1.04	0.7127	0.8360	1.09	0.3842	0.9889	1.03	0.7293	0.9881
Methylmalonyl-CoA epimerase, mitochondrial	MCEE_HUMAN	Q96PE7	1.19	0.0428	0.2873	1.05	0.5929	0.9982	0.86	0.1322	0.9960	0.91	0.2672	0.9939	0.93	0.4414	0.9951	1.19	0.1011	0.9856	0.89	0.2132	0.7171	1.18	0.0957	0.3129	1.10	0.3158	0.9889	1.02	0.8464	0.9881

Methylmalonyl-CoA mutase, mitochondrial	MUTA_HUMAN	P22033	1.13	0.0987	0.3566	1.11	0.1605	0.9982	0.92	0.3044	0.9960	1.00	0.9908	0.9981	1.03	0.6748	0.9951	1.10	0.3121	0.9856	0.97	0.6694	0.8929	0.99	0.8712	0.9284	1.08	0.3295	0.9889	1.05	0.4660	0.9607
MICOS complex subunit MIC13	MIC13_HUMAN	Q5XKP0	1.02	0.7625	0.8882	1.03	0.6951	0.9982	0.98	0.7937	0.9960	1.02	0.7030	0.9939	1.02	0.7258	0.9951	0.97	0.6972	0.9856	0.92	0.2302	0.7187	1.05	0.4755	0.6794	1.09	0.1874	0.9889	1.06	0.3640	0.9607
MICOS complex subunit MIC19	MIC19_HUMAN	Q9NX63	0.87	0.0795	0.3281	1.01	0.8675	0.9982	0.99	0.8817	0.9960	0.92	0.2862	0.9939	0.94	0.4354	0.9951	0.97	0.7301	0.9857	0.97	0.7321	0.9218	0.98	0.7944	0.8884	1.12	0.1711	0.9889	1.00	0.9561	0.9938
MICOS complex subunit MIC26	MIC26_HUMAN	Q9BUR5	1.01	0.8950	0.9442	1.09	0.2457	0.9982	1.04	0.6456	0.9960	1.05	0.5395	0.9939	1.06	0.4726	0.9951	0.96	0.6469	0.9856	0.94	0.4111	0.7917	1.01	0.8826	0.9368	1.09	0.2867	0.9889	1.02	0.7852	0.9881
MICOS complex subunit MIC27	MIC27_HUMAN	Q6UXV4	1.00	0.9371	0.9649	1.08	0.2219	0.9982	1.06	0.3684	0.9960	1.00	0.9555	0.9939	1.03	0.6204	0.9951	0.92	0.2773	0.9856	1.00	0.9629	0.9924	0.91	0.1338	0.3740	1.04	0.5728	0.9889	1.02	0.7008	0.9881
MICOS complex subunit MIC60	MIC60_HUMAN	Q16891	0.97	0.6527	0.8297	0.97	0.7083	0.9982	1.02	0.8196	0.9960	0.98	0.7871	0.9939	1.03	0.6834	0.9951	0.95	0.5332	0.9856	0.97	0.6906	0.9047	1.03	0.7441	0.8595	1.10	0.1885	0.9889	1.06	0.4246	0.9607
Microfibril-associated glycoprotein 4	MFAP4_HUMAN	P55083	0.92	0.6503	0.8297	0.97	0.8810	0.9982	0.88	0.5623	0.9960	0.65	0.0213	0.9874	0.76	0.1640	0.9951	1.24	0.3631	0.9856	0.94	0.7550	0.9282	0.77	0.2146	0.4815	1.16	0.4385	0.9889	1.20	0.3407	0.9607
Microfibrillar-associated protein 5	MFAP5_HUMAN	Q13361	1.00	0.9767	0.9869	0.81	0.1811	0.9982	1.03	0.8625	0.9960	0.92	0.5851	0.9939	1.12	0.4811	0.9951	1.02	0.9087	0.9959	1.24	0.1871	0.7149	0.86	0.3819	0.6123	0.98	0.8918	0.9889	1.14	0.3908	0.9607
Microsomal glutathione S-transferase 3	MGST3_HUMAN	O14880	0.92	0.4719	0.7106	0.93	0.5442	0.9982	1.24	0.1281	0.9960	1.03	0.7828	0.9939	0.93	0.5790	0.9951	0.76	0.0691	0.9641	0.76	0.0277	0.6824	1.27	0.0766	0.2846	0.85	0.1800	0.9889	0.94	0.5866	0.9881
Microtubule-associated protein 1B	MAP1B_HUMAN	P46821	1.03	0.6839	0.8479	1.03	0.7179	0.9982	1.06	0.4748	0.9960	1.05	0.4762	0.9939	0.93	0.3533	0.9951	0.93	0.4570	0.9856	1.03	0.6927	0.9047	0.95	0.5320	0.7227	0.99	0.8568	0.9889	1.00	0.9899	0.9992
Microtubule-associated protein 4	MAP4_HUMAN	P27816	0.94	0.4061	0.6612	0.93	0.3086	0.9982	1.03	0.6957	0.9960	1.03	0.6773	0.9939	0.92	0.2616	0.9951	1.06	0.5508	0.9856	0.84	0.0229	0.6824	1.25	0.0063	0.1323	0.99	0.8809	0.9889	0.89	0.1139	0.9607
Microtubule-associated protein RP/EB family member 1	MARE1_HUMAN	Q15691	1.02	0.7396	0.8798	0.99	0.8357	0.9982	1.03	0.7352	0.9960	1.00	0.9465	0.9939	0.96	0.6163	0.9951	1.09	0.3144	0.9856	1.02	0.7705	0.9300	1.10	0.2294	0.4943	0.98	0.8101	0.9889	0.96	0.5209	0.9846
Microtubule-associated protein RP/EB family member 2	MARE2_HUMAN	Q15555	0.91	0.3240	0.5917	0.88	0.1873	0.9982	1.16	0.1695	0.9960	1.03	0.7557	0.9939	0.87	0.1696	0.9951	0.93	0.5517	0.9856	0.86	0.1494	0.6871	1.45	0.0003	0.0621	1.09	0.3723	0.9889	0.91	0.2978	0.9607
Microtubule-associated protein tau	TAU_HUMAN	P10636	1.20	0.2271	0.5010	0.88	0.3983	0.9982	0.80	0.1962	0.9960	1.19	0.2513	0.9939	0.98	0.9032	0.9951	1.22	0.2826	0.9856	1.10	0.5565	0.8601	1.18	0.3126	0.5563	1.10	0.5451	0.9889	0.85	0.2938	0.9607
Midasin	MDN1_HUMAN	Q9NU22	1.04	0.8853	0.9400	1.69	0.0711	0.9982	0.93	0.8369	0.9960	0.97	0.9111	0.9939	0.60	0.0912	0.9951	0.80	0.5378	0.9856	1.06	0.8623	0.9638	0.77	0.4174	0.6425	0.71	0.2516	0.9889	0.69	0.1977	0.9607
Mimecan	MIME_HUMAN	P20774	0.63	0.0234	0.2873	0.90	0.6299	0.9982	0.78	0.3034	0.9960	1.05	0.8144	0.9939	0.75	0.1774	0.9951	1.27	0.3629	0.9856	1.36	0.1663	0.6906	0.61	0.0340	0.2017	1.08	0.7069	0.9889	0.86	0.4651	0.9607
Mitochondrial 2-oxoglutarate/malate carrier protein	M2OM_HUMAN	Q02978	1.07	0.4064	0.6612	1.06	0.4843	0.9982	1.00	0.9911	0.9960	1.01	0.9128	0.9939	1.02	0.8481	0.9951	0.97	0.7614	0.9857	0.97	0.6847	0.9004	1.03	0.7100	0.8360	1.06	0.4570	0.9889	1.00	0.9794	0.9992
Mitochondrial calcium uniporter regulator 1	MCUR1_HUMAN	Q96AQ8	1.02	0.7395	0.8798	1.10	0.1646	0.9982	0.95	0.5425	0.9960	1.04	0.5792	0.9939	1.01	0.8299	0.9951	1.01	0.9380	0.9959	1.04	0.5501	0.8601	1.10	0.2059	0.4687	1.08	0.2442	0.9889	1.06	0.4007	0.9607
Mitochondrial carnitine/acylcarnitine carrier protein	MCAT_HUMAN	O43772	1.11	0.2053	0.4823	1.13	0.1368	0.9982	0.97	0.7274	0.9960	1.07	0.3914	0.9939	1.06	0.5314	0.9951	1.04	0.6918	0.9856	1.06	0.5433	0.8580	1.07	0.4715	0.6780	1.04	0.6358	0.9889	1.06	0.5101	0.9846
Mitochondrial fission 1 protein	FIS1_HUMAN	Q9Y3D6	0.94	0.2830	0.5570	1.06	0.3419	0.9982	1.01	0.8262	0.9960	0.95	0.3851	0.9939	0.92	0.1478	0.9951	0.93	0.3000	0.9856	0.93	0.2439	0.7187	1.05	0.4629	0.6748	1.03	0.5615	0.9889	0.99	0.8480	0.9881
Mitochondrial fission factor	MFF_HUMAN	Q9GZY8	1.08	0.2841	0.5578	1.06	0.4247	0.9982	0.85	0.0487	0.9960	1.08	0.2599	0.9939	1.10	0.1809	0.9951	1.12	0.1822	0.9856	0.96	0.5649	0.8601	1.04	0.5914	0.7646	1.04	0.6320	0.9889	1.11	0.1399	0.9607
Mitochondrial import inner membrane translocase subunit Tim10	TIM10_HUMAN	P62072	1.14	0.0432	0.2873	1.02	0.7327	0.9982	1.11	0.1707	0.9960	0.91	0.1444	0.9874	0.93	0.2553	0.9951	0.87	0.0928	0.9856	0.85	0.0174	0.6779	1.05	0.4961	0.6959	1.03	0.6303	0.9889	0.99	0.9009	0.9938
Mitochondrial import inner membrane translocase subunit Tim13	TIM13_HUMAN	Q9Y5L4	1.00	0.9577	0.9734	0.94	0.2126	0.9982	0.99	0.8985	0.9960	0.97	0.5064	0.9939	0.90	0.0531	0.9951	1.04	0.5814	0.9856	0.94	0.2529	0.7187	1.25	<0.0001	0.0597	0.98	0.7391	0.9889	0.91	0.0702	0.9607
Mitochondrial import inner membrane translocase subunit Tim21	TIM21_HUMAN	Q9BVV7	1.16	0.0672	0.3105	1.09	0.3169	0.9982	0.95	0.6239	0.9960	1.04	0.6137	0.9939	1.16	0.0832	0.9951	1.00	0.9984	0.9995	0.94	0.4917	0.8413	0.94	0.5157	0.7105	1.02	0.7913	0.9889	1.13	0.1267	0.9607
Mitochondrial import inner membrane translocase subunit TIM44	TIM44_HUMAN	O43615	1.08	0.1049	0.3654	0.99	0.8428	0.9982	0.95	0.3139	0.9960	1.00	0.9698	0.9961	1.06	0.2538	0.9951	1.02	0.7535	0.9857	1.00	0.9700	0.9937	1.08	0.1698	0.4217	1.04	0.4671	0.9889	1.06	0.2372	0.9607
Mitochondrial import inner membrane translocase subunit TIM50	TIM50_HUMAN	Q3ZCQ8	1.07	0.3007	0.5713	1.02	0.7089	0.9982	1.01	0.9099	0.9960	1.01	0.8957	0.9939	1.09	0.1749	0.9951	0.96	0.5776	0.9856	1.03	0.6265	0.8830	0.98	0.7730	0.8789	1.06	0.3769	0.9889	1.08	0.2181	0.9607
Mitochondrial import inner membrane translocase subunit Tim8 A	TIM8A_HUMAN	O60220	1.16	0.0188	0.2873	0.99	0.9243	0.9982	1.00	0.9858	0.9960	0.97	0.6484	0.9939	0.95	0.3951	0.9951	0.97	0.6917	0.9856	0.92	0.2412	0.7187	1.20	0.0094	0.1378	1.03	0.6816	0.9889	0.95	0.3976	0.9607
Mitochondrial import inner membrane translocase subunit Tim8 B	TIM8B_HUMAN	Q9Y5J9	1.11	0.1806	0.4557	0.91	0.2380	0.9982	0.94	0.4606	0.9960	0.92	0.2411	0.9939	0.94	0.4004	0.9951	1.06	0.5076	0.9856	0.96	0.6414	0.8892	1.32	0.0007	0.0732	0.94	0.4087	0.9889	0.92	0.2584	0.9607
Mitochondrial import inner membrane translocase subunit Tim9	TIM9_HUMAN	Q9Y5J7	1.02	0.7087	0.8648	0.94	0.2736	0.9982	1.16	0.0203	0.9960	0.93	0.1762	0.9907	0.95	0.4160	0.9951	0.89	0.0789	0.9856	0.94	0.3022	0.7361	1.01	0.8532	0.9202	0.92	0.1725	0.9889	0.98	0.7104	0.9881
Mitochondrial import receptor subunit TOM70	TOM70_HUMAN	O94826	0.98	0.5784	0.7764	0.98	0.7067	0.9982	1.02	0.7205	0.9960	1.04	0.4334	0.9939	1.02	0.6028	0.9951	0.92	0.1483	0.9856	1.00	0.9892	0.9992	0.94	0.2121	0.4786	0.97	0.5744	0.9889	1.02	0.6804	0.9881
Mitochondrial intermediate peptidase	MIPEP_HUMAN	Q99797	1.17	0.0470	0.2898	1.08	0.3501	0.9982	0.93	0.4314	0.9960	1.04	0.5939	0.9939	1.05	0.5109	0.9951	1.10	0.3357	0.9856	0.89	0.1447	0.6871	1.10	0.2685	0.5288	1.06	0.4384	0.9889	1.08	0.3029	0.9607
Mitochondrial intermembrane space import and assembly protein 40	MIA40_HUMAN	Q8N4Q1	1.06	0.4813	0.7173	0.94	0.4670	0.9982	1.03	0.7462	0.9960	0.94	0.4905	0.9939	0.99	0.8884	0.9951	0.96	0.7411	0.9857	0.93	0.4668	0.8278	1.21	0.0533	0.2446	1.04	0.6445	0.9889	1.02	0.8529	0.9881
Mitochondrial peptide methionine sulfoxide reductase	MSRA_HUMAN	Q9UJ68	1.15	0.0426	0.2873	0.99	0.9133	0.9982	0.96	0.5844	0.9960	1.01	0.9056	0.9939	1.01	0.8755	0.9951	1.05	0.6037	0.9856	0.93	0.3202	0.7400	1.11	0.1658	0.4176	1.01	0.8858	0.9889	1.00	0.9602	0.9947
Mitochondrial potassium channel	MITOK_HUMAN	Q96ER9	0.97	0.5907	0.7870	1.07	0.2787	0.9982	0.95	0.4648	0.9960	1.03	0.6403	0.9939	0.97	0.6861	0.9951	1.07	0.4102	0.9856	1.12	0.0914	0.6824	1.03	0.6717	0.8133	1.15	0.0302	0.9889	1.03	0.5916	0.9881
Mitochondrial proton/calcium exchanger protein	LETM1_HUMAN	O95202	1.07	0.2918	0.5638	1.00	0.9665	0.9982	0.94	0.3446	0.9960	1.03	0.6095	0.9939	1.06	0.3483	0.9951	1.01	0.8662	0.9959	0.93	0.2848	0.7212	1.03	0.6298	0.7896	1.06	0.3559	0.9889	1.06	0.3017	0.9607
Mitochondrial pyruvate carrier 1	MPC1_HUMAN	Q9Y5U8	1.02	0.7620	0.8882	1.03	0.7171	0.9982	1.11	0.1912	0.9960	1.03	0.6927	0.9939	1.02	0.7504	0.9951	0.86	0.0665	0.9641	1.02	0.8233	0.9481	1.11	0.1699	0.4217	0.97	0.6254	0.9889	1.05	0.4848	0.9652
Mitochondrial-processing peptidase subunit alpha	MPPA_HUMAN	Q10713	1.05	0.3633	0.6264	0.97	0.6055	0.9982	1.04	0.4912	0.9960	0.96	0.3956	0.9939	0.96	0.4165	0.9951	0.96	0.5483	0.9856	0.97	0.5977	0.8646	1.06	0.3012	0.5492	1.00	0.9665	0.9964	0.98	0.7575	0.9881
Mitochondrial-processing peptidase subunit beta	MPPB_HUMAN	O75439	1.05	0.3637	0.6264	0.97	0.5830	0.9982	0.96	0.5154	0.9960	0.97	0.5997	0.9939	0.98	0.7573	0.9951	1.03	0.6071	0.9856	0.95	0.3139	0.7400	1.12	0.0482	0.2317	1.04	0.4847	0.9889	1.00	0.9595	0.9946
Mitofusin-2	MFN2_HUMAN	O95140	1.03	0.6476	0.8293	1.00	0.9535	0.9982	1.00	0.9546	0.9960	1.08	0.1645	0.9874	1.09	0.1466	0.9951	0.94	0.3914	0.9856	1.03	0.6541	0.8915	1.02	0.7692	0.8762	1.04	0.5284	0.9889	1.07	0.2199	0.9607
Mitogen-activated protein kinase 1	MK01_HUMAN	P28482	0.90	0.0729	0.3242	1.01	0.8270	0.9982	1.02	0.8005	0.9960	1.01	0.8694	0.9939	0.94	0.3460	0.9951	0.98	0.7909	0.9903	1.03	0.6039	0.8682	0.92	0.1958	0.4564	1.00	0.9722	0.9976	0.95	0.4371	0.9607
Mitogen-activated protein kinase kinase kinase 20	M3K20_HUMAN	Q9NYL2	1.03	0.7951	0.9014	0.96	0.6862	0.9982	1.12	0.3395	0.9960	0.81	0.0289	0.9874	0.87	0.1659	0.9951	0.95	0.6642	0.9856	0.84	0.1036	0.6824	1.49	0.0002	0.0621	0.94	0.5633	0.9889	0.90	0.2883	0.9607
Moesin	MOES_HUMAN	P26038	0.88	0.0431	0.2873	0.96	0.5301	0.9982	1.02	0.7447	0.9960	0.93	0.2589	0.9939	0.86	0.0221	0.9951	1.02	0.8210	0.9959	1.02	0.7844	0.9373	0.99	0.9011	0.9448	0.96	0.5154	0.9889	0.88	0.0461	0.9607
Monoglyceride lipase	MGLL_HUMAN	Q99685	1.11	0.2657	0.5347	0.99	0.8902	0.9982	1.11	0.3299	0.9960	1.08	0.3957	0.9939	1.21	0.0512	0.9951	0.87	0.2210	0.9856	1.16	0.1433	0.6871	1.03	0.8153	0.8997	0.93	0.4837	0.9889	1.12	0.2345	0.9607
Mth938 domain-containing protein	AAMDC_HUMAN	Q9H7C9	1.20	0.0498	0.2898	1.06	0.5559	0.9982	1.03	0.7779	0.9960	0.87	0.1333	0.9874	0.91	0.3366	0.9951	1.04	0.7654	0.9857	0.85	0.1017	0.6824	1.08	0.4475	0.6635	0.99	0.9579	0.9961	0.98	0.8508	0.9881
Multifunctional procollagen lysine hydroxylase and glycosyltransferase LH	PLOD3_HUMAN	O60568	1.07	0.5159	0.7330	1.15	0.2035	0.9982	0.96	0.7649	0.9960	1.04	0.7435	0.9939	1.11	0.3672	0.9951	1.13	0.3547	0.9856	1.04	0.7337	0.9218	0.86	0.2278	0.4943	0.97	0.7697	0.9889	1.09	0.4430	0.9607
Multimerin-2	MMRN2_HUMAN	Q9H8L6	0.96	0.5444	0.7505	0.94	0.3876	0.9982	0.96	0.6792	0.9960	0.93	0.2990	0.9939	1.01	0.8694	0.9951	1.01	0.9123	0.9959	0.98	0.8189	0.9481	0.97	0.7625	0.8704	0.92	0.2881	0.9889	1.02	0.7986	0.9881
Muscular LMNA-interacting protein	MLIP_HUMAN	Q5VWP3	1.00	0.9721	0.9848	1.00	0.9966	0.9985	0.89	0.1500	0.9960	0.98	0.7897	0.9939	0.98	0.8274	0.9951	1.19	0.0385	0.9411	0.90	0.1488	0.6871	1.08	0.3350	0.5707	1.04	0.5419	0.9889	0.99	0.9026	0.9938
Mycophenolic acid acyl-glucuronide esterase, mitochondrial	ABHDA_HUMAN	Q9NUJ1	1.15	0.1018	0.3619	1.07	0.4339	0.9982	0.97	0.7950	0.9960	0.91	0.2531	0.9939	0.94	0.4930	0.9951	1.05	0.6634	0.9856	0.91	0.3106	0.7378	1.20	0.0507	0.2394	1.05	0.5792	0.9889	0.99	0.8666	0.9907
Myeloid-derived growth factor	MYDGF_HUMAN	Q969H8	0.86	0.0421	0.2873	1.01	0.9350	0.9982	1.13	0.1508	0.9960	1.04	0.6158	0.9939	0.88	0.1113	0.9951	0.92	0.3646	0.9856	0.98	0.8409	0.9538	1.05	0.5654	0.7461	0.89	0.1136	0.9889	0.88	0.0740	0.9607
Myoglobin	MYG_HUMAN	P02144	1.16	0.1518	0.4208	0.99	0.9539	0.9982	0.91	0.4467	0.9960	0.85	0.1163	0.9874	0.95	0.5995	0.9951	1.19	0.1712	0.9856	0.87	0.2000	0.7149	1.20	0.1097	0.3362	0.97	0.8092	0.9889	1.03	0.7674	0.9881
Myomesin-1	MYOM1_HUMAN	P52179	1.13	0.1573	0.4259	1.00	0.9704	0.9982	1.11	0.2731	0.9960	0.98	0.8583	0.9939	1.01	0.9511	0.9951	0.87	0.1818	0.9856	0.84	0.0478	0.6824	1.19	0.0678	0.2662	1.15	0.1167	0.9889	1.10	0.2594	0.9607
Myomesin-2	MYOM2_HUMAN	P54296	1.12	0.1686	0.4389	0.97	0.7224	0.9982	1.00	0.9894	0.9960	0.96	0.6148	0.9939	1.00	0.9995	0.9996	1.00	0.9671	0.9967	0.86	0.0907	0.6824	1.26	0.0106	0.1462	1.09	0.3167	0.9889	1.07	0.4320	0.9607
Myomesin-3	MYOM3_HUMAN	Q5VTT5	0.96	0.6278	0.8160	0.97	0.6534	0.9982	1.01	0.9038	0.9960	1.01	0.9266	0.9939	0.98	0.7724	0.9951	0.95	0.6121	0.9856	0.91	0.2144	0.7171	1.17	0.0540	0.2446	1.08	0.2931	0.9889	1.03	0.6816	0.9881
Myopalladin	MYPN_HUMAN	Q86TC9	1.08	0.4520	0.6961	1.01	0.9265	0.9982	0.99	0.9325	0.9960	0.99	0.9297	0.9939	0.99	0.8851	0.9951	1.01	0.9354	0.9959	0.74	0.0034	0.6779	1.24	0.0540	0.2446	1.13	0.2178	0.9889	1.10	0.3168	0.9607
Myosin light chain 1/3, skeletal muscle isoform	MYL1_HUMAN	P05976	1.10	0.4159	0.6668	0.90	0.3307	0.9982	1.12	0.3725	0.9960	1.17	0.1528	0.9874	1.05	0.6973	0.9951	0.86	0.2584	0.9856	0.92	0.4998	0.8476	1.34	0.0180	0.1645	1.01	0.9400	0.9922	1.04	0.7296	0.9881
Myosin light chain 3	MYL3_HUMAN	P08590	1.03	0.7267	0.8738	0.94	0.3797	0.9982	0.99	0.8809	0.9960	1.13	0.1144	0.9874	1.07	0.3882	0.9951	0.97	0.7254	0.9857	0.93	0.3629	0.7668	1.12	0.1646	0.4153	1.03	0.7429	0.9889	1.06	0.4186	0.9607
Myosin light chain 4	MYL4_HUMAN	P12829	1.07	0.7692	0.8891	1.09	0.7138	0.9982	0.71	0.1929	0.9960	1.11	0.6451	0.9939	1.32	0.2483	0.9951	1.37	0.2686	0.9856	0.99	0.9635	0.9924	0.94	0.8115	0.8963	1.90	0.0057	0.9889	1.49	0.0835	0.9607
Myosin light chain 6B	MYL6B_HUMAN	P14649	0.85	0.1411	0.4031	0.87	0.2042	0.9982	0.99	0.9631	0.9960	1.18	0.1135	0.9874	1.03	0.7739	0.9951	0.96	0.7434	0.9857	0.96	0.7264	0.9205	1.03	0.8116	0.8963	1.05	0.6909	0.9889	1.00	0.9969	0.9999
Myosin light chain kinase 3	MYLK3_HUMAN	Q32MK0	1.16	0.0762	0.3266	1.18	0.0433	0.9982	0.96	0.6612	0.9960	1.09	0.3016	0.9939	1.11	0.2385	0.9951	1.07	0.5037	0.9856	1.00	0.9748	0.9939	0.96	0.6904	0.8252	1.21	0.0279	0.9889	1.17	0.0652	0.9607
Myosin light polypeptide 6	MYL6_HUMAN	P60660	0.73	0.0066	0.2487	0.98	0.8499	0.9982	0.90	0.4287	0.9960	0.87	0.2358	0.9939	0.80	0.0659	0.9951	1.15	0.3403	0.9856	1.00	0.9922	0.9992	0.79	0.0679	0.2662	0.95	0.6425	0.9889	0.91	0.4307	0.9607
Myosin regulatory light chain 12B	ML12B_HUMAN	O14950	0.79	0.0044	0.2034	0.91	0.2819	0.9982	1.04	0.6982	0.9960	0.93	0.4129	0.9939	0.80	0.0095	0.9951	0.96	0.6662	0.9856	0.92	0.3557	0.7644	1.12	0.2166	0.4826	1.00	0.9720	0.9976	0.86	0.0812	0.9607
Myosin regulatory light chain 2, atrial isoform	MLRA_HUMAN	Q01449	1.10	0.5866	0.7845	0.74	0.0798	0.9982	0.98	0.9343	0.9960	0.90	0.5354	0.9939	0.86	0.4217	0.9951	1.16	0.4939	0.9856	0.65	0.0214	0.6804	1.85	0.0012	0.0763	0.93	0.7091	0.9889	0.85	0.3393	0.9607
Myosin regulatory light chain 2, ventricular/cardiac muscle isoform	MLRV_HUMAN	P10916	1.08	0.3753	0.6324	0.97	0.7221	0.9982	1.00	0.9629	0.9960	1.09	0.2916	0.9939	1.02	0.8588	0.9951	0.98	0.8144	0.9959	0.90	0.2407	0.7187	1.18	0.0689	0.2689	1.03	0.7486	0.9889	1.05	0.5534	0.9881
Myosin regulatory light polypeptide 9	MYL9_HUMAN	P24844	0.84	0.0608	0.3030	1.07	0.4896	0.9982	0.97	0.7698	0.9960	0.93	0.4361	0.9939	0.85	0.0765	0.9951	1.07	0.5608	0.9856	0.98	0.8443	0.9558	0.84	0.0880	0.2995	0.97	0.7163	0.9889	0.95	0.5584	0.9881
Myosin-10	MYH10_HUMAN	P35580	0.72	0.0670	0.3105	1.27	0.1834	0.9982	0.86	0.4866	0.9960	0.90	0.5746	0.9939	0.83	0.3081	0.9951	1.19	0.4424	0.9856	1.28	0.2025	0.7149	0.69	0.0665	0.2648	1.10	0.6234	0.9889	0.91	0.5895	0.9881
Myosin-11	MYH11_HUMAN	P35749	0.64	0.0043	0.2034	1.06	0.7086	0.9982	0.95	0.7994	0.9960	0.66	0.0096	0.9874	0.61	0.0030	0.9389	1.11	0.6159	0.9856	0.90	0.5263	0.8558	0.93	0.6955	0.8265	0.98	0.9231	0.9889	0.84	0.2717	0.9607
Myosin-7	MYH7_HUMAN	P12883	0.85	0.0485	0.2898	0.97	0.6840	0.9982	0.91	0.3315	0.9960	0.93	0.3958	0.9939	0.91	0.2803	0.9951	1.11	0.3357	0.9856	0.98	0.8151	0.9478	0.99	0.9203	0.9569	1.10	0.2949	0.9889	0.97	0.7371	0.9881
Myosin-9	MYH9_HUMAN	P35579	0.64	0.0026	0.2005	1.01	0.9392	0.9982	0.96	0.8304	0.9960	0.82	0.1947	0.9939	0.74	0.0544	0.9951	1.07	0.7386	0.9857	1.23	0.2101	0.7157	0.74	0.0805	0.2899	0.93	0.6580	0.9889	0.82	0.2066	0.9607
Myosin-binding protein C, cardiac-type	MYPC3_HUMAN	Q14896	1.10	0.2504	0.5219	1.01	0.9069	0.9982	1.06	0.5403	0.9960	1.07	0.4187	0.9939	0.98	0.8420	0.9951	0.90	0.2719	0.9856	0.90	0.1984	0.7149	1.27	0.0065	0.1326	1.07	0.4450	0.9889	1.07	0.4339	0.9607
Myozenin-2	MYOZ2_HUMAN	Q9NPC6	1.11	0.1200	0.3810	1.00	0.9923	0.9985	0.90	0.1850	0.9960	1.15	0.0407	0.9874	1.14	0.0556	0.9951	1.01	0.8672	0.9959	1.10	0.1980	0.7149	1.06	0.4607	0.6738	1.05	0.5261	0.9889	1.16	0.0250	0.9607
N(G),N(G)-dimethylarginine dimethylaminohydrolase 1	DDAH1_HUMAN	O94760	0.93	0.4407	0.6862	0.90	0.2498	0.9982	1.08	0.4921	0.9960	1.05	0.6259	0.9939	0.98	0.8616	0.9951	1.08	0.5243	0.9856	0.91	0.3279	0.7411	1.18	0.1097	0.3362	0.91	0.3087	0.9889	0.91	0.3106	0.9607
N(G),N(G)-dimethylarginine dimethylaminohydrolase 2	DDAH2_HUMAN	O95865	0.92	0.2618	0.5327	0.92	0.2769	0.9982	1.02	0.8565	0.9960	1.00	0.9787	0.9961	0.97	0.6943	0.9951	1.09	0.3608	0.9856	1.06	0.4386	0.8116	1.05	0.5592	0.7404	0.93	0.3346	0.9889	0.92	0.2420	0.9607
Na(+)/H(+) exchange regulatory cofactor NHE-RF2	NHRF2_HUMAN	Q15599	1.03	0.5909	0.7870	1.01	0.9136	0.9982	1.03	0.6532	0.9960	1.00	0.9679	0.9961	0.99	0.7862	0.9951	0.97	0.5984	0.9856	1.02	0.7694	0.9298	1.00	0.9619	0.9782	1.06	0.2222	0.9889	1.02	0.6482	0.9881
NAD kinase 2, mitochondrial	NAKD2_HUMAN	Q4G0N4	0.91	0.1515	0.4208	0.96	0.5232	0.9982	0.98	0.8382	0.9960	1.13	0.0637	0.9874	0.99	0.8952	0.9951	0.99	0.8900	0.9959	1.01	0.8608	0.9636	1.03	0.6893	0.8252	1.00	0.9588	0.9961	0.98	0.7435	0.9881
NAD-dependent malic enzyme, mitochondrial	MAOM_HUMAN	P23368	1.04	0.5313	0.7434	0.95	0.5074	0.9982	0.98	0.7827	0.9960	0.97	0.6830	0.9939	0.94	0.4035	0.9951	1.04	0.6164	0.9856	0.97	0.6981	0.9081	1.15	0.0761	0.2841	0.97	0.6184	0.9889	0.87	0.0487	0.9607
NAD-dependent protein deacetylase sirtuin-3, mitochondrial	SIR3_HUMAN	Q9NTG7	1.18	0.1103	0.3718	1.00	0.9931	0.9985	0.85	0.1798	0.9960	0.97	0.7905	0.9939	0.97	0.7964	0.9951	1.23	0.0979	0.9856	0.90	0.3369	0.7460	1.27	0.0326	0.2017	1.08	0.4656	0.9889	1.05	0.6221	0.9881
NAD-dependent protein deacylase sirtuin-5, mitochondrial	SIR5_HUMAN	Q9NXA8	1.27	0.0029	0.2005	1.00	0.9564	0.9982	0.88	0.1916	0.9960	1.04	0.6313	0.9939	1.16	0.0717	0.9951	1.19	0.0801	0.9856	1.04	0.6508	0.8905	1.11	0.2587	0.5237	1.06	0.5002	0.9889	1.15	0.0847	0.9607
NAD(P) transhydrogenase, mitochondrial	NNTM_HUMAN	Q13423	1.05	0.4741	0.7121	1.04	0.6265	0.9982	1.00	0.9885	0.9960	1.03	0.6665	0.9939	1.06	0.4738	0.9951	0.97	0.7637	0.9857	0.91	0.2487	0.7187	1.12	0.1620	0.4114	1.08	0.3054	0.9889	1.07	0.3650	0.9607
NAD(P)H-hydrate epimerase	NNRE_HUMAN	Q8NCW5	1.20	0.0501	0.2898	1.06	0.5119	0.9982	1.00	0.9848	0.9960	0.90	0.2381	0.9939	0.95	0.6220	0.9951	1.02	0.8706	0.9959	0.83	0.0491	0.6824	1.12	0.2649	0.5268	0.94	0.5136	0.9889	0.98	0.8296	0.9881
NADH dehydrogenase [ubiquinone] 1 alpha subcomplex assembly factor 2	NDUF2_HUMAN	Q8N183	0.97	0.6023	0.7965	1.07	0.2100	0.9982	0.98	0.7182	0.9960	1.03	0.5600	0.9939	0.99	0.8778	0.9951	0.96	0.5252	0.9856	1.08	0.1584	0.6871	0.96	0.5391	0.7261	1.03	0.6081	0.9889	1.03	0.6308	0.9881
NADH dehydrogenase [ubiquinone] 1 alpha subcomplex assembly factor 3	NDUF3_HUMAN	Q9BU61	1.18	0.0160	0.2873	1.09	0.2339	0.9982	1.02	0.7827	0.9960	1.08	0.2798	0.9939	1.11	0.1427	0.9951	0.96	0.6006	0.9856	0.95	0.4680	0.8280	1.03	0.7399	0.8574	1.01	0.8768	0.9889	1.10	0.1592	0.9607
NADH dehydrogenase [ubiquinone] 1 alpha subcomplex assembly factor 4	NDUF4_HUMAN	Q9P032	0.98	0.7324	0.8760	1.03	0.6934	0.9982	0.98	0.8154	0.9960	0.98	0.7226	0.9939	0.96	0.6147	0.9951	1.01	0.8721	0.9959	0.92	0.2424	0.7187	1.09	0.2730	0.5288	1.03	0.6652	0.9889	1.03	0.6922	0.9881
NADH dehydrogenase [ubiquinone] 1 alpha subcomplex assembly factor 8	NDUF8_HUMAN	A1L188	1.10	0.1974	0.4751	1.02	0.8397	0.9982	0.90	0.2236	0.9960	0.97	0.6936	0.9939	0.97	0.7299	0.9951	1.14	0.1700	0.9856	0.82	0.0128	0.6779	1.19	0.0414	0.2169	1.04	0.5857	0.9889	1.03	0.7254	0.9881
NADH dehydrogenase [ubiquinone] 1 alpha subcomplex subunit 10, mito	NDUAA_HUMAN	O95299	1.16	0.0625	0.3051	1.12	0.1727	0.9982	0.93	0.4612	0.9960	0.98	0.7788	0.9939	1.08	0.3533	0.9951	1.02	0.8700	0.9959	0.89	0.1915	0.7149	1.05	0.5856	0.7618	1.07	0.3830	0.9889	1.12	0.1500	0.9607
NADH dehydrogenase [ubiquinone] 1 alpha subcomplex subunit 12	NDUAC_HUMAN	Q9UI09	1.15	0.0711	0.3218	1.08	0.3405	0.9982	0.92	0.3446	0.9960	0.97	0.6651	0.9939	1.09	0.2826	0.9951	1.03	0.7385	0.9857	0.99	0.9062	0.9840	1.04	0.6767	0.8186	1.13	0.1202	0.9889	1.16	0.0507	0.9607
NADH dehydrogenase [ubiquinone] 1 alpha subcomplex subunit 13	NDUAD_HUMAN	Q9P0J0	1.17	0.0412	0.2873	1.13	0.1020	0.9982	0.90	0.2167	0.9960	1.01	0.9455	0.9939	1.10	0.2156	0.9951	1.05	0.5807	0.9856	0.96	0.6172	0.8752	1.04	0.6224	0.7879	1.11	0.1750	0.9889	1.18	0.0314	0.9607
NADH dehydrogenase [ubiquinone] 1 alpha subcomplex subunit 2	NDUA2_HUMAN	O43678	1.12	0.1717	0.4464	1.00	0.9877	0.9985	0.95	0.5655	0.9960	0.88	0.1193	0.9874	1.00	0.9625	0.9951	1.00	0.9989	0.9995	0.92	0.3439	0.7576	1.14	0.1450	0.3898	1.17	0.0699	0.9889	1.11	0.2024	0.9607
NADH dehydrogenase [ubiquinone] 1 alpha subcomplex subunit 3	NDUA3_HUMAN	O95167	1.09	0.3559	0.6186	1.05	0.6075	0.9982	0.91	0.3378	0.9960	0.89	0.1973	0.9939	0.93	0.4550	0.9951	1.06	0.6066	0.9856	0.89	0.2370	0.7187	1.21	0.0570	0.2478	1.07	0.4813	0.9889	1.05	0.6172	0.9881
NADH dehydrogenase [ubiquinone] 1 alpha subcomplex subunit 5	NDUA5_HUMAN	Q16718	1.10	0.2438	0.5179	1.01	0.9127	0.9982	0.94	0.5426	0.9960	0.90	0.2232	0.9939	1.01	0.9452	0.9951	1.02	0.8295	0.9959	0.89	0.2096	0.7156	1.17	0.0941	0.3118	1.17	0.0738	0.9889	1.12	0.1792	0.9607
NADH dehydrogenase [ubiquinone] 1 alpha subcomplex subunit 6	NDUA6_HUMAN	P56556	1.08	0.3995	0.6575	0.84	0.0633	0.9982	1.07	0.5495	0.9960	0.84	0.0678	0.9874	1.00	0.9866	0.9964	0.99	0.9334	0.9959	1.03	0.7697	0.9298	1.07	0.5385	0.7261	0.98	0.8231	0.9889	1.11	0.2779	0.9607
NADH dehydrogenase [ubiquinone] 1 alpha subcomplex subunit 7	NDUA7_HUMAN	O95182	1.04	0.5210	0.7363	0.99	0.9284	0.9982	0.93	0.3128	0.9960	0.98	0.6974	0.9939	1.04	0.5348	0.9951	1.02	0.8285	0.9959	0.96	0.5881	0.8645	1.03	0.6476	0.7998	1.06	0.4131	0.9889	1.07	0.2800	0.9607
NADH dehydrogenase [ubiquinone] 1 alpha subcomplex subunit 8	NDUA8_HUMAN	P51970	1.04	0.5853	0.7836	1.03	0.7073	0.9982	0.92	0.3749	0.9960	0.91	0.2207	0.9939	0.95	0.5032	0.9951	1.03	0.7521	0.9857	0.85	0.0556	0.6824	1.13	0.1739	0.4257	1.13	0.1130	0.9889	1.07	0.4038	0.9607
NADH dehydrogenase [ubiquinone] 1 alpha subcomplex subunit 9, mitocl	NDUA9_HUMAN	Q16795	1.18	0.0415	0.2873	1.00	0.9572	0.9982	0.94	0.5306	0.9960	0.97	0.7035	0.9939	1.13	0.1532	0.9951	1.03	0.7653	0.9857	0.98	0.8369	0.9532	1.06	0.5117	0.7066	1.07	0.4383	0.9889	1.16	0.0687	0.9607
NADH dehydrogenase [ubiquinone] 1 beta subcomplex subunit 10	NDUBA_HUMAN	O96000	0.98	0.8572	0.9307	1.03	0.7215	0.9982	0.93	0.4509	0.9960	0.86	0.0826	0.9874	0.93	0.4630	0.9951	1.06	0.5683	0.9856	0.96	0.6473	0.8897	1.12	0.2641	0.5265	1.11	0.2617	0.9889	1.03	0.7076	0.9881
NADH dehydrogenase [ubiquinone] 1 beta subcomplex subunit 11, mitoc	NDUBB_HUMAN	Q9NX14	0.91	0.2370	0.5105	1.03	0.7580	0.9982	0.84	0.0595	0.9960	1.00	0.9927	0.9985	0.91	0.2709	0.9951	1.19	0.0833	0.9856	1.01	0.9517	0.9923	1.10	0.2897	0.5428	1.08	0.3644	0.9889	1.00	0.9675	0.9985
NADH dehydrogenase [ubiquinone] 1 beta subcomplex subunit 3	NDUB3_HUMAN	O43676	0.93	0.3447	0.6081	0.97	0.7152	0.9982	0.85	0.0928	0.9960	0.85	0.0511	0.9874	0.91	0.2561	0.9951	1.17	0.1225	0.9856	0.89	0.1855	0.7149	1.04	0.6636	0.8089	1.08	0.3670	0.9889	1.01	0.9073	0.9938
NADH dehydrogenase [ubiquinone] 1 beta subcomplex subunit 4	NDUB4_HUMAN	O95168	1.25	0.0021	0.2005	1.13	0.1116	0.9982	0.95	0.5864	0.9960	1.02	0.7836	0.9939	1.14	0.0853	0.9951	0.99	0.8730	0.9959	0.96	0.6352	0.8887	0.99	0.8686	0.9275	1.06	0.4481	0.9889	1.12	0.1173	0.9607
NADH dehydrogenase [ubiquinone] 1 beta subcomplex subunit 5, mitoch	NDUB5_HUMAN	O43674	1.08	0.4088	0.6630	1.08	0.4179	0.9982	0.86	0.1570	0.9960	0.92	0.3951	0.9939	1.00	0.9653	0.9951	1.16	0.2076	0.9856	0.85	0.1069	0.6824	1.14	0.2287	0.4943	1.11	0.2975	0.9889	1.12	0.2105	0.9607
NADH dehydrogenase [ubiquinone] 1 beta subcomplex subunit 6	NDUB6_HUMAN	O95139	1.06	0.4910	0.7219	1.07	0.4272	0.9982	0.94	0.5510	0.9960	0.98	0.8069	0.9939	1.13	0.1821	0.9951	1.03	0.7582	0.9857	0.99	0.9411	0.9903	0.96	0.6906	0.8252	1.08	0.3553	0.9889	1.12	0.1730	0.9607

NADH dehydrogenase [ubiquinone] 1 beta subcomplex subunit 7	NDUB7_HUMAN	P17568	0.91	0.2160	0.4877	0.95	0.5241	0.9982	0.97	0.7436	0.9960	0.85	0.0288	0.9874	0.89	0.1302	0.9951	1.00	0.9700	0.9967	0.93	0.3939	0.7856	1.17	0.0653	0.2631	1.03	0.7005	0.9889	0.96	0.5505	0.9881
NADH dehydrogenase [ubiquinone] 1 beta subcomplex subunit 8, mitoch	NDUB8_HUMAN	O95169	1.08	0.3675	0.6280	1.06	0.4549	0.9982	0.95	0.5542	0.9960	0.89	0.1609	0.9874	1.03	0.7201	0.9951	1.02	0.8144	0.9959	0.93	0.4195	0.8021	1.03	0.7846	0.8852	1.06	0.5227	0.9889	1.09	0.2851	0.9607
NADH dehydrogenase [ubiquinone] 1 beta subcomplex subunit 9	NDUB9_HUMAN	Q9Y6M9	1.01	0.9287	0.9592	1.03	0.7459	0.9982	0.91	0.3876	0.9960	0.85	0.0762	0.9874	0.94	0.4979	0.9951	1.13	0.3033	0.9856	0.92	0.3747	0.7717	1.19	0.0908	0.3041	1.13	0.2067	0.9889	1.03	0.7470	0.9881
NADH dehydrogenase [ubiquinone] 1 subunit C2	NDUC2_HUMAN	O95298	0.99	0.9112	0.9528	1.03	0.6833	0.9982	0.95	0.5616	0.9960	0.89	0.1561	0.9874	0.93	0.4128	0.9951	0.99	0.9166	0.9959	0.86	0.0895	0.6824	1.13	0.1645	0.4153	1.02	0.7975	0.9889	1.02	0.8483	0.9881
NADH dehydrogenase [ubiquinone] flavoprotein 1, mitochondrial	NDUV1_HUMAN	P49821	1.08	0.3663	0.6279	1.01	0.8989	0.9982	0.95	0.6256	0.9960	0.83	0.0322	0.9874	0.94	0.5210	0.9951	1.00	0.9808	0.9992	0.88	0.1585	0.6871	1.16	0.1354	0.3764	1.15	0.1182	0.9889	1.05	0.5485	0.9881
NADH dehydrogenase [ubiquinone] flavoprotein 2, mitochondrial	NDUV2_HUMAN	P19404	1.07	0.4295	0.6776	1.00	0.9706	0.9982	0.88	0.2194	0.9960	0.88	0.1519	0.9874	0.95	0.5968	0.9951	1.10	0.4020	0.9856	0.83	0.0515	0.6824	1.21	0.0570	0.2478	1.21	0.0374	0.9889	1.08	0.4075	0.9607
NADH dehydrogenase [ubiquinone] flavoprotein 3, mitochondrial	NDUV3_HUMAN	P56181	1.06	0.4557	0.6983	1.00	0.9691	0.9982	0.99	0.8951	0.9960	0.93	0.3672	0.9939	1.02	0.8094	0.9951	0.98	0.8268	0.9959	0.97	0.7247	0.9205	1.10	0.2676	0.5288	1.07	0.4221	0.9889	1.06	0.4673	0.9607
NADH dehydrogenase [ubiquinone] iron-sulfur protein 2, mitochondrial	NDUS2_HUMAN	O75306	1.11	0.2344	0.5075	1.01	0.8861	0.9982	0.94	0.5832	0.9960	0.86	0.0803	0.9874	0.98	0.8029	0.9951	1.00	0.9694	0.9967	0.89	0.2007	0.7149	1.17	0.1152	0.3456	1.09	0.3453	0.9889	1.06	0.5207	0.9846
NADH dehydrogenase [ubiquinone] iron-sulfur protein 3, mitochondrial	NDUS3_HUMAN	O75489	1.09	0.3384	0.6022	1.04	0.6253	0.9982	0.95	0.5958	0.9960	0.89	0.1762	0.9907	0.99	0.8792	0.9951	1.01	0.9197	0.9959	0.89	0.2251	0.7187	1.17	0.1235	0.3567	1.16	0.1102	0.9889	1.09	0.3191	0.9607
NADH dehydrogenase [ubiquinone] iron-sulfur protein 4, mitochondrial	NDUS4_HUMAN	O43181	1.06	0.5316	0.7434	1.03	0.7523	0.9982	0.85	0.1347	0.9960	0.97	0.7403	0.9939	1.00	0.9797	0.9964	1.13	0.2758	0.9856	0.84	0.0817	0.6824	1.13	0.2267	0.4943	1.20	0.0511	0.9889	1.10	0.3166	0.9607
NADH dehydrogenase [ubiquinone] iron-sulfur protein 5	NDUS5_HUMAN	O43920	1.03	0.6660	0.8376	1.07	0.3030	0.9982	0.93	0.3688	0.9960	0.96	0.5348	0.9939	0.97	0.6610	0.9951	1.04	0.6259	0.9856	0.92	0.2690	0.7187	1.07	0.3808	0.6115	1.13	0.0758	0.9889	1.06	0.4102	0.9607
NADH dehydrogenase [ubiquinone] iron-sulfur protein 6, mitochondrial	NDUS6_HUMAN	O75380	1.13	0.1316	0.3958	1.06	0.4896	0.9982	0.92	0.4225	0.9960	0.93	0.3642	0.9939	1.04	0.6893	0.9951	1.03	0.7674	0.9857	0.88	0.1550	0.6871	1.12	0.2242	0.4912	1.16	0.0786	0.9889	1.12	0.1667	0.9607
NADH dehydrogenase [ubiquinone] iron-sulfur protein 7, mitochondrial	NDUS7_HUMAN	O75251	1.20	0.0283	0.2873	1.08	0.3347	0.9982	0.92	0.4050	0.9960	0.95	0.5757	0.9939	1.07	0.4480	0.9951	1.04	0.7093	0.9857	0.92	0.3709	0.7673	1.12	0.2314	0.4953	1.09	0.3341	0.9889	1.13	0.1281	0.9607
NADH dehydrogenase [ubiquinone] iron-sulfur protein 8, mitochondrial	NDUS8_HUMAN	O00217	1.08	0.3661	0.6279	1.03	0.7405	0.9982	0.94	0.5531	0.9960	0.89	0.1562	0.9874	0.98	0.8163	0.9951	1.02	0.8602	0.9959	0.89	0.1682	0.6934	1.20	0.0446	0.2250	1.16	0.0781	0.9889	1.10	0.2302	0.9607
NADH-cytochrome b5 reductase 1	NB5R1_HUMAN	Q9UHQ9	1.09	0.3487	0.6115	1.07	0.4355	0.9982	1.04	0.6763	0.9960	1.15	0.1245	0.9874	1.14	0.1611	0.9951	0.86	0.1639	0.9856	0.94	0.5252	0.8558	0.95	0.6313	0.7907	1.12	0.2205	0.9889	1.15	0.1239	0.9607
NADH-cytochrome b5 reductase 3	NB5R3_HUMAN	P00387	0.91	0.1874	0.4611	0.95	0.5196	0.9982	1.13	0.1481	0.9960	0.99	0.9121	0.9939	0.95	0.5455	0.9951	0.88	0.1874	0.9856	1.06	0.4932	0.8421	0.91	0.2910	0.5447	1.04	0.6198	0.9889	0.98	0.7462	0.9881
NADH-ubiquinone oxidoreductase 75 kDa subunit, mitochondrial	NDUS1_HUMAN	P28331	1.10	0.2946	0.5663	1.01	0.9042	0.9982	0.96	0.7183	0.9960	0.85	0.0692	0.9874	0.95	0.6242	0.9951	0.99	0.9290	0.9959	0.86	0.1281	0.6868	1.20	0.0829	0.2939	1.18	0.0915	0.9889	1.09	0.3474	0.9607
NADP-dependent malic enzyme	MAOX_HUMAN	P48163	1.13	0.2002	0.4802	0.96	0.6841	0.9982	0.99	0.8886	0.9960	1.07	0.4481	0.9939	1.05	0.6005	0.9951	1.19	0.1215	0.9856	0.98	0.8746	0.9720	1.22	0.0520	0.2413	0.96	0.6460	0.9889	0.99	0.9245	0.9938
NADP-dependent malic enzyme, mitochondrial	MAON_HUMAN	Q16798	1.19	0.0704	0.3213	1.14	0.1788	0.9982	0.99	0.9139	0.9960	0.99	0.9129	0.9939	0.99	0.8910	0.9951	1.00	0.9958	0.9992	0.88	0.1960	0.7149	1.05	0.6779	0.8195	1.00	0.9812	0.9976	1.02	0.8166	0.9881
NADPH--cytochrome P450 reductase	NCPR_HUMAN	P16435	0.94	0.2893	0.5630	1.04	0.5343	0.9982	0.96	0.5195	0.9960	1.01	0.8865	0.9939	0.95	0.3586	0.9951	0.95	0.4412	0.9856	1.03	0.6491	0.8897	0.88	0.0349	0.2017	1.02	0.7327	0.9889	0.99	0.7965	0.9881
Nascent polypeptide-associated complex subunit alpha, muscle-specific f	NACAM_HUMAN	E9PAV3	1.10	0.3191	0.5886	1.04	0.7137	0.9982	1.15	0.2147	0.9960	1.02	0.8237	0.9939	0.98	0.8307	0.9951	0.93	0.5659	0.9856	0.88	0.2085	0.7149	1.26	0.0291	0.1909	1.05	0.5967	0.9889	1.05	0.5751	0.9881
Natriuretic peptides A	ANF_HUMAN	P01160	1.25	0.4762	0.7136	1.84	0.0467	0.9982	0.73	0.3815	0.9960	1.31	0.3796	0.9939	1.11	0.7418	0.9951	1.49	0.2979	0.9856	1.15	0.6711	0.8938	0.70	0.2967	0.5464	2.47	0.0034	0.9889	1.25	0.4677	0.9607
Nebulette	NEBL_HUMAN	O76041	0.97	0.6648	0.8375	1.04	0.5690	0.9982	1.03	0.7790	0.9960	0.93	0.3382	0.9939	0.89	0.1369	0.9951	0.92	0.3826	0.9856	0.88	0.1313	0.6871	1.04	0.6707	0.8127	1.01	0.8718	0.9889	0.99	0.9079	0.9938
Nebulin-related-anchoring protein	NRAP_HUMAN	Q86VF7	1.03	0.6934	0.8528	0.90	0.1774	0.9982	1.00	0.9793	0.9960	1.09	0.2755	0.9939	1.08	0.3239	0.9951	0.92	0.3941	0.9856	1.05	0.5428	0.8580	1.07	0.4729	0.6780	1.04	0.6198	0.9889	1.16	0.0578	0.9607
NEDD8	NEDD8_HUMAN	Q15843	1.22	0.0636	0.3086	0.88	0.2344	0.9982	0.97	0.8136	0.9960	1.04	0.7074	0.9939	1.13	0.3010	0.9951	1.11	0.4474	0.9856	0.92	0.4569	0.8225	1.14	0.2886	0.5420	1.06	0.5924	0.9889	1.12	0.2891	0.9607
Nestin	NEST_HUMAN	P48681	0.89	0.2008	0.4810	0.91	0.3122	0.9982	1.03	0.7435	0.9960	1.05	0.5564	0.9939	0.89	0.2187	0.9951	1.05	0.6823	0.9856	0.93	0.4617	0.8243	1.27	0.0163	0.1636	1.02	0.8279	0.9889	0.91	0.2732	0.9607
Neudesin	NENF_HUMAN	Q9UMX5	0.99	0.8902	0.9422	1.08	0.2944	0.9982	1.20	0.0383	0.9960	0.96	0.5935	0.9939	0.94	0.4652	0.9951	0.87	0.1498	0.9856	0.98	0.8293	0.9494	0.97	0.7051	0.8348	0.99	0.8991	0.9889	0.96	0.5467	0.9881
Neural cell adhesion molecule 1	NCAM1_HUMAN	P13591	0.96	0.4809	0.7173	0.96	0.5044	0.9982	1.06	0.4137	0.9960	1.05	0.4311	0.9939	0.99	0.9282	0.9951	0.91	0.1919	0.9856	0.93	0.2750	0.7196	0.98	0.7795	0.8827	0.97	0.6654	0.9889	1.00	0.9917	0.9992
Neuroblast differentiation-associated protein AHNAK	AHNK_HUMAN	Q09666	0.84	0.0297	0.2873	1.04	0.6390	0.9982	1.07	0.4452	0.9960	1.04	0.6238	0.9939	0.85	0.0591	0.9951	0.95	0.6125	0.9856	1.00	0.9609	0.9924	0.81	0.0153	0.1606	0.97	0.7057	0.9889	0.86	0.0510	0.9607
Neuropilin-1	NRP1_HUMAN	O14786	0.89	0.1395	0.4030	1.07	0.3995	0.9982	1.05	0.5938	0.9960	1.02	0.8294	0.9939	0.97	0.7050	0.9951	0.96	0.6347	0.9856	0.98	0.8421	0.9543	0.89	0.1566	0.4056	1.04	0.6259	0.9889	0.95	0.5365	0.9881
Neuroplastin	NPTN_HUMAN	Q9Y639	0.84	0.0469	0.2898	0.92	0.3488	0.9982	1.19	0.0857	0.9960	1.09	0.3498	0.9939	0.95	0.5459	0.9951	0.80	0.0322	0.9411	1.08	0.4049	0.7880	0.80	0.0240	0.1763	0.91	0.3190	0.9889	0.93	0.3994	0.9607
Neutral alpha-glucosidase AB	GANAB_HUMAN	Q14697	0.99	0.7756	0.8925	0.97	0.4479	0.9982	1.04	0.4101	0.9960	1.03	0.5461	0.9939	0.98	0.6044	0.9951	0.95	0.3717	0.9856	1.02	0.7143	0.9154	1.02	0.7141	0.8360	0.94	0.1694	0.9889	0.95	0.2851	0.9607
Neutral cholesterol ester hydrolase 1	NCEH1_HUMAN	Q6PIU2	0.99	0.8308	0.9186	0.97	0.6794	0.9982	1.14	0.1066	0.9960	1.13	0.0608	0.9874	1.07	0.3586	0.9951	0.83	0.0294	0.9411	1.02	0.7684	0.9296	1.08	0.3321	0.5701	0.92	0.2630	0.9889	0.98	0.8149	0.9881
Neutrophil defensin 1	DEF1_HUMAN	P59665	0.94	0.7011	0.8595	1.02	0.9208	0.9982	1.39	0.1041	0.9960	1.01	0.9427	0.9939	1.00	0.9837	0.9964	0.63	0.0315	0.9411	1.35	0.1070	0.6824	0.70	0.0650	0.2631	0.81	0.2508	0.9889	0.94	0.7370	0.9881
Nexilin	NEXN_HUMAN	Q0ZGT2	0.90	0.0772	0.3266	0.97	0.6488	0.9982	0.96	0.5685	0.9960	1.06	0.3031	0.9939	0.99	0.8476	0.9951	1.00	0.9704	0.9967	1.00	0.9872	0.9992	0.94	0.3837	0.6141	0.99	0.8871	0.9889	1.00	0.9438	0.9938
NFU1 iron-sulfur cluster scaffold homolog, mitochondrial	NFU1_HUMAN	Q9UMS0	1.11	0.2121	0.4843	1.09	0.2930	0.9982	0.96	0.6672	0.9960	0.93	0.3779	0.9939	0.94	0.4856	0.9951	1.07	0.5200	0.9856	0.87	0.1117	0.6824	1.08	0.4284	0.6500	0.97	0.6944	0.9889	0.97	0.7030	0.9881
NHL repeat-containing protein 2	NHLC2_HUMAN	Q8NBF2	1.04	0.5234	0.7378	0.92	0.1980	0.9982	1.04	0.5964	0.9960	1.06	0.4002	0.9939	1.00	0.9535	0.9951	1.08	0.3364	0.9856	0.94	0.4119	0.7917	1.08	0.2628	0.5247	0.97	0.6526	0.9889	1.04	0.5683	0.9881
NHP2-like protein 1	NH2L1_HUMAN	P55769	0.89	0.0763	0.3266	1.05	0.5095	0.9982	1.05	0.5041	0.9960	0.92	0.2422	0.9939	0.90	0.1339	0.9951	0.92	0.3325	0.9856	1.01	0.9118	0.9848	0.88	0.0763	0.2842	0.95	0.4742	0.9889	0.94	0.3613	0.9607
Nicastrin	NICA_HUMAN	Q92542	1.01	0.8348	0.9199	1.14	0.0372	0.9982	1.03	0.6637	0.9960	1.08	0.2162	0.9939	1.07	0.3306	0.9951	0.91	0.2283	0.9856	1.09	0.2068	0.7149	0.80	0.0014	0.0763	1.03	0.6421	0.9889	0.97	0.6695	0.9881
Nicotinamide phosphoribosyltransferase	NAMPT_HUMAN	P43490	0.99	0.9493	0.9699	1.07	0.4538	0.9982	1.10	0.3373	0.9960	0.89	0.1630	0.9874	0.88	0.1510	0.9951	0.89	0.2422	0.9856	0.97	0.7384	0.9218	0.91	0.3035	0.5503	0.83	0.0299	0.9889	0.85	0.0513	0.9607
Nidogen-1	NID1_HUMAN	P14543	0.94	0.3160	0.5859	0.97	0.6523	0.9982	1.15	0.0579	0.9960	1.04	0.5907	0.9939	0.96	0.5971	0.9951	0.87	0.0869	0.9856	1.11	0.1230	0.6868	1.00	0.9702	0.9832	0.93	0.3062	0.9889	0.88	0.0500	0.9607
Nidogen-2	NID2_HUMAN	Q14112	0.85	0.0504	0.2898	0.98	0.8011	0.9982	1.17	0.1038	0.9960	0.93	0.4078	0.9939	0.89	0.1776	0.9951	0.85	0.1035	0.9856	1.08	0.4039	0.7880	0.86	0.1018	0.3256	0.96	0.6756	0.9889	0.83	0.0265	0.9607
Nodal modulator 1	NOMO1_HUMAN	Q15155	0.90	0.0760	0.3266	0.99	0.8205	0.9982	1.15	0.0364	0.9960	1.01	0.8736	0.9939	0.96	0.5322	0.9951	0.86	0.0407	0.9411	1.12	0.0551	0.6824	0.94	0.3715	0.6006	0.96	0.4610	0.9889	0.96	0.5387	0.9881
Non-histone chromosomal protein HMG-17	HMGN2_HUMAN	P05204	0.93	0.4976	0.7246	1.01	0.9372	0.9982	0.98	0.8465	0.9960	0.89	0.2248	0.9939	0.88	0.2091	0.9951	1.15	0.2703	0.9856	0.82	0.0625	0.6824	1.21	0.0822	0.2933	1.02	0.8562	0.9889	0.87	0.1696	0.9607
Non-POU domain-containing octamer-binding protein	NONO_HUMAN	Q15233	0.98	0.7422	0.8802	1.08	0.1592	0.9982	1.00	0.9576	0.9960	1.04	0.4874	0.9939	0.94	0.2840	0.9951	0.98	0.7630	0.9857	1.07	0.2627	0.7187	0.94	0.3150	0.5586	1.04	0.4879	0.9889	0.97	0.5796	0.9881
Non-specific lipid-transfer protein	NLTP_HUMAN	P22307	1.02	0.7690	0.8891	0.93	0.2519	0.9982	0.86	0.0399	0.9960	0.98	0.7228	0.9939	1.00	0.9462	0.9951	1.17	0.0464	0.9535	1.11	0.1115	0.6824	1.06	0.3847	0.6145	0.94	0.3493	0.9889	0.95	0.4608	0.9607
NPC intracellular cholesterol transporter 2	NPC2_HUMAN	P61916	1.04	0.6051	0.7977	1.15	0.0949	0.9982	0.94	0.5223	0.9960	0.91	0.2550	0.9939	0.98	0.8617	0.9951	1.10	0.3435	0.9856	0.95	0.5417	0.8573	0.88	0.1706	0.4217	0.94	0.4669	0.9889	0.95	0.5398	0.9881
NSFL1 cofactor p47	NSF1C_HUMAN	Q9UNZ2	1.05	0.5700	0.7691	1.03	0.7576	0.9982	0.97	0.7803	0.9960	0.90	0.2625	0.9939	0.96	0.6571	0.9951	1.11	0.3375	0.9856	0.88	0.1861	0.7149	1.20	0.0730	0.2778	0.87	0.1333	0.9889	0.92	0.3659	0.9607
Nuclear migration protein nudC	NUDC_HUMAN	Q9Y266	1.00	0.9296	0.9592	1.09	0.1191	0.9982	1.13	0.0374	0.9960	1.00	0.9519	0.9939	0.96	0.4163	0.9951	0.88	0.0373	0.9411	1.00	0.9909	0.9992	0.95	0.3573	0.5925	0.92	0.1372	0.9889	0.92	0.0878	0.9607
Nuclear transport factor 2	NTF2_HUMAN	P61970	1.12	0.2535	0.5227	0.97	0.7232	0.9982	1.04	0.7127	0.9960	0.89	0.2196	0.9939	0.99	0.9575	0.9951	1.03	0.7902	0.9903	0.81	0.0357	0.6824	1.24	0.0412	0.2167	0.92	0.3864	0.9889	0.97	0.7504	0.9881
Nucleobindin-1	NUCB1_HUMAN	Q02818	1.02	0.7237	0.8735	0.98	0.6711	0.9982	1.00	0.9542	0.9960	1.00	0.9641	0.9947	1.01	0.7816	0.9951	1.00	0.9970	0.9992	1.04	0.4983	0.8476	0.92	0.1085	0.3351	1.00	0.9290	0.9894	1.01	0.8321	0.9881
Nucleobindin-2	NUCB2_HUMAN	P80303	1.01	0.8637	0.9348	0.95	0.2947	0.9982	1.01	0.8408	0.9960	1.01	0.8936	0.9939	0.97	0.5278	0.9951	1.05	0.4805	0.9856	1.02	0.7667	0.9291	1.00	0.9527	0.9734	0.95	0.3571	0.9889	0.97	0.5604	0.9881
Nucleolar protein 3	NOL3_HUMAN	O60936	1.17	0.0306	0.2873	1.01	0.8855	0.9982	0.92	0.3110	0.9960	1.10	0.1990	0.9939	1.06	0.4513	0.9951	1.16	0.1037	0.9856	0.92	0.2673	0.7187	1.11	0.1810	0.4355	1.06	0.4433	0.9889	1.06	0.3904	0.9607
Nucleolin	NUCL_HUMAN	P19338	1.06	0.4086	0.6630	1.06	0.4017	0.9982	1.02	0.7689	0.9960	0.94	0.3495	0.9939	0.93	0.2956	0.9951	1.02	0.8112	0.9959	0.90	0.1416	0.6871	1.10	0.1815	0.4355	0.99	0.9153	0.9889	0.93	0.2606	0.9607
Nucleophosmin	NPM_HUMAN	P06748	0.91	0.0546	0.2939	0.97	0.5992	0.9982	1.01	0.8434	0.9960	0.94	0.2463	0.9939	0.91	0.0617	0.9951	1.01	0.8846	0.9959	1.01	0.8524	0.9583	1.05	0.4101	0.6358	0.98	0.6470	0.9889	0.94	0.2440	0.9607
Nucleoporin Nup37	NUP37_HUMAN	Q8NFH4	1.06	0.3565	0.6187	1.08	0.2274	0.9982	1.04	0.6492	0.9960	1.01	0.9130	0.9939	0.94	0.3748	0.9951	0.94	0.4472	0.9856	1.01	0.8459	0.9565	0.91	0.2000	0.4593	1.00	0.9769	0.9976	0.93	0.2559	0.9607
Nucleoprotein TPR	TPR_HUMAN	P12270	0.87	0.0539	0.2939	0.97	0.6628	0.9982	1.01	0.9078	0.9960	1.02	0.8184	0.9939	0.98	0.7516	0.9951	1.02	0.8174	0.9959	1.00	0.9907	0.9992	0.87	0.0833	0.2940	1.15	0.0570	0.9889	1.08	0.2816	0.9607
Nucleoside diphosphate kinase B	NDKB_HUMAN	P22392	1.00	0.9478	0.9699	1.00	0.9799	0.9985	0.98	0.6693	0.9960	0.98	0.6608	0.9939	1.00	0.9739	0.9951	1.05	0.3708	0.9856	0.97	0.5861	0.8645	1.00	0.9886	0.9937	1.01	0.8697	0.9889	0.99	0.8813	0.9938
Nucleoside diphosphate-linked moiety X motif 19	NUD19_HUMAN	A8MXV4	1.17	0.0500	0.2898	0.92	0.2779	0.9982	0.81	0.0212	0.9960	1.06	0.4763	0.9939	1.04	0.6180	0.9951	1.27	0.0150	0.9411	0.98	0.7965	0.9431	1.21	0.0319	0.2001	1.01	0.8840	0.9889	1.09	0.2689	0.9607
Nucleosome assembly protein 1-like 1	NP1L1_HUMAN	P55209	0.94	0.3195	0.5886	0.96	0.4686	0.9982	0.99	0.8676	0.9960	1.03	0.6648	0.9939	0.89	0.0711	0.9951	1.01	0.9067	0.9959	1.04	0.5473	0.8600	1.00	0.9944	0.9968	1.01	0.8511	0.9889	0.91	0.1218	0.9607
Nucleosome assembly protein 1-like 4	NP1L4_HUMAN	Q99733	1.03	0.5386	0.7494	1.02	0.6528	0.9982	1.02	0.6762	0.9960	1.08	0.1166	0.9874	1.02	0.6817	0.9951	1.00	0.9586	0.9967	0.98	0.6578	0.8915	1.00	0.9949	0.9968	1.02	0.6632	0.9889	1.01	0.8868	0.9938
Obg-like ATPase 1	OLA1_HUMAN	Q9NTK5	1.00	0.9427	0.9668	1.05	0.4756	0.9982	0.93	0.3273	0.9960	0.98	0.7531	0.9939	0.95	0.5009	0.9951	1.13	0.1350	0.9856	0.98	0.7582	0.9282	1.08	0.3001	0.5487	1.02	0.8074	0.9889	0.98	0.7680	0.9881
Obscurin	OBSCN_HUMAN	Q5VST9	1.00	0.9799	0.9886	1.00	0.9560	0.9982	1.08	0.4355	0.9960	0.95	0.5530	0.9939	0.91	0.3304	0.9951	0.93	0.5038	0.9856	0.88	0.1787	0.7130	1.17	0.1146	0.3452	0.91	0.2883	0.9889	0.95	0.5249	0.9846
OCIA domain-containing protein 1	OCAD1_HUMAN	Q9NX40	0.90	0.1074	0.3670	1.03	0.6554	0.9982	1.08	0.3200	0.9960	0.97	0.6419	0.9939	0.97	0.6167	0.9951	0.94	0.4338	0.9856	1.04	0.5250	0.8558	0.90	0.1502	0.3999	1.00	0.9809	0.9976	0.92	0.2146	0.9607
Oligoribonuclease, mitochondrial	ORN_HUMAN	Q9Y3B8	1.22	0.0190	0.2873	1.06	0.4941	0.9982	0.97	0.7531	0.9960	0.89	0.1719	0.9874	1.00	0.9860	0.9964	1.04	0.6767	0.9856	0.85	0.0760	0.6824	1.11	0.2859	0.5395	0.99	0.9429	0.9922	1.03	0.7135	0.9881
Omega-amidase NIT2	NIT2_HUMAN	Q9NQR4	1.11	0.2100	0.4843	1.04	0.6461	0.9982	0.92	0.3734	0.9960	0.90	0.2040	0.9939	0.97	0.6886	0.9951	1.12	0.2692	0.9856	0.90	0.2485	0.7187	1.17	0.0815	0.2918	0.98	0.7943	0.9889	1.00	0.9999	0.9999
Optineurin	OPTN_HUMAN	Q96CV9	0.95	0.3710	0.6312	0.94	0.3115	0.9982	1.03	0.6429	0.9960	0.91	0.1239	0.9874	0.86	0.0134	0.9951	0.99	0.9029	0.9959	0.88	0.0480	0.6824	1.07	0.3006	0.5487	0.96	0.5169	0.9889	0.93	0.1934	0.9607
Oxysterol-binding protein 1	OSBP1_HUMAN	P22059	1.02	0.7422	0.8802	0.96	0.5238	0.9982	1.04	0.6232	0.9960	1.05	0.4351	0.9939	0.99	0.8896	0.9951	0.96	0.6371	0.9856	1.09	0.2088	0.7149	1.12	0.1078	0.3341	0.95	0.4579	0.9889	1.00	0.9967	0.9999
Palladin	PALLD_HUMAN	Q8WX93	0.94	0.3319	0.5968	1.07	0.3222	0.9982	1.05	0.5013	0.9960	1.00	0.9706	0.9961	1.02	0.7575	0.9951	0.94	0.4234	0.9856	1.03	0.7140	0.9154	0.79	0.0005	0.0621	0.99	0.8536	0.9889	1.07	0.3210	0.9607
Palmdelphin	PALMD_HUMAN	Q9NP74	1.16	0.0106	0.2530	1.03	0.6388	0.9982	0.96	0.5660	0.9960	1.04	0.5574	0.9939	1.08	0.2086	0.9951	1.03	0.6615	0.9856	0.92	0.2202	0.7187	1.06	0.3888	0.6178	1.01	0.9069	0.9889	1.06	0.3352	0.9607
Palmitoyl-protein thioesterase 1	PPT1_HUMAN	P50897	1.09	0.1815	0.4568	1.07	0.3158	0.9982	0.96	0.6080	0.9960	1.01	0.9239	0.9939	1.12	0.1163	0.9951	1.01	0.8965	0.9959	0.99	0.8914	0.9754	0.95	0.5054	0.7019	1.03	0.7244	0.9889	1.08	0.2811	0.9607
Paralemmin-1	PALM_HUMAN	O75781	1.11	0.1935	0.4723	1.07	0.4016	0.9982	0.94	0.4961	0.9960	1.15	0.0845	0.9874	1.13	0.1339	0.9951	1.07	0.4832	0.9856	0.98	0.7806	0.9362	0.87	0.1144	0.3452	1.04	0.6067	0.9889	1.08	0.3326	0.9607
Paralemmin-2	PALM2_HUMAN	Q8IXS6	0.98	0.8555	0.9307	1.00	0.9701	0.9982	0.98	0.8746	0.9960	0.98	0.8023	0.9939	0.95	0.6342	0.9951	0.91	0.4477	0.9856	0.93	0.4824	0.8361	1.26	0.0304	0.1924	1.13	0.2246	0.9889	0.97	0.7209	0.9881
Paraplegin	SPG7_HUMAN	Q9UQ90	1.06	0.5068	0.7286	1.10	0.2473	0.9982	1.03	0.7696	0.9960	1.09	0.3185	0.9939	1.07	0.4412	0.9951	0.91	0.3559	0.9856	0.99	0.9146	0.9848	0.98	0.8034	0.8927	1.02	0.7968	0.9889	1.00	0.9970	0.9999
Parathymosin	PTMS_HUMAN	P20962	1.10	0.2336	0.5075	1.02	0.8017	0.9982	0.91	0.3203	0.9960	1.06	0.4546	0.9939	1.03	0.6947	0.9951	1.21	0.0427	0.9411	0.98	0.8468	0.9567	0.99	0.9363	0.9616	0.94	0.4075	0.9889	0.99	0.9100	0.9938
PDZ and LIM domain protein 1	PDLI1_HUMAN	O00151	0.96	0.5319	0.7434	0.89	0.0395	0.9982	0.98	0.7965	0.9960	1.02	0.8006	0.9939	1.01	0.8857	0.9951	1.04	0.6052	0.9856	0.92	0.1970	0.7149	1.12	0.1005	0.3232	0.98	0.7739	0.9889	0.99	0.8594	0.9881
PDZ and LIM domain protein 3	PDLI3_HUMAN	Q53GG5	1.12	0.2652	0.5347	1.07	0.5112	0.9982	1.07	0.5490	0.9960	0.98	0.8516	0.9939	0.96	0.7244	0.9951	0.90	0.3918	0.9856	0.89	0.2820	0.7212	0.74	0.0068	0.1326	1.08	0.4659	0.9889	1.12	0.2432	0.9607
PDZ and LIM domain protein 5	PDLI5_HUMAN	Q96HC4	1.18	0.0312	0.2873	0.97	0.7448	0.9982	0.86	0.1026	0.9960	1.00	0.9556	0.9939	1.09	0.2913	0.9951	1.08	0.4120	0.9856	1.05	0.5789	0.8632	1.12	0.2121	0.4786	1.05	0.5227	0.9889	1.20	0.0196	0.9607
Pentatricopeptide repeat domain-containing protein 3, mitochondrial	PTCD3_HUMAN	Q96EY7	1.02	0.7964	0.9014	0.97	0.6573	0.9982	1.05	0.5535	0.9960	0.99	0.8821	0.9939	0.99	0.9270	0.9951	0.95	0.5115	0.9856	0.93	0.3174	0.7400	0.99	0.8871	0.9377	1.15	0.0337	0.9889	1.07	0.3020	0.9607
Peptidyl-glycine alpha-amidating monooxygenase	AMD_HUMAN	P19021	0.94	0.4879	0.7214	1.04	0.6155	0.9982	1.05	0.5939	0.9960	1.12	0.1596	0.9874	0.97	0.7230	0.9951	0.88	0.2076	0.9856	0.99	0.9426	0.9903	0.84	0.0559	0.2474	0.99	0.8698	0.9889	1.02	0.8487	0.9881
Peptidyl-prolyl cis-trans isomerase A	PPIA_HUMAN	P62937	1.11	0.1522	0.4208	0.99	0.8469	0.9982	1.00	0.9782	0.9960	0.88	0.0672	0.9874	0.95	0.4967	0.9951	1.07	0.4399	0.9856	0.85	0.0342	0.6824	1.17	0.0547	0.2454	0.90	0.1393	0.9889	0.94	0.4273	0.9607
Peptidyl-prolyl cis-trans isomerase B	PPIB_HUMAN	P23284	0.80	0.0169	0.2873	0.98	0.8036	0.9982	1.07	0.5537	0.9960	0.96	0.6510	0.9939	0.81	0.0307	0.9951	0.96	0.7164	0.9857	1.09	0.3975	0.7880	0.92	0.4558	0.6696	0.92	0.3939	0.9889	0.81	0.0258	0.9607
Peptidyl-prolyl cis-trans isomerase F, mitochondrial	PPIF_HUMAN	P30405	1.25	0.0346	0.2873	1.05	0.6681	0.9982	0.92	0.4999	0.9960	0.98	0.8231	0.9939	0.97	0.7870	0.9951	1.10	0.4562	0.9856	0.87	0.2276	0.7187	1.24	0.0627	0.2590	1.08	0.4989	0.9889	1.03	0.7739	0.9881
Peptidyl-prolyl cis-trans isomerase FKBP1A	FKB1A_HUMAN	P62942	1.20	0.1308	0.3958	0.99	0.9106	0.9982	1.07	0.6128	0.9960	1.07	0.5942	0.9939	1.10	0.4760	0.9951	1.06	0.7221	0.9857	0.91	0.4901	0.8395	1.10	0.4691	0.6776	0.97	0.8067	0.9889	0.95	0.6989	0.9881
Peptidyl-prolyl cis-trans isomerase FKBP2	FKBP2_HUMAN	P26885	1.00	0.9866	0.9915	1.08	0.3198	0.9982	1.12	0.1790	0.9960	1.06	0.4464	0.9939	1.00	0.9791	0.9964	0.91	0.3295	0.9856	1.17	0.0548	0.6824	0.90	0.2153	0.4816	0.98	0.8035	0.9889	0.90	0.1795	0.9607
Peptidyl-prolyl cis-trans isomerase FKBP9	FKBP9_HUMAN	O95302	0.96	0.4111	0.6642	1.02	0.6677	0.9982	0.98	0.7354	0.9960	1.05	0.3723	0.9939	0.96	0.4213	0.9951	1.06	0.3798	0.9856	0.98	0.7172	0.9164	1.00	0.9507	0.9719	0.98	0.6920	0.9889	0.94	0.2430	0.9607
Peptidyl-prolyl cis-trans isomerase-like 1	PPIL1_HUMAN	Q9Y3C6	1.08	0.4455	0.6908	0.97	0.7559	0.9982	1.04	0.7421	0.9960	1.00	0.9788	0.9961	1.06	0.6236	0.9951	1.08	0.5597	0.9856	0.80	0.0384	0.6824	1.14	0.2494	0.5144	0.96	0.7308	0.9889	1.03	0.8017	0.9881
Perilipin-3	PLIN3_HUMAN	O60664	0.99	0.8374	0.9202	1.01	0.8059	0.9982	0.95	0.4889	0.9960	1.01	0.8257	0.9939	1.04	0.5349	0.9951	1.03	0.7339	0.9857	1.10	0.1267	0.6868	1.03	0.6323	0.7914	1.02	0.6949	0.9889	1.03	0.6215	0.9881
Perilipin-4	PLIN4_HUMAN	Q96Q06	1.04	0.5643	0.7665	1.09	0.1755	0.9982	1.07	0.3662	0.9960	1.12	0.0740	0.9874	0.95	0.4005	0.9951	0.90	0.1707	0.9856	0.96	0.5582	0.8601	0.95	0.4663	0.6760	0.94	0.3861	0.9889	0.95	0.4274	0.9607
Periostin	POSTN_HUMAN	Q15063	0.76	0.2911	0.5638	0.70	0.1557	0.9982	0.77	0.3734	0.9960	0.78	0.3293	0.9939	0.80	0.3915	0.9951	1.14	0.6744	0.9856	1.03	0.9029	0.9831	0.54	0.0264	0.1832	1.02	0.9482	0.9935	1.09	0.7375	0.9881
Peroxiredoxin-1	PRDX1_HUMAN	Q06830	0.94	0.3158	0.5859	1.01	0.7980	0.9982	1.03	0.6354	0.9960	0.93	0.2201	0.9939	0.91	0.1285	0.9951	1.04	0.6119	0.9856	1.00	0.9453	0.9905	1.03	0.6087	0.7788	0.96	0.5014	0.9889	0.95	0.3406	0.9607
Peroxiredoxin-2	PRDX2_HUMAN	P32119	1.10	0.2282	0.5015	0.98	0.7886	0.9982	0.97	0.6979	0.9960	0.92	0.2583	0.9939	0.96	0.5840	0.9951	1.13	0.1995	0.9856	0.95	0.5625	0.8601	1.24	0.0093	0.1378	1.00	0.9508	0.9945	1.00	0.9656	0.9983
Peroxiredoxin-4	PRDX4_HUMAN	Q13162	0.97	0.7713	0.8895	1.00	0.9728	0.9982	1.02	0.8727	0.9960	1.08	0.4412	0.9939	1.07	0.5316	0.9951	1.04	0.7486	0.9857	1.20	0.0879	0.6824	0.76	0.0168	0.1636	0.96	0.7293	0.9889	0.98	0.8631	0.9881
Peroxiredoxin-5, mitochondrial	PRDX5_HUMAN	P30044	1.05	0.4294	0.6776	1.04	0.4993	0.9982	0.89	0.0977	0.9960	0.96	0.4390	0.9939	0.95	0.3820	0.9951	1.13	0.0966	0.9856	0.93	0.2531	0.7187	1.10	0.1407	0.3837	1.03	0.6173	0.9889	0.98	0.6985	0.9881
Peroxiredoxin-6	PRDX6_HUMAN	P30041	1.06	0.4645	0.7050	1.04	0.6179	0.9982	1.02	0.8294	0.9960	0.95	0.5032	0.9939	0.95	0.5365	0.9951	1.01	0.9390	0.9959	0.92	0.3505	0.7593	1.06	0.4977	0.6969	0.98	0.8516	0.9889	0.94	0.4514	0.9607
Peroxiredoxin-like 2A	PXL2A_HUMAN	Q9BRX8	0.98	0.7515	0.8824	0.91	0.1505	0.9982	1.03	0.6800	0.9960	1.06	0.3821	0.9939	1.06	0.3914	0.9951	0.89	0.1657	0.9856	1.10	0.1602	0.6891	1.03	0.7199	0.8402	0.94	0.3609	0.9889	1.06	0.3826	0.9607
Peroxisomal membrane protein PEX14	PEX14_HUMAN	O75381	0.91	0.1140	0.3748	0.94	0.3177	0.9982	0.99	0.8654	0.9960	0.94	0.2956	0.9939	0.97	0.6379	0.9951	0.97	0.6968	0.9856	1.07	0.3228	0.7400	0.99	0.9144	0.9536	1.06	0.3418	0.9889	1.02	0.7658	0.9881
Peroxisomal multifunctional enzyme type 2	DHB4_HUMAN	P51659	0.90	0.2126	0.4846	1.03	0.7234	0.9982	1.03	0.7359	0.9960	1.08	0.3210	0.9939	1.10	0.2676	0.9951	0.96	0.6619	0.9856	1.09	0.3200	0.7400	0.83	0.0389	0.2155	0.97	0.6951	0.9889	1.01	0.9218	0.9938
Persulfide dioxygenase ETHE1, mitochondrial	ETHE1_HUMAN	O95571	1.15	0.0609	0.3030	1.06	0.4117	0.9982	1.07	0.4669	0.9960	1.00	0.9944	0.9986	1.14	0.0845	0.9951	0.92	0.3375	0.9856	1.07	0.4045	0.7880	0.85	0.0590	0.2505	0.94	0.3909	0.9889	1.02	0.7572	0.9881

Phosphate carrier protein, mitochondrial	MPCP_HUMAN	Q00325	1.03	0.7831	0.8985	1.02	0.8020	0.9982	1.06	0.6078	0.9960	0.97	0.7747	0.9939	1.01	0.9592	0.9951	0.91	0.4307	0.9856	0.88	0.1990	0.7149	1.10	0.3624	0.5970	0.98	0.8246	0.9889	1.02	0.8081	0.9881
Phosphatidylethanolamine-binding protein 1	PEBP1_HUMAN	P30086	1.12	0.3051	0.5744	0.98	0.8579	0.9982	1.01	0.9365	0.9960	0.86	0.1609	0.9874	0.93	0.5218	0.9951	1.13	0.3547	0.9856	0.84	0.1270	0.6868	1.31	0.0228	0.1743	0.96	0.7285	0.9889	0.97	0.8093	0.9881
Phosphatidylinositide phosphatase SAC1	SAC1_HUMAN	Q9NTJ5	1.01	0.8814	0.9384	0.98	0.7470	0.9982	0.99	0.8627	0.9960	1.02	0.6674	0.9939	1.07	0.2511	0.9951	0.98	0.7147	0.9857	1.01	0.8045	0.9457	0.90	0.1009	0.3232	0.96	0.4630	0.9889	1.03	0.6294	0.9881
Phosphoglucomutase-1	PGM1_HUMAN	P36871	1.04	0.7522	0.8825	0.87	0.2042	0.9982	0.89	0.3484	0.9960	0.85	0.1317	0.9874	0.91	0.3967	0.9951	1.21	0.1519	0.9856	0.82	0.0939	0.6824	1.37	0.0082	0.1361	0.85	0.1540	0.9889	0.89	0.2719	0.9607
Phosphoglucomutase-2	PGM2_HUMAN	Q96G03	1.08	0.3934	0.6514	0.96	0.6535	0.9982	0.70	0.0004	0.6184	0.88	0.1530	0.9874	0.94	0.4891	0.9951	1.48	0.0003	0.3206	0.93	0.4470	0.8181	1.05	0.6429	0.7988	0.99	0.9064	0.9889	0.93	0.4468	0.9607
Phosphoglucomutase-like protein 5	PGM5_HUMAN	Q15124	1.08	0.3020	0.5721	0.98	0.7397	0.9982	1.05	0.5387	0.9960	0.96	0.6073	0.9939	0.97	0.6855	0.9951	0.90	0.2310	0.9856	0.88	0.0988	0.6824	1.14	0.0877	0.2995	1.03	0.7118	0.9889	1.03	0.6837	0.9881
Phosphoglycerate kinase 1	PGK1_HUMAN	P00558	1.13	0.1133	0.3748	0.94	0.4378	0.9982	0.90	0.2558	0.9960	0.85	0.0359	0.9874	0.97	0.7510	0.9951	1.19	0.0661	0.9641	0.89	0.1527	0.6871	1.13	0.1727	0.4240	0.98	0.7802	0.9889	1.02	0.8282	0.9881
Phosphoglycerate mutase 1	PGAM1_HUMAN	P18669	1.07	0.4012	0.6589	0.94	0.4513	0.9982	0.96	0.6854	0.9960	0.91	0.2491	0.9939	0.99	0.8785	0.9951	1.15	0.1487	0.9856	0.87	0.1126	0.6824	1.11	0.2314	0.4953	0.96	0.6551	0.9889	0.96	0.6426	0.9881
Phosphoglycerate mutase 2	PGAM2_HUMAN	P15259	1.18	0.1362	0.3997	0.96	0.7449	0.9982	0.88	0.3228	0.9960	0.88	0.2186	0.9939	0.96	0.7524	0.9951	1.28	0.0657	0.9641	0.82	0.0890	0.6824	1.30	0.0274	0.1861	0.95	0.6433	0.9889	0.99	0.9402	0.9938
Phosphoribosyl pyrophosphate synthase-associated protein 2	KPRB_HUMAN	O60256	1.01	0.8745	0.9374	1.02	0.7491	0.9982	1.06	0.4073	0.9960	0.99	0.8344	0.9939	1.01	0.8347	0.9951	0.94	0.4311	0.9856	0.98	0.7099	0.9142	1.03	0.6666	0.8098	1.01	0.9094	0.9889	0.97	0.6446	0.9881
Phytanoyl-CoA dioxygenase, peroxisomal	PAHX_HUMAN	O14832	1.08	0.3479	0.6108	0.92	0.3592	0.9982	0.90	0.2958	0.9960	1.00	0.9613	0.9945	0.95	0.5321	0.9951	1.18	0.1230	0.9856	0.99	0.8896	0.9753	1.21	0.0393	0.2155	1.09	0.3021	0.9889	0.98	0.8062	0.9881
Plakophilin-2	PKP2_HUMAN	Q99959	0.99	0.8932	0.9439	1.03	0.6420	0.9982	1.02	0.8282	0.9960	1.01	0.9270	0.9939	1.02	0.7458	0.9951	0.92	0.3549	0.9856	0.98	0.7621	0.9282	0.96	0.5923	0.7646	1.05	0.4564	0.9889	1.10	0.1629	0.9607
Plasma membrane calcium-transporting ATPase 4	AT2B4_HUMAN	P23634	0.85	0.0508	0.2898	0.95	0.5350	0.9982	1.12	0.2194	0.9960	1.00	0.9563	0.9939	0.94	0.4887	0.9951	0.83	0.0643	0.9641	0.94	0.4439	0.8180	1.02	0.8531	0.9202	0.91	0.2617	0.9889	0.97	0.6811	0.9881
Plasma protease C1 inhibitor	IC1_HUMAN	P05155	0.82	0.1376	0.4012	1.03	0.8334	0.9982	1.07	0.6718	0.9960	0.86	0.2474	0.9939	0.84	0.2209	0.9951	0.93	0.6589	0.9856	1.15	0.3246	0.7400	0.74	0.0422	0.2200	0.97	0.8145	0.9889	0.87	0.2867	0.9607
Plasminogen	PLMN_HUMAN	P00747	0.86	0.1973	0.4751	1.15	0.2338	0.9982	1.07	0.6402	0.9960	0.94	0.5822	0.9939	0.95	0.6522	0.9951	0.91	0.5127	0.9856	1.13	0.3150	0.7400	0.71	0.0085	0.1361	1.04	0.7260	0.9889	0.87	0.2189	0.9607
Plasminogen activator inhibitor 1 RNA-binding protein	PAIRB_HUMAN	Q8NC51	0.79	0.0138	0.2710	0.84	0.0590	0.9982	1.23	0.0546	0.9960	0.96	0.6479	0.9939	0.90	0.2707	0.9951	0.93	0.5506	0.9856	1.03	0.8054	0.9457	1.12	0.2693	0.5288	0.88	0.2109	0.9889	0.79	0.0129	0.9304
Plastin-2	PLSL_HUMAN	P13796	0.81	0.1115	0.3738	1.16	0.2519	0.9982	1.08	0.6144	0.9960	0.87	0.2973	0.9939	0.81	0.1268	0.9951	0.92	0.6110	0.9856	1.08	0.5736	0.8630	0.81	0.1565	0.4056	0.86	0.2856	0.9889	0.74	0.0199	0.9607
Plastin-3	PLST_HUMAN	P13797	0.94	0.3855	0.6435	0.91	0.1848	0.9982	0.92	0.3397	0.9960	0.91	0.1699	0.9874	0.91	0.2121	0.9951	1.19	0.0484	0.9535	0.99	0.9204	0.9848	1.01	0.8624	0.9252	1.02	0.8151	0.9889	0.94	0.3529	0.9607
Platelet glycoprotein 4	CD36_HUMAN	P16671	0.97	0.7257	0.8738	0.93	0.4748	0.9982	1.21	0.0817	0.9960	0.99	0.9177	0.9939	1.04	0.6918	0.9951	0.78	0.0258	0.9411	0.96	0.6693	0.8929	0.91	0.3461	0.5794	0.77	0.0054	0.9889	0.90	0.2484	0.9607
Platelet-activating factor acetylhydrolase IB subunit alpha	LIS1_HUMAN	P43034	1.05	0.3147	0.5854	1.04	0.4531	0.9982	1.05	0.3637	0.9960	1.09	0.1013	0.9874	1.06	0.2666	0.9951	0.96	0.4933	0.9856	1.02	0.6615	0.8929	0.93	0.2120	0.4786	1.06	0.2855	0.9889	1.03	0.5740	0.9881
Platelet-activating factor acetylhydrolase IB subunit beta	PA1B2_HUMAN	P68402	1.00	0.9852	0.9915	0.92	0.3910	0.9982	1.00	0.9915	0.9960	0.85	0.1035	0.9874	0.92	0.4137	0.9951	0.99	0.9210	0.9959	0.87	0.1879	0.7149	1.17	0.1616	0.4114	0.96	0.7038	0.9889	0.97	0.7331	0.9881
Plectin	PLEC_HUMAN	Q15149	0.89	0.0545	0.2939	1.06	0.3605	0.9982	1.08	0.2810	0.9960	0.95	0.4412	0.9939	0.93	0.3002	0.9951	0.95	0.4979	0.9856	0.98	0.8098	0.9473	0.93	0.2985	0.5473	1.05	0.4250	0.9889	1.00	0.9405	0.9938
Plexin domain-containing protein 2	PXDC2_HUMAN	Q6UX71	1.02	0.8865	0.9402	0.86	0.2600	0.9982	0.94	0.6686	0.9960	1.13	0.3645	0.9939	1.15	0.3107	0.9951	1.04	0.8173	0.9959	1.33	0.0444	0.6824	0.69	0.0127	0.1544	0.97	0.8510	0.9889	1.05	0.6905	0.9881
Podocalyxin	PODXL_HUMAN	O00592	0.91	0.2060	0.4823	1.09	0.2425	0.9982	1.03	0.7404	0.9960	1.01	0.9463	0.9939	0.96	0.6437	0.9951	0.94	0.5112	0.9856	1.04	0.5886	0.8645	0.83	0.0228	0.1743	1.03	0.6635	0.9889	0.96	0.6293	0.9881
Poly(rC)-binding protein 1	PCBP1_HUMAN	Q15365	0.99	0.8567	0.9307	1.06	0.1585	0.9982	1.07	0.1733	0.9960	1.01	0.8685	0.9939	0.97	0.5482	0.9951	0.96	0.3903	0.9856	1.07	0.1379	0.6871	0.95	0.2869	0.5402	0.91	0.0252	0.9889	0.95	0.1898	0.9607
Poly(rC)-binding protein 2	PCBP2_HUMAN	Q15366	0.94	0.3670	0.6279	0.97	0.6984	0.9982	0.96	0.6185	0.9960	0.90	0.1356	0.9874	0.87	0.0583	0.9951	0.99	0.8919	0.9959	0.98	0.7417	0.9230	1.10	0.2210	0.4893	0.86	0.0391	0.9889	0.94	0.3979	0.9607
Polyadenylate-binding protein 1	PABP1_HUMAN	P11940	0.95	0.2926	0.5638	1.04	0.3620	0.9982	1.03	0.6250	0.9960	1.05	0.2616	0.9939	0.95	0.3124	0.9951	0.99	0.8084	0.9959	1.01	0.8509	0.9579	0.92	0.0849	0.2956	1.01	0.8726	0.9889	0.96	0.3702	0.9607
Polyadenylate-binding protein 4	PABP4_HUMAN	Q13310	1.06	0.0948	0.3539	0.98	0.6190	0.9982	1.03	0.5727	0.9960	1.00	0.9952	0.9986	1.05	0.1962	0.9951	1.00	0.9447	0.9959	1.00	0.9695	0.9937	1.05	0.2139	0.4807	1.05	0.1717	0.9889	1.06	0.1464	0.9607
Polymerase delta-interacting protein 2	PDIP2_HUMAN	Q9Y2S7	1.06	0.3525	0.6152	1.05	0.4080	0.9982	1.06	0.3994	0.9960	1.05	0.3595	0.9939	1.03	0.5769	0.9951	0.91	0.1983	0.9856	1.01	0.8496	0.9579	1.08	0.2271	0.4943	1.02	0.7056	0.9889	1.00	0.9746	0.9992
Polypyrimidine tract-binding protein 1	PTBP1_HUMAN	P26599	1.02	0.8064	0.9060	1.05	0.4208	0.9982	0.96	0.5399	0.9960	0.89	0.0723	0.9874	0.96	0.5109	0.9951	1.04	0.5827	0.9856	1.11	0.1116	0.6824	0.98	0.8230	0.9062	1.06	0.3654	0.9889	0.93	0.2852	0.9607
Polyribonucleotide nucleotidyltransferase 1, mitochondrial	PNPT1_HUMAN	Q8TCS8	1.11	0.1124	0.3748	1.07	0.2706	0.9982	1.03	0.6526	0.9960	1.08	0.2143	0.9939	0.99	0.8334	0.9951	0.96	0.6418	0.9856	0.96	0.5154	0.8536	1.09	0.2401	0.5050	1.06	0.3544	0.9889	1.01	0.9252	0.9938
PRA1 family protein 3	PRAF3_HUMAN	O75915	0.86	0.0091	0.2530	0.96	0.4925	0.9982	1.05	0.4445	0.9960	1.16	0.0077	0.9874	0.99	0.8295	0.9951	0.89	0.1067	0.9856	1.11	0.0923	0.6824	0.99	0.8347	0.9130	0.94	0.3055	0.9889	0.93	0.2280	0.9607
Prefoldin subunit 1	PFD1_HUMAN	O60925	1.08	0.2187	0.4914	1.05	0.4517	0.9982	1.07	0.3639	0.9960	0.97	0.6578	0.9939	1.02	0.7698	0.9951	0.96	0.6467	0.9856	0.93	0.2639	0.7187	1.02	0.7579	0.8671	1.01	0.9106	0.9889	0.99	0.9016	0.9938
Prefoldin subunit 2	PFD2_HUMAN	Q9UHV9	1.09	0.1615	0.4334	0.99	0.8344	0.9982	1.00	0.9585	0.9960	1.00	0.9368	0.9939	1.03	0.6166	0.9951	1.06	0.4731	0.9856	0.92	0.2169	0.7187	1.16	0.0328	0.2017	0.94	0.3603	0.9889	0.99	0.8282	0.9881
Prefoldin subunit 3	PFD3_HUMAN	P61758	1.10	0.1268	0.3932	0.97	0.6492	0.9982	1.05	0.5284	0.9960	0.98	0.7848	0.9939	1.01	0.9058	0.9951	0.99	0.9353	0.9959	0.94	0.3738	0.7708	1.11	0.1432	0.3877	0.97	0.6520	0.9889	1.00	0.9913	0.9992
Prefoldin subunit 4	PFD4_HUMAN	Q9NQP4	1.01	0.9110	0.9528	0.87	0.0632	0.9982	1.01	0.9360	0.9960	0.94	0.4473	0.9939	0.96	0.6206	0.9951	0.98	0.8329	0.9959	0.98	0.7800	0.9362	1.10	0.2704	0.5288	0.84	0.0212	0.9889	0.99	0.8708	0.9925
Prefoldin subunit 5	PFD5_HUMAN	Q99471	1.04	0.5471	0.7505	1.01	0.9385	0.9982	1.08	0.3109	0.9960	0.95	0.4651	0.9939	0.99	0.8469	0.9951	0.98	0.7899	0.9903	0.88	0.0733	0.6824	1.17	0.0373	0.2106	0.93	0.3330	0.9889	0.95	0.5033	0.9818
Prefoldin subunit 6	PFD6_HUMAN	O15212	1.15	0.0242	0.2873	1.09	0.1701	0.9982	1.09	0.2742	0.9960	1.06	0.3786	0.9939	1.06	0.4166	0.9951	0.99	0.9105	0.9959	0.94	0.3625	0.7668	1.07	0.3200	0.5613	1.00	0.9914	0.9984	0.98	0.8067	0.9881
Prelamin-A/C	LMNA_HUMAN	P02545	0.84	0.0491	0.2898	1.03	0.7364	0.9982	1.01	0.8919	0.9960	0.94	0.5042	0.9939	0.92	0.3783	0.9951	0.99	0.9307	0.9959	1.11	0.2832	0.7212	0.80	0.0325	0.2017	1.11	0.2586	0.9889	1.01	0.8767	0.9938
Prenylcysteine oxidase 1	PCYOX_HUMAN	Q9UHG3	1.09	0.2230	0.4953	1.09	0.2406	0.9982	1.05	0.5727	0.9960	1.01	0.8769	0.9939	1.01	0.8903	0.9951	0.89	0.2001	0.9856	0.95	0.4859	0.8365	1.01	0.8852	0.9377	0.99	0.9197	0.9889	1.06	0.4378	0.9607
Presequence protease, mitochondrial	PREP_HUMAN	Q5JRX3	1.25	0.0179	0.2873	1.03	0.7613	0.9982	0.78	0.0244	0.9960	0.99	0.9550	0.9939	1.09	0.3935	0.9951	1.20	0.1150	0.9856	0.90	0.3162	0.7400	1.01	0.9141	0.9536	1.10	0.3370	0.9889	1.10	0.3264	0.9607
Pro-neuropeptide Y	NPY_HUMAN	P01303	1.10	0.6555	0.8297	0.98	0.9309	0.9982	0.57	0.0205	0.9960	1.07	0.7366	0.9939	0.81	0.3444	0.9951	1.77	0.0297	0.9411	1.07	0.7627	0.9282	1.87	0.0079	0.1361	0.84	0.4435	0.9889	0.82	0.3628	0.9607
Probable 2-oxoglutarate dehydrogenase E1 component DHKTD1, mitocho	DHTK1_HUMAN	Q96HY7	1.11	0.2748	0.5458	0.98	0.7897	0.9982	1.08	0.4998	0.9960	0.93	0.4243	0.9939	0.98	0.8548	0.9951	0.95	0.6580	0.9856	0.90	0.3103	0.7378	1.18	0.1219	0.3555	0.95	0.5791	0.9889	1.05	0.6270	0.9881
Probable cysteine--tRNA ligase, mitochondrial	SYCM_HUMAN	Q9HA77	1.03	0.7394	0.8798	1.02	0.8486	0.9982	1.28	0.0182	0.9960	1.07	0.4651	0.9939	1.07	0.4799	0.9951	0.78	0.0265	0.9411	1.07	0.4707	0.8308	0.87	0.1583	0.4067	0.95	0.5642	0.9889	0.91	0.2816	0.9607
Probable D-lactate dehydrogenase, mitochondrial	LDHD_HUMAN	Q86WU2	1.19	0.0532	0.2939	1.06	0.5290	0.9982	0.83	0.0654	0.9960	0.97	0.7395	0.9939	1.07	0.4806	0.9951	1.20	0.0986	0.9856	0.97	0.7173	0.9164	0.98	0.8542	0.9202	1.24	0.0183	0.9889	1.25	0.0114	0.9304
Probable serine carboxypeptidase CPVL	CPVL_HUMAN	Q9H3G5	1.16	0.0367	0.2873	0.94	0.4236	0.9982	1.08	0.3625	0.9960	1.09	0.2329	0.9939	1.17	0.0369	0.9951	0.83	0.0371	0.9411	1.12	0.1408	0.6871	1.01	0.9459	0.9696	1.05	0.5324	0.9889	1.03	0.6662	0.9881
Profilin-1	PROF1_HUMAN	P07737	1.05	0.5620	0.7643	1.00	0.9978	0.9985	1.01	0.9137	0.9960	0.87	0.0954	0.9874	0.92	0.3207	0.9951	1.09	0.4102	0.9856	0.81	0.0182	0.6779	1.19	0.0580	0.2501	0.90	0.2270	0.9889	0.91	0.2362	0.9607
Profilin-2	PROF2_HUMAN	P35080	1.07	0.4235	0.6720	0.91	0.3125	0.9982	1.02	0.8616	0.9960	0.98	0.8609	0.9939	1.13	0.1839	0.9951	1.01	0.9220	0.9959	0.93	0.4549	0.8208	1.26	0.0199	0.1655	0.87	0.1345	0.9889	1.08	0.3857	0.9607
Programmed cell death 6-interacting protein	PDC6I_HUMAN	Q8WUM4	1.03	0.6225	0.8128	1.02	0.7512	0.9982	1.00	0.9496	0.9960	1.01	0.8734	0.9939	1.03	0.6410	0.9951	1.01	0.8550	0.9959	1.03	0.5516	0.8601	0.94	0.3205	0.5613	0.95	0.3238	0.9889	0.98	0.7675	0.9881
Programmed cell death protein 5	PDCD5_HUMAN	O14737	1.10	0.2876	0.5619	1.00	0.9667	0.9982	0.94	0.5688	0.9960	0.93	0.4170	0.9939	0.92	0.3776	0.9951	1.15	0.2279	0.9856	0.82	0.0368	0.6824	1.27	0.0205	0.1665	0.96	0.6517	0.9889	0.93	0.4648	0.9607
Progranulin	GRN_HUMAN	P28799	0.90	0.2386	0.5125	1.08	0.3938	0.9982	1.00	0.9889	0.9960	1.01	0.8892	0.9939	0.97	0.7320	0.9951	1.04	0.7618	0.9857	1.10	0.3248	0.7400	0.77	0.0114	0.1499	1.01	0.9187	0.9889	0.95	0.5772	0.9881
Prohibitin	PHB_HUMAN	P35232	0.97	0.6613	0.8357	1.05	0.4557	0.9982	0.96	0.5401	0.9960	1.06	0.3132	0.9939	1.06	0.3648	0.9951	0.99	0.8488	0.9959	1.06	0.3985	0.7880	0.95	0.4589	0.6728	1.07	0.2891	0.9889	1.07	0.2830	0.9607
Prohibitin-2	PHB2_HUMAN	Q99623	0.98	0.7654	0.8882	1.05	0.4125	0.9982	1.00	0.9695	0.9960	1.05	0.4480	0.9939	1.07	0.2934	0.9951	0.97	0.6546	0.9856	1.09	0.2053	0.7149	0.94	0.3690	0.5979	1.02	0.8006	0.9889	1.05	0.4726	0.9607
Prolargin	PRELP_HUMAN	P51888	0.67	0.0732	0.3245	1.04	0.8719	0.9982	0.65	0.0942	0.9960	0.97	0.8786	0.9939	0.83	0.4218	0.9951	1.31	0.3251	0.9856	1.16	0.5317	0.8567	0.55	0.0138	0.1581	1.12	0.6115	0.9889	1.04	0.8491	0.9881
Prolow-density lipoprotein receptor-related protein 1	LRP1_HUMAN	Q07954	0.83	0.0779	0.3281	1.04	0.7253	0.9982	1.01	0.9096	0.9960	1.04	0.7095	0.9939	0.93	0.5176	0.9951	1.00	0.9967	0.9992	1.23	0.0552	0.6824	0.75	0.0104	0.1452	0.92	0.4304	0.9889	0.94	0.5245	0.9846
Prolyl 4-hydroxylase subunit alpha-1	P4HA1_HUMAN	P13674	0.95	0.4047	0.6612	0.98	0.7640	0.9982	1.05	0.5006	0.9960	1.06	0.4052	0.9939	1.00	0.9996	0.9996	0.95	0.5022	0.9856	1.08	0.2577	0.7187	0.86	0.0486	0.2330	0.91	0.1872	0.9889	0.96	0.5051	0.9818
Prolyl endopeptidase	PPCE_HUMAN	P48147	1.11	0.2513	0.5219	0.94	0.4767	0.9982	0.98	0.8577	0.9960	0.99	0.8923	0.9939	1.06	0.5336	0.9951	1.14	0.2217	0.9856	0.96	0.6469	0.8897	1.18	0.0878	0.2995	0.93	0.4447	0.9889	0.99	0.8824	0.9938
Propionyl-CoA carboxylase alpha chain, mitochondrial	PCCA_HUMAN	P05165	1.16	0.0561	0.2942	1.06	0.4317	0.9982	1.06	0.5024	0.9960	1.04	0.6124	0.9939	1.00	0.9987	0.9996	0.96	0.6885	0.9856	0.91	0.2478	0.7187	1.17	0.0616	0.2558	1.08	0.3278	0.9889	1.04	0.5694	0.9881
Propionyl-CoA carboxylase beta chain, mitochondrial	PCCB_HUMAN	P05166	1.15	0.0977	0.3563	1.08	0.4001	0.9982	1.06	0.5274	0.9960	1.00	0.9807	0.9967	0.97	0.7233	0.9951	0.97	0.7875	0.9903	0.91	0.2740	0.7196	1.17	0.0898	0.3034	1.07	0.4347	0.9889	1.02	0.8592	0.9881
Prosaposin	SAP_HUMAN	P07602	1.07	0.3891	0.6465	1.10	0.2172	0.9982	0.98	0.7935	0.9960	0.90	0.1357	0.9874	1.00	0.9730	0.9951	1.01	0.9173	0.9959	0.97	0.6720	0.8943	0.94	0.4640	0.6758	0.91	0.2118	0.9889	0.94	0.3740	0.9607
Prostacyclin synthase	PTGIS_HUMAN	Q16647	0.93	0.5261	0.7379	1.10	0.3797	0.9982	0.99	0.9498	0.9960	1.23	0.0472	0.9874	1.06	0.6182	0.9951	0.91	0.5032	0.9856	1.34	0.0093	0.6779	0.74	0.0120	0.1530	0.99	0.9224	0.9889	0.95	0.6120	0.9881
Prostaglandin E synthase 2	PGES2_HUMAN	Q9H7Z7	1.14	0.0248	0.2873	1.09	0.1370	0.9982	0.98	0.7444	0.9960	1.05	0.3902	0.9939	1.09	0.1835	0.9951	0.96	0.5611	0.9856	0.99	0.9120	0.9848	0.99	0.8907	0.9398	1.06	0.3791	0.9889	1.10	0.1180	0.9607
Prostaglandin E synthase 3	TEBP_HUMAN	Q15185	0.99	0.8315	0.9186	1.00	0.9460	0.9982	0.89	0.1188	0.9960	0.90	0.1101	0.9874	0.89	0.0932	0.9951	1.11	0.1777	0.9856	0.91	0.1881	0.7149	0.97	0.6350	0.7937	0.92	0.2316	0.9889	0.91	0.1603	0.9607
Prostaglandin F2 receptor negative regulator	FPRP_HUMAN	Q9P2B2	0.80	0.0271	0.2873	1.03	0.7726	0.9982	1.19	0.1398	0.9960	1.15	0.1698	0.9874	1.01	0.9559	0.9951	0.81	0.0905	0.9856	1.00	0.9656	0.9931	0.73	0.0054	0.1317	0.91	0.3667	0.9889	0.89	0.2595	0.9607
Prostaglandin reductase 1	PTGR1_HUMAN	Q14914	1.10	0.4222	0.6720	1.04	0.7228	0.9982	1.04	0.7856	0.9960	0.88	0.2684	0.9939	1.03	0.8261	0.9951	1.03	0.8432	0.9959	0.96	0.7631	0.9282	1.04	0.7394	0.8574	0.87	0.2474	0.9889	0.99	0.9179	0.9938
Prostaglandin reductase 2	PTGR2_HUMAN	Q8N8N7	1.28	0.0286	0.2873	0.95	0.6601	0.9982	1.10	0.4881	0.9960	0.90	0.3844	0.9939	1.09	0.4978	0.9951	0.96	0.7654	0.9857	1.10	0.4260	0.8072	1.14	0.3020	0.5500	0.96	0.7170	0.9889	1.00	0.9705	0.9992
Prostaglandin-H2 D-isomerase	PTGDS_HUMAN	P41222	1.23	0.0276	0.2873	1.05	0.6444	0.9982	0.90	0.3480	0.9960	0.96	0.6824	0.9939	1.07	0.5101	0.9951	1.15	0.2367	0.9856	0.99	0.8872	0.9753	1.11	0.3349	0.5707	1.06	0.5689	0.9889	1.05	0.6214	0.9881
Proteasomal ubiquitin receptor ADRM1	ADRM1_HUMAN	Q16186	0.95	0.4857	0.7203	1.05	0.4998	0.9982	1.07	0.4268	0.9960	0.99	0.8864	0.9939	0.92	0.2526	0.9951	0.99	0.9361	0.9959	1.00	0.9776	0.9952	1.12	0.1841	0.4384	0.96	0.6029	0.9889	0.94	0.4010	0.9607
Proteasome activator complex subunit 1	PSME1_HUMAN	Q06323	1.06	0.3244	0.5917	1.11	0.0739	0.9982	1.00	0.9756	0.9960	1.04	0.4688	0.9939	1.00	0.9654	0.9951	1.06	0.4592	0.9856	0.99	0.9281	0.9880	0.93	0.2407	0.5050	0.96	0.5467	0.9889	0.98	0.7465	0.9881
Proteasome activator complex subunit 2	PSME2_HUMAN	Q9UL46	1.14	0.0632	0.3074	1.20	0.0081	0.9982	1.00	0.9812	0.9960	1.02	0.7900	0.9939	1.01	0.8963	0.9951	1.04	0.6825	0.9856	0.97	0.6921	0.9047	0.95	0.4867	0.6890	0.94	0.3750	0.9889	0.99	0.9005	0.9938
Proteasome subunit alpha type-1	PSA1_HUMAN	P25786	1.03	0.5017	0.7246	1.04	0.3877	0.9982	1.02	0.6677	0.9960	1.03	0.5713	0.9939	0.97	0.6082	0.9951	1.03	0.6274	0.9856	1.00	0.9438	0.9903	1.05	0.4098	0.6358	0.95	0.2817	0.9889	0.98	0.5981	0.9881
Proteasome subunit alpha type-2	PSA2_HUMAN	P25787	1.03	0.6740	0.8415	1.02	0.7515	0.9982	1.13	0.0885	0.9960	1.06	0.3435	0.9939	0.98	0.7384	0.9951	0.97	0.6697	0.9856	1.03	0.7064	0.9121	1.18	0.0137	0.1576	0.98	0.7776	0.9889	0.96	0.4664	0.9607
Proteasome subunit alpha type-3	PSA3_HUMAN	P25788	1.00	0.9162	0.9536	1.07	0.1507	0.9982	1.03	0.5361	0.9960	1.03	0.5500	0.9939	0.95	0.2951	0.9951	1.00	0.9456	0.9959	0.93	0.1535	0.6871	1.09	0.0945	0.3121	0.95	0.2922	0.9889	0.95	0.3099	0.9607
Proteasome subunit alpha type-4	PSA4_HUMAN	P25789	1.04	0.5104	0.7292	1.06	0.3246	0.9982	1.03	0.6901	0.9960	1.02	0.7154	0.9939	0.96	0.4423	0.9951	1.00	0.9971	0.9992	0.91	0.0818	0.6824	1.14	0.0257	0.1832	0.97	0.5413	0.9889	0.96	0.5010	0.9818
Proteasome subunit alpha type-5	PSA5_HUMAN	P28066	1.10	0.0876	0.3401	1.11	0.0512	0.9982	1.09	0.1717	0.9960	1.08	0.1440	0.9874	1.01	0.8924	0.9951	0.96	0.5618	0.9856	0.95	0.4204	0.8021	1.01	0.8543	0.9202	0.94	0.2919	0.9889	1.00	0.9517	0.9938
Proteasome subunit alpha type-6	PSA6_HUMAN	P60900	1.06	0.3235	0.5917	1.06	0.2870	0.9982	1.13	0.0630	0.9960	1.03	0.6137	0.9939	0.96	0.5311	0.9951	0.91	0.1842	0.9856	0.94	0.3053	0.7378	1.05	0.4277	0.6497	0.93	0.2084	0.9889	0.96	0.4378	0.9607
Proteasome subunit alpha type-7	PSA7_HUMAN	O14818	1.09	0.0905	0.3428	1.05	0.3726	0.9982	1.02	0.6893	0.9960	1.05	0.3222	0.9939	1.05	0.3814	0.9951	1.01	0.9045	0.9959	1.01	0.7977	0.9431	1.01	0.9239	0.9571	0.97	0.5474	0.9889	1.03	0.5704	0.9881
Proteasome subunit beta type-1	PSB1_HUMAN	P20618	1.08	0.1141	0.3748	1.05	0.2854	0.9982	1.04	0.5039	0.9960	1.08	0.1131	0.9874	1.01	0.8136	0.9951	0.98	0.7781	0.9897	0.98	0.7496	0.9272	1.03	0.5933	0.7648	1.01	0.8522	0.9889	1.00	0.9318	0.9938
Proteasome subunit beta type-10	PSB10_HUMAN	P40306	1.04	0.5506	0.7540	1.07	0.2851	0.9982	0.95	0.4839	0.9960	1.08	0.2367	0.9939	1.00	0.9441	0.9951	1.13	0.1250	0.9856	1.02	0.7743	0.9323	0.99	0.8457	0.9170	1.01	0.8623	0.9889	0.94	0.3635	0.9607
Proteasome subunit beta type-2	PSB2_HUMAN	P49721	1.05	0.5096	0.7292	1.05	0.5054	0.9982	1.06	0.4996	0.9960	1.03	0.7056	0.9939	1.00	0.9643	0.9951	0.97	0.7369	0.9857	0.96	0.5952	0.8646	1.16	0.0700	0.2711	0.98	0.8198	0.9889	1.03	0.6947	0.9881
Proteasome subunit beta type-3	PSB3_HUMAN	P49720	1.01	0.8713	0.9356	1.08	0.2333	0.9982	1.01	0.9083	0.9960	1.00	0.9514	0.9939	0.91	0.1406	0.9951	1.02	0.8315	0.9959	0.95	0.4286	0.8098	1.16	0.0388	0.2155	1.01	0.8455	0.9889	0.98	0.6974	0.9881
Proteasome subunit beta type-4	PSB4_HUMAN	P28070	1.12	0.1370	0.4005	1.11	0.1601	0.9982	1.02	0.8273	0.9960	1.08	0.3000	0.9939	0.99	0.9446	0.9951	0.98	0.8156	0.9959	1.12	0.1568	0.6871	1.02	0.8241	0.9067	0.96	0.6369	0.9889	0.98	0.8141	0.9881
Proteasome subunit beta type-5	PSB5_HUMAN	P28074	1.15	0.0338	0.2873	1.09	0.2008	0.9982	1.03	0.7387	0.9960	1.01	0.8284	0.9939	1.00	0.9884	0.9967	1.01	0.9112	0.9959	0.91	0.1903	0.7149	1.09	0.2692	0.5288	1.00	0.9711	0.9976	1.06	0.4110	0.9607
Proteasome subunit beta type-6	PSB6_HUMAN	P28072	1.09	0.1239	0.3890	1.07	0.2573	0.9982	1.01	0.9118	0.9960	1.06	0.3416	0.9939	0.99	0.9257	0.9951	1.03	0.7005	0.9857	0.97	0.6554	0.8915	1.07	0.3198	0.5613	1.00	0.9740	0.9976	1.02	0.7561	0.9881
Proteasome subunit beta type-7	PSB7_HUMAN	Q99436	1.08	0.1548	0.4236	1.10	0.0990	0.9982	1.03	0.6968	0.9960	1.05	0.3978	0.9939	0.98	0.7823	0.9951	0.98	0.7974	0.9943	0.96	0.4543	0.8208	1.04	0.5611	0.7423	0.99	0.8697	0.9889	0.99	0.9261	0.9938
Proteasome subunit beta type-9	PSB9_HUMAN	P28065	0.92	0.3741	0.6312	1.10	0.2890	0.9982	0.92	0.4070	0.9960	0.98	0.8560	0.9939	0.97	0.7481	0.9951	1.18	0.1512	0.9856	1.16	0.1263	0.6868	0.82	0.0586	0.2503	0.85	0.0749	0.9889	0.88	0.1589	0.9607
Protein ABHD11	ABHDB_HUMAN	Q8NFV4	1.18	0.0380	0.2873	1.08	0.3233	0.9982	1.00	0.9789	0.9960	0.93	0.4012	0.9939	1.02	0.7885	0.9951	1.04	0.7274	0.9857	0.90	0.2395	0.7187	1.10	0.2868	0.5402	0.98	0.8084	0.9889	1.07	0.4139	0.9607
Protein AMBP	AMBP_HUMAN	P02760	0.87	0.2121	0.4843	1.26	0.0444	0.9982	1.13	0.3493	0.9960	1.04	0.7382	0.9939	0.94	0.6038	0.9951	0.97	0.8370	0.9959	1.05	0.7142	0.9154	0.86	0.2527	0.5157	1.21	0.1112	0.9889	0.95	0.6481	0.9881
Protein canopy homolog 2	CNPY2_HUMAN	Q9Y2B0	1.01	0.8964	0.9449	1.09	0.2196	0.9982	1.03	0.7136	0.9960	1.01	0.8603	0.9939	0.97	0.6357	0.9951	0.98	0.8621	0.9959	1.11	0.1812	0.7142	0.90	0.1692	0.4217	0.88	0.0808	0.9889	0.92	0.2242	0.9607
Protein canopy homolog 3	CNPY3_HUMAN	Q9BT09	0.88	0.1227	0.3867	1.12	0.1834	0.9982	1.12	0.2400	0.9960	1.04	0.6152	0.9939	0.93	0.3977	0.9951	0.90	0.3266	0.9856	1.14	0.1462	0.6871	0.80	0.0218	0.1727	0.96	0.6781	0.9889	0.90	0.2368	0.9607
Protein CutA	CUTA_HUMAN	O60888	1.12	0.1853	0.4609	1.02	0.7982	0.9982	0.96	0.6679	0.9960	0.93	0.4033	0.9939	0.99	0.9423	0.9951	1.10	0.3570	0.9856	0.94	0.4826	0.8361	1.12	0.2219	0.4900	1.03	0.7121	0.9889	0.98	0.7674	0.9881
Protein disulfide isomerase CRELD1	CREL1_HUMAN	Q96HD1	0.96	0.4574	0.6991	1.08	0.1408	0.9982	1.14	0.0395	0.9960	1.03	0.5726	0.9939	0.95	0.4049	0.9951	0.85	0.0116	0.9411	0.98	0.7841	0.9373	0.86	0.0115	0.1499	0.95	0.4012	0.9889	0.99	0.8406	0.9881
Protein disulfide-isomerase	PDIA1_HUMAN	P07237	0.98	0.7365	0.8780	0.92	0.1371	0.9982	1.06	0.3833	0.9960	0.98	0.6610	0.9939	0.95	0.3804	0.9951	0.98	0.8285	0.9959	0.99	0.8770	0.9720	1.12	0.0776	0.2849	0.92	0.1679	0.9889	0.89	0.0482	0.9607
Protein disulfide-isomerase A3	PDIA3_HUMAN	P30101	0.90	0.0749	0.3265	0.97	0.6109	0.9982	1.06	0.4166	0.9960	1.04	0.5468	0.9939	0.96	0.5517	0.9951	0.93	0.2988	0.9856	1.11	0.1089	0.6824	0.91	0.1546	0.4044	0.96	0.5538	0.9889	0.92	0.1600	0.9607
Protein disulfide-isomerase A4	PDIA4_HUMAN	P13667	0.91	0.2279	0.5015	0.99	0.8617	0.9982	1.06	0.5052	0.9960	1.05	0.5291	0.9939	0.98	0.7831	0.9951	0.98	0.7951	0.9928	1.14	0.1149	0.6824	0.89	0.1602	0.4096	0.89	0.1500	0.9889	0.90	0.1579	0.9607
Protein disulfide-isomerase A6	PDIA6_HUMAN	Q15084	0.94	0.3009	0.5713	1.00	0.9502	0.9982	1.04	0.6097	0.9960	1.08	0.2273	0.9939	1.01	0.8723	0.9951	0.97	0.6598	0.9856	1.13	0.0629	0.6824	0.95	0.4479	0.6635	1.00	0.9726	0.9976	0.94	0.3028	0.9607
Protein disulfide-isomerase TMX3	TMX3_HUMAN	Q96JJ7	0.92	0.1013	0.3610	0.94	0.2364	0.9982	1.06	0.3276	0.9960	1.06	0.2182	0.9939	0.97	0.6030	0.9951	0.92	0.1908	0.9856	0.99	0.9231	0.9864	1.04	0.5008	0.6993	1.01	0.8589	0.9889	0.98	0.6257	0.9881
Protein enabled homolog	ENAH_HUMAN	Q8N8S7	0.93	0.2892	0.5630	0.92	0.2759	0.9982	1.00	0.9784	0.9960	1.11	0.1456	0.9874	1.00	0.9640	0.9951	1.03	0.7059	0.9857	1.04	0.6267	0.8830	0.99	0.8927	0.9399	0.96	0.6126	0.9889	1.01	0.8468	0.9881
Protein FAM114A2	F1142_HUMAN	Q9NRY5	1.04	0.4814	0.7173	0.96	0.4066	0.9982	1.00	0.9797	0.9960	1.02	0.6612	0.9939	1.06	0.2636	0.9951	0.99	0.9326	0.9959	1.01	0.8331	0.9513	1.07	0.2293	0.4943	0.94	0.2336	0.9889	1.02	0.7291	0.9881

Protein FAM136A	F136A_HUMAN	Q96C01	1.14	0.0443	0.2877	1.05	0.4353	0.9982	0.85	0.0293	0.9960	1.02	0.7695	0.9939	1.06	0.3532	0.9951	1.23	0.0081	0.9411	0.98	0.7475	0.9272	1.11	0.1376	0.3791	1.02	0.7280	0.9889	1.00	0.9495	0.9938
Protein FAM162A	F162A_HUMAN	Q96A26	1.03	0.7276	0.8741	1.05	0.4738	0.9982	1.02	0.7872	0.9960	1.11	0.1357	0.9874	1.08	0.2878	0.9951	0.92	0.3539	0.9856	1.04	0.6353	0.8887	0.85	0.0428	0.2201	1.06	0.4294	0.9889	1.12	0.1208	0.9607
Protein FAM177A1	F177A_HUMAN	Q8N128	0.90	0.2217	0.4949	0.99	0.9495	0.9982	1.14	0.1708	0.9960	1.13	0.1487	0.9874	1.01	0.9143	0.9951	0.84	0.0845	0.9856	0.99	0.9378	0.9903	0.97	0.7433	0.8595	1.01	0.9010	0.9889	1.08	0.3707	0.9607
Protein FAM3C	FAM3C_HUMAN	Q92520	0.87	0.0539	0.2939	0.97	0.6694	0.9982	0.93	0.4030	0.9960	0.84	0.0149	0.9874	0.89	0.1235	0.9951	1.09	0.3420	0.9856	0.87	0.0879	0.6824	0.90	0.1912	0.4497	1.05	0.5506	0.9889	0.93	0.3067	0.9607
Protein flightless-1 homolog	FLII_HUMAN	Q13045	0.86	0.0198	0.2873	0.88	0.0452	0.9982	1.02	0.8122	0.9960	0.98	0.7141	0.9939	0.99	0.9167	0.9951	0.96	0.6521	0.9856	0.96	0.5852	0.8645	0.96	0.6054	0.7758	0.99	0.9144	0.9889	1.00	0.9910	0.9992
Protein kinase C and casein kinase substrate in neurons protein 2	PACN2_HUMAN	Q9UNF0	0.95	0.4112	0.6642	1.01	0.8852	0.9982	1.15	0.0368	0.9960	0.98	0.6757	0.9939	0.96	0.4544	0.9951	0.84	0.0170	0.9411	1.03	0.6466	0.8897	0.96	0.5546	0.7387	0.99	0.8887	0.9889	0.96	0.5398	0.9881
Protein kinase C and casein kinase substrate in neurons protein 3	PACN3_HUMAN	Q9UKS6	0.91	0.3017	0.5721	0.87	0.1056	0.9982	1.04	0.7002	0.9960	0.99	0.8718	0.9939	0.92	0.3624	0.9951	0.85	0.1401	0.9856	1.00	0.9902	0.9992	1.05	0.6121	0.7814	0.99	0.9423	0.9922	0.95	0.5500	0.9881
Protein NDRG1	NDRG1_HUMAN	Q92597	0.99	0.9137	0.9536	1.12	0.0682	0.9982	1.04	0.5447	0.9960	1.07	0.2463	0.9939	0.93	0.2853	0.9951	0.99	0.9293	0.9959	1.00	0.9732	0.9937	0.90	0.1301	0.3676	0.93	0.2307	0.9889	0.97	0.6081	0.9881
Protein NDRG2	NDRG2_HUMAN	Q9UN36	1.16	0.0764	0.3266	1.02	0.8326	0.9982	0.94	0.5089	0.9960	0.92	0.2988	0.9939	1.01	0.9038	0.9951	1.14	0.2027	0.9856	0.90	0.2410	0.7187	1.33	0.0019	0.0815	1.00	0.9775	0.9976	1.07	0.4051	0.9607
Protein NDRG4	NDRG4_HUMAN	Q9ULP0	1.13	0.2015	0.4813	0.97	0.7363	0.9982	1.01	0.9250	0.9960	0.96	0.6644	0.9939	1.03	0.7240	0.9951	0.97	0.8238	0.9959	0.88	0.1969	0.7149	1.26	0.0231	0.1743	0.96	0.6667	0.9889	1.11	0.2779	0.9607
Protein Niban	NIBA1_HUMAN	Q9BZQ8	1.04	0.5842	0.7830	0.91	0.1603	0.9982	1.03	0.7004	0.9960	0.99	0.8388	0.9939	0.98	0.7845	0.9951	0.99	0.9021	0.9959	0.94	0.3648	0.7673	1.30	0.0004	0.0621	0.91	0.1760	0.9889	0.94	0.3841	0.9607
Protein NipSnap homolog 1	NIPS1_HUMAN	Q9BPW8	1.06	0.4819	0.7174	1.15	0.0818	0.9982	1.09	0.3326	0.9960	1.07	0.3861	0.9939	1.02	0.8478	0.9951	0.86	0.1391	0.9856	1.16	0.0822	0.6824	0.96	0.6846	0.8231	0.94	0.4722	0.9889	0.98	0.7929	0.9881
Protein NipSnap homolog 2	NIPS2_HUMAN	O75323	1.07	0.3502	0.6124	1.04	0.6086	0.9982	1.02	0.7985	0.9960	0.97	0.6920	0.9939	1.00	0.9717	0.9951	0.95	0.5839	0.9856	0.89	0.1631	0.6896	1.17	0.0650	0.2631	1.06	0.4394	0.9889	1.03	0.7100	0.9881
Protein NipSnap homolog 3A	NPS3A_HUMAN	Q9UFN0	1.15	0.1069	0.3670	1.06	0.5349	0.9982	1.00	0.9881	0.9960	0.96	0.6587	0.9939	0.98	0.8163	0.9951	1.01	0.9216	0.9959	0.99	0.8900	0.9753	1.21	0.0460	0.2280	1.04	0.6285	0.9889	0.93	0.4292	0.9607
Protein NipSnap homolog 3B	NPS3B_HUMAN	Q9BS92	1.08	0.6538	0.8297	0.95	0.7807	0.9982	0.89	0.5376	0.9960	1.03	0.8712	0.9939	0.88	0.4421	0.9951	1.05	0.8168	0.9959	1.06	0.7488	0.9272	1.47	0.0348	0.2017	1.07	0.6975	0.9889	0.92	0.6137	0.9881
Protein O-glucosyltransferase 3	PLGT3_HUMAN	Q7Z4H8	1.03	0.7629	0.8882	1.15	0.1219	0.9982	0.97	0.8018	0.9960	1.04	0.6493	0.9939	1.03	0.7325	0.9951	1.04	0.7367	0.9857	1.11	0.2726	0.7196	0.79	0.0210	0.1683	0.95	0.6214	0.9889	1.05	0.5803	0.9881
Protein phosphatase 1 regulatory subunit 12A	MYPT1_HUMAN	O14974	0.98	0.7434	0.8802	1.09	0.0928	0.9982	1.02	0.7289	0.9960	1.09	0.0981	0.9874	1.00	0.9290	0.9951	1.04	0.5325	0.9856	0.96	0.4349	0.8109	0.94	0.2963	0.5464	1.07	0.2438	0.9889	1.03	0.5590	0.9881
Protein phosphatase 1 regulatory subunit 12B	MYPT2_HUMAN	O60237	1.05	0.4371	0.6833	1.01	0.9254	0.9982	0.95	0.4950	0.9960	1.02	0.7618	0.9939	0.99	0.8395	0.9951	1.00	0.9789	0.9982	0.92	0.2064	0.7149	1.02	0.7133	0.8360	1.07	0.2681	0.9889	1.06	0.3291	0.9607
Protein phosphatase 1 regulatory subunit 12C	PP12C_HUMAN	Q9BZL4	0.88	0.0880	0.3401	0.95	0.4756	0.9982	1.18	0.0442	0.9960	1.01	0.8771	0.9939	0.91	0.2264	0.9951	0.80	0.0124	0.9411	0.88	0.0842	0.6824	1.04	0.6045	0.7753	1.07	0.3712	0.9889	0.99	0.9022	0.9938
Protein phosphatase 1 regulatory subunit 7	PP1R7_HUMAN	Q15435	0.99	0.9164	0.9536	1.02	0.8409	0.9982	1.09	0.3864	0.9960	0.94	0.4648	0.9939	0.90	0.2517	0.9951	0.93	0.4840	0.9856	0.95	0.5811	0.8633	1.16	0.1231	0.3567	0.84	0.0429	0.9889	0.86	0.0717	0.9607
Protein phosphatase inhibitor 2	IPP2_HUMAN	P41236	1.02	0.7963	0.9014	0.91	0.2487	0.9982	0.96	0.6551	0.9960	0.91	0.2438	0.9939	1.03	0.6912	0.9951	1.20	0.0675	0.9641	0.95	0.5387	0.8573	1.03	0.7122	0.8360	0.94	0.4817	0.9889	0.98	0.7759	0.9881
Protein preY, mitochondrial	PREY_HUMAN	Q96I23	1.19	0.0243	0.2873	0.95	0.5627	0.9982	0.89	0.2184	0.9960	1.01	0.9343	0.9939	1.07	0.4328	0.9951	1.10	0.3551	0.9856	0.89	0.1557	0.6871	1.23	0.0176	0.1636	1.05	0.5867	0.9889	1.17	0.0536	0.9607
Protein PRRC1	PRRC1_HUMAN	Q96M27	1.01	0.8854	0.9400	1.05	0.3469	0.9982	1.00	0.9498	0.9960	1.04	0.4850	0.9939	1.05	0.3706	0.9951	0.97	0.6905	0.9856	1.07	0.2451	0.7187	0.90	0.0715	0.2736	0.92	0.1429	0.9889	1.01	0.8492	0.9881
Protein quaking	QKI_HUMAN	Q96PU8	0.94	0.2606	0.5311	0.96	0.4493	0.9982	1.02	0.7888	0.9960	0.91	0.0507	0.9874	0.89	0.0340	0.9951	0.99	0.8398	0.9959	1.01	0.8301	0.9496	1.07	0.2484	0.5136	0.96	0.4429	0.9889	0.91	0.0573	0.9607
Protein S100-A1	S10A1_HUMAN	P23297	1.11	0.5528	0.7563	1.05	0.7809	0.9982	0.91	0.6584	0.9960	0.79	0.1892	0.9939	0.72	0.0776	0.9951	1.22	0.3625	0.9856	0.63	0.0114	0.6779	1.73	0.0044	0.1246	0.98	0.9316	0.9904	0.86	0.3991	0.9607
Protein S100-A10	S10AA_HUMAN	P60903	0.85	0.0395	0.2873	0.97	0.7467	0.9982	1.04	0.6489	0.9960	1.02	0.7560	0.9939	0.88	0.1342	0.9951	1.00	0.9880	0.9992	1.05	0.5846	0.8645	1.02	0.8642	0.9252	1.00	0.9823	0.9976	0.90	0.1762	0.9607
Protein S100-A11	S10AB_HUMAN	P31949	0.86	0.1387	0.4015	1.13	0.2423	0.9982	0.93	0.5620	0.9960	1.07	0.5439	0.9939	0.93	0.5150	0.9951	1.13	0.3572	0.9856	1.08	0.4694	0.8297	0.83	0.1008	0.3232	0.96	0.6935	0.9889	0.91	0.3521	0.9607
Protein S100-A13	S10AD_HUMAN	Q99584	0.97	0.6906	0.8506	0.99	0.8822	0.9982	0.99	0.9347	0.9960	0.92	0.3554	0.9939	0.91	0.3350	0.9951	1.01	0.9174	0.9959	0.80	0.0167	0.6779	1.03	0.7917	0.8865	0.90	0.2705	0.9889	0.87	0.1279	0.9607
Protein S100-A4	S10A4_HUMAN	P26447	0.95	0.6694	0.8391	0.97	0.8020	0.9982	0.93	0.5778	0.9960	1.02	0.8533	0.9939	0.98	0.8651	0.9951	1.15	0.3282	0.9856	1.19	0.1467	0.6871	0.78	0.0461	0.2280	0.91	0.4215	0.9889	0.96	0.7311	0.9881
Protein S100-A6	S10A6_HUMAN	P06703	0.98	0.7364	0.8780	1.02	0.7828	0.9982	1.17	0.0477	0.9960	1.03	0.6524	0.9939	0.96	0.5578	0.9951	0.85	0.0537	0.9631	0.94	0.3857	0.7810	0.94	0.4070	0.6337	0.94	0.3489	0.9889	0.97	0.6069	0.9881
Protein S100-A7	S10A7_HUMAN	P31151	1.16	0.4908	0.7219	1.88	0.0028	0.9982	1.32	0.2732	0.9960	1.59	0.0303	0.9874	1.23	0.3601	0.9951	0.78	0.3467	0.9856	1.29	0.2762	0.7212	0.79	0.3312	0.5692	1.23	0.3481	0.9889	0.98	0.9087	0.9938
Protein S100-A8	S10A8_HUMAN	P05109	0.90	0.6075	0.7989	1.37	0.1376	0.9982	1.86	0.0106	0.9960	1.05	0.8183	0.9939	0.88	0.5548	0.9951	0.51	0.0084	0.9411	1.21	0.4027	0.7880	0.86	0.5388	0.7261	0.82	0.3713	0.9889	0.73	0.1413	0.9607
Protein S100-A9	S10A9_HUMAN	P06702	0.86	0.4695	0.7091	1.25	0.2652	0.9982	1.61	0.0374	0.9960	1.11	0.6107	0.9939	0.91	0.6589	0.9951	0.60	0.0349	0.9411	1.15	0.5109	0.8517	0.83	0.3971	0.6232	0.83	0.3695	0.9889	0.75	0.1574	0.9607
Protein SCO1 homolog, mitochondrial	SCO1_HUMAN	O75880	1.08	0.2136	0.4853	1.02	0.8014	0.9982	0.96	0.5527	0.9960	1.09	0.1418	0.9874	1.16	0.0176	0.9951	1.03	0.7435	0.9857	0.93	0.2509	0.7187	0.93	0.3262	0.5681	1.03	0.6797	0.9889	1.17	0.0085	0.9304
Protein SEC13 homolog	SEC13_HUMAN	P55735	0.91	0.1319	0.3958	1.00	0.9749	0.9982	1.08	0.3024	0.9960	1.00	0.9759	0.9961	0.89	0.0729	0.9951	0.95	0.5310	0.9856	0.94	0.3594	0.7644	0.88	0.0540	0.2446	0.92	0.2057	0.9889	0.90	0.0769	0.9607
Protein SET	SET_HUMAN	Q01105	1.04	0.6874	0.8496	0.88	0.1664	0.9982	1.07	0.4990	0.9960	0.93	0.4049	0.9939	1.02	0.8571	0.9951	1.00	0.9755	0.9967	1.09	0.3956	0.7877	1.08	0.4494	0.6646	0.86	0.1046	0.9889	0.92	0.3636	0.9607
Protein TFG	TFG_HUMAN	Q92734	1.01	0.8319	0.9186	0.97	0.4829	0.9982	1.03	0.6629	0.9960	1.02	0.6275	0.9939	1.00	0.9693	0.9951	0.99	0.8565	0.9959	0.96	0.4298	0.8104	1.10	0.0778	0.2849	0.97	0.5701	0.9889	0.98	0.6837	0.9881
Protein transport protein Sec23A	SC23A_HUMAN	Q15436	1.01	0.9331	0.9615	0.96	0.5808	0.9982	1.10	0.2877	0.9960	1.03	0.6747	0.9939	1.06	0.5037	0.9951	0.89	0.2384	0.9856	0.99	0.9243	0.9871	0.85	0.0497	0.2366	1.02	0.7645	0.9889	1.06	0.4133	0.9607
Protein transport protein Sec31A	SC31A_HUMAN	O94979	0.99	0.7568	0.8867	0.96	0.4230	0.9982	1.03	0.6391	0.9960	1.01	0.7982	0.9939	0.97	0.5981	0.9951	1.01	0.8819	0.9959	1.04	0.4318	0.8109	1.06	0.2897	0.5428	0.98	0.7309	0.9889	0.91	0.0317	0.9607
Protein unc-45 homolog B	UN45B_HUMAN	Q8IWX7	1.01	0.9053	0.9497	1.00	0.9751	0.9982	1.02	0.8424	0.9960	1.11	0.1398	0.9874	0.98	0.7626	0.9951	1.03	0.7750	0.9875	0.93	0.3659	0.7673	1.14	0.1027	0.3273	1.03	0.6966	0.9889	0.97	0.6779	0.9881
Protein-glutamine gamma-glutamyltransferase 2	TGM2_HUMAN	P21980	0.96	0.6519	0.8297	1.00	0.9703	0.9982	0.91	0.3860	0.9960	0.98	0.8688	0.9939	0.89	0.2499	0.9951	1.07	0.5760	0.9856	0.99	0.9429	0.9903	0.90	0.3470	0.5798	1.23	0.0385	0.9889	1.11	0.3104	0.9607
Protein-L-isoaspartate(D-aspartate) O-methyltransferase	PIMT_HUMAN	P22061	1.11	0.0670	0.3105	0.97	0.6402	0.9982	0.97	0.6222	0.9960	0.97	0.6207	0.9939	1.02	0.7182	0.9951	1.08	0.2919	0.9856	0.93	0.2001	0.7149	1.19	0.0050	0.1313	0.95	0.3732	0.9889	1.04	0.4515	0.9607
Protein/nucleic acid deglycase DJ-1	PARK7_HUMAN	Q99497	1.18	0.0506	0.2898	1.06	0.5076	0.9982	0.92	0.4288	0.9960	0.91	0.2455	0.9939	0.99	0.9275	0.9951	1.13	0.2381	0.9856	0.86	0.1056	0.6824	1.11	0.2706	0.5288	1.00	0.9990	0.9990	1.04	0.6607	0.9881
Prothrombin	THRB_HUMAN	P00734	0.84	0.1738	0.4492	1.15	0.2834	0.9982	1.05	0.7623	0.9960	0.88	0.3225	0.9939	0.83	0.1694	0.9951	0.90	0.5302	0.9856	1.05	0.7134	0.9154	0.67	0.0041	0.1244	1.08	0.5865	0.9889	0.85	0.2106	0.9607
Prothymosin alpha	PTMA_HUMAN	P06454	0.87	0.3198	0.5886	0.93	0.6019	0.9982	0.94	0.6812	0.9960	0.78	0.0791	0.9874	0.77	0.0658	0.9951	1.16	0.3804	0.9856	0.81	0.1522	0.6871	1.33	0.0703	0.2713	0.87	0.3387	0.9889	0.82	0.1672	0.9607
Pterin-4-alpha-carbinolamine dehydratase	PHS_HUMAN	P61457	1.32	0.0138	0.2710	0.99	0.9389	0.9982	1.09	0.5004	0.9960	1.06	0.6165	0.9939	1.12	0.3457	0.9951	1.03	0.8216	0.9959	0.87	0.2679	0.7187	1.28	0.0522	0.2413	0.88	0.3047	0.9889	1.08	0.5220	0.9846
Pterin-4-alpha-carbinolamine dehydratase 2	PHS2_HUMAN	Q9H0N5	1.11	0.1953	0.4726	1.03	0.7451	0.9982	0.96	0.6628	0.9960	0.97	0.7317	0.9939	0.92	0.3300	0.9951	1.07	0.5013	0.9856	0.84	0.0340	0.6824	1.28	0.0041	0.1244	1.05	0.5169	0.9889	1.00	0.9973	0.9999
Puromycin-sensitive aminopeptidase	PSA_HUMAN	P55786	1.18	0.0362	0.2873	1.01	0.9294	0.9982	0.96	0.6295	0.9960	0.90	0.2058	0.9939	1.01	0.9239	0.9951	1.15	0.1774	0.9856	0.92	0.3581	0.7644	1.24	0.0181	0.1645	0.98	0.7891	0.9889	1.04	0.6304	0.9881
Putative deoxyribonuclease TATDN1	TATD1_HUMAN	Q6P1N9	1.27	0.0026	0.2005	1.03	0.7066	0.9982	0.94	0.4758	0.9960	1.01	0.9434	0.9939	1.10	0.2449	0.9951	1.11	0.2891	0.9856	0.93	0.3908	0.7841	1.11	0.2407	0.5050	1.05	0.5845	0.9889	1.08	0.3455	0.9607
Putative transferase CAF17, mitochondrial	CAF17_HUMAN	Q5T440	1.12	0.0413	0.2873	1.03	0.5674	0.9982	0.91	0.1646	0.9960	1.02	0.7767	0.9939	1.02	0.7842	0.9951	1.09	0.2129	0.9856	0.96	0.5033	0.8476	1.07	0.3156	0.5590	1.03	0.6612	0.9889	1.06	0.3220	0.9607
Pyridoxal kinase	PDXK_HUMAN	O00764	1.08	0.3623	0.6254	1.02	0.8295	0.9982	0.94	0.5506	0.9960	0.90	0.2146	0.9939	1.05	0.6115	0.9951	1.13	0.2413	0.9856	0.97	0.7595	0.9282	1.10	0.3087	0.5505	0.87	0.1026	0.9889	0.98	0.8510	0.9881
Pyridoxal phosphate homeostasis protein	PLPHP_HUMAN	O94903	1.11	0.0902	0.3428	1.09	0.1933	0.9982	0.92	0.2478	0.9960	0.97	0.6707	0.9939	1.00	0.9822	0.9964	1.09	0.2467	0.9856	1.01	0.9203	0.9848	0.93	0.3073	0.5505	1.05	0.4875	0.9889	1.00	0.9897	0.9992
Pyruvate dehydrogenase E1 component subunit alpha, somatic form, mit	ODPA_HUMAN	P08559	1.15	0.0217	0.2873	1.08	0.2164	0.9982	1.01	0.9021	0.9960	1.08	0.2224	0.9939	1.09	0.1975	0.9951	0.95	0.4980	0.9856	0.97	0.6057	0.8689	1.02	0.7918	0.8865	1.08	0.2260	0.9889	1.12	0.0716	0.9607
Pyruvate dehydrogenase E1 component subunit beta, mitochondrial	ODPB_HUMAN	P11177	1.08	0.2255	0.4987	1.04	0.5125	0.9982	1.00	0.9911	0.9960	1.08	0.2462	0.9939	1.04	0.5815	0.9951	0.94	0.4759	0.9856	0.93	0.2997	0.7338	1.09	0.2390	0.5042	1.08	0.2546	0.9889	1.09	0.1792	0.9607
Pyruvate dehydrogenase phosphatase regulatory subunit, mitochondrial	PDPR_HUMAN	Q8NCN5	1.16	0.0251	0.2873	1.05	0.4204	0.9982	0.91	0.1984	0.9960	0.99	0.8387	0.9939	1.02	0.7600	0.9951	1.16	0.0674	0.9641	0.93	0.2796	0.7212	1.13	0.0986	0.3193	1.05	0.4359	0.9889	1.00	0.9451	0.9938
Pyruvate dehydrogenase protein X component, mitochondrial	ODPX_HUMAN	O00330	1.03	0.6297	0.8170	0.98	0.7368	0.9982	0.99	0.8913	0.9960	1.00	0.9499	0.9939	1.02	0.7993	0.9951	0.93	0.3201	0.9856	0.94	0.3316	0.7438	1.03	0.6938	0.8265	1.09	0.1570	0.9889	1.05	0.3930	0.9607
Pyruvate kinase PKM	KPYM_HUMAN	P14618	1.14	0.1094	0.3710	1.00	0.9652	0.9982	0.90	0.2354	0.9960	0.92	0.2878	0.9939	0.98	0.7696	0.9951	1.20	0.0619	0.9641	0.95	0.5104	0.8517	1.01	0.9285	0.9587	0.99	0.8713	0.9889	1.00	0.9545	0.9938
Quinone oxidoreductase	QOR_HUMAN	Q08257	1.08	0.4117	0.6643	1.20	0.0497	0.9982	0.92	0.4638	0.9960	0.99	0.9009	0.9939	0.98	0.8460	0.9951	1.09	0.4834	0.9856	0.99	0.8887	0.9753	0.85	0.1338	0.3740	1.10	0.3441	0.9889	1.04	0.6419	0.9881
Rab GDP dissociation inhibitor alpha	GDIA_HUMAN	P31150	1.04	0.5433	0.7505	0.97	0.6310	0.9982	1.05	0.4408	0.9960	0.95	0.4201	0.9939	0.94	0.3553	0.9951	0.99	0.9231	0.9959	0.93	0.2300	0.7187	1.08	0.2656	0.5275	0.97	0.6699	0.9889	0.96	0.5221	0.9846
Rab GDP dissociation inhibitor beta	GDIB_HUMAN	P50395	1.04	0.5009	0.7246	1.00	0.9558	0.9982	1.02	0.7793	0.9960	1.02	0.7410	0.9939	1.03	0.5938	0.9951	0.97	0.5939	0.9856	1.03	0.5586	0.8601	0.90	0.0915	0.3057	0.94	0.2258	0.9889	0.99	0.9195	0.9938
Rab GTPase-binding effector protein 1	RABE1_HUMAN	Q15276	0.92	0.2658	0.5347	0.90	0.1499	0.9982	0.97	0.7281	0.9960	0.99	0.8663	0.9939	1.00	0.9934	0.9979	0.97	0.7479	0.9857	0.95	0.5126	0.8518	1.10	0.2419	0.5055	0.94	0.4125	0.9889	1.01	0.8478	0.9881
Radixin	RADI_HUMAN	P35241	0.95	0.4295	0.6776	0.93	0.3067	0.9982	1.06	0.5004	0.9960	1.01	0.8697	0.9939	0.93	0.2989	0.9951	0.98	0.8401	0.9959	1.05	0.5228	0.8558	1.02	0.8112	0.8963	0.99	0.8677	0.9889	0.93	0.3196	0.9607
Ragulator complex protein LAMTOR1	LTOR1_HUMAN	Q6IAA8	1.02	0.7547	0.8848	1.08	0.1635	0.9982	1.00	0.9459	0.9960	1.14	0.0165	0.9874	1.07	0.2283	0.9951	0.96	0.5494	0.9856	1.05	0.3762	0.7727	0.92	0.1742	0.4258	0.97	0.5598	0.9889	0.97	0.6395	0.9881
Ragulator complex protein LAMTOR3	LTOR3_HUMAN	Q9UHA4	1.05	0.4785	0.7150	0.95	0.4816	0.9982	1.05	0.5154	0.9960	1.12	0.0978	0.9874	1.17	0.0329	0.9951	0.95	0.5282	0.9856	1.14	0.0849	0.6824	0.90	0.1982	0.4593	0.92	0.2236	0.9889	1.04	0.5691	0.9881
Ragulator complex protein LAMTOR5	LTOR5_HUMAN	O43504	0.97	0.6687	0.8389	0.90	0.0897	0.9982	1.10	0.1768	0.9960	0.99	0.8898	0.9939	1.04	0.5805	0.9951	0.91	0.2010	0.9856	1.12	0.0914	0.6824	0.97	0.6954	0.8265	0.96	0.4997	0.9889	0.95	0.4226	0.9607
Ras GTPase-activating protein-binding protein 1	G3BP1_HUMAN	Q13283	1.08	0.1726	0.4478	1.06	0.3230	0.9982	0.95	0.4584	0.9960	1.02	0.7276	0.9939	1.01	0.8296	0.9951	1.05	0.5097	0.9856	0.92	0.1809	0.7142	1.02	0.7133	0.8360	1.01	0.8860	0.9889	1.05	0.3956	0.9607
Ras GTPase-activating-like protein IQGAP1	IQGA1_HUMAN	P46940	0.88	0.1175	0.3810	1.00	0.9631	0.9982	0.94	0.5252	0.9960	1.08	0.3299	0.9939	0.97	0.6943	0.9951	1.08	0.4347	0.9856	1.09	0.3201	0.7400	0.97	0.7121	0.8360	0.93	0.3833	0.9889	0.94	0.4595	0.9607
Ras suppressor protein 1	RSU1_HUMAN	Q15404	1.02	0.6773	0.8429	1.03	0.5106	0.9982	0.98	0.7591	0.9960	0.96	0.3796	0.9939	0.97	0.5019	0.9951	1.00	0.9905	0.9992	0.94	0.2062	0.7149	1.02	0.6872	0.8249	0.92	0.1080	0.9889	0.96	0.4533	0.9607
Ras-related C3 botulinum toxin substrate 1	RAC1_HUMAN	P63000	0.88	0.1469	0.4117	0.93	0.4220	0.9982	0.98	0.8266	0.9960	1.06	0.4879	0.9939	1.08	0.4061	0.9951	1.02	0.8646	0.9959	1.26	0.0101	0.6779	0.91	0.3335	0.5705	1.07	0.4230	0.9889	0.98	0.8462	0.9881
Ras-related protein R-Ras	RRAS_HUMAN	P10301	0.82	0.0068	0.2487	1.01	0.9429	0.9982	1.12	0.1844	0.9960	1.02	0.7905	0.9939	0.93	0.3370	0.9951	0.84	0.0538	0.9631	1.08	0.3282	0.7411	0.86	0.0700	0.2711	0.89	0.1111	0.9889	0.91	0.1804	0.9607
Ras-related protein Rab-10	RAB10_HUMAN	P61026	0.97	0.6476	0.8293	1.02	0.7910	0.9982	0.97	0.6982	0.9960	1.08	0.1786	0.9939	1.06	0.3449	0.9951	0.99	0.8527	0.9959	1.01	0.8636	0.9639	0.92	0.2230	0.4912	0.96	0.4766	0.9889	1.06	0.3357	0.9607
Ras-related protein Rab-11B	RB11B_HUMAN	Q15907	1.04	0.4327	0.6798	1.06	0.2608	0.9982	0.99	0.8956	0.9960	1.16	0.0030	0.8235	1.12	0.0335	0.9951	0.97	0.6003	0.9856	1.16	0.0047	0.6779	0.88	0.0225	0.1743	0.96	0.4635	0.9889	1.04	0.4586	0.9607
Ras-related protein Rab-14	RAB14_HUMAN	P61106	1.02	0.7346	0.8772	1.10	0.1129	0.9982	1.07	0.3218	0.9960	1.06	0.3699	0.9939	1.01	0.8772	0.9951	0.86	0.0474	0.9535	0.97	0.6828	0.9001	0.92	0.2037	0.4651	0.96	0.5274	0.9889	1.01	0.8969	0.9938
Ras-related protein Rab-18	RAB18_HUMAN	Q9NP72	0.97	0.5489	0.7524	0.98	0.7215	0.9982	0.99	0.9252	0.9960	1.02	0.7839	0.9939	1.02	0.7496	0.9951	0.95	0.4519	0.9856	0.94	0.3251	0.7400	0.99	0.8057	0.8941	0.98	0.7767	0.9889	1.03	0.5738	0.9881
Ras-related protein Rab-1A	RAB1A_HUMAN	P62820	0.98	0.7215	0.8735	1.02	0.7527	0.9982	1.03	0.5793	0.9960	1.03	0.5786	0.9939	1.01	0.8339	0.9951	0.94	0.3211	0.9856	1.05	0.3514	0.7593	0.95	0.3391	0.5744	0.96	0.4407	0.9889	1.00	0.9195	0.9938
Ras-related protein Rab-1B	RAB1B_HUMAN	Q9H0U4	1.01	0.8674	0.9356	1.00	0.9619	0.9982	0.87	0.0734	0.9960	1.09	0.1902	0.9939	1.03	0.7020	0.9951	1.09	0.2926	0.9856	0.92	0.2668	0.7187	1.02	0.7481	0.8622	1.04	0.5412	0.9889	1.10	0.1516	0.9607
Ras-related protein Rab-21	RAB21_HUMAN	Q9UL25	1.01	0.9009	0.9479	1.04	0.5245	0.9982	1.03	0.6918	0.9960	1.09	0.2004	0.9939	1.02	0.7321	0.9951	0.91	0.2563	0.9856	0.92	0.2559	0.7187	1.09	0.2742	0.5288	1.00	0.9581	0.9961	1.01	0.8847	0.9938
Ras-related protein Rab-2A	RAB2A_HUMAN	P61019	1.02	0.7035	0.8605	1.05	0.3140	0.9982	0.99	0.8438	0.9960	1.00	0.9329	0.9939	1.02	0.6986	0.9951	0.97	0.6286	0.9856	0.98	0.7533	0.9282	0.99	0.8916	0.9399	1.03	0.6341	0.9889	1.03	0.5456	0.9881
Ras-related protein Rab-5C	RAB5C_HUMAN	P51148	1.07	0.4986	0.7246	1.00	0.9745	0.9982	1.31	0.0152	0.9960	1.10	0.3290	0.9939	1.06	0.5503	0.9951	0.79	0.0458	0.9535	1.12	0.2686	0.7187	0.88	0.2611	0.5247	0.97	0.7536	0.9889	0.99	0.8821	0.9938
Ras-related protein Rab-6A	RAB6A_HUMAN	P20340	1.05	0.3856	0.6435	1.07	0.2852	0.9982	1.06	0.4048	0.9960	1.02	0.6953	0.9939	1.10	0.1306	0.9951	0.90	0.1564	0.9856	0.98	0.7617	0.9282	0.92	0.2353	0.4981	0.91	0.1185	0.9889	1.06	0.3045	0.9607
Ras-related protein Rab-7a	RAB7A_HUMAN	P51149	1.01	0.7995	0.9037	1.01	0.8128	0.9982	1.06	0.3052	0.9960	1.00	0.9293	0.9939	1.02	0.6652	0.9951	0.91	0.1438	0.9856	1.01	0.8109	0.9478	0.97	0.6133	0.7814	0.96	0.3890	0.9889	1.02	0.6650	0.9881
Ras-related protein Ral-A	RALA_HUMAN	P11233	0.87	0.0495	0.2898	0.99	0.8725	0.9982	0.95	0.5725	0.9960	0.94	0.4209	0.9939	0.87	0.0772	0.9951	0.99	0.9544	0.9967	1.08	0.3239	0.7400	0.94	0.4458	0.6635	0.92	0.2822	0.9889	0.89	0.1023	0.9607
Ras-related protein Ral-B	RALB_HUMAN	P11234	0.83	0.0106	0.2530	0.94	0.3995	0.9982	1.03	0.7312	0.9960	0.99	0.8786	0.9939	0.92	0.2728	0.9951	0.95	0.6094	0.9856	1.02	0.8003	0.9447	0.96	0.6541	0.8025	1.03	0.6986	0.9889	0.96	0.5517	0.9881
Ras-related protein Rap-1A	RAP1A_HUMAN	P62834	0.77	0.0010	0.1794	0.92	0.3439	0.9982	1.15	0.1602	0.9960	0.97	0.6902	0.9939	0.84	0.0421	0.9951	0.85	0.1159	0.9856	1.03	0.7266	0.9205	1.09	0.3661	0.5972	0.95	0.5149	0.9889	0.85	0.0441	0.9607
Ras-related protein Rap-1b	RAP1B_HUMAN	P61224	0.90	0.0972	0.3563	1.04	0.5838	0.9982	1.07	0.3412	0.9960	1.03	0.6046	0.9939	1.00	0.9800	0.9964	0.91	0.2443	0.9856	1.17	0.0194	0.6779	0.87	0.0482	0.2317	0.99	0.8756	0.9889	1.00	0.9910	0.9992
Receptor expression-enhancing protein 5	REEP5_HUMAN	Q00765	0.87	0.0200	0.2873	0.96	0.4553	0.9982	1.01	0.9322	0.9960	1.10	0.1155	0.9874	0.98	0.6873	0.9951	0.93	0.3505	0.9856	1.04	0.5640	0.8601	0.99	0.9406	0.9654	0.97	0.6494	0.9889	0.98	0.7643	0.9881
Receptor of activated protein C kinase 1	RACK1_HUMAN	P63244	1.07	0.3367	0.6019	1.04	0.5736	0.9982	1.07	0.4029	0.9960	0.90	0.1522	0.9874	0.94	0.4423	0.9951	0.91	0.2978	0.9856	0.87	0.0844	0.6824	1.05	0.5301	0.7227	1.03	0.6957	0.9889	0.93	0.3213	0.9607
Regulator of microtubule dynamics protein 1	RMD1_HUMAN	Q96DB5	1.20	0.0168	0.2873	1.03	0.7322	0.9982	0.94	0.4727	0.9960	0.99	0.9091	0.9939	1.06	0.4628	0.9951	1.03	0.7457	0.9857	1.02	0.8376	0.9533	1.14	0.1168	0.3471	1.09	0.2561	0.9889	1.11	0.1590	0.9607
RelA-associated inhibitor	IASPP_HUMAN	Q8WUF5	0.94	0.5043	0.7271	0.95	0.5586	0.9982	0.91	0.3286	0.9960	0.98	0.7972	0.9939	0.99	0.9217	0.9951	1.16	0.1570	0.9856	0.89	0.1794	0.7130	1.12	0.2440	0.5086	0.97	0.7029	0.9889	0.98	0.8538	0.9881
Reticulocalbin-1	RCN1_HUMAN	Q15293	0.90	0.2489	0.5210	1.03	0.7559	0.9982	0.90	0.3160	0.9960	1.04	0.6476	0.9939	0.93	0.4704	0.9951	1.09	0.4468	0.9856	1.14	0.1774	0.7117	0.88	0.2242	0.4912	0.93	0.4308	0.9889	0.92	0.3503	0.9607
Reticulocalbin-2	RCN2_HUMAN	Q14257	0.96	0.5414	0.7503	0.91	0.1979	0.9982	1.09	0.2790	0.9960	0.99	0.8933	0.9939	0.98	0.8252	0.9951	0.96	0.6581	0.9856	0.95	0.4497	0.8181	1.05	0.5098	0.7055	0.94	0.4284	0.9889	1.00	0.9757	0.9992
Reticulon-2	RTN2_HUMAN	O75298	0.89	0.2961	0.5677	0.91	0.3940	0.9982	0.98	0.8892	0.9960	1.09	0.4407	0.9939	1.00	0.9799	0.9964	1.01	0.9133	0.9959	1.16	0.1914	0.7149	0.90	0.4121	0.6378	0.97	0.7953	0.9889	1.06	0.5884	0.9881
Reticulon-3	RTN3_HUMAN	O95197	0.88	0.1358	0.3997	0.95	0.5433	0.9982	1.02	0.8190	0.9960	1.07	0.4469	0.9939	0.93	0.4484	0.9951	0.89	0.2846	0.9856	0.99	0.9422	0.9903	0.97	0.7886	0.8865	0.97	0.7519	0.9889	0.98	0.8040	0.9881
Reticulon-4	RTN4_HUMAN	Q9NQC3	0.99	0.9222	0.9555	1.07	0.5939	0.9982	0.96	0.8057	0.9960	1.06	0.6489	0.9939	0.94	0.6356	0.9951	1.03	0.8345	0.9959	1.13	0.3698	0.7673	0.79	0.1045	0.3288	1.07	0.6356	0.9889	1.08	0.5570	0.9881
Reticulon-4-interacting protein 1, mitochondrial	RT4I1_HUMAN	Q8WWV3	1.16	0.0488	0.2898	1.00	0.9917	0.9985	0.94	0.4598	0.9960	1.11	0.1421	0.9874	1.10	0.2085	0.9951	1.04	0.6897	0.9856	0.97	0.6624	0.8929	1.12	0.1752	0.4263	1.08	0.2951	0.9889	1.21	0.0093	0.9304
Retinal dehydrogenase 1	AL1A1_HUMAN	P00352	0.88	0.1259	0.3929	0.98	0.7770	0.9982	0.98	0.8380	0.9960	0.97	0.7238	0.9939	0.92	0.3470	0.9951	1.09	0.3702	0.9856	1.14	0.1197	0.6868	1.02	0.7987	0.8908	0.94	0.4665	0.9889	0.94	0.4351	0.9607
Retinol dehydrogenase 13	RDH13_HUMAN	Q8NBN7	1.19	0.0264	0.2873	1.01	0.8744	0.9982	0.92	0.3885	0.9960	0.93	0.3555	0.9939	1.09	0.2737	0.9951	1.03	0.7904	0.9903	0.87	0.1028	0.6824	1.09	0.3286	0.5681	1.13	0.1402	0.9889	1.24	0.0051	0.9304
Retinol-binding protein 4	RET4_HUMAN	P02753	0.91	0.3071	0.5769	1.23	0.0297	0.9982	0.99	0.9406	0.9960	1.06	0.5442	0.9939	0.94	0.5474	0.9951	1.11	0.4022	0.9856	1.05	0.6309	0.8874	0.87	0.1981	0.4593	1.03	0.7324	0.9889	0.93	0.4222	0.9607
Rho GDP-dissociation inhibitor 1	GDIR1_HUMAN	P52565	0.92	0.2161	0.4877	0.97	0.6491	0.9982	0.99	0.9059	0.9960	0.95	0.4711	0.9939	0.87	0.0318	0.9951	1.09	0.2823	0.9856	0.99	0.9169	0.9848	1.09	0.2502	0.5149	0.95	0.4162	0.9889	0.86	0.0143	0.9304

Rho GDP-dissociation inhibitor 2	GDIR2_HUMAN	P52566	1.03	0.6618	0.8357	0.94	0.3817	0.9982	0.91	0.3147	0.9960	0.94	0.4198	0.9939	1.02	0.8227	0.9951	1.12	0.2141	0.9856	0.97	0.7107	0.9146	1.10	0.2589	0.5237	0.87	0.0648	0.9889	0.96	0.6004	0.9881
Rho GTPase-activating protein 1	RHG01_HUMAN	Q07960	1.07	0.3245	0.5917	1.01	0.9017	0.9982	1.07	0.3722	0.9960	1.05	0.5023	0.9939	1.03	0.6646	0.9951	0.92	0.3550	0.9856	1.03	0.6433	0.8896	1.06	0.4304	0.6512	1.11	0.1494	0.9889	1.06	0.4279	0.9607
Rho-related GTP-binding protein RhoC	RHOC_HUMAN	P08134	0.89	0.0879	0.3401	0.96	0.5790	0.9982	1.03	0.6988	0.9960	1.07	0.3190	0.9939	0.99	0.8734	0.9951	1.00	0.9728	0.9967	1.10	0.2006	0.7149	0.96	0.5562	0.7396	1.01	0.8898	0.9889	0.99	0.9190	0.9938
Ribonuclease inhibitor	RINI_HUMAN	P13489	1.11	0.1185	0.3810	1.04	0.5921	0.9982	1.02	0.7860	0.9960	1.03	0.6103	0.9939	1.01	0.9063	0.9951	1.02	0.8485	0.9959	0.91	0.1588	0.6871	0.94	0.4312	0.6512	0.99	0.9151	0.9889	1.04	0.5690	0.9881
Ribose-5-phosphate isomerase	RPIA_HUMAN	P49247	1.11	0.1083	0.3689	0.98	0.7156	0.9982	0.99	0.9240	0.9960	0.94	0.3347	0.9939	1.03	0.6360	0.9951	0.99	0.9044	0.9959	0.95	0.5214	0.8558	1.09	0.2620	0.5247	0.96	0.5186	0.9889	1.06	0.3757	0.9607
Ribose-phosphate pyrophosphokinase 1	PRPS1_HUMAN	P60891	0.98	0.8389	0.9204	0.89	0.1660	0.9982	1.03	0.7618	0.9960	0.89	0.1403	0.9874	0.93	0.4045	0.9951	0.90	0.2863	0.9856	1.00	0.9569	0.9923	1.13	0.1903	0.4483	1.04	0.6141	0.9889	1.00	0.9624	0.9956
Ribosome-binding protein 1	RRBP1_HUMAN	Q9P2E9	0.87	0.0947	0.3539	0.90	0.2048	0.9982	1.04	0.7136	0.9960	1.06	0.4541	0.9939	0.96	0.6495	0.9951	1.03	0.7976	0.9943	1.14	0.1264	0.6868	0.99	0.9446	0.9689	0.93	0.3738	0.9889	0.93	0.3638	0.9607
Ribosome-recycling factor, mitochondrial	RRFM_HUMAN	Q96E11	1.06	0.3146	0.5854	0.99	0.9301	0.9982	0.88	0.0525	0.9960	1.04	0.5100	0.9939	1.02	0.7945	0.9951	1.12	0.0979	0.9856	0.98	0.7369	0.9218	1.05	0.4489	0.6643	1.07	0.2396	0.9889	1.03	0.6419	0.9881
RNA transcription, translation and transport factor protein	RTRAF_HUMAN	Q9Y224	0.90	0.0401	0.2873	0.95	0.3728	0.9982	1.04	0.5403	0.9960	0.98	0.7445	0.9939	0.93	0.1959	0.9951	0.97	0.6574	0.9856	0.95	0.3965	0.7880	1.07	0.2734	0.5288	0.99	0.7781	0.9889	0.96	0.4162	0.9607
RNA-binding protein 8A	RBM8A_HUMAN	Q9Y5S9	1.14	0.0487	0.2898	1.08	0.2431	0.9982	0.98	0.8053	0.9960	0.93	0.2868	0.9939	1.06	0.4314	0.9951	1.02	0.8036	0.9959	0.99	0.8798	0.9722	0.86	0.0364	0.2067	0.85	0.0187	0.9889	1.01	0.8990	0.9938
RNA-binding protein Raly	RALY_HUMAN	Q9UKM9	1.06	0.3884	0.6461	1.03	0.6442	0.9982	0.97	0.6658	0.9960	0.96	0.5193	0.9939	1.05	0.4475	0.9951	1.04	0.6067	0.9856	1.04	0.5899	0.8646	1.05	0.4792	0.6833	1.07	0.3042	0.9889	0.99	0.8624	0.9881
RNA-binding protein with multiple splicing	RBPMS_HUMAN	Q93062	1.30	0.0410	0.2873	1.31	0.0381	0.9982	0.91	0.5291	0.9960	1.11	0.4409	0.9939	1.33	0.0335	0.9951	1.16	0.3567	0.9856	0.77	0.0575	0.6824	0.80	0.1233	0.3567	0.92	0.5541	0.9889	1.38	0.0108	0.9304
RNA-splicing ligase RtcB homolog	RTCB_HUMAN	Q9Y3I0	0.91	0.2284	0.5015	0.96	0.5795	0.9982	0.98	0.7822	0.9960	1.07	0.3273	0.9939	1.02	0.8329	0.9951	1.02	0.8257	0.9959	1.14	0.1016	0.6824	0.85	0.0412	0.2167	1.04	0.6077	0.9889	1.00	0.9521	0.9938
rRNA methyltransferase 3, mitochondrial	MRM3_HUMAN	Q9HC36	1.02	0.7813	0.8981	1.02	0.8062	0.9982	0.89	0.0953	0.9960	1.08	0.1913	0.9939	1.07	0.3202	0.9951	1.05	0.5360	0.9856	1.07	0.3250	0.7400	0.93	0.2798	0.5337	1.01	0.8179	0.9889	1.05	0.4705	0.9607
RuvB-like 1	RUVB1_HUMAN	Q9Y265	1.04	0.4078	0.6627	1.04	0.4069	0.9982	1.07	0.1875	0.9960	1.01	0.8777	0.9939	1.01	0.7646	0.9951	0.94	0.3236	0.9856	1.04	0.3983	0.7880	0.96	0.4846	0.6878	1.03	0.6106	0.9889	1.00	0.9812	0.9992
RuvB-like 2	RUVB2_HUMAN	Q9Y230	1.06	0.2548	0.5233	1.04	0.4590	0.9982	1.04	0.5165	0.9960	1.02	0.7541	0.9939	1.09	0.1063	0.9951	0.95	0.4646	0.9856	0.95	0.4023	0.7880	0.88	0.0251	0.1799	1.00	0.9592	0.9961	1.16	0.0049	0.9304
Ryanodine receptor 2	RYR2_HUMAN	Q92736	1.18	0.1189	0.3810	0.99	0.9170	0.9982	0.92	0.5244	0.9960	1.05	0.6260	0.9939	1.09	0.4276	0.9951	0.93	0.5894	0.9856	1.07	0.5672	0.8601	1.07	0.5900	0.7646	1.11	0.3427	0.9889	1.20	0.0897	0.9607
S-adenosylmethionine synthase isoform type-2	METK2_HUMAN	P31153	1.00	0.9860	0.9915	0.88	0.0795	0.9982	0.99	0.9021	0.9960	0.98	0.7687	0.9939	0.96	0.5922	0.9951	1.08	0.3640	0.9856	0.84	0.0165	0.6779	1.29	0.0012	0.0763	1.01	0.8631	0.9889	1.02	0.8322	0.9881
S-formylglutathione hydrolase	ESTD_HUMAN	P10768	1.12	0.1733	0.4489	0.98	0.7671	0.9982	0.98	0.8702	0.9960	0.94	0.4571	0.9939	1.03	0.6980	0.9951	1.12	0.2550	0.9856	0.90	0.2121	0.7171	1.24	0.0146	0.1589	0.98	0.7920	0.9889	1.02	0.8000	0.9881
S-methyl-5'-thioadenosine phosphorylase	MTAP_HUMAN	Q13126	1.20	0.0440	0.2873	0.94	0.5015	0.9982	1.07	0.5311	0.9960	0.93	0.4294	0.9939	1.03	0.7793	0.9951	1.06	0.6016	0.9856	0.86	0.0999	0.6824	1.22	0.0475	0.2309	0.89	0.2161	0.9889	1.01	0.9216	0.9938
S-phase kinase-associated protein 1	SKP1_HUMAN	P63208	0.97	0.5119	0.7292	0.99	0.7983	0.9982	1.06	0.3347	0.9960	0.96	0.4251	0.9939	0.97	0.5407	0.9951	1.00	0.9873	0.9992	0.88	0.0157	0.6779	1.07	0.2516	0.5154	0.96	0.4465	0.9889	0.97	0.5079	0.9846
Saccharopine dehydrogenase-like oxidoreductase	SCPDL_HUMAN	Q8NBX0	0.91	0.1305	0.3958	1.00	0.9640	0.9982	1.03	0.6928	0.9960	1.05	0.4636	0.9939	1.02	0.7365	0.9951	0.94	0.4388	0.9856	1.01	0.9155	0.9848	1.02	0.8113	0.8963	1.00	0.9805	0.9976	1.04	0.5504	0.9881
Sarcalumenin	SRCA_HUMAN	Q86TD4	1.01	0.9058	0.9497	0.91	0.2582	0.9982	1.03	0.7551	0.9960	1.07	0.3808	0.9939	1.00	0.9746	0.9951	0.94	0.5600	0.9856	0.91	0.2850	0.7212	1.22	0.0262	0.1832	0.97	0.7542	0.9889	0.97	0.6810	0.9881
Sarcolemmal membrane-associated protein	SLMAP_HUMAN	Q14BN4	0.84	0.0065	0.2487	0.93	0.2789	0.9982	1.03	0.6632	0.9960	1.07	0.3064	0.9939	0.94	0.3789	0.9951	0.96	0.5980	0.9856	0.96	0.5990	0.8646	1.02	0.7436	0.8595	0.99	0.8764	0.9889	0.96	0.5028	0.9818
Sarcoplasmic reticulum histidine-rich calcium-binding protein	SRCH_HUMAN	P23327	1.10	0.2057	0.4823	0.93	0.3621	0.9982	1.05	0.5952	0.9960	1.05	0.5456	0.9939	1.03	0.7185	0.9951	0.95	0.5566	0.9856	0.98	0.8137	0.9478	1.15	0.1049	0.3293	1.01	0.8726	0.9889	1.08	0.2924	0.9607
Sarcoplasmic/endoplasmic reticulum calcium ATPase 2	AT2A2_HUMAN	P16615	1.11	0.2449	0.5184	1.06	0.4919	0.9982	1.07	0.5093	0.9960	1.11	0.2372	0.9939	1.05	0.5568	0.9951	0.90	0.3349	0.9856	0.93	0.4009	0.7880	1.03	0.7470	0.8615	0.97	0.7538	0.9889	1.07	0.4499	0.9607
Scaffold attachment factor B1	SAFB1_HUMAN	Q15424	0.84	0.0076	0.2487	0.95	0.4758	0.9982	0.91	0.2320	0.9960	0.84	0.0106	0.9874	0.89	0.0942	0.9951	1.17	0.0578	0.9641	0.96	0.6086	0.8709	1.03	0.7108	0.8360	0.98	0.7272	0.9889	0.90	0.1346	0.9607
Sec1 family domain-containing protein 1	SCFD1_HUMAN	Q8WVM8	1.09	0.0384	0.2873	1.06	0.1654	0.9982	0.96	0.3985	0.9960	1.05	0.2693	0.9939	1.08	0.0916	0.9951	1.03	0.5913	0.9856	1.03	0.5296	0.8563	0.99	0.8691	0.9275	1.01	0.8297	0.9889	1.03	0.4535	0.9607
Secernin-1	SCRN1_HUMAN	Q12765	1.09	0.2705	0.5392	1.01	0.9245	0.9982	0.96	0.6271	0.9960	1.00	0.9646	0.9947	1.11	0.2093	0.9951	1.04	0.6711	0.9856	0.98	0.8204	0.9481	0.97	0.7580	0.8671	0.93	0.3675	0.9889	0.99	0.8697	0.9925
Sepiapterin reductase	SPRE_HUMAN	P35270	1.17	0.0078	0.2487	1.02	0.7718	0.9982	0.87	0.0462	0.9960	1.03	0.5903	0.9939	1.10	0.1042	0.9951	1.09	0.2320	0.9856	1.05	0.4514	0.8191	0.96	0.5891	0.7646	0.99	0.8283	0.9889	1.06	0.3327	0.9607
Septin-11	SEP11_HUMAN	Q9NVA2	0.91	0.2365	0.5100	1.02	0.7656	0.9982	1.04	0.6847	0.9960	1.07	0.3688	0.9939	0.90	0.1966	0.9951	0.96	0.7023	0.9857	1.02	0.8257	0.9492	0.82	0.0286	0.1909	0.98	0.8121	0.9889	0.91	0.2184	0.9607
Septin-2	SEPT2_HUMAN	Q15019	0.89	0.0755	0.3266	1.06	0.3730	0.9982	0.99	0.9096	0.9960	1.01	0.8700	0.9939	0.89	0.1034	0.9951	1.02	0.8322	0.9959	1.05	0.5104	0.8517	0.85	0.0299	0.1912	1.01	0.8479	0.9889	0.91	0.1752	0.9607
Septin-7	SEPT7_HUMAN	Q16181	0.88	0.0679	0.3121	1.06	0.3834	0.9982	1.00	0.9722	0.9960	0.97	0.7186	0.9939	0.86	0.0350	0.9951	0.99	0.8874	0.9959	1.03	0.6953	0.9067	0.83	0.0142	0.1589	1.03	0.6764	0.9889	0.90	0.1257	0.9607
Septin-9	SEPT9_HUMAN	Q9UHD8	0.94	0.3463	0.6090	1.09	0.1821	0.9982	1.14	0.0795	0.9960	0.91	0.1493	0.9874	0.89	0.0892	0.9951	0.87	0.0997	0.9856	0.96	0.5754	0.8630	0.88	0.0802	0.2899	1.01	0.8407	0.9889	0.88	0.0579	0.9607
Serine beta-lactamase-like protein LACTB, mitochondrial	LACTB_HUMAN	P83111	1.06	0.4707	0.7095	1.05	0.4777	0.9982	1.03	0.7695	0.9960	1.06	0.4161	0.9939	1.16	0.0465	0.9951	0.94	0.5352	0.9856	1.05	0.5028	0.8476	0.91	0.2735	0.5288	1.06	0.4136	0.9889	1.09	0.2558	0.9607
Serine hydroxymethyltransferase, mitochondrial	GLYM_HUMAN	P34897	0.99	0.8748	0.9374	1.01	0.8494	0.9982	1.06	0.4128	0.9960	0.96	0.5595	0.9939	0.94	0.3558	0.9951	0.93	0.3611	0.9856	0.97	0.6664	0.8929	1.06	0.4312	0.6512	0.97	0.6574	0.9889	0.85	0.0101	0.9304
Serine protease HTRA2, mitochondrial	HTRA2_HUMAN	O43464	1.24	0.1286	0.3940	1.03	0.8215	0.9982	1.11	0.5163	0.9960	0.95	0.7300	0.9939	1.05	0.7591	0.9951	0.86	0.3952	0.9856	1.21	0.2044	0.7149	1.03	0.8350	0.9130	0.95	0.7379	0.9889	1.01	0.9698	0.9992
Serine--tRNA ligase, mitochondrial	SYSM_HUMAN	Q9NP81	1.17	0.0198	0.2873	1.22	0.0039	0.9982	0.96	0.6097	0.9960	1.11	0.1424	0.9874	1.09	0.2353	0.9951	1.06	0.4724	0.9856	1.02	0.7899	0.9417	1.03	0.7014	0.8317	1.07	0.3241	0.9889	1.07	0.3285	0.9607
Serine-threonine kinase receptor-associated protein	STRAP_HUMAN	Q9Y3F4	1.02	0.7429	0.8802	0.88	0.0132	0.9982	1.07	0.2837	0.9960	0.93	0.1732	0.9907	0.96	0.4643	0.9951	0.97	0.6392	0.9856	0.89	0.0316	0.6824	1.13	0.0347	0.2017	0.94	0.2930	0.9889	0.95	0.3163	0.9607
Serine/arginine-rich splicing factor 1	SRSF1_HUMAN	Q07955	1.07	0.3367	0.6019	1.24	0.0019	0.9982	0.97	0.6769	0.9960	1.15	0.0531	0.9874	1.03	0.6865	0.9951	0.99	0.9078	0.9959	1.10	0.2056	0.7149	0.80	0.0051	0.1313	1.05	0.5356	0.9889	1.05	0.5116	0.9846
Serine/threonine-protein kinase PAK 2	PAK2_HUMAN	Q13177	1.03	0.5971	0.7926	1.01	0.7852	0.9982	1.01	0.9126	0.9960	1.02	0.7166	0.9939	1.07	0.2159	0.9951	1.07	0.2792	0.9856	0.99	0.8191	0.9481	1.00	0.9915	0.9952	0.97	0.5547	0.9889	1.05	0.3383	0.9607
Serine/threonine-protein phosphatase 2A 55 kDa regulatory subunit B alp	2ABA_HUMAN	P63151	1.01	0.8300	0.9186	0.98	0.7278	0.9982	1.18	0.0068	0.9960	0.98	0.7017	0.9939	0.97	0.5850	0.9951	0.89	0.0662	0.9641	0.97	0.5332	0.8567	1.06	0.3342	0.5706	0.94	0.2570	0.9889	0.97	0.5187	0.9846
Serine/threonine-protein phosphatase 2A 56 kDa regulatory subunit alph	2A5A_HUMAN	Q15172	0.94	0.4169	0.6671	0.91	0.2060	0.9982	1.01	0.8866	0.9960	1.12	0.1249	0.9874	0.96	0.6048	0.9951	1.07	0.4916	0.9856	1.06	0.5098	0.8517	1.18	0.0571	0.2478	0.82	0.0132	0.9889	0.98	0.7832	0.9881
Serine/threonine-protein phosphatase 2A 65 kDa regulatory subunit A alp	2AAA_HUMAN	P30153	1.10	0.1457	0.4103	0.99	0.9189	0.9982	1.06	0.4899	0.9960	0.99	0.8609	0.9939	0.99	0.8588	0.9951	0.99	0.9263	0.9959	0.88	0.0700	0.6824	1.22	0.0072	0.1331	0.94	0.3691	0.9889	0.97	0.7037	0.9881
Serine/threonine-protein phosphatase 2A catalytic subunit alpha isoform	PP2AA_HUMAN	P67775	1.00	0.9431	0.9668	0.97	0.6477	0.9982	1.06	0.3850	0.9960	0.98	0.7179	0.9939	0.96	0.5305	0.9951	0.95	0.4536	0.9856	0.86	0.0098	0.6779	1.18	0.0074	0.1348	0.93	0.2500	0.9889	0.96	0.4361	0.9607
Serine/threonine-protein phosphatase 2A catalytic subunit beta isoform	PP2AB_HUMAN	P62714	1.02	0.7032	0.8605	0.98	0.6574	0.9982	1.07	0.2780	0.9960	1.00	0.9805	0.9967	0.96	0.4565	0.9951	0.96	0.4882	0.9856	0.88	0.0128	0.6779	1.11	0.0737	0.2794	0.92	0.0935	0.9889	0.96	0.3749	0.9607
Serine/threonine-protein phosphatase 5	PPP5_HUMAN	P53041	0.98	0.7601	0.8882	1.05	0.4509	0.9982	0.97	0.6595	0.9960	1.01	0.8350	0.9939	0.93	0.2546	0.9951	1.07	0.3805	0.9856	0.97	0.6398	0.8892	0.97	0.6949	0.8265	0.99	0.9124	0.9889	0.90	0.1168	0.9607
Serine/threonine-protein phosphatase PP1-alpha catalytic subunit	PP1A_HUMAN	P62136	0.89	0.0341	0.2873	0.96	0.4471	0.9982	1.20	0.0046	0.9960	0.98	0.7483	0.9939	0.90	0.0711	0.9951	0.87	0.0394	0.9411	0.98	0.7962	0.9431	1.15	0.0216	0.1725	0.93	0.2244	0.9889	0.89	0.0295	0.9607
Serine/threonine-protein phosphatase PP1-beta catalytic subunit	PP1B_HUMAN	P62140	1.04	0.5660	0.7665	1.08	0.2624	0.9982	0.92	0.2671	0.9960	0.97	0.6132	0.9939	1.02	0.7676	0.9951	1.11	0.2001	0.9856	1.00	0.9704	0.9937	0.99	0.8654	0.9254	1.02	0.7699	0.9889	1.04	0.5507	0.9881
Serotransferrin	TRFE_HUMAN	P02787	0.87	0.2196	0.4919	0.99	0.9328	0.9982	1.11	0.4031	0.9960	0.99	0.9219	0.9939	1.00	0.9670	0.9951	0.94	0.6678	0.9856	1.12	0.3299	0.7411	0.80	0.0760	0.2841	0.99	0.9335	0.9907	0.95	0.6458	0.9881
Serpin B6	SPB6_HUMAN	P35237	1.09	0.3244	0.5917	1.01	0.8970	0.9982	1.05	0.5807	0.9960	0.94	0.4947	0.9939	0.96	0.6182	0.9951	1.03	0.7899	0.9903	0.82	0.0264	0.6824	1.15	0.1346	0.3748	0.92	0.3490	0.9889	0.93	0.3557	0.9607
Serpin H1	SERPH_HUMAN	P50454	0.83	0.1400	0.4030	0.90	0.3990	0.9982	0.94	0.6785	0.9960	1.05	0.6938	0.9939	0.95	0.6751	0.9951	1.01	0.9431	0.9959	1.27	0.0747	0.6824	0.81	0.1204	0.3537	0.88	0.3238	0.9889	0.91	0.4554	0.9607
Serum albumin	ALBU_HUMAN	P02768	0.89	0.2854	0.5588	1.04	0.6876	0.9982	1.07	0.5683	0.9960	1.02	0.8507	0.9939	1.00	0.9776	0.9964	0.98	0.8869	0.9959	1.08	0.5047	0.8478	0.90	0.3597	0.5943	0.94	0.5627	0.9889	0.89	0.2862	0.9607
Serum amyloid A-1 protein	SAA1_HUMAN	P0DJI8	1.03	0.9401	0.9668	1.48	0.2793	0.9982	1.08	0.8515	0.9960	1.03	0.9302	0.9939	1.12	0.7588	0.9951	0.66	0.3507	0.9856	1.60	0.2214	0.7187	0.34	0.0060	0.1317	0.72	0.3880	0.9889	0.97	0.9315	0.9938
Serum amyloid P-component	SAMP_HUMAN	P02743	0.83	0.1867	0.4611	1.22	0.1583	0.9982	1.19	0.2912	0.9960	0.95	0.7275	0.9939	0.92	0.5681	0.9951	0.84	0.3132	0.9856	1.30	0.0805	0.6824	0.83	0.2328	0.4959	1.16	0.2943	0.9889	0.94	0.6538	0.9881
SH3 domain-binding glutamic acid-rich protein	SH3BG_HUMAN	P55822	0.91	0.2119	0.4843	1.04	0.6303	0.9982	1.11	0.2003	0.9960	1.04	0.5531	0.9939	0.86	0.0472	0.9951	0.83	0.0399	0.9411	1.00	0.9589	0.9924	1.09	0.3039	0.5503	0.95	0.4758	0.9889	0.86	0.0327	0.9607
SH3 domain-binding glutamic acid-rich-like protein 3	SH3L3_HUMAN	Q9H299	0.92	0.5452	0.7505	1.18	0.2192	0.9982	1.00	0.9889	0.9960	1.15	0.2972	0.9939	1.05	0.7344	0.9951	1.00	0.9879	0.9992	1.40	0.0167	0.6779	0.69	0.0111	0.1498	0.81	0.1223	0.9889	0.88	0.3403	0.9607
Short-chain specific acyl-CoA dehydrogenase, mitochondrial	ACADS_HUMAN	P16219	0.97	0.8137	0.9115	0.96	0.7705	0.9982	0.89	0.4438	0.9960	1.04	0.7512	0.9939	1.02	0.8923	0.9951	1.08	0.6378	0.9856	0.94	0.6405	0.8892	1.04	0.8013	0.8919	1.22	0.1474	0.9889	0.95	0.6808	0.9881
Short/branched chain specific acyl-CoA dehydrogenase, mitochondrial	ACDSB_HUMAN	P45954	1.05	0.5367	0.7490	0.92	0.3226	0.9982	1.04	0.6541	0.9960	1.01	0.9379	0.9939	0.95	0.5288	0.9951	0.97	0.7431	0.9857	1.09	0.3467	0.7593	1.10	0.2818	0.5346	1.17	0.0562	0.9889	0.99	0.8549	0.9881
Sialate O-acetylesterase	SIAE_HUMAN	Q9HAT2	1.34	0.0105	0.2530	1.13	0.2931	0.9982	0.95	0.6973	0.9960	1.01	0.9240	0.9939	1.11	0.3732	0.9951	1.11	0.4565	0.9856	0.87	0.2786	0.7212	0.88	0.3259	0.5681	1.05	0.7104	0.9889	1.12	0.3193	0.9607
Sialic acid synthase	SIAS_HUMAN	Q9NR45	1.12	0.2424	0.5169	1.08	0.4462	0.9982	1.08	0.5285	0.9960	0.88	0.1873	0.9939	0.97	0.7346	0.9951	1.14	0.2997	0.9856	0.92	0.4328	0.8109	1.12	0.3268	0.5681	0.89	0.2560	0.9889	0.88	0.2017	0.9607
Signal recognition particle 9 kDa protein	SRP09_HUMAN	P49458	0.86	0.0327	0.2873	0.94	0.4356	0.9982	1.03	0.7428	0.9960	0.90	0.1483	0.9874	0.79	0.0011	0.9389	1.04	0.6379	0.9856	0.94	0.3913	0.7841	1.16	0.0665	0.2648	0.95	0.5256	0.9889	0.85	0.0257	0.9607
Single-stranded DNA-binding protein, mitochondrial	SSBP_HUMAN	Q04837	1.18	0.0214	0.2873	1.06	0.4160	0.9982	1.02	0.8004	0.9960	0.93	0.3515	0.9939	0.91	0.2239	0.9951	0.95	0.5543	0.9856	0.86	0.0563	0.6824	1.03	0.7104	0.8360	1.05	0.5464	0.9889	0.91	0.2245	0.9607
SLC35A4 upstream open reading frame protein	S35U4_HUMAN	L0R6Q1	0.97	0.5119	0.7292	1.04	0.4472	0.9982	1.04	0.5269	0.9960	1.07	0.1403	0.9874	1.01	0.9161	0.9951	0.92	0.1415	0.9856	0.98	0.6409	0.8892	0.95	0.3338	0.5705	1.05	0.2802	0.9889	0.99	0.9128	0.9938
Small glutamine-rich tetratricopeptide repeat-containing protein alpha	SGTA_HUMAN	O43765	1.06	0.5648	0.7665	0.99	0.9208	0.9982	1.07	0.5301	0.9960	0.87	0.1573	0.9874	0.95	0.6365	0.9951	0.98	0.8463	0.9959	0.82	0.0435	0.6824	1.14	0.2341	0.4972	0.95	0.5776	0.9889	0.91	0.3216	0.9607
Small muscular protein	SMPX_HUMAN	Q9UHP9	1.11	0.2920	0.5638	1.03	0.7954	0.9982	1.12	0.3305	0.9960	1.12	0.2593	0.9939	1.00	0.9848	0.9964	0.89	0.3475	0.9856	1.01	0.9137	0.9848	1.20	0.0867	0.2982	0.89	0.2652	0.9889	0.90	0.2757	0.9607
Smoothelin	SMTN_HUMAN	P53814	1.21	0.4907	0.7219	0.91	0.7383	0.9982	0.68	0.2224	0.9960	1.17	0.5591	0.9939	1.63	0.0849	0.9951	1.39	0.3299	0.9856	1.00	0.9978	0.9992	1.44	0.2316	0.4953	1.47	0.1713	0.9889	1.53	0.1147	0.9607
Sodium/calcium exchanger 1	NAC1_HUMAN	P32418	0.97	0.5784	0.7764	0.98	0.7222	0.9982	1.12	0.1239	0.9960	0.98	0.7963	0.9939	1.00	0.9975	0.9996	0.84	0.0258	0.9411	0.93	0.2745	0.7196	1.01	0.8610	0.9245	0.96	0.5426	0.9889	1.01	0.8329	0.9881
Sodium/potassium-transporting ATPase subunit alpha-1	AT1A1_HUMAN	P05023	0.87	0.0619	0.3038	1.00	0.9523	0.9982	1.02	0.8609	0.9960	0.87	0.0764	0.9874	0.89	0.1543	0.9951	0.95	0.5966	0.9856	0.99	0.9411	0.9903	0.83	0.0345	0.2017	0.94	0.4385	0.9889	0.95	0.5449	0.9881
Sodium/potassium-transporting ATPase subunit alpha-2	AT1A2_HUMAN	P50993	0.92	0.2468	0.5201	1.02	0.7978	0.9982	1.03	0.7215	0.9960	0.99	0.8274	0.9939	0.95	0.4818	0.9951	0.93	0.3908	0.9856	0.92	0.2217	0.7187	0.89	0.1371	0.3785	1.00	0.9933	0.9985	0.99	0.8511	0.9881
Sodium/potassium-transporting ATPase subunit alpha-3	AT1A3_HUMAN	P13637	1.01	0.9242	0.9555	1.08	0.2113	0.9982	1.00	0.9501	0.9960	0.95	0.3712	0.9939	0.96	0.4831	0.9951	1.05	0.5504	0.9856	0.91	0.1590	0.6871	0.95	0.4757	0.6794	1.04	0.5768	0.9889	1.02	0.7993	0.9881
Sodium/potassium-transporting ATPase subunit beta-1	AT1B1_HUMAN	P05026	0.94	0.3285	0.5962	1.05	0.4337	0.9982	1.03	0.6830	0.9960	0.91	0.1072	0.9874	0.95	0.3783	0.9951	0.93	0.3597	0.9856	0.90	0.1078	0.6824	0.86	0.0294	0.1909	1.00	0.9387	0.9922	0.97	0.6639	0.9881
Solute carrier family 2, facilitated glucose transporter member 4	GLUT4_HUMAN	P14672	1.20	0.0552	0.2942	1.12	0.2168	0.9982	0.97	0.8040	0.9960	1.17	0.0989	0.9874	1.14	0.1869	0.9951	0.97	0.7642	0.9857	0.97	0.7335	0.9218	0.97	0.7667	0.8739	1.24	0.0275	0.9889	1.11	0.2767	0.9607
Sorbin and SH3 domain-containing protein 1	SRBS1_HUMAN	Q9BX66	1.01	0.9180	0.9542	0.97	0.5373	0.9982	1.00	0.9492	0.9960	0.98	0.7160	0.9939	1.02	0.7581	0.9951	0.94	0.3325	0.9856	0.92	0.1718	0.6993	1.00	0.9706	0.9832	1.06	0.3074	0.9889	1.06	0.2666	0.9607
Sorbin and SH3 domain-containing protein 2	SRBS2_HUMAN	O94875	0.91	0.1870	0.4611	0.97	0.7321	0.9982	1.12	0.1839	0.9960	1.00	0.9832	0.9969	0.97	0.6608	0.9951	0.82	0.0314	0.9411	0.87	0.0846	0.6824	0.91	0.2337	0.4971	1.08	0.3133	0.9889	1.04	0.6021	0.9881
Sorcin	SORCN_HUMAN	P30626	1.11	0.1416	0.4039	1.02	0.7481	0.9982	0.97	0.7152	0.9960	0.91	0.1947	0.9939	0.92	0.2730	0.9951	1.08	0.3826	0.9856	0.86	0.0575	0.6824	1.17	0.0545	0.2454	0.95	0.5349	0.9889	0.92	0.2559	0.9607
Sortilin	SORT_HUMAN	Q99523	0.83	0.0512	0.2898	0.96	0.7141	0.9982	0.96	0.7095	0.9960	0.96	0.6482	0.9939	0.82	0.0568	0.9951	1.03	0.8230	0.9959	0.93	0.4639	0.8245	1.09	0.4422	0.6607	1.02	0.8367	0.9889	0.86	0.1146	0.9607
Sorting nexin-1	SNX1_HUMAN	Q13596	1.06	0.4399	0.6862	1.11	0.2009	0.9982	0.97	0.7054	0.9960	1.13	0.1081	0.9874	1.04	0.6182	0.9951	1.03	0.7717	0.9857	0.93	0.3572	0.7644	0.91	0.2567	0.5217	1.01	0.8626	0.9889	1.14	0.1010	0.9607
SPARC-like protein 1	SPRL1_HUMAN	Q14515	0.99	0.8811	0.9384	0.98	0.8157	0.9982	0.93	0.5490	0.9960	0.92	0.3834	0.9939	0.93	0.4704	0.9951	0.99	0.9375	0.9959	1.07	0.5286	0.8563	0.83	0.0964	0.3134	0.98	0.8744	0.9889	1.08	0.4415	0.9607
Spectrin alpha chain, non-erythrocytic 1	SPTN1_HUMAN	Q13813	0.87	0.0091	0.2530	0.97	0.5338	0.9982	1.04	0.5827	0.9960	0.96	0.4271	0.9939	0.91	0.0859	0.9951	0.96	0.5074	0.9856	0.99	0.8075	0.9460	0.98	0.7350	0.8540	1.08	0.1649	0.9889	0.98	0.6460	0.9881
Spectrin beta chain, erythrocytic	SPTB1_HUMAN	P11277	0.97	0.6441	0.8286	0.90	0.1306	0.9982	1.16	0.0594	0.9960	1.02	0.7634	0.9939	1.05	0.4810	0.9951	0.84	0.0369	0.9411	0.99	0.8343	0.9516	1.11	0.1554	0.4053	1.01	0.8516	0.9889	1.06	0.3914	0.9607
Spectrin beta chain, non-erythrocytic 1	SPTB2_HUMAN	Q01082	0.89	0.0340	0.2873	0.98	0.7800	0.9982	1.05	0.4390	0.9960	0.95	0.3197	0.9939	0.89	0.0415	0.9951	0.97	0.6433	0.9856	0.99	0.8784	0.9720	1.01	0.8399	0.9140	1.07	0.2046	0.9889	0.98	0.6678	0.9881
Spermatogenesis-associated protein 20	SPT20_HUMAN	Q8TB22	0.89	0.2081	0.4843	1.08	0.4137	0.9982	0.88	0.2728	0.9960	0.92	0.4015	0.9939	0.93	0.4422	0.9951	1.22	0.0862	0.9856	1.04	0.7212	0.9201	1.18	0.1255	0.3597	1.07	0.5130	0.9889	0.92	0.4133	0.9607
Spliceosome RNA helicase DDX39B	DX39B_HUMAN	Q13838	0.92	0.1915	0.4689	1.10	0.1476	0.9982	1.03	0.6583	0.9960	0.92	0.2350	0.9939	0.90	0.1151	0.9951	1.01	0.8727	0.9959	1.01	0.8723	0.9709	1.00	0.9692	0.9831	0.93	0.3007	0.9889	0.89	0.0670	0.9607
Splicing factor 3B subunit 1	SF3B1_HUMAN	O75533	1.02	0.7966	0.9014	0.92	0.2677	0.9982	1.07	0.3955	0.9960	1.10	0.1639	0.9874	1.09	0.2320	0.9951	0.96	0.6770	0.9856	0.97	0.7054	0.9121	1.09	0.2625	0.5247	1.02	0.8288	0.9889	1.05	0.4591	0.9607
Splicing factor 3B subunit 2	SF3B2_HUMAN	Q13435	0.99	0.8503	0.9276	0.94	0.4653	0.9982	1.05	0.6068	0.9960	1.12	0.1611	0.9874	1.09	0.2926	0.9951	0.94	0.4958	0.9856	1.08	0.3512	0.7593	0.96	0.6263	0.7895	1.00	0.9823	0.9976	0.99	0.8817	0.9938
Splicing factor 3B subunit 3	SF3B3_HUMAN	Q15393	0.94	0.2655	0.5347	0.95	0.3005	0.9982	1.14	0.0228	0.9960	1.03	0.5699	0.9939	0.96	0.4751	0.9951	0.87	0.0311	0.9411	1.01	0.8053	0.9457	1.02	0.7492	0.8625	1.00	0.9625	0.9961	0.97	0.5717	0.9881
Splicing factor, proline- and glutamine-rich	SFPQ_HUMAN	P23246	0.85	0.0477	0.2898	1.02	0.7993	0.9982	1.22	0.0321	0.9960	0.98	0.7877	0.9939	0.86	0.0801	0.9951	0.81	0.0327	0.9411	1.14	0.1185	0.6868	0.87	0.1369	0.3785	1.00	0.9749	0.9976	0.92	0.3068	0.9607
SPRY domain-containing protein 4	SPRY4_HUMAN	Q8WW59	1.28	0.0318	0.2873	1.14	0.2458	0.9982	0.99	0.9143	0.9960	0.87	0.2322	0.9939	0.96	0.7281	0.9951	1.02	0.8956	0.9959	0.81	0.0934	0.6824	1.27	0.0604	0.2529	0.96	0.7264	0.9889	1.00	0.9902	0.9992
SRA stem-loop-interacting RNA-binding protein, mitochondrial	SLIRP_HUMAN	Q9GZT3	1.22	0.0035	0.2012	1.07	0.3463	0.9982	0.91	0.2530	0.9960	1.08	0.2903	0.9939	1.13	0.0973	0.9951	1.08	0.3522	0.9856	0.96	0.6083	0.8709	0.94	0.4626	0.6748	1.09	0.2521	0.9889	1.13	0.0799	0.9607
Src substrate cortactin	SRC8_HUMAN	Q14247	1.00	0.9993	0.9993	0.96	0.4917	0.9982	0.99	0.8120	0.9960	1.03	0.5479	0.9939	1.01	0.8193	0.9951	1.01	0.9205	0.9959	1.04	0.5388	0.8573	1.16	0.0113	0.1499	1.06	0.2970	0.9889	0.97	0.5733	0.9881
Staphylococcal nuclease domain-containing protein 1	SND1_HUMAN	Q7KZF4	0.83	0.0057	0.2453	0.99	0.9251	0.9982	1.05	0.5539	0.9960	0.98	0.8031	0.9939	0.93	0.3039	0.9951	0.96	0.6356	0.9856	1.03	0.6633	0.8929	0.98	0.7986	0.8908	0.91	0.2063	0.9889	0.95	0.4919	0.9712
StAR-related lipid transfer protein 7, mitochondrial	STAR7_HUMAN	Q9NQZ5	1.23	0.0667	0.3105	1.04	0.7501	0.9982	1.05	0.7222	0.9960	0.80	0.0438	0.9874	1.04	0.7116	0.9951	0.84	0.2071	0.9856	0.90	0.3707	0.7673	1.00	0.9959	0.9972	0.98	0.8422	0.9889	1.16	0.1825	0.9607
Starch-binding domain-containing protein 1	STBD1_HUMAN	O95210	0.89	0.1767	0.4521	0.98	0.8497	0.9982	1.04	0.6744	0.9960	1.06	0.5103	0.9939	0.90	0.2434	0.9951	0.86	0.1493	0.9856	0.94	0.4874	0.8376	0.94	0.5046	0.7017	1.06	0.5163	0.9889	0.93	0.4181	0.9607
Stathmin	STMN1_HUMAN	P16949	1.04	0.6790	0.8437	1.03	0.7332	0.9982	0.98	0.8723	0.9960	1.01	0.9312	0.9939	0.94	0.5130	0.9951	1.08	0.4937	0.9856	1.08	0.4445	0.8180	0.96	0.7081	0.8360	0.95	0.6037	0.9889	0.90	0.2231	0.9607
Stomatin-like protein 2, mitochondrial	STML2_HUMAN	Q9UJZ1	1.04	0.4957	0.7237	1.04	0.5677	0.9982	0.89	0.1207	0.9960	1.07	0.2567	0.9939	1.09	0.1887	0.9951	1.05	0.5270	0.9856	1.10	0.1403	0.6871	0.99	0.8770	0.9321	1.00	0.9837	0.9976	1.06	0.3149	0.9607
Stress-70 protein, mitochondrial	GRP75_HUMAN	P38646	1.06	0.1992	0.4787	1.01	0.8845	0.9982	0.94	0.2708	0.9960	0.95	0.2439	0.9939	0.98	0.7091	0.9951	1.05	0.3736	0.9856	0.93	0.1105	0.6824	1.05	0.3432	0.5778	1.08	0.0789	0.9889	1.05	0.2958	0.9607
Stress-induced-phosphoprotein 1	STIP1_HUMAN	P31948	0.98	0.7019	0.8598	0.95	0.3432	0.9982	1.00	0.9828	0.9960	0.99	0.8732	0.9939	0.97	0.5294	0.9951	1.06	0.3598	0.9856	0.93	0.2034	0.7149	1.15	0.0190	0.1650	0.93	0.1610	0.9889	0.94	0.2433	0.9607
Striatin	STRN_HUMAN	O43815	0.98	0.7662	0.8882	0.93	0.2320	0.9982	1.07	0.3278	0.9960	0.97	0.6653	0.9939	0.99	0.9216	0.9951	0.91	0.2458	0.9856	0.89	0.0772	0.6824	1.07	0.3201	0.5613	0.92	0.1920	0.9889	0.95	0.4090	0.9607

Striatin-3	STRN3_HUMAN	Q13033	1.02	0.7864	0.8986	0.96	0.4800	0.9982	0.98	0.7460	0.9960	1.00	0.9916	0.9981	0.97	0.6586	0.9951	0.99	0.9253	0.9959	0.97	0.6736	0.8951	1.04	0.5233	0.7179	0.95	0.4429	0.9889	0.96	0.4932	0.9712
Succinate dehydrogenase [ubiquinone] flavoprotein subunit, mitochondr	SDHA_HUMAN	P31040	1.22	0.0040	0.2034	1.07	0.3115	0.9982	0.96	0.6040	0.9960	0.97	0.6166	0.9939	1.06	0.4304	0.9951	1.04	0.6393	0.9856	0.91	0.2035	0.7149	1.05	0.5367	0.7260	1.11	0.1663	0.9889	1.16	0.0306	0.9607
Succinate dehydrogenase [ubiquinone] iron-sulfur subunit, mitochondria	SDHB_HUMAN	P21912	1.15	0.0567	0.2942	1.06	0.4001	0.9982	1.03	0.7192	0.9960	0.96	0.6167	0.9939	1.05	0.5000	0.9951	0.94	0.5337	0.9856	0.89	0.1523	0.6871	1.10	0.2704	0.5288	1.03	0.6931	0.9889	1.14	0.0647	0.9607
Succinate dehydrogenase assembly factor 2, mitochondrial	SDHF2_HUMAN	Q9NX18	1.13	0.2115	0.4843	0.97	0.7602	0.9982	0.98	0.8871	0.9960	0.90	0.2839	0.9939	0.95	0.5796	0.9951	1.02	0.8791	0.9959	1.12	0.2476	0.7187	0.98	0.8527	0.9202	1.09	0.3592	0.9889	1.04	0.7039	0.9881
Succinate dehydrogenase assembly factor 4, mitochondrial	SDHF4_HUMAN	Q5VUM1	0.99	0.9501	0.9699	0.92	0.4808	0.9982	0.94	0.6823	0.9960	0.97	0.8350	0.9939	0.90	0.4180	0.9951	1.10	0.5471	0.9856	0.79	0.0732	0.6824	1.38	0.0165	0.1636	1.08	0.5682	0.9889	0.96	0.7605	0.9881
Succinate--CoA ligase [ADP-forming] subunit beta, mitochondrial	SUCB1_HUMAN	Q9P2R7	1.13	0.0781	0.3281	1.04	0.5657	0.9982	0.94	0.4772	0.9960	1.02	0.7316	0.9939	1.01	0.9137	0.9951	1.06	0.5368	0.9856	0.98	0.7673	0.9291	1.11	0.1813	0.4355	1.05	0.5355	0.9889	1.02	0.7577	0.9881
Succinate--CoA ligase [ADP/GDP-forming] subunit alpha, mitochondrial	SUCA_HUMAN	P53597	1.15	0.0399	0.2873	1.04	0.5797	0.9982	0.87	0.0940	0.9960	0.99	0.8711	0.9939	1.01	0.9200	0.9951	1.16	0.0740	0.9641	0.97	0.7290	0.9213	1.10	0.2308	0.4953	1.06	0.4228	0.9889	1.04	0.6186	0.9881
Succinate--CoA ligase [GDP-forming] subunit beta, mitochondrial	SUCB2_HUMAN	Q96I99	1.11	0.1201	0.3810	1.00	0.9698	0.9982	0.93	0.3050	0.9960	0.98	0.7904	0.9939	1.01	0.8610	0.9951	1.10	0.2283	0.9856	1.00	0.9528	0.9923	1.03	0.6609	0.8074	1.02	0.7782	0.9889	1.01	0.8518	0.9881
Succinate-semialdehyde dehydrogenase, mitochondrial	SSDH_HUMAN	P51649	1.23	0.0290	0.2873	1.03	0.7930	0.9982	0.89	0.3020	0.9960	0.98	0.8679	0.9939	1.07	0.5217	0.9951	1.09	0.4748	0.9856	0.97	0.7336	0.9218	1.21	0.0758	0.2841	1.05	0.6001	0.9889	1.09	0.3695	0.9607
Succinyl-CoA:3-ketoacid coenzyme A transferase 1, mitochondrial	SCOT1_HUMAN	P55809	1.02	0.8328	0.9190	0.95	0.6088	0.9982	1.03	0.7826	0.9960	0.93	0.4714	0.9939	0.88	0.2257	0.9951	0.98	0.8535	0.9959	0.84	0.1125	0.6824	1.18	0.1371	0.3785	1.07	0.4999	0.9889	0.95	0.5937	0.9881
Sulfide:quinone oxidoreductase, mitochondrial	SQOR_HUMAN	Q9Y6N5	0.98	0.7646	0.8882	1.05	0.4589	0.9982	1.06	0.4384	0.9960	1.14	0.0382	0.9874	1.08	0.2340	0.9951	0.90	0.2083	0.9856	1.08	0.2884	0.7212	0.86	0.0297	0.1909	0.99	0.8778	0.9889	1.02	0.7572	0.9881
Sulfite oxidase, mitochondrial	SUOX_HUMAN	P51687	1.27	0.0031	0.2005	1.10	0.2664	0.9982	0.96	0.6752	0.9960	0.97	0.6879	0.9939	1.05	0.5551	0.9951	1.05	0.6493	0.9856	0.93	0.3836	0.7787	1.09	0.3667	0.5972	1.02	0.8596	0.9889	1.03	0.6944	0.9881
SUN domain-containing protein 2	SUN2_HUMAN	Q9UH99	0.93	0.4452	0.6908	1.03	0.7487	0.9982	1.06	0.5801	0.9960	1.06	0.4815	0.9939	1.04	0.6339	0.9951	0.92	0.4326	0.9856	1.00	0.9867	0.9992	0.77	0.0080	0.1361	1.03	0.7165	0.9889	1.07	0.4201	0.9607
Superoxide dismutase [Cu-Zn]	SODC_HUMAN	P00441	1.11	0.2603	0.5311	0.98	0.8487	0.9982	0.93	0.4569	0.9960	0.89	0.2017	0.9939	0.96	0.6477	0.9951	1.15	0.2002	0.9856	0.86	0.1161	0.6824	1.17	0.1188	0.3515	0.98	0.8129	0.9889	0.99	0.9067	0.9938
Superoxide dismutase [Mn], mitochondrial	SODM_HUMAN	P04179	1.13	0.1402	0.4030	1.09	0.2815	0.9982	0.94	0.5353	0.9960	0.96	0.5765	0.9939	0.97	0.6754	0.9951	1.06	0.5572	0.9856	0.89	0.1583	0.6871	1.05	0.6163	0.7833	1.01	0.9134	0.9889	0.96	0.5810	0.9881
Supervillin	SVIL_HUMAN	O95425	0.98	0.7508	0.8824	0.98	0.8108	0.9982	1.08	0.3881	0.9960	1.05	0.5111	0.9939	1.04	0.6524	0.9951	0.91	0.2846	0.9856	0.95	0.5322	0.8567	0.93	0.4147	0.6399	1.03	0.6712	0.9889	1.04	0.5859	0.9881
Synaptic vesicle membrane protein VAT-1 homolog	VAT1_HUMAN	Q99536	0.86	0.0341	0.2873	0.98	0.8054	0.9982	1.11	0.1921	0.9960	1.11	0.1306	0.9874	0.95	0.4567	0.9951	0.87	0.1079	0.9856	1.26	0.0013	0.6779	0.92	0.3061	0.5503	0.92	0.2274	0.9889	0.91	0.1687	0.9607
Synaptic vesicle membrane protein VAT-1 homolog-like	VAT1L_HUMAN	Q9HCJ6	0.85	0.1565	0.4253	0.87	0.2212	0.9982	1.34	0.0302	0.9960	0.97	0.7665	0.9939	0.93	0.5667	0.9951	0.82	0.1650	0.9856	0.95	0.7116	0.9149	1.28	0.0583	0.2503	0.81	0.0829	0.9889	1.01	0.9089	0.9938
Synaptojanin-2-binding protein	SYJ2B_HUMAN	P57105	0.98	0.7850	0.8986	1.09	0.2267	0.9982	0.95	0.5497	0.9960	1.04	0.6266	0.9939	0.95	0.5134	0.9951	0.96	0.6848	0.9856	0.96	0.5768	0.8630	0.95	0.5036	0.7017	1.12	0.1337	0.9889	0.98	0.7344	0.9881
Synaptopodin	SYNPO_HUMAN	Q8N3V7	0.97	0.5244	0.7378	0.96	0.4569	0.9982	1.03	0.5930	0.9960	1.00	0.9603	0.9942	1.02	0.6974	0.9951	0.90	0.1232	0.9856	1.03	0.5474	0.8600	0.97	0.5708	0.7500	0.99	0.8427	0.9889	1.03	0.6202	0.9881
Synaptopodin 2-like protein	SYP2L_HUMAN	Q9H987	0.95	0.5301	0.7430	0.93	0.3787	0.9982	0.92	0.4210	0.9960	1.11	0.2121	0.9939	1.03	0.7743	0.9951	1.01	0.9302	0.9959	0.99	0.9176	0.9848	1.05	0.5832	0.7599	1.07	0.4557	0.9889	0.99	0.9452	0.9938
Synaptopodin-2	SYNP2_HUMAN	Q9UMS6	0.91	0.1519	0.4208	1.01	0.8846	0.9982	1.06	0.4476	0.9960	0.90	0.1248	0.9874	0.86	0.0414	0.9951	0.91	0.2978	0.9856	0.85	0.0280	0.6824	0.99	0.8477	0.9185	1.10	0.1729	0.9889	1.00	0.9823	0.9992
Syndecan-2	SDC2_HUMAN	P34741	0.93	0.4668	0.7074	1.02	0.8300	0.9982	1.09	0.4724	0.9960	1.13	0.2346	0.9939	0.96	0.7235	0.9951	0.88	0.3288	0.9856	1.16	0.1844	0.7149	0.77	0.0263	0.1832	0.91	0.3758	0.9889	0.96	0.7369	0.9881
Synemin	SYNEM_HUMAN	O15061	0.91	0.1272	0.3936	1.04	0.4985	0.9982	1.15	0.0612	0.9960	0.95	0.4086	0.9939	0.91	0.1343	0.9951	0.85	0.0372	0.9411	0.92	0.2008	0.7149	0.92	0.2411	0.5050	1.05	0.4212	0.9889	1.00	0.9450	0.9938
Syntaxin-4	STX4_HUMAN	Q12846	0.94	0.2523	0.5219	1.06	0.2293	0.9982	1.01	0.8552	0.9960	0.99	0.7906	0.9939	0.91	0.0624	0.9951	0.92	0.1986	0.9856	0.99	0.8136	0.9478	0.92	0.1245	0.3578	0.94	0.2238	0.9889	0.93	0.1741	0.9607
Syntaxin-binding protein 3	STXB3_HUMAN	O00186	0.94	0.2930	0.5639	1.12	0.0573	0.9982	1.04	0.5726	0.9960	1.06	0.3702	0.9939	1.00	0.9488	0.9951	0.94	0.4086	0.9856	1.11	0.0979	0.6824	0.85	0.0119	0.1527	0.92	0.1685	0.9889	0.95	0.3682	0.9607
T-complex protein 1 subunit alpha	TCPA_HUMAN	P17987	0.96	0.5250	0.7378	1.07	0.2295	0.9982	1.00	0.9883	0.9960	1.12	0.0500	0.9874	0.96	0.4655	0.9951	0.98	0.7805	0.9897	0.99	0.8900	0.9753	0.93	0.2570	0.5217	1.01	0.9319	0.9904	0.96	0.4625	0.9607
T-complex protein 1 subunit beta	TCPB_HUMAN	P78371	0.98	0.6883	0.8496	1.05	0.3669	0.9982	1.08	0.2530	0.9960	1.09	0.1186	0.9874	1.01	0.8481	0.9951	0.91	0.1883	0.9856	1.05	0.4582	0.8237	0.87	0.0322	0.2014	0.93	0.2332	0.9889	0.98	0.6664	0.9881
T-complex protein 1 subunit delta	TCPD_HUMAN	P50991	0.98	0.7228	0.8735	1.07	0.3129	0.9982	0.99	0.9450	0.9960	1.13	0.0457	0.9874	1.01	0.9001	0.9951	1.01	0.9460	0.9959	1.09	0.2136	0.7171	0.84	0.0116	0.1499	0.94	0.3607	0.9889	0.94	0.3509	0.9607
T-complex protein 1 subunit epsilon	TCPE_HUMAN	P48643	0.97	0.6037	0.7965	1.10	0.1603	0.9982	1.02	0.8010	0.9960	1.10	0.1427	0.9874	0.98	0.7847	0.9951	0.96	0.6245	0.9856	1.05	0.5242	0.8558	0.83	0.0102	0.1449	0.94	0.3408	0.9889	0.96	0.5487	0.9881
T-complex protein 1 subunit eta	TCPH_HUMAN	Q99832	0.97	0.7072	0.8642	1.11	0.1705	0.9982	1.04	0.6881	0.9960	1.15	0.0727	0.9874	0.99	0.9353	0.9951	0.94	0.5224	0.9856	1.17	0.0652	0.6824	0.80	0.0080	0.1361	0.92	0.2956	0.9889	0.93	0.3752	0.9607
T-complex protein 1 subunit gamma	TCPG_HUMAN	P49368	0.94	0.2978	0.5691	1.04	0.5682	0.9982	1.02	0.7313	0.9960	1.08	0.2191	0.9939	0.97	0.6563	0.9951	0.95	0.4822	0.9856	1.07	0.2715	0.7196	0.90	0.1044	0.3288	0.92	0.1761	0.9889	0.94	0.3185	0.9607
T-complex protein 1 subunit theta	TCPQ_HUMAN	P50990	1.01	0.7884	0.8986	1.08	0.1648	0.9982	1.08	0.1969	0.9960	1.07	0.1906	0.9939	0.98	0.7315	0.9951	0.89	0.0652	0.9641	1.02	0.7933	0.9431	0.89	0.0393	0.2155	0.91	0.0689	0.9889	0.95	0.2982	0.9607
T-complex protein 1 subunit zeta	TCPZ_HUMAN	P40227	0.91	0.2670	0.5351	1.04	0.6576	0.9982	1.04	0.6953	0.9960	1.10	0.2435	0.9939	0.97	0.7475	0.9951	0.94	0.5712	0.9856	1.16	0.0755	0.6824	0.81	0.0180	0.1645	0.93	0.3972	0.9889	0.91	0.2553	0.9607
Talin-1	TLN1_HUMAN	Q9Y490	0.94	0.1821	0.4573	0.98	0.6828	0.9982	1.00	0.9578	0.9960	0.98	0.6115	0.9939	0.96	0.3419	0.9951	1.02	0.7600	0.9857	1.02	0.7264	0.9205	0.94	0.1888	0.4471	0.97	0.5622	0.9889	0.98	0.5678	0.9881
Talin-2	TLN2_HUMAN	Q9Y4G6	0.98	0.7161	0.8711	1.00	0.9964	0.9985	1.06	0.4507	0.9960	0.99	0.8277	0.9939	0.95	0.4582	0.9951	0.92	0.2758	0.9856	0.87	0.0339	0.6824	1.04	0.5742	0.7520	1.03	0.7049	0.9889	1.00	0.9421	0.9938
Tapasin	TPSN_HUMAN	O15533	0.94	0.5843	0.7830	1.13	0.2880	0.9982	1.08	0.5782	0.9960	0.98	0.8889	0.9939	0.98	0.8775	0.9951	0.88	0.3845	0.9856	1.22	0.1084	0.6824	0.76	0.0292	0.1909	0.93	0.5414	0.9889	0.89	0.3236	0.9607
TAR DNA-binding protein 43	TADBP_HUMAN	Q13148	0.97	0.5117	0.7292	0.96	0.4372	0.9982	0.94	0.3227	0.9960	0.94	0.2410	0.9939	0.95	0.3620	0.9951	1.05	0.4662	0.9856	0.99	0.7805	0.9362	1.10	0.0770	0.2846	1.03	0.5895	0.9889	0.95	0.3098	0.9607
Target of Nesh-SH3	TARSH_HUMAN	Q7Z7G0	0.78	0.1201	0.3810	0.91	0.5614	0.9982	0.91	0.6165	0.9960	1.02	0.9227	0.9939	0.79	0.1611	0.9951	1.18	0.4076	0.9856	1.07	0.7005	0.9088	0.82	0.2886	0.5420	1.14	0.4374	0.9889	1.00	0.9857	0.9992
Telethonin	TELT_HUMAN	O15273	1.06	0.6761	0.8429	1.20	0.1579	0.9982	1.18	0.2665	0.9960	1.15	0.2989	0.9939	1.08	0.5794	0.9951	0.82	0.2088	0.9856	0.81	0.1376	0.6871	0.83	0.2082	0.4733	1.16	0.2573	0.9889	1.04	0.7427	0.9881
Tenascin	TENA_HUMAN	P24821	0.75	0.0984	0.3566	0.99	0.9311	0.9982	1.40	0.0870	0.9960	0.94	0.6982	0.9939	0.92	0.6354	0.9951	0.66	0.0469	0.9535	1.00	0.9934	0.9992	0.68	0.0404	0.2167	0.69	0.0356	0.9889	0.74	0.0753	0.9607
Tenascin-X	TENX_HUMAN	P22105	0.92	0.2428	0.5171	0.94	0.3995	0.9982	1.08	0.3436	0.9960	0.92	0.2482	0.9939	0.91	0.2246	0.9951	0.92	0.3559	0.9856	0.94	0.3940	0.7856	0.96	0.6668	0.8098	1.08	0.3160	0.9889	0.99	0.8622	0.9881
Tensin-1	TENS1_HUMAN	Q9HBL0	1.05	0.3317	0.5968	1.13	0.0117	0.9982	1.01	0.8834	0.9960	1.00	0.9215	0.9939	1.02	0.7736	0.9951	0.98	0.7294	0.9857	0.98	0.6648	0.8929	0.91	0.1043	0.3288	1.02	0.6878	0.9889	1.07	0.1784	0.9607
Tetratricopeptide repeat protein 1	TTC1_HUMAN	Q99614	0.98	0.8429	0.9230	0.95	0.5202	0.9982	1.13	0.1691	0.9960	1.02	0.8336	0.9939	0.99	0.8524	0.9951	0.93	0.4113	0.9856	0.97	0.7416	0.9230	1.05	0.6007	0.7719	1.00	0.9871	0.9976	0.87	0.0711	0.9607
Thioredoxin	THIO_HUMAN	P10599	0.99	0.8669	0.9356	1.00	0.9899	0.9985	1.06	0.3981	0.9960	0.99	0.8522	0.9939	0.94	0.3151	0.9951	1.03	0.6683	0.9856	0.95	0.4496	0.8181	1.18	0.0155	0.1606	0.90	0.1200	0.9889	0.95	0.3984	0.9607
Thioredoxin domain-containing protein 12	TXD12_HUMAN	O95881	1.05	0.4036	0.6612	1.01	0.8801	0.9982	1.07	0.2734	0.9960	0.96	0.4838	0.9939	1.00	0.9836	0.9964	0.93	0.2725	0.9856	0.97	0.5949	0.8646	0.97	0.6357	0.7937	0.95	0.3772	0.9889	0.91	0.1027	0.9607
Thioredoxin domain-containing protein 5	TXND5_HUMAN	Q8NBS9	0.93	0.3049	0.5744	0.99	0.8751	0.9982	1.06	0.4681	0.9960	1.04	0.5922	0.9939	0.99	0.8615	0.9951	0.97	0.7162	0.9857	1.14	0.0694	0.6824	0.87	0.0590	0.2505	0.92	0.2505	0.9889	0.94	0.3842	0.9607
Thioredoxin reductase 1, cytoplasmic	TRXR1_HUMAN	Q16881	1.02	0.7456	0.8821	0.97	0.6187	0.9982	0.95	0.5087	0.9960	0.99	0.8608	0.9939	0.99	0.8951	0.9951	1.06	0.5054	0.9856	0.99	0.9353	0.9903	1.10	0.1997	0.4593	0.85	0.0199	0.9889	0.96	0.5666	0.9881
Thioredoxin reductase 2, mitochondrial	TRXR2_HUMAN	Q9NNW7	1.15	0.1116	0.3738	0.95	0.5214	0.9982	0.99	0.9032	0.9960	0.96	0.6261	0.9939	0.97	0.7511	0.9951	1.05	0.6374	0.9856	0.95	0.5763	0.8630	1.13	0.1994	0.4593	1.03	0.7149	0.9889	0.93	0.3942	0.9607
Thioredoxin-dependent peroxide reductase, mitochondrial	PRDX3_HUMAN	P30048	1.13	0.1140	0.3748	1.04	0.6131	0.9982	0.99	0.9196	0.9960	0.90	0.1593	0.9874	0.95	0.4936	0.9951	1.04	0.6974	0.9856	0.81	0.0123	0.6779	1.17	0.0693	0.2698	1.02	0.7955	0.9889	1.01	0.8582	0.9881
Thioredoxin-like protein 1	TXNL1_HUMAN	O43396	1.04	0.6028	0.7965	0.95	0.4972	0.9982	0.98	0.7783	0.9960	1.02	0.7920	0.9939	1.04	0.6312	0.9951	1.06	0.5398	0.9856	0.94	0.4077	0.7885	1.20	0.0205	0.1665	1.00	0.9601	0.9961	1.10	0.1851	0.9607
Thioredoxin-related transmembrane protein 4	TMX4_HUMAN	Q9H1E5	0.97	0.5537	0.7569	1.05	0.4286	0.9982	1.09	0.1882	0.9960	1.06	0.3322	0.9939	1.03	0.6523	0.9951	0.90	0.1296	0.9856	1.03	0.5960	0.8646	0.99	0.9122	0.9526	0.98	0.7955	0.9889	1.05	0.3778	0.9607
Thioredoxin, mitochondrial	THIOM_HUMAN	Q99757	1.12	0.1843	0.4603	1.04	0.6233	0.9982	0.90	0.2825	0.9960	0.89	0.1682	0.9874	0.92	0.3304	0.9951	1.16	0.1494	0.9856	0.86	0.0964	0.6824	1.13	0.1995	0.4593	1.00	0.9767	0.9976	0.93	0.4161	0.9607
Thiosulfate sulfurtransferase	THTR_HUMAN	Q16762	1.12	0.1665	0.4371	1.04	0.6589	0.9982	0.87	0.1320	0.9960	1.02	0.8074	0.9939	1.02	0.7860	0.9951	1.17	0.1209	0.9856	1.16	0.0881	0.6824	1.02	0.8534	0.9202	0.92	0.2928	0.9889	0.98	0.8231	0.9881
Thrombospondin-4	TSP4_HUMAN	P35443	0.83	0.2121	0.4843	0.77	0.0881	0.9982	1.05	0.7715	0.9960	0.91	0.5244	0.9939	0.87	0.3754	0.9951	0.90	0.5891	0.9856	1.10	0.5642	0.8601	0.83	0.2797	0.5337	0.94	0.6753	0.9889	0.83	0.2362	0.9607
Thymidine phosphorylase	TYPH_HUMAN	P19971	1.04	0.7243	0.8735	1.06	0.5798	0.9982	0.93	0.5301	0.9960	1.13	0.2127	0.9939	1.12	0.2669	0.9951	1.05	0.6694	0.9856	1.05	0.6490	0.8897	0.72	0.0019	0.0815	0.91	0.3469	0.9889	0.98	0.8496	0.9881
Thymosin beta-4	TYB4_HUMAN	P62328	0.84	0.1069	0.3670	1.00	0.9648	0.9982	0.97	0.8172	0.9960	0.92	0.4098	0.9939	0.82	0.0799	0.9951	1.10	0.4797	0.9856	1.00	0.9990	0.9992	1.01	0.9595	0.9773	0.88	0.2515	0.9889	0.80	0.0351	0.9607
Tight junction protein ZO-1	ZO1_HUMAN	Q07157	0.97	0.4598	0.7012	0.95	0.2648	0.9982	1.05	0.2700	0.9960	0.99	0.8118	0.9939	1.01	0.7322	0.9951	0.95	0.3468	0.9856	1.02	0.6680	0.8929	1.04	0.4423	0.6607	1.05	0.2299	0.9889	1.01	0.8253	0.9881
Titin	TITIN_HUMAN	Q8WZ42	1.08	0.5099	0.7292	1.05	0.6813	0.9982	1.12	0.4088	0.9960	0.86	0.1916	0.9939	0.88	0.3202	0.9951	0.86	0.3038	0.9856	0.76	0.0335	0.6824	1.08	0.5529	0.7377	1.22	0.1095	0.9889	1.12	0.3358	0.9607
TOM1-like protein 2	TM1L2_HUMAN	Q6ZVM7	0.98	0.6297	0.8170	0.93	0.1436	0.9982	1.02	0.7601	0.9960	1.06	0.2848	0.9939	1.02	0.6683	0.9951	1.03	0.6556	0.9856	1.02	0.7665	0.9291	1.11	0.0805	0.2899	0.99	0.7911	0.9889	0.95	0.2979	0.9607
Torsin-1A-interacting protein 1	TOIP1_HUMAN	Q5JTV8	0.87	0.0334	0.2873	0.98	0.7180	0.9982	1.00	0.9984	0.9990	0.99	0.8161	0.9939	0.88	0.0644	0.9951	0.95	0.5424	0.9856	1.02	0.7497	0.9272	0.91	0.2159	0.4816	1.04	0.5253	0.9889	0.93	0.2806	0.9607
Trans-Golgi network integral membrane protein 2	TGON2_HUMAN	O43493	0.85	0.0351	0.2873	1.02	0.7634	0.9982	1.01	0.9044	0.9960	1.02	0.8048	0.9939	1.03	0.7263	0.9951	0.99	0.8946	0.9959	1.03	0.7060	0.9121	0.83	0.0273	0.1861	1.04	0.6251	0.9889	1.01	0.8855	0.9938
Transaldolase	TALDO_HUMAN	P37837	1.05	0.5552	0.7582	1.02	0.8542	0.9982	0.96	0.6800	0.9960	0.87	0.0701	0.9874	0.97	0.7481	0.9951	1.14	0.1849	0.9856	0.93	0.4116	0.7917	1.04	0.6282	0.7896	0.90	0.2099	0.9889	0.97	0.6845	0.9881
Transcription factor A, mitochondrial	TFAM_HUMAN	Q00059	1.08	0.1313	0.3958	1.02	0.6430	0.9982	0.92	0.1503	0.9960	1.07	0.2167	0.9939	1.02	0.6982	0.9951	1.05	0.4200	0.9856	0.99	0.8226	0.9481	1.05	0.4224	0.6454	1.07	0.2056	0.9889	1.02	0.7390	0.9881
Transcriptional activator protein Pur-alpha	PURA_HUMAN	Q00577	1.06	0.3433	0.6081	1.06	0.3510	0.9982	1.03	0.6576	0.9960	0.99	0.8558	0.9939	0.98	0.7170	0.9951	0.98	0.8006	0.9957	0.90	0.0745	0.6824	1.05	0.4478	0.6635	1.09	0.1310	0.9889	1.02	0.6973	0.9881
Transcriptional coactivator YAP1	YAP1_HUMAN	P46937	1.04	0.5621	0.7643	0.95	0.4454	0.9982	0.97	0.6706	0.9960	0.95	0.4084	0.9939	0.99	0.9372	0.9951	1.03	0.7366	0.9857	1.03	0.6262	0.8830	1.01	0.8546	0.9202	0.96	0.5189	0.9889	0.94	0.2970	0.9607
Transforming acidic coiled-coil-containing protein 2	TACC2_HUMAN	O95359	1.12	0.0840	0.3334	0.95	0.4433	0.9982	0.95	0.5368	0.9960	0.95	0.4782	0.9939	1.03	0.7013	0.9951	1.03	0.7570	0.9857	0.87	0.0405	0.6824	1.08	0.3187	0.5613	1.00	0.9564	0.9961	0.99	0.8981	0.9938
Transforming growth factor-beta-induced protein ig-h3	BGH3_HUMAN	Q15582	0.95	0.6004	0.7949	0.93	0.4916	0.9982	0.99	0.9511	0.9960	0.97	0.8014	0.9939	1.02	0.8427	0.9951	0.93	0.5838	0.9856	1.06	0.6002	0.8646	0.70	0.0020	0.0815	0.96	0.6849	0.9889	0.96	0.7151	0.9881
Transforming protein RhoA	RHOA_HUMAN	P61586	0.82	0.0018	0.2005	1.00	0.9845	0.9985	1.08	0.3041	0.9960	1.00	0.9408	0.9939	0.90	0.1265	0.9951	0.92	0.3078	0.9856	1.05	0.4785	0.8352	0.96	0.6293	0.7896	0.93	0.3300	0.9889	0.89	0.0884	0.9607
Transgelin	TAGL_HUMAN	Q01995	1.04	0.7181	0.8715	1.18	0.1451	0.9982	0.98	0.8482	0.9960	0.85	0.1296	0.9874	0.85	0.1585	0.9951	1.11	0.4523	0.9856	0.85	0.1645	0.6896	0.78	0.0403	0.2167	0.96	0.6909	0.9889	1.04	0.7484	0.9881
Transgelin-2	TAGL2_HUMAN	P37802	1.07	0.3259	0.5935	1.04	0.5855	0.9982	0.98	0.8156	0.9960	0.90	0.1414	0.9874	0.93	0.3440	0.9951	1.10	0.2588	0.9856	0.97	0.7010	0.9088	0.99	0.8827	0.9368	0.94	0.4194	0.9889	0.94	0.3574	0.9607
Transitional endoplasmic reticulum ATPase	TERA_HUMAN	P55072	1.07	0.1407	0.4031	0.99	0.8519	0.9982	1.01	0.8191	0.9960	1.03	0.5764	0.9939	1.01	0.9129	0.9951	0.97	0.5482	0.9856	0.96	0.3981	0.7880	1.05	0.3457	0.5794	0.98	0.6826	0.9889	0.99	0.8798	0.9938
Transketolase	TKT_HUMAN	P29401	0.90	0.2027	0.4813	1.04	0.6510	0.9982	1.02	0.8316	0.9960	0.99	0.8826	0.9939	0.91	0.2505	0.9951	1.06	0.5828	0.9856	1.13	0.1459	0.6871	0.91	0.2940	0.5464	0.83	0.0208	0.9889	0.87	0.0774	0.9607
Translation initiation factor IF-3, mitochondrial	IF3M_HUMAN	Q9H2K0	1.01	0.9302	0.9592	0.97	0.6520	0.9982	1.05	0.4800	0.9960	1.05	0.4206	0.9939	0.99	0.8714	0.9951	0.94	0.4286	0.9856	0.92	0.1947	0.7149	1.04	0.6013	0.7719	1.04	0.5157	0.9889	1.04	0.4934	0.9712
Translationally-controlled tumor protein	TCTP_HUMAN	P13693	1.13	0.1371	0.4005	0.98	0.8496	0.9982	1.00	0.9700	0.9960	0.89	0.1505	0.9874	0.96	0.6408	0.9951	1.08	0.4338	0.9856	0.87	0.1118	0.6824	1.16	0.1100	0.3366	0.93	0.4161	0.9889	0.97	0.7349	0.9881
Translin	TSN_HUMAN	Q15631	1.17	0.1327	0.3961	0.99	0.9463	0.9982	0.85	0.1724	0.9960	1.12	0.2968	0.9939	1.02	0.8508	0.9951	1.28	0.0591	0.9641	0.91	0.4199	0.8021	1.46	0.0008	0.0763	1.11	0.3213	0.9889	1.16	0.1499	0.9607
Translocon-associated protein subunit delta	SSRD_HUMAN	P51571	0.85	0.1545	0.4236	1.07	0.5523	0.9982	1.02	0.9005	0.9960	1.02	0.8596	0.9939	1.01	0.9364	0.9951	0.95	0.6867	0.9856	1.10	0.4130	0.7921	0.72	0.0086	0.1364	0.88	0.2752	0.9889	0.95	0.6322	0.9881
Transmembrane emp24 domain-containing protein 1	TMED1_HUMAN	Q13445	0.84	0.0293	0.2873	0.99	0.9181	0.9982	1.05	0.6202	0.9960	0.92	0.2810	0.9939	0.85	0.0432	0.9951	0.90	0.3066	0.9856	0.98	0.8573	0.9623	1.03	0.7530	0.8646	0.97	0.7513	0.9889	0.91	0.2101	0.9607
Transmembrane emp24 domain-containing protein 10	TMEDA_HUMAN	P49755	0.80	0.0381	0.2873	1.08	0.4904	0.9982	1.03	0.8013	0.9960	1.02	0.8222	0.9939	0.92	0.4578	0.9951	0.92	0.5439	0.9856	1.14	0.2420	0.7187	0.76	0.0175	0.1636	0.98	0.8368	0.9889	0.93	0.5148	0.9846
Transmembrane emp24 domain-containing protein 2	TMED2_HUMAN	Q15363	0.93	0.4462	0.6912	1.09	0.3987	0.9982	1.01	0.9620	0.9960	1.03	0.7283	0.9939	0.95	0.5928	0.9951	0.94	0.6145	0.9856	1.11	0.3320	0.7438	0.74	0.0045	0.1246	0.86	0.1149	0.9889	1.01	0.9539	0.9938
Transmembrane emp24 domain-containing protein 9	TMED9_HUMAN	Q9BVK6	0.82	0.0194	0.2873	0.96	0.6837	0.9982	1.04	0.6920	0.9960	0.92	0.3441	0.9939	0.92	0.3767	0.9951	0.93	0.5269	0.9856	1.06	0.5073	0.8502	0.87	0.1456	0.3908	0.95	0.5701	0.9889	0.89	0.1802	0.9607
Transmembrane glycoprotein NMB	GPNMB_HUMAN	Q14956	1.13	0.2490	0.5210	0.88	0.2227	0.9982	1.05	0.6624	0.9960	0.85	0.1021	0.9874	1.03	0.7596	0.9951	0.84	0.1622	0.9856	0.99	0.9636	0.9924	1.04	0.7259	0.8460	0.95	0.5971	0.9889	0.92	0.4260	0.9607
Transmembrane protein 109	TM109_HUMAN	Q9BVC6	0.94	0.3732	0.6312	1.03	0.6287	0.9982	1.11	0.2008	0.9960	1.04	0.5481	0.9939	0.94	0.4086	0.9951	0.85	0.0461	0.9535	1.05	0.4862	0.8365	0.96	0.5817	0.7586	1.01	0.9349	0.9909	0.97	0.6672	0.9881
Transmembrane protein 120A	T120A_HUMAN	Q9BXJ8	1.01	0.8560	0.9307	0.93	0.3382	0.9982	0.99	0.8916	0.9960	0.96	0.5386	0.9939	1.04	0.6380	0.9951	1.06	0.5137	0.9856	0.95	0.5070	0.8502	1.17	0.0604	0.2529	1.14	0.0916	0.9889	1.16	0.0430	0.9607
Transmembrane protein 143	TM143_HUMAN	Q96AN5	1.18	0.0595	0.3008	1.18	0.0585	0.9982	1.01	0.9495	0.9960	1.01	0.8656	0.9939	1.10	0.3091	0.9951	0.93	0.4810	0.9856	1.16	0.1115	0.6824	0.87	0.1514	0.4008	1.07	0.4799	0.9889	1.18	0.0604	0.9607
Transmembrane protein 65	TMM65_HUMAN	Q6PI78	1.13	0.0816	0.3290	1.15	0.0412	0.9982	1.00	0.9632	0.9960	1.02	0.8159	0.9939	1.02	0.8254	0.9951	0.89	0.1769	0.9856	0.98	0.7993	0.9443	0.91	0.2423	0.5057	1.03	0.7086	0.9889	1.04	0.5383	0.9881
Transportin-1	TNPO1_HUMAN	Q92973	1.20	0.0662	0.3105	0.98	0.8115	0.9982	1.06	0.6159	0.9960	0.87	0.1711	0.9874	1.04	0.7202	0.9951	0.92	0.4947	0.9856	1.02	0.8216	0.9481	1.33	0.0096	0.1392	1.13	0.2290	0.9889	1.01	0.9430	0.9938
Transthyretin	TTHY_HUMAN	P02766	0.86	0.1786	0.4545	0.95	0.6366	0.9982	1.03	0.8136	0.9960	0.95	0.6729	0.9939	0.92	0.5017	0.9951	1.08	0.5902	0.9856	1.14	0.2856	0.7212	1.06	0.6375	0.7953	0.93	0.5377	0.9889	0.89	0.3023	0.9607
Trifunctional enzyme subunit alpha, mitochondrial	ECHA_HUMAN	P40939	1.12	0.0760	0.3266	1.12	0.0920	0.9982	0.99	0.8916	0.9960	1.15	0.0293	0.9874	1.09	0.2052	0.9951	0.99	0.9071	0.9959	1.01	0.9135	0.9848	1.08	0.3039	0.5503	1.05	0.4779	0.9889	1.07	0.3179	0.9607
Trifunctional enzyme subunit beta, mitochondrial	ECHB_HUMAN	P55084	1.10	0.1348	0.3975	1.11	0.1094	0.9982	1.01	0.9080	0.9960	1.17	0.0136	0.9874	1.10	0.1475	0.9951	0.96	0.6347	0.9856	1.04	0.5990	0.8646	1.05	0.5311	0.7227	1.03	0.6483	0.9889	1.05	0.4263	0.9607
Trimethyllysine dioxygenase, mitochondrial	TMLH_HUMAN	Q9NVH6	0.94	0.5373	0.7490	1.04	0.6844	0.9982	1.07	0.5339	0.9960	0.98	0.8278	0.9939	0.80	0.0277	0.9951	0.94	0.5933	0.9856	0.80	0.0293	0.6824	1.19	0.1190	0.3515	1.17	0.1205	0.9889	0.89	0.2196	0.9607
Triosephosphate isomerase	TPIS_HUMAN	P60174	1.16	0.1031	0.3639	0.96	0.6409	0.9982	0.94	0.5355	0.9960	0.87	0.1158	0.9874	1.02	0.8325	0.9951	1.18	0.1275	0.9856	0.92	0.3901	0.7838	1.12	0.2647	0.5268	0.93	0.4101	0.9889	1.04	0.6773	0.9881
Tripeptidyl-peptidase 1	TPP1_HUMAN	O14773	1.01	0.8317	0.9186	1.01	0.8583	0.9982	0.93	0.3309	0.9960	0.90	0.0989	0.9874	1.03	0.6197	0.9951	1.00	0.9899	0.9992	0.93	0.2619	0.7187	0.94	0.3868	0.6165	0.97	0.6167	0.9889	0.97	0.6639	0.9881
Tripeptidyl-peptidase 2	TPP2_HUMAN	P29144	0.98	0.8398	0.9208	0.99	0.9517	0.9982	1.40	0.0014	0.9960	0.99	0.8849	0.9939	0.97	0.7542	0.9951	0.70	0.0017	0.6489	1.19	0.0785	0.6824	1.00	0.9816	0.9891	0.88	0.2005	0.9889	0.97	0.7153	0.9881
Tropomodulin-1	TMOD1_HUMAN	P28289	0.90	0.1674	0.4377	0.98	0.7413	0.9982	0.98	0.7882	0.9960	0.99	0.8572	0.9939	0.96	0.6427	0.9951	0.95	0.5744	0.9856	0.99	0.8849	0.9751	1.02	0.8578	0.9230	1.09	0.2708	0.9889	1.05	0.5595	0.9881
Tropomodulin-3	TMOD3_HUMAN	Q9NYL9	0.89	0.1955	0.4726	1.04	0.6831	0.9982	1.00	0.9807	0.9960	0.97	0.7780	0.9939	0.98	0.8298	0.9951	0.98	0.8562	0.9959	1.12	0.2512	0.7187	0.74	0.0016	0.0774	1.04	0.7049	0.9889	0.91	0.2771	0.9607
Tropomyosin alpha-1 chain	TPM1_HUMAN	P09493	1.01	0.8313	0.9186	1.03	0.7208	0.9982	1.05	0.5450	0.9960	1.12	0.1109	0.9874	1.04	0.6337	0.9951	0.89	0.1632	0.9856	1.02	0.7523	0.9282	1.05	0.5075	0.7037	1.03	0.6897	0.9889	1.05	0.4648	0.9607
Tropomyosin alpha-3 chain	TPM3_HUMAN	P06753	0.96	0.7704	0.8895	0.85	0.2600	0.9982	1.03	0.8550	0.9960	1.17	0.2805	0.9939	1.03	0.8488	0.9951	0.73	0.0736	0.9641	0.99	0.9603	0.9924	0.88	0.4215	0.6447	0.82	0.1846	0.9889	0.92	0.5569	0.9881
Tropomyosin alpha-4 chain	TPM4_HUMAN	P67936	0.93	0.3802	0.6372	1.01	0.9020	0.9982	0.94	0.4547	0.9960	1.06	0.4475	0.9939	0.99	0.9090	0.9951	1.09	0.3510	0.9856	1.17	0.0509	0.6824	0.86	0.0864	0.2982	0.94	0.4420	0.9889	0.94	0.4000	0.9607

Tropomyosin beta chain	TPM2_HUMAN	P07951	1.00	0.9737	0.9855	0.98	0.7674	0.9982	0.92	0.3585	0.9960	1.04	0.6373	0.9939	0.99	0.9007	0.9951	1.02	0.8177	0.9959	1.00	0.9749	0.9939	1.15	0.1156	0.3460	1.05	0.5448	0.9889	1.06	0.4724	0.9607
Troponin C, slow skeletal and cardiac muscles	TNNC1_HUMAN	P63316	1.12	0.2545	0.5233	0.99	0.9389	0.9982	0.95	0.6395	0.9960	1.08	0.4578	0.9939	1.02	0.8683	0.9951	1.04	0.7789	0.9897	0.88	0.2338	0.7187	1.26	0.0381	0.2132	0.97	0.8055	0.9889	1.03	0.7464	0.9881
Troponin I, cardiac muscle	TNNI3_HUMAN	P19429	1.05	0.5182	0.7355	0.96	0.6276	0.9982	0.96	0.6140	0.9960	1.13	0.1055	0.9874	1.04	0.6119	0.9951	0.97	0.7666	0.9857	0.98	0.8264	0.9492	1.16	0.0806	0.2899	1.03	0.7311	0.9889	1.06	0.4263	0.9607
Troponin I, slow skeletal muscle	TNNI1_HUMAN	P19237	0.79	0.3275	0.5952	1.16	0.5400	0.9982	1.21	0.4920	0.9960	0.92	0.7401	0.9939	1.14	0.5892	0.9951	0.86	0.5976	0.9856	1.05	0.8606	0.9636	0.52	0.0123	0.1544	1.86	0.0095	0.9889	1.62	0.0390	0.9607
Troponin T, cardiac muscle	TNNT2_HUMAN	P45379	1.01	0.8507	0.9276	0.98	0.7639	0.9982	1.01	0.9390	0.9960	1.14	0.0746	0.9874	1.03	0.6854	0.9951	0.92	0.3328	0.9856	1.05	0.5365	0.8573	1.04	0.5933	0.7648	1.01	0.8625	0.9889	1.06	0.4227	0.9607
Tryptase beta-2	TRYB2_HUMAN	P20231	0.99	0.9040	0.9491	1.06	0.5693	0.9982	0.96	0.7404	0.9960	1.19	0.0941	0.9874	0.98	0.8556	0.9951	1.01	0.9292	0.9959	1.06	0.5801	0.8633	1.02	0.8534	0.9202	1.24	0.0422	0.9889	1.02	0.8121	0.9881
Tryptophan--tRNA ligase, cytoplasmic	SYWC_HUMAN	P23381	1.10	0.2379	0.5117	0.98	0.7712	0.9982	0.88	0.1623	0.9960	1.03	0.7006	0.9939	1.05	0.5564	0.9951	1.18	0.0798	0.9856	1.06	0.4765	0.8339	1.20	0.0344	0.2017	1.02	0.7993	0.9889	0.93	0.3621	0.9607
Tubulin alpha-1A chain	TBA1A_HUMAN	Q71U36	0.83	0.0145	0.2783	0.95	0.5480	0.9982	1.10	0.2999	0.9960	1.05	0.5534	0.9939	0.90	0.1970	0.9951	0.95	0.5735	0.9856	1.12	0.1672	0.6922	1.00	0.9569	0.9770	0.96	0.5885	0.9889	0.88	0.0908	0.9607
Tubulin alpha-4A chain	TBA4A_HUMAN	P68366	0.91	0.2153	0.4877	1.00	0.9543	0.9982	1.09	0.3341	0.9960	1.05	0.5063	0.9939	0.91	0.2439	0.9951	0.91	0.3200	0.9856	1.01	0.8781	0.9720	1.08	0.3915	0.6208	1.01	0.8958	0.9889	0.91	0.2176	0.9607
Tubulin alpha-8 chain	TBA8_HUMAN	Q9NY65	1.07	0.4238	0.6720	1.05	0.5410	0.9982	0.93	0.4708	0.9960	1.05	0.5514	0.9939	0.96	0.6234	0.9951	1.05	0.6228	0.9856	1.00	0.9576	0.9923	1.33	0.0024	0.0880	1.01	0.8791	0.9889	0.97	0.7646	0.9881
Tubulin beta chain	TBB5_HUMAN	P07437	0.79	0.0441	0.2873	0.90	0.3729	0.9982	1.00	0.9953	0.9979	1.12	0.3179	0.9939	0.99	0.9484	0.9951	1.00	0.9923	0.9992	1.38	0.0084	0.6779	0.85	0.2295	0.4943	0.95	0.6847	0.9889	0.90	0.3663	0.9607
Tubulin beta-2A chain	TBB2A_HUMAN	Q13885	0.88	0.1646	0.4371	0.97	0.7121	0.9982	1.03	0.7771	0.9960	1.12	0.1986	0.9939	1.04	0.7000	0.9951	0.99	0.9321	0.9959	1.14	0.1720	0.6993	0.94	0.5774	0.7542	0.93	0.4678	0.9889	0.94	0.5073	0.9846
Tubulin beta-4B chain	TBB4B_HUMAN	P68371	0.85	0.0381	0.2873	0.94	0.4553	0.9982	1.07	0.4745	0.9960	1.07	0.3954	0.9939	0.97	0.6775	0.9951	0.96	0.6504	0.9856	1.18	0.0478	0.6824	0.93	0.4415	0.6607	0.98	0.7654	0.9889	0.93	0.3344	0.9607
Tubulin-folding cofactor B	TBCB_HUMAN	Q99426	1.05	0.5256	0.7379	0.98	0.8032	0.9982	0.89	0.1683	0.9960	1.00	0.9785	0.9961	0.93	0.3574	0.9951	1.15	0.1160	0.9856	0.92	0.2532	0.7187	0.96	0.6451	0.7991	1.03	0.6544	0.9889	0.99	0.9256	0.9938
Tubulin-specific chaperone A	TBCA_HUMAN	O75347	1.02	0.8645	0.9348	0.96	0.7157	0.9982	0.91	0.4150	0.9960	0.94	0.5497	0.9939	0.93	0.4945	0.9951	1.20	0.1387	0.9856	0.85	0.1287	0.6868	1.22	0.0773	0.2846	0.89	0.2679	0.9889	0.88	0.1890	0.9607
Tumor protein D54	TPD54_HUMAN	O43399	0.98	0.6452	0.8286	0.92	0.1303	0.9982	0.99	0.8588	0.9960	1.11	0.0379	0.9874	1.05	0.3983	0.9951	0.96	0.5330	0.9856	1.10	0.0857	0.6824	1.04	0.5217	0.7170	1.00	0.9387	0.9922	1.02	0.7013	0.9881
Twinfilin-2	TWF2_HUMAN	Q6IBS0	0.89	0.0715	0.3224	1.04	0.5059	0.9982	1.04	0.5604	0.9960	0.94	0.3547	0.9939	0.89	0.0845	0.9951	1.01	0.8718	0.9959	0.99	0.9209	0.9848	1.13	0.0946	0.3121	0.96	0.5894	0.9889	0.89	0.0643	0.9607
Tyrosine-protein phosphatase non-receptor type 11	PTN11_HUMAN	Q06124	0.93	0.3793	0.6371	0.99	0.8880	0.9982	0.84	0.0878	0.9960	0.94	0.5014	0.9939	0.92	0.3492	0.9951	1.32	0.0075	0.9411	0.87	0.1137	0.6824	1.11	0.2957	0.5464	1.11	0.2445	0.9889	0.97	0.6845	0.9881
U1 small nuclear ribonucleoprotein A	SNRPA_HUMAN	P09012	0.93	0.1658	0.4371	1.00	0.9903	0.9985	1.06	0.3641	0.9960	0.94	0.2585	0.9939	0.90	0.0397	0.9951	0.96	0.5030	0.9856	0.94	0.2851	0.7212	0.97	0.5584	0.7400	0.99	0.8979	0.9889	0.98	0.6865	0.9881
Ubiquilin-1	UBQL1_HUMAN	Q9UMX0	1.05	0.4630	0.7039	1.07	0.3129	0.9982	1.03	0.6663	0.9960	1.07	0.2678	0.9939	1.07	0.3181	0.9951	0.97	0.7274	0.9857	1.04	0.5873	0.8645	0.93	0.3188	0.5613	0.97	0.5981	0.9889	0.98	0.7750	0.9881
Ubiquilin-2	UBQL2_HUMAN	Q9UHD9	1.10	0.2026	0.4813	1.04	0.5980	0.9982	1.00	0.9616	0.9960	0.92	0.2810	0.9939	0.96	0.6101	0.9951	1.06	0.5020	0.9856	0.97	0.6970	0.9074	1.02	0.8289	0.9088	0.97	0.6794	0.9889	0.94	0.3810	0.9607
Ubiquinol-cytochrome-c reductase complex assembly factor 2	UQCC2_HUMAN	Q9BRT2	0.98	0.7892	0.8986	0.99	0.8155	0.9982	1.01	0.8570	0.9960	1.01	0.9212	0.9939	0.94	0.3122	0.9951	1.00	0.9831	0.9992	0.97	0.6559	0.8915	1.07	0.3052	0.5503	1.17	0.0124	0.9889	0.96	0.4829	0.9652
Ubiquinone biosynthesis monooxygenase COQ6, mitochondrial	COQ6_HUMAN	Q9Y2Z9	1.13	0.0438	0.2873	1.10	0.1371	0.9982	0.97	0.7205	0.9960	1.05	0.3992	0.9939	1.10	0.1157	0.9951	0.99	0.9207	0.9959	1.11	0.1006	0.6824	0.97	0.6882	0.8252	1.05	0.4832	0.9889	1.10	0.1281	0.9607
Ubiquinone biosynthesis O-methyltransferase, mitochondrial	COQ3_HUMAN	Q9NZJ6	1.29	0.0063	0.2487	1.08	0.3928	0.9982	0.93	0.5349	0.9960	1.13	0.1867	0.9939	1.21	0.0539	0.9951	1.04	0.7515	0.9857	1.00	0.9713	0.9937	1.09	0.4190	0.6429	1.11	0.2926	0.9889	1.23	0.0281	0.9607
Ubiquinone biosynthesis protein COQ9, mitochondrial	COQ9_HUMAN	O75208	1.18	0.0562	0.2942	1.01	0.9409	0.9982	0.94	0.5334	0.9960	1.03	0.7737	0.9939	1.02	0.7982	0.9951	1.06	0.6103	0.9856	0.92	0.4018	0.7880	1.22	0.0465	0.2282	1.05	0.5867	0.9889	1.07	0.4726	0.9607
Ubiquitin carboxyl-terminal hydrolase 14	UBP14_HUMAN	P54578	1.01	0.8652	0.9348	1.05	0.4753	0.9982	1.08	0.3277	0.9960	0.98	0.8047	0.9939	0.89	0.1048	0.9951	0.91	0.2929	0.9856	0.90	0.1554	0.6871	1.04	0.6152	0.7826	1.01	0.8632	0.9889	0.94	0.3706	0.9607
Ubiquitin carboxyl-terminal hydrolase 5	UBP5_HUMAN	P45974	1.04	0.5467	0.7505	0.95	0.4138	0.9982	0.96	0.5553	0.9960	0.96	0.5296	0.9939	0.99	0.8733	0.9951	1.12	0.1108	0.9856	0.92	0.2009	0.7149	1.11	0.1260	0.3600	0.99	0.9005	0.9889	0.99	0.8921	0.9938
Ubiquitin carboxyl-terminal hydrolase isozyme L1	UCHL1_HUMAN	P09936	1.02	0.8318	0.9186	1.11	0.2650	0.9982	0.80	0.0436	0.9960	0.99	0.9249	0.9939	0.96	0.6593	0.9951	1.28	0.0369	0.9411	1.12	0.2718	0.7196	0.94	0.5454	0.7330	0.99	0.9103	0.9889	0.95	0.6274	0.9881
Ubiquitin recognition factor in ER-associated degradation protein 1	UFD1_HUMAN	Q92890	1.06	0.3784	0.6370	1.03	0.6756	0.9982	1.20	0.0101	0.9960	0.99	0.8405	0.9939	0.97	0.5923	0.9951	0.85	0.0299	0.9411	0.99	0.8279	0.9492	0.99	0.9018	0.9448	0.99	0.8437	0.9889	0.95	0.3899	0.9607
Ubiquitin thioesterase OTUB1	OTUB1_HUMAN	Q96FW1	1.00	0.9776	0.9871	0.92	0.3159	0.9982	1.05	0.6021	0.9960	0.98	0.7819	0.9939	0.99	0.8628	0.9951	1.06	0.5490	0.9856	0.91	0.2940	0.7277	1.31	0.0019	0.0815	0.94	0.4828	0.9889	0.95	0.4903	0.9712
Ubiquitin-40S ribosomal protein S27a	RS27A_HUMAN	P62979	0.91	0.0564	0.2942	0.99	0.8884	0.9982	0.99	0.8366	0.9960	1.00	0.9571	0.9939	0.96	0.4590	0.9951	1.03	0.5928	0.9856	1.01	0.9206	0.9848	0.89	0.0349	0.2017	0.95	0.3225	0.9889	0.93	0.1396	0.9607
Ubiquitin-associated protein 2-like	UBP2L_HUMAN	Q14157	1.07	0.3319	0.5968	0.99	0.8993	0.9982	0.93	0.3832	0.9960	1.03	0.7061	0.9939	1.05	0.5188	0.9951	1.07	0.4173	0.9856	0.93	0.3343	0.7458	1.09	0.2803	0.5337	0.92	0.2811	0.9889	1.01	0.9085	0.9938
Ubiquitin-conjugating enzyme E2 N	UBE2N_HUMAN	P61088	0.97	0.6768	0.8429	1.04	0.5199	0.9982	1.00	0.9727	0.9960	0.94	0.3090	0.9939	0.90	0.1352	0.9951	1.02	0.7677	0.9857	0.95	0.4350	0.8109	1.19	0.0166	0.1636	0.92	0.1937	0.9889	0.87	0.0310	0.9607
Ubiquitin-conjugating enzyme E2 variant 1	UB2V1_HUMAN	Q13404	1.13	0.2630	0.5333	1.03	0.7786	0.9982	0.90	0.4061	0.9960	0.85	0.1368	0.9874	0.97	0.7777	0.9951	1.13	0.3456	0.9856	0.84	0.1300	0.6868	1.13	0.3082	0.5505	0.87	0.2106	0.9889	0.97	0.7785	0.9881
Ubiquitin-like modifier-activating enzyme 1	UBA1_HUMAN	P22314	1.06	0.3718	0.6312	0.99	0.8337	0.9982	0.97	0.6934	0.9960	0.91	0.1359	0.9874	0.96	0.5770	0.9951	1.12	0.1559	0.9856	0.91	0.1698	0.6939	1.09	0.2344	0.4973	0.95	0.4053	0.9889	0.98	0.7856	0.9881
UDP-glucose:glycoprotein glucosyltransferase 1	UGGG1_HUMAN	Q9NYU2	0.99	0.8275	0.9186	1.04	0.4236	0.9982	1.10	0.1090	0.9960	1.00	0.9279	0.9939	0.97	0.5977	0.9951	0.92	0.1978	0.9856	0.98	0.7615	0.9282	1.03	0.6240	0.7880	0.98	0.7465	0.9889	0.95	0.2518	0.9607
UMP-CMP kinase	KCY_HUMAN	P30085	1.12	0.1127	0.3748	0.98	0.7486	0.9982	0.98	0.8122	0.9960	1.01	0.8974	0.9939	1.10	0.2154	0.9951	1.04	0.6372	0.9856	0.96	0.5645	0.8601	0.95	0.5691	0.7497	0.90	0.1689	0.9889	1.06	0.4003	0.9607
Unconventional myosin-Ic	MYO1C_HUMAN	O00159	0.99	0.9293	0.9592	0.99	0.8578	0.9982	1.01	0.8988	0.9960	0.98	0.7433	0.9939	1.01	0.8304	0.9951	0.97	0.6527	0.9856	1.04	0.5196	0.8558	1.01	0.9024	0.9449	0.96	0.4941	0.9889	1.03	0.6192	0.9881
UTP--glucose-1-phosphate uridylyltransferase	UGPA_HUMAN	Q16851	1.10	0.2494	0.5210	0.98	0.8388	0.9982	0.97	0.7747	0.9960	1.02	0.7598	0.9939	0.95	0.5191	0.9951	1.09	0.3964	0.9856	0.90	0.2040	0.7149	1.23	0.0172	0.1636	1.08	0.3628	0.9889	1.02	0.7959	0.9881
Utrophin	UTRO_HUMAN	P46939	0.93	0.1647	0.4371	0.97	0.5787	0.9982	1.01	0.8570	0.9960	0.95	0.3106	0.9939	0.91	0.0871	0.9951	1.04	0.5616	0.9856	1.03	0.5734	0.8630	1.04	0.4721	0.6780	0.98	0.6908	0.9889	1.01	0.8232	0.9881
UV excision repair protein RAD23 homolog B	RD23B_HUMAN	P54727	1.01	0.9429	0.9668	0.96	0.6072	0.9982	1.02	0.7832	0.9960	0.91	0.1750	0.9907	0.90	0.1452	0.9951	1.02	0.7912	0.9903	0.86	0.0517	0.6824	1.20	0.0218	0.1727	0.96	0.5390	0.9889	0.89	0.0940	0.9607
V-type proton ATPase catalytic subunit A	VATA_HUMAN	P38606	0.81	0.0042	0.2034	1.03	0.6604	0.9982	1.14	0.1199	0.9960	0.91	0.1998	0.9939	0.85	0.0292	0.9951	0.88	0.1766	0.9856	1.00	0.9950	0.9992	0.89	0.1640	0.4153	0.88	0.0950	0.9889	0.83	0.0101	0.9304
V-type proton ATPase subunit B, brain isoform	VATB2_HUMAN	P21281	0.87	0.1180	0.3810	1.04	0.6952	0.9982	1.02	0.8238	0.9960	1.05	0.5849	0.9939	0.97	0.6970	0.9951	0.98	0.8572	0.9959	1.09	0.3566	0.7644	0.83	0.0609	0.2543	0.97	0.7712	0.9889	0.89	0.1635	0.9607
V-type proton ATPase subunit d 1	VA0D1_HUMAN	P61421	1.01	0.9154	0.9536	1.04	0.6152	0.9982	1.00	0.9750	0.9960	0.97	0.7396	0.9939	0.95	0.5781	0.9951	1.00	0.9632	0.9967	1.08	0.4037	0.7880	0.93	0.4228	0.6454	0.89	0.1649	0.9889	0.94	0.4391	0.9607
V-type proton ATPase subunit E 1	VATE1_HUMAN	P36543	0.76	0.0003	0.1653	0.95	0.5482	0.9982	1.09	0.3405	0.9960	1.05	0.5249	0.9939	0.89	0.1666	0.9951	0.91	0.3508	0.9856	1.19	0.0389	0.6824	0.91	0.2744	0.5288	0.95	0.5092	0.9889	0.85	0.0312	0.9607
Vacuolar protein sorting-associated protein 29	VPS29_HUMAN	Q9UBQ0	1.15	0.0203	0.2873	1.03	0.6159	0.9982	0.98	0.7817	0.9960	0.92	0.1802	0.9939	1.02	0.7918	0.9951	1.02	0.8222	0.9959	0.93	0.2523	0.7187	1.03	0.7119	0.8360	0.89	0.0736	0.9889	1.02	0.7832	0.9881
Valacyclovir hydrolase	BPHL_HUMAN	Q86WA6	1.17	0.2082	0.4843	1.30	0.0368	0.9982	0.82	0.1602	0.9960	1.02	0.8861	0.9939	0.98	0.8790	0.9951	1.31	0.0811	0.9856	1.02	0.8763	0.9720	0.97	0.8338	0.9130	1.14	0.3021	0.9889	1.05	0.6835	0.9881
Valine--tRNA ligase	SYVC_HUMAN	P26640	1.05	0.4236	0.6720	0.99	0.8527	0.9982	1.07	0.3384	0.9960	1.08	0.2282	0.9939	1.01	0.8655	0.9951	0.95	0.5322	0.9856	1.07	0.3505	0.7593	1.05	0.4929	0.6932	0.95	0.4484	0.9889	0.99	0.8279	0.9881
Vasodilator-stimulated phosphoprotein	VASP_HUMAN	P50552	0.95	0.3742	0.6312	0.96	0.4488	0.9982	1.01	0.8344	0.9960	1.13	0.0304	0.9874	1.05	0.4400	0.9951	1.00	0.9995	0.9995	1.11	0.1114	0.6824	0.97	0.6143	0.7821	1.01	0.8729	0.9889	0.98	0.7232	0.9881
Versican core protein	CSPG2_HUMAN	P13611	0.78	0.1336	0.3966	0.94	0.7185	0.9982	0.94	0.7631	0.9960	0.91	0.5445	0.9939	0.78	0.1441	0.9951	1.23	0.3069	0.9856	1.06	0.7227	0.9201	0.79	0.2024	0.4634	1.12	0.5169	0.9889	0.97	0.8494	0.9881
Very long-chain specific acyl-CoA dehydrogenase, mitochondrial	ACADV_HUMAN	P49748	1.14	0.0976	0.3563	1.03	0.7067	0.9982	1.02	0.8044	0.9960	1.11	0.2017	0.9939	1.17	0.0527	0.9951	1.00	0.9691	0.9967	1.09	0.3089	0.7378	1.09	0.3468	0.5798	1.05	0.5889	0.9889	1.09	0.2740	0.9607
Very-long-chain 3-oxoacyl-CoA reductase	DHB12_HUMAN	Q53GQ0	0.88	0.1123	0.3748	1.02	0.8202	0.9982	1.14	0.1612	0.9960	1.09	0.3168	0.9939	1.04	0.6379	0.9951	0.81	0.0401	0.9411	1.06	0.5416	0.8573	0.82	0.0319	0.2001	0.91	0.2611	0.9889	0.99	0.8628	0.9881
Vesicle-associated membrane protein-associated protein A	VAPA_HUMAN	Q9P0L0	0.88	0.0218	0.2873	0.96	0.4159	0.9982	1.00	0.9497	0.9960	1.01	0.8151	0.9939	0.98	0.7136	0.9951	0.99	0.8627	0.9959	0.99	0.8797	0.9722	0.92	0.2040	0.4651	0.99	0.8063	0.9889	0.98	0.7431	0.9881
Vesicle-associated membrane protein-associated protein B/C	VAPB_HUMAN	O95292	0.90	0.0247	0.2873	0.96	0.4165	0.9982	0.99	0.8453	0.9960	1.02	0.7016	0.9939	0.99	0.7843	0.9951	1.00	0.9498	0.9967	0.95	0.3674	0.7673	1.02	0.7734	0.8789	1.00	0.9887	0.9976	1.00	0.9257	0.9938
Vesicle-trafficking protein SEC22b	SC22B_HUMAN	O75396	0.89	0.0557	0.2942	0.98	0.7356	0.9982	0.99	0.9421	0.9960	1.05	0.4227	0.9939	0.96	0.5739	0.9951	0.95	0.4805	0.9856	1.11	0.1374	0.6871	0.93	0.3446	0.5782	0.95	0.4583	0.9889	0.91	0.1226	0.9607
Vesicular integral-membrane protein VIP36	LMAN2_HUMAN	Q12907	0.97	0.8062	0.9060	1.08	0.5458	0.9982	1.23	0.1352	0.9960	0.97	0.8037	0.9939	1.02	0.9035	0.9951	0.75	0.0511	0.9621	1.33	0.0255	0.6824	0.79	0.0751	0.2824	0.83	0.1261	0.9889	0.93	0.5231	0.9846
Vimentin	VIME_HUMAN	P08670	0.81	0.0356	0.2873	0.99	0.9081	0.9982	0.96	0.7105	0.9960	0.93	0.4864	0.9939	0.89	0.2807	0.9951	1.07	0.5932	0.9856	1.12	0.2982	0.7319	0.76	0.0145	0.1589	0.99	0.9041	0.9889	0.99	0.9174	0.9938
Vinculin	VINC_HUMAN	P18206	0.99	0.8447	0.9230	0.97	0.5394	0.9982	1.06	0.3037	0.9960	0.96	0.4338	0.9939	0.94	0.2167	0.9951	0.91	0.1162	0.9856	0.87	0.0065	0.6779	1.06	0.2947	0.5464	0.99	0.8525	0.9889	0.97	0.6002	0.9881
Vitamin D-binding protein	VTDB_HUMAN	P02774	0.83	0.1075	0.3670	1.03	0.7747	0.9982	1.08	0.5519	0.9960	1.04	0.7200	0.9939	1.01	0.9642	0.9951	0.95	0.7313	0.9857	1.12	0.3590	0.7644	0.81	0.0847	0.2956	0.94	0.6107	0.9889	0.91	0.3850	0.9607
Vitamin K-dependent protein S	PROS_HUMAN	P07225	0.84	0.0806	0.3290	0.85	0.1113	0.9982	1.19	0.1200	0.9960	1.21	0.0482	0.9874	1.12	0.2717	0.9951	0.77	0.0334	0.9411	1.05	0.6228	0.8807	0.93	0.4971	0.6967	0.91	0.3644	0.9889	1.03	0.7930	0.9881
Vitronectin	VTNC_HUMAN	P04004	0.79	0.1770	0.4521	1.27	0.1719	0.9982	1.14	0.5095	0.9960	0.91	0.5910	0.9939	0.91	0.6061	0.9951	0.81	0.3190	0.9856	1.25	0.2287	0.7187	0.52	0.0005	0.0655	1.10	0.5829	0.9889	0.89	0.5037	0.9818
Voltage-dependent anion-selective channel protein 1	VDAC1_HUMAN	P21796	1.13	0.2581	0.5281	1.08	0.4901	0.9982	1.15	0.2556	0.9960	0.96	0.6704	0.9939	1.09	0.4428	0.9951	0.80	0.0887	0.9856	0.83	0.1109	0.6824	1.00	0.9969	0.9975	0.95	0.6560	0.9889	1.08	0.4548	0.9607
Voltage-dependent anion-selective channel protein 2	VDAC2_HUMAN	P45880	1.07	0.4306	0.6786	1.07	0.4106	0.9982	1.04	0.6943	0.9960	0.95	0.5027	0.9939	1.02	0.8579	0.9951	0.93	0.4875	0.9856	0.92	0.3293	0.7411	0.93	0.4318	0.6515	0.98	0.8172	0.9889	1.08	0.3420	0.9607
Voltage-dependent anion-selective channel protein 3	VDAC3_HUMAN	Q9Y277	1.05	0.5937	0.7894	1.07	0.5188	0.9982	1.05	0.6852	0.9960	1.00	0.9758	0.9961	1.03	0.7581	0.9951	0.94	0.6353	0.9856	0.87	0.1873	0.7149	1.01	0.9171	0.9558	1.04	0.6660	0.9889	1.09	0.3671	0.9607
Voltage-dependent calcium channel subunit alpha-2/delta-1	CA2D1_HUMAN	P54289	0.98	0.7327	0.8760	0.97	0.5610	0.9982	1.01	0.8276	0.9960	1.06	0.3161	0.9939	1.00	0.9555	0.9951	0.94	0.4081	0.9856	0.96	0.4856	0.8365	0.95	0.3945	0.6230	1.02	0.7620	0.9889	1.00	0.9379	0.9938
von Willebrand factor	VWF_HUMAN	P04275	1.01	0.8780	0.9384	1.17	0.0671	0.9982	0.98	0.8729	0.9960	1.04	0.6283	0.9939	1.07	0.4564	0.9951	1.03	0.7701	0.9857	1.08	0.3774	0.7741	0.69	<0.0001	0.0511	1.00	0.9654	0.9961	1.05	0.5590	0.9881
WD repeat-containing protein 1	WDR1_HUMAN	O75083	1.07	0.2833	0.5570	0.97	0.6731	0.9982	1.05	0.5166	0.9960	0.89	0.0928	0.9874	0.94	0.3358	0.9951	1.01	0.9058	0.9959	0.84	0.0127	0.6779	1.19	0.0183	0.1650	0.92	0.2325	0.9889	0.95	0.4277	0.9607
WD repeat-containing protein 61	WDR61_HUMAN	Q9GZS3	1.09	0.1337	0.3966	1.01	0.8610	0.9982	1.12	0.1013	0.9960	1.01	0.8562	0.9939	1.01	0.8368	0.9951	1.00	0.9852	0.9992	0.92	0.1739	0.7016	1.09	0.1889	0.4471	1.03	0.6416	0.9889	1.08	0.1980	0.9607
Wiskott-Aldrich syndrome protein family member 2	WASF2_HUMAN	Q9Y6W5	0.97	0.6551	0.8297	0.97	0.6432	0.9982	1.06	0.4910	0.9960	0.93	0.3219	0.9939	1.00	0.9615	0.9951	0.96	0.6497	0.9856	1.06	0.4068	0.7885	0.89	0.1398	0.3825	1.01	0.8465	0.9889	0.92	0.2497	0.9607
Wolframin	WFS1_HUMAN	O76024	1.11	0.2923	0.5638	1.25	0.0242	0.9982	1.06	0.6244	0.9960	0.96	0.6649	0.9939	1.09	0.4344	0.9951	0.92	0.4879	0.9856	1.03	0.8145	0.9478	0.83	0.0858	0.2982	1.15	0.1685	0.9889	1.26	0.0178	0.9607
X-ray repair cross-complementing protein 5	XRCC5_HUMAN	P13010	1.00	0.9840	0.9915	1.08	0.1346	0.9982	0.98	0.7528	0.9960	0.98	0.6234	0.9939	0.92	0.1060	0.9951	1.05	0.4597	0.9856	0.94	0.2288	0.7187	1.00	0.9903	0.9947	0.99	0.9038	0.9889	0.94	0.2432	0.9607
X-ray repair cross-complementing protein 6	XRCC6_HUMAN	P12956	0.96	0.4236	0.6720	1.04	0.3846	0.9982	0.99	0.9189	0.9960	1.00	0.9586	0.9942	0.91	0.0623	0.9951	1.06	0.3191	0.9856	1.02	0.7175	0.9164	1.02	0.7556	0.8663	0.97	0.6010	0.9889	0.92	0.0863	0.9607
Xaa-Pro aminopeptidase 1	XPP1_HUMAN	Q9NQW7	1.12	0.2054	0.4823	1.03	0.7508	0.9982	1.07	0.4855	0.9960	0.88	0.1535	0.9874	0.96	0.6507	0.9951	1.01	0.9364	0.9959	0.95	0.5987	0.8646	1.09	0.3656	0.5972	0.91	0.3062	0.9889	0.89	0.1991	0.9607
Xaa-Pro aminopeptidase 3	XPP3_HUMAN	Q9NQH7	1.15	0.0551	0.2942	0.92	0.2657	0.9982	1.07	0.4331	0.9960	1.03	0.6521	0.9939	1.08	0.3073	0.9951	0.93	0.4144	0.9856	0.96	0.5679	0.8601	1.04	0.6214	0.7872	1.09	0.2708	0.9889	1.09	0.2531	0.9607
Xin actin-binding repeat-containing protein 1	XIRP1_HUMAN	Q702N8	0.93	0.3731	0.6312	0.92	0.2705	0.9982	1.03	0.7821	0.9960	1.10	0.2299	0.9939	0.92	0.2672	0.9951	0.91	0.3388	0.9856	0.98	0.7663	0.9291	1.09	0.3293	0.5682	1.01	0.9261	0.9889	0.95	0.5117	0.9846
Xin actin-binding repeat-containing protein 2	XIRP2_HUMAN	A4UGR9	1.10	0.3441	0.6081	1.02	0.8239	0.9982	0.93	0.5136	0.9960	1.01	0.9390	0.9939	1.01	0.9594	0.9951	1.02	0.8441	0.9959	0.93	0.4596	0.8243	1.04	0.7020	0.8318	1.21	0.0557	0.9889	1.06	0.5519	0.9881
Zinc-alpha-2-glycoprotein	ZA2G_HUMAN	P25311	0.93	0.3846	0.6432	1.01	0.8836	0.9982	1.09	0.3848	0.9960	1.13	0.1528	0.9874	1.10	0.2895	0.9951	0.93	0.5189	0.9856	1.15	0.1373	0.6871	0.89	0.2295	0.4943	0.99	0.8802	0.9889	0.98	0.7786	0.9881
Zyxin	ZYX_HUMAN	Q15942	1.04	0.5407	0.7503	1.06	0.3870	0.9982	0.94	0.4074	0.9960	1.00	0.9989	0.9995	1.02	0.7374	0.9951	0.96	0.5837	0.9856	0.99	0.8326	0.9513	0.88	0.0975	0.3162	1.06	0.3843	0.9889	0.99	0.8812	0.9938

Supplementary Table VIII. Term enrichment for proteins significantly altered in the SDS extracts of heart failure patients according to medication usage.

Medication	Category	Term ID	Term description	Count	Genes	List Total	Pop Hits	Pop Total	P-value	Benjamini	Fold Enrichment
Beta-blockers (n=328)	GO_CC	GO:0070062	Extracellular exosome	189	RPL5, PDCD5, PEBP1, APEH, ENO3, ACTB, C4B, NPEPPS, LGALS3, PCMT1, CAPNS1, LAMP1, PPP2R1A, FAM162A, CFL2, PSMD2, RUVBL2, KPNA4, CAPN1, PRKACA, B2M, GYG1, RPS13, ENTPD1, ACTN2, RPS5, CMBL, HLA-A, ATP1B1, MTAP, SERPING1, S100A4, PHPT1, CFB, KPNB1, PFN2, TMED10, CFH, STXBP3, AK1, LTBP4, LPL, FBLN1, LTBP2, A1BG, TXN, ATP5PD, FBLN5, ADH5, KNG1, ACAT1, RAP1B, C3, VTN, PRDX2, NCSTN, LDHA, PRDX4, PODXL, C9, ESD, H3C1, ISOC1, IGFBP7, MAP4, APOE, A2M, DECR1, HSPA8, GSTM2, HSPA4, FN1, GNG12, CP, IGF2R, CCT6A, EHD1, H4C1, MACROH2A2, NACA, ARPC3, GNB2, OGN, GNB1, UBE2N, ESAM, TGFBI, ITGB1, YWHAE, SERPINA3, COL18A1, PYGB, ORM1, SERPINA1, WDR1, GBE1, CLTC, HBB, AKR1B1, DBI, PYGM, H2AC20, HEBP2, HINT1, LASP1, SEPTIN7, ACP2, ITGAV, SH3BGRL3, ACADM, TNPO1, YWHAG, PGM1, CTSB, EIF5A, FGB, CCT2, FGA, WARS1, ANXA1, VWF, MACROH2A1, SSR4, H2AZ1, GSTO1, ANXA4, ANXA5, PGAM2, H2AX, FGG, BGN, NUTF2, F2, SEPTIN2, CKAP4, PSMA4, CTTN, PSMA2, COL6A2, COL6A1, COL6A3, PFDN2, MFGE8, CD44, OTUB1, ITIH4, GRN, ASAH1, ITIH2, AHCY, AHNAK, SERPINC1, SRSF1, NIBAN1, PRELP, GLRX, PLG, ATP1A1, GNAI2, PPP2CA, UGP2, PSMB3, DDT, GNA11, ELOB, STOM, BPNT1, RPS2, CCT8, CCT7, RPS27A, CCT5, CCT4, SEPTIN11, MDH1, LUM, TXNL1, APOC3, PLXDC2, RAB11B, PPP1CA, FABP3, LRG1, DES, APOC2, CAPZA2, APOC1, SAA1, VIM	311	1241	2818	3.59E-10	1.45E-07	1.38
Beta-blockers (n=328)	GO_CC	GO:0005615	**Extracellular space**	66	SERPINA3, COL18A1, ORM1, SERPINA1, TNC, AKR1B1, ENO3, ACTB, C4B, LGALS3, CFL2, B2M, CTSB, FGB, FGA, POSTN, ANXA1, PTGIS, FGG, F2, DCN, COL6A2, COL6A3, SERPING1, S100A4, MFGE8, CFB, ENG, ASAH1, GRN, CFH, SERPINC1, LTBP4, LPL, FBLN1, PRELP, LTBP2, PLG, A1BG, FBLN5, KNG1, C3, VTN, PRDX4, PODXL, NPY, STOM, IGFBP7, APOE, RPS27A, TIMM8B, HSPA8, MDH1, LUM, FN1, APOC3, CP, IGF2R, FABP3, LRG1, APOC2, GNB2, OGN, SAA1, TGFBI, FMOD	311	324	2818	2.25E-07	2.27E-05	1.85
Beta-blockers (n=328)	GO_CC	GO:0005576	**Extracellular region**	59	SERPINA3, COL18A1, ORM1, SERPINA1, WDR1, TNC, HBB, F13A1, C4B, B2M, CTSB, FGB, FGA, ANXA1, VWF, ACTN2, FGG, BGN, F2, DCN, COL6A2, COL6A1, COL6A3, SERPING1, MFGE8, CFB, ITIH4, ITIH2, CFH, SERPINC1, LTBP4, LPL, FBLN1, PRELP, PLG, A1BG, FBLN5, KNG1, C3, VTN, C9, NPY, H3C1, IGFBP7, APOE, A2M, LUM, FN1, APOC3, CP, H4C1, LRG1, APOC2, CAPZA2, APOC1, OGN, SAA1, TGFBI, FMOD	311	260	2818	2.26E-08	3.03E-06	2.06
Beta-blockers (n=328)	GO_CC	GO:0031012	**Extracellular matrix**	37	COL18A1, CLTC, LTBP4, TNC, LPL, FBLN1, LTBP2, PRELP, FBLN5, VTN, LGALS3, LMNA, IGFBP7, APOE, RPS13, CCT2, HSPA8, POSTN, VWF, LUM, RPS5, FN1, BGN, CKAP4, DCN, ASPN, CCT6A, H4C1, COL6A2, COL6A1, OGN, COL6A3, TGFBI, VIM, MFGE8, FMOD, NES	311	171	2818	5.69E-05	2.86E-03	1.96
Beta-blockers (n=328)	GO_CC	GO:0072562	**Blood microparticle**	29	SERPINA3, ITIH4, ORM1, ITIH2, CFH, SERPINC1, HBB, F13A1, PLG, A1BG, HBD, KNG1, ACTB, C3, C4B, VTN, C9, STOM, APOE, A2M, FGB, FGA, HSPA8, FGG, FN1, F2, CP, SERPING1, CFB	311	75	2818	1.16E-09	2.33E-07	3.50
Beta-blockers (n=328)	GO_BP	GO:0030198	**Extracellular matrix organization**	24	FGB, ITGB1, FGA, COL18A1, POSTN, VWF, LUM, FGG, TNC, BGN, FN1, FBLN1, HSD17B12, DCN, FBLN5, VTN, PRDX4, COL6A2, COL6A1, COL6A3, ITGA7, ITGAV, TGFBI, CD44	307	76	2721	4.27E-06	6.24E-03	2.80
Beta-blockers (n=328)	GO_CC	GO:0005578	**Proteinaceous extracellular matrix**	19	COL18A1, POSTN, SERPINA1, VWF, LUM, LTBP4, BGN, FN1, PRELP, FBLN1, LTBP2, HSD17B12, ASPN, FBLN5, COL6A2, OGN, COL6A3, TGFBI, FMOD	311	55	2818	1.09E-05	7.33E-04	3.13
Beta-blockers (n=328)	GO_BP	GO:0007596	**Blood coagulation**	18	FGB, FGA, ENTPD1, SERPINA1, VWF, SERPINC1, ANXA5, FGG, HBB, F13A1, PLG, HBD, F2, EHD1, PRKAR1A, CAPZA2, H3C1, PRKACA	307	48	2721	7.73E-06	6.24E-03	3.32
Beta-blockers (n=328)	KEGG	hsa04610	**Complement and coagulation cascades**	17	FGB, FGA, SERPINA1, CFH, VWF, SERPINC1, FGG, F13A1, PLG, F2, KNG1, C3, C4B, C9	175	37	1527	6.25E-07	1.22E-04	4.01
Beta-blockers (n=328)	REACTOME	R-HSA-216083	**Integrin cell surface interactions**	16	FGB, ITGB1, FGA, COL18A1, VWF, LUM, FGG, TNC, FN1, VTN, COL6A2, COL6A1, COL6	234	45	1877	1.81E-04	4.65E-02	2.85
Beta-blockers (n=328)	GO_CC	GO:0031093	**Platelet alpha granule lumen**	15	FGB, FGA, SERPINA3, ORM1, SERPINA1, VWF, ACTN2, FGG, FN1, F13A1, PLG, A1BG, K	311	32	2818	2.13E-06	1.72E-04	4.25
Beta-blockers (n=328)	GO_CC	GO:1903561	**extracellular vesicle**	14	FGB, FGA, CLTC, PRELP, ATP1A1, ATP1B1, GNAI2, PCMT1, COL6A2, OGN, GNB1, COL	311	32	2818	1.35E-05	7.74E-04	3.96
Beta-blockers (n=328)	REACTOME	R-HSA-3000178	**ECM proteoglycans**	14	ITGB1, LUM, TNC, BGN, FN1, DCN, ASPN, VTN, COL6A2, COL6A1, COL6A3, ITGA7, ITG	234	37	1877	2.73E-04	4.65E-02	3.04
Beta-blockers (n=328)	KEGG	hsa05034	**Alcoholism**	13	MACROH2A1, H2AZ1, H2AX, GNG12, H2AC20, GNAI2, PPP1CA, H4C1, MACROH2A2, NP	175	35	1527	2.60E-04	2.55E-02	3.24
Beta-blockers (n=328)	GO_CC	GO:0000786	Nucleosome	8	H4C1, H1-0, MACROH2A2, MACROH2A1, H2AZ1, H2AX, H3C1, H2AC20	311	15	2818	5.37E-04	2.40E-02	4.83
Beta-blockers (n=328)	REACTOME	R-HSA-390450	Folding of actin by CCT/TriC	7	CCT6A, CCT2, CCT8, CCT7, CCT5, ACTB, CCT4	234	9	1877	2.09E-04	4.65E-02	6.24
Beta-blockers (n=328)	GO_CC	GO:0005832	Chaperonin-containing T-complex	6	CCT6A, CCT2, CCT8, CCT7, CCT5, CCT4	311	8	2818	6.60E-04	2.66E-02	6.80
Statins (n=267)	GO_CC	GO:0005739	Mitochondrion	94	MIPEP, ISCA2, NDUFA13, ACAA2, HIBADH, ECI1, ETFA, ETFB, HK1, GCSH, FUNDC2, HINT2, ABHD11, NUDT19, LONP1, MACROD1, IDH3B, UQCRFS1, MLYCD, ATP6V1E1, HADH, YWHAH, ACAD8, BCKDHA, MCCC2, ATPAF2, HSDL2, LYPLA1, MRPS27, MRPS23, ECH1, SIRT5, REXO2, SDHA, HDHD3, SND1, COX6B1, PYURF, SARS2, ALDH5A1, KARS1, FDX1, UQCRC1, SUCLG1, L2HGDH, SLC25A12, ALDH7A1, BCAT2, ATP6V1A, GCDH, NDUFB4, TIMM10, NOL3, COX5B, FHIT, NAPG, CKMT2, ATP5F1B, GRPEL1, PITRM1, HDHD5, CKB, RDH13, DECR1, MPST, BCAP31, NDUFA9, TIMM8A, TRAP1, FDPS, GATD3A, DUT, PDHA1, SPRYD4, PTGES2, CCDC58, ADHFE1, IDH2, GSR, SUOX, HARS2, COQ7, LRPPRC, COQ6, NUDT9, QDPR, MSRA, COQ3, OGDH, ACO1, ACO2, SSBP1, SLIRP, CRAT	260	625	2818	8.12E-08	1.38E-05	1.63

Statins (n=267)	KEGG	hsa01100	**Metabolic pathways**	61	NDUFA13, ACAA2, HIBADH, PYGM, ENO1, HK1, GCSH, MCEE, SPR, ALDH2, IDH3B, UQCRFS1, MLYCD, ATP6V1E1, HADH, PTGDS, ACAD8, BCKDHA, MCCC2, CBR1, DCXR, SDHA, COX6B1, MTAP, ALDH5A1, NDUFS7, UQCRC1, SUCLG1, ALDH7A1, CES1, BCAT2, ATP6V1A, GCDH, RPN2, NDUFB4, AK2, COX5B, COX5A, CKMT2, ATP5B, BPNT1, CKB, ACSS1, MPST, NDUFA9, GGT5, FDPS, DUT, PDHA1, CKM, PTGES2, AGL, IDH2, COQ7, COQ6, QDPR, GRHPR, COQ3, OGDH, ACO1, ACO2	155	383	1527	4.15E-05	7.76E-03	1.57
Statins (n=267)	GO_CC	GO:0005759	**Mitochondrial matrix**	50	MIPEP, ISCA2, HIBADH, ECI1, ETFA, ETFB, GCSH, MCEE, ALDH2, LONP1, IDH3B, MLYCD, HADH, ACAD8, BCKDHA, MCCC2, SIRT5, REXO2, SARS2, ALDH5A1, NDUFS7, KARS1, FDX1, PPIF, SUCLG1, HAGH, ALDH7A1, BCAT2, GCDH, IBA57, ATP5B, PDPR, GRPEL1, PITRM1, ACSS1, DECR1, NDUFA9, TRAP1, PDHA1, ADHFE1, IDH2, GSR, SUOX, HARS2, NUDT9, COQ3, LYRM7, OGDH, ACO2, SSBP1	260	208	2818	8.56E-11	2.92E-08	2.61
Statins (n=267)	REACTOME	R-HSA-977225	Amyloid fiber formation	9	CST3, H4C1, GSN, H2AZ1, H2BC10, H2AX, H3C1, H2AC20, H2AC4	192	25	1877	2.34E-03	4.12E-02	3.52
Statins (n=267)	GO_CC	GO:0000786	nucleosome	8	H4C1, MACROH2A2, H2AZ1, H2BC10, H2AX, H3C1, H2AC20, H2AC4	260	15	2818	1.73E-04	1.96E-02	5.78
Statins (n=267)	REACTOME	R-HSA-5250924	B-WICH complex positively regulates rRNA expression	8	H4C1, H2AZ1, H2BC10, H2AX, H3C1, ACTB, H2AC20, H2AC4	192	12	1877	5.17E-05	2.23E-03	6.52
Statins (n=267)	REACTOME	R-HSA-1221632	Meiotic synapsis	8	H4C1, H2AZ1, H2BC10, LMNA, H2AX, LMNB1, H2AC20, H2AC4	192	15	1877	3.21E-04	7.40E-03	5.21
Statins (n=267)	REACTOME	R-HSA-5334118	DNA methylation	7	H4C1, H2AZ1, H2BC10, H2AX, H3C1, H2AC20, H2AC4	192	7	1877	6.62E-06	8.15E-04	9.78
Statins (n=267)	REACTOME	R-HSA-427359	SIRT1 negatively regulates rRNA expression	7	H4C1, H2AZ1, H2BC10, H2AX, H3C1, H2AC20, H2AC4	192	7	1877	6.62E-06	8.15E-04	9.78
Statins (n=267)	REACTOME	R-HSA-5625886	Activated PKN1 stimulates transcription of AR (androgen receptor) regulated genes KLK2 and KLK3	7	H4C1, H2AZ1, H2BC10, H2AX, H3C1, H2AC20, H2AC4	192	7	1877	6.62E-06	8.15E-04	9.78
Statins (n=267)	REACTOME	R-HSA-73728	RNA Polymerase I Promoter Opening	7	H4C1, H2AZ1, H2BC10, H2AX, H3C1, H2AC20, H2AC4	192	8	1877	2.43E-05	1.49E-03	8.55
Statins (n=267)	REACTOME	R-HSA-2299718	Condensation of Prophase Chromosomes	7	H4C1, H2AZ1, H2BC10, H2AX, H3C1, H2AC20, H2AC4	192	8	1877	2.43E-05	1.49E-03	8.55
Statins (n=267)	REACTOME	R-HSA-427413	NoRC negatively regulates rRNA expression	7	H4C1, H2AZ1, H2BC10, H2AX, H3C1, H2AC20, H2AC4	192	8	1877	2.43E-05	1.49E-03	8.55
Statins (n=267)	REACTOME	R-HSA-201722	Formation of the beta-catenin:TCF transactivating complex	7	H4C1, H2AZ1, H2BC10, H2AX, H3C1, H2AC20, H2AC4	192	9	1877	6.66E-05	2.23E-03	7.60
Statins (n=267)	REACTOME	R-HSA-73777	RNA Polymerase I Chain Elongation	7	H4C1, H2AZ1, H2BC10, H2AX, H3C1, H2AC20, H2AC4	192	9	1877	6.66E-05	2.23E-03	7.60
Statins (n=267)	REACTOME	R-HSA-212300	PRC2 methylates histones and DNA	7	H4C1, H2AZ1, H2BC10, H2AX, H3C1, H2AC20, H2AC4	192	9	1877	6.66E-05	2.23E-03	7.60
Statins (n=267)	REACTOME	R-HSA-3214847	HATs acetylate histones	7	H4C1, H2BC10, ACTL6A, H3C1, ACTB, H2AC20, H2AC4	192	10	1877	1.52E-04	4.69E-03	6.84
Statins (n=267)	REACTOME	R-HSA-5617472	Activation of anterior HOX genes in hindbrain development during early embryogenesis	7	H4C1, H2AZ1, H2BC10, H2AX, H3C1, H2AC20, H2AC4	192	11	1877	3.07E-04	7.40E-03	6.22
Statins (n=267)	REACTOME	R-HSA-912446	Meiotic recombination	7	H4C1, H2AZ1, H2BC10, H2AX, H3C1, H2AC20, H2AC4	192	11	1877	3.07E-04	7.40E-03	6.22
Statins (n=267)	REACTOME	R-HSA-2559582	Senescence-Associated Secretory Phenotype (SASP)	7	H4C1, H2AZ1, H2BC10, H2AX, H3C1, H2AC20, H2AC4	192	12	1877	5.63E-04	1.22E-02	5.70
Statins (n=267)	REACTOME	R-HSA-3214858	RMTs methylate histone arginines	7	H4C1, H2AZ1, ACTL6A, H2AX, H3C1, H2AC20, H2AC4	192	14	1877	1.53E-03	3.15E-02	4.89
Statins (n=267)	REACTOME	R-HSA-2559580	Oxidative Stress Induced Senescence	7	H4C1, H2AZ1, H2BC10, H2AX, H3C1, H2AC20, H2AC4	192	15	1877	2.34E-03	4.12E-02	4.56
Statins (n=267)	REACTOME	R-HSA-171306	Packaging Of Telomere Ends	6	H4C1, H2AZ1, H2BC10, H2AX, H2AC20, H2AC4	192	6	1877	5.73E-05	2.23E-03	9.78
Statins (n=267)	REACTOME	R-HSA-2559586	DNA Damage/Telomere Stress Induced Senescence	6	H4C1, H2AZ1, H2BC10, H2AX, H2AC20, H2AC4	192	7	1877	1.84E-04	5.22E-03	8.38
Statins (n=267)	REACTOME	R-HSA-606279	Deposition of new CENPA-containing nucleosomes at the centromere	6	H4C1, H2AZ1, H2BC10, H2AX, H2AC20, H2AC4	192	10	1877	1.71E-03	3.31E-02	5.87

Terms highlighted in bold were included in Figure 6B. Please note that REACTOME terms found significant for the use of statins are highly overlappig, often consisting in the same proteins.

Supplemental Table IX. Clinical characteristics of ischemic HF patients based on β-blocker usage.

	No β-blockers	β-blockers	P-value
**General**			
n	21	44	
M:F	14:7	41:3	0.010
Age	58.4±5.4	57.4±6.7	0.55
BMI	26.6±3.8	27.1±3.4	0.78
**Co-morbidities (n [%])**			
Diabetes	14 [66.7%]	13 [29.5%]	0.007
Hypertension	14 [66.7%]	28 [63.6%]	1.00
Atrial fibrillation	6 [28.6%]	17 [38.6%]	0.58
**Clinical parameters**			
Ejection fraction (%)	23.2±9.2	25.7±7.7	0.10
Heart rate (bpm)	84.6±14.1	75.7±15.3	0.019
Cardiac output	4.2±1.1	3.9±1.1	0.30
Haemoglobin (g/dl)	12.1±2.2	12.8±2.5	0.14
Platelet count ($10^3/mm^3$)	216.4±96.7	206.3±79.3	0.45
Leukocyte count ($10^3/mm^3$)	8.2±3.9	9.0±3.0	0.13
AST/GOT (U/l)	43.3±82.0	33.1±22.5	0.37
ALT/GPT (U/l)	36.8±70.9	36.4±50.9	0.15
Glomerular filtration rate (ml/min)	65.1±21.1	63.5±26.0	0.95
Creatinine (μmol/L)	125.3±35.2	118.1±43.8	0.27
**Main medications (n [%])**			
β-blockers	0 [0%]	44 [100%]	
ARBs	2 [9.5%]	11 [25%]	0.19
ACEi	17 [80.9%]	32 [72.7%]	0.55
Other anti-hypertensive drugs	19 [90.5%]	43 [97.7%]	0.24
Statins	10 [47.6%]	19 [43.2%]	0.79
Antiarrhythmics	10 [47.6%]	18 [40.9%]	0.79
Antiplatelet therapy	11 [52.4%]	18 [40.9%]	0.43
All anticoagulants	15 [71.4%]	26 [59.1%]	0.42
**Other medications (n [%])**			
Insulin	6 [28.6%]	2 [4.5%]	0.011
Metformin	0 [0.0%]	4 [9.1%]	0.30
All anti-diabetic	7 [33.3%]	6 [13.6%]	0.10
Antianemics	2 [9.5%]	3 [6.8%]	0.65
Thyroid hormone replacement	0 [0.0%]	2 [4.5%]	1.00
COPD medication	4 [19.0%]	3 [6.8%]	0.20

Continuous data are shown as mean±standard deviation and categorical data as numbers (% of yes). Mann-Whitney U test was used for the statistical comparison of continuous variables and Fisher's exact test for the categorical variables.

Supplemental Table X. Major resources.

Animals

Species/Strain	Source	Background Strain	Sex
Adamts5Δcat; JAX stock no. 005771, B6.129P2-Adamts5tm1Dgen/J	The Jackson Laboratory	129P2/OlaHs backcrossed 8 times onto C57BL/6J, one additional backcross was performed to generate the WT littermates for experiments.	Male/female

Primary Antibodies

Target antigen	Vendor	Catalog #	Working concentration
Versican DPEAAE neoepitope	Abcam	ab19345	WB: 1μg/ml IHC: 10μg/ml
Full-length versican	Millipore	AB1033	WB: 0.5μg/ml IHC: 2.5μg/ml
Biglycan	Abcam	Ab94460	WB: 1μg/ml
Clusterin	Santa Cruz	sc-5289	WB: 1μg/ml
Decorin	Merck	SAB2100540	WB: 1μg/ml
Decorin	Santa Cruz	sc-22753	WB: 1μg/ml
Dermatopontin	Abcam	ab85162	WB: 2.5μg/ml
Mimecan	Abcam	ab110558	WB: 1μg/ml
Myosin light chain 3	Abcam	ab680	WB: 10μg/ml
Cardiac Troponin I	Abcam	ab47003	WB: 1μg/ml
Vimentin	Abcam	ab8069	WB: 1μg/ml
GAPDH	Sigma	G8795	WB: 0.05μg/ml
Galectin-1	Abcam	ab38328	WB: 1μg/ml
Serum amyloid P/SAP	Abcam	ab45151	WB: 0.05μg/ml
TGFβ	Abcam	ab179695	WB: 0.5μg/ml
Integrin beta 1	Abcam	ab52971	WB: 1.1μg/ml
Filamin A	Abcam	ab51217	WB: 3.3μg/ml
Connexin 43	Cell Signalling	3512	WB: n/a, 1:1000 of stock

Secondary Antibodies

Target species (label)	Vendor	Catalog #	Working concentration
Rabbit (HRP)	Jackson ImmunoResearch	211-032-171 (Lot # 130846)	WB: 80ng/ml
Mouse (HRP)	Jackson ImmunoResearch	115-035-174 (Lot # 129518)	WB: 80ng/ml
Goat (Alexa Fluor 594)	Invitrogen	A-21069	IHC: 2μg/ml

Materials

Name	Vendor	Catalog #
**1.Chemicals**		
2x Rapid Ligation Buffer	Promega	C671A
Acetonitrile	Fisher Scientific	10001334
Acetonitrile, 80% (aq), with 0.1% Formic Acid	Fisher Scientific	15431423
rhADAMTS5	R&D System	2198-AD
Angiotensin II human (AngII)	Sigma-Aldrich	A9525
Agar	Sigma-Aldrich	A1296
Bisoprolol Fumarate	British Farmacopoeia	1058
Cocktail of proteinase inhibitor	Thermo Scientific	51101
Chloroform	Sigma-Aldrich	C2432
Competent Cells (Max efficiency DH5α)	Invitrogen	18258-012
CutSmart Buffer	New England Biolabs	B72045
DMEM	Gibco	10938-025
EDTA	Sigma-Aldrich	E5134
EGTA	Sigma-Aldrich	E3889
Ethanol	Fisher Scientific	BP2818-500
FastAP Alkaline Phosphatase	Thermo Scientific	EF0654
Foetal Bovine Serum (FBS)	Gibco	10500-064
$H_2^{18}O$	Taiyo Nippon Salso	FO3-0027
Hydroxylamide, 50% (aq)	Sigma-Aldrich	438227
Iodoacetamine	Sigma-Aldrich	A3221
Gelatin	Sigma	G1393

Glycine	Fisher Bioreagents	BP381-1
GoTaq Hot Start Polymerase	Promega	D6001
Guanidine hydrochloride	Sigma-Aldrich	G3272
LB broth	Sigma-Aldrich	L3152
L-Glutamine	ThermoFisher	25030-024
Lipofectamine 2000 transfection reagent	ThermoFisher	11668019
Methanol	Fisher Chemical	M/4056/17
NuPAGE MOPS SDS Running Buffer (20X)	Life Technologies	NP0001-02
Pen-STREP	Gibco	15140-122
Phosphate-buffered saline (PBS), 10X	Severn Biotech Ltd	20-7400-10
pGEM-T vector	Promega	A362A
Phosho STOP	Roche	4906837001
Plasmid Safe	Lucigen	E3101K
Ponceau	Sigma	P7170-1L
Quiazol Lysis Reagent	Qiagen	79306
Sodium Acetate	Sigma-Aldrich	S2889
Sodium Azide	Sigma-Aldrich	S2002
Sodium Chloride	Sigma-Aldrich	S9888
Sodium dodecyl sulfate, SDS	Genomic Solution	80-0175
Sodium Citrate dihydrate	Sigma-Aldrich	W302600
Super Script VILO MasterMix	Invitrogen	11755050
SyberGreen	Applied Biosystem	4472908
T4 DNA ligase	Promega	M180A
TaqMan Universal PCR Master Mix	Applied Biosystem	4324018
Triethylammonium bicarbonate (TEAB)	Sigma-Aldrich	T7408
Trifluoroacetic acid (TFA)	Thermo Scientific	85183
Thiourea	Supelco	107979
Trypsin-EDTA (0.05%)	Gibco	25300-054
Tris-hydrochloride (Tris-HCl)	Sigma-Aldrich	T3253
Triton X-100	Sigma-Aldrich	X100
Trizma base	Sigma-Aldrich	T1503
Tween-20	Sigma-Aldrich	P7949
Urea	Fisher Scientific	11337898
Water, with 0.1% Formic Acid	Fisher Scientific	10188164
X-gal	Promega	V3941
**2. Enzymes**		
α2-3,6,8,9-neuroaminidase (Sialidase)	EDM Millipore	362280 (KP0012)
β1,4-galactosidase	EDM Millipore	362280 (KP0004)
β-N-acetylglucosaminidase	EDM Millipore	362280 (KP0013)
Chrondroitinase ABC	Sigma-Aldrich	C3667
Endo-α-N-acetylgalactosaminidase (O-glycosidase)	EDM Millipore	362280 (KP0011)
Heparinase II	Sigma-Aldrich	H6512
Keratanase	Sigma-Aldrich	G6920
NheI	New England Biolabs	R0131S
PNGaseF (N-Glycosidase)	EDM Millipore	362280 (KP0001)
SalI	New England Biolabs	R3138S
Trypsin, MS Grade	Thermo Scientific	90057
**3. Reagent Kits**		
GeneJet Gel Extraction and DNA Cleanup Micro Kit	Fisher Scientific	13293149
High pH Reversed-Phase Peptide Fractionation Kit	Thermo Scientific	84868
KAPA HiFi HotStart ReadyMix	Roche	7958927001
NuPAGE 4-12% Bis-Tris gels	Thermo Scientific	NP0336BOX
miRNeasy Mini kit	Qiagen	4053228006220
QIAprep Spin Miniprep kit	Qiagen	27104
TMT10plex Isobaric Label Reagent Set	Thermo Scientific	90406

Primers

Target	Vendor	Sequence	Species
Vcan isoform V0	Integrated DNA Technologies	fw: AAGACAGGTCGATTGAGTGATATG<br>rv: CGGGTAAAATTTCAGCAAACA	Mus Musculus
Vcan isoform V1	Integrated DNA Technologies	fw: GAGGTCTACTTGGGGTGAGAAC<br>rv: TCACTCAATCGACGTTTAAAGCA	Mus Musculus

Vcan isoform V2	Integrated DNA Technologies	fw: AGAGAAAACAAGACAGGACCTGAT<br>rv: CAGGTGCACACATAGGAAGTCT	Mus Musculus
Vcan isoform V3	Integrated DNA Technologies	fw: CCCAGTGTGGAGGAGGTCTA<br>rv: CAGAGATCAGGTCGTTTAAAGC	Mus Musculus
Gapdh	Integrated DNA Technologies	fw: AGGTCGGTGTGAACGGATTTG<br>rv: GGGGTCGTTGATGGCAACA	Mus Musculus
Lum	Integrated DNA Technologies	fw: GAACTGGCTGATAGCGGGGT<br>rv: CAGTGGTCCCAGGATCTTACA	Mus Musculus
Adamts-5	Integrated DNA Technologies	fw: TGCCCACCCAATGGTAAATCTT<br>rv: CAGGACACCTGCATATTTGGGA	Mus Musculus
Ogn	Integrated DNA Technologies	fw: CTGACTGCCAAGGATTTTGC<br>rv: AGCTTTGGAGGAAGAACTGGA	Mus Musculus
Aspn	Integrated DNA Technologies	fw: TGGAAGACACAGATGATGACGA<br>rv: TTGGAACCGATGTCAGACCT	Mus Musculus
Bgn	Integrated DNA Technologies	fw: TGGAAGACACAGATGATGACGA<br>rv: TTGGAACCGATGTCAGACCT	Mus Musculus
Prelp	Integrated DNA Technologies	fw: AGAGAAAATCAACGGGACCCAG<br>rv: AAAATTTCCATCCAGCCGCAG	Mus Musculus
Postn	Integrated DNA Technologies	fw: ACCTGGATTCTGACATTCGCA<br>rv: TGCCGTGTTTCAGGTCCTTG	Mus Musculus
Ddr2	Integrated DNA Technologies	fw: CTGATTGGTTGCTTGGTGGC<br>rv: TTGAACATGCTGGACTCGCT	Mus Musculus
Hyal1	Integrated DNA Technologies	fw: GGCTTCCCTGACTGCTACAA<br>rv: ACCCTAGCTGGTCGTTCTGA	Mus Musculus
Hyal2	Integrated DNA Technologies	fw: TGAGCCAGGTGGACCTTATC<br>rv: CTTGAAGCGTCTTCCGAGTC	Mus Musculus
Hyas1	Integrated DNA Technologies	fw: GGGTCAGCTTCTTGAGCAGT<br>rv: ACCACTGATGCAGGACACAC	Mus Musculus
Hyas2	Integrated DNA Technologies	fw: CCCTGGGATTAAAGTCGTGA<br>rv: TTGTCCCTGCCCATAACTTC	Mus Musculus
Hyas3	Integrated DNA Technologies	fw: CGGGTGAAGGAGAGACAGAG<br>rv: GCATGATGCATGAGAAGGTG	Mus Musculus
Vcan isoform V0	Integrated DNA Technologies	fw: CCAGCAAGCACAAAATTTCA<br>rv: TTGGATGACCAATTACACTCAAA	Homo Sapiens
Vcan isoform V1	Integrated DNA Technologies	fw: TGAGAACCAGACAGGCTTCC<br>rv: TTGGATGACCAATTACACTCAAA	Homo Sapiens
Vcan isoform V2	Integrated DNA Technologies	fw: CCAGCAAGCACAAAATTTCA<br>rv: GTTAAGGCACGGGTTCATTTT	Homo Sapiens
Vcan isoform V3	Integrated DNA Technologies	fw: CTTGGGGTGAGAACCCTGTA<br>rv: CATTTTGCAGCGATCAGGT	Homo Sapiens

Taqman Probes

Target	Vendor	Assay	Species
ADAMTS5	ThermoFisher	Hs00199841_m1	Homo Sapiens
LUM	ThermoFisher	Hs00929860_m1	Homo Sapiens
OGN	ThermoFisher	Hs00247901_m1	Homo Sapiens
BGN	ThermoFisher	Hs00959141_g1	Homo Sapiens
COL1A1	ThermoFisher	Hs00164004_m1	Homo Sapiens
COL3A1	ThermoFisher	Hs00943809_m1	Homo Sapiens
GAPDH	ThermoFisher	Hs02786624_g1	Homo Sapiens
Gapdh	ThermoFisher	Mm99999915_g1	Mus musculus
Cd68	ThermoFisher	Mm03047343_m1	Mus musculus
Lgals3	ThermoFisher	Mm00802901_m1	Mus musculus
